# Discovery of gramicidin A analogues with altered activities by multidimensional screening of a one-bead-one-compound library

**DOI:** 10.1038/s41467-020-18711-2

**Published:** 2020-10-01

**Authors:** Yuri Takada, Hiroaki Itoh, Atmika Paudel, Suresh Panthee, Hiroshi Hamamoto, Kazuhisa Sekimizu, Masayuki Inoue

**Affiliations:** 1 Graduate School of Pharmaceutical Sciences, The University of Tokyo, 7-3-1 Hongo, Bunkyo-ku, Tokyo, 113-0033 Japan; 2 Teikyo University Institute of Medical Mycology, 359 Otsuka, Hachioji, Tokyo, 192-0395 Japan

**Keywords:** Peptides, Natural product synthesis, Solid-phase synthesis, Chemical libraries, Screening

## Abstract

Gramicidin A (**1**) is a peptide antibiotic that disrupts the transmembrane ion concentration gradient by forming an ion channel in a lipid bilayer. Although long used clinically, it is limited to topical application because of its strong hemolytic activity and mammalian cytotoxicity, likely arising from the common ion transport mechanism. Here we report an integrated high-throughput strategy for discovering analogues of **1** with altered biological activity profiles. The 4096 analogue structures are designed to maintain the charge-neutral, hydrophobic, and channel forming properties of **1**. Synthesis of the analogues, tandem mass spectrometry sequencing, and 3 microscale screenings enable us to identify 10 representative analogues. Re-synthesis and detailed functional evaluations find that all 10 analogues share a similar ion channel function, but have different cytotoxic, hemolytic, and antibacterial activities. Our large-scale structure-activity relationship studies reveal the feasibility of developing analogues of **1** that selectively induce toxicity toward target organisms.

## Abstract (web summary)

The strong hemolytic activity and mammalian cytotoxicity of gramicidin A, a peptide antibiotic, has hindered its non-topical clinical application. Here, the authors report a high-throughput strategy for the discovery of gramicidin A analogues with altered biological activity profiles.

## Introduction

Biologically active natural products have long been regarded as invaluable sources of inspiration for drug design, with particular effectiveness against infectious diseases and cancer^1–6^. The structures of these natural products were obviously optimized through evolutionary selection for the benefit of the host, and not for their safety or efficacy in humans. Thus, the discovery of selective therapeutic agents usually requires both the generation and biological evaluation of natural-product analogues. Such structure–activity relationship (SAR) studies can be used to pinpoint structural factors essential for a specific activity, and to clarify a natural product’s molecular mode of action. Total synthesis is a highly versatile and powerful approach for analogue preparation because it allows for deep-seated structural modifications of the parent structure^7,8^. One-by-one total synthesis of a wide variety of structurally complex analogues, however, is time-consuming and impractical for fully exploring the potential of natural products. Therefore, a new high-throughput strategy for rapidly preparing and evaluating a large number of natural product-based compounds should accelerate the acquisition of SAR information and the development of ideal pharmaceuticals^9,10^.

**Fig. 1:**
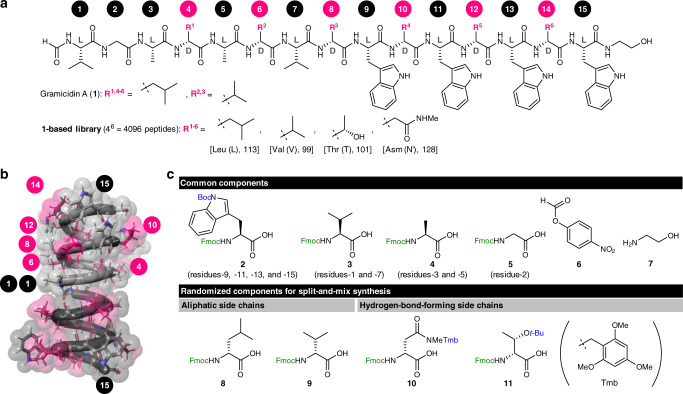
Structures of compounds used in this study. **a** Structures of gramicidin A (**1**) and prepared analogues. Both the three-letter and one-letter codes of the amino acids, and molecular weights of the residues, are displayed in brackets. d-Asm (N′) d-*N*_γ_-methylaspargine. **b** A three-dimensional structure of the head-to-head dimer of the β^6.3^-helix of **1** (PDB ID: 1MAG). The substituted aliphatic residues are highlighted in pink. **c** Synthetic component amino acids of the **1**-based OBOC library. Boc *tert*-butoxycarbonyl, Fmoc 9-fluorenylmethoxycarbonyl, *t*-Bu *tert*-butyl, Tmb 2,4,6-trimethoxybenzyl.

Gramicidin A (**1**, Fig. 1a), discovered in 1939 from the soil bacterium *Bacillus brevis*^11,12^, was the first antibiotic to be manufactured commercially^13,14^. This peptidic natural product displays potent broad-spectrum antibiotic activity against Gram-positive strains, even multidrug-resistant strains^15^. Despite being an efficient antibiotic, **1** has the disadvantage of high hemolytic activity^16^. Therefore, this molecule cannot be administered internally as a systemic antibiotic and is rather used as an ingredient in topical antibiotics for the treatment of infected surface wounds, and eye, nose, and throat infections. Moreover, because of its high toxicity toward mammalian cancer cells, **1** has potential value as an anticancer agent^17^.

The linear 15-mer peptide sequence of **1** (molecular weight = 1882 Da) is composed of alternating d- and l-amino acids, except for the Gly at residue-2, and is blocked with a formyl group at the N-terminus and with 2-aminoethanol at the C-terminus^18^. Its large structure is highly hydrophobic and charge neutral due to its aliphatic or aromatic amino acid components and end-capped termini. The chirality-alternating sequence makes **1** less susceptible to proteolytic degradation^19^, thus presenting major advantage over conventional peptide therapeutics. Owing to the alternating stereochemistry, all the hydrophobic side chains of **1** are positioned at one side of the strand in the hydrophobic interior of a lipid bilayer membrane. It thereby folds into a helix with 6.3 residues per turn, i.e., a β^6.3^-helix (Fig. 1b)^20–24^. The folded monomers dimerize head-to-head to form a hollow tubular structure spanning over 26 Å with a 4 Å diameter pore^25^. This nanotube functions as a transmembrane channel that allows for facile diffusion of monovalent cations (e.g., H^+^, Na^+^, and K^+^)^26^. The resulting disruption of transmembrane ion concentration gradients is believed to contribute to the antibacterial and cytotoxic activities of **1**.

Since its discovery more than 80 years ago, the unique structure and function of **1** has attracted considerable interest from the scientific community. Many effort has been made by chemists to investigate or control the ion channel function of **1**^27–30^, collectively leading to the generation of approximately 350 artificial analogues (counted by SciFinder, 27 March 2020). Although these studies provided valuable information on the structure and channel function of **1**, only a few analogues have been identified to display different bioactivities from those of **1**^15,31–34^. Because available SAR data are limited, it remains unclear how the ion channel function of **1** relates to its antibacterial and cytotoxic activities. Extensive exploration and biological evaluation of the analogues of **1** are necessary for developing lead compounds for systemic antibiotics or anticancer agents^35–37^.

To rapidly discover peptides with the desired pharmacological profiles, we decided to synthesize over 4000 analogues of **1**, more than 10-fold the total number prepared in the last 80 years, and envisioned developing new multidimensional high-throughput assays. We are particularly interested in altering the activity profiles of the parent natural product **1** with minimal perturbation of its physicochemical properties.

Here we report the design and construction of a **1**-based library comprising 4096 peptides, and the development of the three-assay system for evaluating the ion transport, cytotoxic, and antibacterial activities. The SAR studies of thousands of strategically designed analogues of **1** lead us to find 10 ion channel-forming analogues with distinct profiles for antibacterial, hemolytic, and cytotoxic activities, and to elucidate the structural elements are important for modulating their activity profiles.

## Results

### Design of the gramicidin A-based OBOC library

To realize high-throughput synthesis and evaluation of thousands of analogues of **1**, we adopted a one-bead-one-compound (OBOC) strategy rather than a one-by-one approach^38–41^. This strategy utilizes solid-phase peptide synthesis (SPPS) to construct the library and rapidly diversifies the structures using split-and-mix randomization. Importantly, as each bead carries a unique sequence, all the spatially separated compounds on the beads can be structurally determined and functionally assayed in a concurrent, yet independent, manner.

Because the antibacterial and cytotoxic activities of **1** are thought to relate to its ion transport function, the OBOC library of **1** was designed in such a way that it would not to disturb the nanotube formation in a hydrophobic lipid bilayer (Fig. 1a). First, the d,l-alternating chiralities were maintained to uphold the folding propensity, and the capping functionalities at the termini of the sequence were kept to retain the charge-neutral nature. Second, residues-1 and -2^42,43^, and all the Trp residues (residues-9, -11, -13, and -15)^44,45^, were to remain because of their pivotal roles in the channel activity. These considerations narrowed down the potential randomization sites to 9 of the 15 residues. Among these nine residues, we deselected the three l-configured residues and selected all the six d-configured residues to realize highly randomized sequences without preparing components having both l- and d-chiralities. Hence, four d-Leu residues (residues-4, -10, -12, and -14) and two d-Val (residues-6 and -8) were determined as the substitution sites.

The substituting residues for the **1**-based OBOC library were selected such that the overall hydrophobic and charge-neutral character was maintained, but the hydrogen-bonding ability was changed (Fig. 1a). Accordingly, d-*N*_γ_-methylaspargine (d-Asm)^46^ and d-Thr were adopted together with the original d-Leu and d-Val. Whereas the side chains of d-Asm and d-Thr emulate the molecular shapes of the branched aliphatic chains of d-Leu and d-Val, respectively, the amide group of d-Asm and the hydroxy group of d-Thr allow for hydrogen-bonding interactions between residues or with surrounding molecules. Moreover, the *N*_γ_-methyl and β-methyl groups of these amino acids compensate for the hydrophilicity of the polar functional groups and thus help to retain the hydrophobicity of the analogues of **1**.

Randomization of six sites (residues-4, -6, -8, -10, -12, and -14) using four amino acids (d-Leu, d-Val, d-Asm, and d-Thr) would generate a library comprising 4096 (=4^6^) peptides. This library contains the parent peptide sequence of **1** to serve as a positive control in the biological screening. The 4096 sequences can be structurally determined simply by tandem mass spectrometry (MS/MS) because each randomized amino acid residue at the same residue number has a unique mass unit (113 for Leu, 99 for Val, 101 for Thr, and 128 for Asm, Fig. 1a). Hence, one sequence on one bead can be decoded by a unique fragmentation pattern.

To construct the **1**-based OBOC library, we used Fmoc-based SPPS. Thus, all the amino acid components were in their *N*_*α*_-Fmoc-protected forms (**2**–**5** and **8**–**11**, Fig. 1c). The side chains of the components were blocked by acid-labile protective groups to avoid potential side reactions or interchain aggregation, which decreases the coupling efficiency of the amino acids. Specifically, the enamine group of l-Trp, the hydroxy group of d-Thr, and the *N*_γ_-methylamide group of d-Asm^47^ were protected with Boc, *t*-Bu, and 2,4,6-trimethoxybenzyl (Tmb) groups^48^, respectively.

### Optimization of the synthesis and evaluation of gramicidin A

Our OBOC strategy necessitated high-yielding synthesis of **1** and its analogues using beads, structural determination and multidimensional functional analyses of peptides derived from a single bead. To validate the strategy, we aimed to establish an SPPS of the parent **1** and MS/MS-based structural determination in microscale prior to constructing the OBOC library. Furthermore, we comprehensively characterized wide spectrum of activity profile of **1**, selected a set of three representative functions, and devised the microscale assay protocols to evaluate the three functions.

**Fig. 2:**
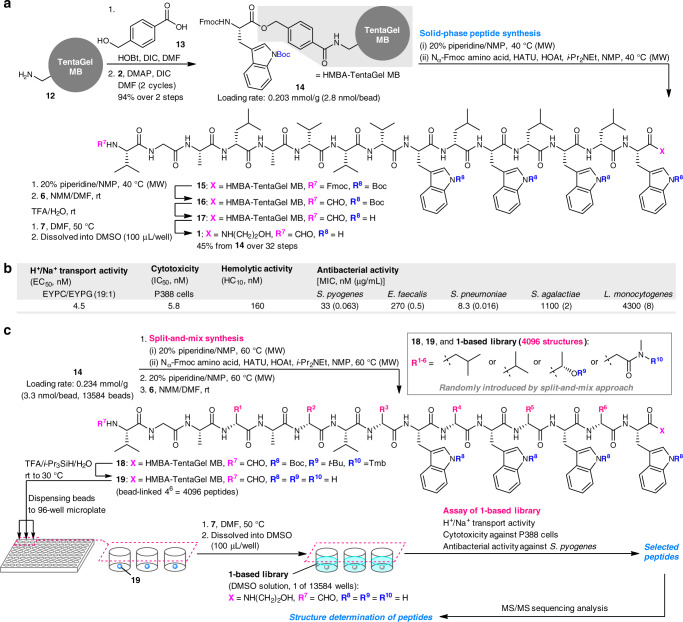
Pilot study and construction of the **1**-based OBOC library. **a** SPPS of **1** from TentaGel macrobeads **12**. **b** H^+^/Na^+^ transport, cytotoxic, hemolytic, and antibacterial activities of **1**. **c** A high-throughput platform for constructing and screening 4096 gramicidin A analogues. DIC *N*,*N*′-diisopropylcarbodiimide, DMAP *N*,*N*-dimethyl-4-aminopyridine, HATU *O*-(7-aza-1*H*-benzotriazol-1-yl)-*N*,*N*,*N*′,*N*′-tetramethyluronium hexafluorophosphate, HMBA hydroxymethylbenzoic acid, HOAt 1-hydroxy-7-azabenzotriazole, HOBt 1-hydroxybenzotriazole, HC_10_ concentration causing 10% hemolysis, MIC minimum inhibitory concentration, MW microwave, NMM *N*-methylmorpholine, TentaGel MB TentaGel macrobeads, TFA trifluoroacetic acid, EC_50_ median effective concentration, EYPC egg yolk phosphatidylcholine, EYPG egg yolk phosphatidylglycerol, IC_50_ median inhibitory concentration. MICs (μg/mL) were determined by the microdilution method. *S. pyogenes* *Streptococcus pyogenes* SSI-9, *E. faecalis* *Enterococcus faecalis* JCM5803, *S. pneumoniae* *Streptococcus pneumoniae* NBRC 102642, *S. agalactiae* *Streptococcus agalactiae* JCM5671, *L. monocytogenes* *Listeria monocytogenes* 10403S.

TentaGel^49^ macrobeads (TentaGel MB, 0.3 mm in diameter, **12**) were utilized as a solid support for the Fmoc-based SPPS of **1** (Fig. 2a)^50,51^. An acid-stable hydroxymethylbenzoic acid (HMBA, **13**) linker^52^ was adopted to conjugate the peptide and the bead because it enabled C-terminal modification by 2-aminoethanol (**7**) as the last step after removing the acid-sensitive protective groups.

The first step of the SPPS of **1** was the condensation of HMBA **13** with the amine of the TentaGel MB **12** (Fig. 2a). Subsequent esterification with Fmoc-l-Trp-OH **2** provided Fmoc-l-Trp(Boc)-loaded HMBA-TentaGel MB (**14**, 2.8 nmol per bead) in 94% yield over two steps. The thus-obtained beads were submitted to microwave-assisted SPPS at 40 °C to facilitate synthesis^53–55^. Specifically, pentadecapeptide **15** was elongated from **14** by 14 cycles of *N*_α_-deprotection with piperidine, and amidation of Fmoc amino acids (**2**, **3**, **4**, **5**, **8**, and **9**) in the presence of 1-hydroxy-7-azabenzotriazole (HOAt) and *O*-(7-aza-1*H*-benzotriazol-1-yl)-*N*,*N*,*N*′,*N*′-tetramethyluronium hexafluorophosphate (HATU)^56^. After Fmoc removal from **15**, the liberated terminal amine was formylated using *p*-nitrophenyl formate (**6**) to afford **16**. The Boc groups at the four Trp residues of **16** were then simultaneously removed using TFA/H_2_O to provide **17**. Finally, C-terminal modification through ester-amide exchange was attained by treating **17** with **7** in DMF, releasing crude **1** from the resin. Ultrahigh performance liquid chromatography (UHPLC) analysis revealed a high overall yield (45%, 32 steps) of **1** from **14**. Consequently, a single bead was calculated to carry 2.7 μg of **1**, which was dissolved in 100 μL DMSO in one well of a 96-well plate and used as a crude solution (15 μM of **1**) for microscale structural determination and assays. We found that 10% or less of the single bead-derived **1** (0.3 μg) was sufficient for complete MS/MS sequencing of 15-mer **1**.

Prior to devising the miniaturized assay protocols, various activities of **1** were assessed (Fig. 2b). First, we evaluated the ion channel function of **1** using liposomes. To mimic a negatively charged Gram-positive bacterial membrane, the liposomes were prepared as large unilamellar vesicles (LUVs) comprising a 19:1 mixture of egg yolk phosphatidylcholine (EYPC) and egg yolk phosphatidylglycerol (EYPG)^57^. Pyranine, a pH-dependent fluorescent dye^58^, was encapsulated in the LUVs and a pH gradient was applied across the membrane. H^+^/Na^+^ transport induced by a peptide was monitored by changes in the fluorescence intensity of pyranine. By measuring the fluorescence changes (%) caused by varied concentrations of **1**, we quantified the half-maximal response (EC_50_) value as 4.5 nM.

Second, we examined the cytotoxicity toward mammalian cancer cells and hemolytic activity of **1**. We determined the median inhibitory concentration (IC_50_) against P388 mouse leukemia cells to be 5.8 nM using the 4-[3-(4-iodophenyl)-2-(4-nitrophenyl)-2*H*-5-tetrazolio]-1,3-benzene disulfonate colorimetric method^59^. Hemolytic activity was evaluated by monitoring hemoglobin leakage from rabbit red blood cells as a consequence of membrane damage. The concentration of **1** required to cause 10% hemoglobin leakage (HC_10_) was 160 nM. Thus, the P388 cytotoxicity of **1** was 28-fold more sensitive than its hemolytic activity.

Third, **1** was subjected to five pathogenic Gram-positive bacteria. The minimum inhibitory concentrations (MIC) of **1** were 33 nM (*Streptococcus pyogenes*), 270 nM (*Enterococcus faecalis*), 8.3 nM (*Streptococcus pneumoniae*), 1100 nM (*Streptococcus agalactiae*), and 4300 nM (*Listeria monocytogenes*). The antibacterial activities of **1** varied significantly among strains and *S. pyogenes* and *S. pneumoniae* were found to be the most susceptible strains.

These experiments confirmed that the parent natural product **1** possesses a wide range of functions. We selected a set of three different assays to functionally evaluate the **1**-based OBOC library, including H^+^/Na^+^ transport activity, cytotoxicity against P388 cells, and antibacterial activity against *S. pyogenes*, because **1** displayed high potencies in these assays. Each type of assay must be simplified and miniaturized to assess the activities of the library peptides in a 96-well format for the high-throughput screening. Therefore, we were to adopt one-point assays for the ion transport and cytotoxic activities, in which the threshold concentrations were set to an EC_55.2_ value of **1** (5.9 nM) and an IC_99.6_ value of **1** (30 nM), respectively. Alternatively, we planned to apply three concentrations (380, 94, and 23 nM) around the MIC value of **1** to *S. pyogenes*. These microscale experimental protocols enabled us to perform three different assays using less than 2% of the peptide derived from a single bead.

### Construction of the gramicidin A-based library

According to the pilot study using **1**, the OBOC strategy encompassed five stages (Fig. 2c): (1) parallel solid-phase syntheses of 4096 peptides using the split-and-mix approach; (2) H^+^/Na^+^ ion transport assay; (3) P388 cytotoxicity assay; (4) *S. pyogenes* antibacterial activity assay; and (5) structural determination. To facilitate screening, the positive compounds were chosen according to the most sensitive one-point assay in the second stage. They were then classified on the basis of the one-point assay results in the third stage. The selected compounds from the second and third stages would be submitted to more elaborate experiments in the fourth and fifth stages to search for artificial peptides with distinct structures and activity profiles.

To construct the **1**-based OBOC library of 4096 peptides (Fig. 2c), a threefold greater number of Fmoc-l-Trp(Boc)-conjugated beads (**14**, 13,584 beads) was used to maximize the coverage of this library while minimizing operational burden. In this way, sequences that are found one or more times (appearance frequency ≥1) cover at least 96% of the OBOC library^60^. The reagents and conditions for amidation, and modification of the N- and C-termini were the same as those developed for the route from **14** to **1**, while split-and-mix methods were employed to randomize the six residues. Thus, upon elongation of residues-4, -6, -8, -10, -12, and -14, the beads were divided into four pools that were separately deprotected and condensed with the 4 Fmoc amino acids (**8**, **9**, **10**, and **11**). All the beads were then mixed and subjected to the next attachment of the original component. The combination of randomizations and non-randomizations furnished the bead-linked 15-mer peptide **18** with the N-terminal formyl group. Simultaneous detachment of the three types of groups (Boc, *t*-Bu, and Tmb) of **18** was achieved by TFA/H_2_O treatment in the presence of *i*-Pr_3_SiH^61^. The obtained 13,584 beads **19** were dispensed to 13,584 wells of 155 96-well plates. In each well, the ester-amide exchange using a solution of **7** in DMF detached the solid support, liberating the peptide of the **1**-based library. After removal of DMF and **7** under vacuum, DMSO (100 μL) was added to furnish the 13,584 peptide solutions comprising 4096 structures.

### Three functional assays and MS/MS sequencing of the peptides

**Fig. 3:**
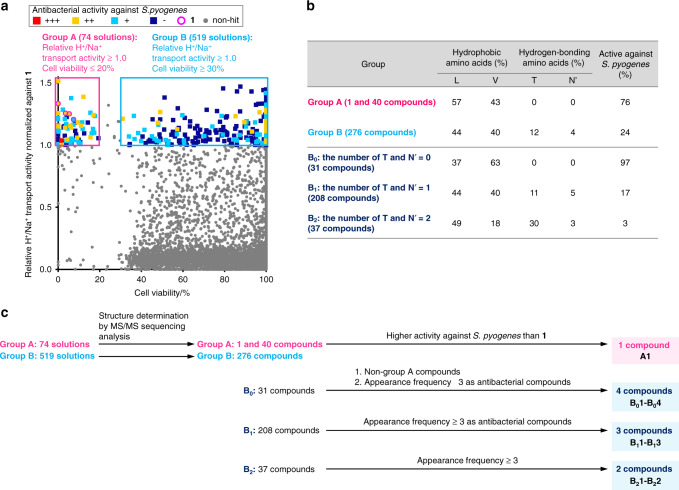
Evaluation and classification of the **1**-based OBOC library. **a** The scatter plot for the three different assays of the bead-derived 13,584 peptide solutions. H^+^/Na^+^ transport activities of the peptides were normalized against the activity of **1**. Cell viability (%) was evaluated using the P388 mouse leukemia cell line. Antibacterial activity against *S. pyogenes* was evaluated using the three concentrations and the potency is coded by three colors. +++ (red square): inhibition by the 640-fold diluted peptide solution, ++ (yellow square): inhibition by the 160-fold diluted peptide solution, + (cyan square): inhibition by the 40-fold diluted peptide solution, - (navy square): no inhibition. The dots denoting the parent **1** are indicated by the purple circles. The dots that did not meet any criteria are shown as gray filled circles. Source data are provided as a Source Data file. **b** Distribution percentages of the randomized four amino acids and percentages of the active peptides against *S. pyogenes*. Amino acids are displayed as one-letter codes (Asm = N′). Compounds were defined as active if one or more solutions was active in the antibacterial assay. **c** Selection criteria of the 10 peptides (**A1**, **B**_**0**_**1**–**B**_**0**_**4**, **B**_**1**_**1**–**B**_**1**_**3**, and **B**_**2**_**1**–**B**_**2**_**2**) from groups A and B.

First, we evaluated the H^+^/Na^+^ transport activity and cytotoxicity against P388 cells using all 13,584 bead-derived peptide solutions (Fig. 3a). The data for the two activities of each peptide were plotted as a dot in a graph where the X- and Y-axes represent the viability of P388 cells and the relative H^+^/Na^+^ transport activity normalized against **1**, respectively. As a result, 600 of 13,584 peptide solutions exhibited more potent transport activity (≥1) than the parent **1**. These 600 peptide solutions were further categorized according to their toxicity against P388 cells. The more toxic (cell viability ≤20%) and less toxic (cell viability ≥30%) peptides were designated as group A (74 solutions) and group B (519 solutions), respectively.

Next, the peptides in groups A and B were subjected to the antibacterial assay and structural determination. Three peptide concentrations were applied to evaluate the growth inhibition of *S. pyogenes* and determine the antibacterial activity. According to the assay results, the activities of the peptides were sorted into four groups with decreasing potency [+++ (red square), ++ (yellow), + (cyan), and - (navy), Fig. 3a]. Finally, the structures of the peptides were determined. Upon MS/MS analysis, the peptides of groups A (74 solutions) and B (519 solutions) afforded clear parent ions and fragmentation patterns, which permitted us to unambiguously establish their structures. Consequently, unique structures of 41 peptides of group A and 276 peptides of group B were identified (Fig. 3b and Supplementary Tables 1–10). Of the 41 compounds, one in group A turned out to be the parent **1**. In fact, as displayed in Fig. 3a (purple circles), four peptide solutions (appearance frequency = 4) that were found to be active in all three assays corresponded to the structure of **1**. These results demonstrated the high reliability of the split-and-mix synthesis and the three-assay system to select active molecules.

Having identified the hit peptides in the three-assay system, we examined their structural features based on their amino acid compositions (Fig. 3b). Analysis of the distribution of the four randomizing amino acids [d-Leu (L), d-Val (V), d-Thr (T), and d-Asm (N′)] revealed that residues-4, -6, -8, -10, -12, and -14 of the 41 peptides of group A carries 57% of L, 43% of V, and 0% of T/N′. Thus, the peptides of group A mimic the hydrophobic sequence of **1**, which contains only L (67%) and V (33%). In contrast, the 276 artificial peptides of group B possess all four monomers, L (44%), V (40%), T (12%), and N′ (4%). For subsequent analyses, we further divided group B into three subgroups according to the number of hydrogen bond-forming amino acids (T and N′); subgroups B_0_ (31 compounds, T and N′ = 0), B_1_ (208 compounds, T and N′ = 1), and B_2_ (37 compounds, T and N′ = 2). Interestingly, no peptide sequence with three or more T/N′ residues was identified in group B.

We also analyzed the characteristics of groups A and B by estimating the percentage of antibacterial peptides in each group (Fig. 3b). Whereas 76% of group A showed antibacterial activity against *S. pyogenes*, only 24% of group B comprised active compounds. Intriguingly, in group B, the order of the active percentages was B_0_ (97%), B_1_ (17%), and B_2_ (3%). Taken together, these data on the amino acid distribution and activities allowed us to uncover SAR information for **1** that was previously inaccessible. Considering the small percentages of T and N′ in the 317 sequences of groups A and B, high levels of hydrophobicity are critical for the peptides to promote the H^+^/Na^+^ ion transport across the liposomal membrane. The characteristics of group A and subgroup B_0_ indicated the importance of the aliphatic amino acids (L and V) for the antibacterial activity, whereas the higher content of the more hydrophobic L residues (57% for group A and 37% for subgroup B_0_) appeared to be beneficial for the potent cytotoxicity.

### Selection of the 10 analogues from groups A and B

To allow for detailed individual analyses of the structure and activity of the promising peptides, we selected 10 analogues (**A1**, **B**_**0**_**1**–**B**_**0**_**4**, **B**_**1**_**1**–**B**_**1**_**3**, **B**_**2**_**1**, and **B**_**2**_**2**) from the 316 peptides to represent key structural and functional features of each group (Fig. 3c). **A1** was the sole compound of the 40 artificial analogues of group A that displayed higher antibacterial activity (+++) than the parent **1** (++ or +). **B**_**0**_**1**–**B**_**0**_**4** in subgroup B_0_ and **B**_**1**_**1**–**B**_**1**_**3** in subgroup B_1_ were the peptides found three or more times as antibacterial compounds in the screening and not found as group A peptides. **B**_**2**_**1** and **B**_**2**_**2** were the only two peptides with an appearance frequency of three or more in subgroup B_2_. Neither of these peptides exhibited growth inhibition activity against *S. pyogenes*.

### Synthesis and functional evaluation of the 10 selected analogues

**Fig. 4:**
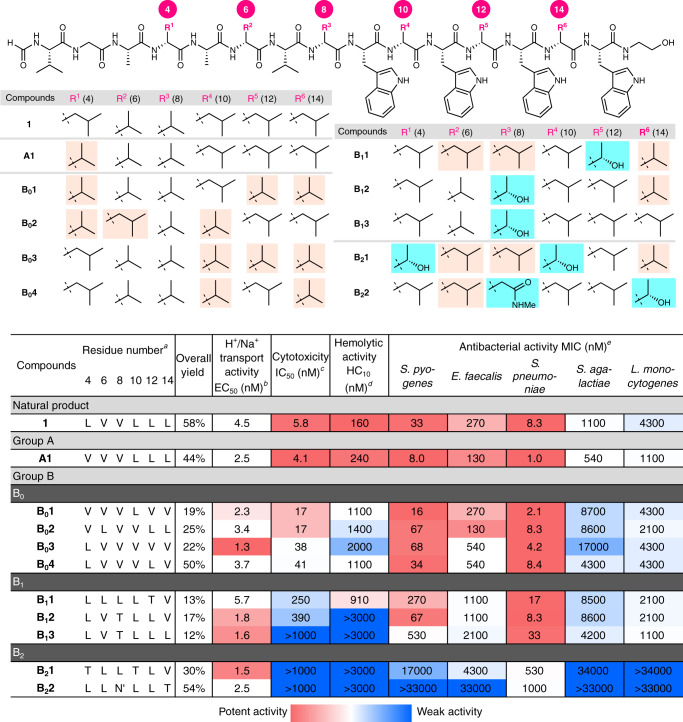
Structures and four activities of the 10 selected analogues of gramicidin A. ^a^Amino acids are displayed as one-letter codes. ^b^EC_50_ value of H^+^/Na^+^ transport activity toward the pH-gradient large unilamellar vesicles consisting of EYPC and EYPG (19:1). Upper and lower limits of the color gradient are 1000 nM (blue) and 1.3 nM (red), respectively. ^c^IC_50_ value against the P388 mouse leukemia cell line. Upper and lower limits of the color gradient are 1000 nM (blue) and 4.1 nM (red), respectively. ^d^HC_10_ value against the rabbit red blood cells. Upper and lower limits of the color gradient are 3000 nM (blue) and 160 nM (red), respectively. ^e^MIC values (nM) were determined by the microdilution method. Upper and lower limits of the color gradient are 33,000 nM (blue) and 8.0 nM (red), respectively.

The structural differences of **A1**, **B**_**0**_**1**–**B**_**0**_**4**, **B**_**1**_**1**–**B**_**1**_**3**, **B**_**2**_**1**, and **B**_**2**_**2** from the parent **1** are illustrated in Fig. 4. In order to conduct multiple functional analyses at larger scales, these 10 artificial structures were separately prepared under the same conditions used for the preparation of **1**, except for the resin (Fig. 2a). The 15-mer sequences were elongated from Fmoc-l-Trp(Boc)-HMBA ChemMatrix resin^62^ and functionalized at their N- and C-termini. The subsequent HPLC purification delivered milligrams of the analogues **A1**, **B**_**0**_**1**–**B**_**0**_**4**, **B**_**1**_**1**–**B**_**1**_**3**, **B**_**2**_**1**, and **B**_**2**_**2** in high overall yields over 32 steps (12–58%).

The sufficient supply of materials allowed us to accurately measure the EC_50_ values (nM) of H^+^/Na^+^ transport function in the LUVs of EYPC and EYPG, the IC_50_ values (nM) of cytotoxicities against P388 cells, the HC_10_ values (nM) of hemolytic activities against red blood cells, and the MIC values (nM) of antibacterial activities against five Gram-positive strains. Each compound in group A, and subgroups B_0_, B_1_, and B_2_ is listed in descending order of its cytotoxicity in Fig. 4. The potency of the peptides is shown in a color gradient heat map.

The H^+^/Na^+^ transport, P388 cytotoxic, and *S. pyogenes* antibacterial activities of **A1**, **B**_**0**_**1**–**B**_**0**_**4**, **B**_**1**_**1**–**B**_**1**_**3**, **B**_**2**_**1**, and **B**_**2**_**2** were in accordance with those of the miniaturized assays used to assess the OBOC library. These results thus corroborated the robustness of our selection method. Reflecting the low threshold concentration in the screening, all 10 analogues displayed potent H^+^/Na^+^ transport activities with EC_50_ values of single-digit nanomolar concentrations (1.3–5.7 nM). Compared with the parent **1** (EC_50_ = 4.5 nM), their EC_50_ numbers ranged from 0.29-fold at a minimum (**B**_**0**_**3**) and 1.3-fold at a maximum (**B**_**1**_**1**). Negligible liposomal membrane-disrupting activity was observed for the 10 analogues at nanomolar concentrations (Supplementary Fig. 1), supporting the assumption that they form ion channels in a membrane to exchange H^+^/Na^+^. We confirmed the channel structures of the analogues by their circular dichroism spectra in the LUVs (Supplementary Fig. 2). Similar to **1**, all 10 compounds in the lipid bilayer produced a positive peak at 220 nm, which is characteristic of the ion-conducting β^6.3^-helical conformation.

As expected from the way they were grouped and selected, significant differences were observed among the 10 analogues in both their cytotoxicity against P388 cells and their antibacterial activities against the five bacterial strains (Fig. 4). The IC_50_, HC_10_, and MIC values varied from 4.1 to >1000 nM, from 160 to >3000 nM, and from 1.0 to >34000 nM, respectively. We next examined the structures and distinct activity profiles of **A1**, **B**_**0**_**1**–**B**_**0**_**4**, **B**_**1**_**1**–**B**_**1**_**3**, **B**_**2**_**1**, and **B**_**2**_**2** individually.

**A1** had a lower level of hemolytic activity (240 nM for **A1** and 160 nM for **1**), but displayed higher P388 cytotoxicity (4.1 nM for **A1** and 5.8 nM for **1**) as well as higher growth inhibition against all five bacterial strains. Significantly, the MIC value of **A1** for *S. pneumoniae* was found to be 1.0 nM, which is eightfold lower than that of **1** (8.3 nM). Moreover, a fourfold decrease in the MIC values of **A1** was observed for *S. pyogenes* (8.0 for **A1** and 33 for **1**) and *L. monocytogenes* (1100 for **A1** and 4300 for **1**). As **A1** structurally differs from **1** only in the aliphatic amino acid at residue-4 (V for **A1** vs. L for **1**), this finding showed that the deletion of a single methylene unit at the C_β_-position of residue-4 in **1** markedly enhanced the antibacterial effect.

**B**_**0**_**1**–**B**_**0**_**4** are structurally similar to **1** and **A1**: residues-4, -6, -8, -10, -12, and -14 of these six sequences were either L or V. The subtle structural alteration with large functional differences among **1**, **A1**, and **B**_**0**_**1**–**B**_**0**_**4** indicated that the L and V residues differentially influenced the activity profiles despite their analogous aliphatic side chains. Consistent with the selection process for group B, **B**_**0**_**1**–**B**_**0**_**4** possessed three- to sevenfold decreased P388 cytotoxicity compared with **1** (17–41 nM for **B**_**0**_**1**–**B**_**0**_**4** vs. 5.8 nM for **1**). The weaker cytotoxicities of **B**_**0**_**1**–**B**_**0**_**4** coincided with the weaker hemolytic activities: the HC_10_ values of **B**_**0**_**1**–**B**_**0**_**4** were 1100–2000 nM. In contrast to these attenuated activities against the mammalian cells, **B**_**0**_**1**–**B**_**0**_**4** and **1** shared high antibacterial potencies against four strains (*S. pyogenes*, *E. faecalis*, *S. pneumoniae*, and *L. monocytogenes*), while *S. agalactiae* was more sensitive to the structural changes in **B**_**0**_**1**–**B**_**0**_**4** from **1**. Noteworthily, **B**_**0**_**1** displayed higher activities against *S. pyogenes* (16 nM) and *S. pneumoniae* (2.1 nM) than **1**, but sevenfold lower activity against red blood cells (1100 nM).

**B**_**1**_**1**–**B**_**1**_**3** contain one T residue in place of the aliphatic L and V residues and the presence of the one hydroxy group altered their biological behavior. The cytotoxicities and hemolytic activities of **B**_**1**_**1**–**B**_**1**_**3** were further decreased from those of **B**_**0**_**1**–**B**_**0**_**4**, corroborating the key role of the aliphatic amino acids for these activities. Moreover, the antibacterial spectra of these three peptides were quite different compared with **B**_**0**_**1**–**B**_**0**_**4**. While having enhanced antibacterial activities against *L. monocytogenes* (1100–2100 nM for **B**_**1**_**1**–**B**_**1**_**3** and 2100–4300 nM for **B**_**0**_**1**–**B**_**0**_**4**), they had decreased activity against *S. pyogenes* and *E. faecalis*. It is remarkable that **B**_**1**_**2** retained the sub-nanomolar antibacterial activities of **1** against *S. pyogenes* (67 nM) and *S. pneumoniae* (8.3 nM), although it had 70-fold weaker cytotoxicity (390 nM) and negligible hemolytic activity (>3000 nM).

**B**_**2**_**1** and **B**_**2**_**2** possess two hydrogen-bond-forming amino acids (T or N′). Despite retaining their potent ion channel functions, these two analogues were much weaker cytotoxic and antibacterial agents, indicating that T or N′ negatively affected these biological activities. This effect was more pronounced for **B**_**2**_**2**. **B**_**2**_**2** exhibited negligible activity against P388 cells (>1000 nM) and red blood cells (>3000 nM), and almost no antibacterial activity against all the bacterial strains tested except for *S. pneumoniae*. Structurally, the two T residues of **B**_**2**_**1**, and the T and N′ residues of **B**_**2**_**2** are six residues apart from each other. They can potentially serve to reinforce the helical folding of 6.3 residues per turn by the hydrogen bonding between the proximal T and T/N′^63^. Thus, the two hydrogen-bond-forming residues of the fixed distance would be critical factors for retaining the ion-conducting β^6.3^-helix in the liposomal membrane.

These detailed SAR studies of **1** and the 10 artificial peptides (**A1**, **B**_**0**_**1**–**B**_**0**_**4**, **B**_**1**_**1**–**B**_**1**_**3**, **B**_**2**_**1**, and **B**_**2**_**2**) clarified the crucial structural features for the ion channel, cytotoxic, hemolytic, and antibacterial activities. The aliphatic residues of **A1** and **B**_**0**_**1**–**B**_**0**_**4** are important for the potent cytotoxic and antibacterial activities, and the number and position of L and V affect the cytotoxic and hemolytic activities. While the presence of the one T residue of **B**_**1**_**1**–**B**_**1**_**3** among the L and V residues decreased the cytotoxic and hemolytic activities and changed the antibacterial spectra, the two hydrogen-bonding residues (T or N′) in **B**_**2**_**1**/**B**_**2**_**2** had detrimental effects on the cytotoxic, hemolytic, and antibacterial activities. These differences in the activity profiles of the 10 analogues likely result from different interactions between the side chains and lipid components. In the LUVs comprising POPC and POPG, all 10 analogues can fold into dimeric β^6.3^-helix structures, which transport H^+^ and Na^+^. The P388 cells, red blood cells, and five Gram-positive bacterial strains all differ from the LUVs and from each other in terms of the ratios and structures of their lipid components^57,64^. Although the precise factors remain to be investigated, we surmise that the formation of ion-conducting channels by **B**_**1**_**1**–**B**_**1**_**3** and **B**_**2**_**1**/**B**_**2**_**2** is disfavored by the presence of mammalian or bacterial strain-specific membrane molecules or environments. These data together indicate that specific modulation of the species- and strain-selectivity of the parent **1** is possible without changing its charge-neutral, hydrophobic, and ion-channel-forming properties. The pharmacological importance of the representative analogues **A1**, **B**_**0**_**1**, and **B**_**1**_**2** is also noteworthy. Because **A1** is superior to **1** in the both cytotoxic and antibacterial activities, and **B**_**0**_**1** and **B**_**1**_**2** possess potent antibiotic activities and much weaker hemolytic activities, **A1** can serve as a structural basis for designing new anticancer and topical antibacterial molecules, and **B**_**0**_**1** and **B**_**1**_**2** will be valuable starting points for the development of antibacterial agents for systemic use.

## Discussion

We developed a high-throughput method for the preparation and multidimensional functional evaluation of 4096 analogues of gramicidin A (**1**), which has potent ion transport, cytotoxic, hemolytic, and antibacterial activities. The analogues of this 15-mer peptidic natural product were strategically designed to maintain the physicochemical properties and ion channel function of **1**, and were randomized at residues-4, -6, -8, -10, -12, and -14 by aliphatic amino acids (L and V), and charge-neutral amino acids with hydroxy and amide groups (T and N′). Accordingly, the 4096-membered OBOC library based on **1** was constructed by integrating a split-and-mix solid-phase synthesis, structural analysis by MS/MS sequencing, and functional assessment by a set of three microscale assays. Peptides with H^+^/Na^+^ transport activity were categorized into a more cytotoxic group A (**1** and 40 compounds) and a less cytotoxic group B (276), the latter of which was further divided into subgroups B_0_ (31), B_1_ (208), and B_2_ (37). The large SAR data of thousands of analogues of **1** uncovered the positive effect of L and V on the cytotoxic and antibacterial activities (group A and subgroup B_0_), and the negative effect of T and N′ on both activities (subgroup B_2_). We selected 10 representative compounds, **A1**, **B**_**0**_**1**–**B**_**0**_**4**, **B**_**1**_**1**–**B**_**1**_**3**, **B**_**2**_**1**, and **B**_**2**_**2**, from the groups for scale-up syntheses and detailed functional analyses. While all 10 compounds retained the H^+^/Na^+^ transport activity in LUVs, the magnitudes of the activities against P388 cells, red blood cells, and the five Gram-positive bacteria varied remarkably. Despite their sequence similarity, these analogues are likely to induce ion transport differently in the liposomal, mammalian, and bacterial membranes. Distinct activity profiles were particularly evident for **A1**, **B**_**0**_**1**, **B**_**1**_**2**, and **B**_**2**_**2**. While **A1** and **1** were analogous in their profiles, **A1** had two- to eightfold more potent antibacterial activity against the five bacterial strains than **1**. **B**_**0**_**1** and **B**_**1**_**2** were at least seven times less hemolytic than **1**, yet had low MIC values. **B**_**2**_**2** was neither toxic to mammalian cells nor to bacterial cells despite its comparable ion transport activity. These four representative analogues were structurally similar to **1** and to each other, highlighting the remarkable sensitivity of the activities to subtle changes within the 15-mer sequence. The detailed biological evaluation of the 10 analogues also allowed us to discover potential lead structures for the development of new anticancer and topical antibacterial agents (**A1**) and new systemic antibacterial agents (**B**_**0**_**1** and **B**_**1**_**2**).

Finding natural-product-based molecules with the desirable functions is highly challenging and time-consuming via the preparation of small libraries or rational design of specific molecules. Thus, the discovery of 10 analogues with different activity profiles demonstrates the advantage of the present high-throughput strategy for preparing thousands of analogues and evaluating multiple biological activities. The overall methodology developed here will be widely applicable as a promising strategy for identifying key structural features and optimizing the pharmacologically favorable activity of natural products.

## Methods

### General remarks

Unless otherwise stated, all reactions sensitive to air or moisture were carried out under argon (Ar) atmosphere in dry solvents. Purification of CH_2_Cl_2_, DMF, and Et_2_O was performed on a Glass Contour solvent dispensing system (Nikko Hansen). All other reagents were used as supplied. SPPS was performed on a microwave-assisted peptide synthesizer MWS-1000 (EYELA) equipped with a sealed reaction vessel, in which the reaction temperature was monitored by an internal temperature probe, or an automated peptide synthesizer Initiator + Alstra (Biotage). High-performance liquid chromatography (HPLC) experiments were performed on an HPLC system equipped with a PU-2089 Plus intelligent pump (JASCO), a PU-2086 Plus intelligent pump (JASCO), a PU-4180 RHPLC pump (JASCO), or a 1100 HPLC system (Agilent). UHPLC experiments were performed with an X-LC system (JASCO) or an Extrema system (JASCO). UV absorbance was measured on a UV-1800 UV-VIS spectrophotometer (Shimadzu). Optical rotations were recorded on a P-2200 polarimeter (JASCO) at ambient temperature using the sodium D line. Infrared spectra were recorded on an FT/IR-4100 spectrometer (JASCO) as a thin film on CaF_2_. ^1^H and ^13^C NMR spectra were recorded on an ECX 500 (500 MHz for ^1^H NMR, 125 MHz for ^13^C NMR) spectrometer (JEOL). Chemical shifts are denoted in ppm on the *δ* scale relative to residual solvent peaks as an internal standard: CD_2_HOD (*δ* 3.31 for ^1^H NMR), DMSO-*d*_5_ (*δ* 2.50 for ^1^H NMR), DMSO-*d*_6_ (*δ* 39.5 for ^13^C NMR). HRMS spectra were recorded on a MicrOTOFII (Bruker Daltonics) electrospray ionization time of flight (TOF) mass spectrometer. Matrix-assisted laser desorption ionization-TOF MS and MS/MS sequencing analyses were performed on a TOF/TOF 5800 system (AB Sciex).

### Experimental data

For MS/MS spectra of compounds **1**, **A1**, **B**_**0**_**1**–**B**_**0**_**4**, **B**_**1**_**1**–**B**_**1**_**3**, **B**_**2**_**1**, and **B**_**2**_**2**, see Supplementary Figs. 8–18. For ^1^H, ^13^C NMR, ^1^H–^1^H DQF-COSY, ^1^H–^1^H TOCSY, ^1^H–^1^H NOESY, ^1^H–^13^C HMBC, and ^1^H–^13^C HMQC spectra of compounds **1**, **A1**, **B**_**1**_**3**, **B**_**2**_**1**, and **B**_**2**_**2** in DMSO-*d*_6_, see Supplementary Figs. 19–38. For chemical shifts of compounds **1**, **A1**, **B**_**1**_**3**, **B**_**2**_**1**, and **B**_**2**_**2** in DMSO-*d*_6_, see Supplementary Tables 11–13. For ^1^H NMR spectra of compounds **1**, **A1**, **B**_**0**_**1**–**B**_**0**_**4**, **B**_**1**_**1**–**B**_**1**_**3**, **B**_**2**_**1**, and **B**_**2**_**2** in CD_3_OD, see Supplementary Figs. 39–44. For HPLC charts showing purification of synthetic compounds, see Supplementary Figs. 45–63. For UHPLC charts of purified synthetic compounds, see Supplementary Figs. 64–74. For MS spectra of 593 bead-derived peptides, see Supplementary Figs. 75–173. For mean IC_50_ values with SDs in the mammalian cytotoxicity assay and mean EC_50_ values with SDs in the H^+^/Na^+^ transport assay, see Supplementary Table 14. For MIC values (μg/mL) against six bacterial strains, see Supplementary Table 15. For experimental procedures and spectroscopic data of compounds, see Supplementary Methods.

### Reporting summary

Further information on research design is available in the Nature Research Reporting Summary linked to this article.

## Supplementary information

Supplementary Information

Reporting Summary

## Data Availability

The data that support the findings of this study are available from the corresponding author upon reasonable request. Source data are provided with this paper.

## Source data

Source data

## References

[1] Koehn FE, Carter GT (2005). The evolving role of natural products in drug discovery. Nat. Rev. Drug Discov..

[2] Li JW-H, Vederas JC (2009). Drug discovery and natural products: end of an era or an endless frontier?. Science.

[3] Harvey AL, Edrada-Ebel R, Quinn RJ (2015). The re-emergence of natural products for drug discovery in the genomics era. Nat. Rev. Drug Discov..

[4] Newman DJ, Cragg GM (2016). Natural products as sources of new drugs from 1981 to 2014. J. Nat. Prod..

[5] Moloney MG (2016). Natural products as a source for novel antibiotics. Trends Pharmacol. Sci..

[6] Walsh, C. & Wencewicz, T. *Antibiotics: Challenges, Mechanisms, Opportunities* (ASM Press, Washington, DC, 2016).

[7] Szpilman AM, Carreira EM (2010). Probing the biology of natural products: molecular editing by diverted total synthesis. Angew. Chem. Int. Ed..

[8] Wright PM, Seiple IB, Myers AG (2014). The evolving role of chemical synthesis in antibacterial drug discovery. Angew. Chem. Int. Ed..

[9] Nandy JP (2009). Advances in solution- and solid-phase synthesis toward the generation of natural product-like libraries. Chem. Rev..

[10] Itoh H, Inoue M (2019). Comprehensive structure–activity relationship studies of macrocyclic natural products enabled by their total syntheses. Chem. Rev..

[11] Dubos RJ (1939). Studies on a bactericidal agent extracted from a soil *Bacillus*. J. Exp. Med.

[12] Hotchkiss RD, Dubos RJ (1940). Fractionation of the bactericidal agent from cultures of a soil *Bacillus*. J. Biol. Chem..

[13] Herrell WE, Heilman D (1941). Experimental and clinical studies on gramicidin. J. Clin. Invest..

[14] Robinson HJ, Molitor H (1942). Some toxicological and pharmacological properties of gramicidin, tyrocidine and tyrothricin. J. Pharmacol. Exp. Ther..

[15] Zerfas BL, Joo Y, Gao J (2016). Gramicidin A mutants with antibiotic activity against both Gram-positive and Gram-negative bacteria. ChemMedChem.

[16] Knopik-Skrocka A, Bielawski J (2002). The mechanism of the hemolytic activity of polyene antibiotics. Cell. Mol. Biol. Lett..

[17] David JM, Owens TA, Barwe SP, Rajasekaran AK (2013). Gramicidin A induces metabolic dysfunction and energy depletion leading to cell death in renal cell carcinoma cells. Mol. Cancer Ther..

[18] Sarges R, Witkop B, Gramicidin AV (1965). The structure of valine- and isoleucine-gramicidin A. J. Am. Chem. Soc..

[19] Fernandez-Lopez S (2001). Antibacterial agents based on the cyclic d,l-α-peptide architecture. Nature.

[20] Urry DW, Goodall MC, Glickson JD, Mayers DF (1971). The gramicidin A transmembrane channel: characteristics of head-to-head dimerized π_(L,D)_ helices. Proc. Natl Acad. Sci. USA.

[21] Arseniev AS, Barsukov IL, Bystrov VF, Lomize AL, Ovchinnikov YA (1985). ^1^H-NMR study of gramicidin A transmembrane ion channel. Head-to-head right-handed, single-stranded helices. FEBS Lett..

[22] Ketchem RR, Hu W, Cross TA (1993). High-resolution conformation of gramicidin A in a lipid bilayer by solid-state NMR. Science.

[23] Wallace BA (1998). Recent advances in the high resolution structures of bacterial channels: gramicidin A. J. Struct. Biol..

[24] Kovacs F, Quine J, Cross TA (1999). Validation of the single-stranded channel conformation of gramicidin A by solid-state NMR. Proc. Natl Acad. Sci. USA.

[25] Kelkar DA, Chattopadhyay A (2007). The gramicidin ion channel: a model membrane protein. Biochim. Biophys. Acta.

[26] Myers VB, Haydon DA (1972). Ion transfer across lipid membranes in the presence of gramicidin A. II. The ion selectivity. Biochim. Biophys. Acta.

[27] Stankovic CJ, Heinemann SH, Delfino JM, Sigworth FJ, Schreiber SL (1989). Transmembrane channels based on tartaric acid-gramicidin A hybrids. Science.

[28] Koeppe RE, Andersen OS (1996). Engineering the gramicidin channel. Annu. Rev. Biophys. Biomol. Struct..

[29] Reiß P, Koert U (2013). Ion-channels: goals for function-oriented synthesis. Acc. Chem. Res..

[30] Mayer M, Yang J (2013). Engineered ion channels as emerging tools for chemical biology. Acc. Chem. Res..

[31] Mao J, Kuranaga T, Hamamoto H, Sekimizu K, Inoue M (2015). Rational design, synthesis, and biological evaluation of lactam-bridged gramicidin A analogues: discovery of a low-hemolytic antibacterial peptide. ChemMedChem.

[32] Mao J, Itoh H, Sakurai K, Inoue M (2020). Phospholipid-dependent functions of a macrocyclic analogue of the ion-channel-forming antibiotic gramicidin A. Chem. Pharm. Bull..

[33] Sorochkina AI (2012). N-Terminally glutamate-substituted analogue of gramicidin A as protonophore and selective mitochondrial uncoupler. PLoS ONE.

[34] Wang F (2012). Solubilized gramicidin A as potential systemic antibiotics. ChemBioChem.

[35] Rossiter SE, Fletcher MH, Wuest WM (2017). Natural products as platforms to overcome antibiotic resistance. Chem. Rev..

[36] Henninot A, Collins JC, Nuss JM (2018). The current state of peptide drug discovery: back to the future?. J. Med. Chem..

[37] Pavithrra G, Rajasekaran R (2020). Gramicidin peptide to combat antibiotic resistance: a review. Int. J. Pept. Res. Ther..

[38] Lam KS, Lebl M, Krchňák V (1997). The “one-bead-one-compound” combinatorial library method. Chem. Rev..

[39] Lam KS, Liu R, Miyamoto S, Lehman AL, Tuscano JM (2003). Applications of one-bead-one-compound combinatorial libraries and chemical microarrays in signal transduction research. Acc. Chem. Res..

[40] Doran TM (2019). Synthesis and screening of bead-displayed combinatorial libraries. Methods Enzymol..

[41] Itoh H (2019). Development of a high-throughput strategy for discovery of potent analogues of antibiotic lysocin E. Nat. Commun..

[42] Russell EWB, Weiss LB, Navetta FI, Koeppe RE, Andersen OS (1986). Single-channel studies on linear gramicidins with altered amino acid side chains. Effects of altering the polarity of the side chain at position 1 in gramicidin A. Biophys. J..

[43] Mattice GL, Koeppe RE, Providence LL, Andersen OS (1995). Stabilizing effect of d-alanine^2^ in gramicidin channels. Biochemistry.

[44] Fonseca V (1992). Gramicidin channels that have no tryptophan residues. Biochemistry.

[45] Jordan JB, Shobana S, Andersen OS, Hinton JF (2006). Effects of glycine substitutions on the structure and function of gramicidin A channels. Biochemistry.

[46] Hamada T, Matsunaga S, Yano G, Fusetani N (2005). Polytheonamides A and B, highly cytotoxic, linear polypeptides with unprecedented structural features, from the marine sponge, *Theonella swinhoei*. J. Am. Chem. Soc..

[47] Hayata A, Itoh H, Inoue M (2018). Solid-phase total synthesis and dual mechanism of action of the channel-forming 48-mer peptide polytheonamide B. J. Am. Chem. Soc..

[48] Weygand F, Steglich W, Bjarnason J (1968). Easily cleavable protective groups for acid amide groups. III. Derivatives of aspargine and glutamine with 2,4-dimethoxybenzyl- and 2,4,6-trimethoxybenzyl-protected amide groups. Chem. Ber..

[49] Bayer E (1991). Towards the chemical synthesis of proteins. Angew. Chem. Int. Ed., Engl..

[50] Meldal M (1997). Properties of solid supports. Methods Enzymol..

[51] Lawrenson S, North M, Peigneguy F, Routledge A (2017). Greener solvents for solid-phase synthesis. Green. Chem..

[52] Atherton E, Logan CJ, Sheppard RC (1981). Peptide synthesis. Part 2. Procedures for solid-phase synthesis using *N*^α^-fluorenylmethoxycarbonylamino-acids on polyamide supports. Synthesis of substance P and of acyl carrier protein 65–74 decapeptide. J. Chem. Soc. Perkin Trans..

[53] Yu H-M, Chen S-T, Wang K-T (1992). Enhanced coupling efficiency in solid-phase peptide synthesis by microwave irradiation. J. Org. Chem..

[54] Bacsa B, Hováti K, Bõsze S, Andreae F, Kappe CO (2008). Solid-phase synthesis of difficult peptide sequences at elevated temperatures: a critical comparison of microwave and conventional heating technologies. J. Org. Chem..

[55] Pedersen SL, Tofteng P, Malik L, Jensen KJ (2012). Microwave heating in solid-phase peptide synthesis. Chem. Soc. Rev..

[56] Carpino LA (1993). 1-Hydroxy-7-azabenzotriazole. An efficient peptide coupling additive. J. Am. Chem. Soc..

[57] Epand RM, Epand RF (2009). Domains in bacterial membranes and the action of antimicrobial agents. Mol. BioSyst..

[58] Clement NR, Gould JM (1981). Pyranine (8-hydroxy-1,3,6-pyrenetrisulfonate) as a probe of internal aqueous hydrogen ion concentration in phospholipid vesicles. Biochemistry.

[59] Ishiyama M, Shiga M, Sasamoto K, Mizoguchi M, He P (1993). A new sulfonated tetrazolium salt that produces a highly water-soluble formazan dye. Chem. Pharm. Bull..

[60] Maillard N, Clouet A, Darbre T, Reymond J-L (2009). Combinatorial libraries of peptide dendrimers: design, synthesis, on-bead high-throughput screening, bead decoding and characterization. Nat. Protoc..

[61] Pearson DA, Blanchette M, Baker ML, Guindon CA (1989). Trialkylsilanes as scavengers for the trifluoroacetic acid deblocking of protecting groups in peptide synthesis. Tetrahedron Lett..

[62] García-Martín F (2006). ChemMatrix, a poly(ethylene glycol)-based support for the solid-phase synthesis of complex peptides. J. Comb. Chem..

[63] Hamada T (2010). Solution structure of polytheonamide B, a highly cytotoxic nonribosomal polypeptide from marine sponge. J. Am. Chem. Soc..

[64] van Meer G, Voelker DR, Feigenson GW (2008). Membrane lipids: where they are and how they behave. Nat. Rev. Mol. Cell. Biol..

