# Supplementary material for: Discovery of gramicidin A analogues with altered activities by multidimensional screening of a one-bead-one-compound library
Source: Nat Commun. 2020 Oct 1;11:4935. doi: 10.1038/s41467-020-18711-2 (PMC7531004; doi:10.1038/s41467-020-18711-2)
Supplement: Supplementary file 1 — Supplementary Information [file 41467_2020_18711_MOESM1_ESM.pdf]

Supplementary Information

Discovery of Gramicidin A Analogues with Altered Activities by Multidimensional Screening of a One-Bead-One-Compound Library

Yuri Takada[1], Hiroaki Itoh[1], Atmika Paudel[2], Suresh Panthee[2], Hiroshi Hamamoto[2], Kazuhisa Sekimizu[2], and Masayuki Inoue*[1]

[1]Graduate School of Pharmaceutical Sciences, The University of Tokyo, 7-3-1 Hongo, Bunkyo-ku, Tokyo 113-0033, Japan.

[2]Teikyo University Institute of Medical Mycology, 359 Otsuka, Hachioji, Tokyo 192-0395, Japan.

*E-mail: inoue@mol.f.u-tokyo.ac.jp

Table of Contents

**Supplementary Figures**	3
**Supplementary Tables**	5
**Supplementary Methods**	19
**Supplementary References**	181

Supplementary Figures

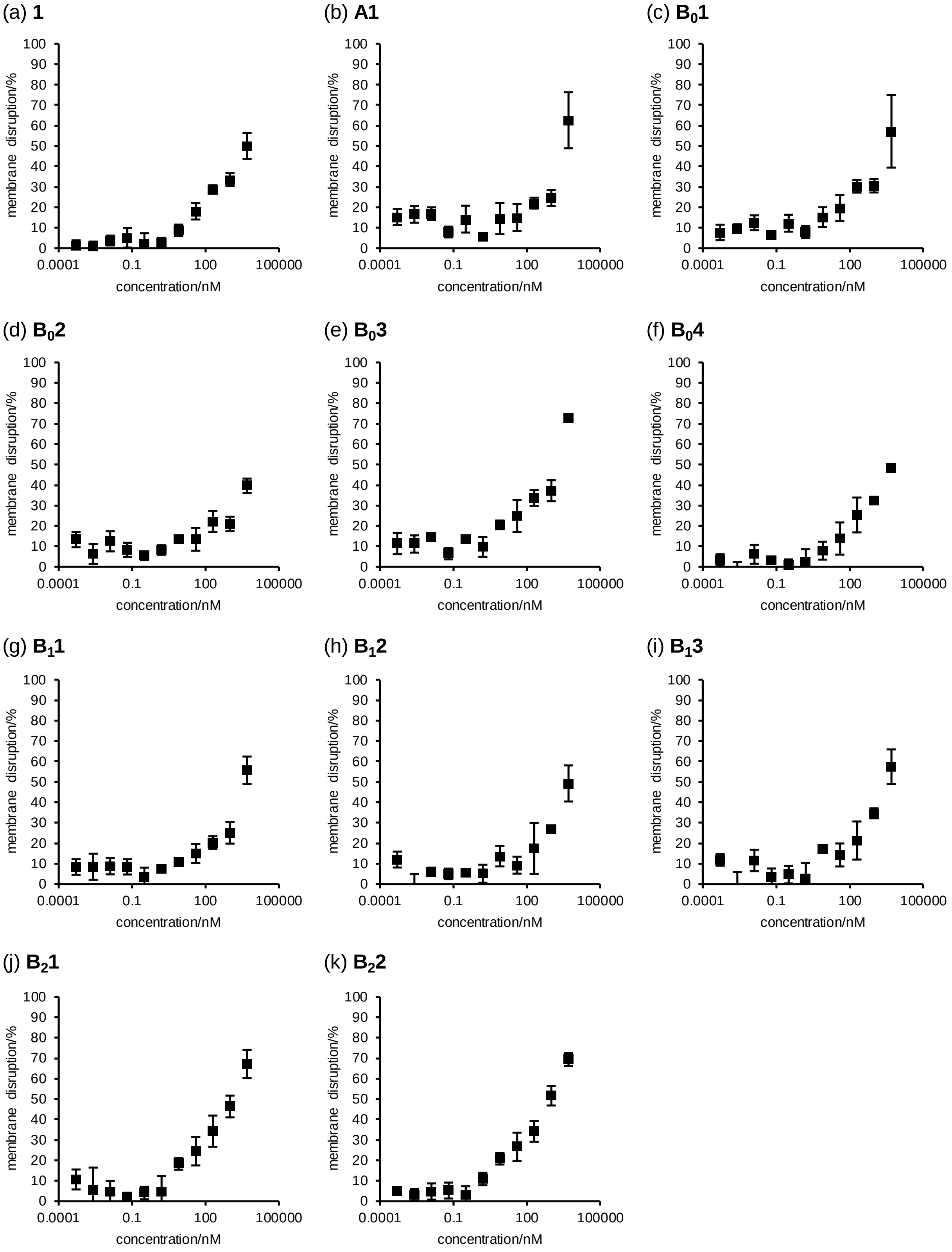

Supplementary Figure 1. Concentration-response curves for membrane disruption assay. Representative plots of **1**, **A1**, **$B_0$1**–**$B_0$4**, **$B_1$1**–**$B_1$3**, **$B_2$1**, and **$B_2$2** towards liposomes (EYPC/EYPG = 19/1) from three independent experiments were shown as mean ± SD. Source data are provided as a Source Data file.

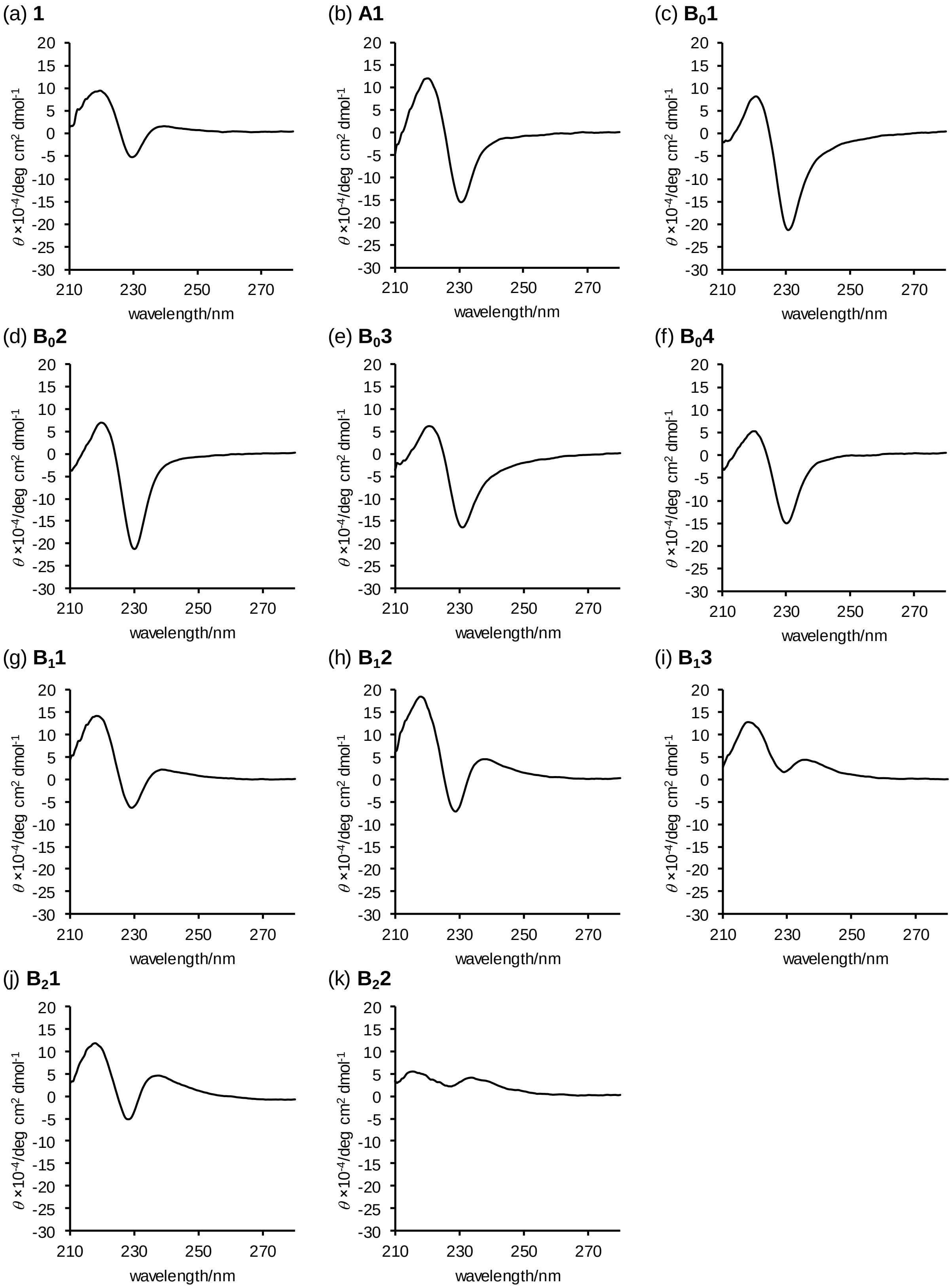

Supplementary Figure 2. CD spectra of peptides. Spectra of **1**, **A1**, $\mathbf{B_0 1}$–$\mathbf{B_0 4}$, $\mathbf{B_1 1}$–$\mathbf{B_1 3}$, $\mathbf{B_2 1}$, and $\mathbf{B_2 2}$ in liposomes (POPC/POPG = 19/1) were shown.

Supplementary Tables

Supplementary Table 1. One-bead-derived peptides of groups A and B in plates 0−14[a]

bead number 1-13584	plate-well	activity			group	residue number					
		cell viability/%	H^+/Na^+ transport activity	antibacterial activity		4	6	8	10	12	14
1	0-1A	100.0	1.07	-	B	Leu	Val	Thr	Val	Val	Val
65	1-5A	100.0	1.08	+	B	Leu	Leu	Val	Leu	Thr	Val
79	1-6G	100.0	1.14	++	B	Val	Val	Leu	Leu	Thr	Val
87	1-7G	100.0	1.12	++	B	Leu	Val	Val	Leu	Thr	Val
94	1-8F	100.0	1.16	-	B	Thr	Val	Leu	Leu	Leu	Val
97	1-9A	59.7	1.24	+	B	Val	Val	Leu	Val	Val	Leu
100	1-9D	9.5	1.18	++	A	Val	Val	Leu	Leu	Val	Val
102	1-9F	100.0	1.09	-	B	Thr	Leu	Leu	Val	Leu	Val
113	1-11A	100.0	1.00	-	B	Leu	Thr	Leu	Leu	Val	Thr
129	2-2A	100.0	1.05	+	B	Val	Leu	Leu	Leu	Leu	Thr
131	2-2C	100.0	1.10	-	B	Thr	Leu	Leu	Thr	Leu	Leu
169	2-7A	100.0	1.04	++	B	Val	Val	Val	Leu	Val	Val
173	2-7E	100.0	1.07	-	B	Leu	Leu	Asm	Leu	Val	Leu
182	2-8F	100.0	1.16	-	B	Thr	Val	Leu	Val	Leu	Val
196	2-10D	100.0	1.06	-	B	Leu	Leu	Leu	Thr	Val	Leu
200	2-10H	100.0	1.04	+	B	Leu	Val	Thr	Leu	Leu	Val
201	2-11A	100.0	1.06	-	B	Leu	Val	Leu	Val	Thr	Leu
231	3-3G	100.0	1.14	-	B	Val	Val	Asm	Leu	Leu	Val
285	3-10E	100.0	1.08	-	B	Leu	Val	Thr	Val	Val	Leu
300	4-1D	100.0	1.00	-	B	Leu	Leu	Leu	Leu	Leu	Asm
346	4-7B	100.0	1.12	-	B	Leu	Asm	Val	Leu	Leu	Val
362	4-9B	100.0	1.05	-	B	Leu	Val	Val	Val	Val	Asm
367	4-9G	100.0	1.24	-	B	Thr	Leu	Leu	Thr	Leu	Val
379	4-11C	100.0	1.19	-	B	Val	Thr	Val	Val	Val	Leu
381	4-11E	100.0	1.03	-	B	Leu	Thr	Leu	Leu	Thr	Val
437	5-7E	100.0	1.11	+	B	Val	Leu	Val	Val	Val	Leu
448	5-8H	34.1	1.07	+	B	Val	Val	Leu	Val	Val	Leu
457	5-10A	95.8	1.08	+	B	Val	Val	Leu	Leu	Thr	Leu
511	6-5G	1.8	1.01	++	A	Val	Val	Leu	Leu	Leu	Val
521	6-7A	100.0	1.05	+	B	Val	Val	Val	Leu	Thr	Leu
527	6-7G	5.9	1.16	++	A	Val	Leu	Leu	Leu	Leu	Leu
532	6-8D	100.0	1.03	-	B	Val	Val	Val	Thr	Val	Val
569	7-2A	100.0	1.01	-	B	Thr	Leu	Leu	Leu	Val	Val
603	7-6C	100.0	1.10	-	B	Val	Leu	Asm	Val	Leu	Leu
611	7-7C	100.0	1.05	-	B	Leu	Thr	Leu	Val	Leu	Leu
629	7-9E	100.0	1.12	-	B	Thr	Val	Val	Leu	Val	Val
638	7-10F	100.0	1.01	-	B	Leu	Leu	Asm	Leu	Leu	Thr
646	7-11F	0.2	1.11	+	A	Leu	Leu	Leu	Leu	Val	Leu
660	8-2D	15.6	1.06	++	A	Val	Leu	Val	Leu	Val	Leu
662	8-2F	1.5	1.04	+++	A	Val	Val	Val	Leu	Leu	Leu
676	8-4D	100.0	1.02	++	B	Val	Val	Val	Val	Leu	Val
725	8-10E	100.0	1.10	+	B	Leu	Val	Val	Val	Val	Val
762	9-4B	96.8	1.01	-	B	Leu	Val	Val	Val	Val	Thr
770	9-5B	100.0	1.11	-	B	Leu	Leu	Leu	Val	Leu	Thr
789	9-7E	100.0	1.08	-	B	Thr	Leu	Leu	Leu	Val	Leu
809	9-10A	100.0	1.13	-	B	Leu	Val	Thr	Val	Val	Leu
885	10-8E	93.4	1.03	-	B	Val	Val	Leu	Val	Val	Val
903	10-10G	89.7	1.15	-	B	Val	Val	Asm	Val	Leu	Leu
905	10-11A	90.6	1.19	+	B	Leu	Leu	Val	Val	Val	Leu
906	10-11B	100.0	1.06	+	B	Val	Leu	Leu	Val	Leu	Val
1004	12-1D	60.4	1.04	+	B	Val	Val	Val	Val	Val	Leu
1119	13-4G	100.0	1.10	-	B	Val	Val	Val	Val	Leu	Leu
1152	13-8H	100.0	1.05	-	B	Val	Leu	Leu	Asm	Val	Val
1156	13-9D	85.9	1.03	-	B	Thr	Val	Leu	Thr	Val	Val
1176	13-11H	100.0	1.01	-	B	Leu	Leu	Thr	Leu	Thr	Leu
1242	14-9B	65.8	1.13	-	B	Val	Leu	Asm	Leu	Leu	Leu
1251	14-10C	52.9	1.00	+	B	Val	Val	Leu	Leu	Thr	Leu
1253	14-10E	58.1	1.03	-	B	Leu	Val	Leu	Thr	Leu	Val

[a]The structures of the residues-4, -6, -8, -10, -12, and -14 are displayed as three-letter codes of the amino acids. H^+/Na^+ transport activity normalized against the value of **1** (1.00) and antibacterial activity are also listed. Antibacterial activity against *S. pyogenes* was evaluated using the three concentrations. +++: inhibition by the 640-fold diluted peptide solution, ++: inhibition by the 160-fold diluted solution, +: inhibition by the 40-fold diluted solution, -: no inhibition.

Supplementary Table 2. One-bead-derived peptides of groups A and B in plates 15−31[a]

bead number 1-13584	plate-well	activity			group	residue number					
		cell viability/%	H^+/Na^+ transport activity	antibacterial activity		4	6	8	10	12	14
1284	15-3D	71.2	1.06	-	B	Thr	Leu	Leu	Thr	Leu	Val
1289	15-4A	95.6	1.03	+	B	Leu	Val	Val	Val	Leu	Val
1343	15-10G	4.0	1.02	-	A	Val	Leu	Leu	Leu	Leu	Leu
1400	16-6H	72.7	1.10	-	B	Leu	Leu	Asm	Leu	Leu	Leu
1406	16-7F	91.1	1.04	+	B	Val	Leu	Val	Val	Leu	Leu
1479	17-5G	100.0	1.19	-	B	Leu	Leu	Asm	Leu	Val	Leu
1489	17-7A	100.0	1.03	-	B	Val	Asm	Leu	Leu	Val	Leu
1509	17-9E	100.0	1.10	-	B	Thr	Val	Leu	Leu	Val	Leu
1516	17-10D	100.0	1.06	-	B	Thr	Leu	Leu	Leu	Val	Leu
1520	17-10H	100.0	1.04	-	B	Leu	Val	Thr	Val	Val	Val
1522	17-11B	100.0	1.03	-	B	Leu	Val	Leu	Val	Leu	Thr
1523	17-11C	100.0	1.04	+	B	Val	Val	Thr	Leu	Leu	Val
1592	18-8H	100.0	1.03	-	B	Leu	Thr	Val	Thr	Leu	Leu
1631	19-2G	100.0	1.04	-	B	Leu	Leu	Thr	Val	Leu	Leu
1711	20-1G	100.0	1.01	-	B	Leu	Val	Thr	Leu	Val	Leu
1751	20-6G	0.0	1.05	-	A	Leu	Leu	Leu	Val	Leu	Val
1796	21-1D	0.0	1.09	-	A	Leu	Leu	Val	Leu	Leu	Val
1819	21-4C	100.0	1.22	+	B	Leu	Val	Thr	Leu	Leu	Leu
1842	21-7B	100.0	1.26	-	B	Thr	Leu	Leu	Val	Val	Val
1844	21-7D	47.9	1.18	++	B	Val	Val	Val	Leu	Val	Leu
1849	21-8A	100.0	1.07	+	B	Val	Val	Val	Leu	Thr	Leu
1873	21-11A	100.0	1.09	-	B	Val	Val	Leu	Val	Val	Val
1888	22-1H	100.0	1.16	-	B	Leu	Leu	Leu	Leu	Thr	Leu
1898	22-3B	100.0	1.07	-	B	Leu	Thr	Leu	Leu	Val	Val
1975	23-1G	100.0	1.04	+	B	Leu	Val	Leu	Leu	Leu	Thr
1983	23-2G	100.0	1.00	-	B	Val	Val	Val	Val	Val	Thr
2020	23-7D	100.0	1.02	-	B	Leu	Val	Val	Leu	Leu	Asm
2066	24-2B	100.0	1.07	-	B	Val	Val	Val	Thr	Leu	Val
2082	24-4B	100.0	1.16	+	B	Leu	Val	Val	Val	Leu	Leu
2085	24-4E	100.0	1.20	-	B	Val	Thr	Leu	Val	Leu	Leu
2117	24-8E	100.0	1.15	+	B	Val	Leu	Leu	Leu	Thr	Val
2118	24-8F	100.0	1.26	-	B	Leu	Val	Asm	Val	Leu	Leu
2120	24-8H	17.0	1.24	++	A	Val	Val	Leu	Val	Leu	Val
2139	24-11C	100.0	1.05	-	B	Thr	Leu	Leu	Thr	Val	Val
2154	25-2B	100.0	1.09	-	B	Leu	Val	Thr	Val	Leu	Val
2158	25-2F	100.0	1.18	-	B	Leu	Leu	Thr	Val	Leu	Leu
2172	25-4D	100.0	1.22	+	B	Leu	Val	Thr	Leu	Leu	Leu
2182	25-5F	100.0	1.28	++	B	Val	Val	Val	Val	Val	Leu
2195	25-7C	100.0	1.34	-	B	Leu	Val	Asm	Leu	Val	Val
2205	25-8E	0.0	1.01	-	A	Leu	Leu	Leu	Leu	Leu	Val
2207	25-8G	100.0	1.18	-	B	Leu	Val	Val	Thr	Val	Leu
2212	25-9D	11.5	1.18	+	A	Val	Leu	Leu	Val	Val	Val
2218	25-10B	100.0	1.32	-	B	Val	Val	Asm	Leu	Leu	Leu
2235	26-1C	100.0	1.01	+	B	Leu	Val	Thr	Leu	Leu	Val
2236	26-1D	100.0	1.03	+	B	Leu	Val	Leu	Thr	Val	Leu
2242	26-2B	100.0	1.15	-	B	Thr	Leu	Leu	Val	Val	Leu
2283	26-7C	100.0	1.07	-	B	Leu	Leu	Leu	Leu	Thr	Leu
2290	26-8B	100.0	1.14	-	B	Leu	Val	Thr	Val	Leu	Leu
2346	27-4B	100.0	1.03	+	B	Leu	Leu	Leu	Leu	Thr	Val
2425	28-3A	100.0	1.07	-	B	Val	Leu	Val	Leu	Val	Asm
2432	28-3H	100.0	1.06	-	B	Leu	Leu	Leu	Asm	Val	Leu
2444	28-5D	100.0	1.32	-	B	Leu	Leu	Asm	Leu	Leu	Leu
2475	28-9C	100.0	1.13	-	B	Leu	Thr	Val	Val	Val	Leu
2495	28-11G	100.0	1.22	-	B	Val	Thr	Leu	Leu	Leu	Val
2526	29-4F	100.0	1.02	-	B	Val	Val	Leu	Val	Leu	Asm
2532	29-5D	100.0	1.22	-	B	Thr	Val	Leu	Val	Val	Leu
2554	29-8B	9.2	1.06	++	A	Val	Val	Leu	Val	Leu	Val
2557	29-8E	31.0	1.21	++	B	Leu	Leu	Val	Leu	Val	Val
2580	29-11D	100.0	1.00	-	B	Val	Val	Leu	Val	Thr	Leu
2682	31-2B	100.0	1.06	+	B	Val	Leu	Leu	Leu	Thr	Leu
2718	31-6F	0.3	1.11	+	A	Leu	Leu	Leu	Leu	Val	Val
2727	31-7G	100.0	1.05	-	B	Val	Leu	Val	Val	Thr	Leu

[a]The structures of the residues-4, -6, -8, -10, -12, and -14 are displayed as three-letter codes of the amino acids. H^+/Na^+ transport activity normalized against the value of **1** (1.00) and antibacterial activity are also listed. Antibacterial activity against *S. pyogenes* was evaluated using the three concentrations. +++: inhibition by the 640-fold diluted peptide solution, ++: inhibition by the 160-fold diluted solution, +: inhibition by the 40-fold diluted solution, -: no inhibition.

Supplementary Table 3. One-bead-derived peptides of groups A and B in plates 32−44[a]

bead number 1-13584	plate-well	activity			group	residue number					
		cell viability/%	H^+/Na^+ transport activity	antibacterial activity		4	6	8	10	12	14
2764	32-1D	75.7	1.12	-	B	Leu	Leu	Leu	Leu	Val	Asm
2793	32-5A	84.6	1.08	-	B	Leu	Leu	Val	Val	Val	Thr
2828	32-9D	2.8	1.15	-	A	Leu	Leu	Leu	Val	Leu	Leu
2829	32-9E	100.0	1.13	-	B	Val	Val	Thr	Leu	Val	Leu
2832	32-9H	17.7	1.23	+	A	Val	Leu	Leu	Val	Val	Val
2881	33-5A	100.0	1.16	-	B	Leu	Val	Asm	Leu	Val	Leu
2887	33-5G	99.0	1.01	-	B	Leu	Leu	Val	Val	Thr	Val
2895	33-6G	95.1	1.06	-	B	Val	Leu	Leu	Val	Leu	Thr
2898	33-7B	100.0	1.13	-	B	Val	Val	Asm	Val	Leu	Leu
2904	33-7H	12.5	1.09	-	A	Leu	Leu	Leu	Leu	Leu	Leu
2911	33-8G	100.0	1.16	-	B	Val	Leu	Val	Val	Val	Asm
2931	33-11C	100.0	1.06	+	B	Leu	Leu	Val	Thr	Val	Leu
2932	33-11D	87.5	1.27	-	B	Leu	Val	Asm	Leu	Val	Val
2951	34-2G	93.1	1.10	-	B	Leu	Leu	Asm	Val	Val	Leu
2953	34-3A	100.0	1.20	-	B	Leu	Leu	Asm	Leu	Val	Leu
2967	34-4G	100.0	1.04	-	B	Leu	Leu	Asm	Leu	Leu	Thr
2978	34-6B	100.0	1.18	-	B	Leu	Leu	Asm	Val	Leu	Leu
2994	34-8B	6.3	1.14	++	A	Val	Val	Val	Leu	Val	Leu
3019	34-11C	100.0	1.12	-	B	Thr	Val	Leu	Val	Val	Val
3111	35-11G	8.2	1.12	-	A	Val	Val	Leu	Val	Val	Leu
3124	36-2D	0.0	1.26	++	A	Leu	Val	Val	Val	Leu	Leu
3130	36-3B	100.0	1.05	-	B	Thr	Leu	Leu	Val	Leu	Thr
3143	36-4G	99.9	1.04	+	B	Leu	Leu	Leu	Leu	Thr	Val
3144	36-4H	100.0	1.01	-	B	Val	Leu	Leu	Asm	Val	Val
3161	36-7A	100.0	1.00	-	B	Val	Val	Leu	Thr	Leu	Val
3205	37-1E	90.2	1.16	-	B	Val	Leu	Leu	Val	Thr	Leu
3206	37-1F	97.4	1.19	-	B	Val	Leu	Thr	Val	Leu	Val
3226	37-4B	87.6	1.06	-	B	Thr	Val	Val	Thr	Leu	Leu
3244	37-6D	100.0	1.03	-	B	Leu	Leu	Leu	Thr	Thr	Leu
3247	37-6G	99.5	1.24	-	B	Leu	Thr	Leu	Leu	Val	Leu
3255	37-7G	4.6	1.13	+	A	Val	Leu	Leu	Leu	Val	Leu
3260	37-8D	100.0	1.10	+	B	Val	Leu	Leu	Thr	Leu	Val
3280	37-10H	100.0	1.20	-	B	Leu	Leu	Asm	Val	Leu	Thr
3308	38-3D	0.1	1.15	++	A	Val	Val	Leu	Leu	Leu	Leu
3311	38-3G	3.3	1.14	++	A	Val	Val	Val	Leu	Leu	Leu
3328	38-5H	100.0	1.08	-	B	Val	Leu	Thr	Leu	Val	Thr
3329	38-6A	100.0	1.33	+	B	Leu	Leu	Val	Asm	Leu	Val
3364	38-10D	100.0	1.09	-	B	Leu	Thr	Val	Val	Leu	Val
3365	38-10E	100.0	1.03	+	B	Val	Leu	Leu	Leu	Leu	Thr
3369	38-11A	100.0	1.00	-	B	Val	Val	Leu	Val	Asm	Val
3375	38-11G	100.0	1.29	-	B	Val	Leu	Thr	Leu	Val	Val
3437	39-8E	100.0	1.01	-	B	Thr	Leu	Leu	Thr	Leu	Val
3492	40-4D	100.0	1.12	-	B	Val	Leu	Thr	Val	Val	Val
3499	40-5C	100.0	1.01	+	B	Leu	Val	Val	Leu	Val	Asm
3548	40-11D	100.0	1.16	-	B	Thr	Val	Val	Leu	Val	Leu
3584	41-4H	100.0	1.22	-	B	Leu	Val	Asm	Val	Leu	Leu
3596	41-6D	100.0	1.06	-	B	Leu	Val	Asm	Val	Val	Leu
3631	41-10G	100.0	1.02	-	B	Thr	Val	Leu	Val	Val	Val
3633	41-11A	1.7	1.04	-	A	Leu	Val	Leu	Val	Leu	Val
3733	43-1E	100.0	1.11	-	B	Leu	Thr	Leu	Val	Val	Val
3773	43-6E	100.0	1.03	-	B	Leu	Leu	Leu	Leu	Asm	Thr
3778	43-7B	100.0	1.08	-	B	Thr	Leu	Leu	Leu	Leu	Val
3780	43-7D	100.0	1.16	-	B	Leu	Leu	Thr	Leu	Thr	Leu
3782	43-7F	100.0	1.12	-	B	Leu	Leu	Thr	Thr	Val	Leu
3786	43-8B	100.0	1.18	-	B	Thr	Val	Val	Leu	Leu	Leu
3800	43-9H	100.0	1.23	-	B	Val	Leu	Asm	Leu	Leu	Val
3828	44-2D	95.2	1.02	+	B	Val	Leu	Leu	Leu	Val	Thr
3847	44-4G	0.0	1.22	+	A	Leu	Val	Leu	Leu	Val	Leu
3854	44-5F	100.0	1.18	-	B	Leu	Leu	Leu	Val	Leu	Asm
3858	44-6B	100.0	1.28	-	B	Leu	Leu	Thr	Thr	Leu	Leu
3861	44-6E	0.1	1.51	++	A	Leu	Val	Val	Leu	Val	Leu
3866	44-7B	100.0	1.31	-	B	Val	Leu	Leu	Val	Val	Asm

[a]The structures of the residues-4, -6, -8, -10, -12, and -14 are displayed as three-letter codes of the amino acids. H^+/Na^+ transport activity normalized against the value of **1** (1.00) and antibacterial activity are also listed. Antibacterial activity against *S. pyogenes* was evaluated using the three concentrations. +++: inhibition by the 640-fold diluted peptide solution, ++: inhibition by the 160-fold diluted solution, +: inhibition by the 40-fold diluted solution, -: no inhibition.

Supplementary Table 4. One-bead-derived peptides of groups A and B in plates 44−59[a]

bead number 1-13584	plate-well	activity			group	residue number					
		cell viability/%	H^+/Na^+ transport activity	antibacterial activity		4	6	8	10	12	14
3885	44-9E	100.0	1.00	-	B	Leu	Leu	Leu	Val	Asm	Thr
3903	44-11G	100.0	1.10	-	B	Val	Val	Leu	Leu	Val	Asm
3911	45-1G	100.0	1.00	-	B	Leu	Leu	Leu	Leu	Val	Asm
3929	45-4A	100.0	1.14	-	B	Leu	Leu	Asm	Leu	Leu	Thr
3931	45-4C	100.0	1.16	-	B	Leu	Leu	Asm	Val	Val	Val
3956	45-7D	100.0	1.12	+	B	Val	Leu	Thr	Leu	Leu	Leu
4031	46-5G	92.4	1.05	-	B	Leu	Leu	Val	Thr	Val	Leu
4049	46-8A	100.0	1.15	-	B	Val	Val	Asm	Val	Leu	Leu
4126	47-6F	100.0	1.06	-	B	Val	Leu	Thr	Leu	Val	Val
4128	47-6H	100.0	1.03	+	B	Leu	Val	Leu	Leu	Thr	Leu
4143	47-8G	100.0	1.22	-	B	Leu	Val	Asm	Leu	Leu	Leu
4186	48-3B	98.7	1.44	-	B	Leu	Leu	Asm	Leu	Leu	Val
4187	48-3C	95.1	1.32	-	B	Thr	Val	Leu	Val	Leu	Val
4195	48-4C	90.6	1.19	-	B	Val	Leu	Thr	Leu	Thr	Leu
4203	48-5C	100.0	1.16	-	B	Val	Val	Leu	Leu	Asm	Leu
4208	48-5H	100.0	1.05	-	B	Leu	Thr	Leu	Val	Leu	Leu
4209	48-6A	96.2	1.33	-	B	Val	Leu	Leu	Leu	Thr	Leu
4223	48-7G	95.6	1.09	+	B	Val	Val	Val	Val	Val	Val
4232	48-8H	93.0	1.05	-	B	Val	Leu	Leu	Thr	Leu	Val
4236	48-9D	93.0	1.43	-	B	Thr	Val	Leu	Leu	Leu	Val
4258	49-1B	85.9	1.00	-	B	Val	Leu	Val	Val	Thr	Val
4279	49-3G	98.8	1.05	-	B	Leu	Val	Thr	Leu	Val	Leu
4297	49-6A	2.3	1.29	+	A	Leu	Leu	Val	Val	Leu	Val
4313	49-8A	3.2	1.21	+	A	Leu	Leu	Leu	Leu	Val	Val
4322	49-9B	100.0	1.05	-	B	Val	Leu	Leu	Leu	Thr	Thr
4328	49-9H	100.0	1.13	+	B	Leu	Val	Leu	Leu	Thr	Thr
4329	49-10A	14.3	1.18	-	A	Val	Val	Leu	Val	Leu	Leu
4357	50-2E	100.0	1.01	-	B	Leu	Val	Thr	Val	Leu	Val
4395	50-7C	100.0	1.07	-	B	Val	Leu	Thr	Val	Val	Leu
4421	50-10E	100.0	1.12	-	B	Leu	Leu	Thr	Thr	Leu	Leu
4427	50-11C	100.0	1.18	-	B	Leu	Val	Thr	Val	Leu	Val
4440	51-1H	85.6	1.15	-	B	Thr	Val	Leu	Leu	Val	Leu
4452	51-3D	100.0	1.30	-	B	Thr	Val	Val	Leu	Leu	Val
4477	51-6E	6.3	1.38	+	A	Val	Leu	Leu	Val	Val	Val
4493	51-8E	100.0	1.19	-	B	Leu	Val	Thr	Val	Val	Leu
4495	51-8G	3.5	1.26	++	A	Leu	Val	Val	Leu	Leu	Leu
4532	52-2D	100.0	1.11	-	B	Leu	Leu	Leu	Thr	Val	Val
4545	52-4A	100.0	1.18	-	B	Thr	Leu	Leu	Thr	Leu	Val
4603	52-11C	100.0	1.12	++	B	Val	Val	Val	Leu	Leu	Thr
4605	52-11E	100.0	1.35	-	B	Thr	Leu	Val	Leu	Leu	Val
4652	53-6D	100.0	1.03	-	B	Thr	Leu	Val	Val	Leu	Leu
4687	53-10G	94.4	1.04	-	B	Thr	Leu	Leu	Val	Leu	Leu
4690	53-11B	100.0	1.01	+	B	Leu	Leu	Thr	Leu	Leu	Val
4710	54-2F	100.0	1.06	-	B	Val	Leu	Thr	Val	Leu	Val
4778	54-11B	100.0	1.09	-	B	Leu	Val	Thr	Val	Val	Leu
4784	54-11H	100.0	1.19	-	B	Leu	Leu	Thr	Val	Val	Leu
4808	55-3H	100.0	1.02	-	B	Leu	Val	Leu	Val	Val	Asm
4830	55-6F	89.5	1.18	++	B	Val	Val	Val	Leu	Val	Val
4868	55-11D	100.0	1.06	-	B	Leu	Val	Leu	Thr	Leu	Thr
4884	56-2D	49.1	1.27	++	B	Leu	Val	Val	Val	Leu	Val
4895	56-3G	48.2	1.16	+	B	Leu	Val	Leu	Val	Leu	Val
4898	56-4B	100.0	1.21	-	B	Leu	Val	Val	Val	Leu	Asm
5006	57-6F	100.0	1.12	-	B	Leu	Leu	Leu	Leu	Leu	Thr
5056	58-1H	100.0	1.01	+	B	Leu	Val	Val	Val	Val	Leu
5058	58-2B	100.0	1.02	-	B	Leu	Val	Leu	Val	Leu	Thr
5091	58-6C	100.0	1.05	+	B	Leu	Leu	Val	Asm	Leu	Val
5164	59-4D	100.0	1.19	-	B	Val	Thr	Val	Val	Val	Leu
5167	59-4G	100.0	1.33	-	B	Leu	Val	Thr	Val	Leu	Val
5172	59-5D	100.0	1.27	++	B	Val	Val	Val	Leu	Leu	Thr
5178	59-6B	100.0	1.22	-	B	Leu	Leu	Leu	Val	Thr	Val
5185	59-7A	100.0	1.40	-	B	Leu	Val	Asm	Val	Leu	Val
5205	59-9E	100.0	1.22	-	B	Val	Val	Val	Thr	Val	Val

[a]The structures of the residues-4, -6, -8, -10, -12, and -14 are displayed as three-letter codes of the amino acids. H^+/Na^+ transport activity normalized against the value of **1** (1.00) and antibacterial activity are also listed. Antibacterial activity against *S. pyogenes* was evaluated using the three concentrations. +++: inhibition by the 640-fold diluted peptide solution, ++: inhibition by the 160-fold diluted solution, +: inhibition by the 40-fold diluted solution, -: no inhibition.

Supplementary Table 5. One-bead-derived peptides of groups A and B in plates 59−74[a]

bead number 1-13584	plate-well	activity			group	residue number					
		cell viability/%	H^+/Na^+ transport activity	antibacterial activity		4	6	8	10	12	14
5211	59-10C	100.0	1.05	-	B	Val	Asm	Val	Val	Leu	Leu
5215	59-10G	100.0	1.33	-	B	Thr	Leu	Leu	Thr	Leu	Leu
5241	60-3A	12.2	1.39	++	A	Val	Leu	Val	Leu	Val	Leu
5255	60-4G	100.0	1.25	-	B	Leu	Leu	Val	Asm	Val	Val
5284	60-8D	100.0	1.02	-	B	Thr	Leu	Leu	Asm	Leu	Leu
5302	60-10F	100.0	1.37	-	B	Leu	Val	Leu	Val	Leu	Asm
5311	60-11G	100.0	1.15	+	B	Val	Val	Leu	Leu	Thr	Val
5329	61-3A	100.0	1.26	-	B	Thr	Leu	Leu	Leu	Val	Leu
5343	61-4G	100.0	1.33	-	B	Leu	Thr	Val	Val	Val	Val
5370	61-8B	100.0	1.18	++	B	Val	Leu	Leu	Leu	Val	Thr
5380	61-9D	100.0	1.05	-	B	Val	Leu	Leu	Asm	Val	Val
5393	61-11A	100.0	1.32	+	B	Leu	Val	Val	Val	Leu	Val
5463	62-8G	100.0	1.11	++	B	Val	Leu	Val	Val	Leu	Leu
5482	62-11B	100.0	1.25	-	B	Leu	Val	Thr	Val	Leu	Leu
5488	62-11H	36.4	1.23	+	B	Leu	Leu	Val	Val	Val	Val
5491	63-1C	100.0	1.24	-	B	Leu	Leu	Asm	Leu	Leu	Val
5508	63-3D	100.0	1.19	-	B	Leu	Leu	Leu	Val	Leu	Thr
5522	63-5B	100.0	1.27	-	B	Thr	Leu	Val	Leu	Leu	Val
5526	63-5F	100.0	1.17	+	B	Val	Val	Leu	Val	Leu	Val
5532	63-6D	100.0	1.06	-	B	Thr	Leu	Leu	Thr	Val	Leu
5536	63-6H	100.0	1.45	-	B	Val	Val	Asm	Leu	Val	Val
5538	63-7B	100.0	1.06	-	B	Val	Val	Leu	Val	Leu	Asm
5542	63-7F	100.0	1.17	-	B	Val	Val	Thr	Val	Val	Leu
5555	63-9C	100.0	1.22	-	B	Leu	Thr	Val	Leu	Val	Leu
5561	63-10A	100.0	1.05	-	B	Leu	Leu	Leu	Thr	Leu	Val
5563	63-10C	100.0	1.08	-	B	Thr	Val	Leu	Leu	Val	Val
5570	63-11B	96.8	1.26	++	B	Val	Val	Val	Leu	Val	Val
5572	63-11D	100.0	1.24	+	B	Val	Leu	Leu	Thr	Val	Leu
5587	64-2C	85.3	1.23	++	B	Leu	Val	Val	Leu	Val	Val
5604	64-4D	69.7	1.03	-	B	Val	Val	Leu	Val	Val	Asm
5611	64-5C	3.9	1.17	+	A	Leu	Leu	Leu	Val	Val	Val
5618	64-6B	85.1	1.13	-	B	Thr	Leu	Leu	Leu	Leu	Leu
5623	64-6G	80.0	1.11	-	B	Thr	Val	Leu	Val	Val	Val
5657	64-11A	81.3	1.09	-	B	Leu	Val	Val	Thr	Leu	Val
5678	65-2F	93.2	1.11	++	B	Val	Val	Val	Val	Leu	Val
5679	65-2G	90.5	1.06	-	B	Leu	Leu	Leu	Thr	Val	Val
5699	65-5C	36.7	1.05	+	B	Val	Leu	Leu	Val	Leu	Leu
5736	65-9H	95.5	1.11	-	B	Leu	Leu	Leu	Val	Thr	Leu
5742	65-10F	11.1	1.16	++	A	Val	Val	Val	Leu	Leu	Val
5746	65-11B	100.0	1.26	-	B	Leu	Leu	Asm	Leu	Leu	Thr
5747	65-11C	100.0	1.09	-	B	Leu	Val	Thr	Thr	Leu	Val
5771	66-3C	79.3	1.46	-	B	Val	Val	Asm	Leu	Leu	Val
5778	66-4B	77.8	1.19	-	B	Leu	Val	Asm	Val	Leu	Val
5838	66-11F	100.0	1.12	-	B	Val	Leu	Val	Asm	Leu	Leu
5872	67-4H	9.9	1.02	+	A	Val	Leu	Val	Leu	Leu	Val
5933	68-1E	73.0	1.05	-	B	Thr	Val	Val	Val	Leu	Val
5991	68-8G	94.7	1.00	-	B	Val	Leu	Asm	Val	Val	Leu
5996	68-9D	78.7	1.08	-	B	Leu	Leu	Leu	Thr	Val	Val
6096	69-10H	7.3	1.16	+	A	Leu	Leu	Leu	Val	Leu	Val
6116	70-2D	93.5	1.13	-	B	Thr	Val	Leu	Val	Leu	Val
6160	70-7H	70.3	1.07	+	B	Val	Leu	Thr	Leu	Leu	Val
6165	70-8E	67.9	1.07	-	B	Val	Val	Val	Val	Leu	Val
6225	71-5A	80.8	1.10	-	B	Leu	Val	Thr	Val	Leu	Leu
6248	71-7H	87.7	1.10	-	B	Thr	Leu	Leu	Thr	Leu	Val
6265	71-10A	79.9	1.03	-	B	Leu	Leu	Leu	Val	Val	Asm
6292	72-2D	5.4	1.25	++	A	Leu	Val	Val	Leu	Leu	Leu
6329	72-7A	43.5	1.03	-	B	Val	Leu	Val	Val	Leu	Asm
6345	72-9A	57.3	1.13	++	B	Leu	Val	Val	Leu	Leu	Val
6360	72-10H	67.2	1.03	-	B	Val	Leu	Asm	Leu	Leu	Val
6510	74-7F	100.0	1.36	-	B	Val	Val	Asm	Leu	Leu	Val
6520	74-8H	100.0	1.08	-	B	Leu	Val	Leu	Val	Val	Asm
6541	74-11E	100.0	1.14	-	B	Val	Leu	Asm	Leu	Val	Leu

[a]The structures of the residues-4, -6, -8, -10, -12, and -14 are displayed as three-letter codes of the amino acids. H^+/Na^+ transport activity normalized against the value of **1** (1.00) and antibacterial activity are also listed. Antibacterial activity against *S. pyogenes* was evaluated using the three concentrations. +++: inhibition by the 640-fold diluted peptide solution, ++: inhibition by the 160-fold diluted solution, +: inhibition by the 40-fold diluted solution, -: no inhibition.

Supplementary Table 6. One-bead-derived peptides of groups A and B in plates 75−86[a]

bead number 1-13584	plate-well	activity			group	residue number					
		cell viability/%	H^+/Na^+ transport activity	antibacterial activity		4	6	8	10	12	14
6549	75-1E	100.0	1.07	-	B	Leu	Thr	Val	Leu	Val	Val
6572	75-4D	100.0	1.09	-	B	Val	Leu	Leu	Thr	Val	Leu
6606	75-8F	100.0	1.03	-	B	Thr	Val	Val	Val	Val	Leu
6639	76-1G	0.0	1.01	+	A	Leu	Leu	Leu	Leu	Leu	Val
6702	76-9F	100.0	1.03	+	B	Leu	Val	Thr	Leu	Leu	Val
6703	76-9G	100.0	1.17	-	B	Leu	Leu	Asm	Val	Leu	Leu
6766	77-6F	100.0	1.07	+	B	Leu	Val	Thr	Leu	Leu	Leu
6781	77-8E	100.0	1.04	+	B	Val	Val	Leu	Val	Val	Thr
6797	77-10E	100.0	1.02	-	B	Thr	Leu	Leu	Val	Leu	Val
6808	77-11H	100.0	1.02	-	B	Leu	Val	Leu	Val	Val	Asm
6820	78-2D	100.0	1.20	-	B	Thr	Val	Val	Val	Leu	Val
6822	78-2F	100.0	1.23	-	B	Val	Leu	Asm	Leu	Val	Val
6829	78-3E	100.0	1.05	-	B	Leu	Leu	Val	Leu	Leu	Asm
6842	78-5B	100.0	1.14	-	B	Val	Leu	Thr	Leu	Val	Leu
6843	78-5C	100.0	1.15	-	B	Thr	Val	Val	Leu	Leu	Val
6851	78-6C	100.0	1.16	-	B	Leu	Leu	Asm	Leu	Leu	Val
6857	78-7A	100.0	1.16	-	B	Thr	Leu	Val	Val	Leu	Val
6861	78-7E	100.0	1.18	+	B	Leu	Val	Val	Val	Leu	Val
6862	78-7F	100.0	1.31	-	B	Val	Val	Asm	Leu	Leu	Leu
6873	78-9A	100.0	1.15	-	B	Leu	Thr	Val	Leu	Leu	Thr
6876	78-9D	100.0	1.13	-	B	Leu	Leu	Val	Val	Thr	Leu
6888	78-10H	78.6	1.23	+	B	Val	Leu	Val	Leu	Val	Val
6889	78-11A	100.0	1.10	-	B	Leu	Leu	Val	Asm	Leu	Val
6896	78-11H	100.0	1.18	-	B	Thr	Leu	Val	Thr	Leu	Leu
6899	79-1C	100.0	1.12	-	B	Val	Val	Asm	Leu	Leu	Val
6943	79-6G	100.0	1.02	-	B	Val	Leu	Leu	Leu	Val	Thr
6950	79-7F	100.0	1.06	-	B	Leu	Leu	Val	Thr	Val	Leu
6959	79-8G	100.0	1.06	+	B	Val	Leu	Leu	Val	Val	Thr
7010	80-4B	100.0	1.07	+	B	Leu	Val	Val	Leu	Val	Thr
7015	80-4G	100.0	1.07	-	B	Thr	Leu	Leu	Thr	Val	Leu
7049	80-9A	100.0	1.10	-	B	Val	Val	Val	Leu	Leu	Asm
7050	80-9B	100.0	1.21	-	B	Val	Val	Leu	Val	Val	Thr
7061	80-10E	100.0	1.01	-	B	Val	Thr	Val	Val	Leu	Val
7067	80-11C	100.0	1.02	-	B	Leu	Leu	Val	Thr	Leu	Leu
7100	81-4D	100.0	1.07	-	B	Leu	Leu	Val	Val	Val	Val
7119	81-6G	100.0	1.15	-	B	Leu	Asm	Leu	Leu	Val	Val
7131	81-8C	100.0	1.08	-	B	Val	Leu	Thr	Val	Leu	Val
7135	81-8G	100.0	1.09	-	B	Thr	Leu	Leu	Thr	Leu	Val
7146	81-10B	100.0	1.17	+	B	Val	Leu	Val	Leu	Val	Leu
7156	81-11D	100.0	1.08	-	B	Leu	Leu	Leu	Leu	Thr	Thr
7169	82-2A	90.3	1.15	+	B	Val	Leu	Val	Val	Leu	Leu
7191	82-4G	0.0	1.15	+	A	Leu	Val	Leu	Leu	Val	Leu
7198	82-5F	16.1	1.09	+	A	Leu	Leu	Val	Val	Leu	Leu
7204	82-6D	100.0	1.17	-	B	Leu	Val	Asm	Leu	Leu	Val
7211	82-7C	100.0	1.03	-	B	Leu	Leu	Leu	Thr	Leu	Leu
7225	82-9A	100.0	1.14	-	B	Thr	Leu	Val	Thr	Val	Leu
7228	82-9D	100.0	1.06	-	B	Val	Leu	Val	Leu	Val	Asm
7253	83-1E	45.4	1.03	-	B	Leu	Leu	Leu	Val	Thr	Thr
7271	83-3G	10.6	1.02	-	A	Val	Val	Leu	Val	Val	Asm
7320	83-9H	58.5	1.04	-	B	Val	Thr	Leu	Val	Val	Leu
7325	83-10E	82.2	1.04	-	B	Val	Leu	Leu	Thr	Val	Leu
7334	83-11F	94.6	1.11	-	B	Val	Leu	Leu	Leu	Val	Asm
7393	84-8A	100.0	1.07	-	B	Val	Leu	Val	Val	Leu	Thr
7400	84-8H	100.0	1.30	-	B	Leu	Leu	Asm	Leu	Val	Leu
7408	84-9H	19.4	1.06	-	A	Leu	Leu	Leu	Val	Val	Val
7437	85-2E	100.0	1.11	-	B	Val	Val	Asm	Leu	Leu	Leu
7479	85-7G	100.0	1.00	-	B	Val	Leu	Val	Val	Val	Asm
7480	85-7H	100.0	1.01	-	B	Leu	Leu	Leu	Thr	Leu	Val
7512	85-11H	100.0	1.05	-	B	Leu	Leu	Leu	Leu	Val	Thr
7516	86-1D	100.0	1.03	+	B	Leu	Val	Val	Val	Val	Val
7532	86-3D	3.9	1.03	+	A	Leu	Val	Leu	Val	Leu	Leu
7548	86-5D	100.0	1.08	-	B	Leu	Leu	Thr	Val	Val	Leu

[a]The structures of the residues-4, -6, -8, -10, -12, and -14 are displayed as three-letter codes of the amino acids. H^+/Na^+ transport activity normalized against the value of **1** (1.00) and antibacterial activity are also listed. Antibacterial activity against *S. pyogenes* was evaluated using the three concentrations. +++: inhibition by the 640-fold diluted peptide solution, ++: inhibition by the 160-fold diluted solution, +: inhibition by the 40-fold diluted solution, -: no inhibition.

Supplementary Table 7. One-bead-derived peptides of groups A and B in plates 88−103[a]

bead number 1-13584	plate-well	activity			group	residue number					
		cell viability/%	H^+/Na^+ transport activity	antibacterial activity		4	6	8	10	12	14
7690	88-1B	100.0	1.03	-	B	Val	Leu	Val	Val	Val	Val
7702	88-2F	100.0	1.01	-	B	Val	Val	Thr	Leu	Val	Leu
7808	89-4H	100.0	1.21	-	B	Leu	Val	Asm	Val	Leu	Leu
7840	89-8H	100.0	1.12	-	B	Val	Leu	Thr	Val	Leu	Leu
7845	89-9E	100.0	1.04	+	B	Leu	Leu	Val	Val	Val	Val
7864	89-11H	10.8	1.01	+	A	Val	Val	Leu	Leu	Leu	Leu
7890	90-4B	100.0	1.11	-	B	Thr	Leu	Leu	Val	Leu	Leu
7905	90-6A	100.0	1.10	-	B	Thr	Leu	Leu	Leu	Leu	Leu
7912	90-6H	100.0	1.12	-	B	Val	Thr	Val	Leu	Val	Leu
7938	90-10B	17.4	1.10	+	A	Leu	Val	Leu	Val	Val	Val
7948	90-11D	100.0	1.04	-	B	Leu	Val	Val	Leu	Val	Asm
8038	91-11F	100.0	1.04	-	B	Asm	Leu	Val	Leu	Leu	Leu
8043	92-1C	100.0	1.04	-	B	Leu	Leu	Thr	Val	Leu	Val
8051	92-2C	16.8	1.13	+	A	Leu	Val	Leu	Val	Val	Leu
8057	92-3A	100.0	1.13	-	B	Val	Val	Asm	Leu	Val	Val
8061	92-3E	100.0	1.05	-	B	Leu	Leu	Asm	Leu	Leu	Leu
8130	93-1B	100.0	1.17	-	B	Leu	Leu	Thr	Leu	Val	Val
8141	93-2E	100.0	1.03	-	B	Val	Thr	Val	Leu	Leu	Val
8265	94-7A	68.3	1.12	-	B	Leu	Asm	Val	Leu	Val	Leu
8288	94-9H	47.4	1.02	-	B	Leu	Thr	Leu	Leu	Leu	Val
8342	95-5F	54.5	1.00	-	B	Leu	Leu	Val	Thr	Leu	Leu
8374	95-9F	66.4	1.02	-	B	Val	Leu	Thr	Thr	Leu	Leu
8397	96-1E	0.0	1.12	+	A	Leu	Val	Leu	Leu	Leu	Leu
8400	96-1H	34.4	1.01	-	B	Val	Val	Leu	Val	Leu	Asm
8459	96-9C	47.5	1.03	-	B	Val	Leu	Asm	Val	Val	Val
8464	96-9H	4.1	1.23	-	A	Leu	Val	Leu	Val	Leu	Leu
8473	96-11A	53.6	1.01	-	B	Val	Leu	Val	Leu	Thr	Val
8498	97-3B	52.6	1.01	-	B	Leu	Leu	Val	Val	Thr	Leu
8516	97-5D	48.7	1.09	-	B	Leu	Leu	Val	Leu	Val	Asm
8535	97-7G	71.4	1.28	-	B	Leu	Asm	Val	Leu	Val	Leu
8545	97-9A	83.1	1.01	-	B	Thr	Val	Val	Val	Val	Val
8554	97-10B	61.3	1.03	-	B	Val	Leu	Leu	Thr	Val	Val
8567	97-11G	60.9	1.22	-	B	Leu	Val	Leu	Val	Val	Asm
8580	98-2D	60.6	1.01	+	B	Val	Val	Leu	Val	Leu	Thr
8583	98-2G	63.4	1.15	-	B	Leu	Asm	Val	Val	Leu	Val
8596	98-4D	56.2	1.07	-	B	Val	Val	Thr	Leu	Val	Val
8600	98-4H	55.3	1.20	-	B	Leu	Leu	Asm	Leu	Val	Leu
8604	98-5D	54.4	1.08	-	B	Leu	Leu	Thr	Leu	Val	Leu
8633	98-9A	61.8	1.00	-	B	Val	Val	Leu	Val	Thr	Leu
8635	98-9C	6.4	1.05	+	A	Leu	Leu	Leu	Leu	Leu	Val
8662	99-1F	61.0	1.08	-	B	Val	Leu	Asm	Val	Val	Val
8687	99-4G	66.9	1.15	-	B	Thr	Leu	Val	Val	Leu	Val
8697	99-6A	100.0	1.04	+	B	Val	Leu	Val	Leu	Val	Val
8722	99-9B	70.8	1.09	-	B	Leu	Leu	Leu	Val	Thr	Val
8731	99-10C	71.2	1.05	-	B	Val	Leu	Val	Val	Leu	Thr
8732	99-10D	72.4	1.11	-	B	Leu	Val	Asm	Val	Leu	Val
8749	100-1E	62.2	1.05	-	B	Leu	Leu	Val	Val	Thr	Val
8755	100-2C	67.3	1.10	-	B	Leu	Thr	Val	Val	Leu	Leu
8779	100-5C	40.6	1.07	-	B	Val	Leu	Thr	Leu	Val	Val
8796	100-7D	74.1	1.15	+	B	Leu	Val	Leu	Val	Val	Val
8827	100-11C	80.7	1.22	-	B	Thr	Val	Leu	Val	Leu	Leu
8831	100-11G	81.1	1.23	-	B	Leu	Val	Asm	Leu	Leu	Val
8858	101-4B	84.7	1.05	+	B	Leu	Leu	Val	Val	Leu	Leu
8906	101-10B	79.1	1.08	+	B	Leu	Val	Leu	Val	Leu	Thr
8909	101-10E	85.9	1.04	+	B	Val	Leu	Val	Val	Val	Val
8969	102-7A	8.7	1.09	-	A	Val	Val	Leu	Leu	Val	Val
8982	102-8F	49.1	1.21	-	B	Val	Val	Asm	Leu	Leu	Leu
8984	102-8H	57.9	1.12	-	B	Val	Leu	Thr	Leu	Leu	Val
8992	102-9H	56.1	1.07	-	B	Leu	Leu	Asm	Leu	Leu	Thr
8999	102-10G	96.1	1.03	+	B	Val	Val	Leu	Val	Leu	Leu
9000	102-10H	7.4	1.21	+	A	Leu	Val	Val	Leu	Leu	Leu
9030	103-3F	71.3	1.12	-	B	Leu	Thr	Val	Val	Leu	Val

[a]The structures of the residues-4, -6, -8, -10, -12, and -14 are displayed as three-letter codes of the amino acids. H^+/Na^+ transport activity normalized against the value of **1** (1.00) and antibacterial activity are also listed. Antibacterial activity against *S. pyogenes* was evaluated using the three concentrations. +++: inhibition by the 640-fold diluted peptide solution, ++: inhibition by the 160-fold diluted solution, +: inhibition by the 40-fold diluted solution, -: no inhibition.

Supplementary Table 8. One-bead-derived peptides of groups A and B in plates 103−120[a]

bead number 1-13584	plate-well	activity			group	residue number					
		cell viability/%	H^+/Na^+ transport activity	antibacterial activity		4	6	8	10	12	14
9036	103-4D	94.7	1.18	+	B	Leu	Val	Val	Val	Leu	Val
9055	103-6G	83.5	1.01	-	B	Val	Thr	Val	Leu	Val	Val
9058	103-7B	58.0	1.14	-	B	Val	Thr	Val	Val	Val	Leu
9075	103-9C	58.6	1.01	-	B	Thr	Val	Leu	Val	Val	Leu
9098	104-1B	51.0	1.13	-	B	Leu	Thr	Val	Leu	Leu	Leu
9112	104-2H	72.6	1.10	-	B	Thr	Leu	Val	Val	Leu	Leu
9125	104-4E	44.2	1.04	-	B	Thr	Leu	Leu	Leu	Val	Val
9145	104-7A	70.9	1.03	-	B	Thr	Leu	Leu	Val	Val	Leu
9177	104-11A	61.7	1.05	-	B	Val	Leu	Val	Leu	Thr	Leu
9192	105-1H	100.0	1.07	-	B	Val	Leu	Val	Val	Val	Leu
9223	105-5G	100.0	1.03	+	B	Leu	Val	Val	Val	Val	Leu
9229	105-6E	100.0	1.15	-	B	Val	Leu	Asm	Leu	Leu	Val
9247	105-8G	6.0	1.02	+	A	Val	Leu	Leu	Leu	Val	Val
9293	106-3E	4.3	1.05	++	A	Val	Val	Val	Leu	Val	Leu
9336	106-8H	100.0	1.18	-	B	Leu	Val	Asm	Leu	Leu	Leu
9352	106-10H	100.0	1.18	-	B	Leu	Asm	Val	Leu	Val	Leu
9354	106-11B	100.0	1.16	-	B	Thr	Val	Val	Val	Val	Leu
9473	108-4A	0.0	1.04	-	A	Leu	Leu	Leu	Leu	Val	Leu
9487	108-5G	100.0	1.15	-	B	Val	Leu	Thr	Leu	Val	Val
9521	108-10A	100.0	1.14	-	B	Leu	Val	Asm	Val	Leu	Val
9535	108-11G	3.8	1.16	-	A	Val	Leu	Val	Leu	Leu	Val
9571	109-5C	100.0	1.14	+	B	Val	Val	Val	Val	Leu	Val
9607	109-9G	48.6	1.03	+	B	Leu	Leu	Val	Leu	Val	Val
9609	109-10A	100.0	1.15	-	B	Leu	Leu	Val	Val	Leu	Asm
9610	109-10B	100.0	1.14	+	B	Leu	Val	Val	Val	Val	Val
9628	110-1D	0.0	1.16	+	A	Leu	Val	Leu	Leu	Leu	Leu
9633	110-2A	100.0	1.06	-	B	Val	Leu	Thr	Val	Leu	Val
9676	110-7D	100.0	1.05	-	B	Leu	Thr	Leu	Leu	Val	Leu
9727	111-2G	100.0	1.10	-	B	Val	Leu	Val	Leu	Val	Asm
9735	111-3G	100.0	1.18	-	B	Leu	Leu	Val	Val	Val	Thr
9738	111-4B	100.0	1.21	-	B	Val	Leu	Thr	Val	Val	Leu
9742	111-4F	0.0	1.21	+	A	Val	Leu	Leu	Val	Leu	Val
9799	111-11G	100.0	1.26	-	B	Thr	Val	Leu	Val	Val	Val
9823	112-3G	100.0	1.08	-	B	Leu	Leu	Val	Val	Leu	Thr
9832	112-4H	100.0	1.26	-	B	Leu	Val	Thr	Val	Leu	Leu
9897	113-2A	8.1	1.04	+	A	Val	Leu	Val	Leu	Leu	Leu
9910	113-3F	100.0	1.07	-	B	Val	Val	Thr	Leu	Val	Val
9992	114-2H	100.0	1.23	+	B	Leu	Val	Val	Val	Val	Val
10002	114-4B	100.0	1.01	-	B	Leu	Leu	Val	Val	Val	Asm
10008	114-4H	100.0	1.15	-	B	Leu	Leu	Leu	Leu	Val	Asm
10042	114-9B	100.0	1.06	-	B	Val	Val	Val	Thr	Leu	Leu
10043	114-9C	56.6	1.09	-	B	Leu	Val	Leu	Val	Leu	Asm
10261	117-3E	46.3	1.05	+	B	Val	Leu	Val	Leu	Val	Leu
10276	117-5D	100.0	1.04	+	B	Val	Val	Val	Val	Val	Val
10313	117-10A	100.0	1.11	-	B	Thr	Leu	Val	Leu	Val	Leu
10338	118-2B	100.0	1.01	-	B	Thr	Leu	Val	Thr	Leu	Val
10340	118-2D	3.4	1.00	-	A	Leu	Leu	Leu	Leu	Leu	Val
10353	118-4A	100.0	1.02	-	B	Val	Val	Thr	Leu	Leu	Leu
10367	118-5G	100.0	1.00	-	B	Val	Leu	Thr	Val	Leu	Leu
10379	118-7C	100.0	1.08	-	B	Leu	Leu	Asm	Leu	Val	Val
10400	118-9H	100.0	1.04	-	B	Leu	Val	Thr	Leu	Val	Val
10415	118-11G	100.0	1.02	-	B	Leu	Thr	Val	Val	Leu	Val
10442	119-4B	100.0	1.15	-	B	Leu	Leu	Leu	Thr	Val	Leu
10456	119-5H	11.8	1.13	+	A	Leu	Leu	Val	Val	Leu	Val
10497	119-11A	100.0	1.24	-	B	Thr	Leu	Leu	Leu	Leu	Leu
10499	119-11C	100.0	1.14	-	B	Leu	Leu	Leu	Thr	Leu	Val
10500	119-11D	100.0	1.14	-	B	Thr	Val	Leu	Val	Val	Leu
10506	120-1B	91.3	1.18	-	B	Val	Val	Thr	Val	Leu	Val
10516	120-2D	100.0	1.10	-	B	Val	Val	Leu	Leu	Thr	Leu
10517	120-2E	100.0	1.05	-	B	Val	Leu	Thr	Thr	Leu	Leu
10536	120-4H	100.0	1.21	+	B	Leu	Leu	Val	Val	Val	Val
10542	120-5F	100.0	1.02	-	B	Leu	Leu	Val	Thr	Val	Leu

[a]The structures of the residues-4, -6, -8, -10, -12, and -14 are displayed as three-letter codes of the amino acids. H^+/Na^+ transport activity normalized against the value of **1** (1.00) and antibacterial activity are also listed. Antibacterial activity against *S. pyogenes* was evaluated using the three concentrations. +++: inhibition by the 640-fold diluted peptide solution, ++: inhibition by the 160-fold diluted solution, +: inhibition by the 40-fold diluted solution, -: no inhibition.

Supplementary Table 9. One-bead-derived peptides of groups A and B in plates 121−140[a]

bead number 1-13584	plate-well	activity			group	residue number					
		cell viability/%	H^+/Na^+ transport activity	antibacterial activity		4	6	8	10	12	14
10593	121-1A	38.5	1.10	+	B	Val	Leu	Val	Leu	Leu	Leu
10609	121-3A	79.4	1.15	-	B	Leu	Leu	Thr	Val	Val	Val
10649	121-8A	100.0	1.19	++	B	Val	Val	Val	Val	Leu	Leu
10653	121-8E	44.1	1.04	-	B	Thr	Val	Val	Val	Leu	Val
10661	121-9E	39.9	1.03	+	B	Val	Val	Leu	Val	Leu	Leu
10668	121-10D	57.1	1.09	+	B	Val	Val	Leu	Val	Val	Leu
10669	121-10E	55.2	1.11	-	B	Leu	Val	Asm	Leu	Val	Leu
10673	121-11A	97.7	1.01	-	B	Asm	Val	Val	Leu	Leu	Leu
10674	121-11B	87.3	1.08	+	B	Leu	Val	Val	Thr	Leu	Leu
10678	121-11F	76.0	1.10	-	B	Leu	Leu	Val	Val	Leu	Asm
10686	122-1F	100.0	1.05	-	B	Val	Thr	Leu	Leu	Val	Leu
10699	122-3C	100.0	1.09	-	B	Val	Val	Val	Val	Val	Leu
10710	122-4F	100.0	1.05	-	B	Leu	Leu	Thr	Val	Leu	Leu
11126	127-1F	8.7	1.07	-	A	Val	Leu	Leu	Val	Val	Leu
11143	127-3G	63.4	1.19	-	B	Val	Leu	Thr	Val	Val	Leu
11146	127-4B	100.0	1.08	+	B	Val	Val	Leu	Val	Leu	Leu
11198	127-10F	3.2	1.08	-	A	Leu	Leu	Leu	Val	Leu	Leu
11199	127-10G	65.2	1.00	-	B	Thr	Val	Val	Leu	Val	Leu
11200	127-10H	66.0	1.06	-	B	Asm	Val	Val	Leu	Leu	Val
11214	128-1F	2.6	1.02	-	A	Leu	Leu	Leu	Leu	Val	Val
11226	128-3B	76.8	1.04	-	B	Val	Leu	Asm	Val	Val	Leu
11228	128-3D	76.4	1.27	-	B	Leu	Leu	Asm	Val	Val	Leu
11238	128-4F	64.7	1.03	-	B	Thr	Val	Leu	Val	Leu	Val
11268	128-8D	100.0	1.06	+	B	Val	Leu	Val	Val	Leu	Val
11280	128-9H	9.9	1.04	-	A	Val	Leu	Leu	Leu	Val	Val
11290	128-11B	87.6	1.47	-	B	Leu	Asm	Val	Leu	Val	Val
11295	128-11G	78.1	1.12	-	B	Leu	Leu	Thr	Leu	Leu	Val
11302	129-1F	49.1	1.05	-	B	Leu	Thr	Leu	Leu	Leu	Val
11305	129-2A	15.5	1.26	+	A	Leu	Leu	Val	Leu	Val	Leu
11317	129-3E	42.1	1.02	-	B	Thr	Val	Leu	Leu	Leu	Val
11323	129-4C	5.2	1.02	-	A	Leu	Leu	Leu	Val	Val	Val
11349	129-7E	34.3	1.00	-	B	Val	Leu	Asm	Leu	Val	Val
11368	129-9H	61.1	1.01	-	B	Val	Val	Leu	Val	Leu	Asm
11374	129-10F	45.1	1.07	+	B	Val	Leu	Val	Leu	Thr	Leu
11383	129-11G	55.3	1.09	-	B	Val	Leu	Asm	Val	Val	Val
11400	130-2H	53.0	1.03	+	B	Val	Leu	Leu	Thr	Leu	Leu
11401	130-3A	56.6	1.09	-	B	Leu	Leu	Val	Leu	Leu	Asm
11408	130-3H	57.5	1.30	-	B	Leu	Leu	Leu	Thr	Leu	Leu
11413	130-4E	45.4	1.21	-	B	Val	Val	Asm	Val	Val	Leu
11422	130-5F	47.4	1.04	+	B	Leu	Val	Val	Leu	Leu	Asm
11425	130-6A	41.5	1.12	+	B	Leu	Val	Leu	Leu	Thr	Leu
11433	130-7A	51.1	1.38	-	B	Val	Leu	Thr	Val	Leu	Leu
11438	130-7F	100.0	1.18	++	B	Leu	Val	Val	Val	Leu	Val
11439	130-7G	8.1	1.18	-	A	Val	Leu	Leu	Val	Leu	Leu
11453	130-9E	49.2	1.04	-	B	Thr	Val	Val	Val	Val	Val
11561	132-1A	100.0	1.01	-	B	Thr	Leu	Leu	Val	Leu	Leu
11601	132-6A	98.9	1.23	-	B	Leu	Leu	Asm	Val	Val	Leu
11625	132-9A	100.0	1.08	-	B	Leu	Val	Leu	Val	Val	Thr
11660	133-2D	100.0	1.09	-	B	Leu	Leu	Leu	Thr	Val	Leu
11772	134-5D	100.0	1.04	-	B	Thr	Val	Leu	Leu	Val	Val
11796	134-8D	100.0	1.33	-	B	Val	Val	Asm	Leu	Leu	Val
12040	137-5H	100.0	1.07	+	B	Leu	Leu	Val	Val	Val	Leu
12081	137-11A	100.0	1.01	-	B	Leu	Leu	Val	Thr	Val	Leu
12092	138-1D	100.0	1.14	+	B	Leu	Val	Val	Val	Val	Val
12099	138-2C	85.4	1.05	+	B	Val	Leu	Val	Val	Leu	Leu
12185	139-2A	98.1	1.02	+	B	Leu	Val	Thr	Leu	Val	Leu
12283	140-3C	100.0	1.08	-	B	Val	Leu	Thr	Val	Val	Val
12285	140-3E	100.0	1.01	-	B	Val	Leu	Val	Val	Leu	Thr
12287	140-3G	100.0	1.16	-	B	Val	Val	Thr	Leu	Leu	Leu
12293	140-4E	100.0	1.00	-	B	Val	Val	Thr	Thr	Leu	Leu
12298	140-5B	100.0	1.04	-	B	Thr	Val	Val	Leu	Val	Val
12307	140-6C	100.0	1.32	-	B	Leu	Leu	Asm	Val	Val	Leu

[a]The structures of the residues-4, -6, -8, -10, -12, and -14 are displayed as three-letter codes of the amino acids. H^+/Na^+ transport activity normalized against the value of **1** (1.00) and antibacterial activity are also listed. Antibacterial activity against *S. pyogenes* was evaluated using the three concentrations. +++: inhibition by the 640-fold diluted peptide solution, ++: inhibition by the 160-fold diluted solution, +: inhibition by the 40-fold diluted solution, -: no inhibition.

Supplementary Table 10. One-bead-derived peptides of groups A and B in plates 141−151[a]

bead number 1-13584	plate-well	activity			group	residue number					
		cell viability/%	H^+/Na^+ transport activity	antibacterial activity		4	6	8	10	12	14
12373	141-3E	100.0	1.04	-	B	Val	Val	Asm	Leu	Leu	Val
12429	141-10E	100.0	1.03	-	B	Leu	Val	Thr	Val	Val	Val
12432	141-10H	6.4	1.01	-	A	Val	Val	Leu	Leu	Val	Leu
12538	143-2B	2.1	1.01	-	A	Val	Val	Leu	Val	Leu	Leu
12576	143-6H	100.0	1.09	-	B	Val	Leu	Asm	Val	Val	Val
12606	143-10F	100.0	1.05	-	B	Val	Val	Val	Val	Val	Leu
12629	144-2E	100.0	1.01	-	B	Thr	Val	Val	Thr	Leu	Val
12636	144-3D	100.0	1.21	-	B	Leu	Val	Asm	Val	Val	Leu
12687	144-9G	100.0	1.29	-	B	Val	Leu	Asm	Leu	Val	Leu
12691	144-10C	100.0	1.11	-	B	Val	Val	Leu	Val	Val	Asm
12707	145-1C	100.0	1.06	-	B	Val	Leu	Asm	Leu	Val	Val
12721	145-3A	51.7	1.04	+	B	Val	Val	Leu	Val	Val	Leu
12789	145-11E	9.6	1.04	-	A	Val	Leu	Val	Leu	Leu	Val
12865	146-10A	91.7	1.10	-	B	Val	Leu	Val	Thr	Val	Leu
12877	146-11E	4.7	1.04	-	A	Val	Leu	Leu	Leu	Leu	Leu
12938	147-8B	100.0	1.07	-	B	Val	Asm	Val	Leu	Val	Leu
12947	147-9C	10.2	1.02	+	A	Val	Val	Leu	Val	Leu	Val
12950	147-9F	97.0	1.08	+	B	Val	Leu	Val	Val	Leu	Val
12972	148-1D	0.0	1.33	++	A	Leu	Val	Val	Leu	Leu	Leu
12975	148-1G	86.0	1.43	-	B	Val	Leu	Asm	Val	Leu	Leu
12984	148-2H	81.1	1.08	-	B	Leu	Val	Leu	Val	Val	Thr
12989	148-3E	100.0	1.16	+	B	Leu	Leu	Leu	Leu	Thr	Val
13002	148-5B	98.3	1.33	-	B	Leu	Thr	Val	Leu	Leu	Val
13030	148-8F	99.9	1.42	+	B	Val	Leu	Val	Val	Val	Val
13033	148-9A	100.0	1.01	-	B	Leu	Leu	Leu	Thr	Leu	Asm
13040	148-9H	100.0	1.01	-	B	Val	Leu	Val	Leu	Thr	Thr
13062	149-1F	92.8	1.06	-	B	Val	Thr	Val	Val	Val	Leu
13123	149-9C	100.0	1.24	-	B	Leu	Asm	Val	Leu	Val	Leu
13128	149-9H	100.0	1.24	+	B	Leu	Leu	Val	Val	Val	Val
13147	150-1C	94.4	1.09	-	B	Val	Leu	Leu	Val	Val	Thr
13148	150-1D	93.6	1.03	-	B	Val	Asm	Leu	Val	Val	Val
13151	150-1G	100.0	1.01	-	B	Val	Thr	Val	Leu	Leu	Leu
13174	150-4F	100.0	1.18	-	B	Val	Leu	Thr	Val	Leu	Val
13191	150-6G	100.0	1.05	-	B	Thr	Leu	Leu	Thr	Val	Val
13245	151-2E	100.0	1.07	-	B	Thr	Val	Leu	Thr	Leu	Val
13252	151-3D	2.6	1.14	-	A	Leu	Val	Leu	Leu	Val	Leu
13268	151-5D	100.0	1.03	+	B	Leu	Val	Thr	Leu	Leu	Val
13273	151-6A	100.0	1.05	-	B	Leu	Leu	Val	Leu	Thr	Val
13317	151-11E	100.0	1.04	-	B	Val	Leu	Val	Val	Leu	Asm

[a]The structures of the residues-4, -6, -8, -10, -12, and -14 are displayed as three-letter codes of the amino acids. H^+/Na^+ transport activity normalized against the value of **1** (1.00) and antibacterial activity are also listed. Antibacterial activity against *S. pyogenes* was evaluated using the three concentrations. +++: inhibition by the 640-fold diluted peptide solution, ++: inhibition by the 160-fold diluted solution, +: inhibition by the 40-fold diluted solution, -: no inhibition.

Supplementary Table 11. ^{1}H NMR chemical shifts of gramicidin A, **A1**, and $\mathbf{B_13}$[a]

residue	position	**1**	residue	position	**A1**	residue	position	$\mathbf{B_13}$
formyl	CHO	8.07	formyl	CHO	8.09	formyl	CHO	8.07
L-Val-1	NHα	8.16	L-Val-1	NHα	8.16	L-Val-1	NHα	8.18
	Hα	4.25		Hα	4.26		Hα	4.22
	Hβ	2.00		Hβ	2.02		Hβ	2.01
	Hγ	0.86		Hγ	0.87		Hγ	0.87
Gly-2	NHα	8.24	Gly-2	NHα	8.25	Gly-2	NHα	8.25
	Hα	3.75		Hα	3.75		Hα	3.74
L-Ala-3	NHα	7.94	L-Ala-3	NHα	7.93	L-Ala-3	NHα	7.94
	Hα	4.29		Hα	4.39		Hα	4.26
	Hβ	1.21		Hβ	1.24		Hβ	1.22
D-Leu-4	NHα	8.05	D-Val-4	NHα	7.88	D-Leu-4	NHα	8.05
	Hα	4.26		Hα	4.17		Hα	4.24
	Hβ	1.48		Hβ	2.00		Hβ	1.48
	Hγ	1.57		Hγ	0.82		Hγ	1.56
	Hδ	0.86					Hδ	0.84
L-Ala-5	NHα	7.88	L-Ala-5	NHα	8.00	L-Ala-5	NHα	7.88
	Hα	4.30		Hα	4.39		Hα	4.31
	Hβ	1.21		Hβ	1.24		Hβ	1.23
D-Val-6	NHα	7.69	D-Val-6	NHα	7.74	D-Val-6	NHα	7.78
	Hα	4.32		Hα	4.32		Hα	4.30
	Hβ	2.03		Hβ	2.02		Hβ	2.05
	Hγ	0.81		Hγ	0.82		Hγ	0.84
L-Val-7	NHα	7.88	L-Val-7	NHα	7.90	L-Val-7	NH	7.95
	Hα	4.30		Hα	4.30		Hα	4.31
	Hβ	2.02		Hβ	2.00		Hβ	2.09
	Hγ	0.81		Hγ	0.82		Hγ	0.84
D-Val-8	NHα	7.73	D-Val-8	NHα	7.76	D-Thr-8	NHα	7.80
	Hα	4.17		Hα	4.18		Hα	4.15
	Hβ	1.84		Hβ	1.86		Hβ	3.88
	Hγ	0.60		Hγ	0.61		Hγ	0.84
							OH	ND[c]
L-Trp-9	NHα	8.11	L-Trp-9	NHα	8.11	L-Trp-9	NHα	8.11
	Hα	4.55		Hα	4.56		Hα	4.50
	Hβ	2.94, 3.19		Hβ	2.96, 3.18		Hβ	2.94, 3.18
	Hδ	7.09		Hδ	7.10		Hδ	7.10
	Hε1 (NH)	10.71 or 10.67		Hε1 (NH)	10.7		Hε1 (NH)	10.6 or 10.7
	Hζ1	7.30		Hζ1	7.30		Hζ1	7.30
	Hη2	7.03		Hη2	7.03		Hη2	7.03
	Hζ3	6.94		Hζ3	6.95		Hζ3	6.94
	Hε2	7.55		Hε2	7.56		Hε2	7.55
D-Leu-10	NHα	7.88	D-Leu-10	NHα	7.88	D-Leu-10	NHα	7.83 or 7.95
	Hα	4.17		Hα	4.17		Hα	4.15
	Hβ	1.13		Hβ	1.13–1.19		Hβ	1.15 or 1.22
	Hγ	0.95		Hγ	0.96		Hγ	0.94 or 0.98 or 1.02
	Hδ	0.60 (and/or 0.54)		Hδ	0.61 or 0.53		Hδ	0.60
L-Trp-11	NHα	8.06	L-Trp-11	NHα	8.08	L-Trp-11	NHα	8.00
	Hα	4.58		Hα	4.59		Hα	4.51
	Hβ	2.94, 3.16		Hβ	2.96, 3.18		Hβ	2.93, 3.18
	Hδ	7.09		Hδ	7.10		Hδ	7.10
	Hε1 (NH)	10.71 or 10.67		Hε1 (NH)	10.7		Hε1 (NH)	10.6 or 10.7
	Hζ1	7.30		Hζ1	7.30		Hζ1	7.30
	Hη2	7.03		Hη2	7.03		Hη2	7.03
	Hζ3	6.94		Hζ3	6.95		Hζ3	6.94
	Hε2	7.55		Hε2	7.56		Hε2	7.55
D-Leu-12	NHα	7.87	D-Leu-12	NHα	7.88	D-Leu-12	NHα	7.85
	Hα	4.17		Hα	4.18		Hα	4.15
	Hβ	1.13		Hβ	1.13–1.19		Hβ	1.15 or 1.22
	Hγ	0.95		Hγ	0.96		Hγ	0.94 or 0.98 or 1.02
	Hδ	0.60 (and/or 0.54)		Hδ	0.61 or 0.53		Hδ	0.60
L-Trp-13	NHα	8.11	L-Trp-13	NHα	8.11	L-Trp-13	NHα	8.11
	Hα	4.56		Hα	4.56		Hα	4.52
	Hβ	2.94, 3.19		Hβ	2.96, 3.18		Hβ	2.94, 3.18
	Hδ	7.09		Hδ	7.10		Hδ	7.10
	Hε1 (NH)	10.71 or 10.67		Hε1 (NH)	10.7		Hε1 (NH)	10.6 or 10.7
	Hζ1	7.30		Hζ1	7.30		Hζ1	7.30
	Hη2	7.03		Hη2	7.03		Hη2	7.03
	Hζ3	6.94		Hζ3	6.95		Hζ3	6.94
	Hε2	7.55		Hε2	7.56		Hε2	7.55
D-Leu-14	NHα	7.96	D-Leu-14	NHα	7.98	D-Leu-14	NHα	7.83 or 7.95
	Hα	4.17		Hα	4.18		Hα	4.15
	Hβ	1.21		Hβ	1.21		Hβ	1.15 or 1.22
	Hγ	1.02		Hγ	1.02		Hγ	0.94 or 0.98 or 1.02
	Hδ	0.59		Hδ	0.65 (and 0.60)		Hδ	0.60
L-Trp-15	NHα	8.19	L-Trp-15	NHα	8.20	L-Trp-15	NHα	8.20
	Hα	4.52		Hα	4.50		Hα	4.48
	Hβ	2.94, 3.22		Hβ	2.96, 3.24		Hβ	2.92, 3.22
	Hδ	7.09		Hδ	7.10		Hδ	7.10
	Hε1 (NH)	10.71 or 10.67		Hε1 (NH)	10.7		Hε1 (NH)	10.6 or 10.7
	Hζ2	7.30		Hζ2	7.30		Hζ2	7.30
	Hη2	7.03		Hη2	7.03		Hη2	7.03
	Hζ3	6.94		Hζ3	6.95		Hζ3	6.94
	Hε2	7.55		Hε2	7.56		Hε2	7.55
2-AE[b]	NH	7.83	2-AE[b]	NH	7.83	2-AE[b]	NH	7.84
	CH_2	3.15		CH_2	3.19		CH_2	3.19
	CH_2	3.43		CH_2	3.44		CH_2	3.43
	OH	ND[c]		OH	ND[c]		OH	ND[c]

[a]The spectra of gramicidin A (**1**, 74.1 mM), **A1** (110 mM), and $\mathbf{B_13}$ (80.1 mM) were obtained in DMSO-d_6 at 40 °C (500 MHz). [b]2-AE = 2-aminoethanol. [c]ND = The chemical shift was not determined.

Supplementary Table 12. ^{1}H NMR chemical shifts of **B₂1** and **B₂2**[a]

residue	position	B_21	residue	position	B_22
formyl	CHO	8.07	formyl	CHO	8.06
L-Val-1	NHα	8.15	L-Val-1	NHα	8.16
	Hα	4.23		Hα	4.21
	Hβ	2.01		Hβ	1.99
	Hγ	0.87		Hγ	0.84
Gly-2	NHα	8.25	Gly-2	NHα	8.24
	Hα	3.73		Hα	3.73
L-Ala-3	NHα	7.97	L-Ala-3	NHα	7.95
	Hα	4.37		Hα	4.24
	Hβ	1.25		Hβ	1.21
D-Thr-4	NHα	7.83	D-Leu-4	NHα	8.06
	Hα	4.15		Hα	4.21
	Hβ	4.00		Hβ	1.46
	Hγ	1.04		Hγ	1.56
	OH	ND[c]		Hδ	0.85
L-Ala-5	NHα	7.80	L-Ala-5	NHα	7.81
	Hα	4.24		Hα	4.21
	Hβ	1.25		Hβ	1.21
D-Leu-6	NHα	7.87	D-Leu-6	NHα	7.99
	Hα	4.35		Hα	4.35
	Hβ	1.47		Hβ	1.48
	Hγ	1.54		Hγ	1.53
	Hδ	0.81		Hδ	0.76
L-Val-7	NHα	7.75	L-Val-7	NH	7.81
	Hα	4.17		Hα	4.12
	Hβ	2.01		Hβ	1.97
	Hγ	0.81		Hγ	0.79
D-Leu-8	NHα	7.97	D-Asm-8	NHα	8.17
	Hα	4.23		Hα	4.58
	Hβ	1.14, 1.24		Hβ	2.36, 2.46
	Hγ	1.24		Hδ (NH)	7.64
	Hδ	0.67		Hε	2.50
L-Trp-9	NHα	8.15	L-Trp-9	NHα	7.95
	Hα	4.59		Hα	4.42
	Hβ	2.94, 3.19		Hβ	2.95, 3.18
	Hδ	7.13		Hδ	7.11
	Hε1 (NH)	10.7		Hε1 (NH)	10.6 or 10.7
	Hζ1	7.30		Hζ1	7.30
	Hη2	7.03		Hη2	7.03
	Hζ3	6.95		Hζ3	6.94
	Hε2	7.57		Hε2	7.55
D-Thr-10	NHα	7.77	D-Leu-10	NHα	7.93
	Hα	4.17		Hα	4.15
	Hβ	3.86		Hβ	1.20 or 1.15
	Hγ	0.74		Hγ	1.02 or 0.95
	OH	ND[c]		Hδ	0.57
L-Trp-11	NHα	7.98	L-Trp-11	NHα	8.00
	Hα	4.56		Hα	4.49
	Hβ	2.96, 3.16		Hβ	2.97, 3.17
	Hδ	7.13		Hδ	7.11
	Hε1 (NH)	10.7		Hε1 (NH)	10.6 or 10.7
	Hζ1	7.30		Hζ1	7.30
	Hη2	7.03		Hη2	7.03
	Hζ3	6.95		Hζ3	6.94
	Hε2	7.57		Hε2	7.55
D-Leu-12	NHα	7.83	D-Leu-12	NHα	7.93
	Hα	4.15		Hα	4.15
	Hβ	1.10		Hβ	1.20 or 1.15
	Hγ	0.95		Hγ	1.02 or 0.95
	Hδ	0.55, 0.60		Hδ	0.57
L-Trp-13	NHα	8.09	L-Trp-13	NHα	8.17
	Hα	4.59		Hα	4.58
	Hβ	2.92, 3.17		Hβ	2.97, 3.19
	Hδ	7.13		Hδ	7.11
	Hε1 (NH)	10.7		Hε1 (NH)	10.6 or 10.7
	Hζ1	7.30		Hζ1	7.30
	Hη2	7.03		Hη2	7.03
	Hζ3	6.95		Hζ3	6.94
	Hε2	7.57		Hε2	7.55
D-Val-14	NHα	7.81	D-Thr-14	NHα	7.84
	Hα	4.09		Hα	4.12
	Hβ	1.81		Hβ	3.86
	Hγ	0.55		Hγ	0.74
				OH	ND[c]
L-Trp-15	NHα	8.19	L-Trp-15	NHα	8.00
	Hα	4.56		Hα	4.49
	Hβ	2.94, 3.19		Hβ	2.97, 3.17
	Hδ	7.13		Hδ	7.11
	Hε1 (NH)	10.7		Hε1 (NH)	10.6 or 10.7
	Hζ2	7.30		Hζ2	7.30
	Hη2	7.03		Hη2	7.03
	Hζ3	6.95		Hζ3	6.94
	Hε2	7.57		Hε2	7.55
2-AE[b]	NH	7.84	2-AE[b]	NH	7.80
	CH_2	3.16		CH_2	3.13
	CH_2	3.42		CH_2	3.36
	OH	ND[c]		OH	ND[c]

[a]The spectra of **B₂1** (68.1 mM) and **B₂2** (26.9 mM) were obtained in DMSO-d_6 at 40 °C (500 MHz). [b]2-AE = 2-aminoethanol. [c]ND = The chemical shift was not determined.

Supplementary Table 13. ^{13}C NMR chemical shifts of gramicidin A, **A1**, **B$_1$3**, **B$_2$1**, and **B$_2$2**[a]

**1**	**A1**	**B$_1$3**	**B$_2$1**	**B$_2$2**
17.5 (2C)	17.47	17.6	17.7	17.7
17.71	17.53	17.7	17.8	17.8
17.75	17.7	17.8	17.85	18.1
18.0	17.8	18.0	17.91	18.8
18.2	17.9	18.2	18.0	19.0 (3C)
18.9	18.4	19.1	18.8	21.09
19.07	18.5	19.2 (3C)	18.9	21.11
19.14	18.95	21.2	19.0	21.3
19.2	19.05	21.5	19.1	21.4
21.2	19.1	21.6	19.4	22.4
21.4	19.16	21.8	21.25	22.5
21.5	19.21	22.4	21.33	22.8
21.7	21.45	22.5 (2C)	21.5	22.9
22.4 (3C)	21.53	22.9	22.5	23.4
22.9	21.8	23.5	22.6	23.5
23.5 (2C)	22.4	23.56	22.8	24.09
23.6	22.5 (2C)	23.61	23.5	24.14
24.2	23.5 (2C)	24.2	23.8	25.4
27.5 (2C)	23.6	27.5 (3C)	24.1	27.2
27.7 (2C)	27.6 (2C)	27.7	27.7 (2C)	27.5 (2C)
30.1	27.8 (2C)	30.2 (2C)	27.8	27.6
30.2	30.2	30.6	27.9	29.9
30.3	30.3 (2C)	40.2	30.0	30.2
30.6	30.4	40.3	30.2	37.5
40.2	30.7	40.41	30.3	40.2
40.4 (2C)	40.3	40.44	40.5 (2C)	40.3 (2C)
40.5	40.4 (2C)	41.7	40.9	40.7
41.7	41.7	41.9	41.6	41.6
41.9	41.9	48.6	41.9	41.8
48.5	48.4	48.7	48.6	48.6
48.6	48.5	51.3	48.7	48.7
51.3	51.5 (2C)	51.6	51.31	50.0
51.5 (2C)	51.7	51.68	51.34	51.16
51.6	53.7	51.72	51.4	51.19
53.7	53.8	53.8	53.5	51.5 (2C)
53.8	53.9	53.9	53.76	53.6
53.85	54.0	54.0 (2C)	53.84	54.0 (2C)
53.95	56.4	56.5	54.1	54.2
56.4	57.6	57.8	56.4	56.4
57.6	57.7	58.0	57.9	58.3
57.7	57.8	58.7	58.3	58.8
58.0	58.1	59.8	58.4	59.6
59.7	59.8	66.5	58.6	66.3
109.5	109.6	109.5	59.7	109.55
109.7 (2C)	109.7 (2C)	109.7 (2C)	66.56	109.65
110.2	110.2	110.2	66.60	109.7
111.1 (4C)	111.1 (4C)	111.1 (4C)	109.6	109.8
118.0 (4C)	118.1 (4C)	118.1 (5C)	109.8	111.0
118.1 (2C)	118.2 (2C)	118.2	109.9	111.06
118.2 (2C)	118.3 (2C)	118.3 (2C)	110.1	111.10 (2C)
120.6 (4C)	120.6 (4C)	120.6 (4C)	111.1 (4C)	118.0 (5C)
123.6 (2C)	123.6 (2C)	123.66	118.0	118.2 (3C)
123.67	123.7	123.73	118.06 (2C)	120.6 (4C)
123.72	123.8	123.8 (2C)	118.09 (2C)	123.6
127.06	127.10	127.07	118.26 (2C)	123.65
127.08	127.13	127.10	118.31	123.71 (2C)
127.12	127.16	127.15	120.6	127.0 (2C)
127.14	127.19	127.18	120.7 (3C)	127.1
136.1 (4C)	136.1 (4C)	136.1 (4C)	123.7 (2C)	127.14
161.2	161.1	161.3	123.76	135.9
168.4	168.3	168.5	123.82	135.97 (2C)
170.7	170.6	170.0	127.1 (4C)	136.01
171.0 (3C)	170.8	171.0	136.0	161.1
171.1	170.97 (2C)	171.11	136.06 (2C)	168.5
171.3	171.02	171.14	136.10	169.6
171.4	171.2	171.2	161.2	169.7
171.5	171.3	171.4	168.5	170.6
171.6	171.4	171.5	169.80	170.7
171.65	171.6	171.6	169.84	170.87
171.72	171.67	171.7 (2C)	170.7	170.94
171.8	171.75	171.8	170.96 (2C)	171.1
172.1	171.8	171.9	171.01	171.4
172.2	172.15	172.2	171.4	171.7
	172.20	172.5	171.6	171.8 (3C)
			171.69	172.16
			171.71	172.24
			171.9	172.3
			172.0 (2C)	
			172.4	

[a]The spectra of gramicidin A (**1**, 74.1 mM), **A1** (110 mM), **B$_1$3** (80.1 mM), **B$_2$1** (68.1 mM), and **B$_2$2** (26.9 mM) were obtained in DMSO-d_6 at 40 °C (125 MHz).

Supplementary Table 14. EC_{50} values of H^+/Na^+ transport activities and IC_{50} values of cytotoxicities

compounds	H^+/Na^+ transport activity/ EC_{50} (nM)[a]	cytotoxicity against P388 cells/ IC_{50} (nM)[a]
gramicidin A (**1**)	4.46 ± 0.48	5.81 ± 0.99
**A1**	2.46 ± 0.90	4.10 ± 0.83
$\mathbf{B_01}$	2.28 ± 0.54	17.5 ± 12.0
$\mathbf{B_02}$	3.38 ± 1.87	16.8 ± 2.82
$\mathbf{B_03}$	1.32 ± 0.40	37.6 ± 1.56
$\mathbf{B_04}$	3.71 ± 0.42	40.6 ± 5.95
$\mathbf{B_11}$	5.65 ± 2.76	250 ± 57.7
$\mathbf{B_12}$	1.83 ± 1.08	387 ± 90.9
$\mathbf{B_13}$	1.55 ± 0.04	>1000
$\mathbf{B_21}$	1.49 ± 0.15	>1000
$\mathbf{B_22}$	2.45 ± 0.50	>1000

[a]The values are displayed as mean ± SD of three independent experiments. Source data are provided as a Source Data file.

Supplementary Table 15. MIC values (μg/mL) against six bacterial strains

compounds	antibacterial activity/MIC (μg/mL)					
	S. pyogenes	*E. faecalis*	*S. pneumoniae*	*S. agalactiae*	*L. monocytogenes*	MSSA1[a]
gramicidin A (**1**)	0.063	0.5	0.016	2	8	32
**A1**	0.015	0.25	0.0019	1	2	8
$\mathbf{B_01}$	0.03	0.5	0.0039	16	8	16
$\mathbf{B_02}$	0.13	0.25	0.016	16	4	32
$\mathbf{B_03}$	0.13	1	0.0078	32	8	32
$\mathbf{B_04}$	0.063	1	0.016	8	8	32
$\mathbf{B_11}$	0.5	2	0.031	16	4	8
$\mathbf{B_12}$	0.13	2	0.016	16	4	4
$\mathbf{B_13}$	1	4	0.063	8	2	8
$\mathbf{B_21}$	32	8	1	64	>64	16
$\mathbf{B_22}$	>64	64	2	>64	>64	>64

[a]MSSA1 = methicillin-susceptible *Staphylococcus aureus.*

Supplementary Methods

General remarks. Unless otherwise stated, all reactions sensitive to air or moisture were carried out under argon (Ar) atmosphere in dry solvents. Purification of CH_2Cl_2, DMF, and Et_2O was performed on a Glass Contour solvent dispensing system (Nikko Hansen). All other reagents were used as supplied. SPPS was performed on a microwave-assisted peptide synthesizer MWS-1000 (EYELA) equipped with a sealed reaction vessel, in which the reaction temperature was monitored by an internal temperature probe, or an automated peptide synthesizer Initiator + Alstra (Biotage). High performance liquid chromatography (HPLC) experiments were performed on an HPLC system equipped with a PU-2089 Plus intelligent pump (JASCO), a PU-2086 Plus intelligent pump (JASCO), a PU-4180 RHPLC pump (JASCO), or a 1100 HPLC system (Agilent). UHPLC experiments were performed with an X-LC system (JASCO) or an Extrema system (JASCO). UV absorbance was measured on a UV-1800 UV-VIS spectrophotometer (Shimadzu). Optical rotations were recorded on a P-2200 polarimeter (JASCO) at ambient temperature using the sodium D line. Infrared spectra were recorded on an FT/IR-4100 spectrometer (JASCO) as a thin film on CaF_2. ^{1}H and ^{13}C NMR spectra were recorded on an ECX 500 (500 MHz for ^{1}H NMR, 125 MHz for ^{13}C NMR) spectrometer (JEOL). Chemical shifts are denoted in ppm on the δ scale relative to residual solvent peaks as an internal standard: CD_2HOD (δ 3.31 for ^{1}H NMR), DMSO-d_5 (δ 2.50 for ^{1}H NMR), DMSO-d_6 (δ 39.5 for ^{13}C NMR). HRMS spectra were recorded on a MicrOTOFII (Bruker Daltonics) electrospray ionization time of flight (TOF) mass spectrometer. Matrix-assisted laser desorption ionization-TOF MS and MS/MS sequencing analyses were performed on a TOF/TOF 5800 system (AB Sciex).

Determination of the loading rate. Fmoc-protected resin was treated with piperidine/DMF (1/1, 1.00 mL) at room temperature for 15 min, and the supernatant was collected. The supernatant (60.0 μL) was diluted with DMF (2.94 mL). UV absorption at 301 nm of the diluted supernatants was measured. The background absorbance was canceled by subtracting the control absorbance obtained from a solution of piperidine/DMF (1/100). The loading rate (x mmol/g) was determined by the following Supplementary Equation 1, where a is the weight of Fmoc-protected resin (mg), and b is absorbance at 301 nm.

$$x = (50000 \times b) / (7800 \times a) \qquad (1)$$

Preloaded resin 14. TentaGel Macrobeads **12** (125 μmol, 0.25 mmol/g, 65550 beads/g) in 5 mL LibraTube (Hipep Laboratories) were washed with CH_2Cl_2 (2.00 mL × 3) and DMF (2.00 mL × 3).

To a solution of 4-(hydroxymethyl)benzoic acid (HMBA, **13**, 57.1 mg, 375 μmol) and 1-hydroxybenzotriazole (50.7 mg, 375 μmol) in DMF (2.00 mL) was added *N,N'*-diisopropylcarbodiimide (57.7 μL, 375 μmol) at 0 °C. The reaction mixture was stirred at 0 °C for 5 min and at room temperature for 5 min. The resultant mixture was transferred to the beads in the 5 mL LibraTube. After being stirred at room temperature for 2.5 h, the reaction mixture was filtered, and washed with DMF (2.00 mL, 30 sec × 6) to give HMBA-TentaGel Macrobeads.

To a solution of Fmoc-L-Trp(Boc)-OH (**21**, 198 mg, 375 μmol) in DMF (2.00 mL) were added *N,N'*-diisopropylcarbodiimide (57.7 μL, 375 μmol) and 4-(*N,N*-dimethylamino)pyridine (2.30 mg, 18.9 μmol) at room temperature. The reaction mixture was stirred at room temperature for 1 min. The resultant mixture was added to the above HMBA-TentaGel Macrobeads in the 5 mL LibraTube at room temperature. After being stirred at room temperature for 12 h, the reaction mixture was filtered, and washed with DMF (2.00 mL, 30 sec × 3), MeOH (2.00 mL, 30 sec × 3), and Et_2O (2.00 mL, 30 sec × 3), and dried under vacuum. The same procedure was repeated (× 2). The reaction mixture was filtered, washed with DMF (2.00 mL, 30 sec × 3), MeOH (2.00 mL, 30 sec × 3), and Et_2O (2.00 mL, 30 sec × 3), and dried under vacuum to give the preloaded resin **14** (575 mg). The loading rate was determined to be 0.234 mmol/g (94% over 2 steps) by the above method.

Procedures for microwave-assisted solid-phase peptide synthesis (SPPS). Peptide **1** was prepared on a peptide synthesizer MWS-1000A. Standard operation was shown as follows:

Step 1: The solid supported N_α-Fmoc peptide was deprotected with piperidine/NMP (1/4, 40 °C, 200 W; 5 min).

Step 2: The 5 mL LibraTube containing the resin was washed with NMP (2.00 mL, 30 sec × 6).

Step 3: N_α-Fmoc-amino acid (4.0 eq) was activated by a solution of *O*-(7-aza-1*H*-benzotriazol-1-yl)-*N,N,N'*,*N'*-tetramethyluronium hexafluorophosphate (HATU)/1-hydroxy-7-azabenzotriazole (HOAt) (4.0 eq, 0.45 M) in NMP. To the solution of activated Fmoc-amino acid was added a solution of *i*-Pr_2NEt (8.0 eq, 2.0 M) in NMP. The resultant mixture was transferred to the 5 mL LibraTube.

Step 4: The activated N_α-Fmoc-amino acid was coupled with the peptide on the resin (40 °C, 200 W; 20 min) and the 5 mL LibraTube containing the resin was washed with NMP (2.00 mL, 30 sec × 6).

Steps 1−4 were repeated and amino acids were condensed on the solid support.

Synthesis of gramicidin A. To preloaded resin **14** (103 mg, 0.203 mmol/g, 20.9 μmol) was added Ac_2O/CH_2Cl_2 (1/3, 1.00 mL) at room temperature for capping the remaining hydroxy groups. After being stirred at room temperature for 25 min, the reaction mixture was filtered, washed with CH_2Cl_2 (2.00 mL, 30 sec × 3), MeOH (2.00 mL, 30 sec × 3), and Et_2O (2.00 mL, 30 sec × 3), and dried under vacuum to give resin for microwave-assisted SPPS.

The above resin in 5 mL LibraTube was washed with CH_2Cl_2 (2.00 mL, 30 sec × 6) and NMP (2.00 mL, 30 sec × 6). The resin was subjected to 14 cycles (N_α-Fmoc-amino acids: **2**–**5**, **8**, and **9**) of the microwave-assisted SPPS protocol to give bead-linked peptide **15**.

The N_α-Fmoc group of the above bead-linked peptide **15** was removed by steps 1 and 2 of the standard SPPS protocol. The resin in the 5 mL LibraTube was washed with CH_2Cl_2 (2.00 mL, 30 sec × 6), MeOH (2.00 mL, 30 sec × 6), and Et_2O (2.00 mL, 30 sec × 6), and dried under vacuum to give H-[1–15 $(Boc)_4$]-HMBA-TentaGel Macrobeads (163 mg).

To the above H-[1–15 $(Boc)_4$]-HMBA-TentaGel Macrobeads (163 mg) in the 5 mL LibraTube were added *p*-nitrophenyl formate (**6**, 18.4 mg, 110 μmol) and *N*-methylmorpholine/DMF (0.4/99.6, 1.91 mL) at room temperature. After being stirred at room temperature for 16 h, the reaction mixture was filtered, washed with DMF (2.00 mL, 30 sec × 6), NMP (2.00 mL, 30 sec × 3), CH_2Cl_2 (2.00 mL, 30 sec × 3), MeOH (2.00 mL, 30 sec × 3), and Et_2O (2.00 mL, 30 sec × 3), and dried under vacuum for 6 h to give bead-linked peptide **16** (151 mg).

The above bead-linked peptide **16** (37.7 mg) in the 5 mL LibraTube was washed with CH_2Cl_2 (2.00 mL, 30 sec × 3) and DMF (2.00 mL, 30 sec × 3). To the resin in the 5 mL LibraTube was added TFA/H_2O (95/5, 2.00 mL) at room temperature. After being stirred at room temperature for 1 h, the reaction mixture was filtered, and washed with DMF (2.00 mL, 30 sec × 3) to give bead-linked peptide **17**.

The one bead of **17** was transferred to a microtube with DMF (10.0 μL), and then DMF was removed under vacuum. To the dried one bead in the microtube was added 2-aminoethanol (**7**)/DMF (1/9, 10.0 μL). The reaction mixture was incubated at 50 °C for 24 h. The supernatant was collected. The resultant bead was washed with DMF (10.0 μL × 3), and the supernatants were collected. The same procedure was repeated (× 2). The combined supernatants were dried under vacuum to give the crude **1**. The crude **1** was dissolved in MeOH/H_2O (4/1, 50.0 μL), and filtered through 0.20 μm PTFE filter. The filtrate was analyzed by reversed-phase UHPLC (column: Accucore C18, 2.1 × 150 mm, eluent A: MeOH + 0.05% TFA, eluent B: H_2O + 0.05% TFA, A/B = 80/20, flow rate: 0.2 mL/min, detection: photodiode array detector 200−650 nm (UV chromatogram: 280 nm), temperature: 40 ºC, t_R = 5.3 min). The peak area of **1** (UV 280 nm) was calculated using ChromNAV (JASCO) to compare with the peak area of purified **1** as a reference compound. The overall yield of **1** from preloaded resin **14** was determined as 44.6 ± 1.6% (mean value of three beads ± SD) over 32 steps (Supplementary Figure 3).

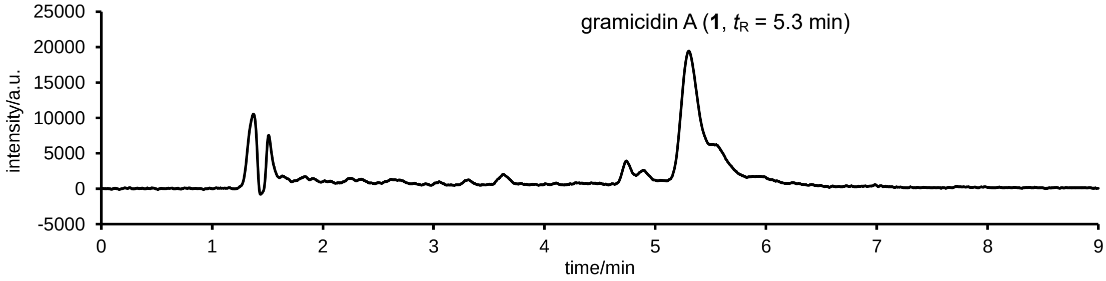

Supplementary Figure 3. UHPLC chart of bead-derived gramicidin A. Column: Accucore C18 2.1 × 150 mm, eluent A: MeOH + 0.05% TFA, eluent B: H_2O + 0.05% TFA, A/B = 80/20, flow rate: 0.20 mL/min, detection: photodiode array detector 200−650 nm (UV chromatogram: 280 nm), temperature: 40 ºC. a.u. = arbitrary unit.

Procedures for split-and-mix synthesis. Bead-linked 4096 peptides were prepared on a peptide synthesizer MWS-1000A. Standard operation was shown as follows:

Step 1: The solid supported N_α-Fmoc peptide suspended in CH_2Cl_2 was split into four 5 mL LibraTubes. The solid supported N_α-Fmoc peptide was washed with MeOH (2.00 mL, 30 sec × 6) and Et_2O (2.00 mL, 30 sec × 6), and dried under vacuum at room temperature for 1 h. The amount of the solid supported N_α-Fmoc peptide in each tube was precisely adjusted based on the weight. The solid supported N_α-Fmoc peptide was washed with CH_2Cl_2 (2.00 mL, 30 sec × 6) and NMP (2.00 mL, 30 sec × 6).

Step 2: The solid supported N_α-Fmoc peptide was deprotected with piperidine/NMP (1/4, 60 °C, 200 W; 5 min).

Step 3: The resin in the 5 mL LibraTube was washed with NMP (2.00 mL, 30 sec × 6).

Step 4: N_α-Fmoc-amino acid (4.0 eq) in a vial was activated by a solution of *O*-(7-aza-1*H*-benzotriazol-1-yl)-*N*,*N*,*N'*,*N'*-tetramethyluronium hexafluorophosphate (HATU, 4.0 eq, 0.45 M)/1-hydroxy-7-azabenzotriazole (HOAt, 4.0 eq, 0.45 M) in NMP. To the solution of activated N_α-Fmoc-amino acid was added a solution of *i*-Pr_2NEt (8.0 eq, 2.0 M) in NMP. The resultant mixture was transferred to the 5 mL LibraTube.

Step 5: The activated N_α-Fmoc-amino acid was coupled with the peptide on the resin (60 °C, 200 W; 20 min) and the 5 mL LibraTube containing the resin was washed with NMP (2.00 mL, 30 sec × 6).

Step 6: The four resin batches suspended in CH_2Cl_2 were mixed in one LibraTube.

Steps 1−6 were conducted for the condensation of the residues-4, -6, -8, -10, -12, and -14.

Steps 2−5 were conducted for the condensation of the residues-1, -2, -3, -5, -7, -9, -11, and -13.

Preparation of gramicidin A-based library. To the above preloaded resin **14** (217 mg, 0.234 mmol/g, 50.9 μmol) was added Ac_2O/CH_2Cl_2 (1/3, 1.00 mL) at room temperature for capping the remaining hydroxy groups. After being stirred at room temperature for 25 min, the reaction mixture was filtered, washed with CH_2Cl_2 (2.00 mL, 30 sec × 3), MeOH (2.00 mL, 30 sec × 3), and Et_2O (2.00 mL, 30 sec × 3), and dried under vacuum to give the beads for split-and-mix synthesis.

The above beads were subjected to 14 cycles (N_α-Fmoc-amino acids: **2**–**5**, **8**, **9**, **10**, and **11**) of microwave-assisted SPPS to give bead-linked pentadecapeptide.

The N_α-Fmoc group of the above bead-linked pentadecapeptide was removed by steps 2 and 3 of the standard split-and-mix synthesis protocol. The beads in the 5 mL LibraTube was washed with CH_2Cl_2 (2.00 mL, 30 sec × 6), MeOH (2.00 mL, 30 sec × 6), and Et_2O (2.00 mL, 30 sec × 6), and dried under vacuum to give the bead-linked amine (306 mg).

To the above bead-linked amine (306 mg) were added *p*-nitrophenyl formate (**6**, 18.4 mg, 110 μmol) and *N*-methylmorpholine/DMF (0.4/99.6, 1.91 mL) at room temperature. After being stirred at room temperature for 16 h, the reaction mixture was filtered, washed with DMF (2.00 mL, 30 sec × 6), NMP (2.00 mL, 30 sec × 3), CH_2Cl_2 (2.00 mL, 30 sec × 3), MeOH (2.00 mL, 30 sec × 3), and Et_2O (2.00 mL, 30 sec × 3), and dried under vacuum to give bead-linked peptide **18** (296 mg).

The above bead-linked peptide **18** (296 mg) in the 5 mL LibraTube was washed with CH_2Cl_2 (2.00 mL, 30 sec × 3) and DMF (2.00 mL, 30 sec × 3). To the bead-linked peptide in the 5 mL LibraTube was added TFA/*i*-Pr_3SiH/H_2O (95/2.5/2.5, 2.00 mL) at room temperature. After being stirred at room temperature for 60 min, the reaction mixture was filtered, and washed with DMF (2.00 mL, 30 sec × 3). To the resultant beads was added TFA/*i*-Pr_3SiH/H_2O (95/2.5/2.5, 2.00 mL) at room temperature. After being stirred at 30 °C for 90 min, the reaction mixture was filtered, washed with DMF (2.00 mL, 30 sec × 3), MeOH (2.00 mL, 30 sec × 3), and Et_2O (2.00 mL, 30 sec × 3), and dried under vacuum to give bead-linked peptide **19** (265 mg).

Each bead of **19** was transferred to each well of 96-well PCR plate (652270, Greiner bio-one, 1 bead/well) with DMF (10.0 μL/well) using a micropipette. The solution was removed under vacuum to give the dried beads of **19**. To the dried beads of **19** in the plate was added 2-aminoethanol (**7**)/DMF (1/9, 10.0 μL/well) at room temperature. The reaction mixture was incubated at 50 °C for 24 h. The resultant mixture was concentrated under vacuum at room temperature. To the residue in the plate was added DMF (10.0 μL/well) at room temperature, and then the residual **7** was azeotropically removed with DMF under vacuum at room temperature for 1 d. To the residue in the plate was added DMSO (100 μL/well), which was used in the assays without further purification.

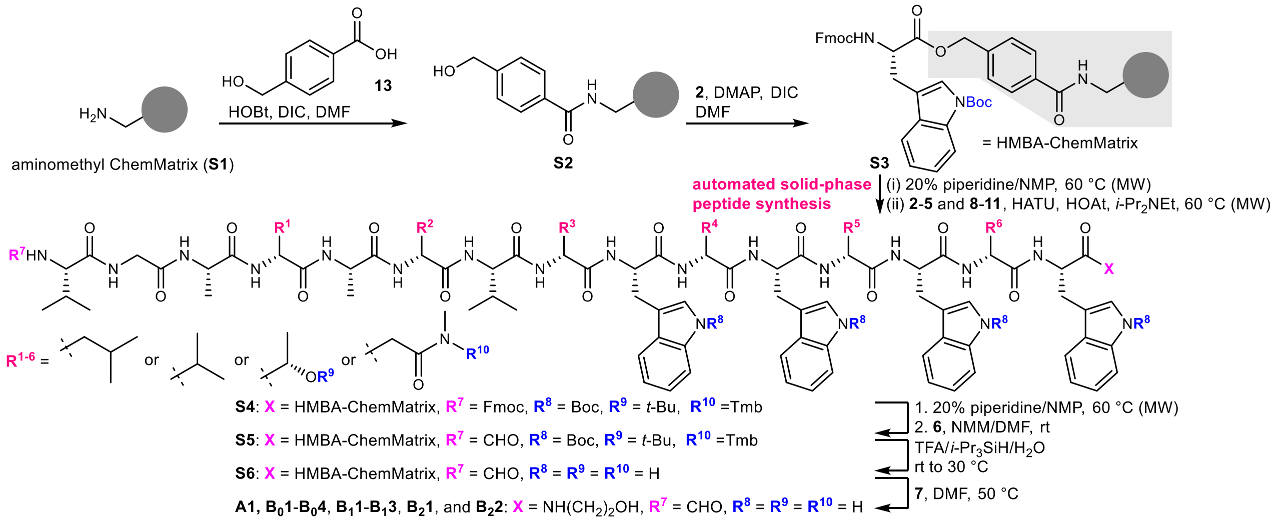

Supplementary Figure 4. Syntheses of peptides using ChemMatrix resin.

Fmoc-L-Trp(Boc)-HMBA-ChemMatrix resin S3. Aminomethyl ChemMatrix resin (**S1**, Supplementary Figure 4, loading rate: 1.00 mmol/g, 1.10 g) in the 20 mL LibraTube was washed with CH_2Cl_2 (5.00 mL, 30 sec × 3) and DMF (5.00 mL, 30 sec × 3).

To a solution of 4-(hydroxymethyl)benzoic acid (HMBA, **13**, 503 mg, 3.30 mmol) and 1-hydroxybenzotriazole (446 mg, 3.30 mmol) in DMF (15.0 mL) was added *N,N′*-diisopropylcarbodiimide (507 μL, 3.30 mmol) at 0 °C. The reaction mixture was stirred at 0 °C for 5 min and at room temperature for 5 min. The resultant mixture was transferred to the above resin in the 20 mL LibraTube. After being stirred at room temperature for 2.5 h, the reaction mixture was filtered, and washed with DMF (5.00 mL, 30 sec × 6) to give HMBA-ChemMatrix resin **S2**.

To a solution of Fmoc-L-Trp(Boc)-OH (**2**, 1.74 g, 3.30 mmol) in DMF (15.0 mL) were added *N,N′*-diisopropylcarbodiimide (507 μL, 3.30 mmol) and 4-(*N*,*N*-dimethylamino)pyridine (20.1 mg, 165 μmol) at room temperature. The reaction mixture was stirred at room temperature for 1 min. The resultant mixture was added to the above HMBA-ChemMatrix resin **S2** in the 20 mL LibraTube at room temperature. After being stirred at room temperature for 12 h, the reaction mixture was filtered, and washed with DMF (5.00 mL, 30 sec × 3), MeOH (5.00 mL, 30 sec × 3), and Et_2O (5.00 mL, 30 sec ×3), and dried under vacuum to give the preloaded resin.

The above preloaded resin (*a* μmol) in the reaction vessel [20 mL LibraTube (for *a* =250 μmol of the preloaded resin) or 5 mL LibraTube (for *a* = 45.0 or 30.0 μmol of the preloaded resin)] was washed with CH_2Cl_2 [5.00 mL (for *a* = 250 μmol of the preloaded resin) or 1.00 mL (for *a* = 45.0 or 30.0 μmol of the preloaded resin)]. To the resin was added Ac_2O/NMP (1/3, 1.00 mL) at room temperature. After being stirred at room temperature for 20 min, the reaction mixture was filtered, and washed with NMP [5.00 mL (for *a* = 250 μmol of the preloaded resin) or 1.00 mL (for *a* = 45.0 or 30.0 μmol of the preloaded resin)] to give resin **S3**, which was used for solid-phase peptide synthesis.

Procedure for automated solid-phase peptide synthesis (automated SPPS). Peptides **A1**, **$B_0$1**–**$B_0$4**, **$B_1$1**–**$B_1$3**, **$B_2$1**, and **$B_2$2** were prepared on an automated peptide synthesizer Initiator + Alstra. Standard operation was shown as follows:

Step 1: The solid supported N_α-Fmoc peptide was deprotected with piperidine/NMP (1/4, 60 °C, 5 min).

Step 2: The resin in a reaction vessel [30 mL filter tube (for 250 μmol of **S3**) or 5 mL filter tube (for 45.0 or 30.0 μmol of **S3**)] was washed with NMP [7.50 mL, 4 min × 6 (for 250 μmol of **S3**) or 2.60 mL, 60 sec × 4 (for 45.0 or 30.0 μmol of **S3**)].

Step 3: A solution of N_α-Fmoc-amino acid (4.0 eq, 0.50 M) in NMP was activated by *O*-(7-azabenzotriazole-1-yl)-*N*,*N*,*N′*,*N′*-tetramethyluronium hexafluorophosphate (HATU, 4.0 eq, 0.45 M)/1-hydroxy-7-azabenzotriazole (HOAt, 4.0 eq, 0.45 M) in NMP. To the solution of activated N_α-Fmoc-amino acid was added *i*-Pr_2NEt (8.0 eq). The resultant mixture was transferred to the reaction vessel.

Step 4: The activated N_α-Fmoc-amino acid was coupled with the peptide on the resin (60 °C, 20 min) and the reaction vessel containing the resin was washed with NMP [7.50 mL, 4 min × 6 (for 250 μmol of **S3**) or 1.50 mL, 60 sec × 4 (for 45.0 or 30.0 μmol of **S3**)].

Steps 1−4 were carried out under a stream of N_2.

Syntheses of gramicidin A analogues. Preloaded resin Fmoc-L-Trp(Boc)-HMBA-ChemMatrix resin (**S3**, *a* μmol) in the reaction vessel [30 mL filter tube (for 250 μmol of **S3**) or 5 mL filter tube (for 45.0 or 30.0 μmol of **S3**)] was washed with CH_2Cl_2 [5.00 mL (for *a* = 250 μmol of **S3**) or 2.00 mL (for *a* = 45.0 or 30.0 μmol of **S3**)] and NMP [5.00 mL (for *a* = 250 μmol of **S3**) or 2.00 mL (for *a* = 45.0 or 30.0 μmol of **S3**)]. The resin was subjected to 14 cycles (N_α-Fmoc-amino acids: **X1** as residue-14, **2** as residue-13, **X2** as residue-12, **2** as residue-11, **X3** as residue-10, **2** as residue-9, **X4** as residue-8, **3** as residue-7, **X5** as residue-6, **4** as residue-5, **X6** as residue-4, **4** as residue-3, **5** as residue-2, and **3** as residue-1) of the automated SPPS protocol to give resin-bound peptide **S4**, where each **X1**−**X6** corresponds to one of **8**, **9**, **10**, and **11**.

The N_α-Fmoc group of the above resin-bound peptide **S4** was removed by steps 1 and 2 of the standard automated SPPS protocol to give the resin-bound amine.

To the above resin-bound amine in the reaction vessel [20 mL LibraTube (for *a* = 250 μmol of **S3**) or 5 mL LibraTube (for *a* = 45.0 or 30.0 μmol of **S3**)] were added *p*-nitrophenyl formate (**6**, 5.0 eq) and *N*-methylmorpholine/DMF [(0.4/99.6, 7.00 mL (for *a* = 250 μmol of **S3**) or 2.50 mL (for *a* = 45.0 or 30.0 μmol of **S3**)] at room temperature. After being stirred at room temperature for 16 h, the reaction mixture was filtered, washed with DMF [5.00 mL, 30 sec × 3 (for *a* = 250 μmol of **S3**) or 2.00 mL, 30 sec × 3 (for *a* = 45.0 or 30.0 μmol of **S3**)], NMP [5.00 mL, 30 sec × 3 (for *a* = 250 μmol of **S3**) or 2.00 mL, 30 sec × 3 (for *a* = 45.0 or 30.0 μmol of **S3**)], CH_2Cl_2 [5.00 mL, 30 sec × 3 (for *a* = 250 μmol of **S3**) or 2.00 mL, 30 sec × 3 (for *a* = 45.0 or 30.0 μmol of **S3**)], MeOH [5.00 mL, 30 sec × 3 (for *a* = 250 μmol of **S3**) or 2.00 mL, 30 sec × 3 (for *a* = 45.0 or 30.0 μmol of **S3**)], and Et_2O [5.00 mL, 30 sec × 3 (for *a* = 250 μmol of **S3**) or 2.00 mL, 30 sec × 3 (for *a* = 45.0 or 30.0 μmol of **S3**)], and dried under vacuum to give resin-bound peptide **S5**.

The above resin-bound **S5** was washed with CH_2Cl_2 [5.00 mL, 30 sec × 3 (for *a* = 125 μmol of **S3**) or 2.00 mL, 30 sec × 3 (for *a* = 15.0 μmol of **S3**)] and DMF [5.00 mL, 30 sec × 3 (for *a* = 125 μmol of **S3**) or 2.00 mL, 30 sec × 3 (for *a* = 15.0 μmol of **S3**)]. To the resin was added TFA/*i*-Pr_3SiH/H_2O [95/2.5/2.5, 8.00 mL (for *a* = 125 μmol of **S3**) or 2.00 mL (for *a* = 15.0 μmol of **S3**)] at room temperature. After being stirred at room temperature for 60 min, the reaction mixture was filtered, and washed with DMF [5.00 mL, 30 sec × 3 (for *a* = 125 μmol of **S3**) or 2.00 mL, 30 sec × 3 (for *a* = 15.0 μmol of **S3**)]. To the resultant resin was added TFA/*i*-Pr_3SiH/H_2O [95/2.5/2.5, 8.00 mL (for *a* = 125 μmol of **S3**) or 2.00 mL (for *a* = 15.0 μmol of **S3**)] at room temperature. After being stirred at 30 °C for 90 min, the reaction mixture was filtered, and washed with DMF [5.00 mL, 30 sec × 3 (for *a* = 125 μmol of **S3**) or 2.00 mL, 30 sec × 3 (for *a* = 15.0 μmol of **S3**)] to give resin-bound peptide **S6**.

To the resin-bound peptide **S6** was added 2-aminoethanol (**7**)/DMF [1/9, 1.50 mL (for *a* = 125 μmol of **S3**) or 0.725 mL (for *a* = 15.0 μmol of **S3**)] at room temperature. The reaction mixture was incubated at 50 °C for 24 h. The resultant mixture was filtered, and washed with DMF [1.00 mL, 30 sec × 5 (for *a* = 125 μmol

of **S3**) or 0.200 mL, 30 sec × 5 (for a = 15.0 μmol of **S3**)]. The filtrate was collected. The same procedure was repeated (× 6). The combined filtrates were concentrated. To the residue was added MeOH at room temperature. The insoluble solid was removed by filtration with 0.20 μm PTFE filter. The filtrate was purified by HPLC to give analogues **A1**, **$B_0$1–$B_0$4**, **$B_1$1–$B_1$3**, **$B_2$1**, and **$B_2$2**.

Peptide A1. Fmoc-L-Trp(Boc)-HMBA-ChemMatrix (**S3**, loading rate: 0.399 mmol/g) was subjected to the general procedures described above using **8** as **X1–X3** and **9** as **X4–X6**. The crude **A1** was purified by reversed-phase HPLC (column: Inertsil C8-3 20 × 250 mm, eluent A: MeOH + 0.05% TFA, eluent B: H_2O + 0.05% TFA, linear gradient A/B = 60/40 to 100/0 over 40 min, flow rate: 5.0 mL/min, detection: UV 280 nm) to give **A1** (103 mg, 55.1 μmol, 44% over 32 steps): white solid; $[\alpha]_D^{28}$ +15.4° (c 0.486, MeOH); IR (film) 1010, 1069, 1097, 1152, 1231, 1283, 1339, 1369, 1389, 1458, 1539, 1636, 2872, 2933, 2961, 3061, 3285 cm^{-1}; ^1H NMR (500 MHz, DMSO-d_6), see Supplementary Table 11; ^{13}C NMR (125 MHz, DMSO-d_6), see Supplementary Table 13; HRMS (ESI-TOF) calcd for $C_{98}H_{138}N_{20}O_{17}Na$ $[M+Na]^+$ 1890.0441, found 1890.0427.

Peptide $B_0$1. Fmoc-L-Trp(Boc)-HMBA-ChemMatrix (**S3**, loading rate: 0.420 mmol/g) was subjected to the general procedures described above using **9** as **X1**, **X2**, and **X4–X6** and **8** as **X3**. The crude **$B_0$1** was purified by reversed-phase HPLC (column: Inertsil C8-3 20 × 250 mm, eluent A: MeOH + 0.05% TFA, eluent B: H_2O + 0.05% TFA, linear gradient A/B = 60/40 to 100/0 over 40 min, flow rate: 5.0 mL/min, detection: UV 280 nm) to give **$B_0$1** (5.36 mg, 2.91 μmol, 19% over 32 steps): white solid; HRMS (ESI-TOF) calcd for $C_{96}H_{134}N_{20}O_{17}Na$ $[M+Na]^+$ 1862.0128, found 1862.0133.

Peptide $B_0$2. Fmoc-L-Trp(Boc)-HMBA-ChemMatrix (**S3**, loading rate: 0.420 mmol/g) was subjected to the general procedures described above using **8** as **X1**, **X2**, and **X5** and **9** as **X3**, **X4**, and **X6**. The crude **$B_0$2** was purified by reversed-phase HPLC (column: Inertsil C8-3 20 × 250 mm, eluent A: MeOH + 0.05% TFA, eluent B: H_2O + 0.05% TFA, linear gradient A/B = 60/40 to 100/0 over 40 min, flow rate: 5.0 mL/min, detection: UV 280 nm) to give **$B_0$2** (7.03 mg, 3.76 μmol, 25% over 32 steps): white solid; HRMS (ESI-TOF) calcd for $C_{98}H_{138}N_{20}O_{17}Na$ $[M+Na]^+$ 1890.0441, found 1890.0455.

Peptide $B_0$3. Fmoc-L-Trp(Boc)-HMBA-ChemMatrix (**S3**, loading rate: 0.420 mmol/g) was subjected to the general procedures described above using **9** as **X1–X5** and **8** as **X6**. The crude **$B_0$3** was purified by reversed-phase HPLC (column: Inertsil C8-3 20 × 250 mm, eluent A: MeOH + 0.05% TFA, eluent B: H_2O + 0.05% TFA, linear gradient A/B = 60/40 to 100/0 over 40 min, flow rate: 5.0 mL/min, detection: UV 280 nm) to give **$B_0$3** (6.07 mg, 3.30 μmol, 22% over 32 steps): white solid; HRMS (ESI-TOF) calcd for $C_{96}H_{134}N_{20}O_{17}Na$ $[M+Na]^+$ 1862.0128, found 1862.0127.

Peptide $B_0$4. Fmoc-L-Trp(Boc)-HMBA-ChemMatrix (**S3**, loading rate: 0.312 mmol/g) was subjected to the general procedures described above using **9** as **X1** and **X3–X5** and **8** as **X2** and **X6**. The crude **$B_0$4** was

purified by 1st reversed-phase HPLC (column: Inertsil C8-3 20 × 250 mm, eluent A: MeOH + 0.05% TFA, eluent B: H_2O + 0.05% TFA, linear gradient A/B = 60/40 to 100/0 over 40 min, flow rate: 5.0 mL/min, detection: UV 280 nm), 2nd HILIC HPLC [column: TSKgel Amide-80 21.5 × 300 mm, eluent A: MeCN + 0.05% TFA, eluent B: H_2O + 0.05% TFA, linear gradient A/B = 95/5 to 80/20 over 40 min, flow rate: 4.5 mL/min, detection: photodiode array detector 200–650 nm (UV chromatogram: 280 nm)], and 3rd reversed-phase HPLC (column: Inertsil C8-3 20 × 250 mm, eluent A: MeOH + 0.05% TFA, eluent B: H_2O + 0.05% TFA, linear gradient A/B = 60/40 to 100/0 over 40 min, flow rate: 5.0 mL/min, detection: UV 280 nm) to give **$B_0$4** (14.0 mg, 7.55 μmol, 50% over 32 steps): white solid; HRMS (ESI-TOF) calcd for $C_{97}H_{136}N_{20}O_{17}Na$ $[M+Na]^+$ 1876.0284, found 1876.0280.

Peptide $B_1$1. Fmoc-L-Trp(Boc)-HMBA-ChemMatrix (**S3**, loading rate: 0.420 mmol/g) was subjected to the general procedures described above using **9** as **X1**, **11** as **X2**, and **8** as **X3**–**X6**. The crude **$B_1$1** was purified by reversed-phase HPLC (column: Inertsil C8-3 20 × 250 mm, eluent A: MeOH + 0.05% TFA, eluent B: H_2O + 0.05% TFA, linear gradient A/B = 60/40 to 100/0 over 40 min, flow rate: 5.0 mL/min, detection: UV 280 nm) to give **$B_1$1** (3.70 mg, 1.96 μmol, 13% over 32 steps): white solid; HRMS (ESI-TOF) calcd for $C_{98}H_{138}N_{20}O_{18}Na$ $[M+Na]^+$ 1906.0390, found 1906.0399.

Peptide $B_1$2. Fmoc-L-Trp(Boc)-HMBA-ChemMatrix (**S3**, loading rate: 0.420 mmol/g) was subjected to the general procedures described above using **9** as **X1** and **X5**, **8** as **X2**, **X3**, and **X6**, and **11** as **X4**. The crude **$B_1$2** was purified by 1st reversed-phase HPLC (column: Inertsil C8-3 20 × 250 mm, eluent A: MeOH + 0.05% TFA, eluent B: H_2O + 0.05% TFA, linear gradient A/B = 60/40 to 100/0 over 40 min, flow rate: 5.0 mL/min, detection: UV 280 nm), 2nd HILIC HPLC [column: TSKgel Amide-80 21.5 × 300 mm, eluent A: MeCN + 0.05% TFA, eluent B: H_2O + 0.05% TFA, linear gradient A/B = 95/5 to 80/20 over 40 min, flow rate: 4.5 mL/min, detection: photodiode array detector 200–650 nm (UV chromatogram: 280 nm)], and 3rd reversed-phase HPLC (column: Inertsil C8-3 20 × 250 mm, eluent A: MeOH + 0.05% TFA, eluent B: H_2O + 0.05% TFA, linear gradient A/B = 60/40 to 100/0 over 40 min, flow rate: 5.0 mL/min, detection: UV 280 nm) to give **$B_1$2** (4.64 mg, 2.48 μmol, 17% over 32 steps): white solid; HRMS (ESI-TOF) calcd for $C_{97}H_{136}N_{20}O_{18}Na$ $[M+Na]^+$ 1892.0233, found 1892.0231.

Peptide $B_1$3. Fmoc-L-Trp(Boc)-HMBA-ChemMatrix (**S3**, loading rate: 0.388 mmol/g) was subjected to the general procedures described above using **8** as **X1**–**X3** and **X6**, **11** as **X4**, and **9** as **X5**. The crude **$B_1$3** was purified by 1st reversed-phase HPLC (column: Inertsil C8-3 20 × 250 mm, eluent A: MeOH + 0.05% TFA, eluent B: H_2O + 0.05% TFA, linear gradient A/B = 60/40 to 100/0 over 40 min, flow rate: 5.0 mL/min, detection: UV 280 nm), 2nd HILIC HPLC [column: TSKgel Amide-80 21.5 × 300 mm, eluent A: MeCN + 0.05% TFA, eluent B: H_2O + 0.05% TFA, linear gradient A/B = 95/5 to 80/20 over 40 min, flow rate: 4.5 mL/min, detection: photodiode array detector 200–650 nm (UV chromatogram: 280 nm)], and 3rd reversed-phase HPLC (column: Inertsil C8-3 20 × 250 mm, eluent A: MeOH + 0.05% TFA, eluent B: H_2O + 0.05% TFA, linear gradient A/B = 65/35 to 100/0 over 80 min, flow rate: 5.0 mL/min, detection: UV 280 nm)

to give **B$_1$3** (90.5 mg, 48.0 μmol, 38% over 32 steps): white solid; $[\alpha]_D^{28}$ +33.2° (*c* 0.494, MeOH); IR (film) 1024, 1143, 1211, 1388, 1419, 1457, 1472, 1508, 1523, 1541, 1652, 2872, 2960, 3059, 3301 cm^{-1}; ^{1}H NMR (500 MHz, DMSO-d_6), see Supplementary Table 11; ^{13}C NMR (125 MHz, DMSO-d_6), see Supplementary Table 13; HRMS (ESI-TOF) calcd for $C_{98}H_{138}N_{20}O_{18}Na$ [M+Na]$^+$ 1906.0390, found 1906.0395.

Peptide B$_2$1. Fmoc-L-Trp(Boc)-HMBA-ChemMatrix (**S3**, loading rate: 0.406 mmol/g) was subjected to the general procedures described above using **9** as **X1**, **8** as **X2**, **X4**, and **X5**, and **11** as **X3** and **X6**. The crude **B$_2$1** was purified by 1st reversed-phase HPLC (column: Inertsil C8-3 20 × 250 mm, eluent A: MeOH + 0.05% TFA, eluent B: H_2O + 0.05% TFA, linear gradient A/B = 60/40 to 100/0 over 40 min, flow rate: 5.0 mL/min, detection: UV 280 nm) and 2nd reversed-phase HPLC (column: Inertsil C8-3 20 × 250 mm, eluent A: MeOH + 0.05% TFA, eluent B: H_2O + 0.05% TFA, linear gradient A/B = 65/35 to 100/0 over 80 min, flow rate: 5.0 mL/min, detection: UV 280 nm) to give **B$_2$1** (70.5 mg, 37.6 μmol, 30% over 32 steps): white solid; $[\alpha]_D^{28}$ +11.1° (*c* 0.493, MeOH); IR (film) 1013, 1105, 1148, 1230, 1340, 1387, 1457, 1539, 1650, 2871, 2931, 2958, 3059, 3284 cm^{-1}; ^{1}H NMR (500 MHz, DMSO-d_6), see Supplementary Table 12; ^{13}C NMR (125 MHz, DMSO-d_6), see Supplementary Table 13; HRMS (ESI-TOF) calcd for $C_{96}H_{134}N_{20}O_{19}Na$ [M+Na]$^+$ 1894.0026, found 1894.0018.

Peptide B$_2$2. Fmoc-L-Trp(Boc)-HMBA-ChemMatrix (**S3**, loading rate: 0.312 mmol/g) was subjected to the general procedures described above using **11** as **X1**, **8** as **X2**, **X3**, **X5**, and **X6**, and **10** as **X4**. The crude **B$_2$2** was purified by 1st reversed-phase HPLC (column: Inertsil C8-3 20 × 250 mm, eluent A: MeOH + 0.05% TFA, eluent B: H_2O + 0.05% TFA, linear gradient A/B = 60/40 to 100/0 over 40 min, flow rate: 5.0 mL/min, detection: UV 280 nm) and 2nd reversed-phase HPLC (column: Inertsil C8-3 20 × 250 mm, eluent A: MeOH + 0.05% TFA, eluent B: H_2O + 0.05% TFA, linear gradient A/B = 65/35 to 100/0 over 80 min, flow rate: 5.0 mL/min, detection: UV 280 nm) to give **B$_2$2** (15.5 mg, 8.10 μmol, 54% over 32 steps): white solid; $[\alpha]_D^{26}$ +37.4° (*c* 0.514, MeOH); IR (film) 1006, 1026, 1050, 1132, 1177, 1202, 1236, 1343, 1358, 1369, 1388, 1441, 1460, 1536, 1649, 1665, 2872, 2934, 2959, 3059, 3288 cm^{-1}; ^{1}H NMR (500 MHz, DMSO-d_6), see Supplementary Table 12; ^{13}C NMR (125 MHz, DMSO-d_6), see Supplementary Table 13; HRMS (ESI-TOF) calcd for $C_{98}H_{137}N_{21}O_{19}Na$ [M+Na]$^+$ 1935.0292, found 1935.0307.

H^+/Na^+ transport assay of bead-derived peptides.[1,2,3] Trisodium 8-hydroxypyrene-1,3,6-trisulfonate (pyranine)-encapsulated large unilamellar vesicles (LUVs) were prepared according to the thin-film hydration method, followed by extrusion through Nuclepore polycarbonate filters (Whatman) mounted in the mini-extruder apparatus (Avanti Polar Lipids). A solution of egg yolk phosphatidylcholine (EYPC, 22.0 mg, 28.9 μmol) in $CHCl_3$ (225 μL) and egg yolk phosphatidylglycerol (1.20 mg, 1.56 μmol) in $CHCl_3$ (60.0 μL) was evaporated, and dried under vacuum for 1 h to form a lipid thin film. The thin film was hydrated and suspended in 1.50 mL of pyranine-containing pH 7.0 HEPES buffer solution (25 mM HEPES, 100 mM NaCl, 1.0 mM pyranine) by sonication. After five-times freeze-thaw cycles, the lipid suspension was extruded 19 times through a polycarbonate filter with 0.1 μm of pore size in the diameter. The external pyranine-containing buffer was replaced by pyranine-free pH 7.0 HEPES buffer solution (25 mM HEPES, 100 mM NaCl) through size exclusion chromatography using a disposable PD-10 column (GE Healthcare). Lipid concentration of the resultant pyranine-encapsulated LUV suspension was deduced from the phosphatidylcholine concentration determined by using Phospholipid C-Test Wako (Fujifilm Wako Pure Chemical). The lipid concentration of the LUV suspension was adjusted to 25 μM with the pyranine-free pH 7.0 HEPES buffer solution (25 mM HEPES, 100 mM NaCl). A suspension of the LUVs was immediately used for the H^+/Na^+ transport assay.

The stock solution of the peptide library was 25-fold diluted with DMSO. The diluted solution of peptide (2.0 μL) and the pyranine-encapsulated LUV suspension (200 μL) were mixed in a black polystyrene flat-bottom 96-well plate (655086, Greiner bio-one). The plate was vortexed at room temperature for 15 min. To the resultant solution was added aqueous 0.50 M NaOH (3.0 μL/well) at room temperature. The plate was vortexed at room temperature for 30 min. The fluorescence (Ex. 444 nm/Em. 510 nm) of each well was measured at room temperature on Gemini EM microplate reader (Molecular Devices). Triton X-100/H_2O (5/95, 3.0 μL) and the LUV suspension were mixed in each plate to determine 100% lysis. The untreated LUV suspension was used to determine 0% lysis. The H^+/Na^+ transport activities of tested peptides (I) were normalized against 100% lysis by Triton X-100 (I_{max}) and 0% lysis (I_0) as following, where I_x is the fluorescence intensity (Supplementary Equation 2).

$$I = 100 \times (I_x - I_0) / (I_{max} - I_0) \tag{2}$$

The data were transformed to the relative values against mean value of the two control wells for the plate. The mean value of the two control wells was set to 1.00.

Mammalian cytotoxicity assay of bead-derived peptides. P388 cells were obtained from Institute of Development Aging and Cancer (Tohoku University). The cells were maintained with growth medium [RPMI1640 with phenol red (Fujifilm Wako Pure Chemical) supplemented with 10% heat-inactivated fetal bovine serum, penicillin G (100 units/mL), and streptomycin (100 μg/mL)] under atmosphere of 5% CO_2 at 37 °C.

P388 cells were cultured in the growth medium at 37 °C according to the above described procedure. The cells were collected by centrifugation at 65 × *g* for 3 min at 4 °C. The collected cells were resuspended into

the growth medium at 2×10^4 cells/mL. The stock solutions of the peptide library were 250-fold diluted with the growth medium. Aliquots of the medium (100 μL) containing peptides and the latter medium containing P388 cells (100 μL) were mixed in a 96-well cell culture plate (TR5003, TrueLine). The final concentration of P388 cells was 1×10^4 cells/mL. After incubation under atmosphere of 5% CO_2 at 37 °C for 92 h, the numbers of viable cells were determined by 2-(4-iodophenyl)-3-(4-nitrophenyl)-5-(2,4-disulfophenyl)-2*H*-tetrazolium, monosodium salt (WST-1) assay. A solution of 1-methoxy-5-methylphenazinium methyl sulfate (1-methoxy PMS) and WST-1 in the growth medium (1-methoxy PMS 0.70 mg/mL in Milli-Q/WST-1 3.6 mg/mL in pH 7.4 HEPES buffer = 1/9) was added to each well of the plate (10.0 μL/well). The plate was incubated for 4 h under atmosphere of 5% CO_2 at 37 °C. The absorbance of each well at 415 nm was measured using a Benchmark microplate reader (Bio-Rad). The cytotoxicities were evaluated as cell viability (%).

Antibacterial activity assay of bead-derived peptides. The stock solution of the peptide library was 4- and 16-fold diluted with DMSO. The non-diluted stock solution, 4-fold diluted solution, and 16-fold diluted solution were transferred to the 96-well round-bottom plate (3367, Corning, 1.0 μL/well). To the solution of peptide in each well was added a suspension of *Streptococcus pyogenes* SSI-9 strain in cation adjusted Mueller Hinton Broth [40.0 μL, 2×10^5 colony forming units (CFU), 21 g/L Mueller Hinton Broth (Difco), 50 mg/L Ca^{2+} (adjusted with $CaCl_2 \cdot 2H_2O$), and 25 mg/L Mg^{2+} (adjusted with $MgCl_2 \cdot 6H_2O$)] supplemented with 5% laked horse blood (Nippon Biotest Laboratories). The plate was incubated at 37 ºC for 18 h, and the precipitation of the grown cells was examined.

MS/MS sequencing analysis. A stock solution of each peptide in DMSO (10.0 μL) was transferred to microtubes. The solution was dried under vacuum at room temperature. The residue was dissolved in a solution of α-cyano-4-hydroxycinnamic acid in $MeCN/H_2O$ (7/3) containing 0.1% TFA (9.993 μL, 4.00 mg/mL) and a solution of diammonium hydrogen citrate in H_2O (0.007 μL, 100 mg/mL). The resultant solution of each peptide was sonicated at room temperature for 5 min and applied to a 384-well MS plate (0.50, 1.0, 3.0, and 5.0 μL/well). To each well of the plate was added a solution of NaCl (150 nmol/well) in a solution of α-cyano-4-hydroxycinnamic acid in $MeCN/H_2O$ (7/3) containing 0.1% TFA (9.993 μL, 4.00 mg/mL) and a solution of diammonium hydrogen citrate in H_2O (0.007 μL, 100 mg/mL). The plate was subjected to the MS/MS sequencing analysis on TOF/TOF 5800 (AB Sciex).

H^+/Na^+ transport assay of purified peptides. The pyranine-encapsulated large unilamellar vesicle (LUV) suspension was prepared as described above.

The purified peptide **A1**, **$B_0$1**–**$B_0$4**, **$B_1$1**–**$B_1$3**, **$B_2$1**, or **$B_2$2** was diluted with DMSO to various concentrations as 5-fold serial dilution. The solution of peptide (2.0 μL) and the pyranine-encapsulated LUV suspension (200 μL) were mixed in a black polystyrene flat-bottom 96-well plate. The suspension was incubated at room temperature for 15 min on a 96-well plate-mixer, and then 0.5 M aqueous NaOH was added to each well (3.0 μL). The resultant suspension was incubated at room temperature for 30 min. The fluorescence (Ex. 444 nm/Em. 510 nm) of each well was measured on Gemini EM microplate reader. Triton X-100/H_2O (5/95, 3.0 μL), DMSO (2.0 μL), and the LUV suspension were mixed in each plate to determine 100% lysis. The mixture of DMSO (2.0 μL) and the LUV suspension was used to determine 0% lysis. The H^+/Na^+ transport activities of tested peptides (I) were normalized against 100% lysis by Triton X-100 (I_{max}) and 0% lysis by DMSO (I_0) according to Supplementary Equation 2, where I_x is the fluorescence intensity.

The H^+/Na^+ transport activities of the purified peptides were evaluated as EC_{50} (nM) by means of three replicates. Sigmoidal curve fittings were performed on R[4] with drc package.[5] Four-parameter logistic model was applied for the fitting (Supplementary Figure 5).

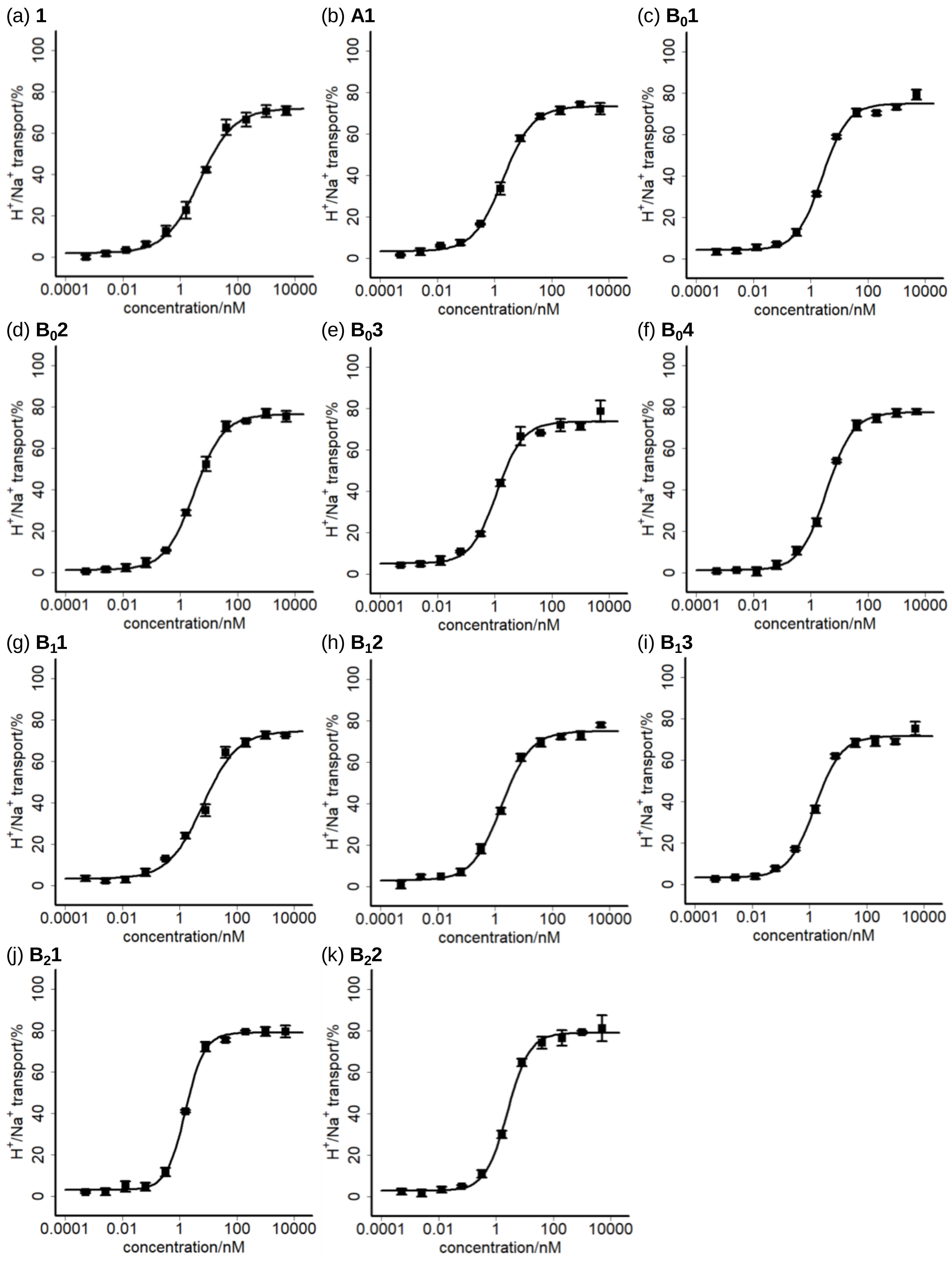

Supplementary Figure 5. Concentration-response curves for H^+/Na^+ transport assay. Representative plots of **1**, **A1**, **B$_0$1**–**B$_0$4**, **B$_1$1**–**B$_1$3**, **B$_2$1**, and **B$_2$2** towards liposomes (EYPC/EYPG = 19/1) from three independent experiments were shown as mean ± SD. Source data are provided as a Source Data file.

Mammalian cytotoxicity assay of purified peptides. The purified peptide **A1**, **$B_0$1–$B_0$4**, **$B_1$1–$B_1$3**, **$B_2$1**, or **$B_2$2** was diluted with the growth medium with 2% DMSO to various concentrations as 5-fold serial dilution. P388 cells were cultured in the growth medium at 37 °C according to the above described procedure. The cells were collected by centrifugation at 65 × *g* for 3 min at 4 °C. The collected cells were resuspended into the growth medium at 2×10^4 cells/mL. Aliquots of the former medium (2% DMSO, 100 µL) containing peptides and the latter medium containing P388 cells (100 µL) were mixed in a 96-well cell culture plate (TR5003, TrueLine). The final concentration of P388 cells and DMSO were 1×10^4 cells/mL and 1%, respectively. After incubation for 92 h under atmosphere of 5% CO_2 at 37 °C, the numbers of viable cells were determined by WST-1 assay. A solution of 1-methoxy PMS and WST-1 in the growth medium (1-methoxy PMS 0.70 mg/mL in Milli-Q/WST-1 3.6 mg/mL in pH 7.4 HEPES buffer = 1/9) was added to each well of the plate (20.0 µL/well). The cells were incubated for 4 h under atmosphere of 5% CO_2 at 37 °C. The absorbance of each well at 415 nm was measured using a Benchmark microplate reader (Bio-Rad) or Thermo Scientific Multiskan GO (Thermo Fisher Scientific). The cytotoxicities of the purified peptides were evaluated as IC_{50} (nM) by means of three replicates. Sigmoidal curve fittings were performed on R[4] with drc package.[5] Four-parameter logistic model was applied for the fitting (Supplementary Figure 6).

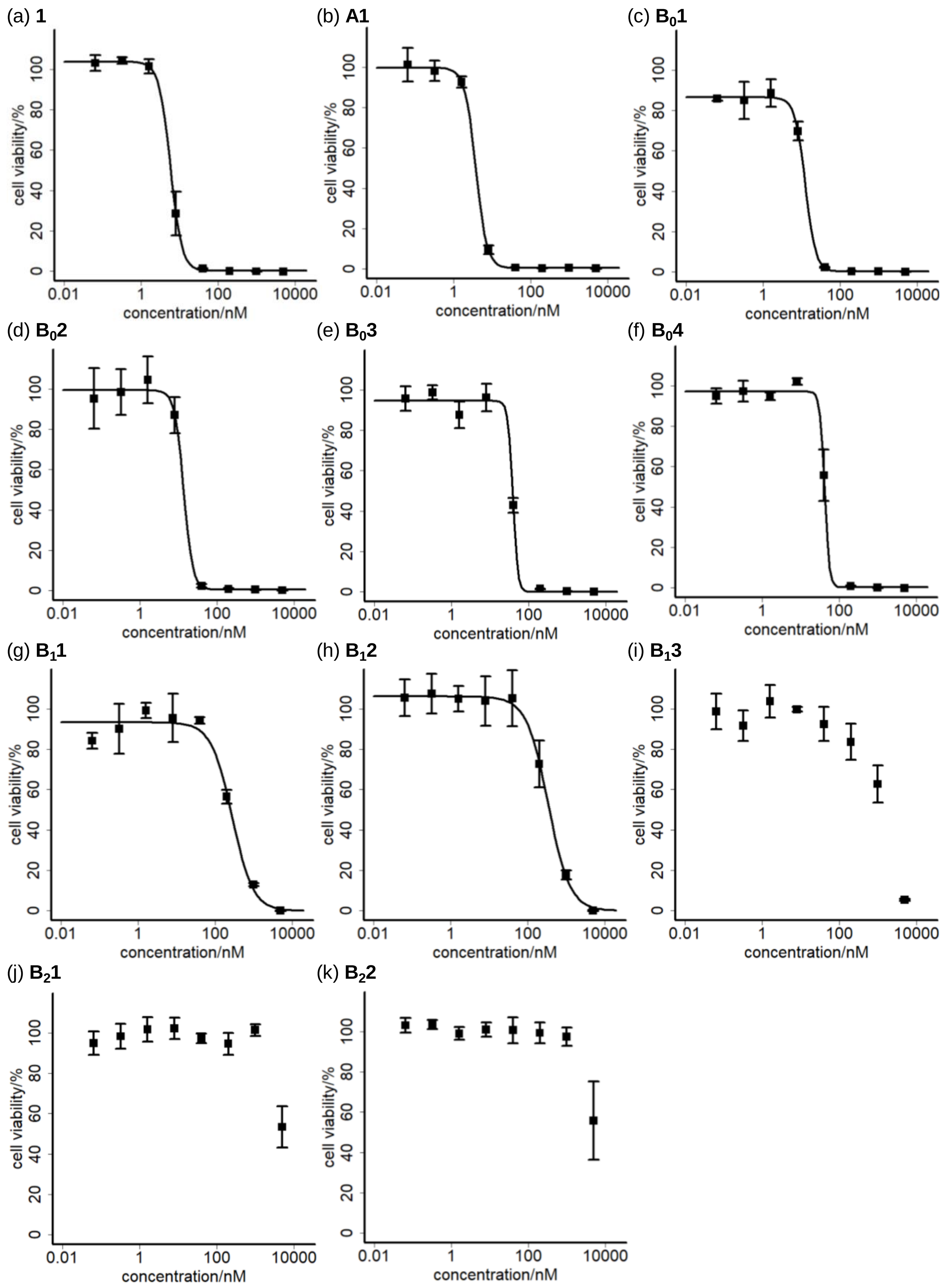

Supplementary Figure 6. Concentration-response curves for mammalian cytotoxicity assay. Representative plots of **1**, **A1**, **B$_0$1**–**B$_0$4**, **B$_1$1**–**B$_1$3**, **B$_2$1**, and **B$_2$2** against P388 cells from three independent experiments were shown as mean ± SD. Source data are provided as a Source Data file.

Hemolysis assay of purified peptides. The purified peptide **A1**, **$B_0$1–$B_0$4**, **$B_1$1–$B_1$3**, **$B_2$1**, or **$B_2$2** was diluted with DMSO to various concentrations as 3-fold serial dilution. A 50% suspension of rabbit blood in Alsever's solution was obtained from Nippon Bio-Supp. Center. The blood suspension (250 μL) was diluted with phosphate buffered saline (no calcium, no magnesium, 1.00 mL). The resultant suspension was centrifuged at 1000 × *g* for 3 min at 8 °C. The supernatant was discarded, and the collected cells were resuspended in phosphate buffered saline (no calcium, no magnesium, 1.00 mL). The same procedure was repeated (× 3). The resultant suspension (100 μL) was diluted with phosphate buffered saline (no calcium, no magnesium, 4.90 mL) to give 0.2% red blood cells suspension. The 0.2% red blood cells suspension (95.0 μL) and each dilution series of the purified peptide in DMSO (5.0 μL) were mixed in 2 mL microtube. After incubation at 37 °C for 4 h, the suspension was centrifuged at 1000 × *g* for 3 min at 8 °C. The supernatant was transferred to a 96-well plate (TR5003, TrueLine). The absorbance of each well at 415 nm was measured using a Thermo Scientific Multiskan GO (Thermo Fisher Scientific). The mixture of Triton X-100/H_2O (5/95, 2.0 μL), DMSO (5.0 μL), and the 0.2% red blood cells suspension (95.0 μL) was used to determine 100% hemolysis. The mixture of DMSO (5.0 μL) and the 0.2% red blood cells suspension (95.0 μL) was used to determine 0% hemolysis. The hemolytic activities of tested peptides (I) were normalized against 100% hemolysis by Triton X-100 (I_{max}) and 0% hemolysis by DMSO (I_0) according to Supplementary Equation 3, where I_x is the UV absorbance.

$$I = 100 \times (I_x - I_0) / (I_{max} - I_0) \quad (3)$$

The hemolytic activities of the purified peptides were evaluated as the concentrations causing 10% hemolysis (HC_{10}, nM) by means of three replicates. Sigmoidal curve fittings were performed on GraphPad Prism (GraphPad software, Supplementary Figure 7).

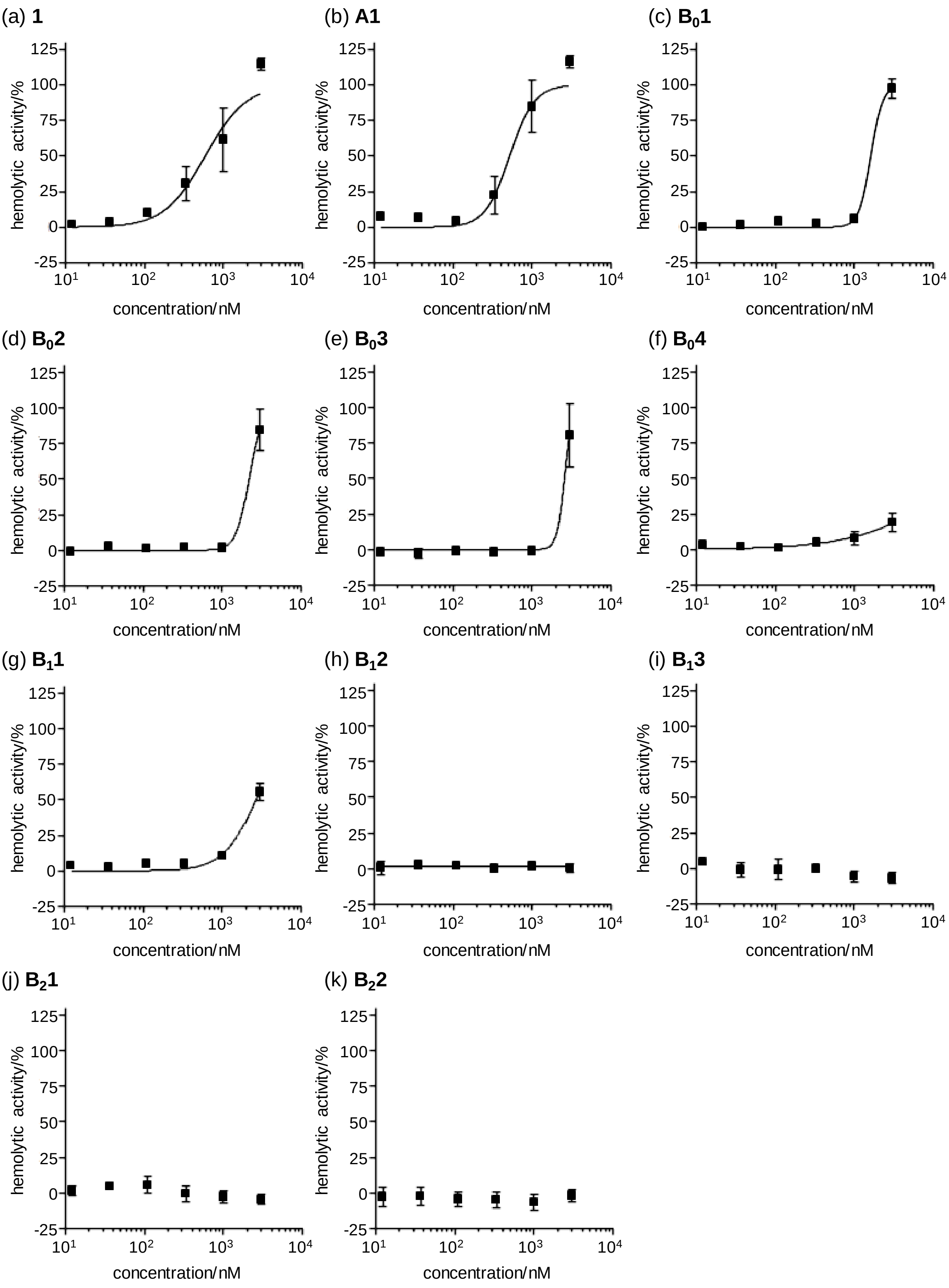

Supplementary Figure 7. Concentration-response curves for hemolysis assay. Plots of **1**, **A1**, **B$_0$1**–**B$_0$4**, **B$_1$1**–**B$_1$3**, **B$_2$1**, and **B$_2$2** towards rabbit red blood cells from three independent experiments were shown as mean ± SD. Source data are provided as a Source Data file.

Evaluation of antibacterial spectra of purified peptides. The MIC assay was performed according to the Clinical and Laboratory Standards Institute protocols. The antibacterial activities of the purified peptides **A1**, **$B_0$1–$B_0$4**, **$B_1$1–$B_1$3**, **$B_2$1**, and **$B_2$2** were measured using the broth microdilution method.[6] Serial dilutions of the peptides were prepared in cation adjusted Mueller Hinton Broth. Each dilution (100 μL) was dispensed to each well of the round-bottom plate to obtain final concentrations ranging from 64 μg/mL to 0.00024 μg/mL. Bacteria were grown at 37 °C overnight on Tryptic Soy Broth or Mueller Hinton Broth agar plates. The bacterial colonies were suspended in 0.9% saline to obtain OD_{625} of 0.08–0.13 using a spectrophotometer UV-1280 (Shimadzu). The resultant suspension was diluted with cation adjusted Mueller Hinton Broth (1/20). The resultant mixture (10.0 μL) was inoculated to each well of the plates (approximately 5 × 10^4 CFU/well), mixed, and incubated at 37 °C for 18–24 h. The MIC value was determined as the minimum concentration that inhibited growth of bacteria. For *Streptococcus* species, cation adjusted Mueller Hinton Broth supplemented with 5% laked horse blood was used.

Membrane disruption assay of purified peptides. 5(6)-Carboxyfluorescein (CF)-encapsulated large unilamellar vesicles (LUVs) were prepared according to the thin-film hydration method, followed by extrusion through Nuclepore polycarbonate filters mounted in the mini-extruder apparatus. A solution of egg yolk phosphatidylcholine (EYPC, 22.0 mg, 28.9 μmol) in $CHCl_3$ (225 μL) and egg yolk phosphatidylglycerol (1.20 mg, 1.56 μmol) in $CHCl_3$ (60.0 μL) was evaporated, and dried under vacuum for 1 h to form a lipid thin film. The thin film was hydrated and suspended in 1.50 mL of CF-containing pH 7.0 HEPES buffer solution (25 mM HEPES, 100 mM NaCl, 20 mM CF) by sonication. After five-times freeze-thaw cycles, the lipid suspension was extruded 19 times through a polycarbonate filter with 0.1 μm of pore size in the diameter. The external CF-containing buffer was replaced by CF-free pH 7.0 HEPES buffer solution (25 mM HEPES, 100 mM NaCl) through size exclusion chromatography using a disposable PD-10 column. Lipid concentration of the resultant CF-encapsulated LUV suspension was deduced from the phosphatidylcholine concentration determined by using Phospholipid C-Test Wako. The lipid concentration of the LUV suspension was adjusted to 25 μM with the CF-free pH 7.0 HEPES buffer solution (25 mM HEPES, 100 mM NaCl). A suspension of the LUVs was immediately used for the membrane disruption assay.

The purified peptide **A1**, **$B_0$1–$B_0$4**, **$B_1$1–$B_1$3**, **$B_2$1**, or **$B_2$2** was diluted with DMSO to various concentrations as 5-fold serial dilution. The solution of peptide (2.0 μL) and the CF-encapsulated LUV suspension (200 μL) were mixed in a black polystyrene flat-bottom 96-well plate. The resultant suspension was incubated at room temperature for 30 min. The fluorescence (Ex. 490 nm/Em. 517 nm) of each well was measured on Gemini EM microplate reader. Triton X-100/H_2O (5/95, 3.0 μL), DMSO (2.0 μL), and the LUV suspension were mixed in each plate to determine 100% lysis. The mixture of DMSO (2.0 μL) and the LUV suspension was used to determine 0% lysis. The membrane disruption activities of tested peptides (I) were normalized against 100% lysis by Triton X-100 (I_{max}) and 0% lysis by DMSO (I_0) according to Supplementary Equation 2, where I_x is the fluorescence intensity.

The membrane disruption activities of the purified peptides were plotted as means of three replicates (Supplementary Figure 1).

CD measurement of purified peptides in liposomes. Large unilamellar vesicles (LUVs) were prepared according to the thin-film hydration method, followed by extrusion through Nuclepore polycarbonate filters mounted in the mini-extruder apparatus. A solution of 1-palmitoyl-2-oleoyl-*sn*-glycero-3-phosphocholine (POPC, 22.5 mg, 29.6 μmol) in $CHCl_3$ (225 μL) and 1-palmitoyl-2-oleoyl-*sn*-glycero-3-phosphatidylglycerol (POPG, 1.20 mg, 1.56 μmol) in $CHCl_3$/MeOH (3/1, 60.0 μL) was evaporated, and dried under vacuum for 1 h to form a lipid thin film. The thin film was hydrated and suspended in 1.50 mL of pH 7.0 HEPES buffer solution (25 mM HEPES) by sonication. After five-times freeze-thaw cycles, the lipid suspension was extruded 19 times through a polycarbonate filter with 0.1 μm of pore size in the diameter. The resultant LUV suspensions were prepared by pH 7.0 HEPES buffer solution (25 mM HEPES) through size exclusion chromatography using a disposable PD-10 column. Lipid concentration of the resultant LUV suspension was deduced from the phosphatidylcholine concentration determined by using Phospholipid C-Test Wako. The lipid concentration of the LUV suspension was adjusted to 1.0 mM with the pH 7.0 HEPES buffer solution (25 mM HEPES). A suspension of the LUVs was immediately used for the CD measurements.

To a solution of purified peptide **A1**, $\mathbf{B_0 1}$–$\mathbf{B_0 4}$, $\mathbf{B_1 1}$–$\mathbf{B_1 3}$, $\mathbf{B_2 1}$, or $\mathbf{B_2 2}$ (20.0 nmol) in MeOH (10.0 μL) was added a suspension of LUVs (390 μL). CD spectra were recorded in a 0.2 cm pathlength cuvette at 25 °C using a J-820 spectropolarimeter equipped with Peltier thermostatted cell holder (JASCO). Data were acquired for 210–280 nm at every 0.5 nm with a standard sensitivity mode (100 mdeg) applying 2 nm band width. Each measurement was repeated four times at a scanning speed of 100 nm/min with a response time of 2 sec and at 25 °C. In all cases, the peptide-free lipid suspension spectra were subtracted from the peptide-lipid suspension spectra. Intensity of CD spectra were expressed in units of molar ellipticity (deg cm^2 $dmol^{-1}$) and plotted against the wavelength (nm) (Supplementary Figure 2).

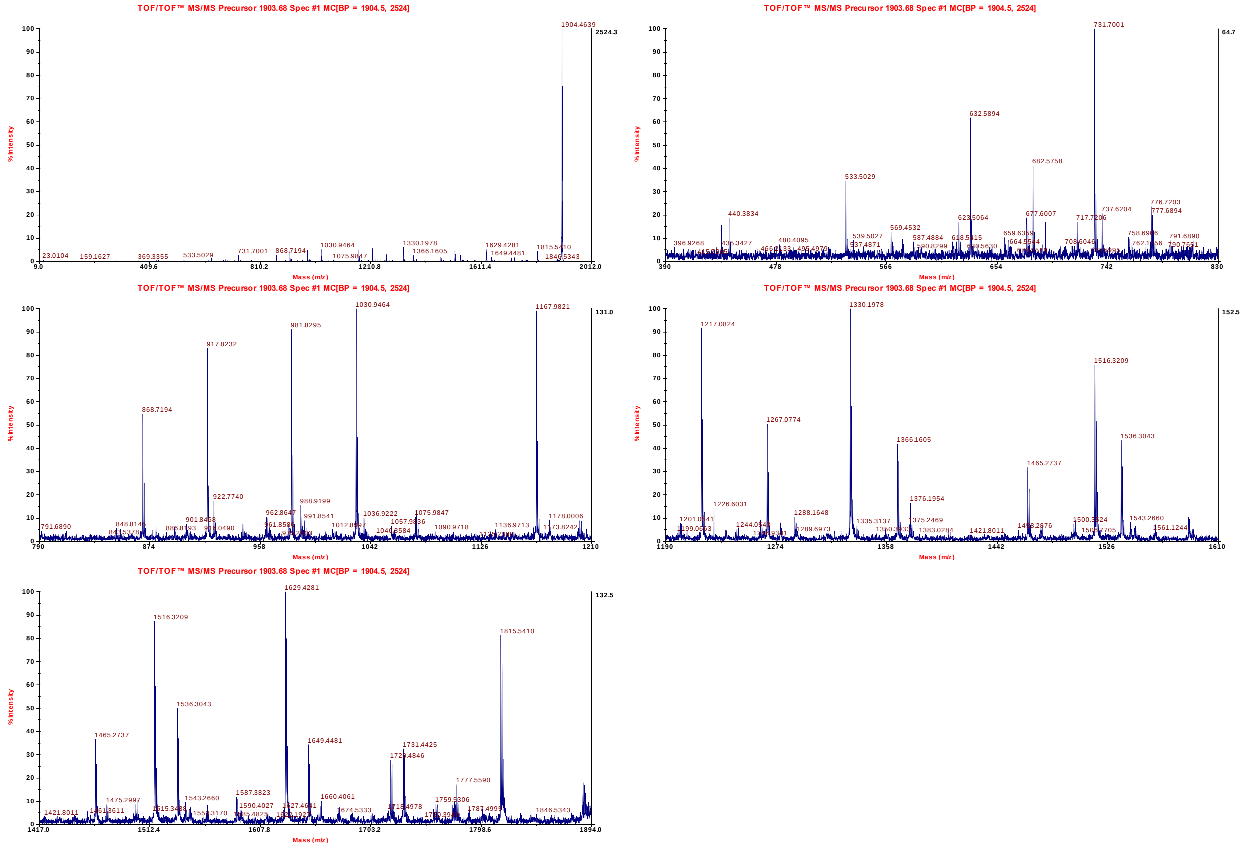

Supplementary Figure 8. MS/MS spectra of **1**. Mass range: *m/z* 9–2012.

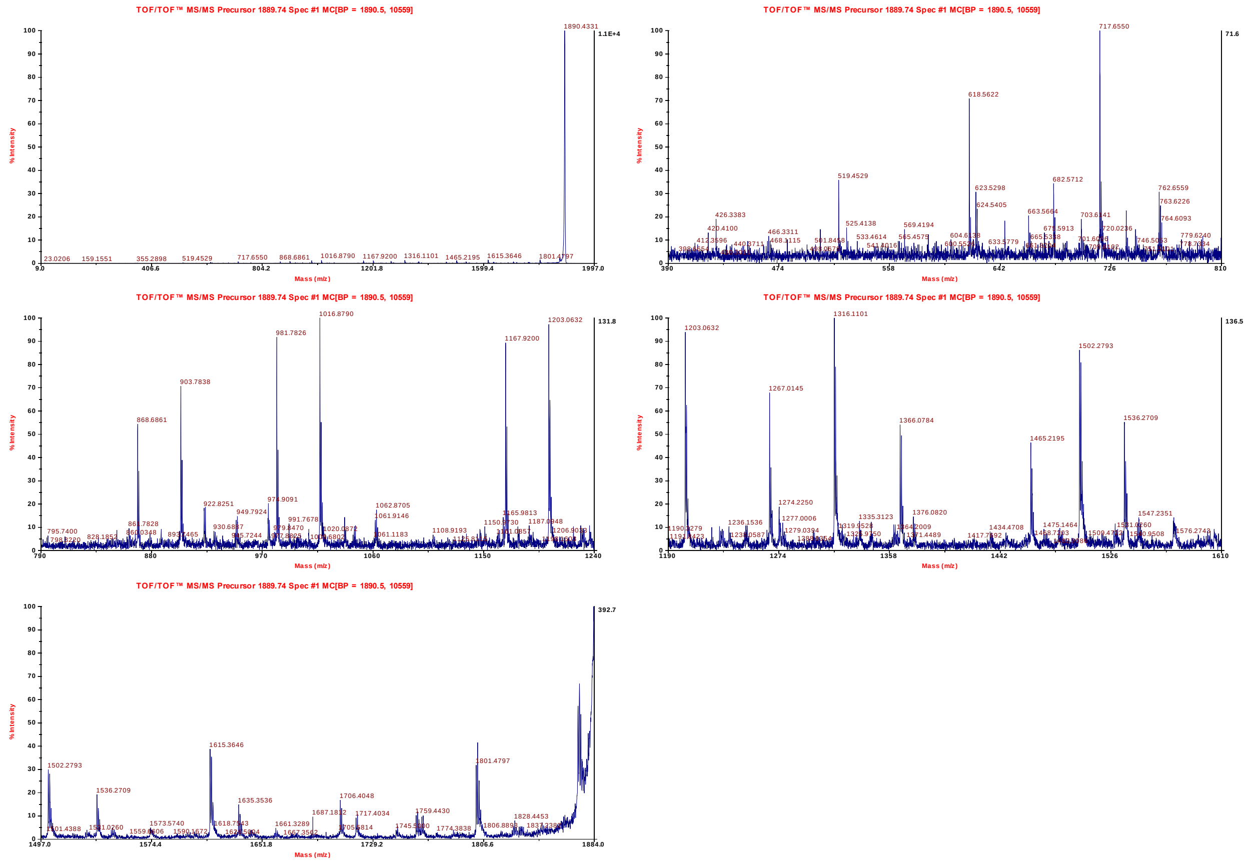

Supplementary Figure 9. MS/MS spectra of **A1**. Mass range: *m/z* 9–1997.

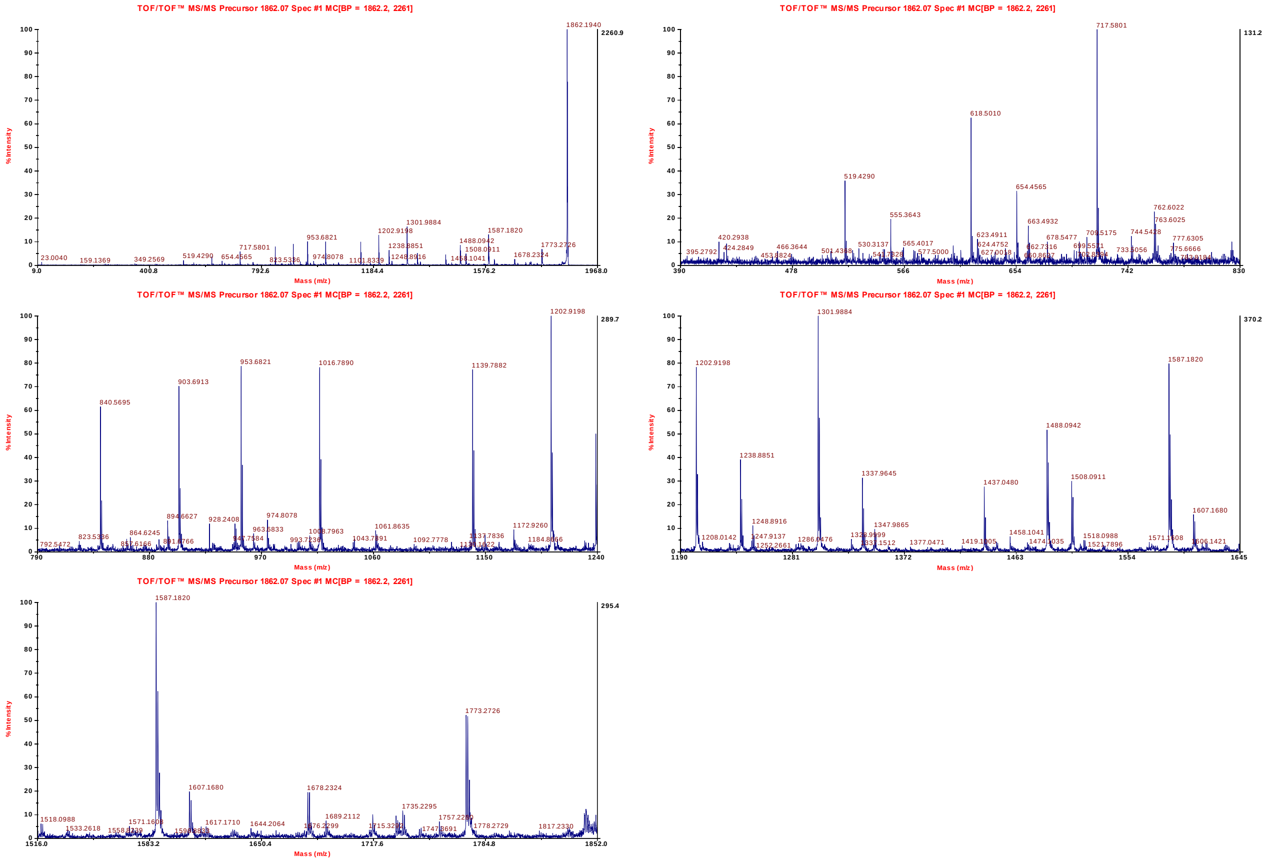

Supplementary Figure 10. MS/MS spectra of $\mathbf{B_01}$. Mass range: *m*/*z* 9–1968.

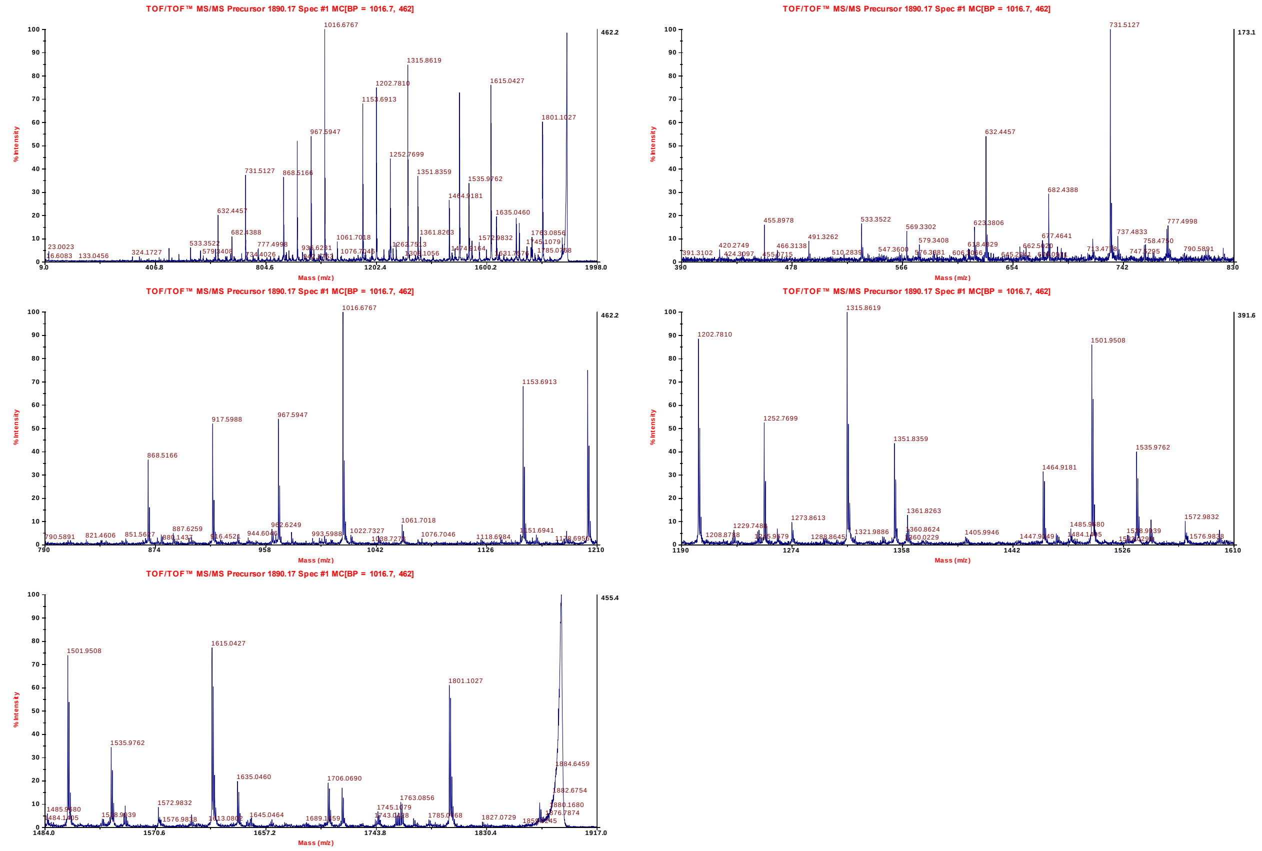

Supplementary Figure 11. MS/MS spectra of $\mathbf{B_02}$. Mass range: *m*/*z* 9–1998.

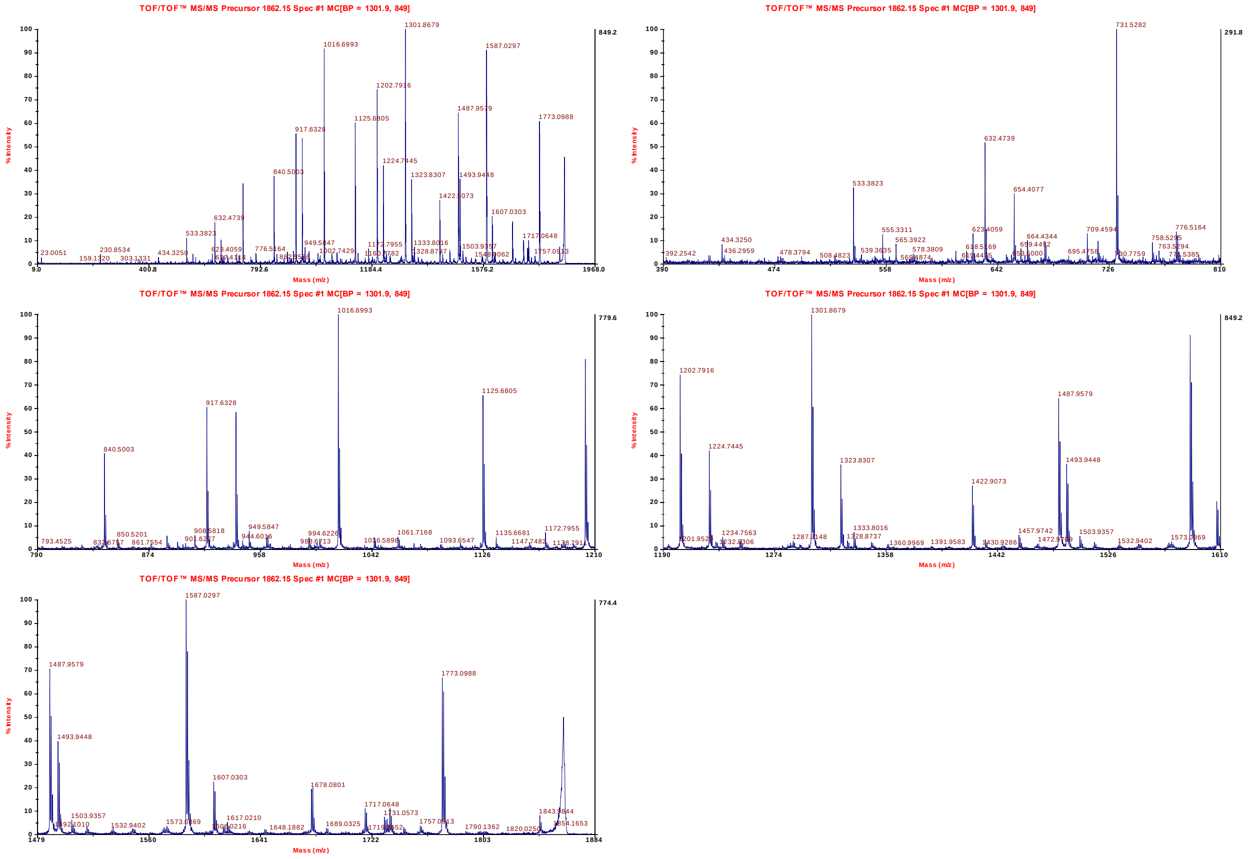

Supplementary Figure 12. MS/MS spectra of **B₀3**. Mass range: *m/z* 9–1968.

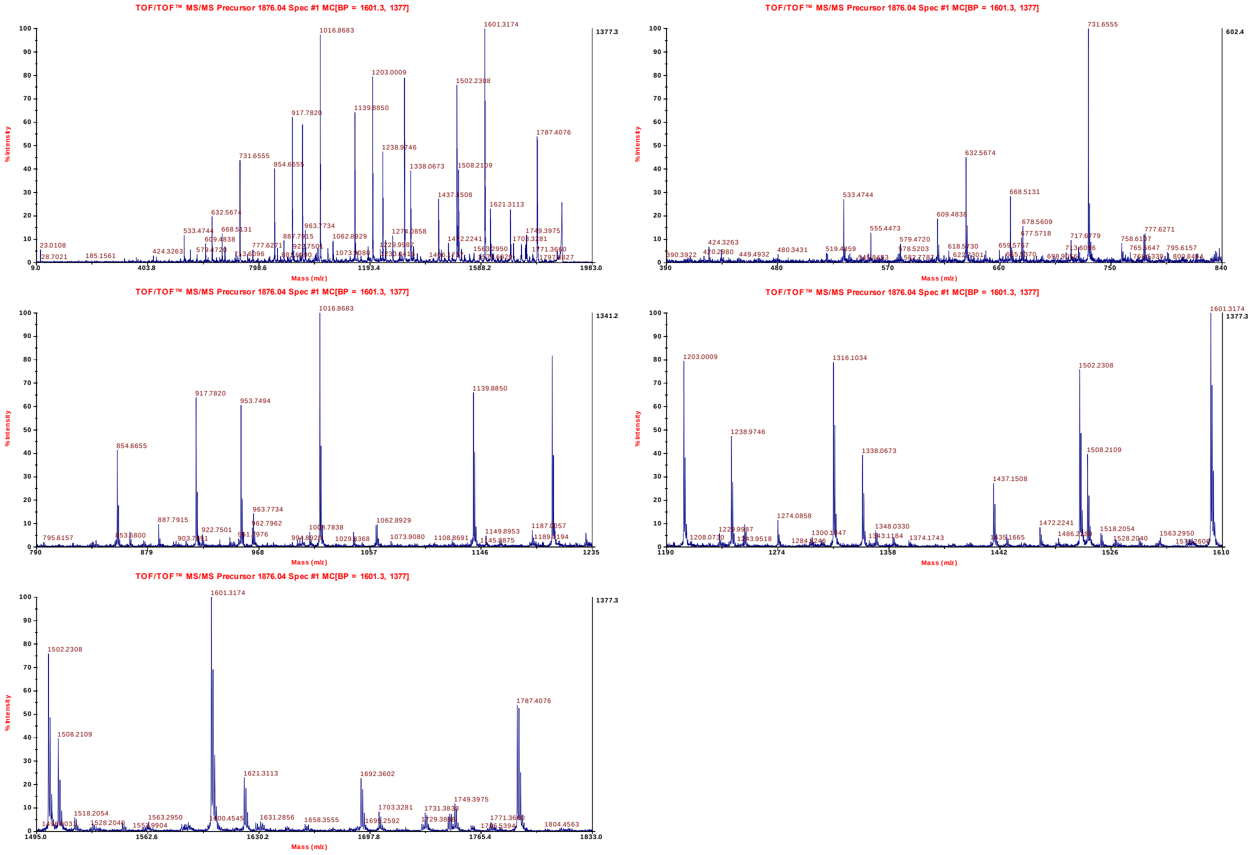

Supplementary Figure 13. MS/MS spectra of **B₀4**. Mass range: *m/z* 9–1983.

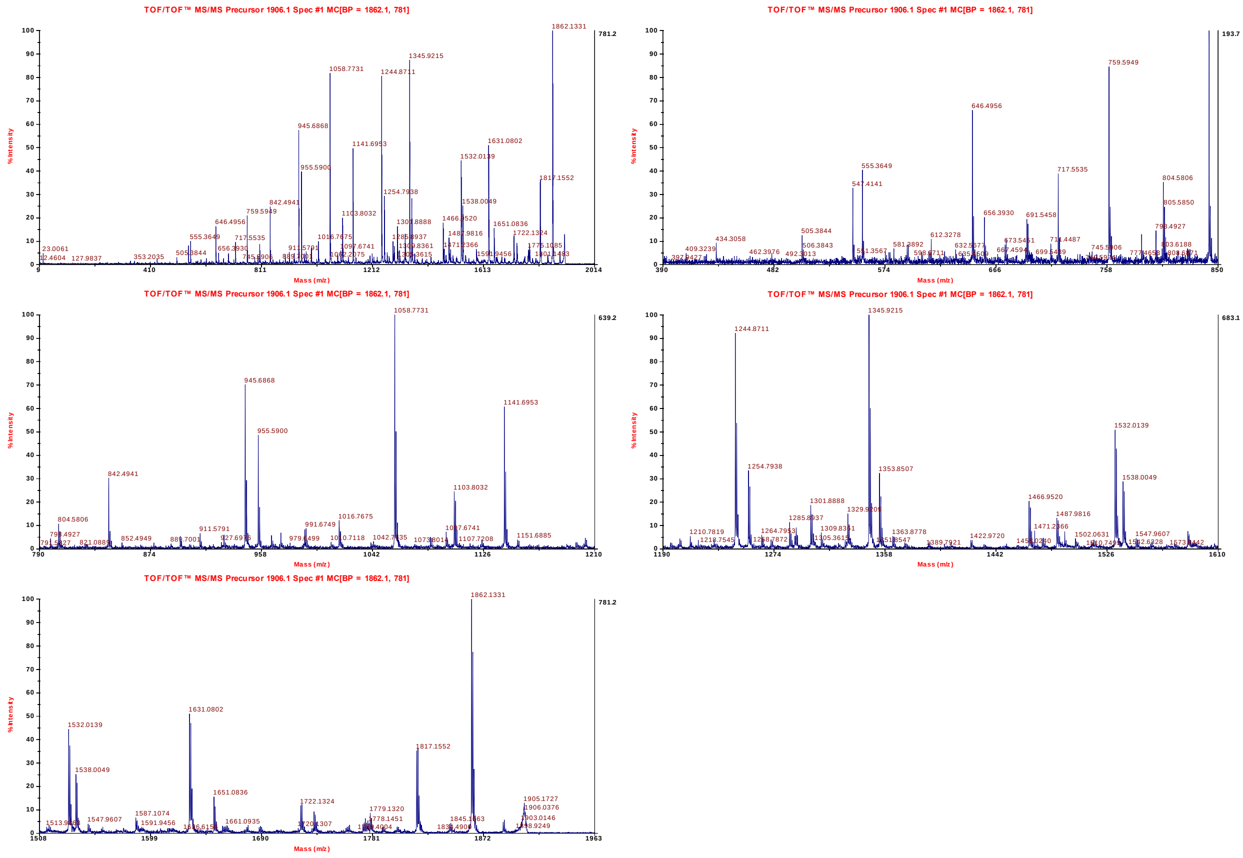

Supplementary Figure 14. MS/MS spectra of **B$_1$1**. Mass range: *m*/*z* 9–2014.

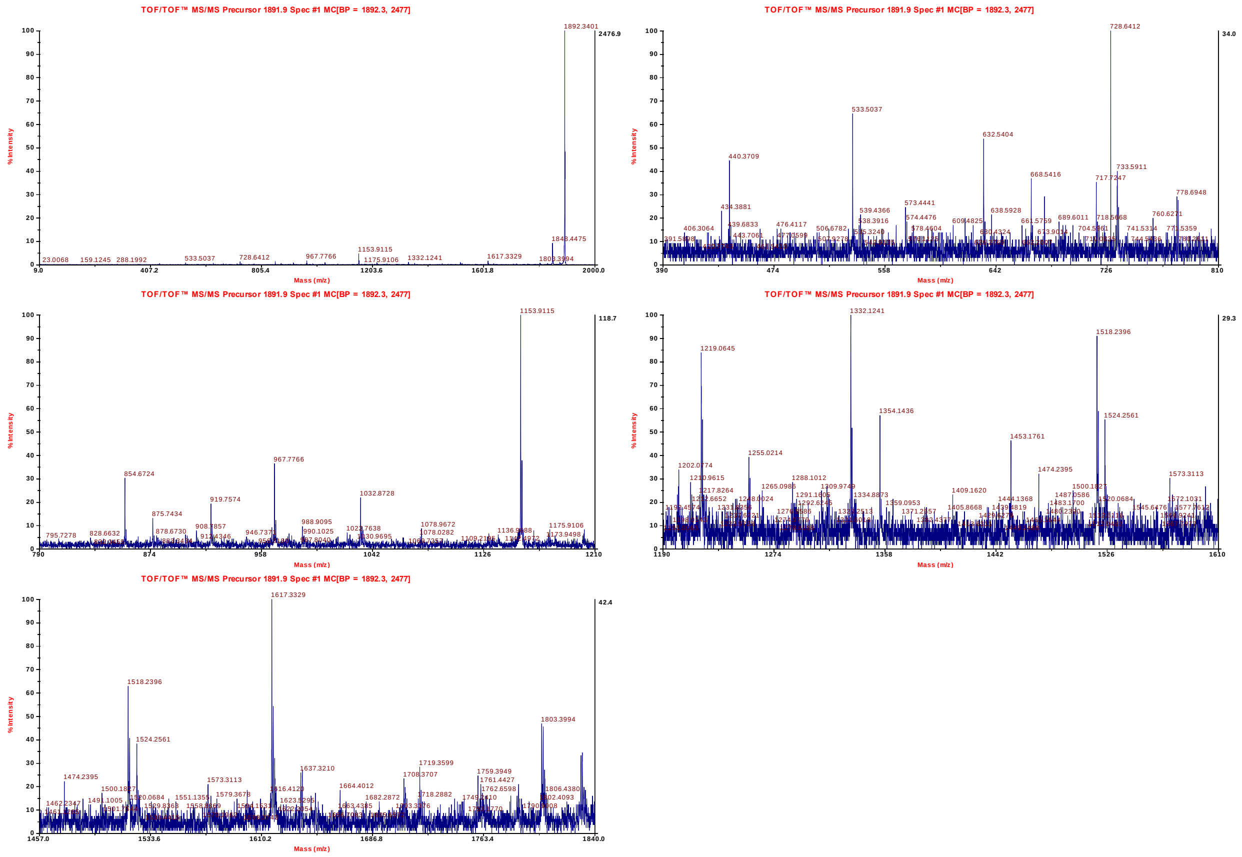

Supplementary Figure 15. MS/MS spectra of **B$_1$2**. Mass range: *m*/*z* 9–2000.

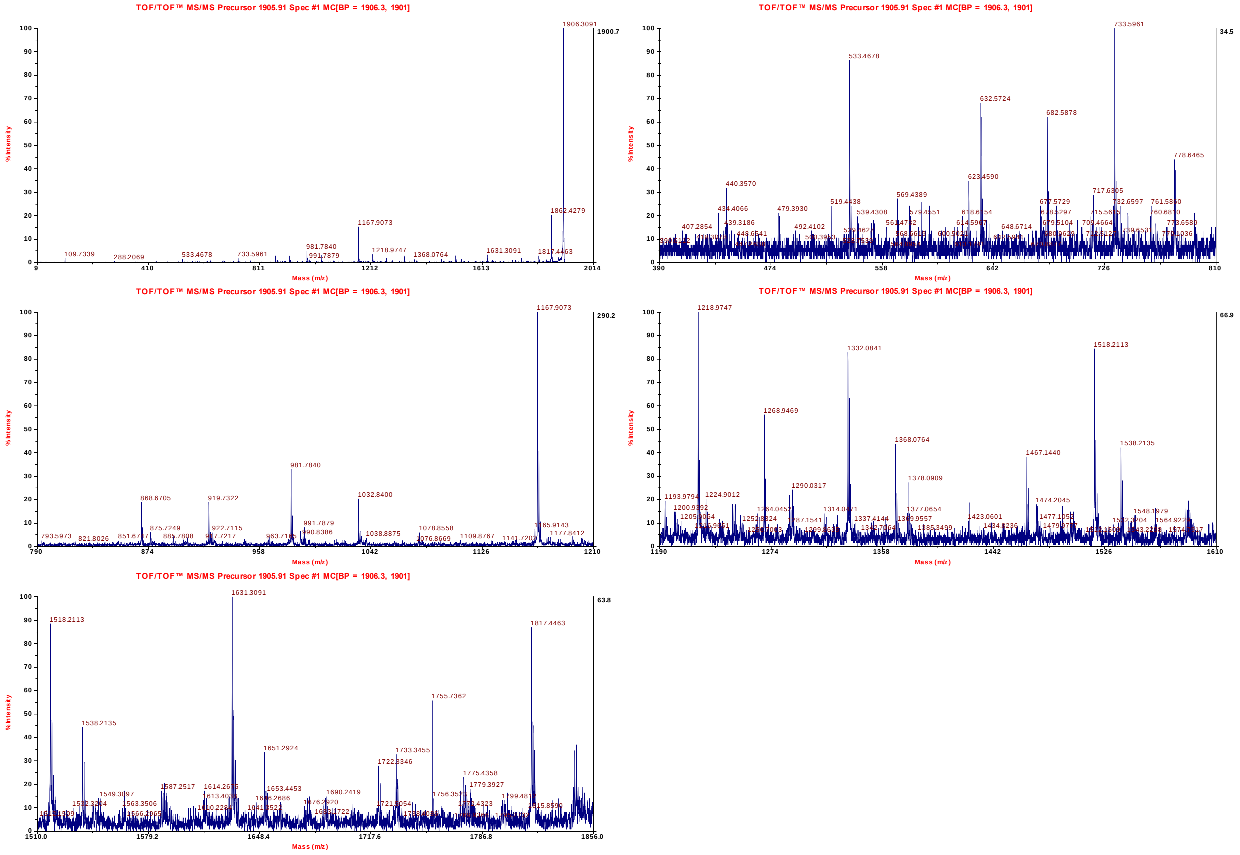

Supplementary Figure 16. MS/MS spectra of **B**$_1$**3**. Mass range: *m*/*z* 9–2014.

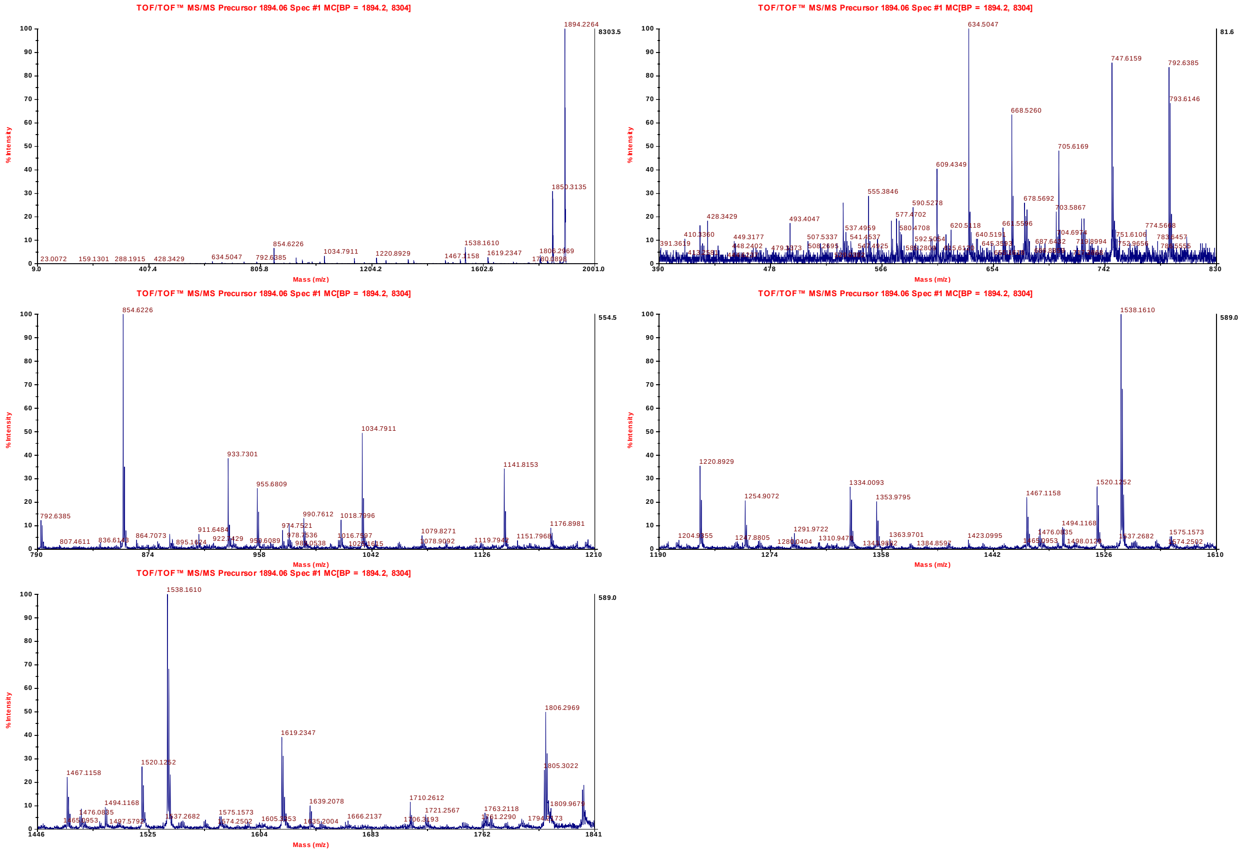

Supplementary Figure 17. MS/MS spectra of **B**$_2$**1**. Mass range: *m*/*z* 9–2001.

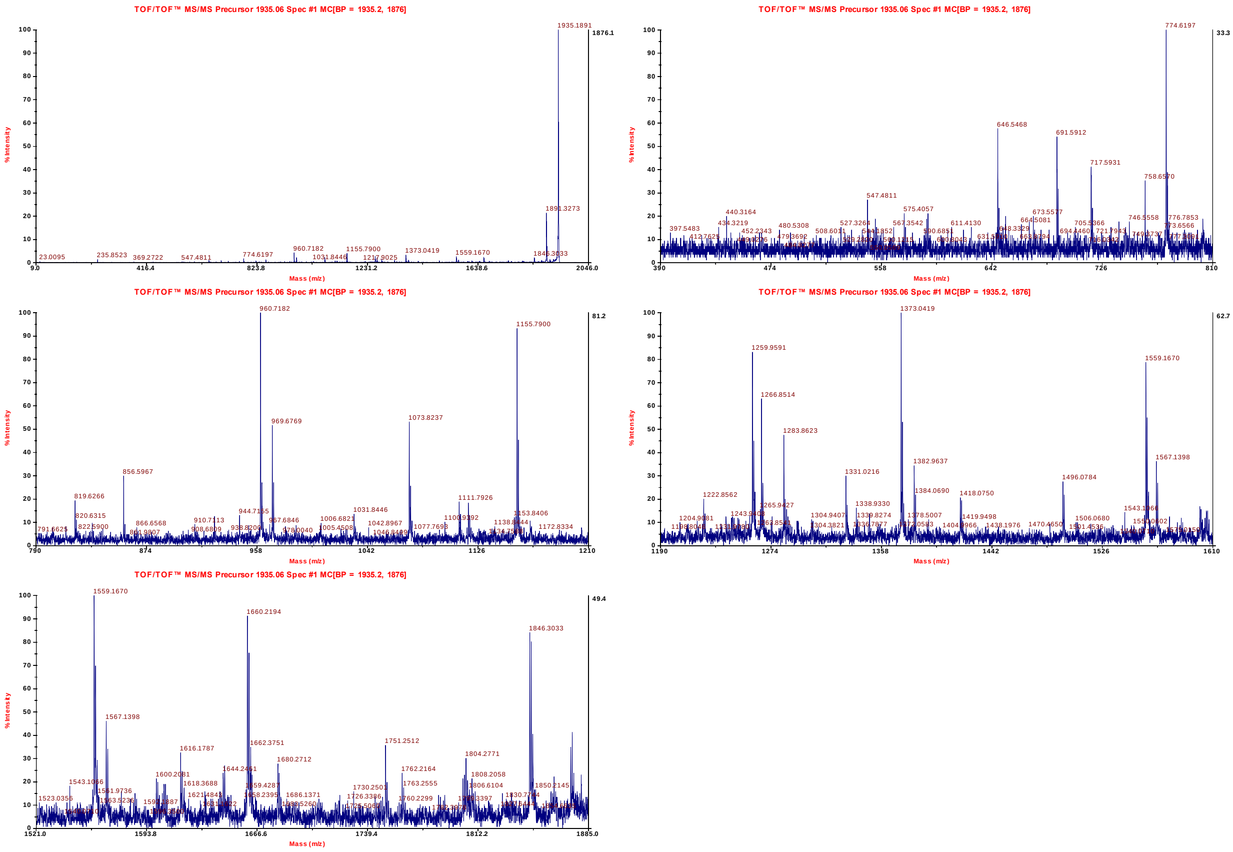

Supplementary Figure 18. MS/MS spectra of **B₂2**. Mass range: *m*/*z* 9–2046.

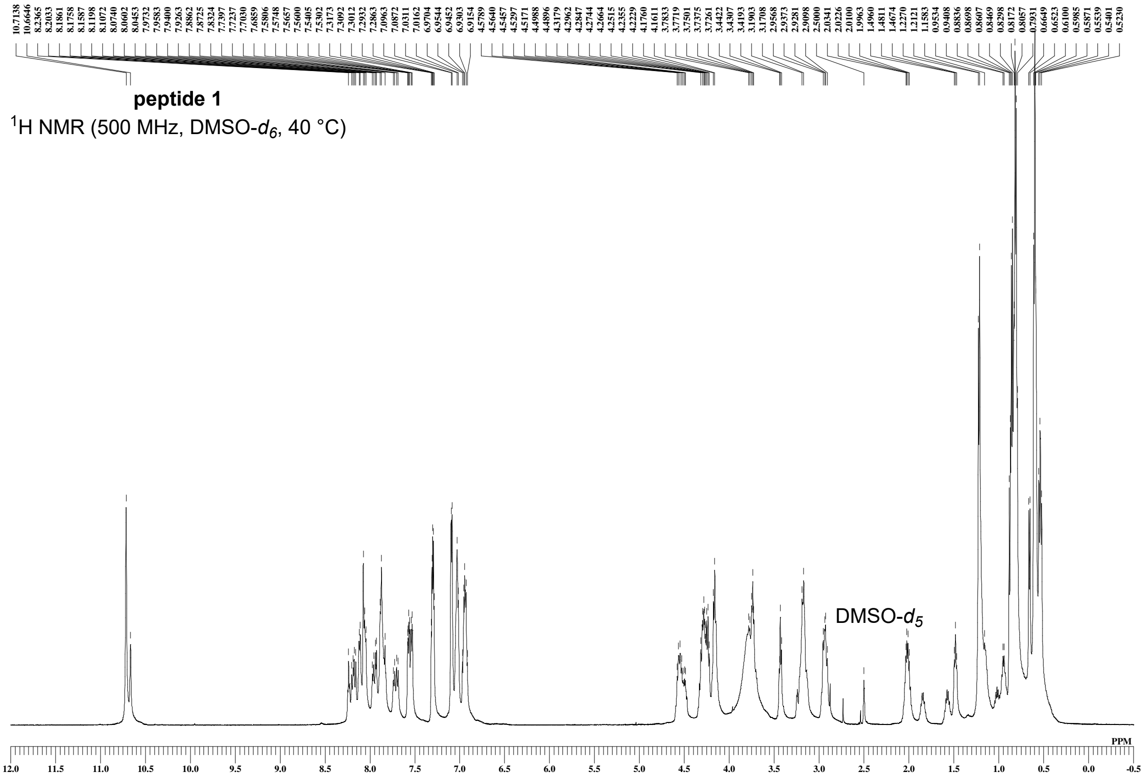

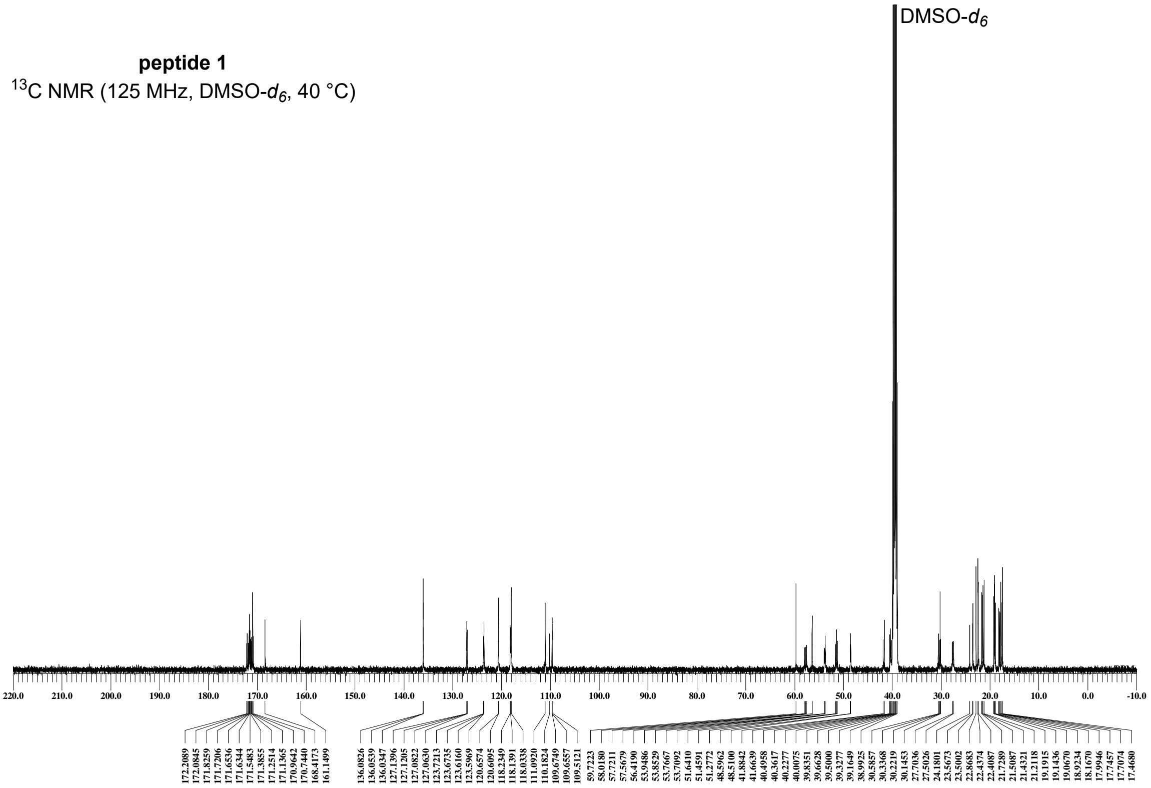

Supplementary Figure 19. ^{1}H and ^{13}C NMR spectra of **1**. The spectra were obtained in DMSO-d_6 at 40 °C.

peptide 1

^{1}H–^{1}H DQF-COSY (500 MHz, DMSO-d_6, 40 °C)

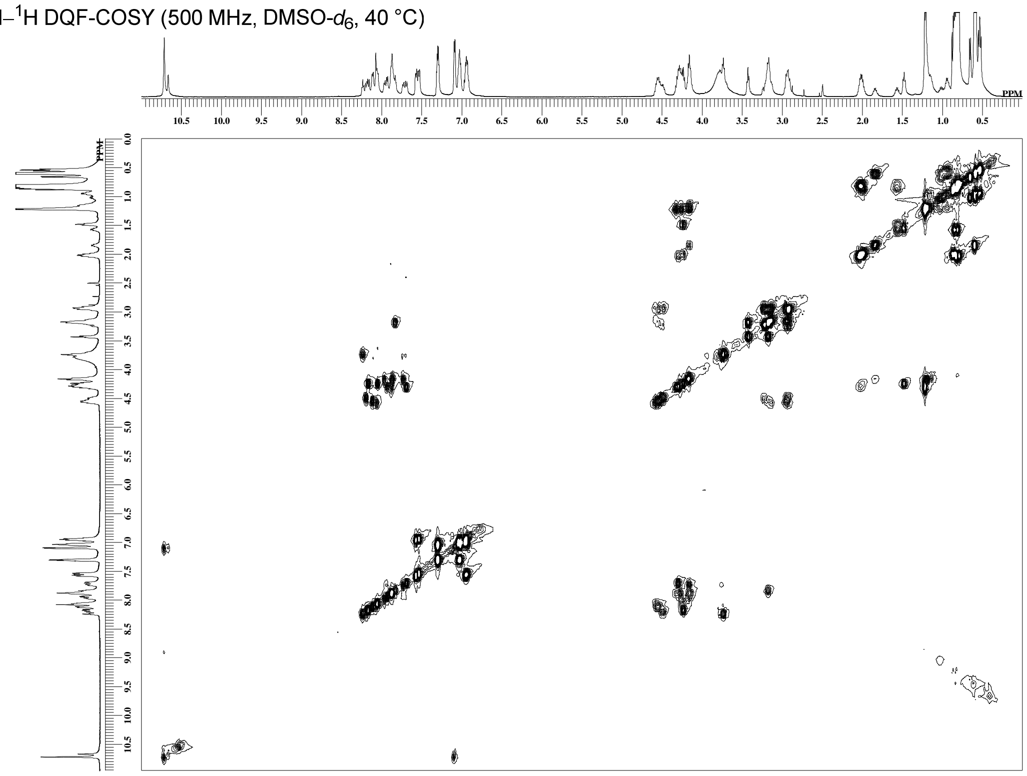

peptide 1

^{1}H–^{1}H TOCSY (500 MHz, DMSO-d_6, 40 °C)

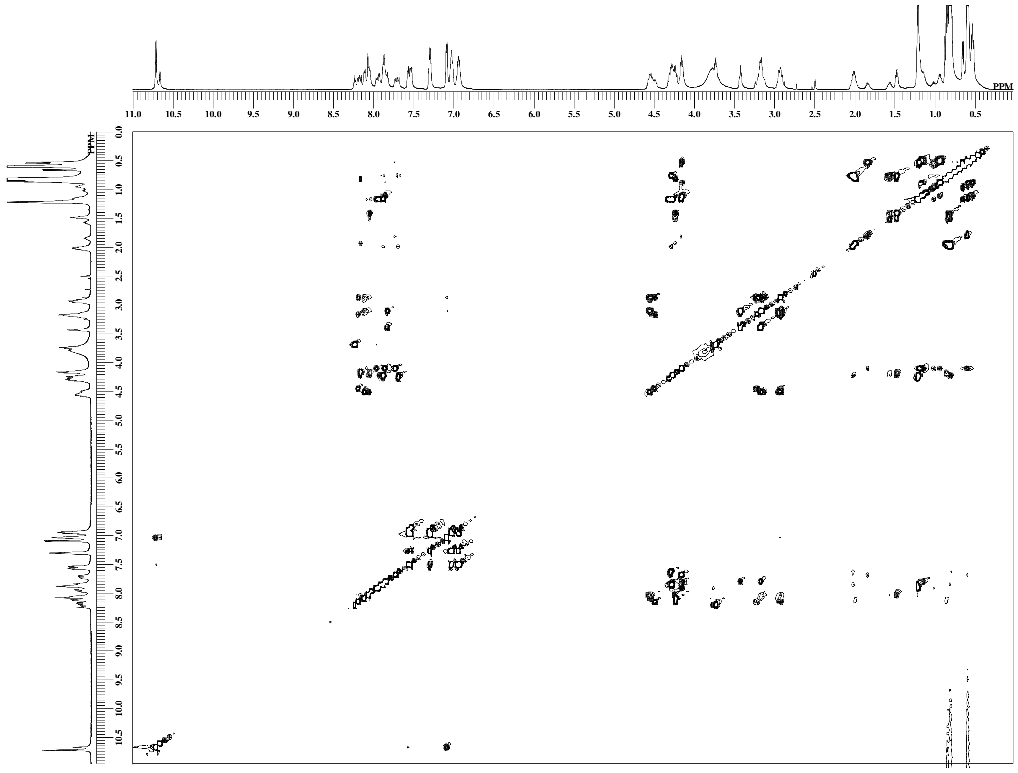

Supplementary Figure 20. ^{1}H–^{1}H DQF-COSY and ^{1}H–^{1}H TOCSY spectra of **1**. The spectra were obtained in DMSO-d_6 at 40 °C.

peptide 1

^{1}H–^{1}H NOESY (500 MHz, DMSO-d_6, 40 °C)

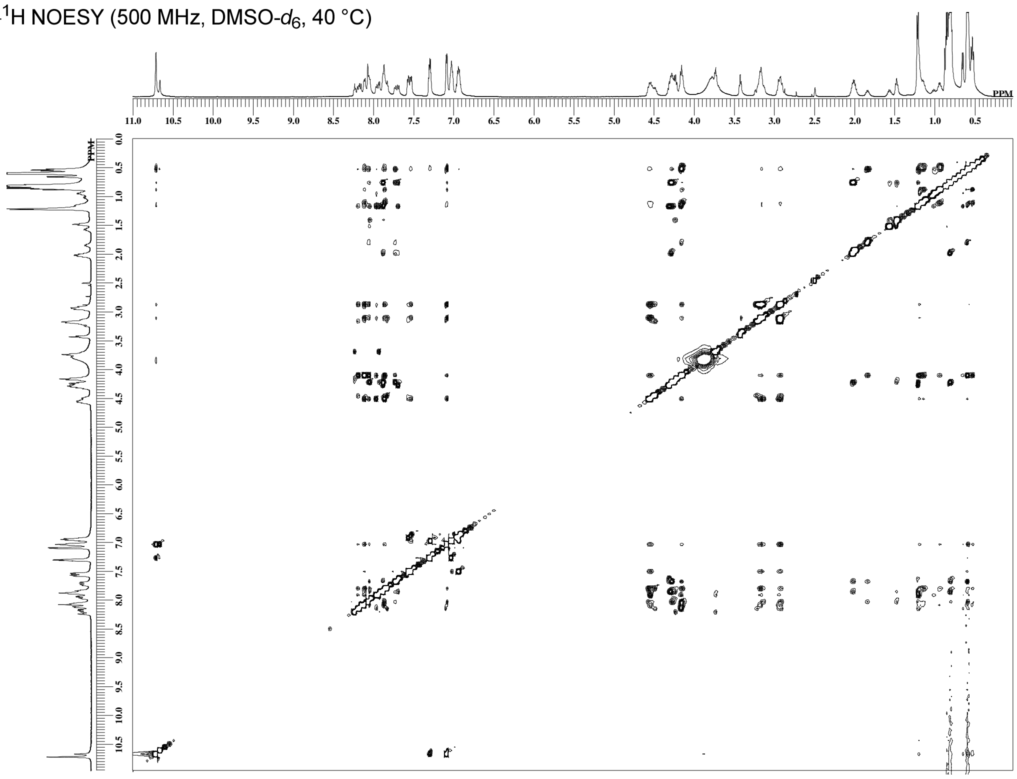

peptide 1

^{1}H–^{13}C HMBC (500 MHz, DMSO-d_6, 40 °C)

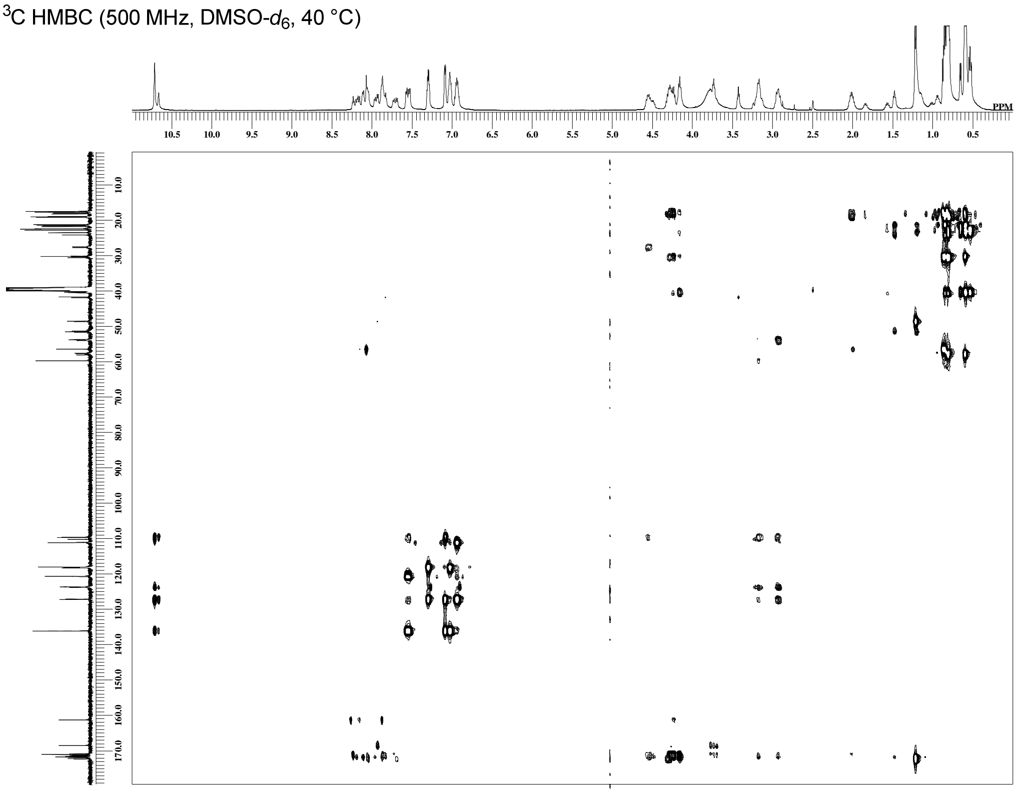

Supplementary Figure 21. ^{1}H–^{1}H NOESY and ^{1}H–^{13}C HMBC spectra of **1**. The spectra were obtained in DMSO-d_6 at 40 °C.

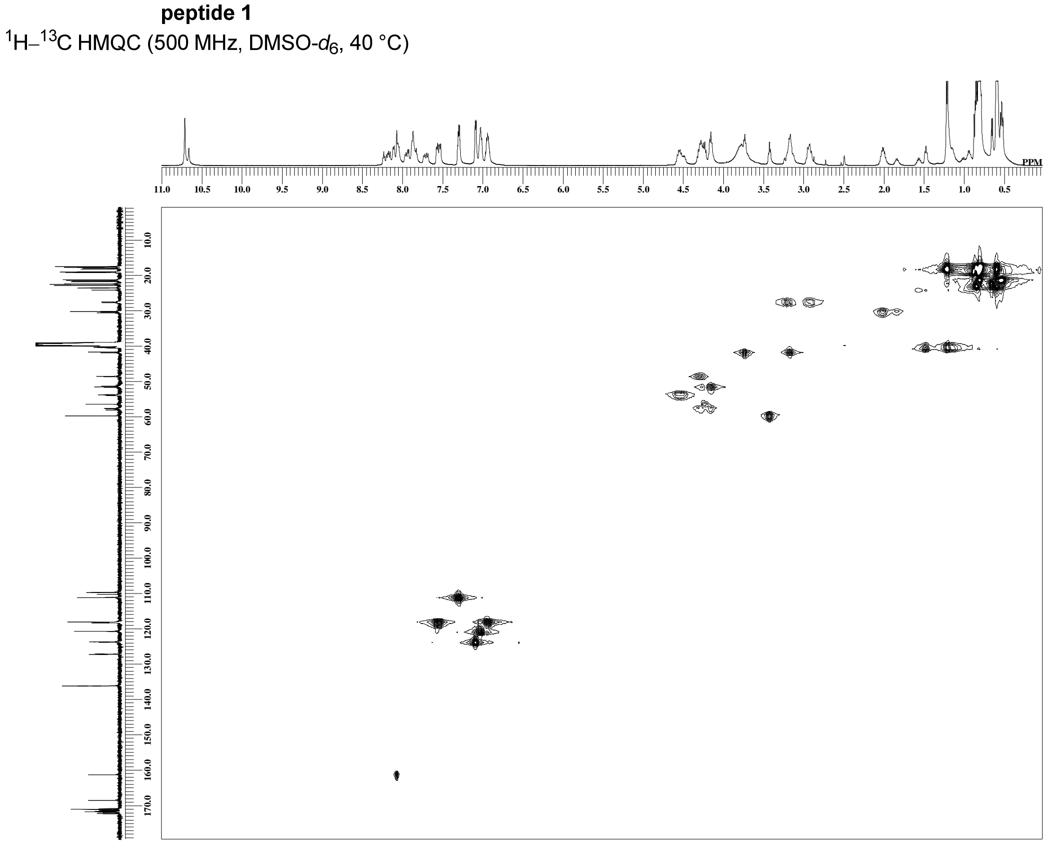

Supplementary Figure 22. ^{1}H–^{13}C HMQC spectrum of **1**. The spectrum was obtained in DMSO-d_6 at 40 °C.

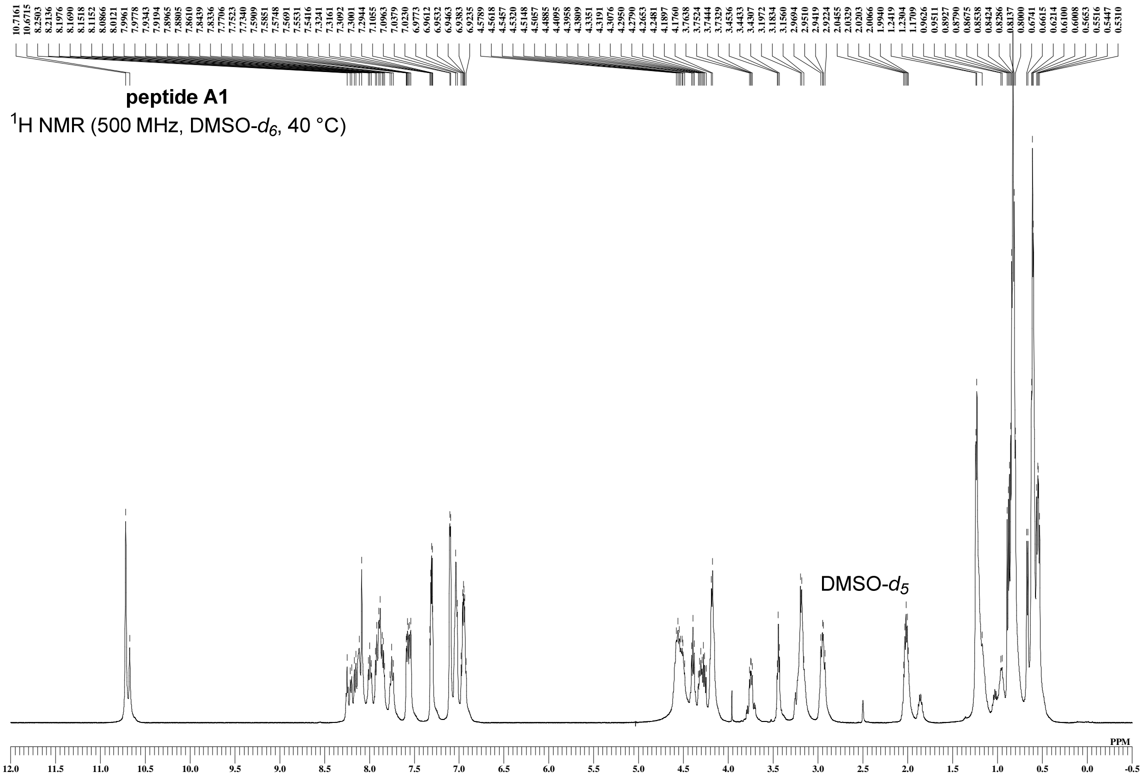

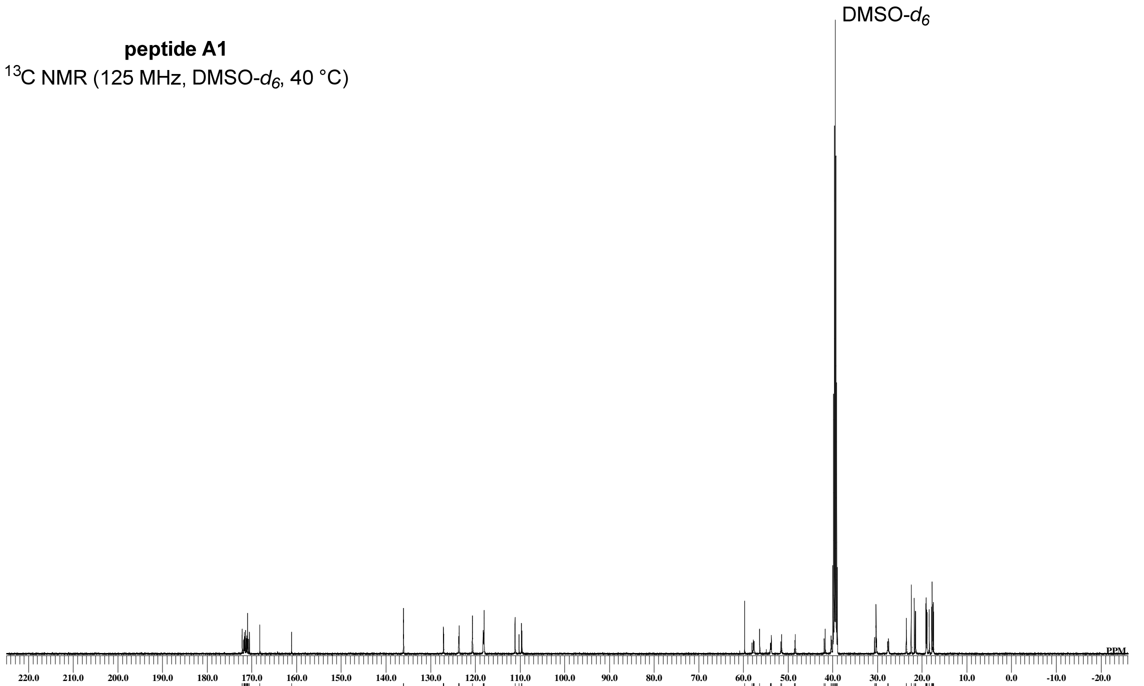

Supplementary Figure 23. ^{1}H and ^{13}C NMR spectra of **A1**. The spectra were obtained in DMSO-d_6 at 40 °C.

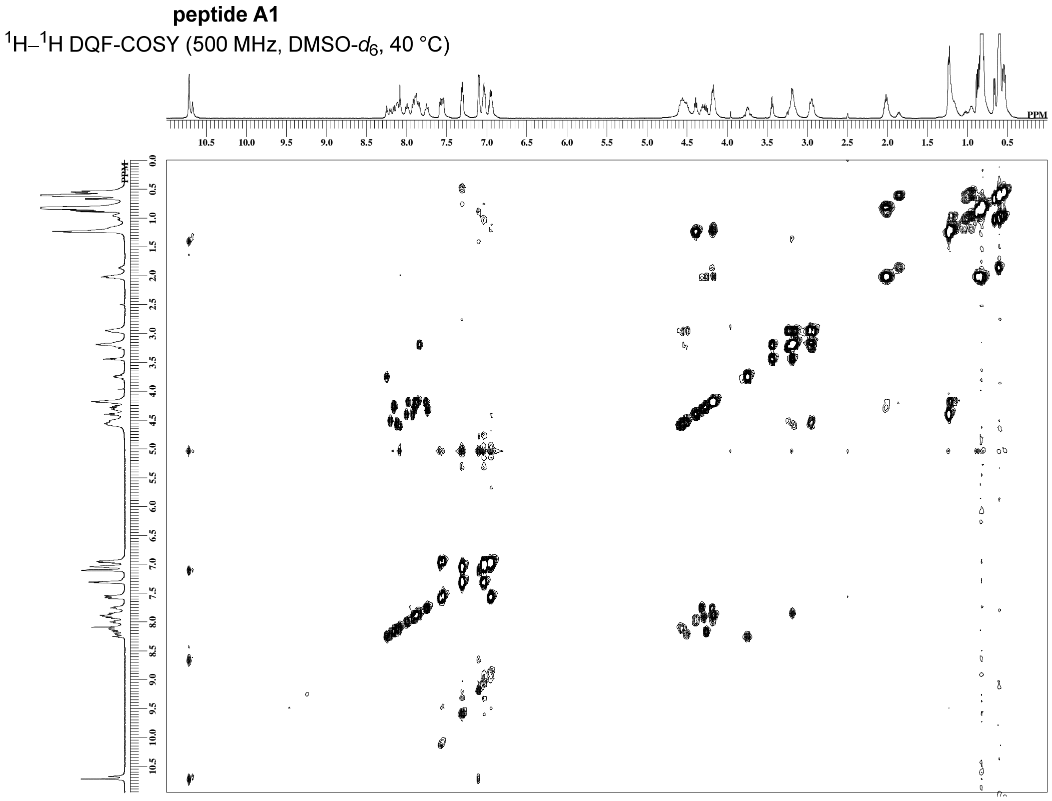

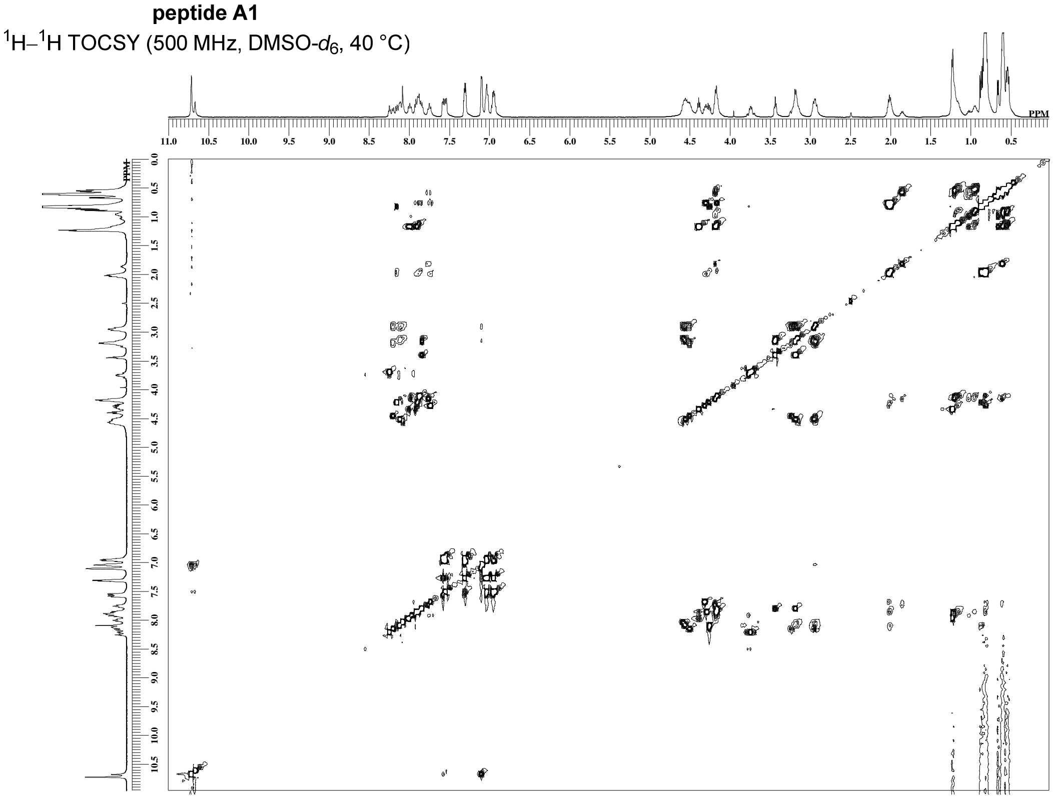

Supplementary Figure 24. ^{1}H–^{1}H DQF-COSY and ^{1}H–^{1}H TOCSY spectra of **A1**. The spectra were obtained in DMSO-d_6 at 40 °C.

peptide A1

^{1}H–^{1}H NOESY (500 MHz, DMSO-d_6, 40 °C)

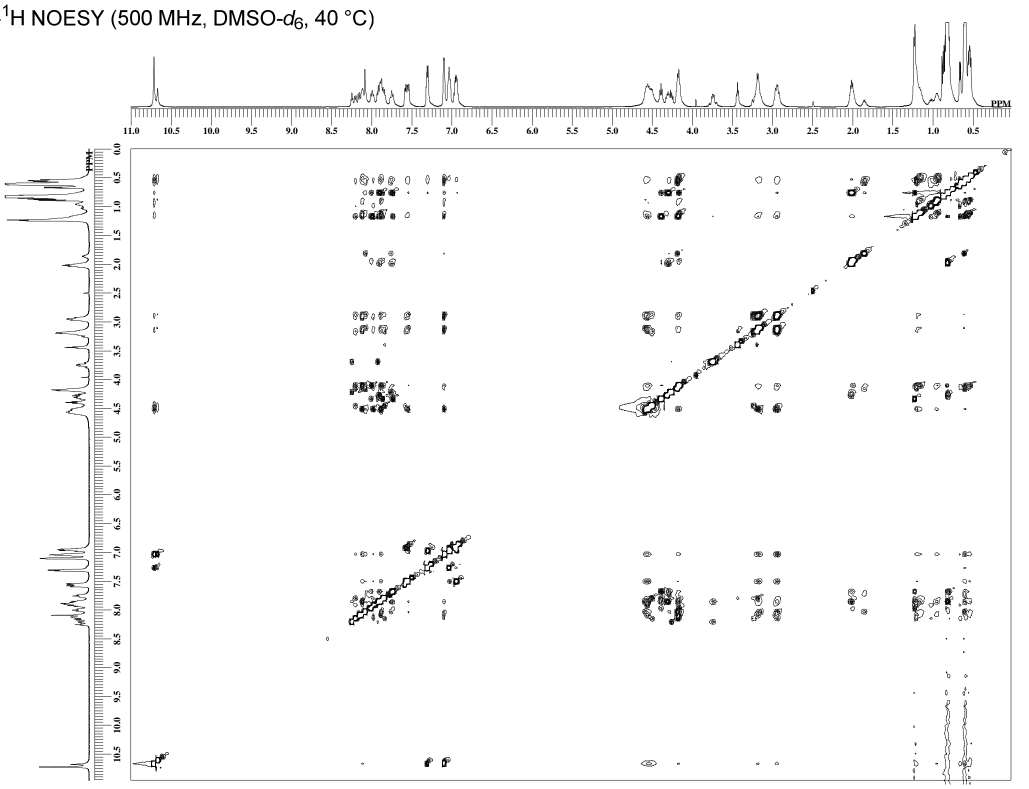

peptide A1

^{1}H–^{13}C HMBC (500 MHz, DMSO-d_6, 40 °C)

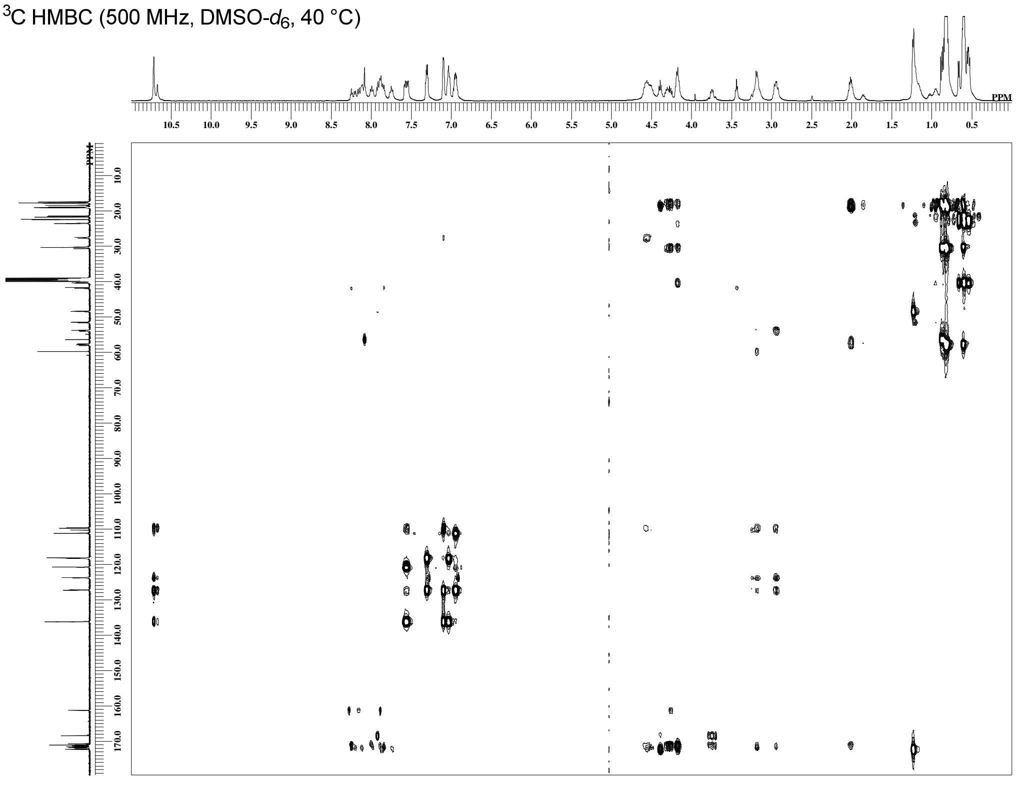

Supplementary Figure 25. ^{1}H–^{1}H NOESY and ^{1}H–^{13}C HMBC spectra of **A1**. The spectra were obtained in DMSO-d_6 at 40 °C.

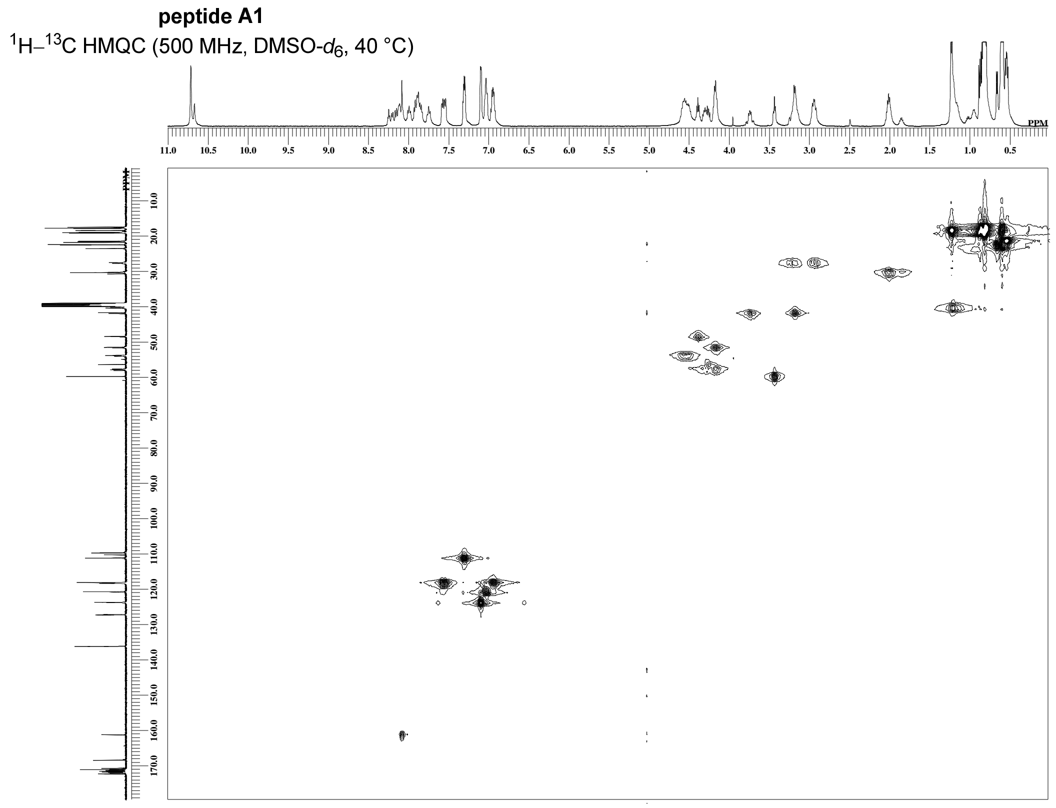

Supplementary Figure 26. ^{1}H–^{13}C HMQC spectrum of **A1**. The spectra were obtained in DMSO-d_6 at 40 °C.

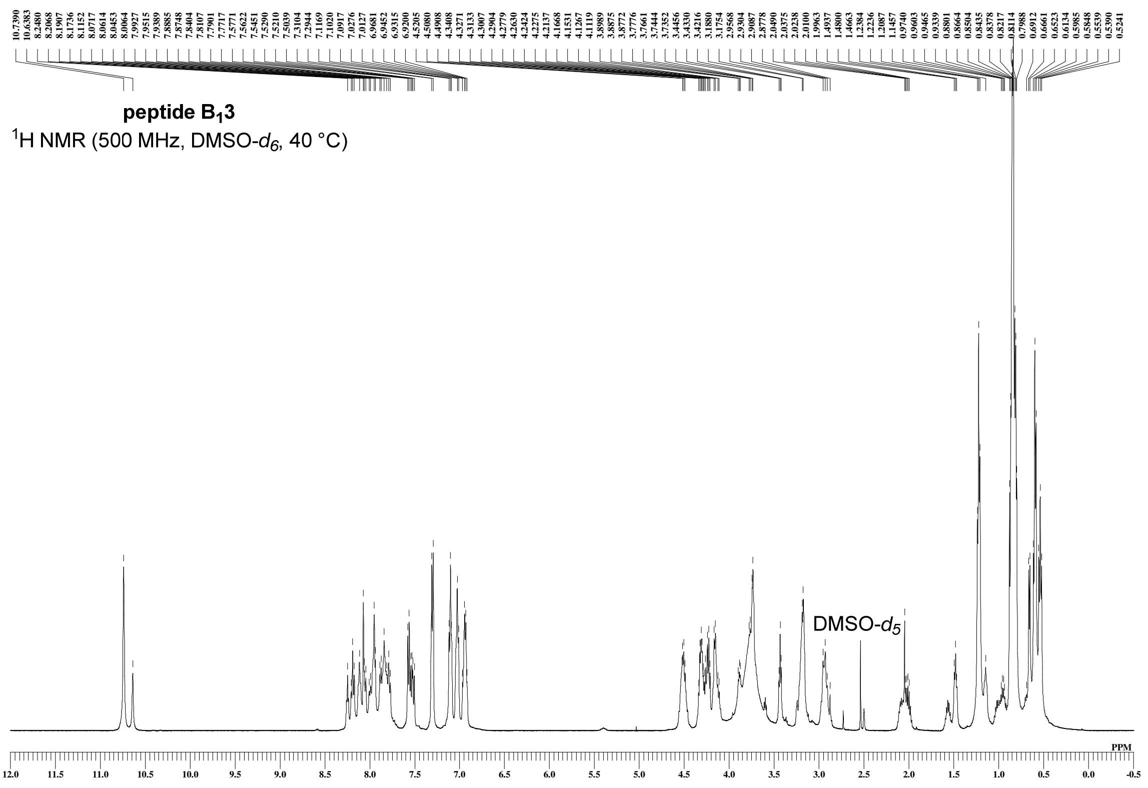

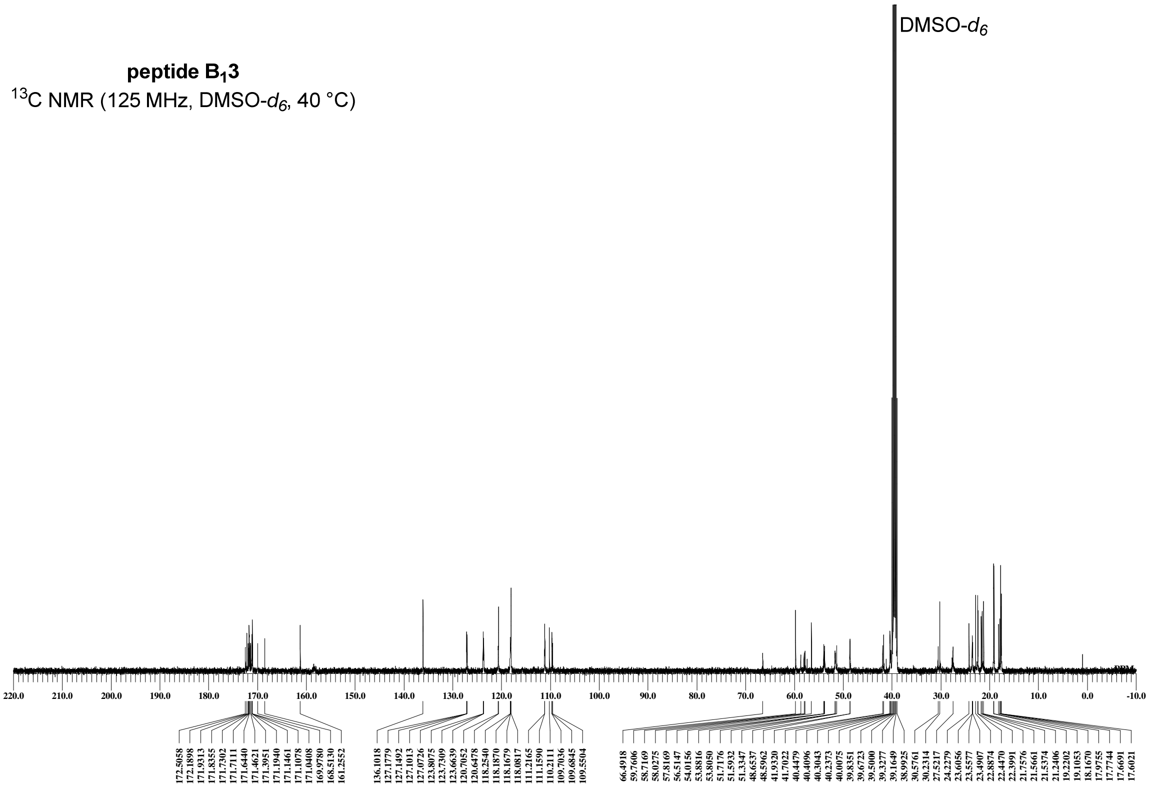

Supplementary Figure 27. ^{1}H and ^{13}C NMR spectra of **B$_1$3**. The spectra were obtained in DMSO-*d*$_6$ at 40 °C.

peptide $B_1$3

$^1H–^1H$ DQF-COSY (500 MHz, DMSO-d_6, 40 °C)

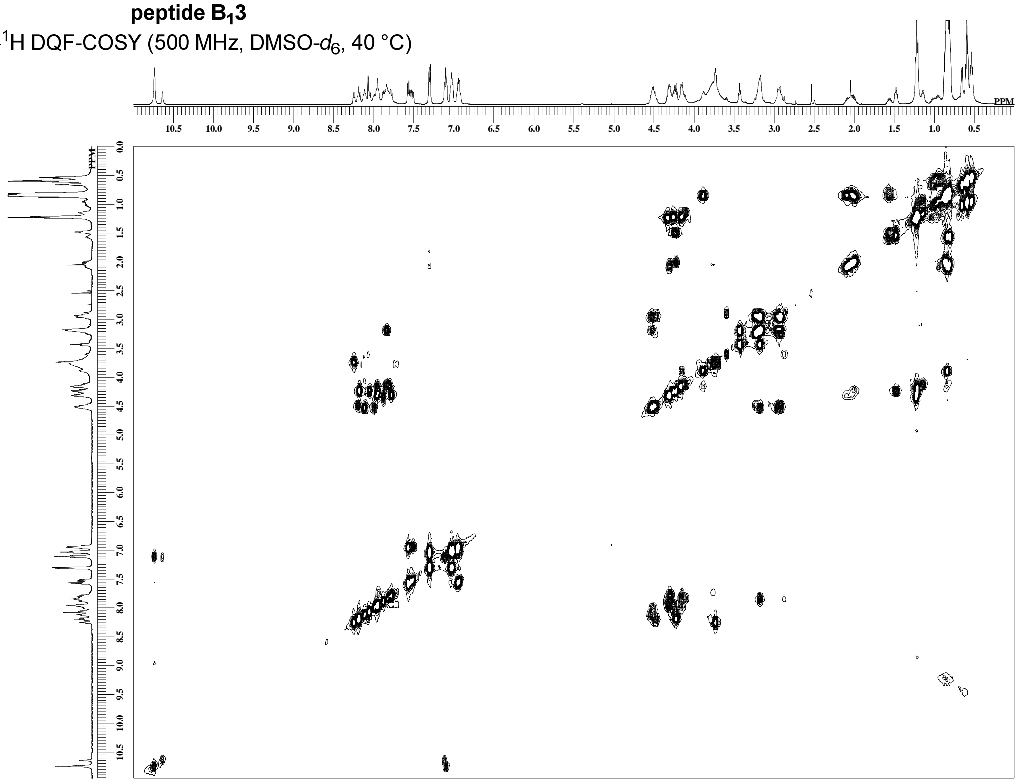

peptide $B_1$3

$^1H–^1H$ TOCSY (500 MHz, DMSO-d_6, 40 °C)

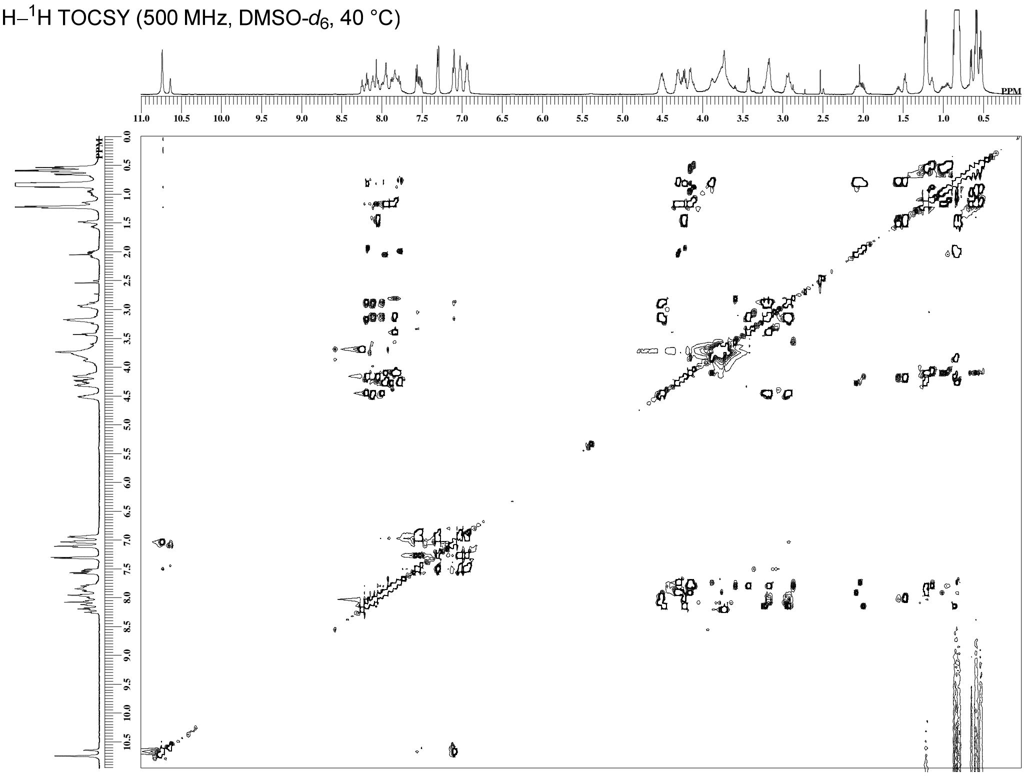

Supplementary Figure 28. $^1H–^1H$ DQF-COSY and $^1H–^1H$ TOCSY spectra of **$B_1$3**. The spectra were obtained in DMSO-d_6 at 40 °C.

peptide B$_1$3

^{1}H–^{1}H NOESY (500 MHz, DMSO-d_6, 40 °C)

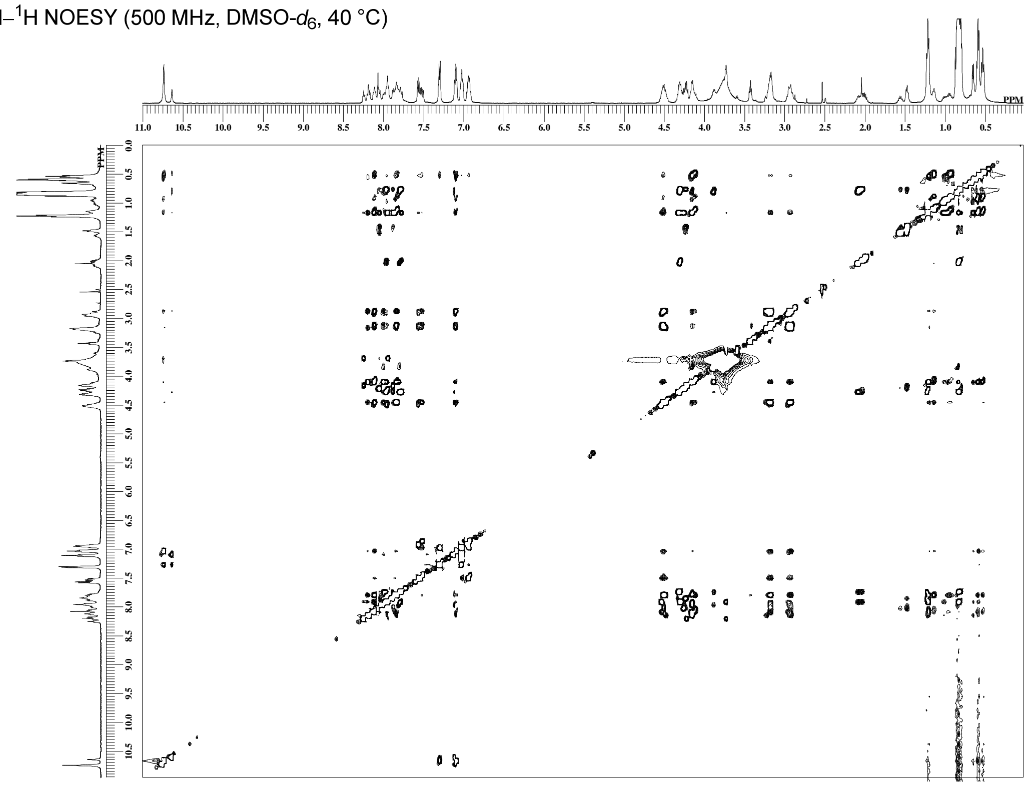

peptide B$_1$3

^{1}H–^{13}C HMBC (500 MHz, DMSO-d_6, 40 °C)

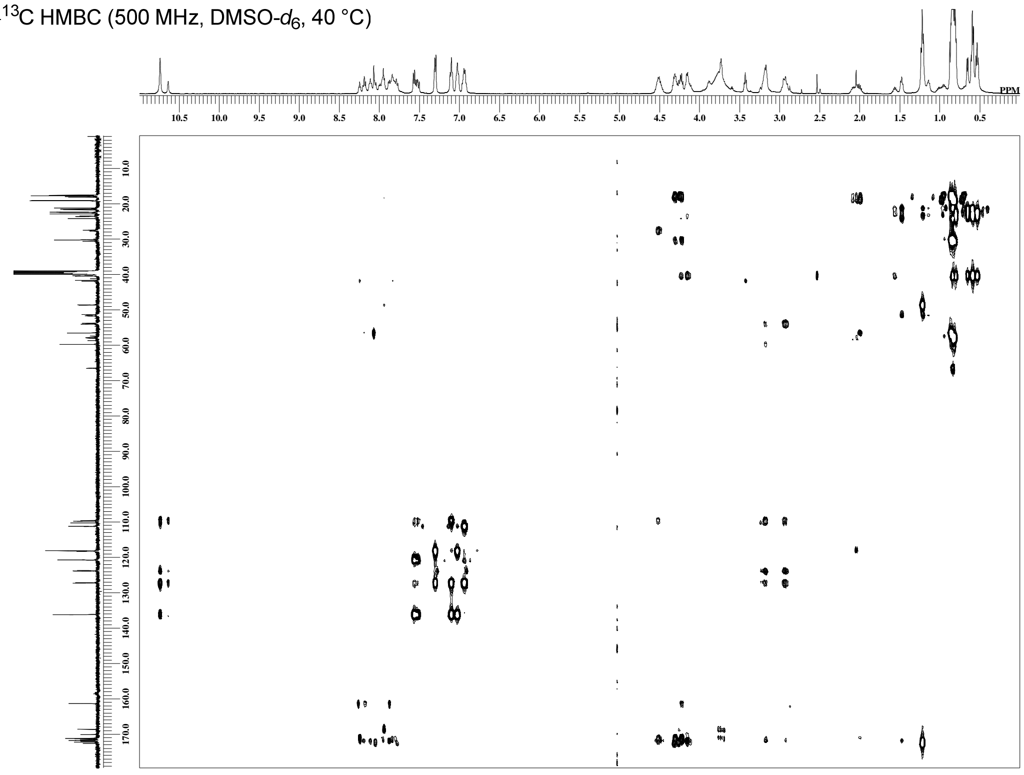

Supplementary Figure 29. ^{1}H–^{1}H NOESY and ^{1}H–^{13}C HMBC spectra of **B$_1$3**. The spectra were obtained in DMSO-d_6 at 40 °C.

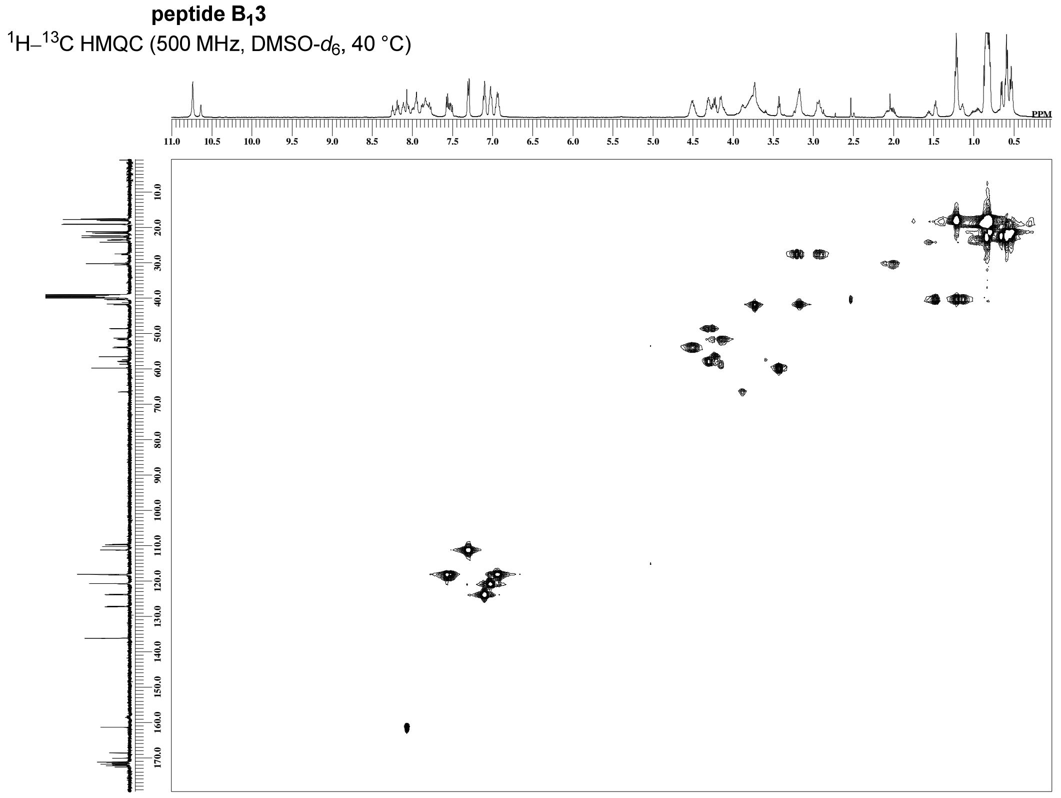

Supplementary Figure 30. ^{1}H–^{13}C HMQC spectrum of **B$_1$3**. The spectrum was obtained in DMSO-d_6 at 40 °C.

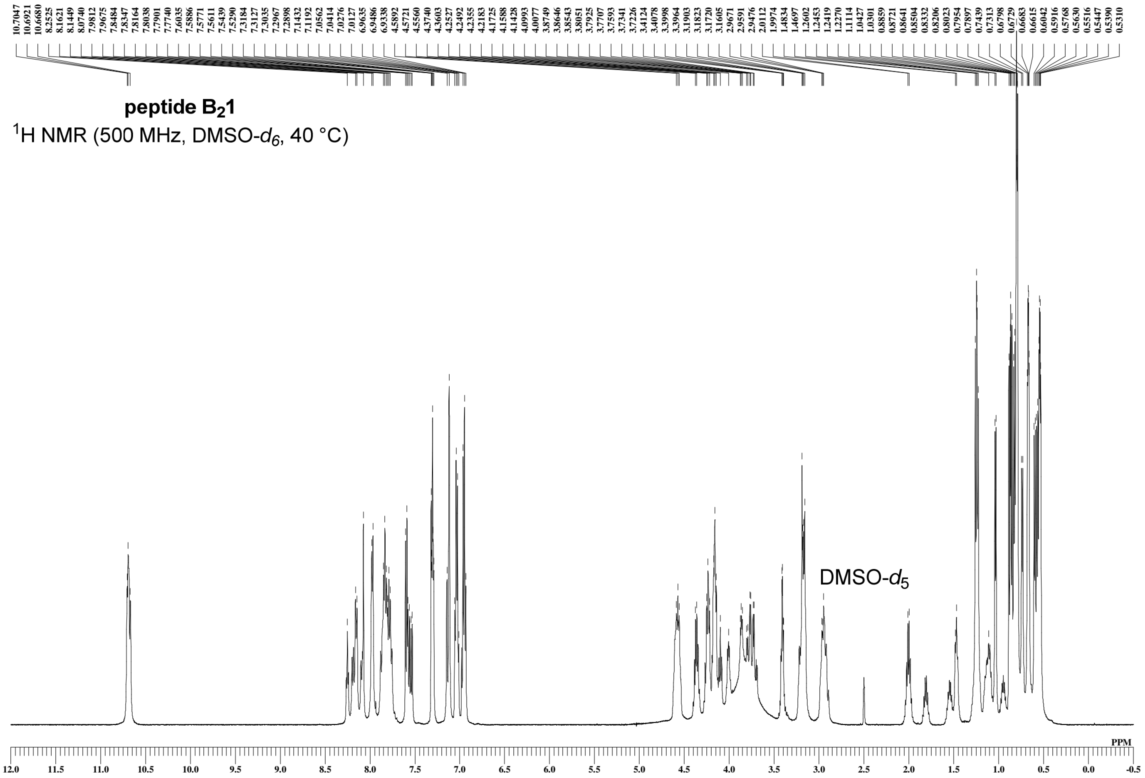

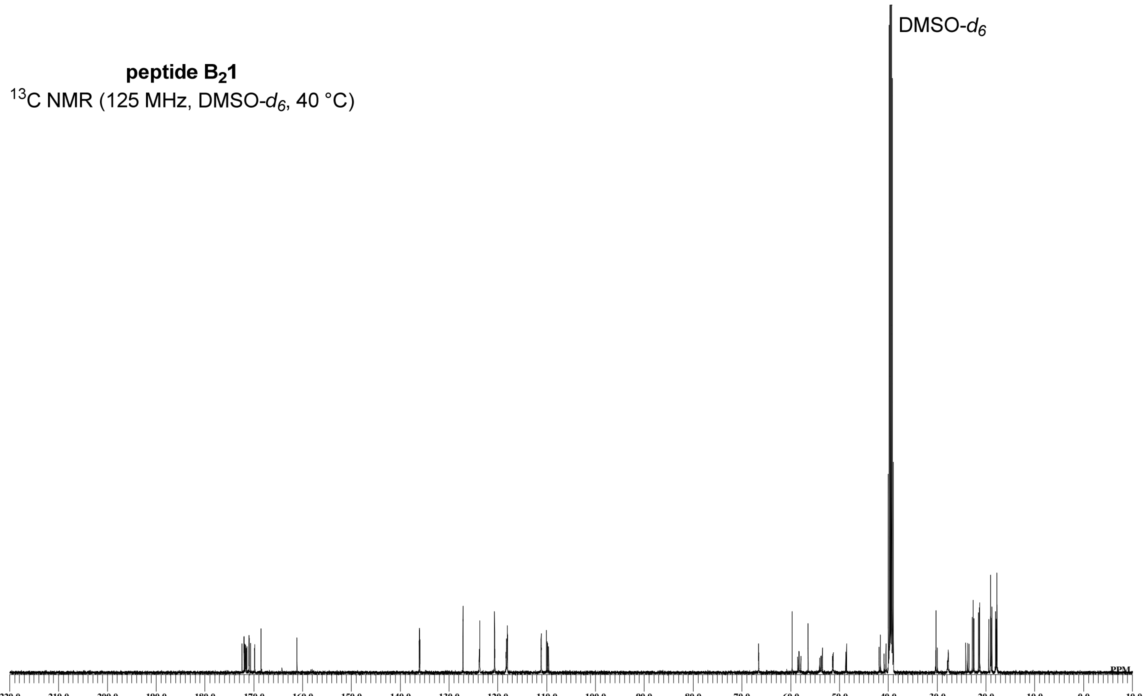

Supplementary Figure 31. ^{1}H and ^{13}C NMR spectra of **$B_2$1**. The spectra were obtained in DMSO-d_6 at 40 °C.

peptide $B_2$1
^{1}H–^{1}H DQF-COSY (500 MHz, DMSO-d_6, 40 °C)

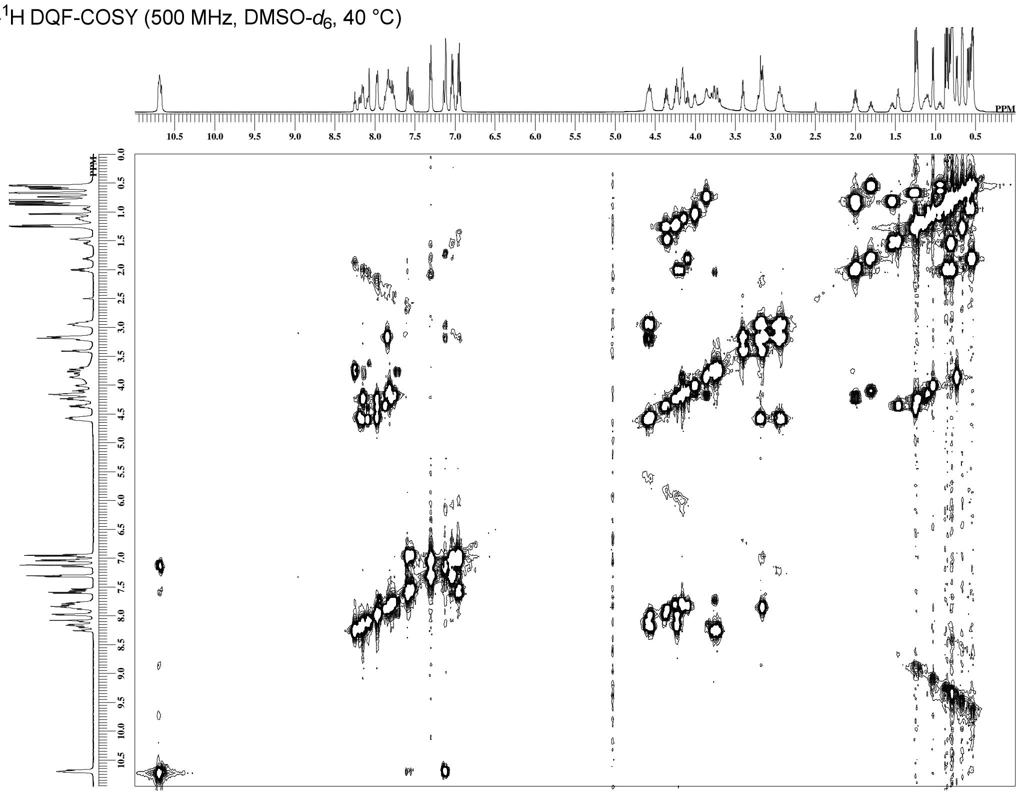

peptide $B_2$1
^{1}H–^{1}H TOCSY (500 MHz, DMSO-d_6, 40 °C)

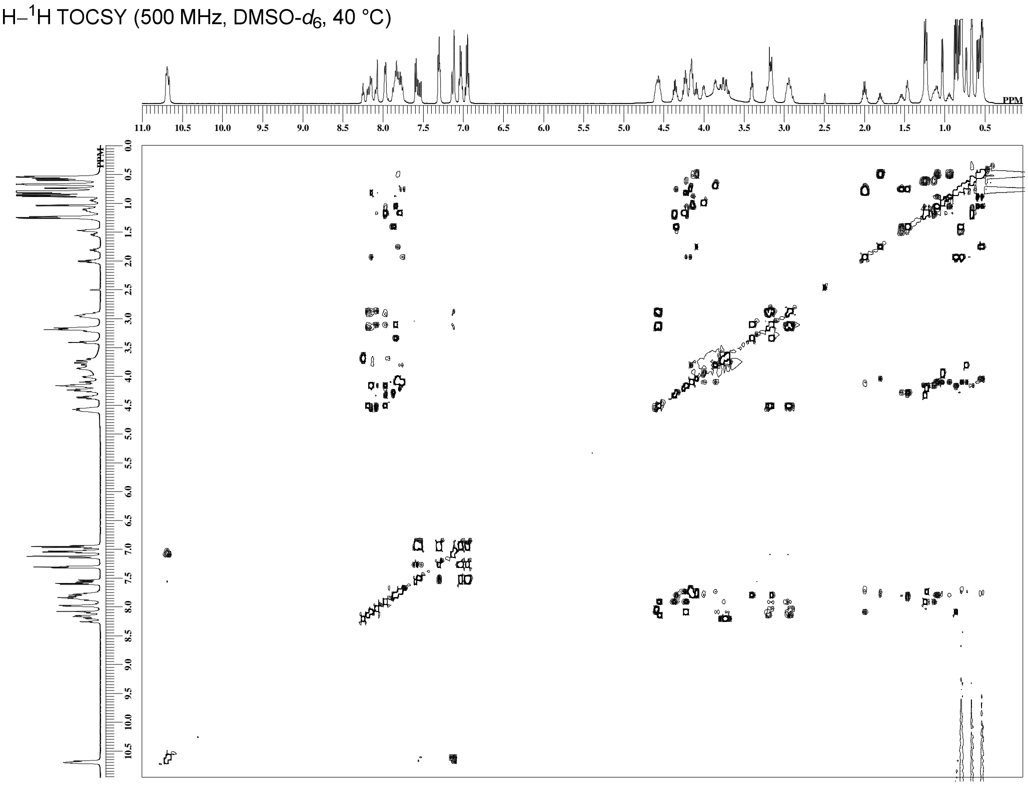

Supplementary Figure 32. ^{1}H–^{1}H DQF-COSY and ^{1}H–^{1}H TOCSY spectra of **$B_2$1**. The spectra were obtained in DMSO-d_6 at 40 °C.

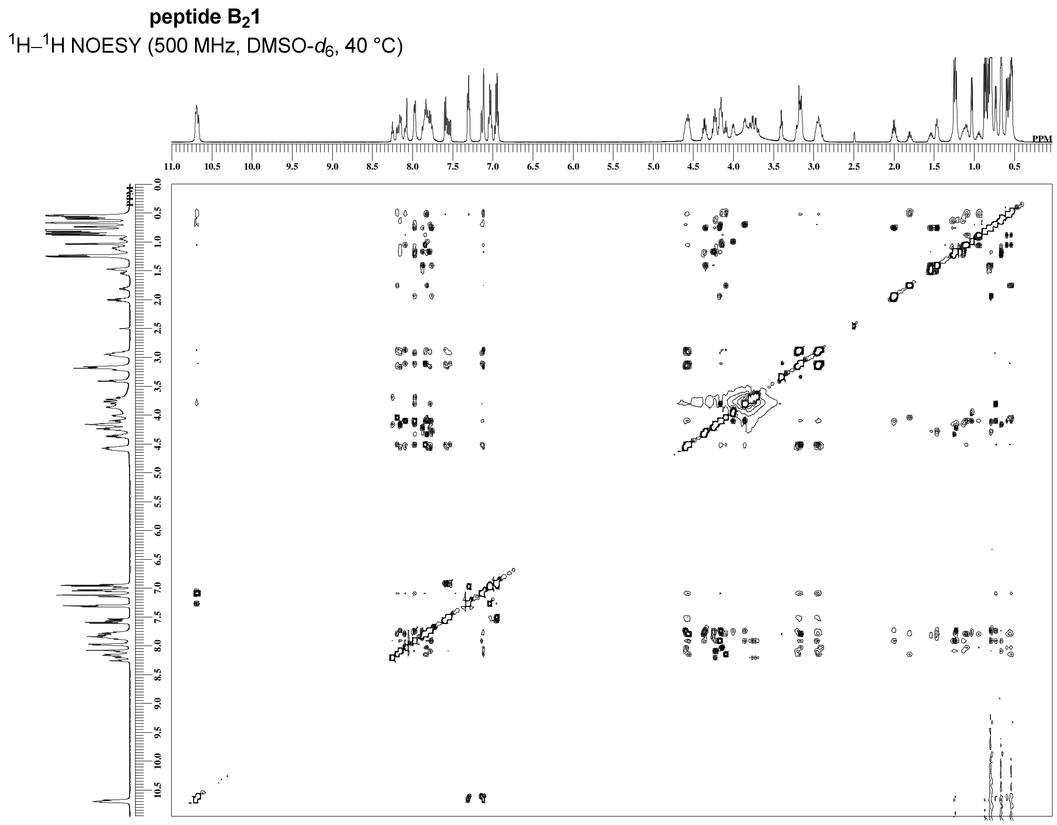

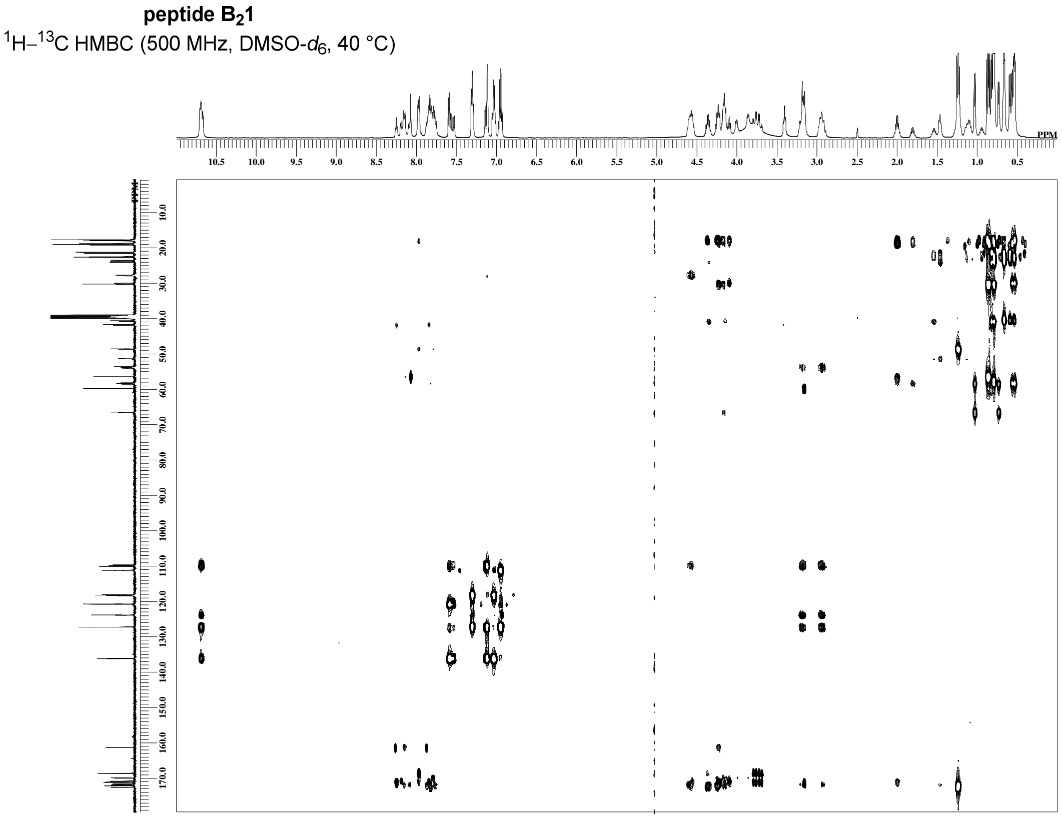

Supplementary Figure 33. ^1H–^1H NOESY and ^1H–^{13}C HMBC spectra of $\mathbf{B_21}$. The spectra were obtained in DMSO-d_6 at 40 °C.

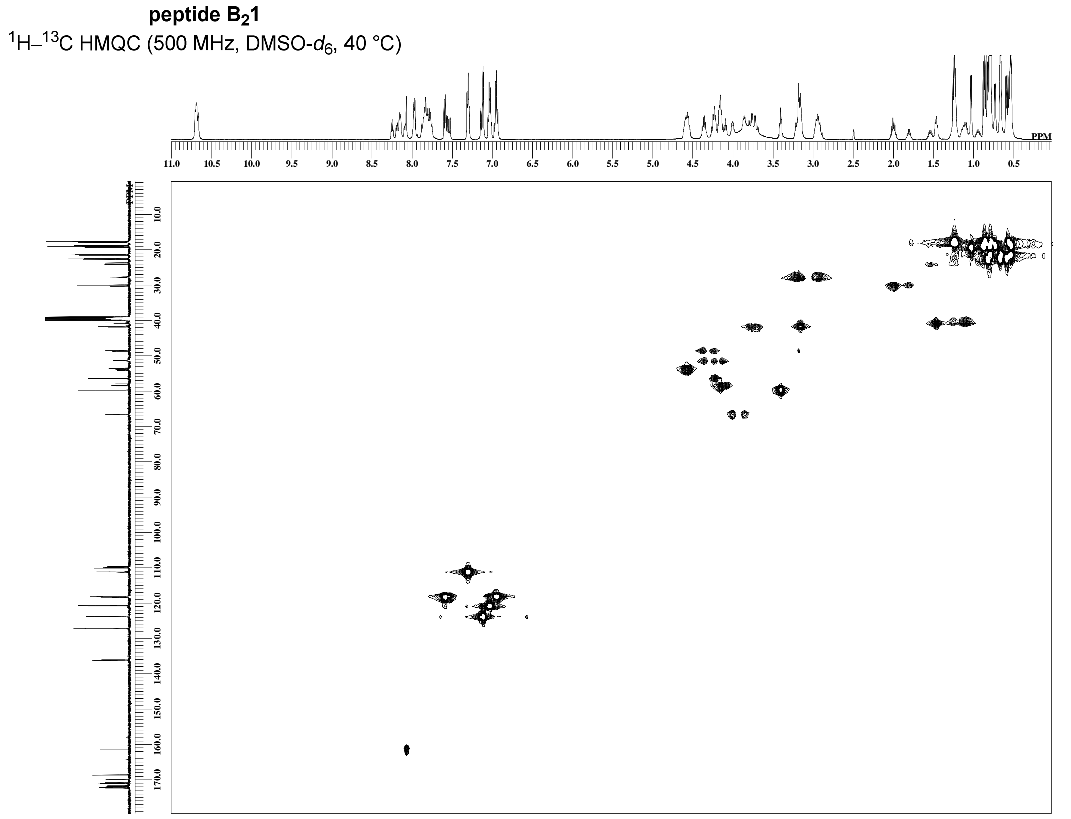

Supplementary Figure 34. ${}^{1}H$–${}^{13}C$ HMQC spectrum of **B₂1**. The spectrum was obtained in DMSO-d_6 at 40 °C.

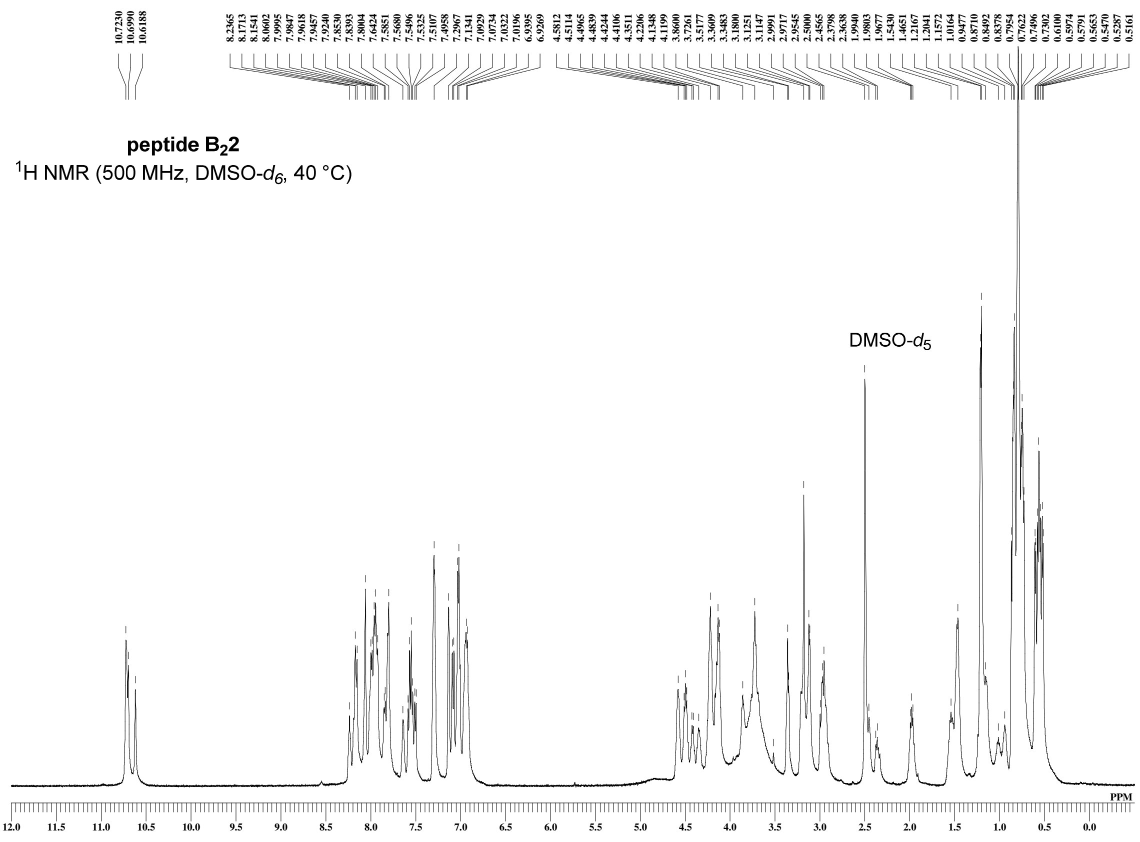

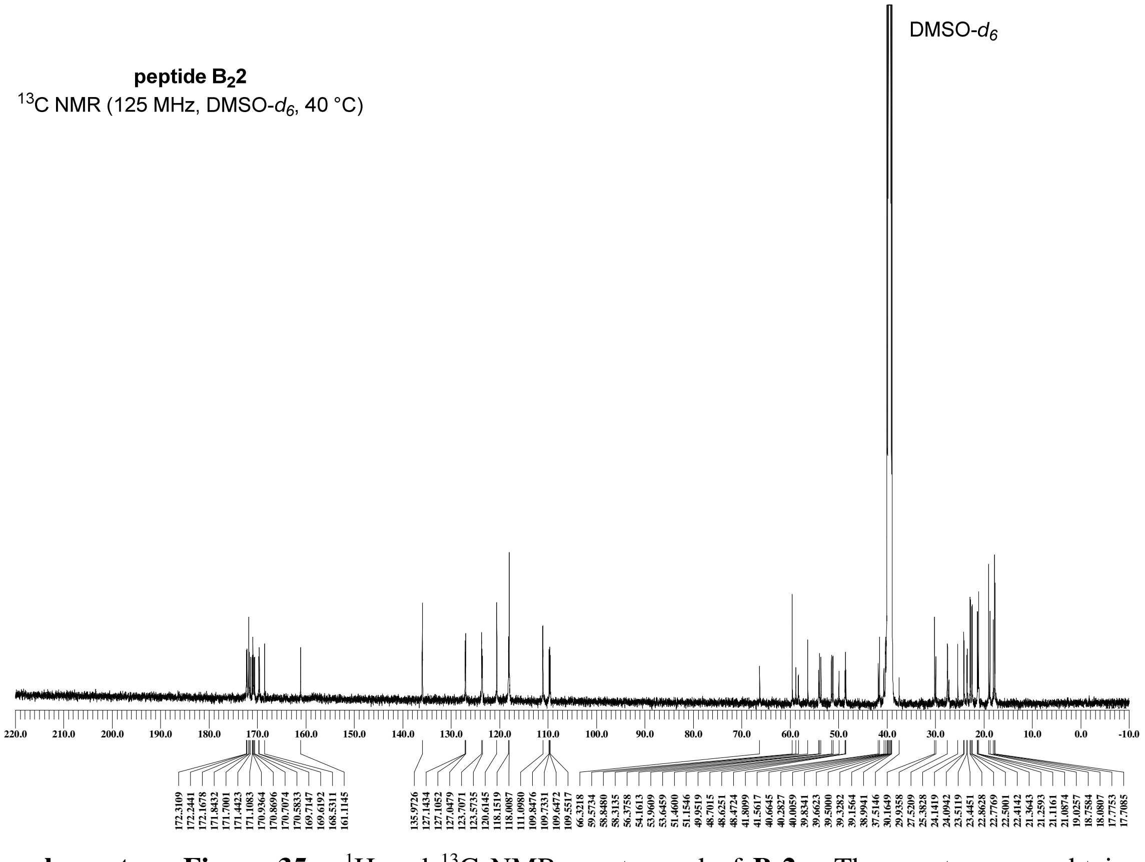

Supplementary Figure 35. ^{1}H and ^{13}C NMR spectra and of **B$_2$2**. The spectra were obtained in DMSO-d_6 at 40 °C.

peptide $B_2$2

^{1}H–^{1}H DQF-COSY (500 MHz, DMSO-d_6, 40 °C)

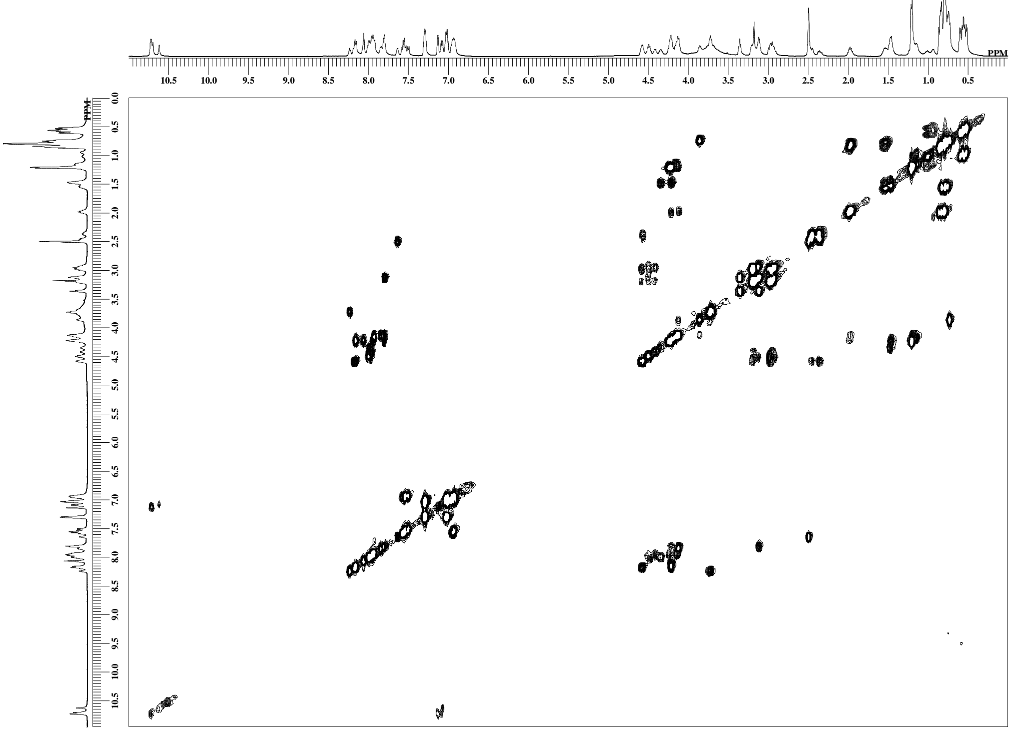

peptide $B_2$2

^{1}H–^{1}H TOCSY (500 MHz, DMSO-d_6, 40 °C)

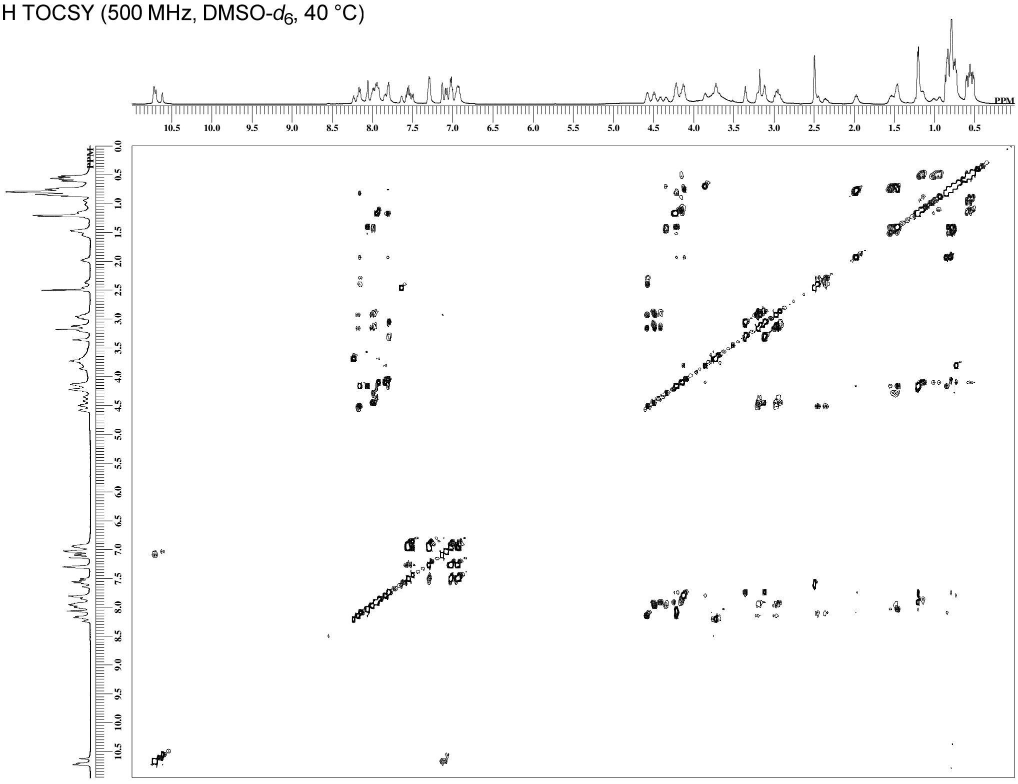

Supplementary Figure 36. ^{1}H–^{1}H DQF-COSY and ^{1}H–^{1}H TOCSY spectra of **$B_2$2**. The spectra were obtained in DMSO-d_6 at 40 °C.

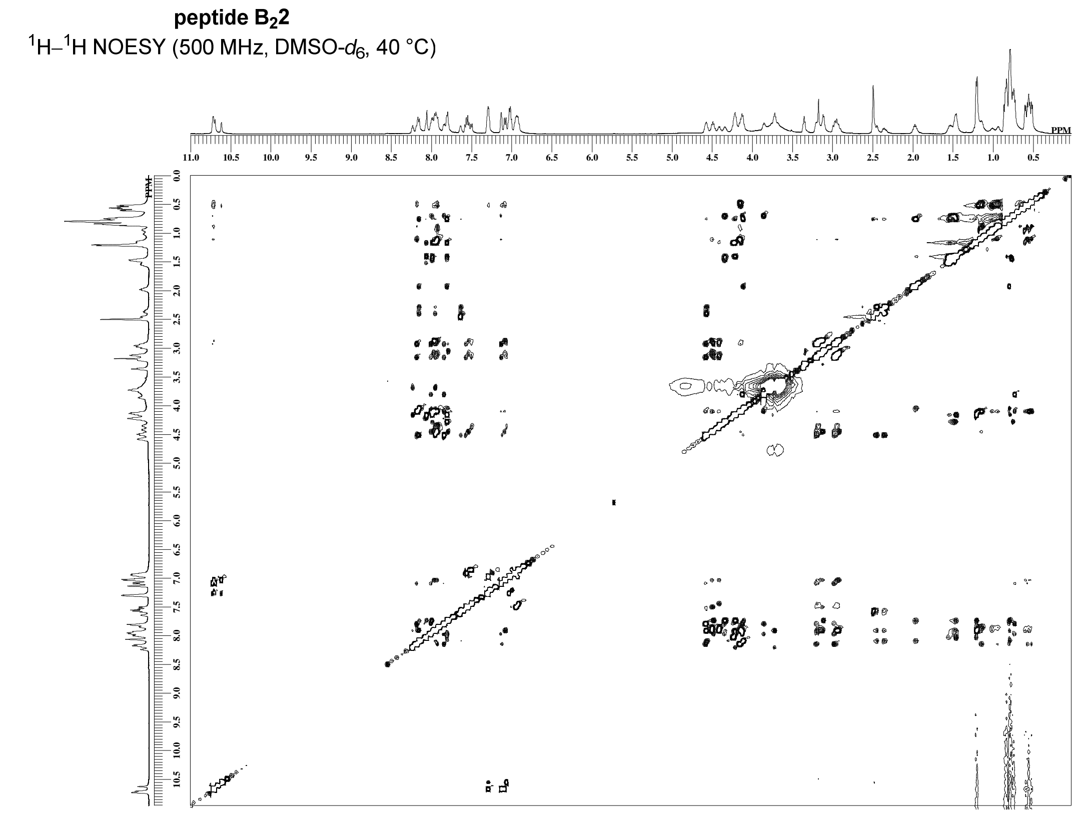

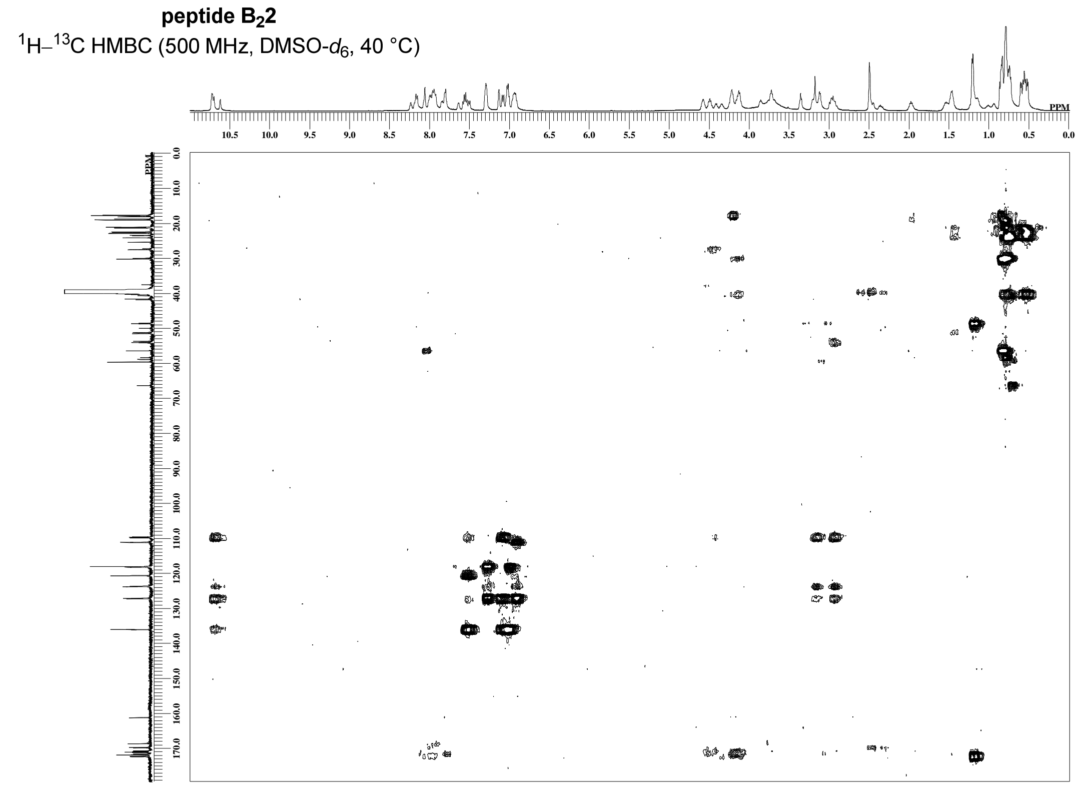

Supplementary Figure 37. ^{1}H–^{1}H NOESY and ^{1}H–^{13}C HMBC spectra of **B$_2$2**. The spectra were obtained in DMSO-d_6 at 40 °C.

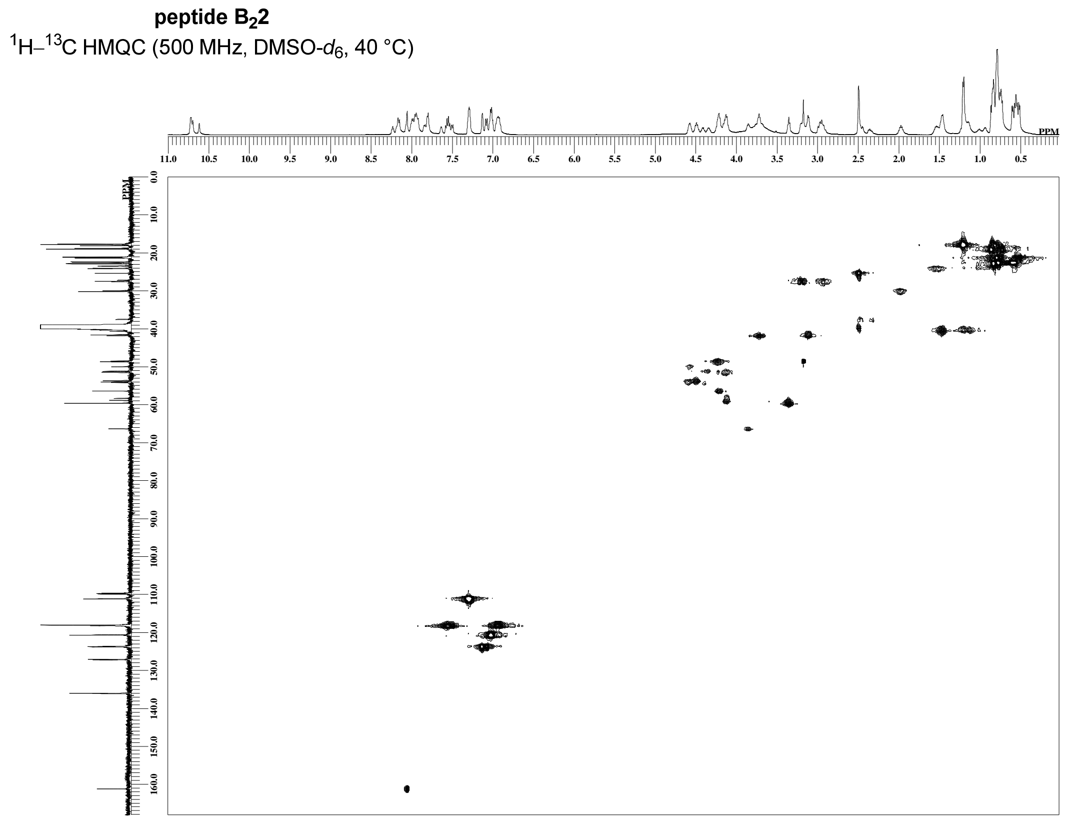

Supplementary Figure 38. $^{1}H–^{13}C$ HMQC spectrum of **$B_2$2.** The spectrum was obtained in DMSO-d_6 at 40 °C.

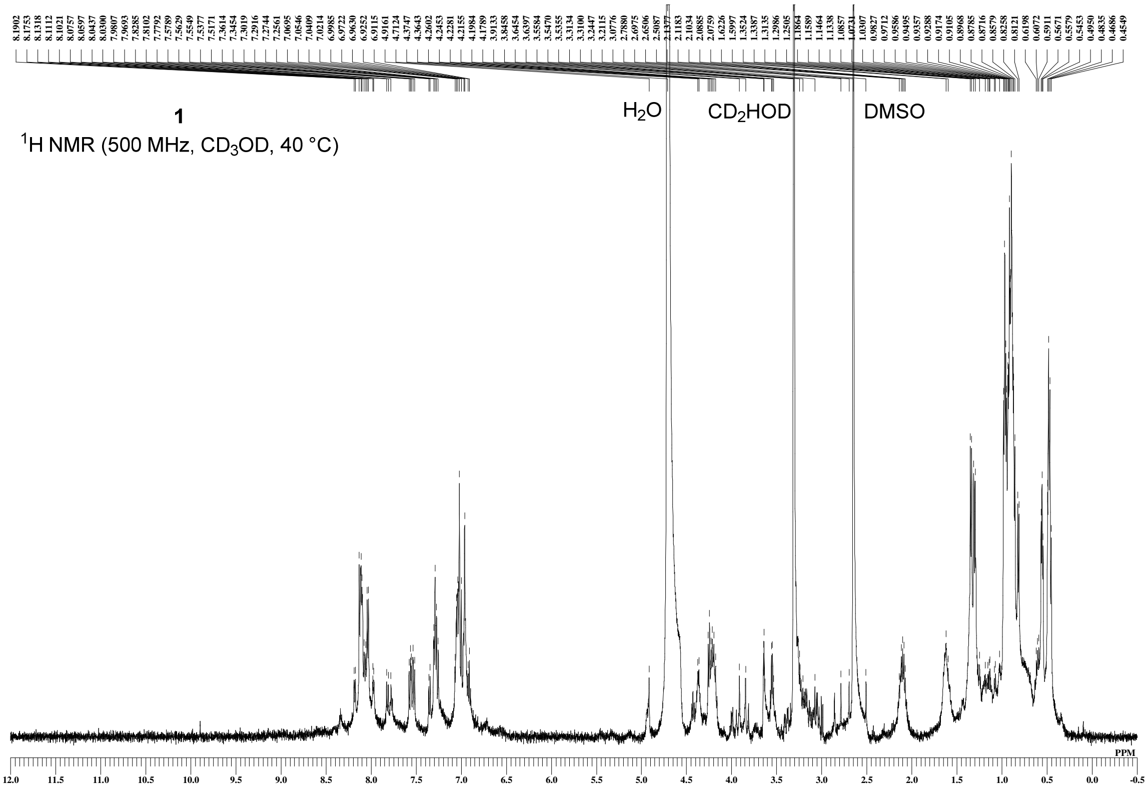

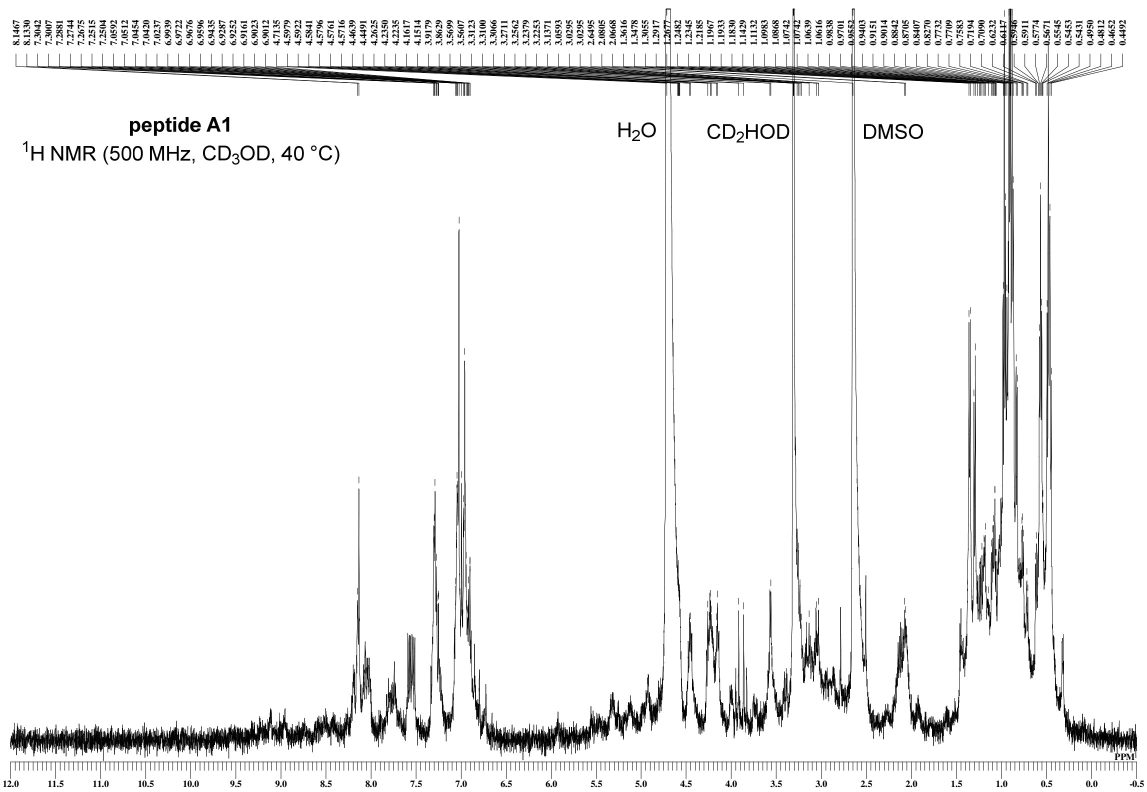

Supplementary Figure 39. ^{1}H NMR spectra of **1** and **A1**. The spectra were obtained in CD_3OD at 40 °C.

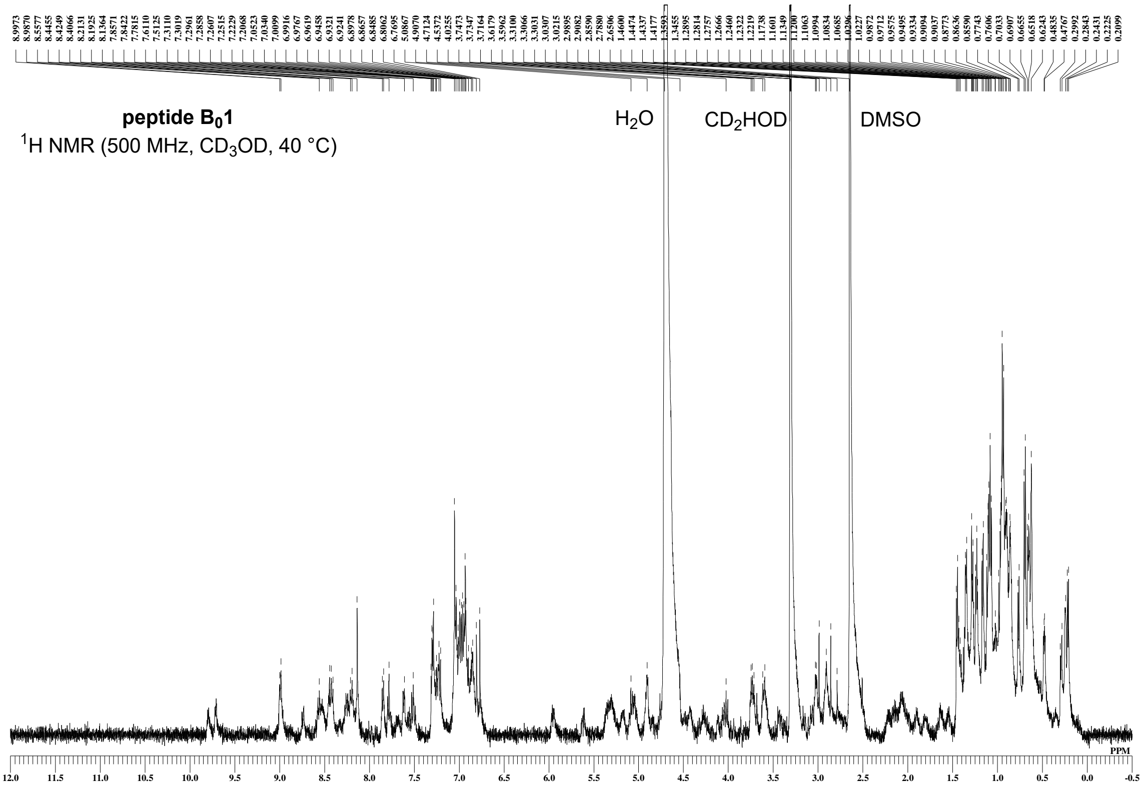

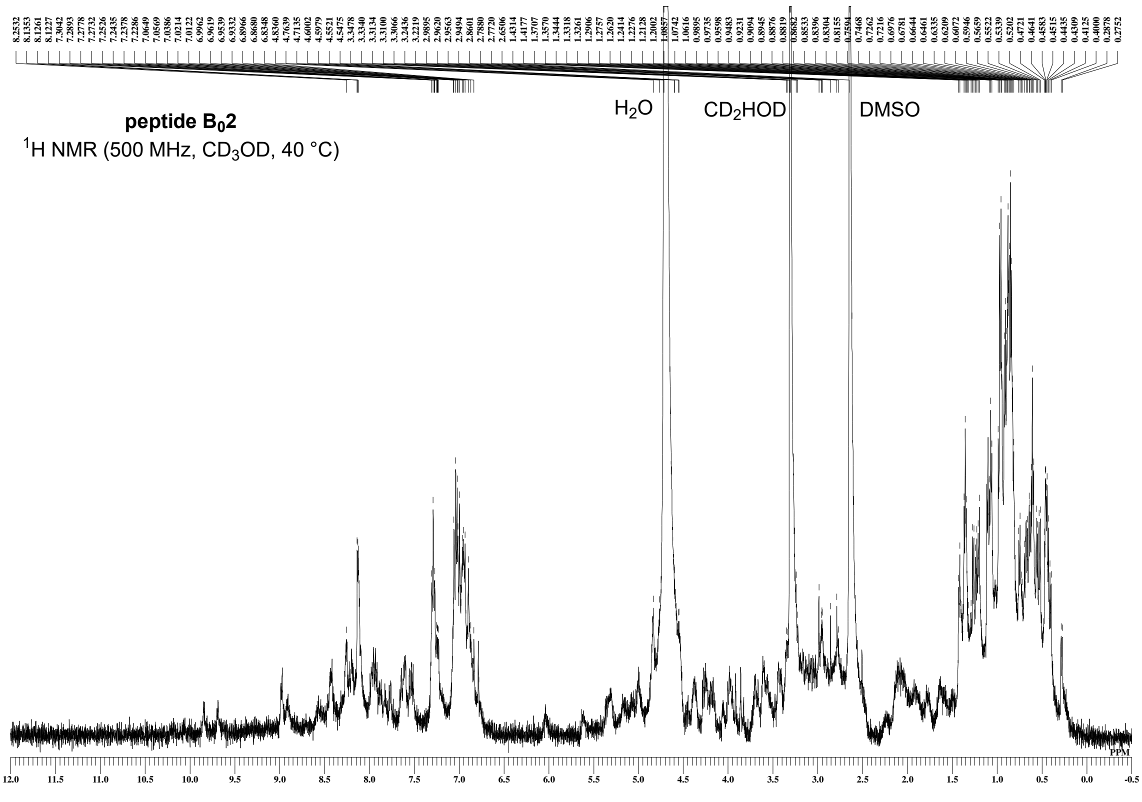

Supplementary Figure 40. ^{1}H NMR spectra and of **B$_0$1** and **B$_0$2**. The spectra were obtained in CD_3OD at 40 °C.

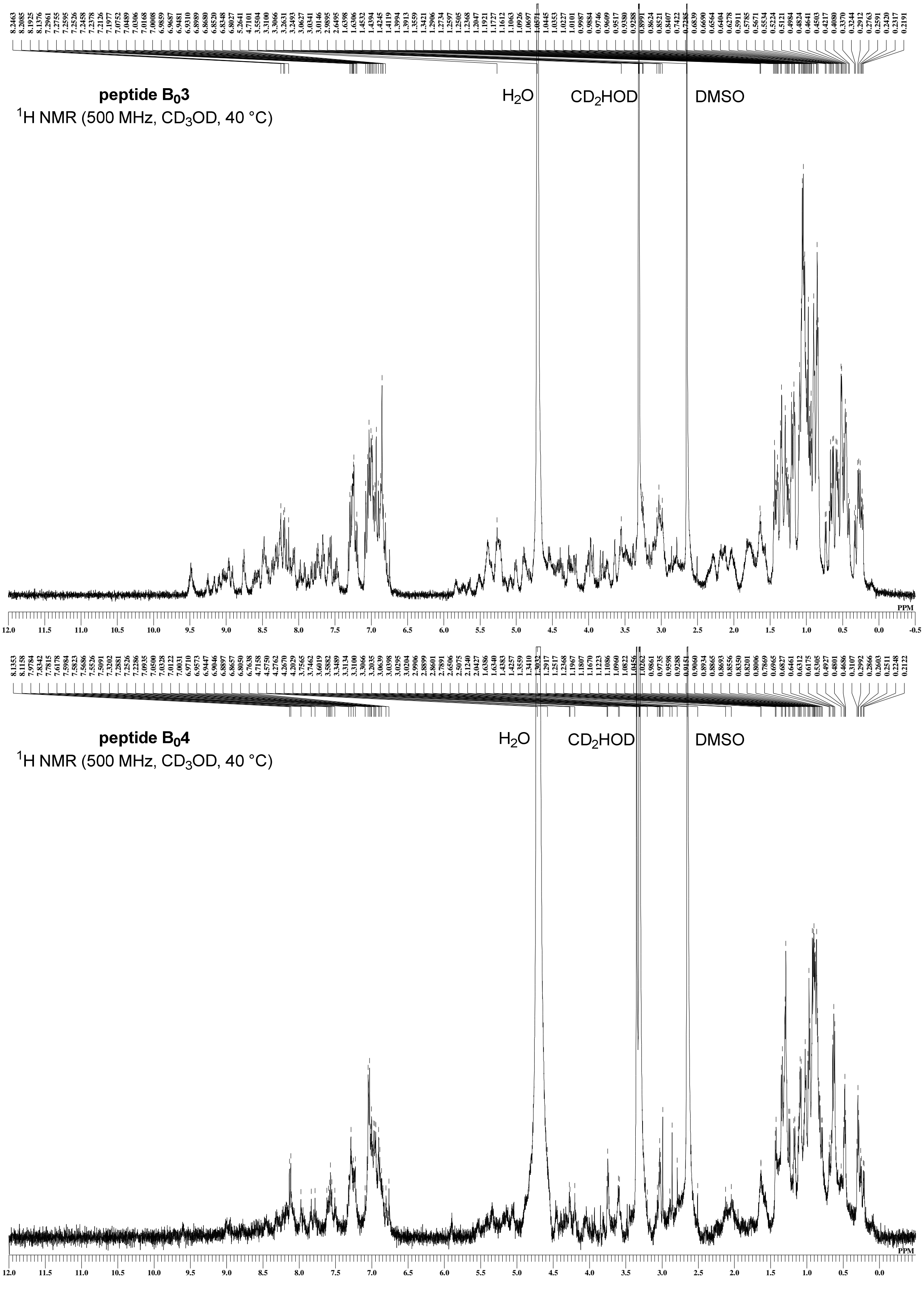

Supplementary Figure 41. ^1H NMR spectra and of $\mathbf{B_03}$ and $\mathbf{B_04}$. The spectra were obtained in CD_3OD at 40 °C.

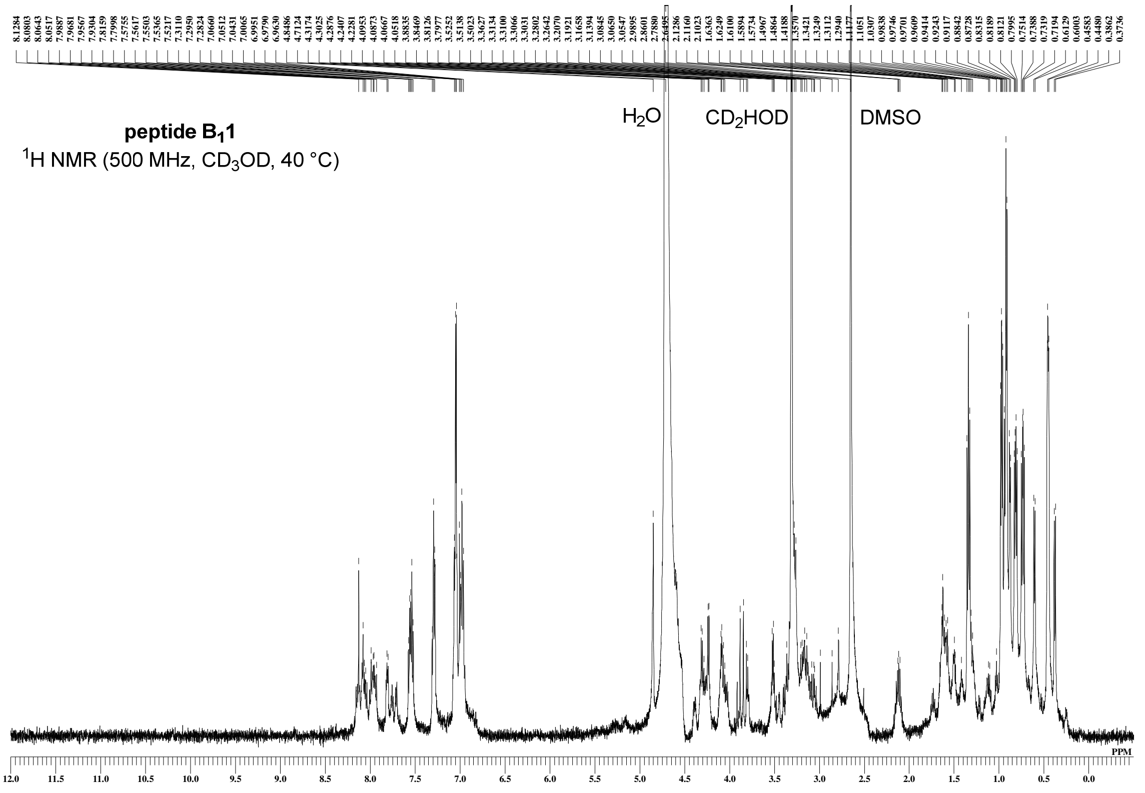

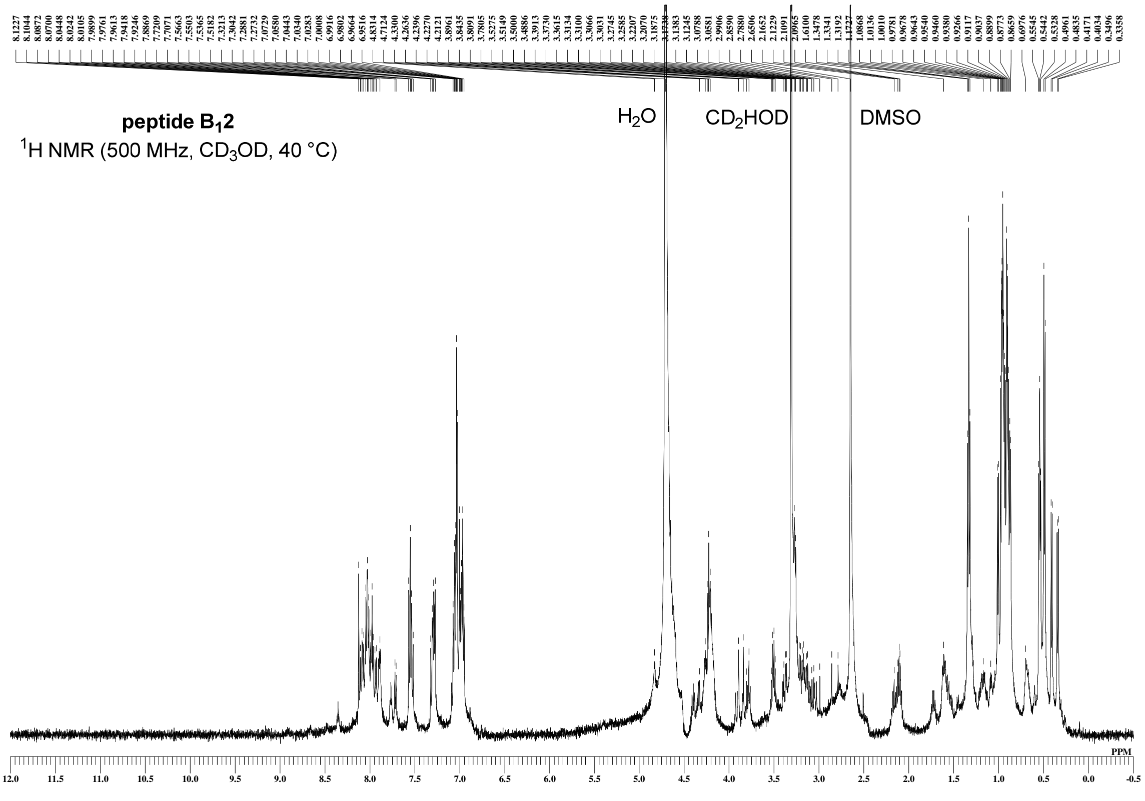

Supplementary Figure 42. ^{1}H NMR spectra of **B$_1$1** and **B$_1$2**. The spectra were obtained in CD_3OD at 40 °C.

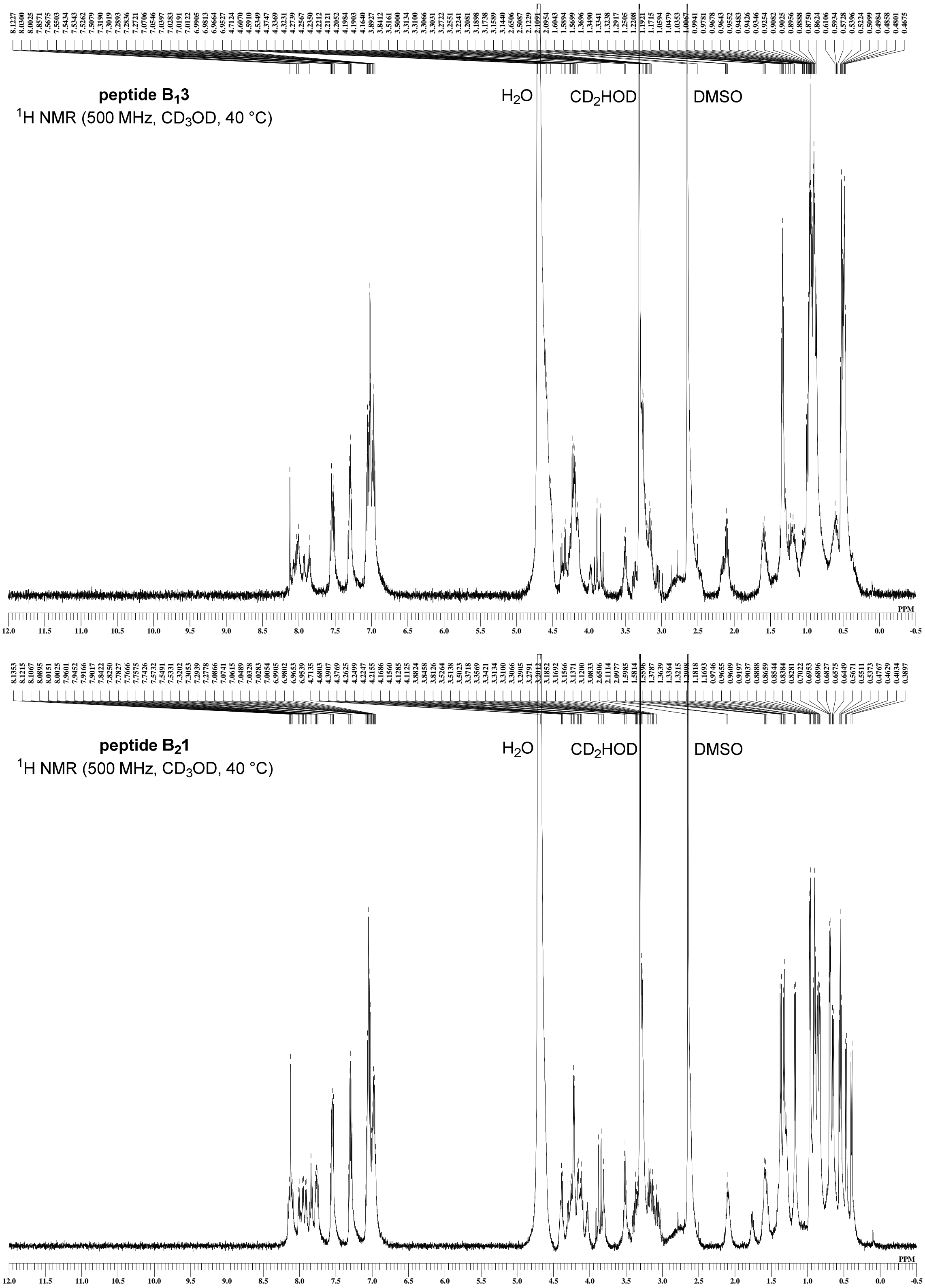

Supplementary Figure 43 ^{1}H NMR spectra of **B₁3** and **B₂1**. The spectra were obtained in CD_3OD at 40 °C.

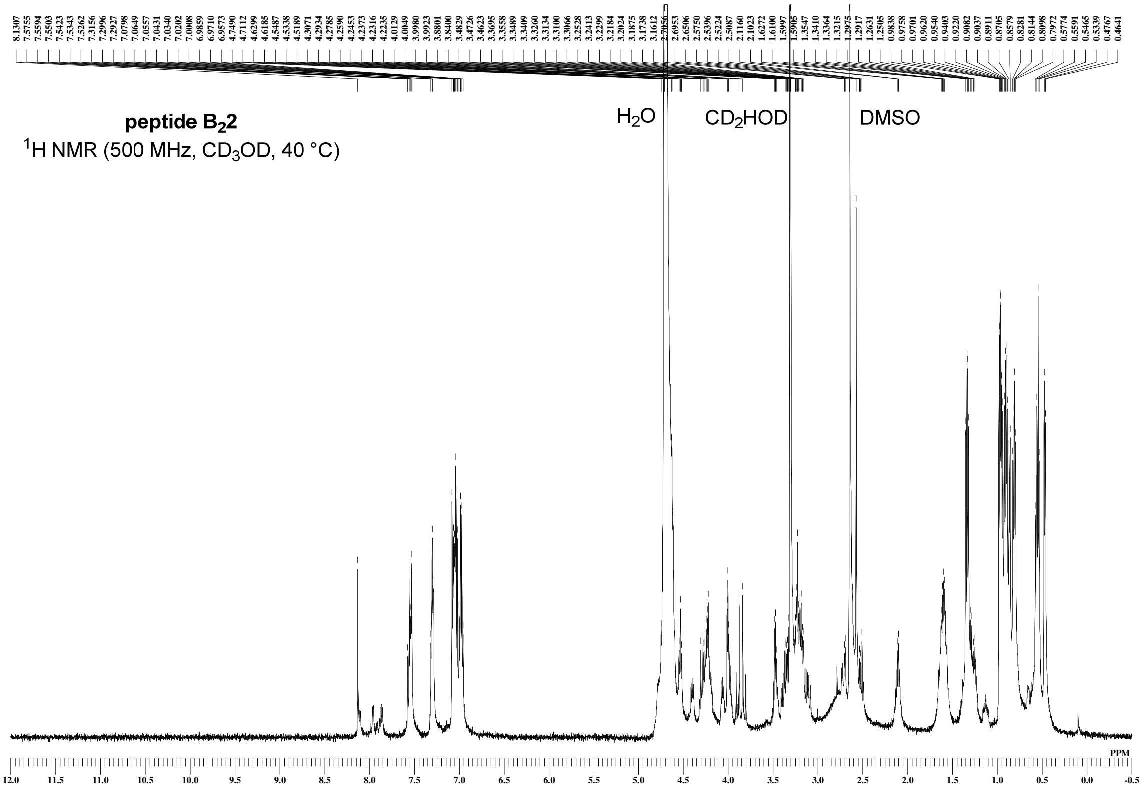

Supplementary Figure 44. ^1H NMR spectrum of **$B_2$2**. The spectrum was obtained in CD_3OD at 40 °C.

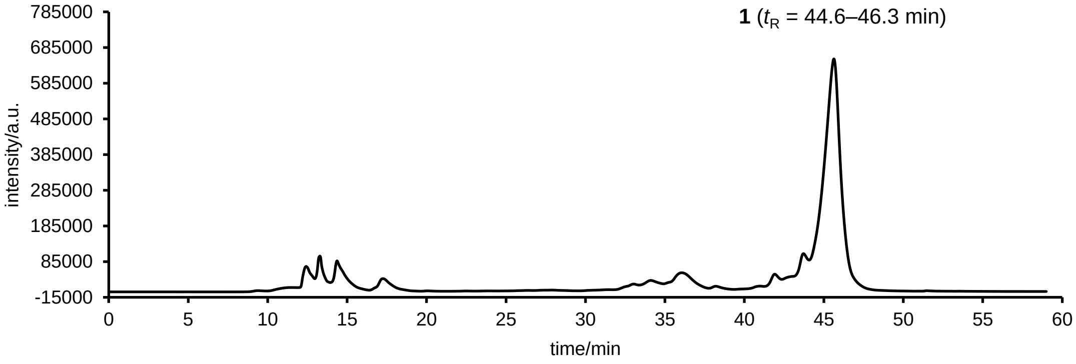

Supplementary Figure 45. HPLC chart for HPLC purification of **1**. Column: Inertsil C8-3 20 × 250 mm, eluent A: MeOH + 0.05% TFA, eluent B: H_2O + 0.05% TFA, linear gradient A/B = 60/40 to 100/0 over 40 min, flow rate: 5.0 mL/min, detection: UV 280 nm.

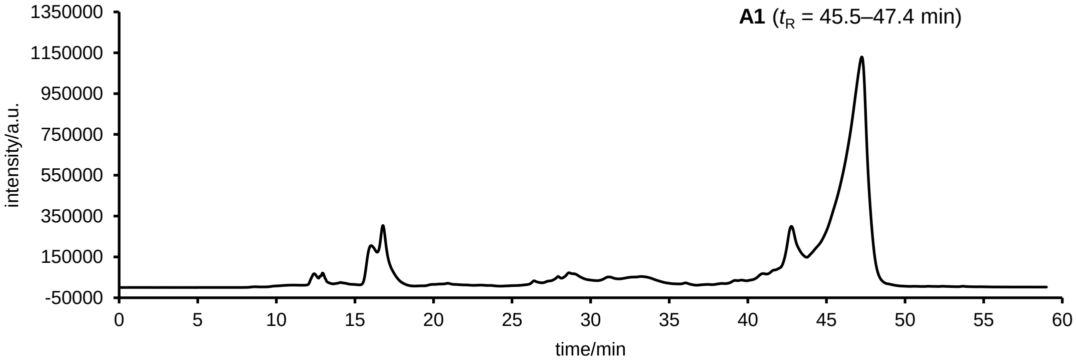

Supplementary Figure 46. HPLC chart for HPLC purification of **A1**. Column: Inertsil C8-3 20 × 250 mm, eluent A: MeOH + 0.05% TFA, eluent B: H_2O + 0.05% TFA, linear gradient A/B = 60/40 to 100/0 over 40 min, flow rate: 5.0 mL/min, detection: UV 280 nm.

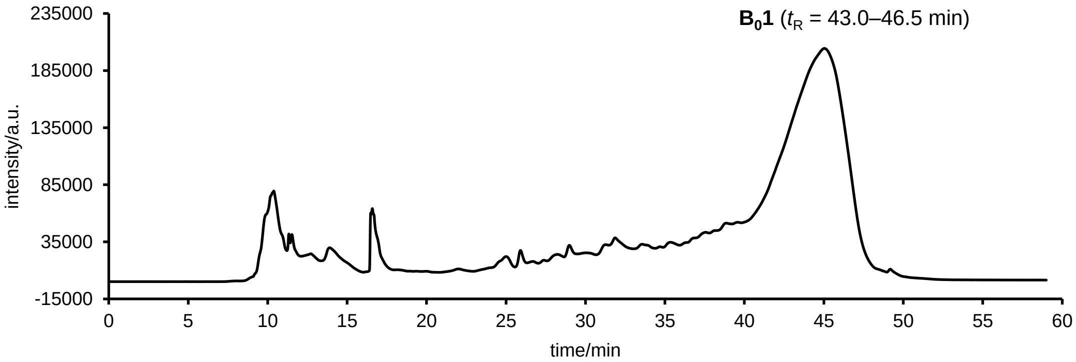

Supplementary Figure 47. HPLC chart for HPLC purification of **$B_0$1**. Column: Inertsil C8-3 20 × 250 mm, eluent A: MeOH + 0.05% TFA, eluent B: H_2O + 0.05% TFA, linear gradient A/B = 60/40 to 100/0 over 40 min, flow rate: 5.0 mL/min, detection: UV 280 nm.

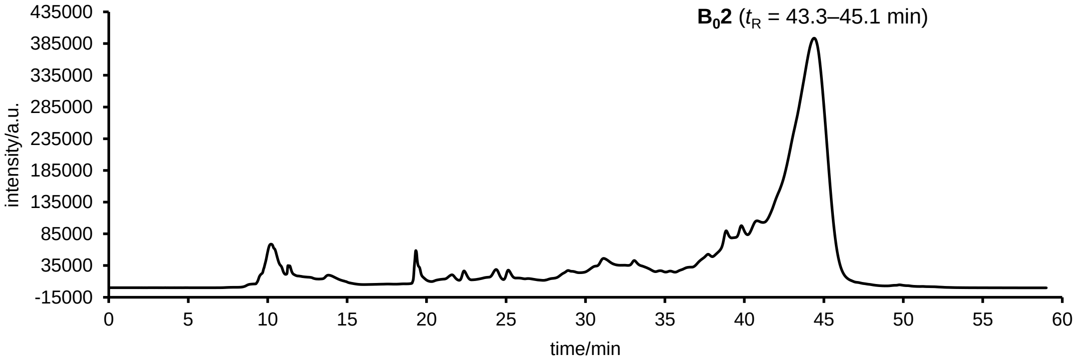

Supplementary Figure 48. HPLC chart for HPLC purification of **$B_0$2**. Column: Inertsil C8-3 20 × 250 mm, eluent A: MeOH + 0.05% TFA, eluent B: H_2O + 0.05% TFA, linear gradient A/B = 60/40 to 100/0 over 40 min, flow rate: 5.0 mL/min, detection: UV 280 nm.

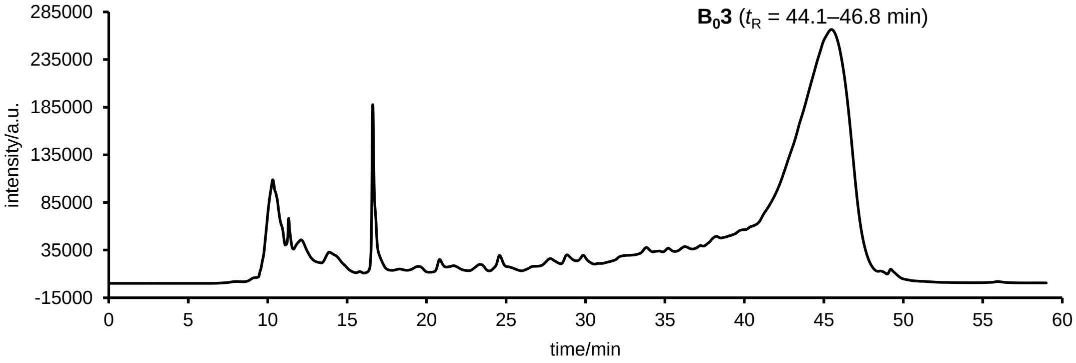

Supplementary Figure 49. HPLC chart for HPLC purification of **$B_0$3**. Column: Inertsil C8-3 20 × 250 mm, eluent A: MeOH + 0.05% TFA, eluent B: H_2O + 0.05% TFA, linear gradient A/B = 60/40 to 100/0 over 40 min, flow rate: 5.0 mL/min, detection: UV 280 nm.

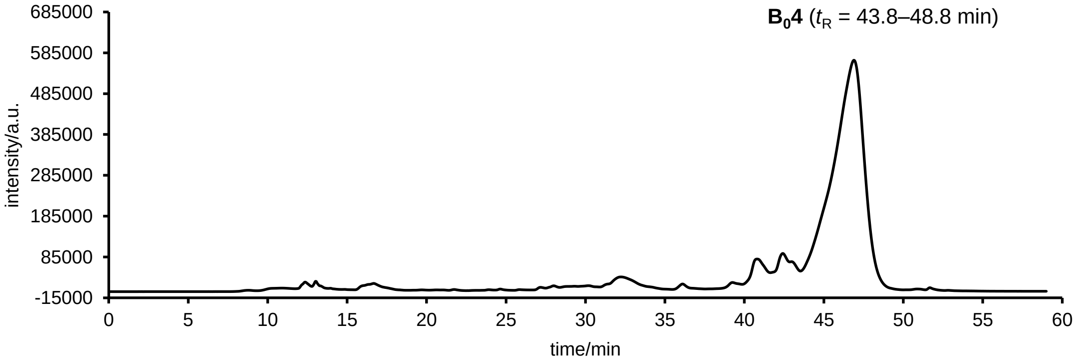

Supplementary Figure 50. HPLC chart for 1st HPLC purification of **$B_0$4**. Column: Inertsil C8-3 20 × 250 mm, eluent A: MeOH + 0.05% TFA, eluent B: H_2O + 0.05% TFA, linear gradient A/B = 60/40 to 100/0 over 40 min, flow rate: 5.0 mL/min, detection: UV 280 nm.

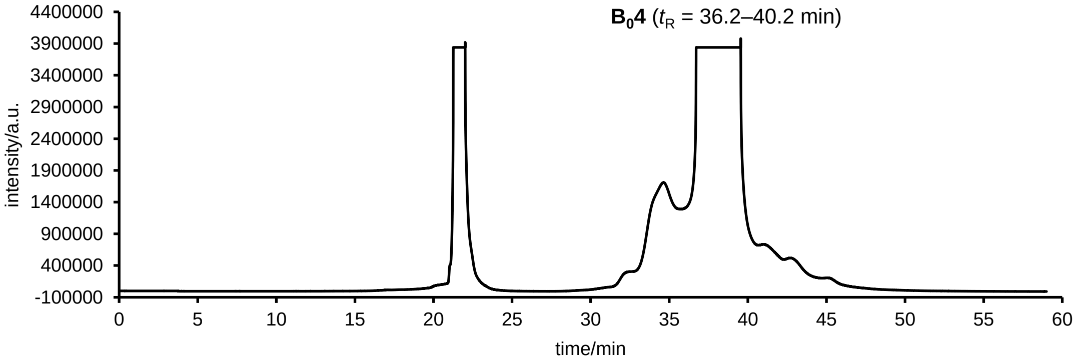

Supplementary Figure 51. HPLC chart for 2nd HPLC purification of **$B_0$4**. Column: TSKgel Amide-80 21.5 × 300 mm, eluent A: MeCN + 0.05% TFA, eluent B: H_2O + 0.05% TFA, linear gradient A/B = 95/5 to 80/20 over 40 min, flow rate: 4.5 mL/min, detection: photodiode array detector 200–650 nm (UV chromatogram: 280 nm).

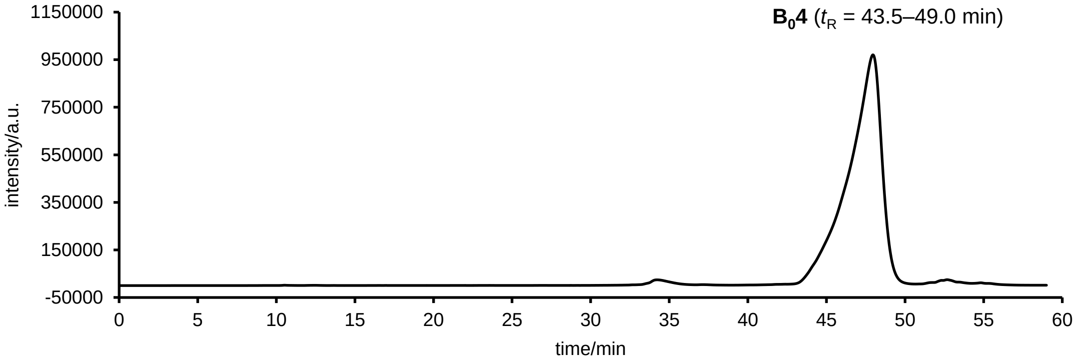

Supplementary Figure 52. HPLC chart for 3rd HPLC purification of **$B_0$4**. Column: Inertsil C8-3 20 × 250 mm, eluent A: MeOH + 0.05% TFA, eluent B: H_2O + 0.05% TFA, linear gradient A/B = 60/40 to 100/0 over 40 min, flow rate: 5.0 mL/min, detection: UV 280 nm.

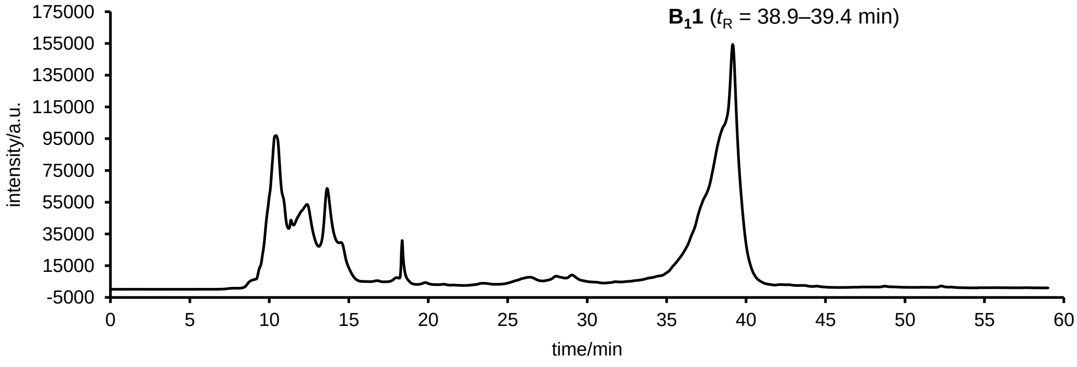

Supplementary Figure 53. HPLC chart for HPLC purification of **$B_1$1**. Column: Inertsil C8-3 20 × 250 mm, eluent A: MeOH + 0.05% TFA, eluent B: H_2O + 0.05% TFA, linear gradient A/B = 60/40 to 100/0 over 40 min, flow rate: 5.0 mL/min, detection: UV 280 nm.

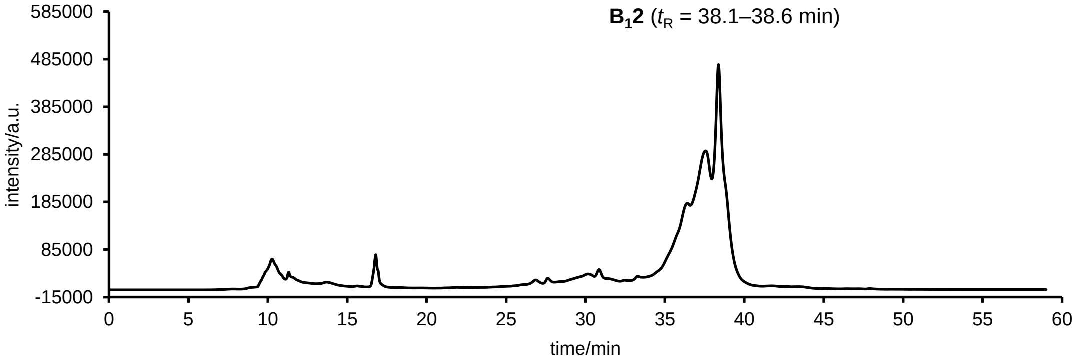

Supplementary Figure 54. HPLC chart for 1st HPLC purification of **B$_1$2**. Column: Inertsil C8-3 20 × 250 mm, eluent A: MeOH + 0.05% TFA, eluent B: H_2O + 0.05% TFA, linear gradient A/B = 60/40 to 100/0 over 40 min, flow rate: 5.0 mL/min, detection: UV 280 nm.

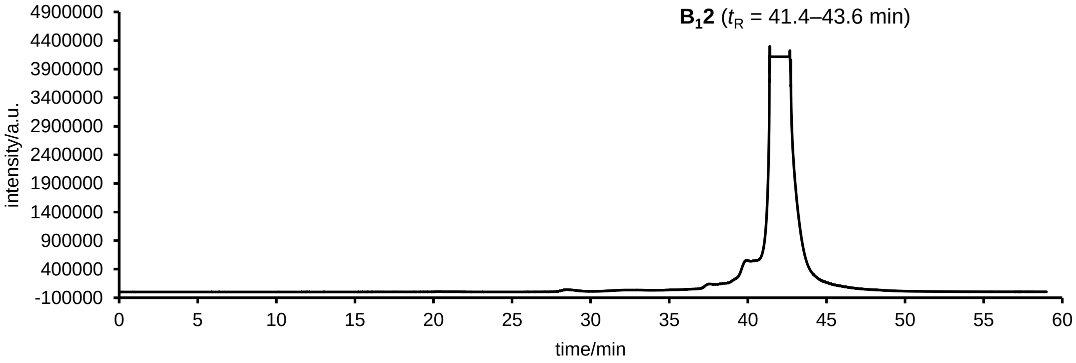

Supplementary Figure 55. HPLC chart for 2nd HPLC purification of **B$_1$2**. Column: TSKgel Amide-80 21.5 × 300 mm, eluent A: MeCN + 0.05% TFA, eluent B: H_2O + 0.05% TFA, linear gradient A/B = 95/5 to 80/20 over 40 min, flow rate: 4.5 mL/min, detection: photodiode array detector 200–650 nm (UV chromatogram: 280 nm).

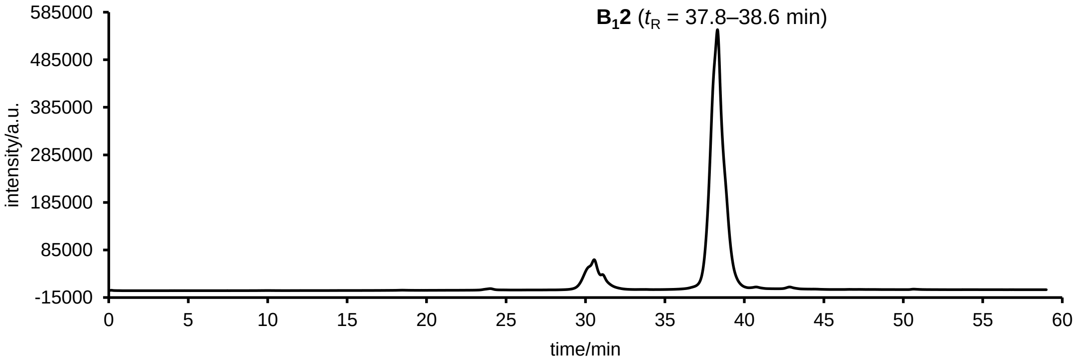

Supplementary Figure 56. HPLC chart for 3rd HPLC purification of **B$_1$2**. Column: Inertsil C8-3 20 × 250 mm, eluent A: MeOH + 0.05% TFA, eluent B: H_2O + 0.05% TFA, linear gradient A/B = 60/40 to 100/0 over 40 min, flow rate: 5.0 mL/min, detection: UV 280 nm.

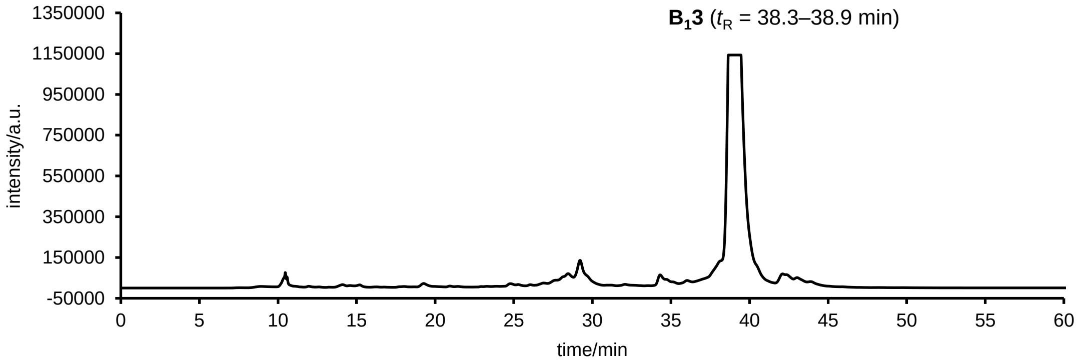

Supplementary Figure 57. HPLC chart for 1st HPLC purification of **$B_1$3**. Column: Inertsil C8-3 20 × 250 mm, eluent A: MeOH + 0.05% TFA, eluent B: H_2O + 0.05% TFA, linear gradient A/B = 60/40 to 100/0 over 40 min, flow rate: 5.0 mL/min, detection: UV 280 nm.

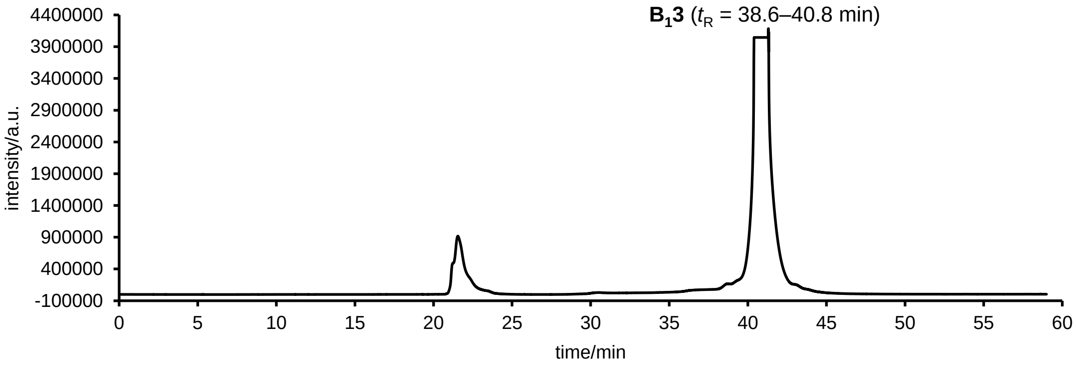

Supplementary Figure 58. HPLC chart for 2nd HPLC purification of **$B_1$3**. Column: TSKgel Amide-80 21.5 × 300 mm, eluent A: MeCN + 0.05% TFA, eluent B: H_2O + 0.05% TFA, linear gradient A/B = 95/5 to 80/20 over 40 min, flow rate: 4.5 mL/min, detection: photodiode array detector 200–650 nm (UV chromatogram: 280 nm).

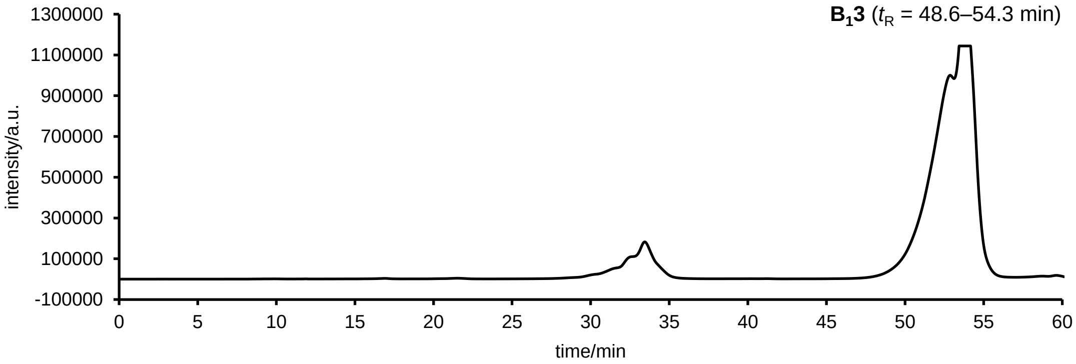

Supplementary Figure 59. HPLC chart for 3rd HPLC purification of **$B_1$3**. Column: Inertsil C8-3 20 × 250 mm, eluent A: MeOH + 0.05% TFA, eluent B: H_2O + 0.05% TFA, linear gradient A/B = 65/35 to 100/0 over 80 min, flow rate: 5.0 mL/min, detection: UV 280 nm.

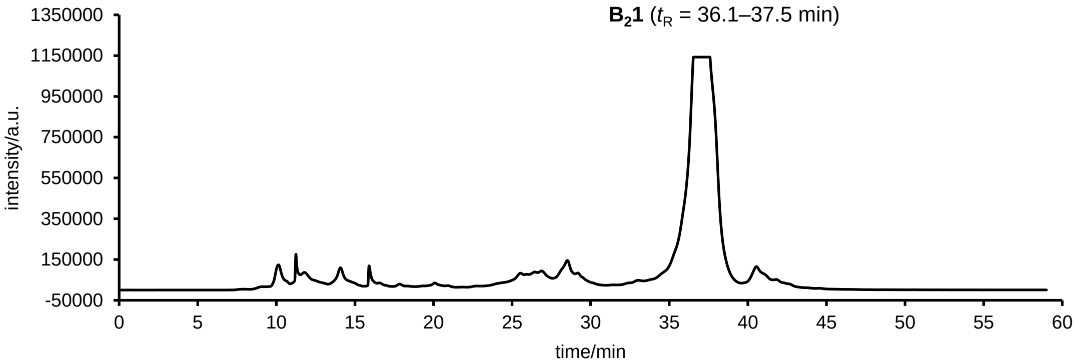

Supplementary Figure 60. HPLC chart for 1st HPLC purification of **$B_2$1**. Column: Inertsil C8-3 20 × 250 mm, eluent A: MeOH + 0.05% TFA, eluent B: H_2O + 0.05% TFA, linear gradient A/B = 60/40 to 100/0 over 40 min, flow rate: 5.0 mL/min, detection: UV 280 nm.

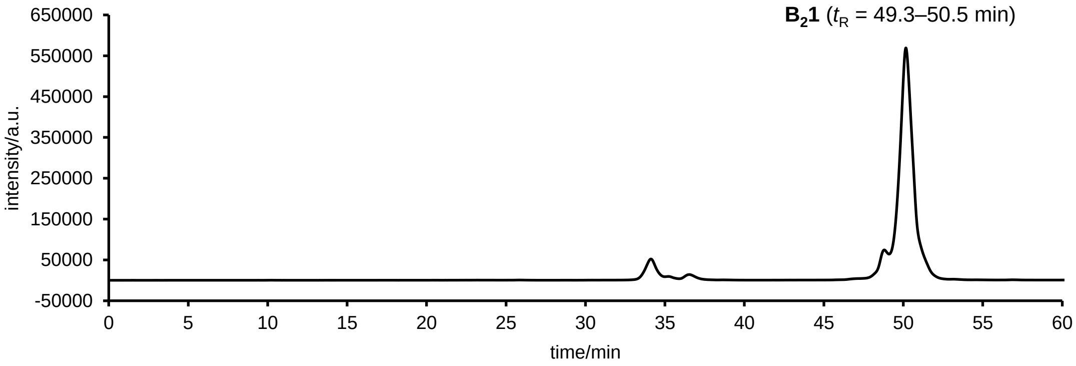

Supplementary Figure 61. HPLC chart for 2nd HPLC purification of **$B_2$1**. Column: Inertsil C8-3 20 × 250 mm, eluent A: MeOH + 0.05% TFA, eluent B: H_2O + 0.05% TFA, linear gradient A/B = 65/35 to 100/0 over 80 min, flow rate: 5.0 mL/min, detection: UV 280 nm.

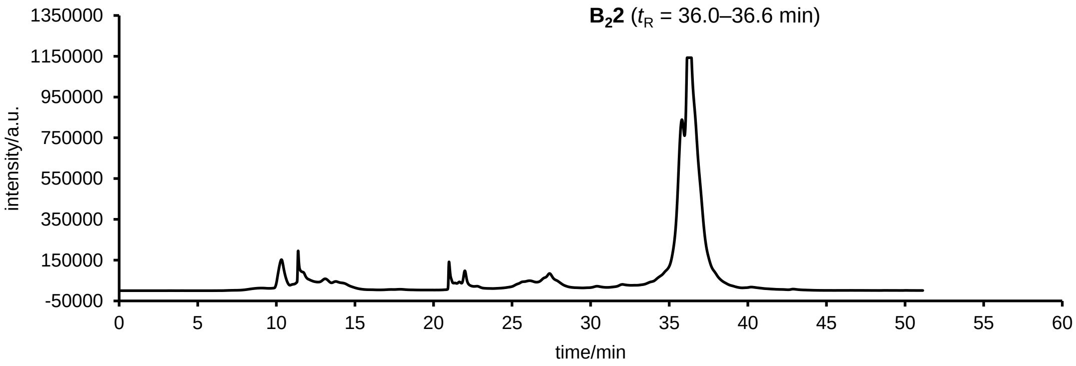

Supplementary Figure 62. HPLC chart for 1st HPLC purification of **$B_2$2**. Column: Inertsil C8-3 20 × 250 mm, eluent A: MeOH + 0.05% TFA, eluent B: H_2O + 0.05% TFA, linear gradient A/B = 60/40 to 100/0 over 40 min, flow rate: 5.0 mL/min, detection: UV 280 nm.

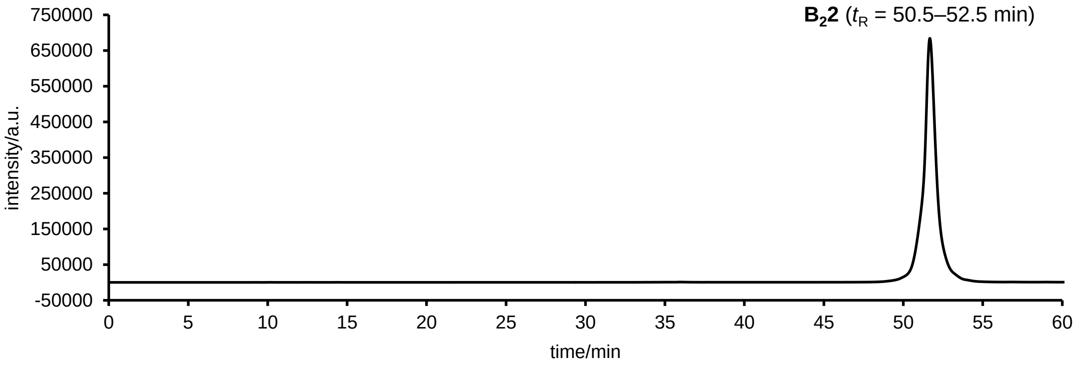

Supplementary Figure 63. HPLC chart for 2nd HPLC purification of **B**$_2$**2**. Column: Inertsil C8-3 20 × 250 mm, eluent A: MeOH + 0.05% TFA, eluent B: H_2O + 0.05% TFA, linear gradient A/B = 65/35 to 100/0 over 80 min, flow rate: 5.0 mL/min, detection: UV 280 nm.

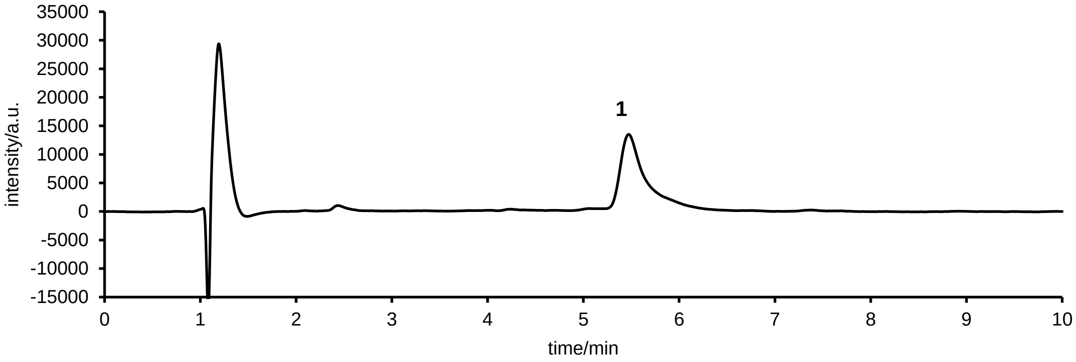

Supplementary Figure 64. UHPLC chart of purified **1**. Column: Accucore C18 2.1 ×150 mm, eluent A: MeOH + 0.05% TFA, eluent B: H_2O + 0.05% TFA, A/B = 79/21, flow rate: 0.30 mL/min, detection: photodiode array detector 200–648 nm (UV chromatogram: 280 nm), temperature: 40 ºC.

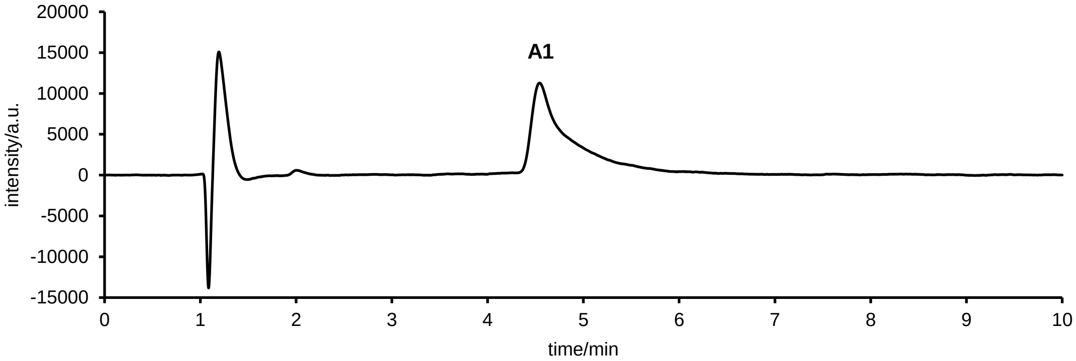

Supplementary Figure 65. UHPLC chart of purified **A1**. Column: Accucore C18 2.1 ×150 mm, eluent A: MeOH + 0.05% TFA, eluent B: H_2O + 0.05% TFA, A/B = 79/21, flow rate: 0.30 mL/min, detection: photodiode array detector 200–648 nm (UV chromatogram: 280 nm), temperature: 40 ºC.

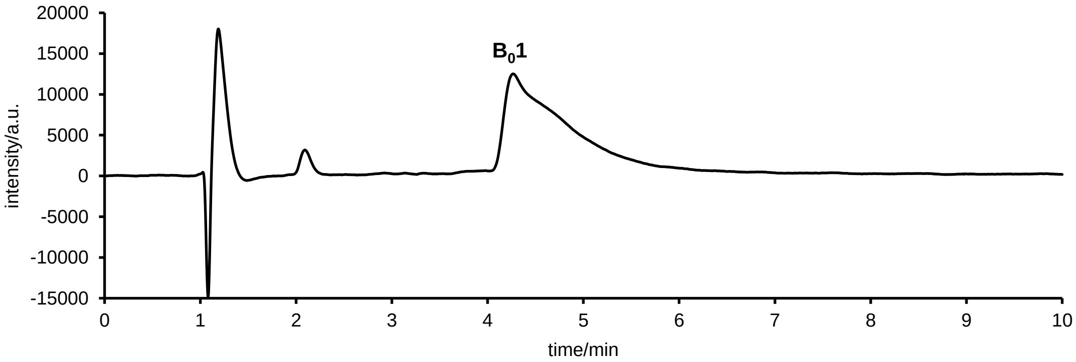

Supplementary Figure 66. UHPLC chart of purified **$B_0$1**. Column: Accucore C18 2.1 ×150 mm, eluent A: MeOH + 0.05% TFA, eluent B: H_2O + 0.05% TFA, A/B = 79/21, flow rate: 0.30 mL/min, detection: photodiode array detector 200–648 nm (UV chromatogram: 280 nm), temperature: 40 ºC.

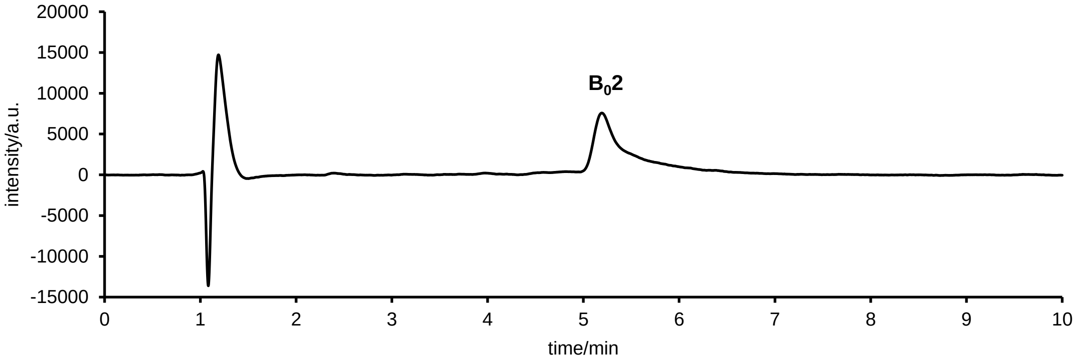

Supplementary Figure 67. UHPLC chart of purified **$B_0$2**. Column: Accucore C18 2.1 ×150 mm, eluent A: MeOH + 0.05% TFA, eluent B: H_2O + 0.05% TFA, A/B = 79/21, flow rate: 0.30 mL/min, detection: photodiode array detector 200–648 nm (UV chromatogram: 280 nm), temperature: 40 ºC.

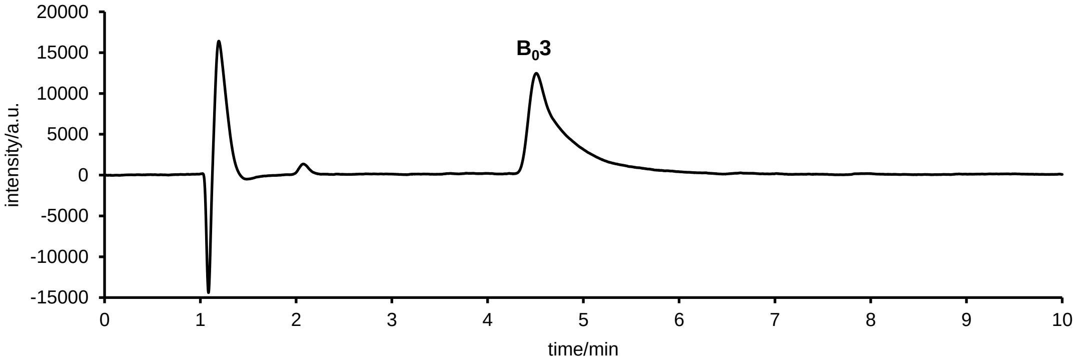

Supplementary Figure 68. UHPLC chart of purified **$B_0$3**. Column: Accucore C18 2.1 ×150 mm, eluent A: MeOH + 0.05% TFA, eluent B: H_2O + 0.05% TFA, A/B = 79/21, flow rate: 0.30 mL/min, detection: photodiode array detector 200–648 nm (UV chromatogram: 280 nm), temperature: 40 ºC.

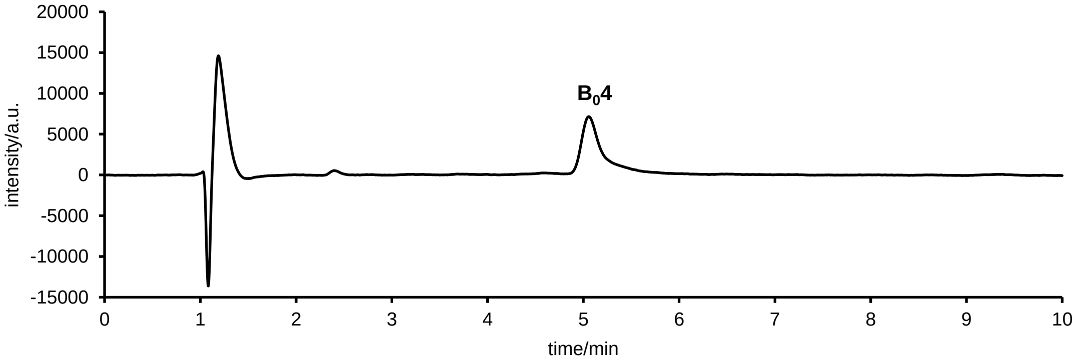

Supplementary Figure 69. UHPLC chart of purified **$B_0$4**. Column: Accucore C18 2.1 ×150 mm, eluent A: MeOH + 0.05% TFA, eluent B: H_2O + 0.05% TFA, A/B = 79/21, flow rate: 0.30 mL/min, detection: photodiode array detector 200–648 nm (UV chromatogram: 280 nm), temperature: 40 ºC.

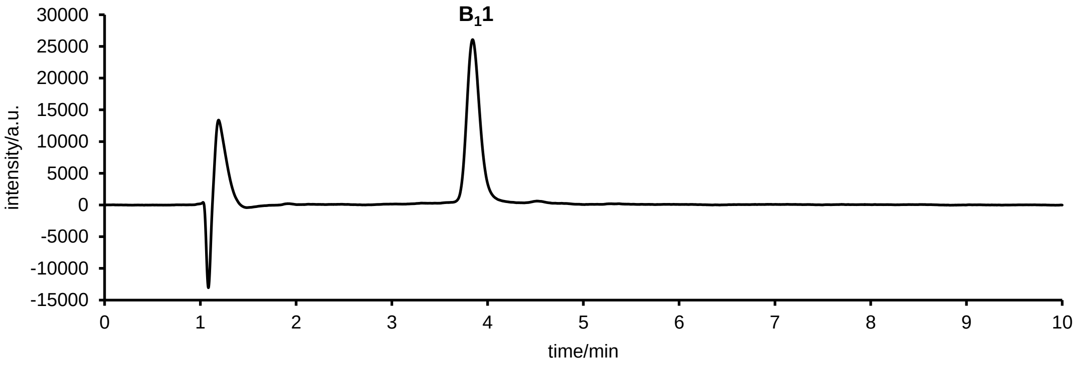

Supplementary Figure 70. UHPLC chart of purified **B$_1$1**. Column: Accucore C18 2.1 ×150 mm, eluent A: MeOH + 0.05% TFA, eluent B: H_2O + 0.05% TFA, A/B = 79/21, flow rate: 0.30 mL/min, detection: photodiode array detector 200–648 nm (UV chromatogram: 280 nm), temperature: 40 ºC.

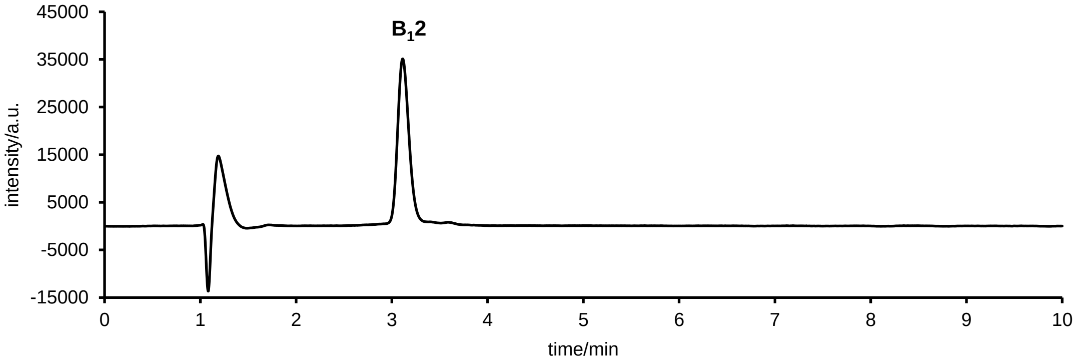

Supplementary Figure 71. UHPLC chart of purified **B$_1$2**. Column: Accucore C18 2.1 ×150 mm, eluent A: MeOH + 0.05% TFA, eluent B: H_2O + 0.05% TFA, A/B = 79/21, flow rate: 0.30 mL/min, detection: photodiode array detector 200–648 nm (UV chromatogram: 280 nm), temperature: 40 ºC.

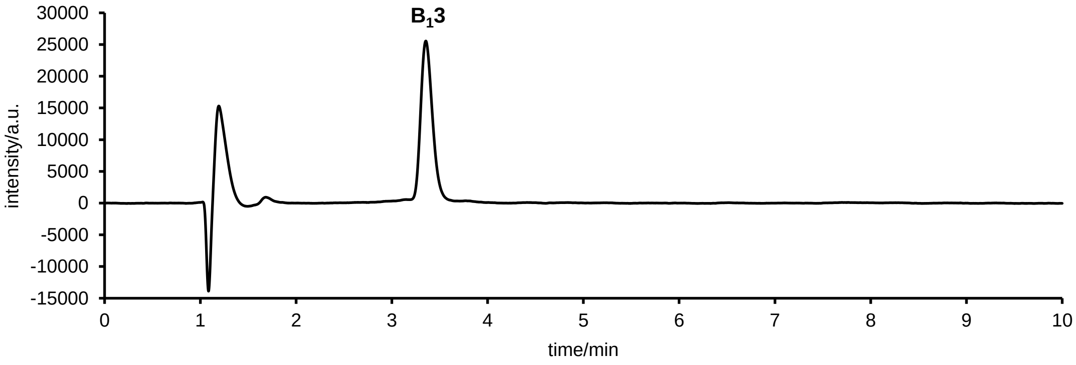

Supplementary Figure 72. UHPLC chart of purified **B$_1$3**. Column: Accucore C18 2.1 ×150 mm, eluent A: MeOH + 0.05% TFA, eluent B: H_2O + 0.05% TFA, A/B = 79/21, flow rate: 0.30 mL/min, detection: photodiode array detector 200–648 nm (UV chromatogram: 280 nm), temperature: 40 ºC.

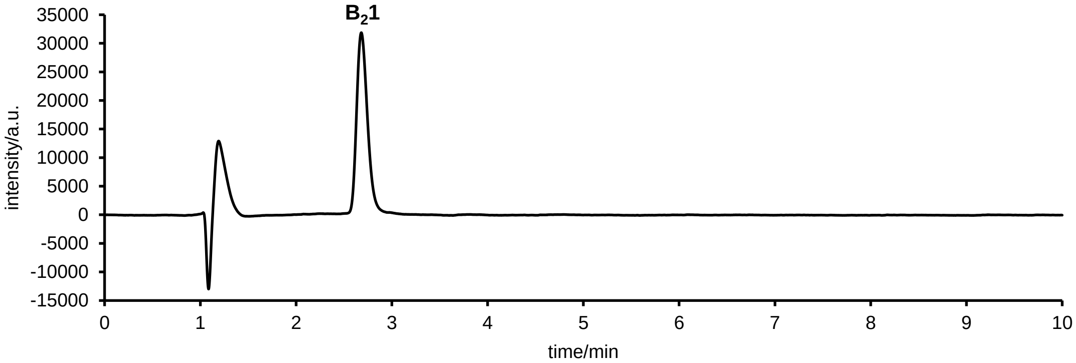

Supplementary Figure 73. UHPLC chart of purified **B**$_2$**1**. Column: Accucore C18 2.1 ×150 mm, eluent A: MeOH + 0.05% TFA, eluent B: H_2O + 0.05% TFA, A/B = 79/21, flow rate: 0.30 mL/min, detection: photodiode array detector 200–648 nm (UV chromatogram: 280 nm), temperature: 40 ºC.

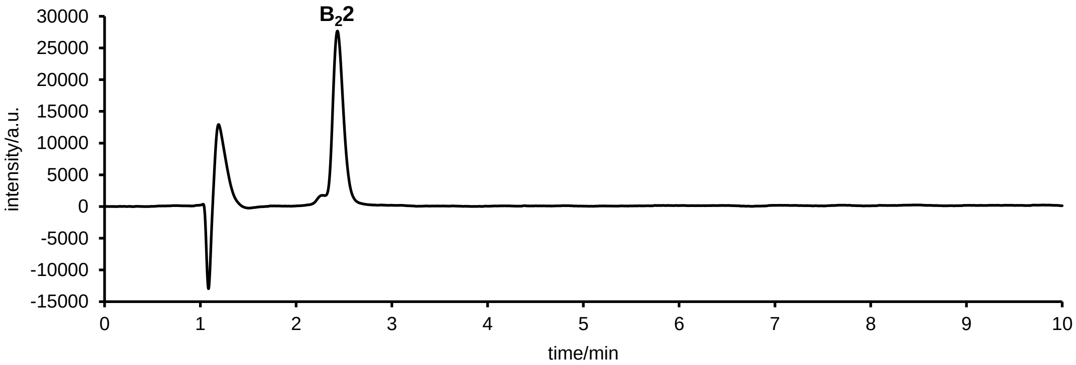

Supplementary Figure 74. UHPLC chart of purified **B**$_2$**2**. Column: Accucore C18 2.1 ×150 mm, eluent A: MeOH + 0.05% TFA, eluent B: H_2O + 0.05% TFA, A/B = 79/21, flow rate: 0.30 mL/min, detection: photodiode array detector 200–648 nm (UV chromatogram: 280 nm), temperature: 40 ºC.

plate 0/line A/column 1

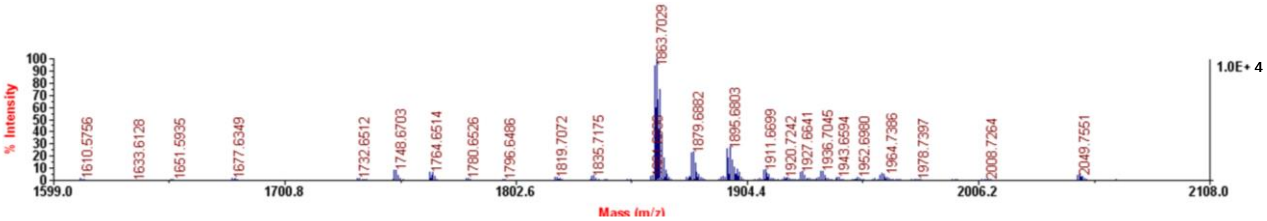

plate 1/line A/column 5

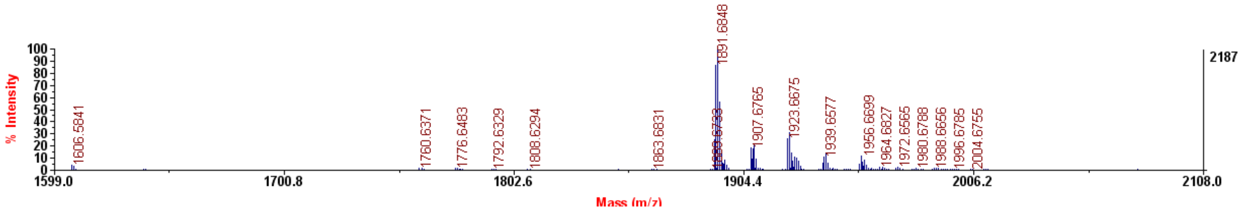

plate 1/line G/column 6

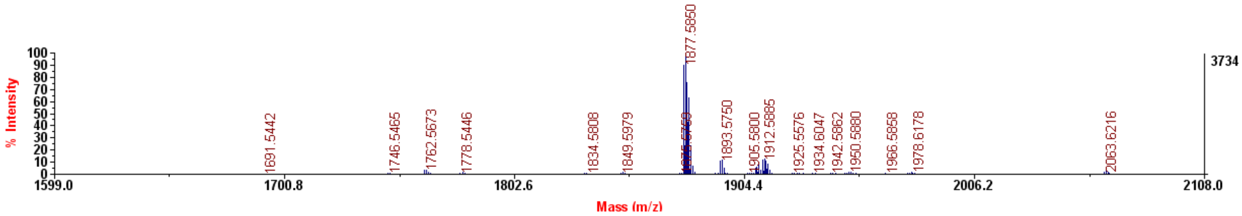

plate 1/line G/column 7

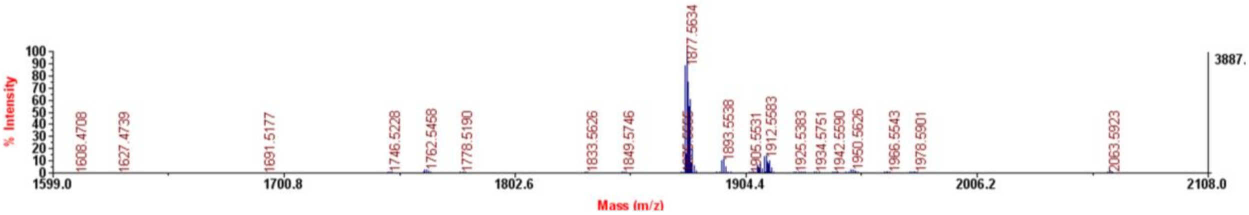

plate 1/line F/column 8

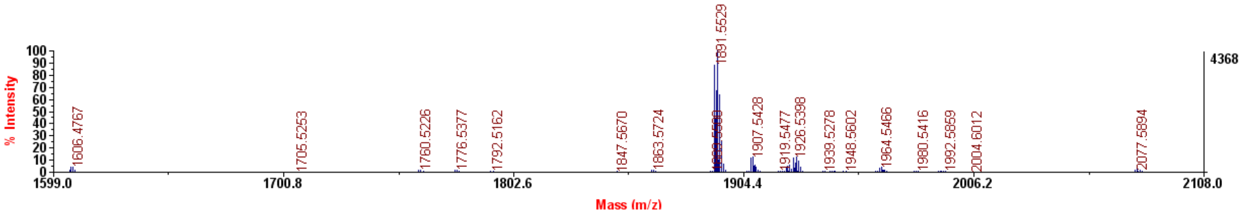

plate 1/line A/column 9

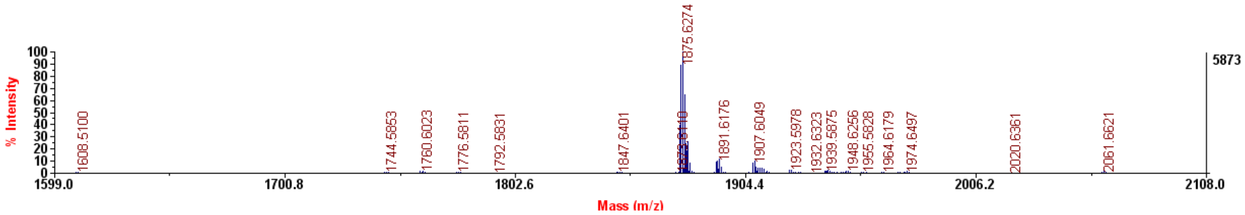

Supplementary Figure 75. MS spectra of plates 0 and 1. The data of A1 in plate 0 and A5, G6, G7, F8, and A9 in plate 1 are shown.

plate 1/line D/column 9

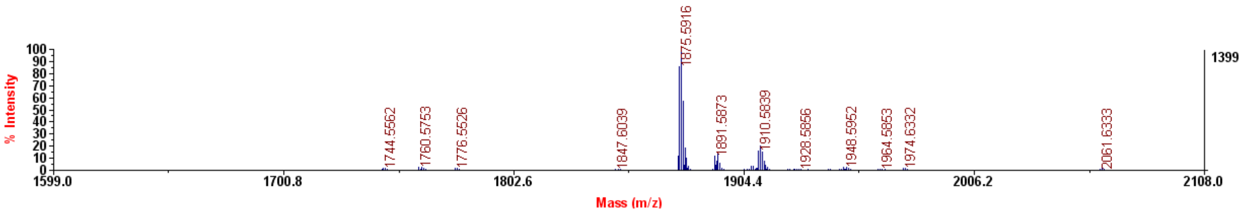

plate 1/line F/column 9

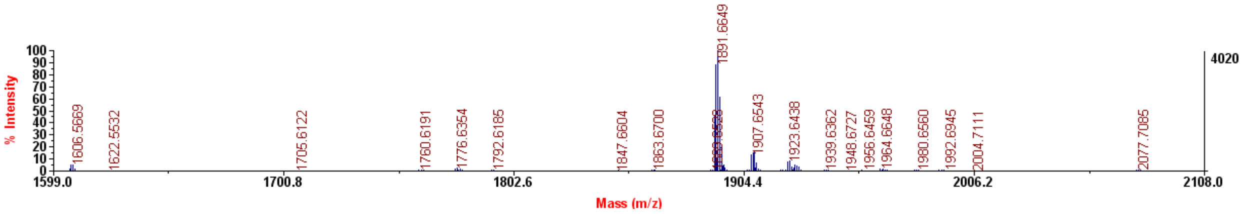

plate 1/line A/column 11

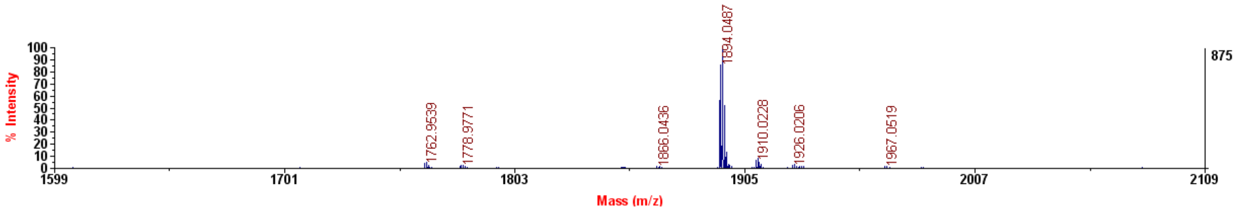

plate 2/line A/column 2

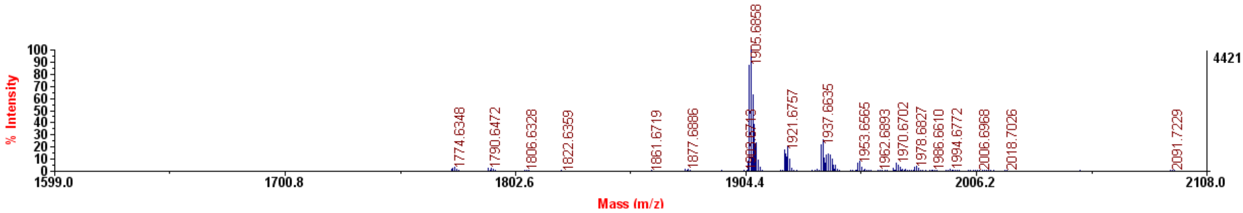

plate 2/line C/column 2

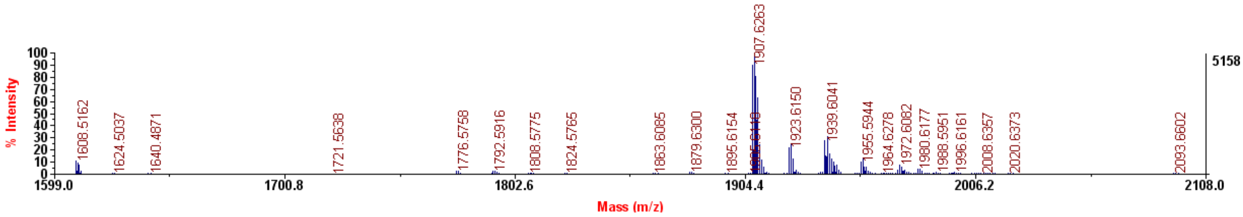

plate 2/line A/column 7

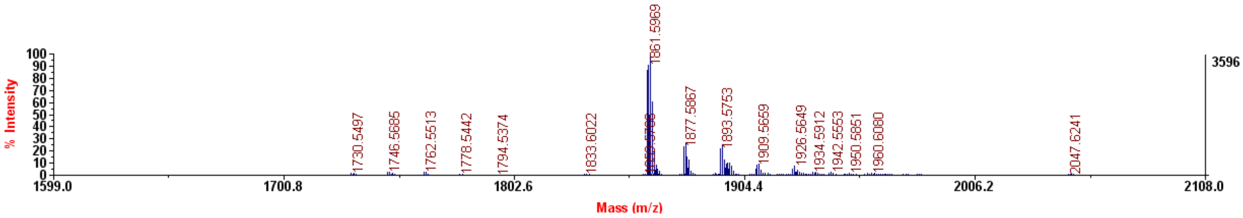

Supplementary Figure 76. MS spectra of plates 1 and 2. The data of D9, F9, and 11A in plate 1 and A2, C2, and A7 in plate 2 are shown.

plate 2/line E/column 7

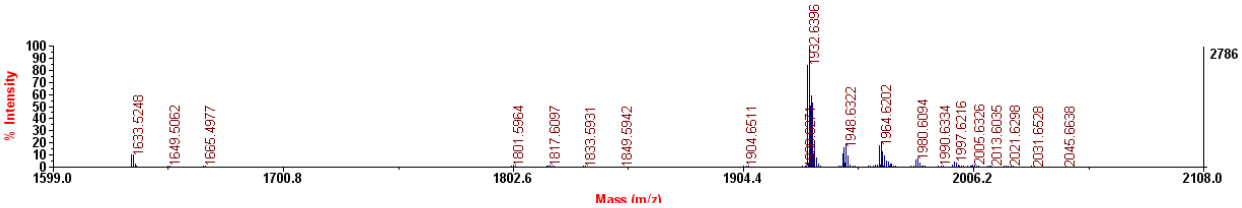

plate 2/line F/column 8

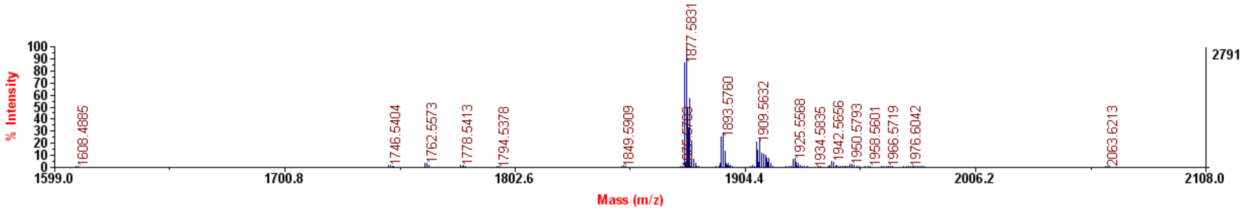

plate 2/line D/column 10

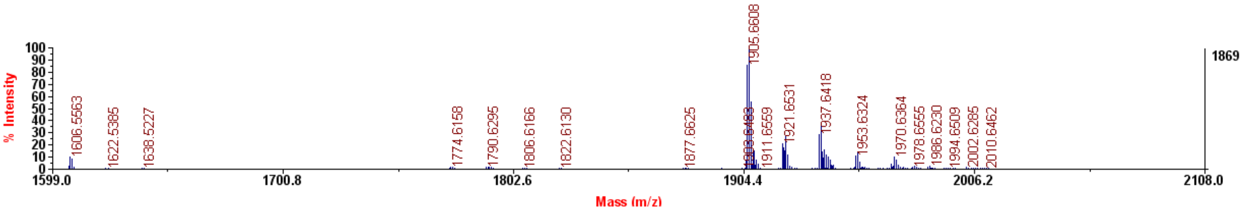

plate 2/line H/column 10

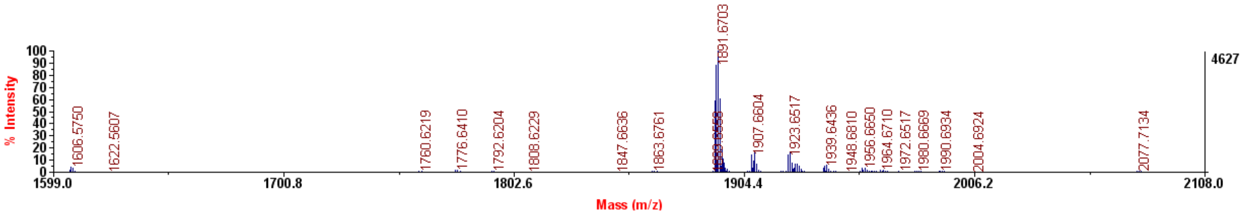

plate 2/line A/column 11

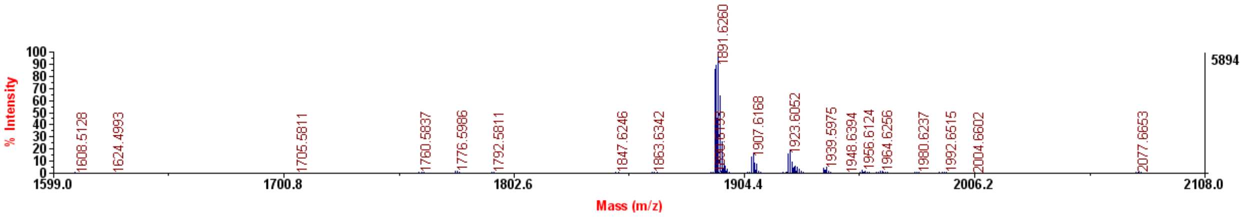

plate 3/line G/column 3

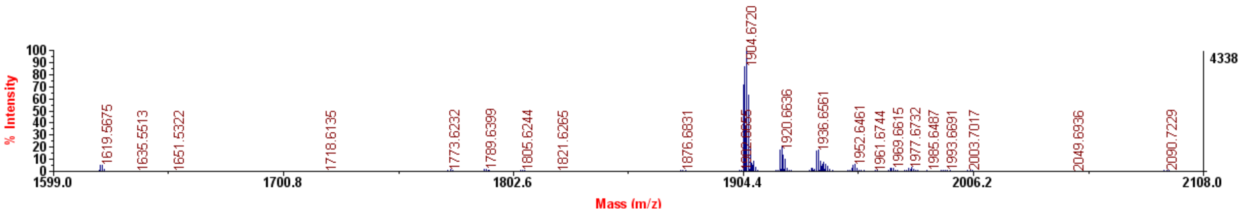

Supplementary Figure 77. MS spectra of plates 2 and 3. The data of E7, F8, D10, H10, and A11 in plate 2 and G3 in plate 3 are shown.

plate 3/line E/column 10

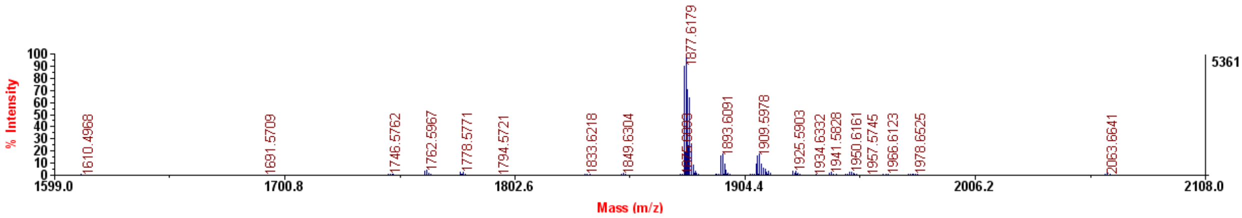

plate 4/line D/column 1

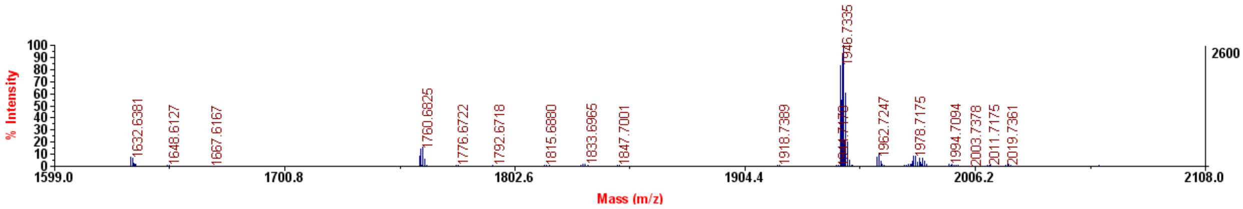

plate 4/line B/column 7

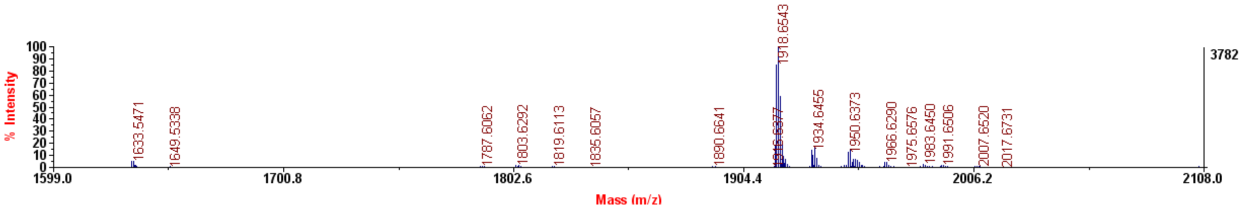

plate 4/line B/column 9

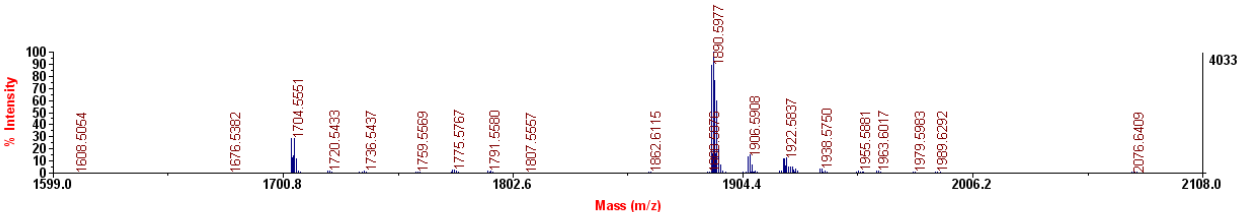

plate 4/line G/column 9

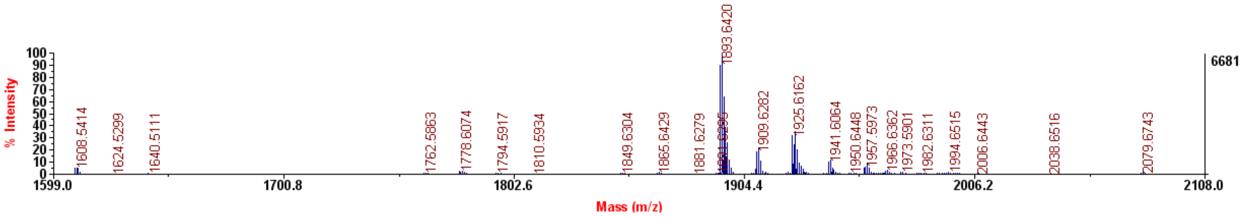

plate 4/line C/column 11

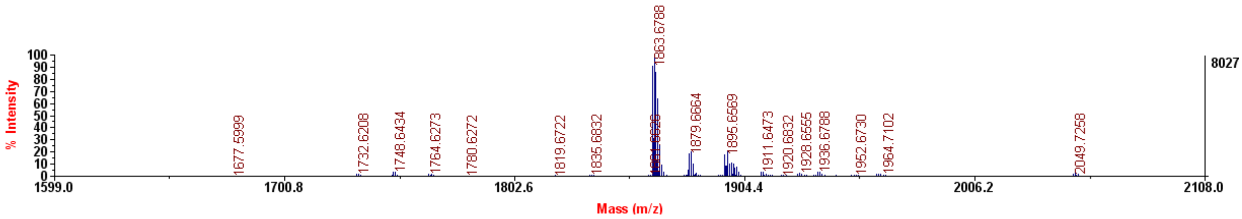

Supplementary Figure 78. MS spectra of plates 3 and 4. The data of E10 in plate 3 and D1, B7, B9, G9, and C11 in plate 4 are shown.

plate 4/line E/column 11

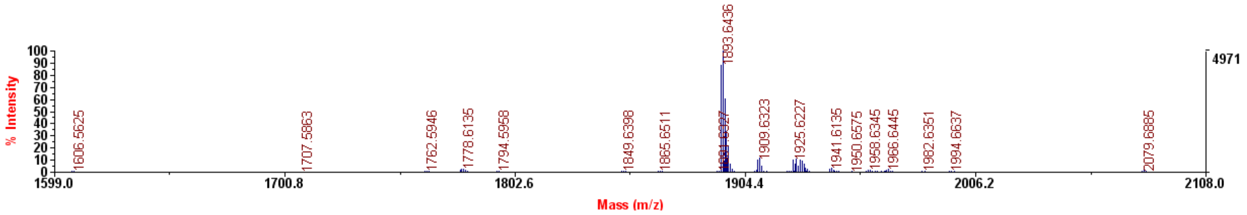

plate 5/line E/column 7

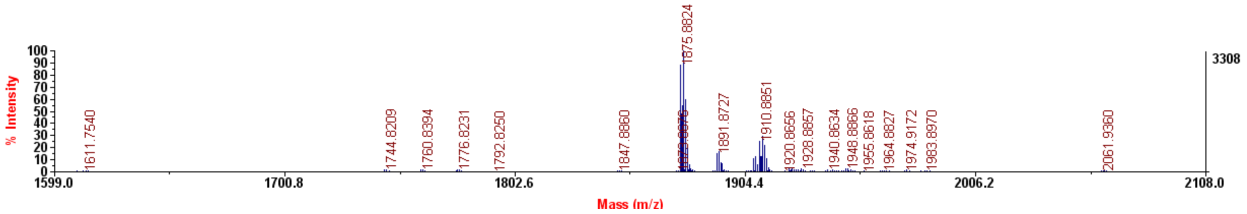

plate 5/line H/column 8

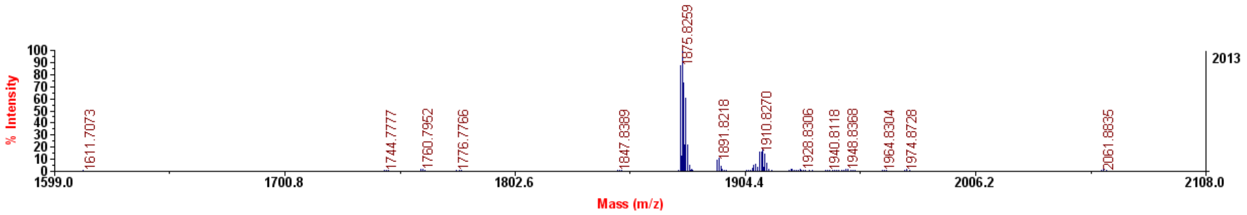

plate 5/line A/column 10

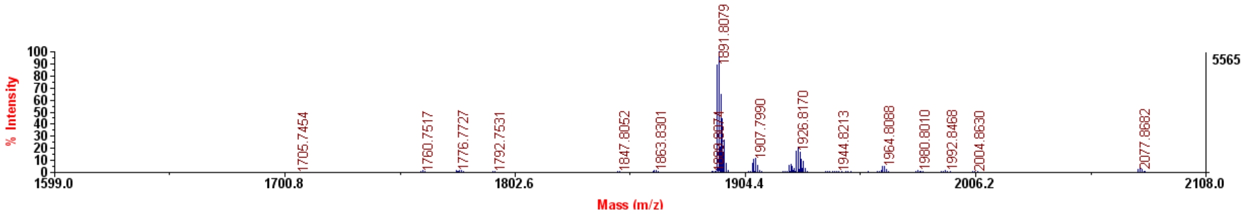

plate 6/line G/column 5

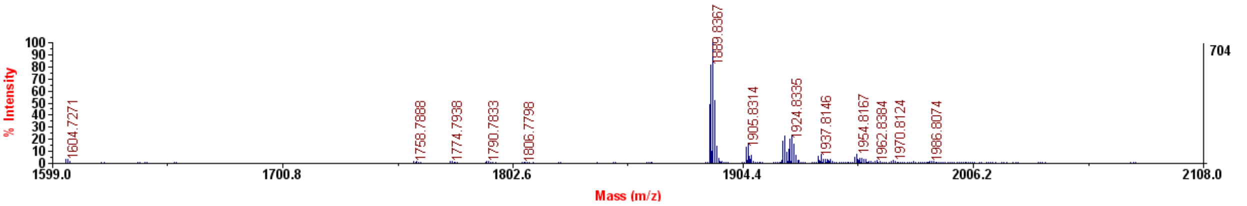

plate 6/line A/column 7

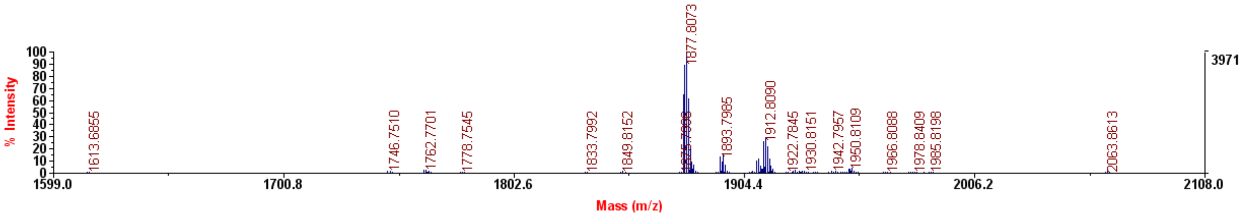

Supplementary Figure 79. MS spectra of plates 4, 5, and 6. The data of E11 in plate 4, E7, H8, and A10 in plate 5, and G5 and A7 in plate 6 are shown.

plate 6/line G/column 7

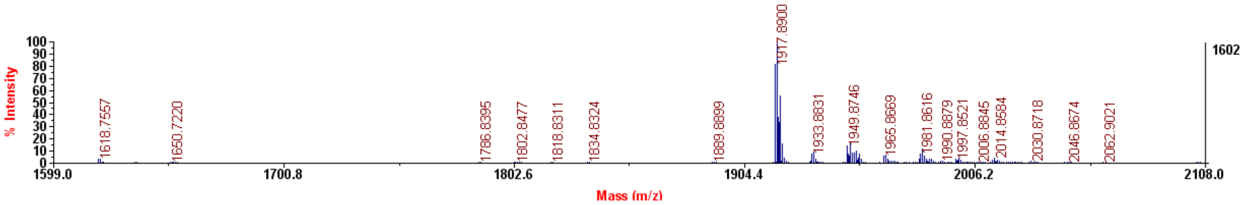

plate 6/line D/column 8

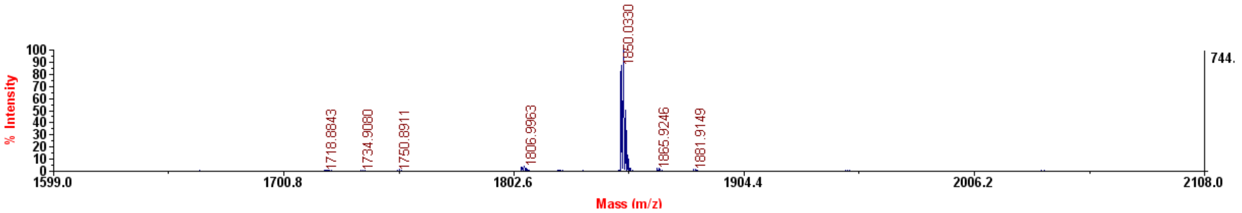

plate 7/line A/column 2

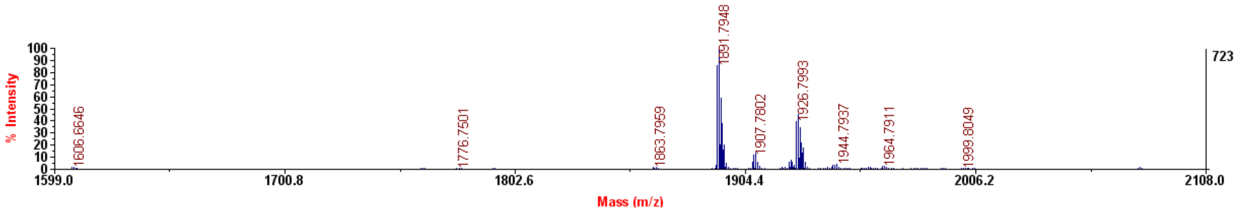

plate 7/line C/column 6

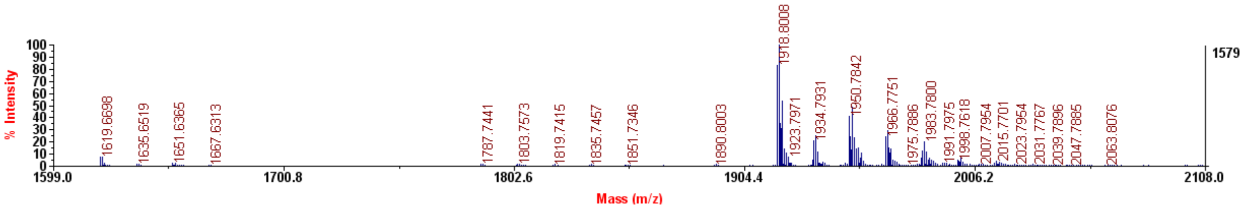

plate 7/line C/column 7

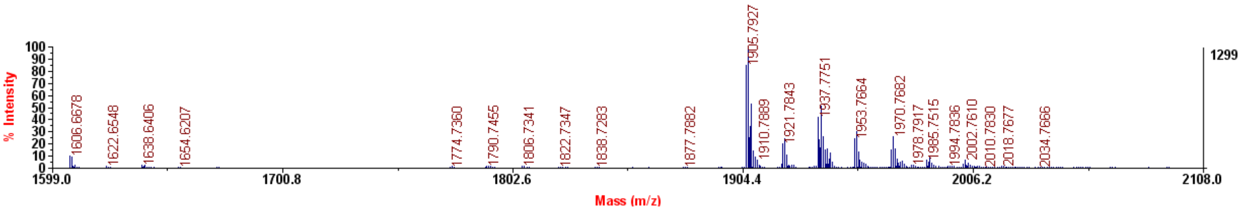

plate 7/line E/column 9

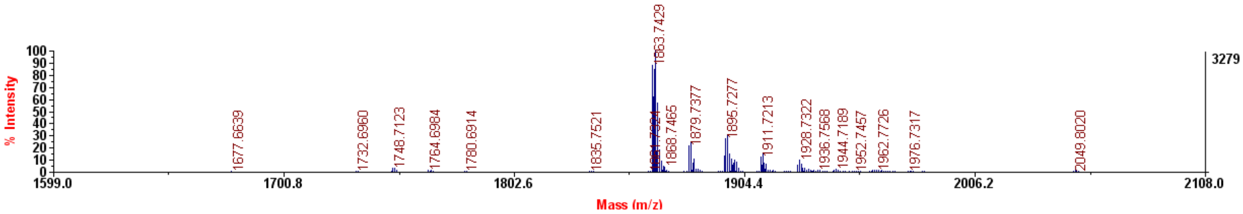

Supplementary Figure 80. MS spectra of plates 6 and 7. The data of G7 and D8 in plate 6 and A2, C6, C7, and E9 in plate 7 are shown.

plate 7/line F/column 10

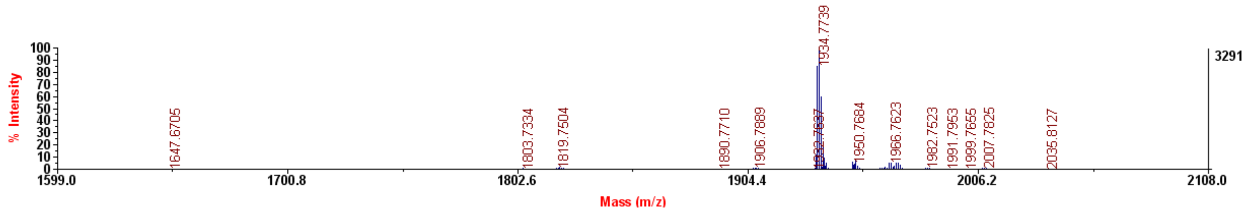

plate 7/line F/column 11

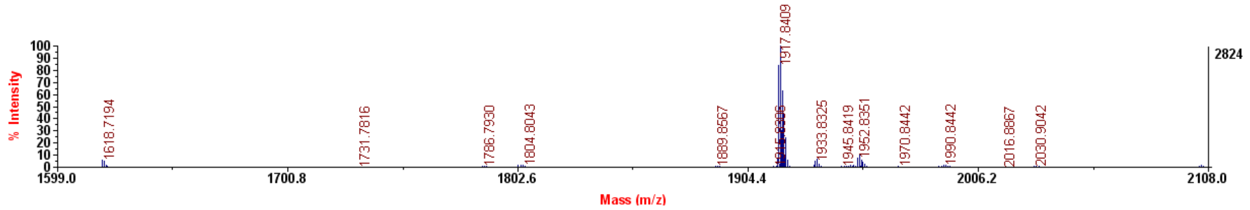

plate 8/line D/column 2

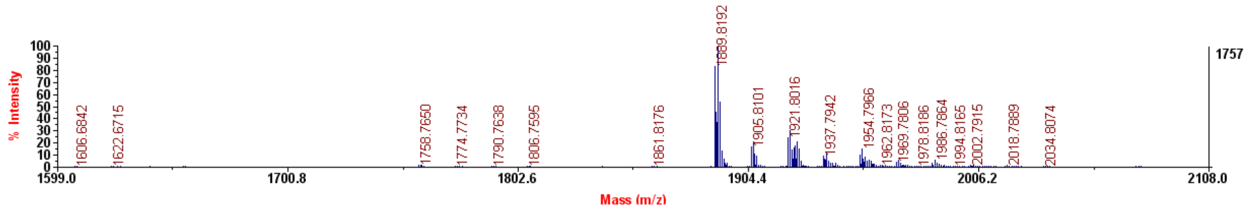

plate 8/line F/column 2

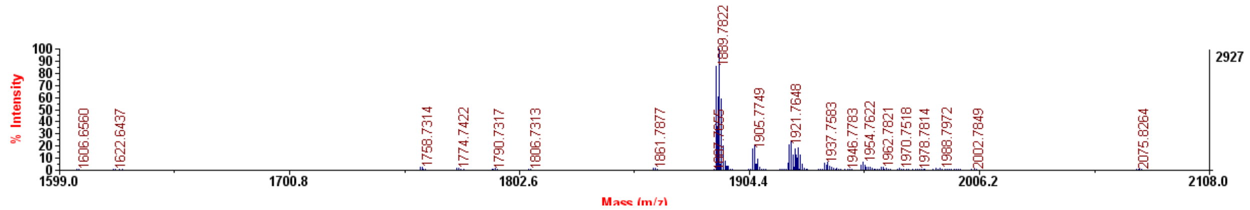

plate 8/line D/column 4

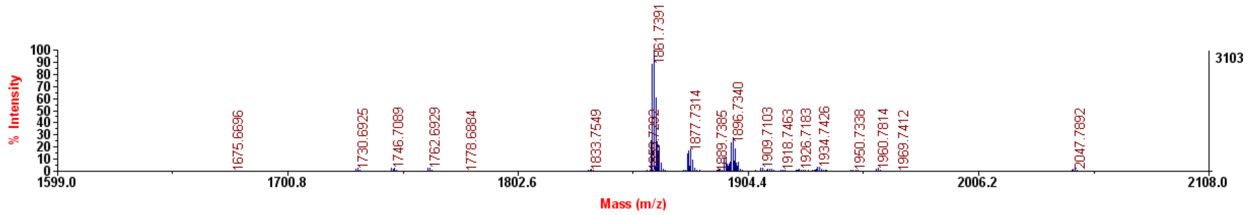

plate 8/line E/column 10

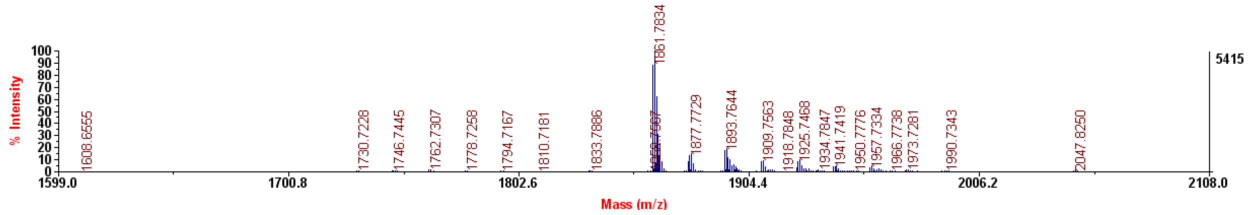

Supplementary Figure 81. MS spectra of plates 7 and 8. The data of F10 and F11 in plate 7 and D2, F2, D4, and E10 in plate 8 are shown.

plate 9/line B/column 4

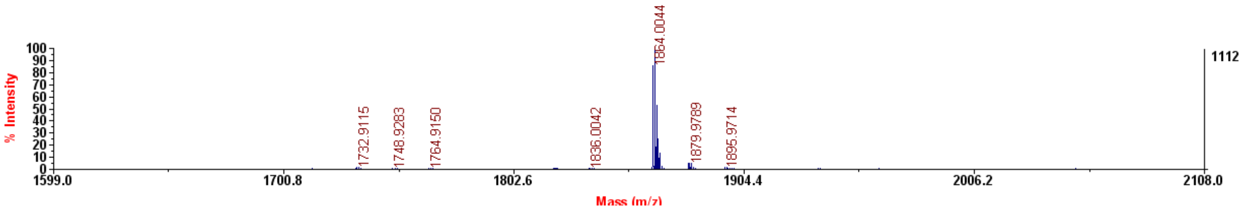

plate 9/line B/column 5

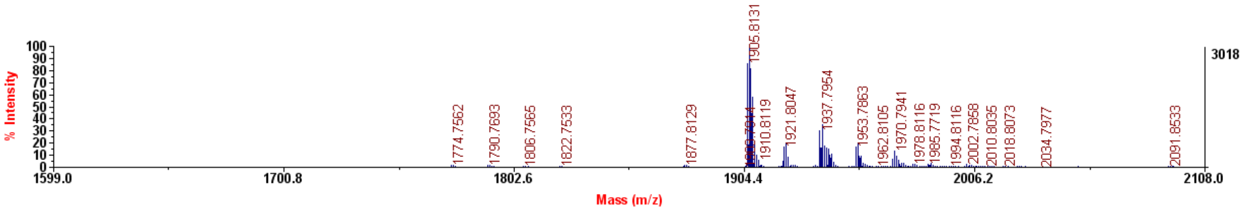

plate 9/line E/column 7

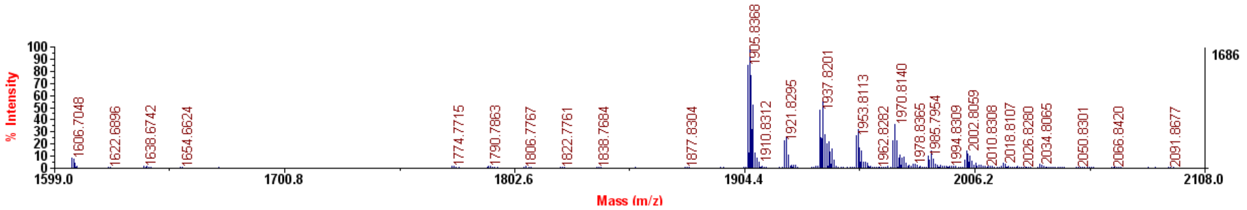

plate 9/line A/column 10

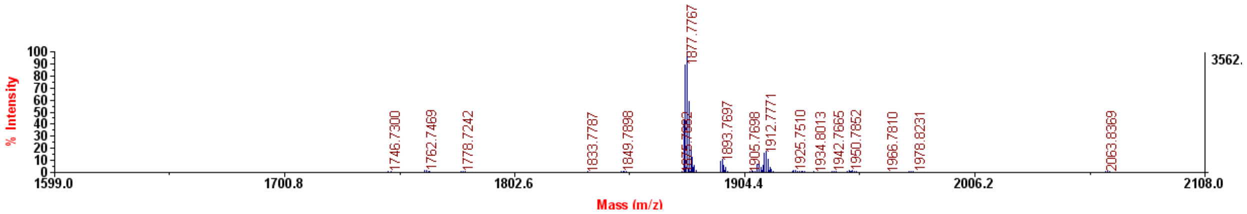

plate 10/line E/column 8

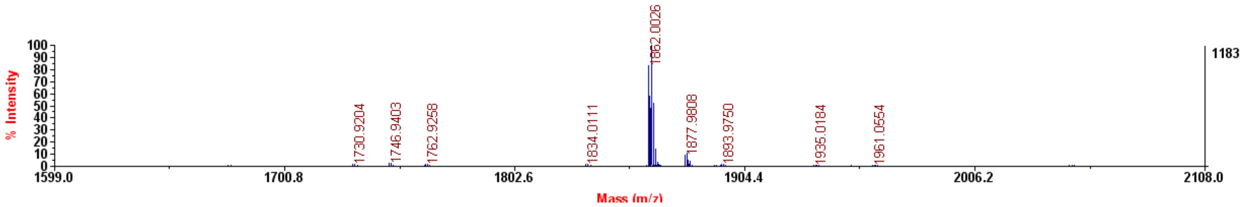

plate 10/line G/column 10

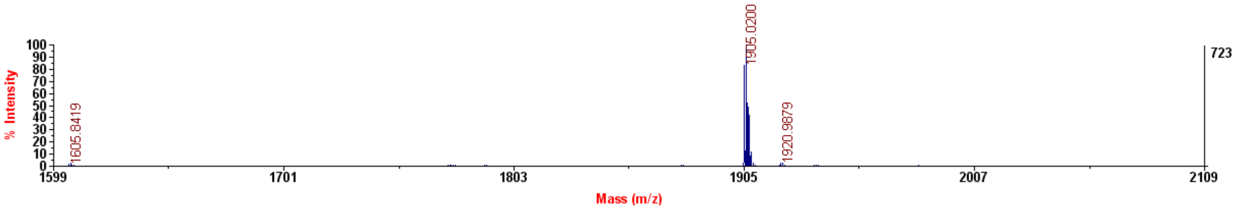

Supplementary Figure 82. MS spectra of plates 9 and 10. The data of B4, B5, E7, and A10 in plate 9 and E8 and G10 in plate 10 are shown.

plate 10/line A/column 11

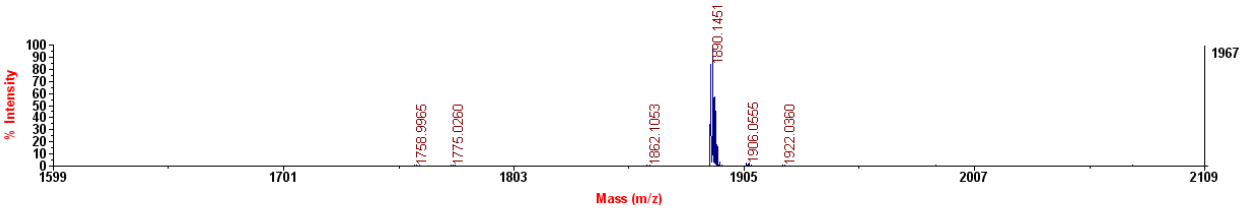

plate 10/line B/column 11

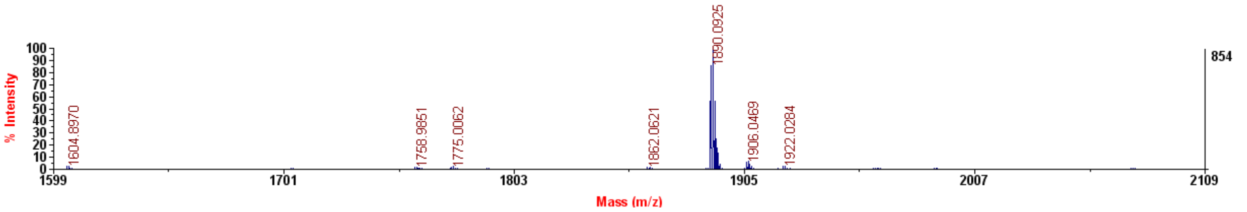

plate 12/line D/column 1

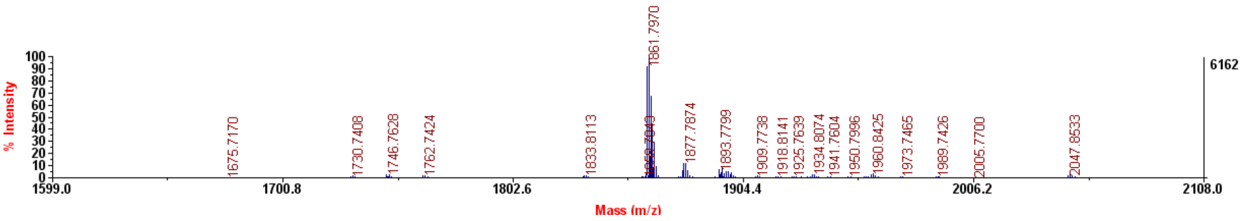

plate 13/line G/column 4

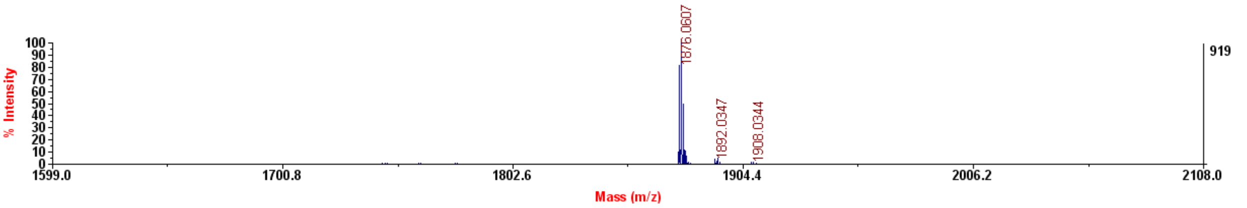

plate 13/line H/column 8

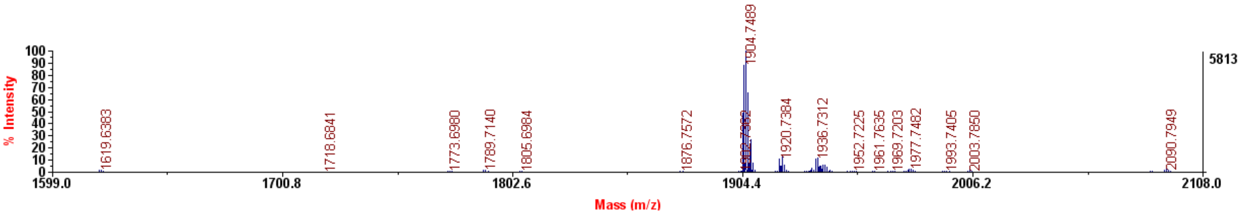

plate 13/line D/column 9

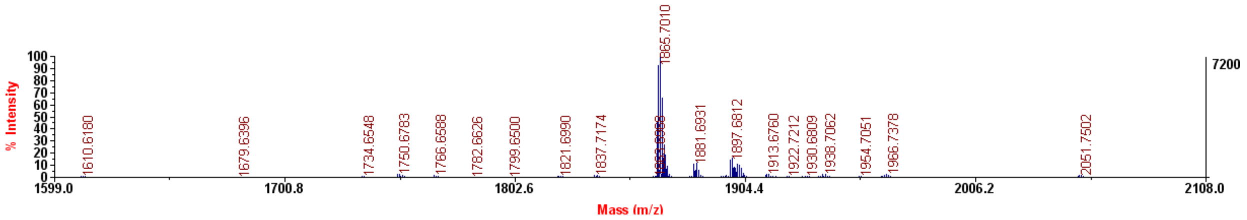

Supplementary Figure 83. MS spectra of plates 10, 12, and 13. The data of A11 and B11 in plate 10, D1 in plate 12, and G4, H8, and D9 in plate 13 are shown.

plate 13/line H/column 11

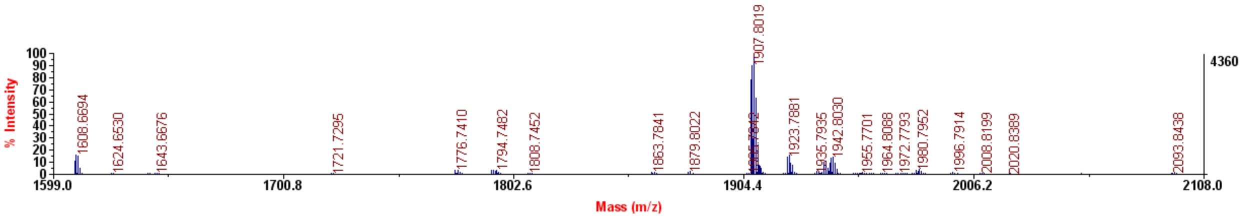

plate 14/line B/column 9

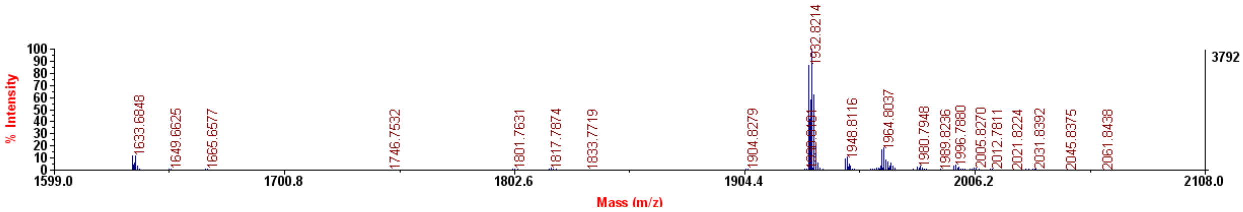

plate 14/line C/column 10

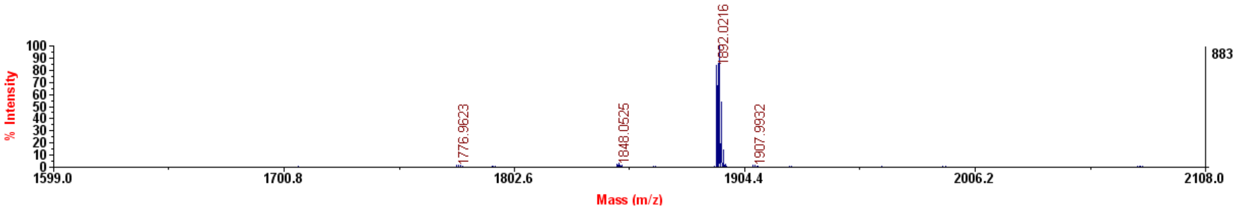

plate 14/line E/column 10

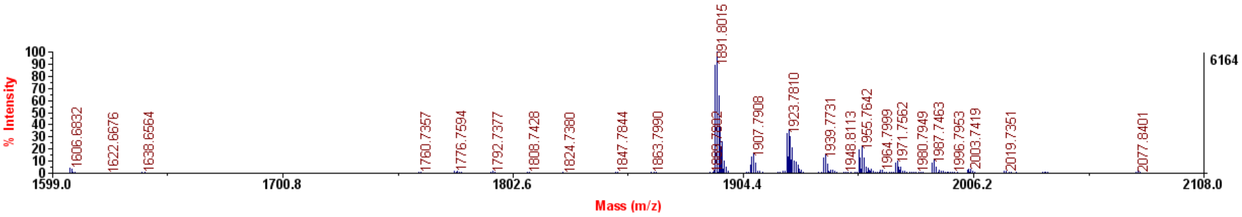

plate 15/line D/column 3

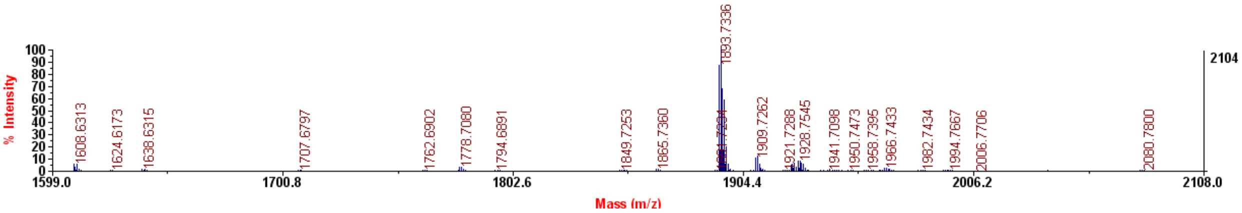

plate 15/line A/column 4

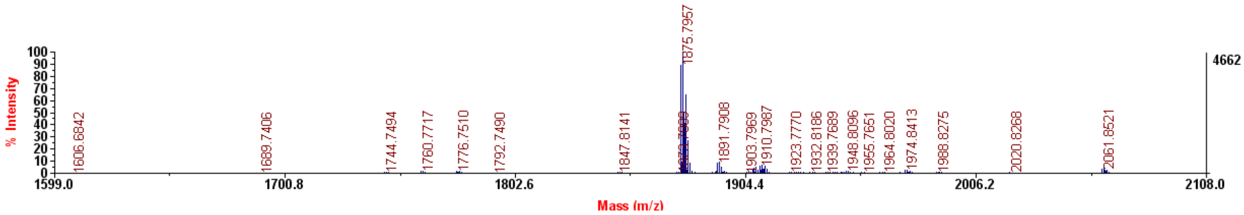

Supplementary Figure 84. MS spectra of plates 13, 14, and 15. The data of H11 in plate 13, B9, C10, and E10 in plate 14, and D3 and A4 in plate 15 are shown.

plate 15/line G/column 10

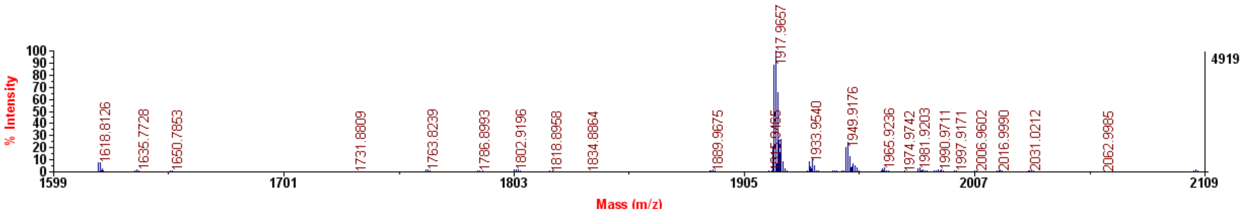

plate 16/line H/column 6

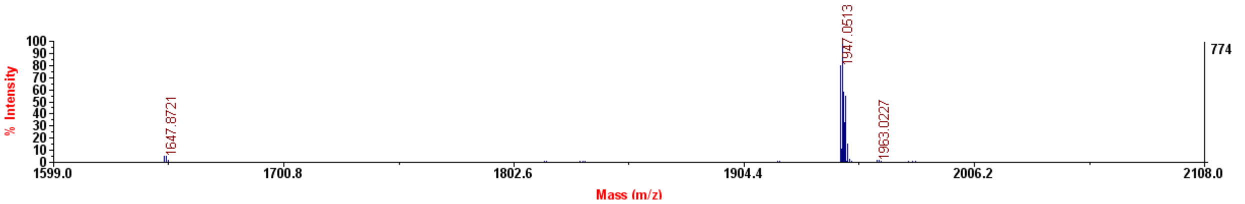

plate 16/line F/column 7

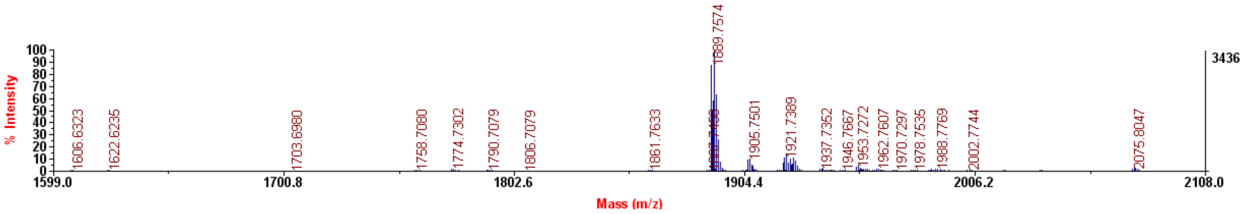

plate 17/line G/column 5

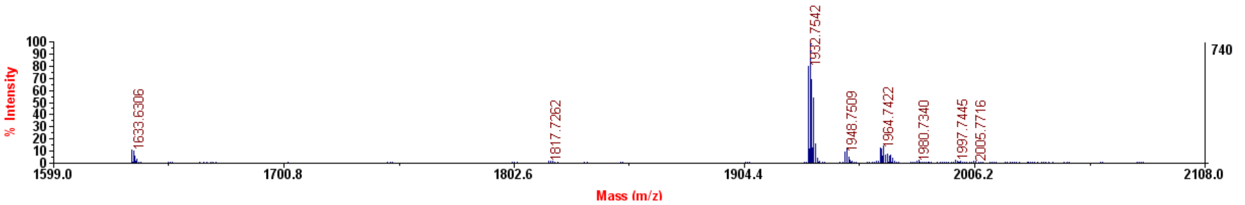

plate 17/line A/column 7

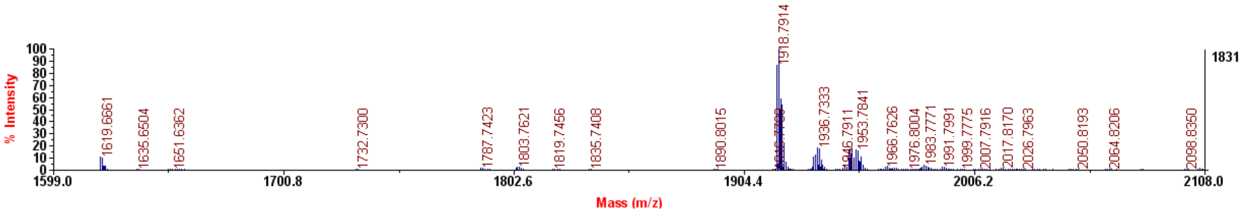

plate 17/line E/column 9

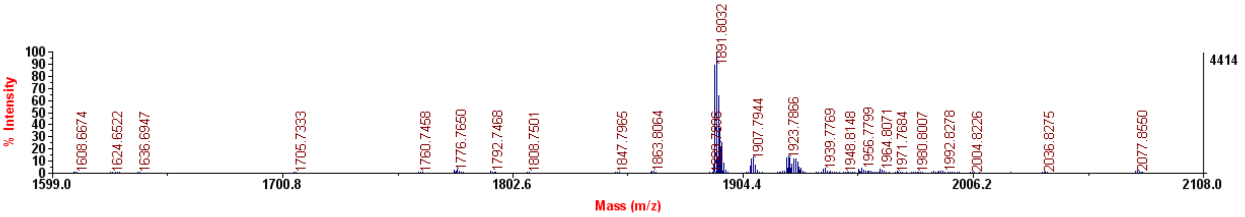

Supplementary Figure 85. MS spectra of plates 15, 16, and 17. The data of G10 in plate 15, H6 and F7 in plate 16, and G5, A7, and E9 in plate 17 are shown.

plate 17/line D/column 10

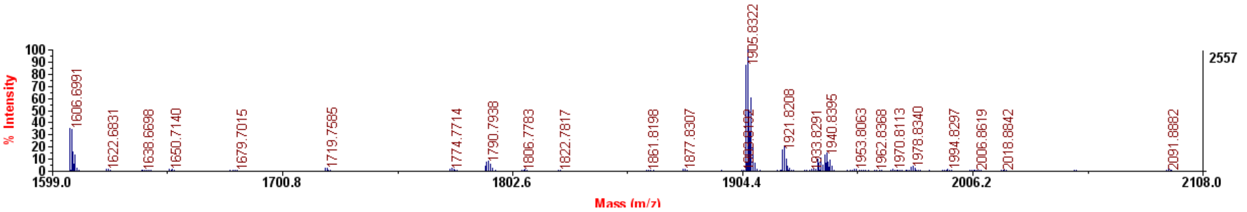

plate 17/line H/column 10

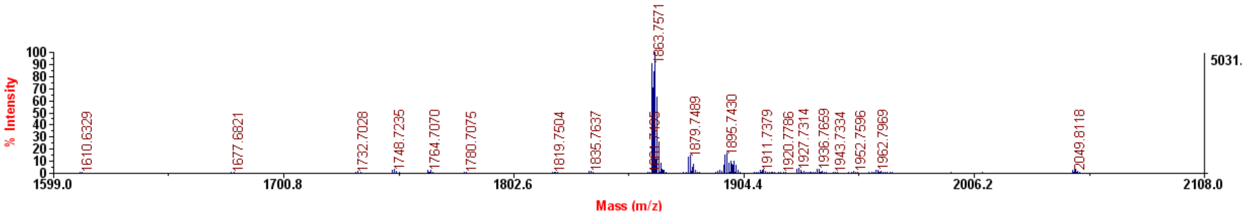

plate 17/line B/column 11

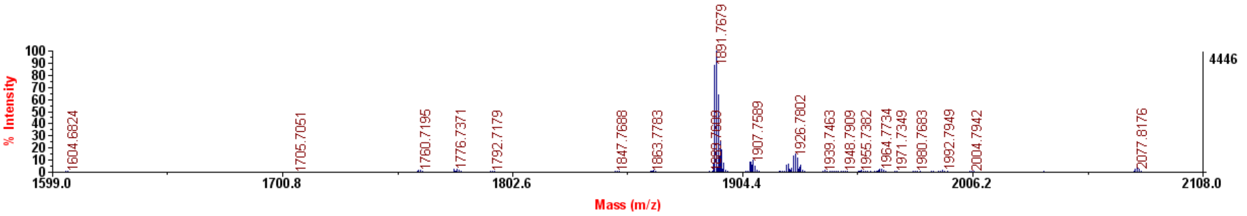

plate 17/line C/column 11

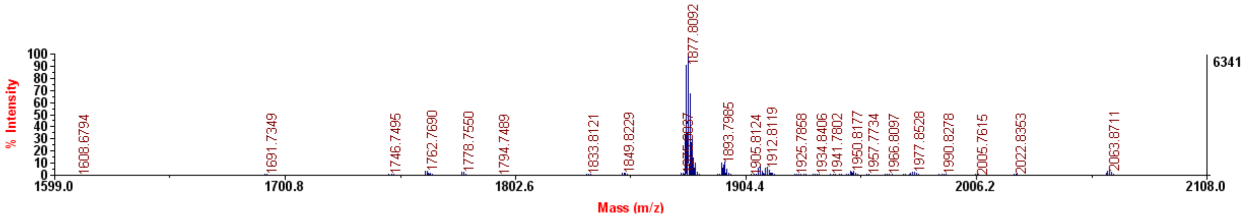

plate 18/line H/column 8

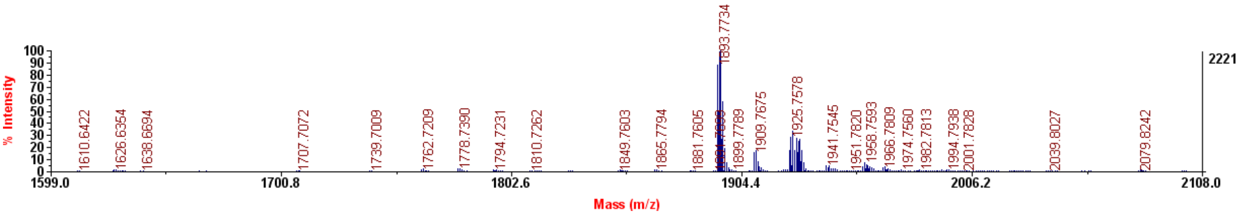

plate 19/line G/column 2

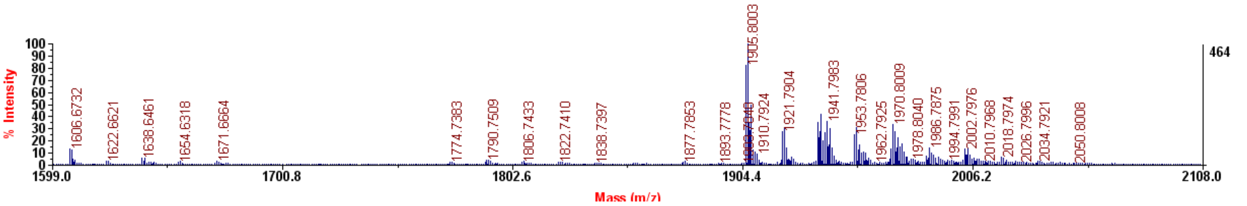

Supplementary Figure 86. MS spectra of plate 17, 18, and 19. The data of D10, H10, B11, and C11 in plate 17, H8 in plate 18, and G2 in plate 19 are shown.

plate 20/line G/column 1

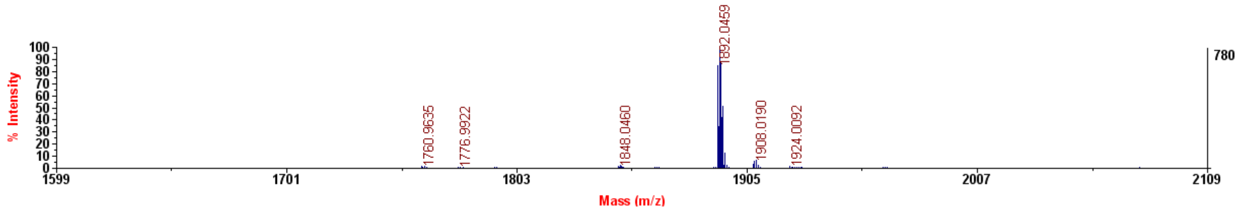

plate 20/line G/column 6

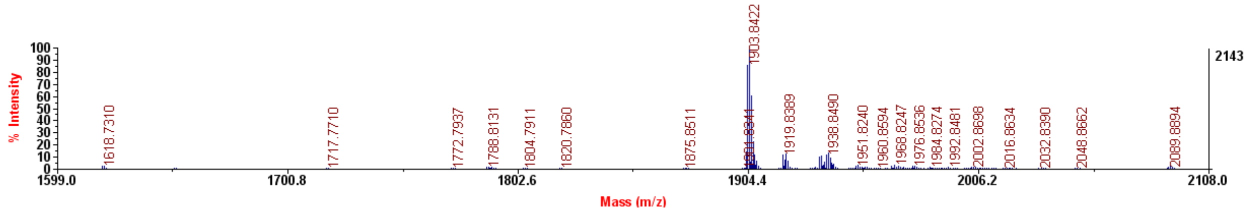

plate 21/line D/column 1

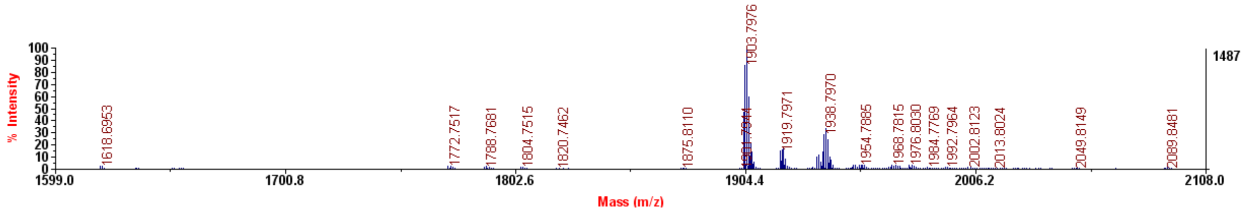

plate 21/line C/column 4

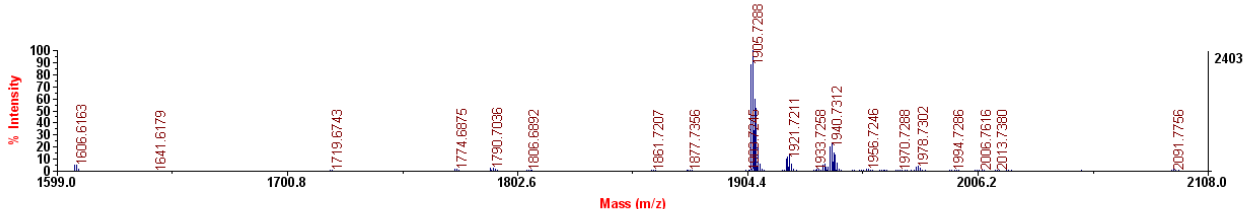

plate 21/line B/column 7

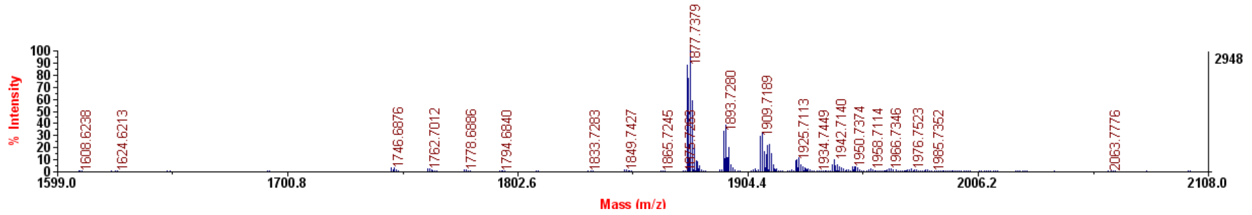

plate 21/line D/column 7

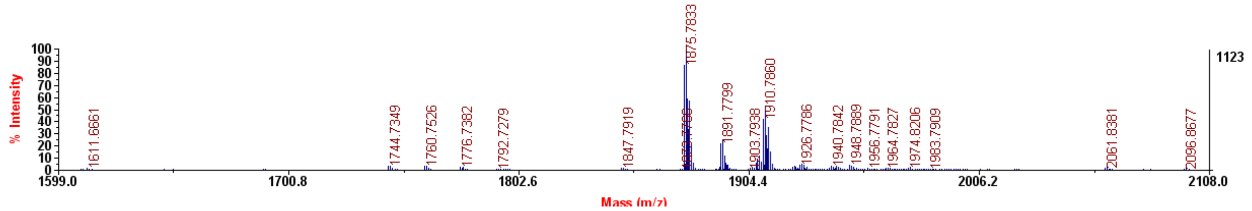

Supplementary Figure 87. MS spectra of plates 20 and 21. The data of G1 and G6 in plate 20 and D1, C4, B7, and D7 in plate 21 are shown.

plate 21/line A/column 8

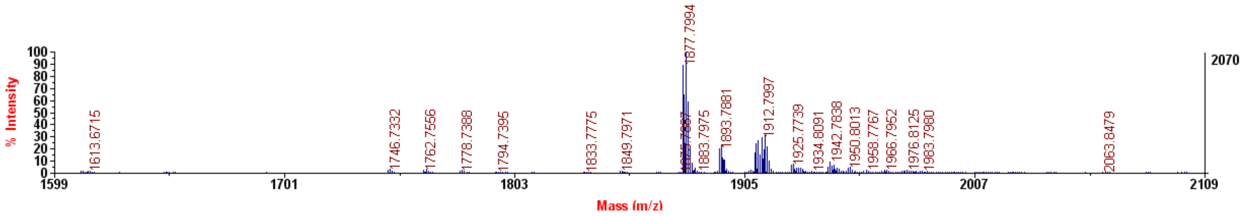

plate 21/line A/column 11

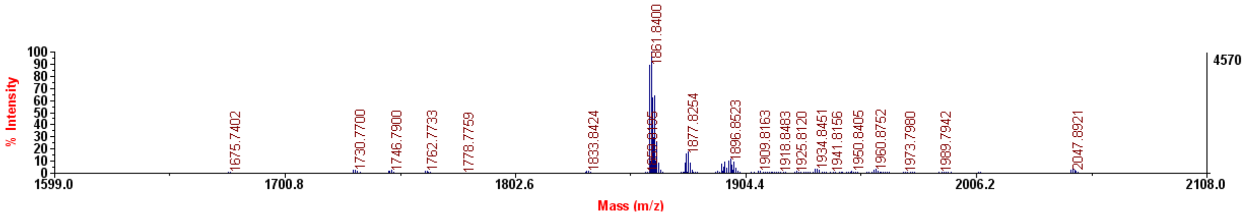

plate 22/line H/column 1

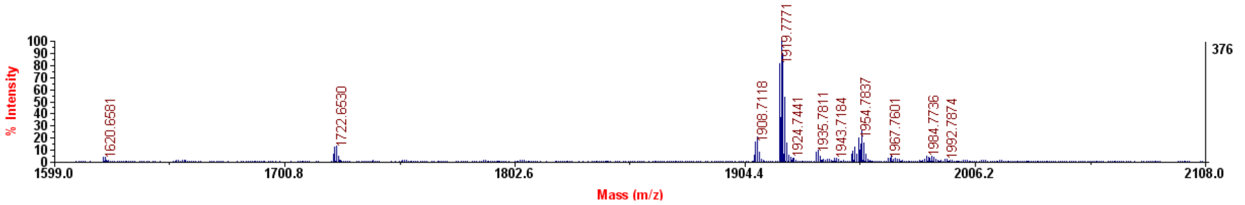

plate 22/line B/column 3

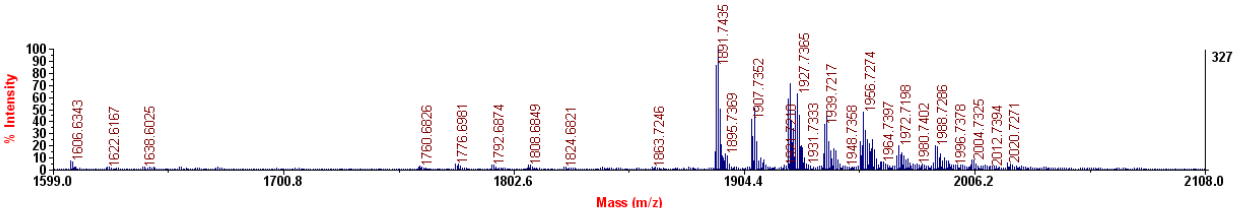

plate 23/line G/column 1

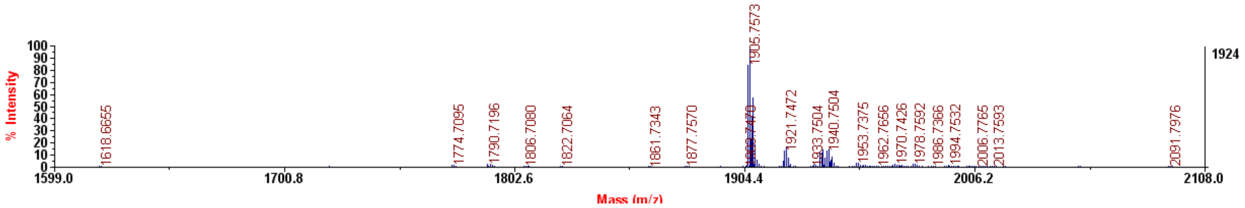

plate 23/line G/column 2

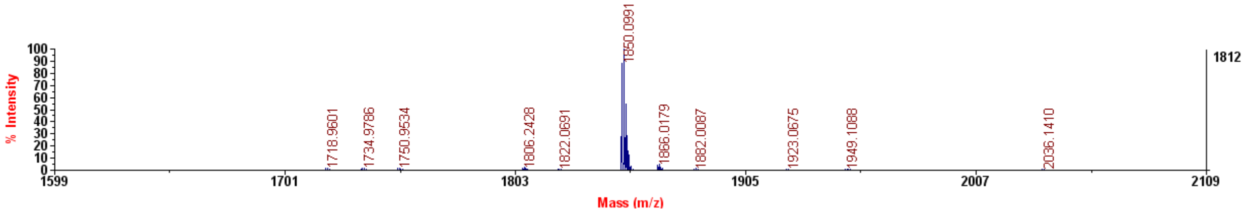

Supplementary Figure 88. MS spectra of plates 21, 22, and 23. The data of A8 and A11 in plate 21, H1 and B3 in plate 22, and G1 and G2 in plate 23 are shown.

plate 23/line D/column 7

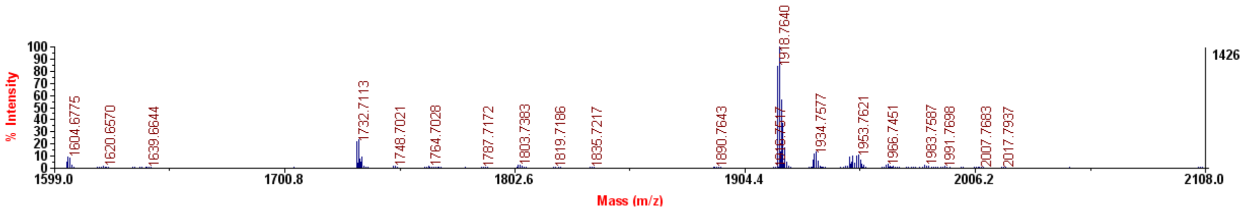

plate 24/line B/column 2

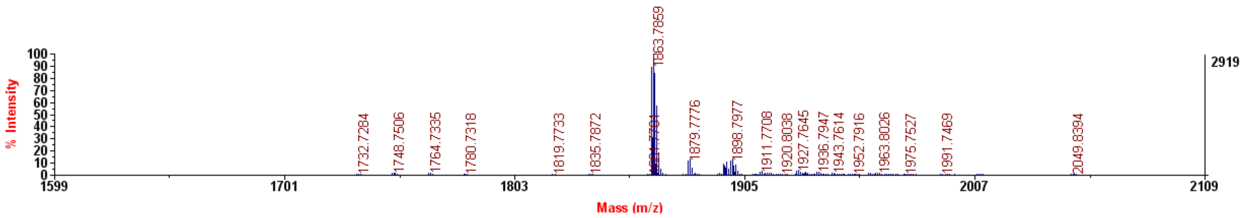

plate 24/line B/column 4

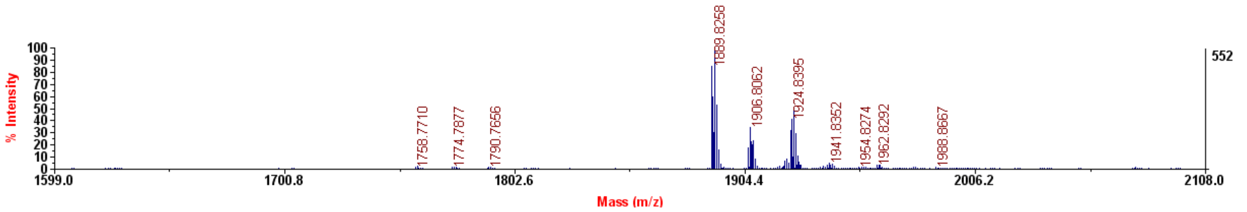

plate 24/line E/column 4

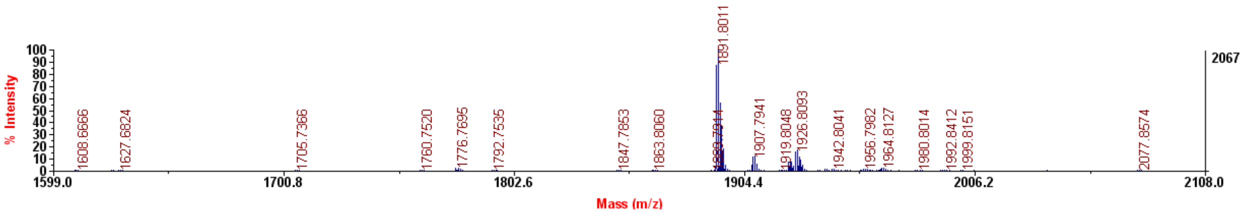

plate 24/line E/column 8

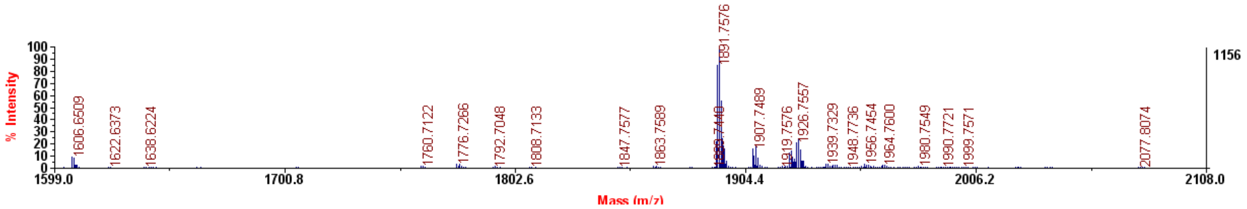

plate 24/line F/column 8

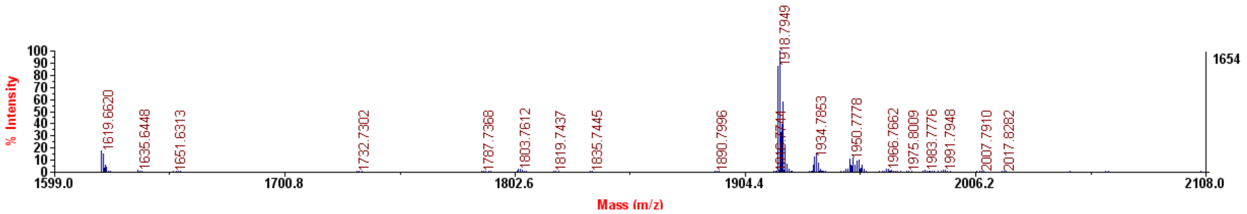

Supplementary Figure 89. MS spectra of plates 23 and 24. The data of D7 in plate 23 and B2, B4, E4, E8, and F8 in plate 24 are shown.

plate 24/line H/column 8

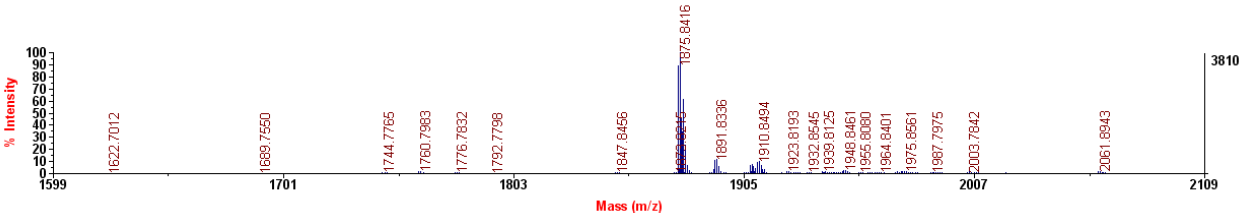

plate 24/line C/column 11

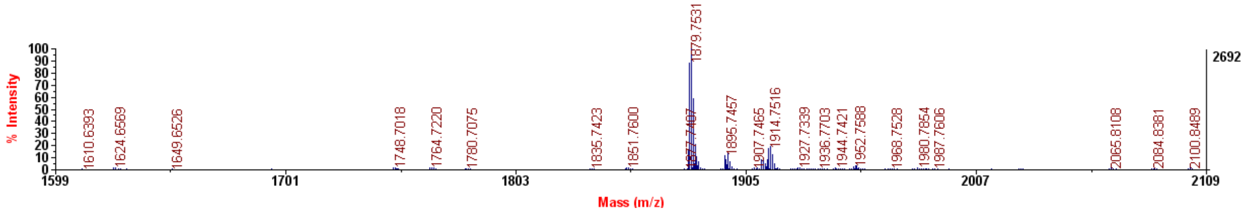

plate 25/line B/column 2

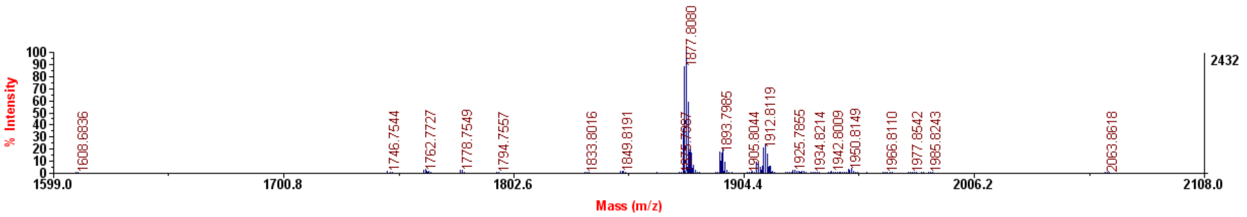

plate 25/line F/column 2

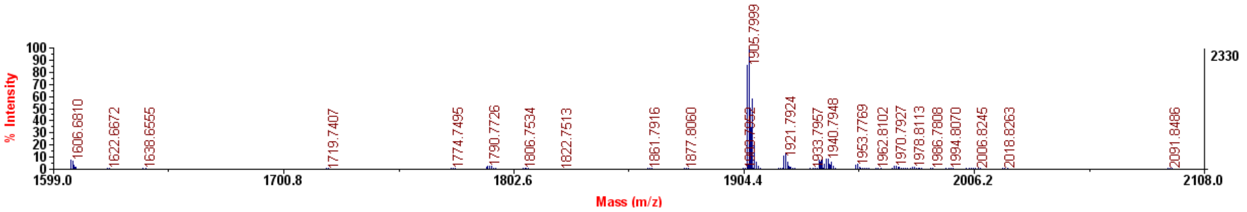

plate 25/line D/column 4

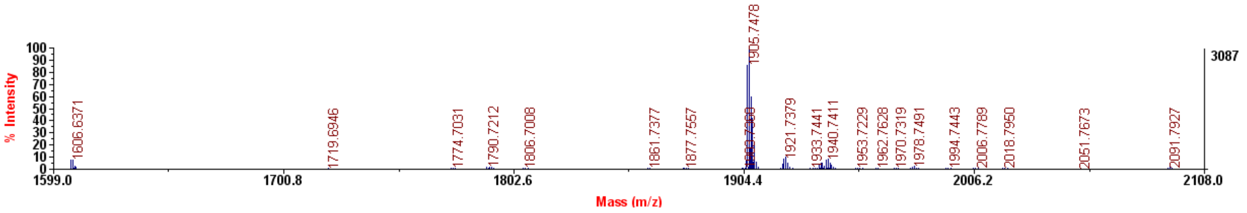

plate 25/line F/column 5

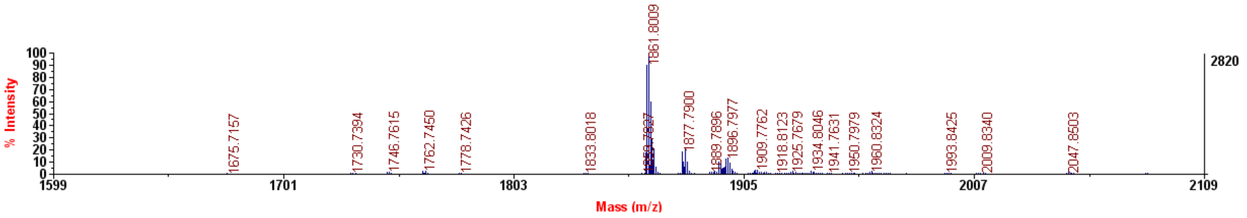

Supplementary Figure 90. MS spectra of plate 24 and 25. The data of H8 and C11 in plate 24 and B2, F2, D4, and F5 in plate 25 are shown.

plate 25/line C/column 7

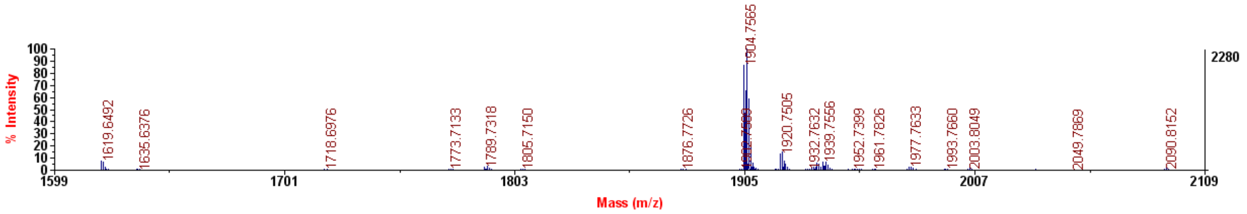

plate 25/line E/column 8

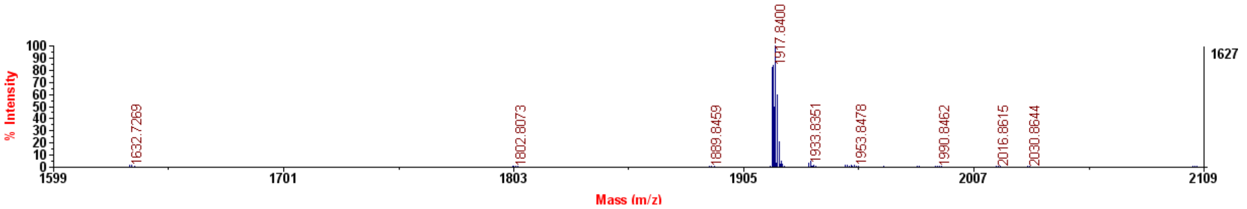

plate 25/line G/column 8

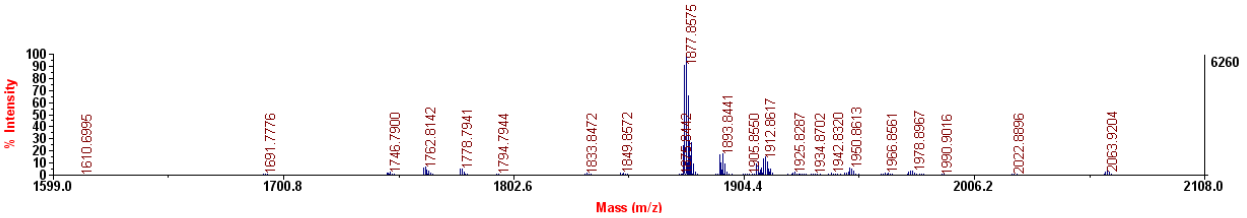

plate 25/line D/column 9

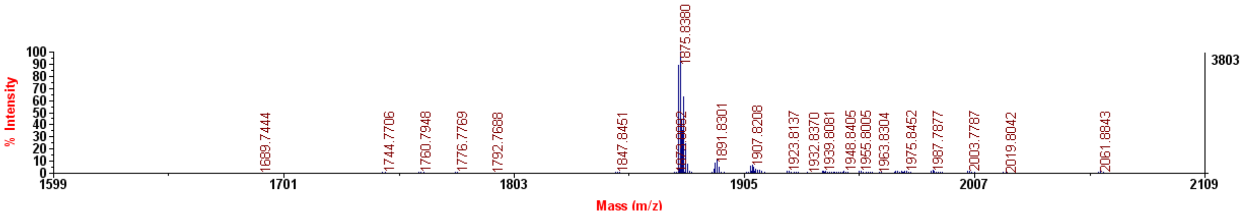

plate 25/line B/column 10

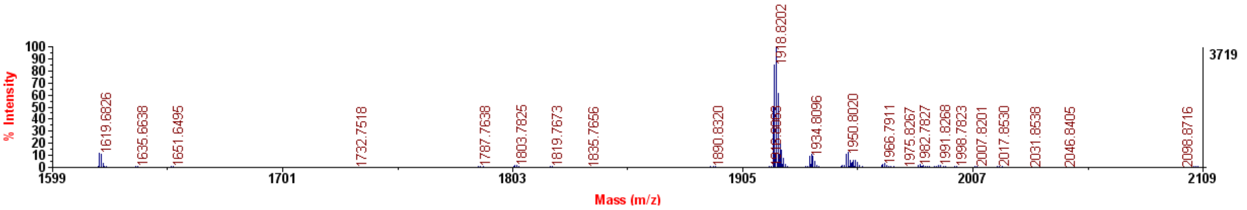

plate 26/line C/column 1

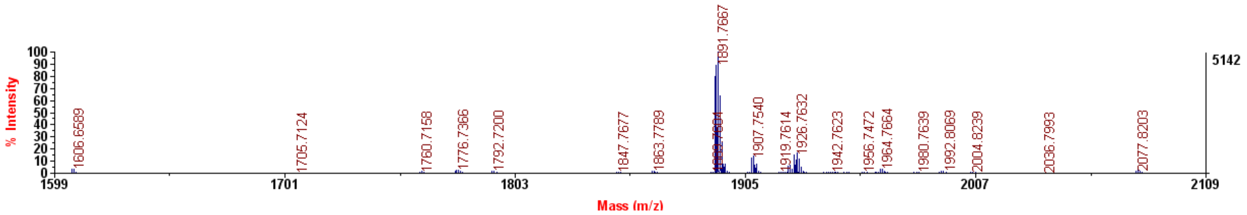

Supplementary Figure 91. MS spectra of plates 25 and 26. The data of C7, E8, G8, D9, and B10 in plate 25 and C1 in plate 26 are shown.

plate 26/line D/column 1

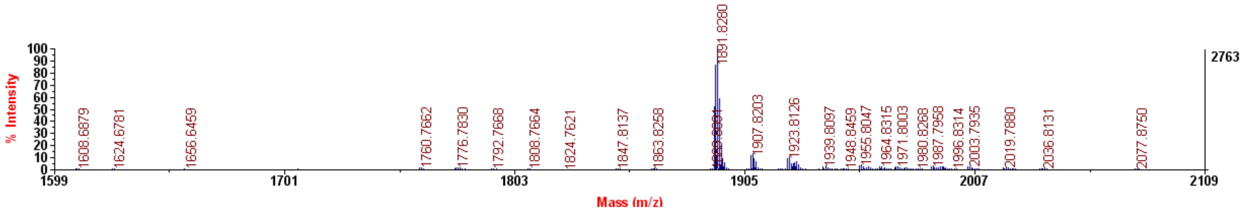

plate 26/line B/column 2

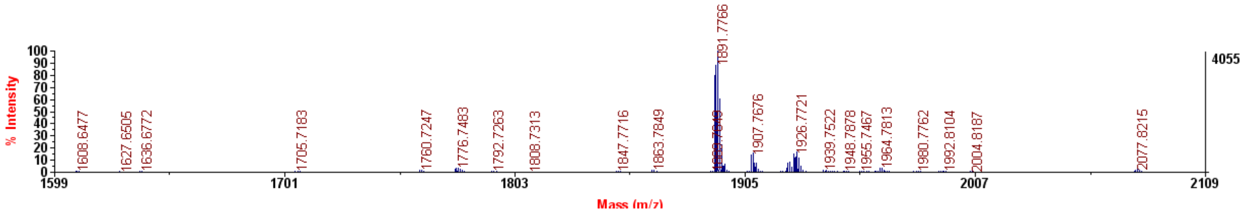

plate 26/line C/column 7

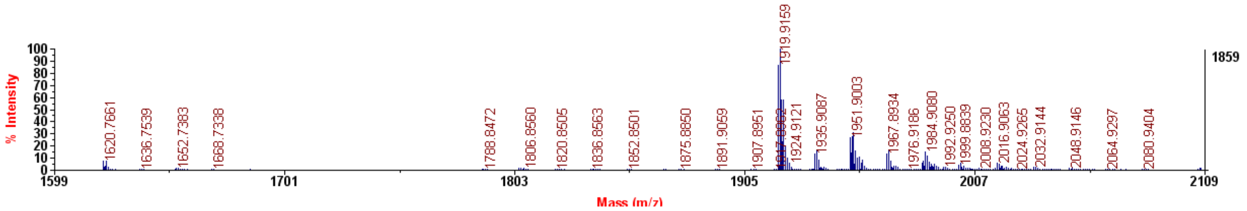

plate 26/line B/column 8

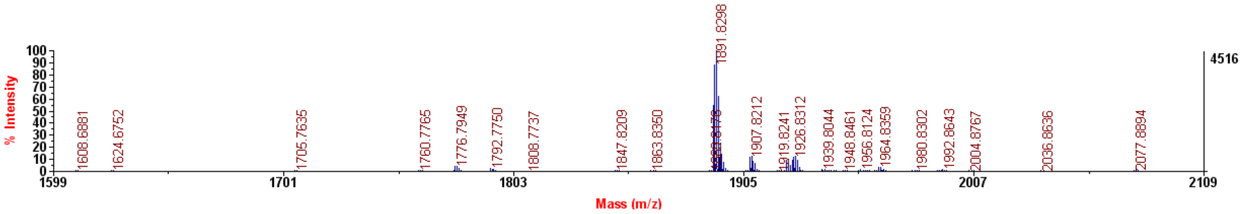

plate 27/line B/column 4

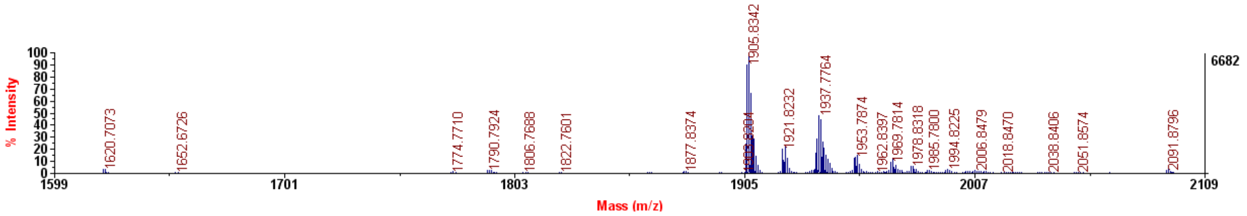

plate 28/line A/column 3

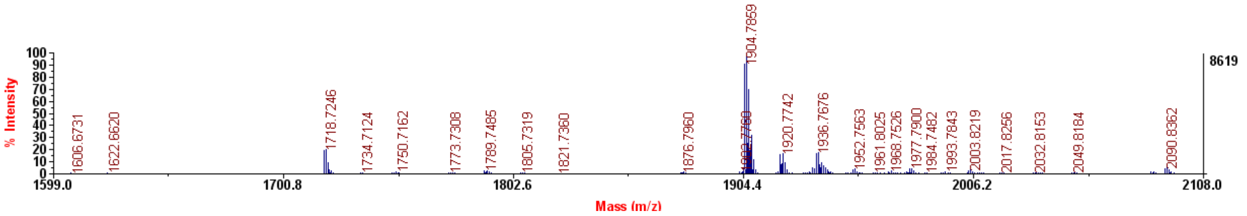

Supplementary Figure 92. MS spectra of plates 26, 27 and 28. The data of D1, B2, C7, and B8 in plate 26, B4 in plate 27, and A3 in plate 28 are shown.

plate 28/line H/column 3

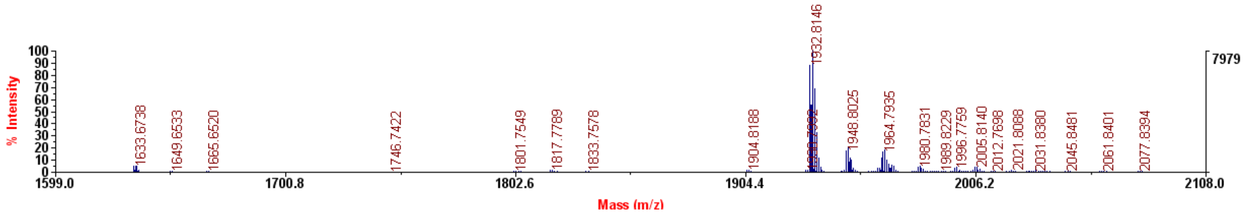

plate 28/line D/column 5

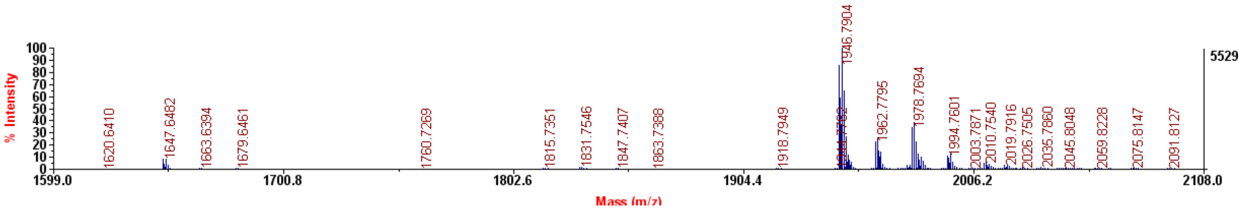

plate 28/line C/column 9

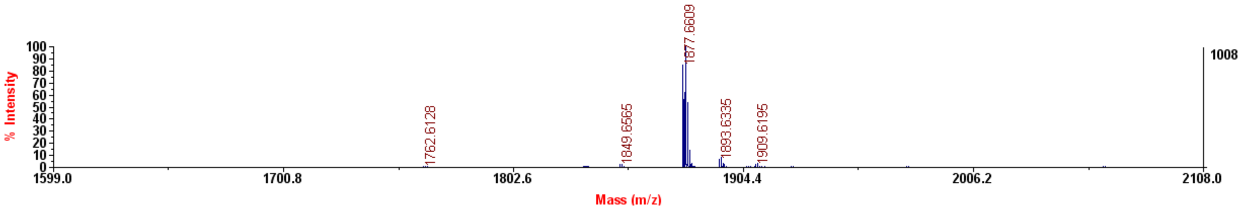

plate 28/line G/column 11

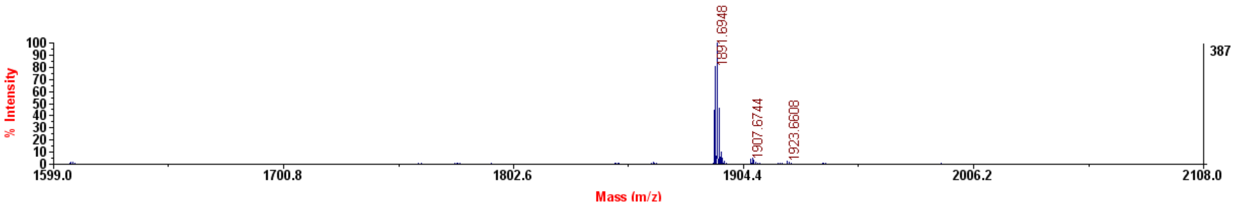

plate 29/line F/column 4

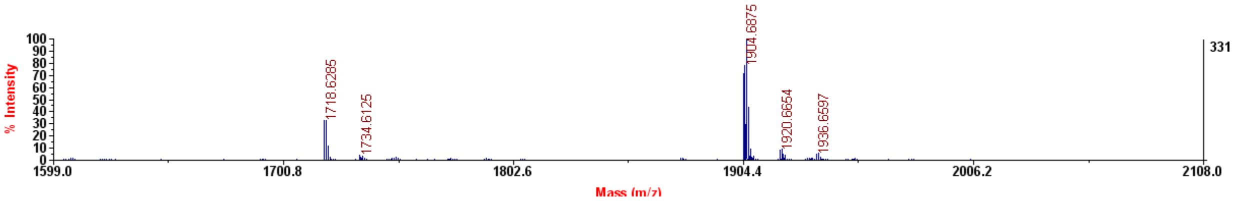

plate 29/line D/column 5

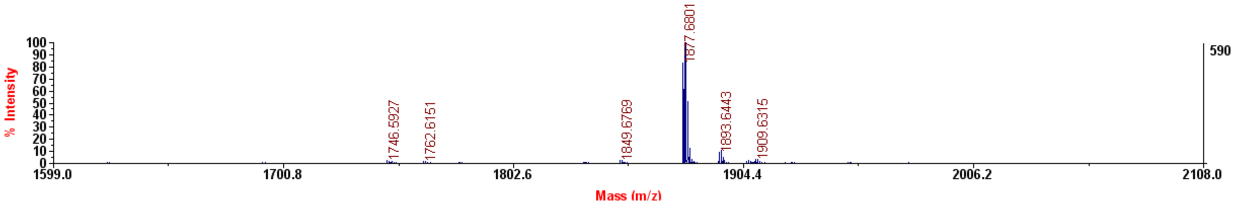

Supplementary Figure 93. MS spectra of plates 28 and 29. The data of H3, D5, C9, and G11 in plate 28 and F4 and D5 in plate 29 are shown.

plate 29/line B/column 8

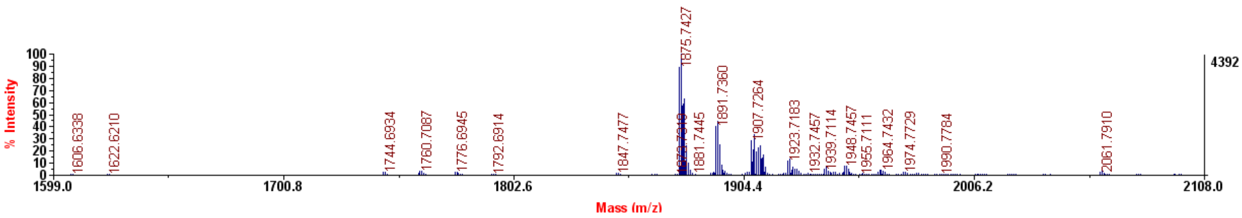

plate 29/line E/column 8

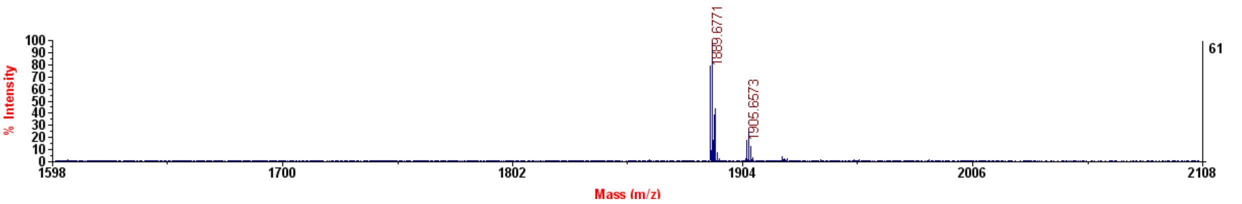

plate 29/line D/column 11

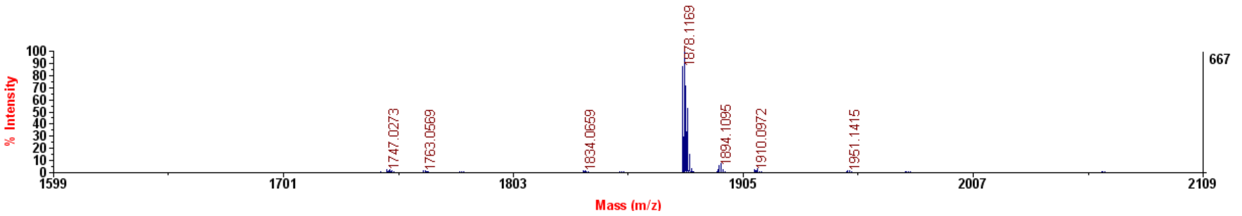

plate 31/line B/column 2

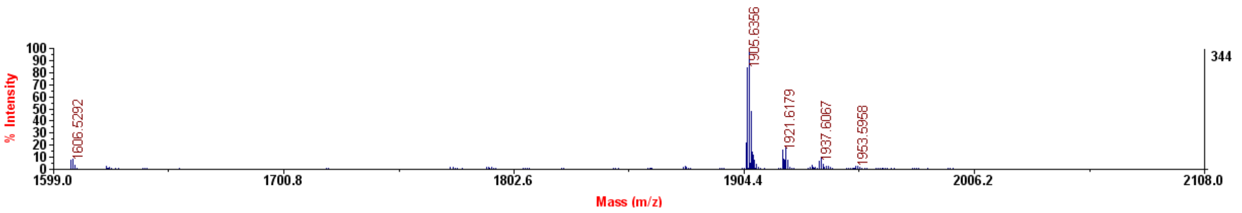

plate 31/line F/column 6

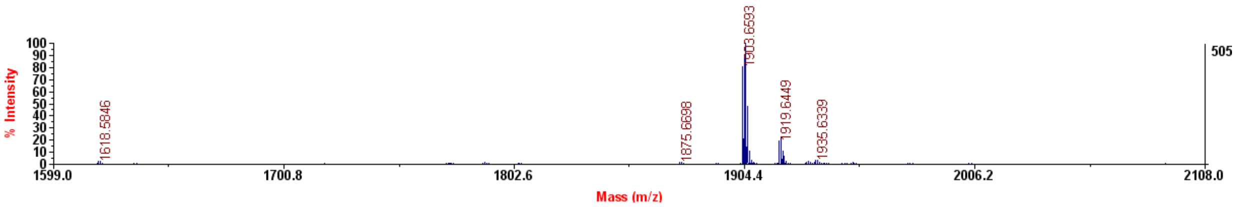

plate 31/line G/column 7

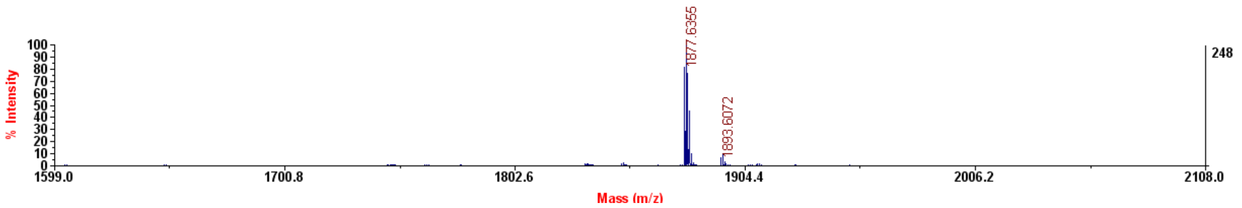

Supplementary Figure 94. MS spectra of plates 29 and 31. The data of B8, E8, and D11 in plate 29 and B2, F6, and G7 in plate 31 are shown.

plate 32/line D/column 1

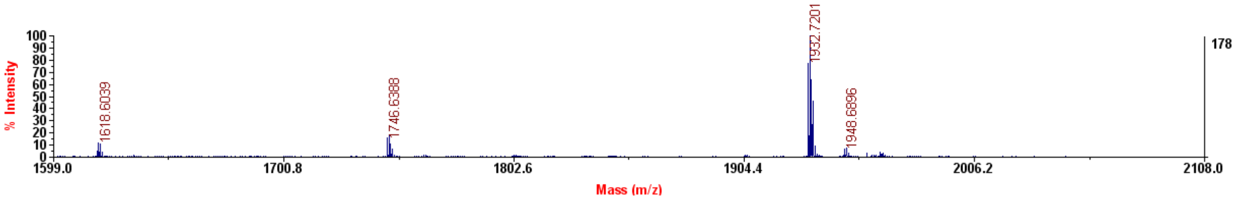

plate 32/line A/column 5

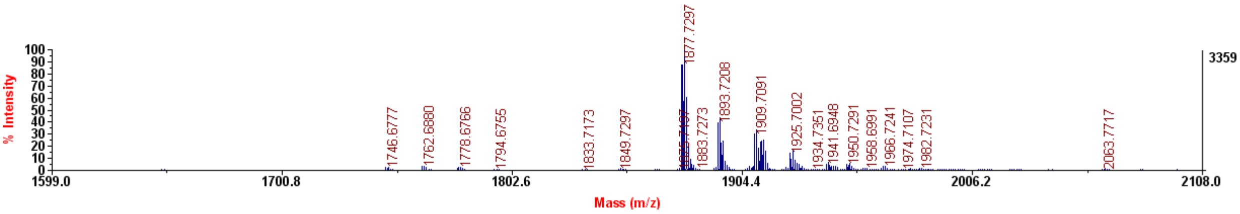

plate 32/line D/column 9

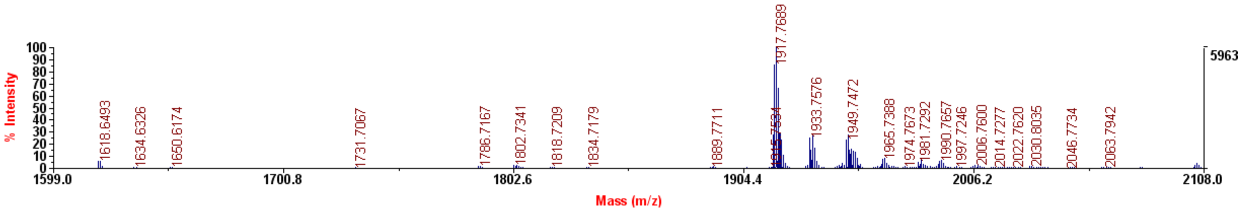

plate 32/line E/column 9

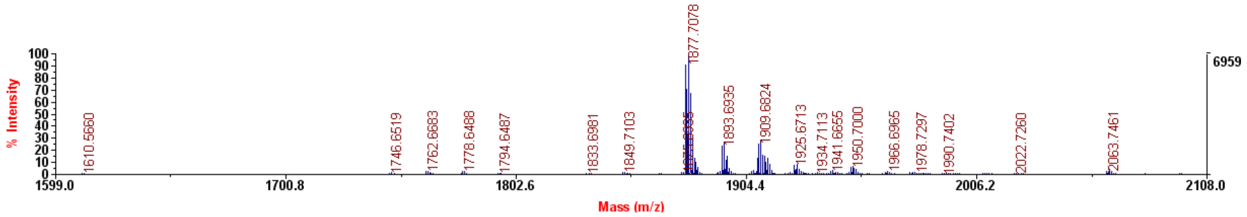

plate 32/line H/column 9

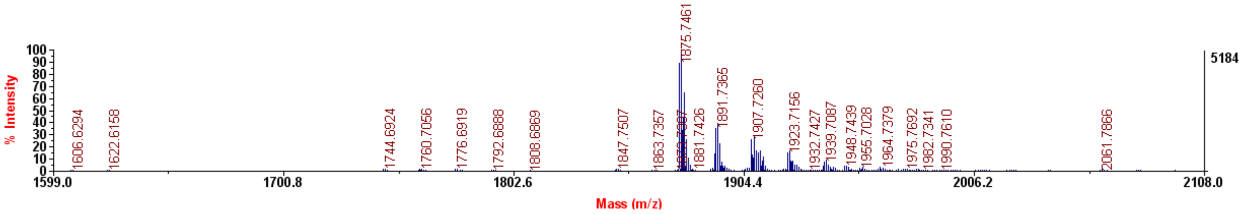

plate 33/line A/column 5

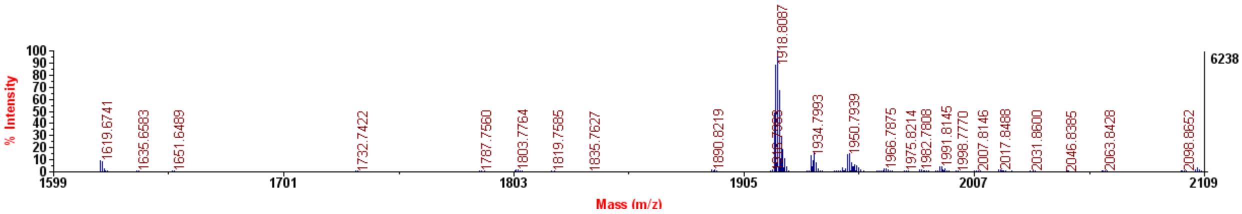

Supplementary Figure 95. MS spectra of plates 32 and 33. The data of D1, A5, D9, E9, and H9 in plate 32 and A5 in plate 33 are shown.

plate 33/line G/column 5

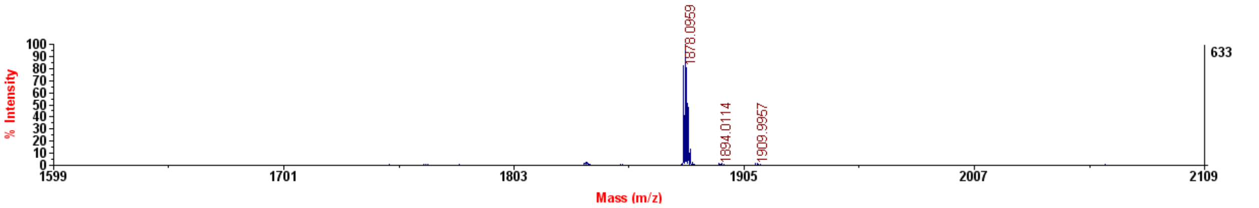

plate 33/line G/column 6

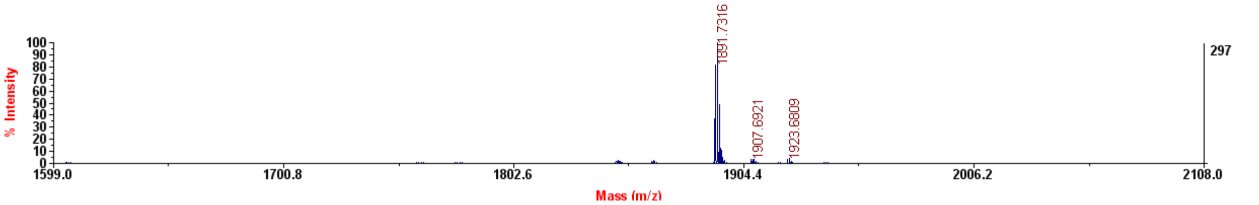

plate 33/line B/column 7

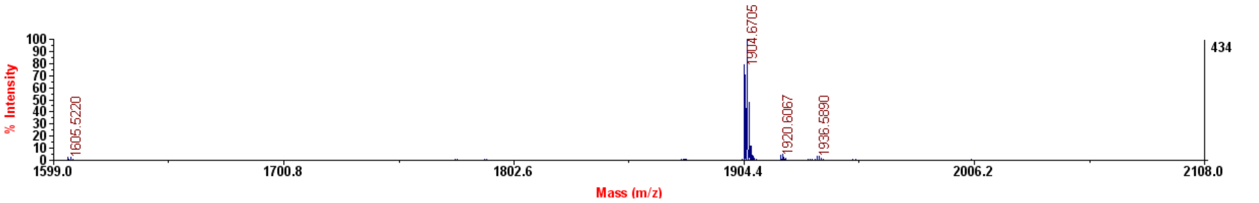

plate 33/line H/column 7

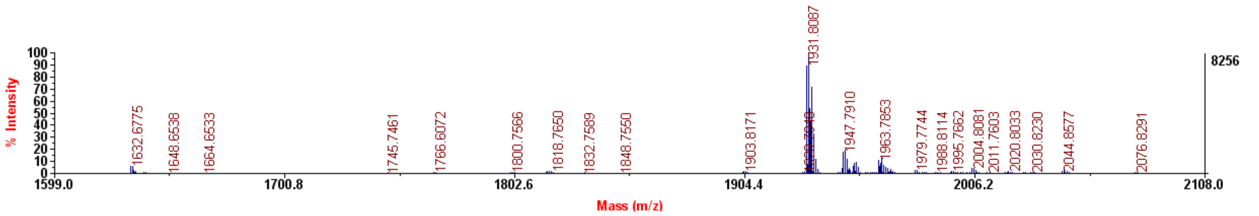

plate 33/line G/column 8

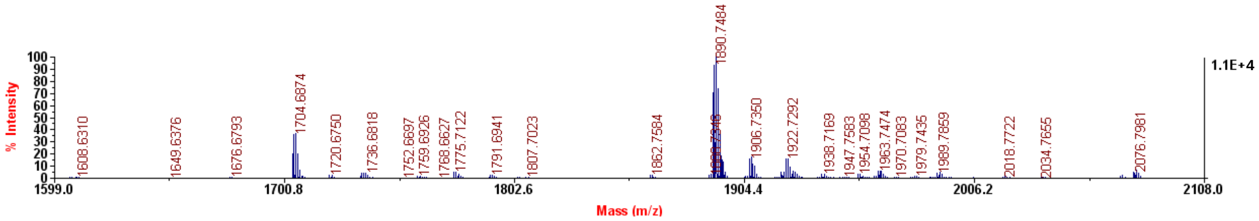

plate 33/line C/column 11

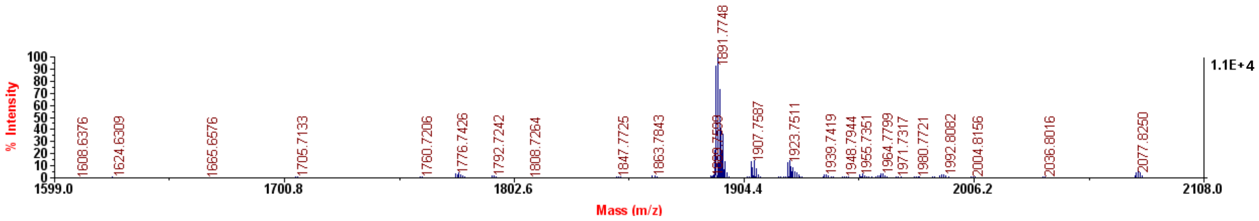

Supplementary Figure 96. MS spectra of plate 33. The data of G5, G6, B7, H7, G8, and C11 in plate 33 are shown.

plate 33/line D/column 11

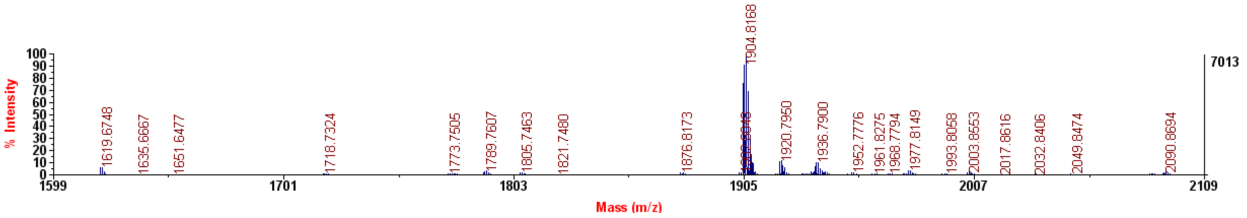

plate 34/line G/column 2

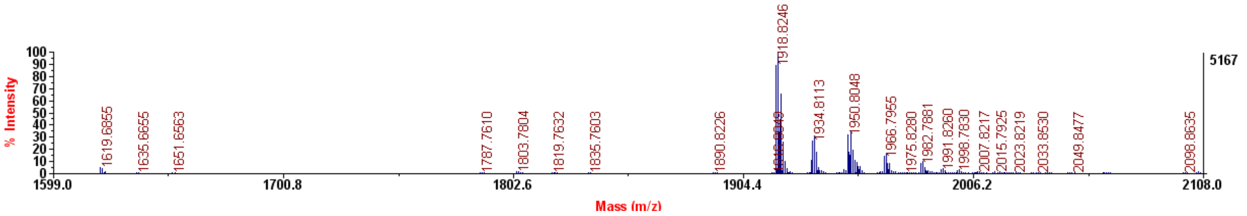

plate 34/line A/column 3

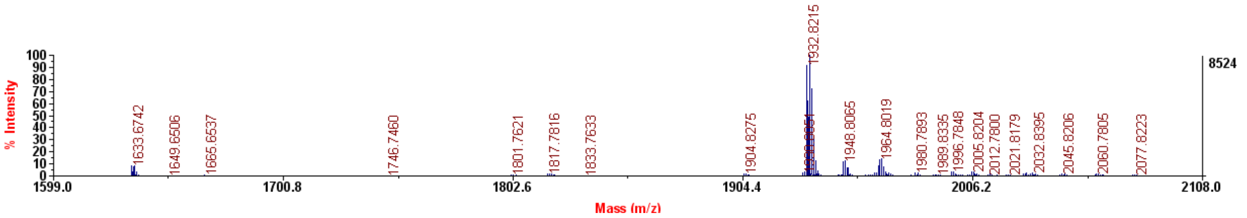

plate 34/line G/column 4

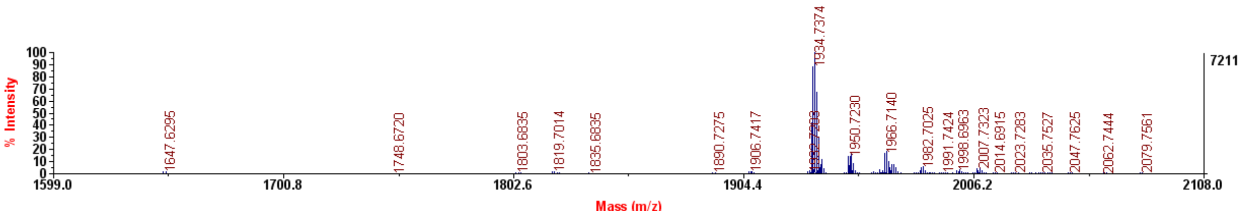

plate 34/line B/column 6

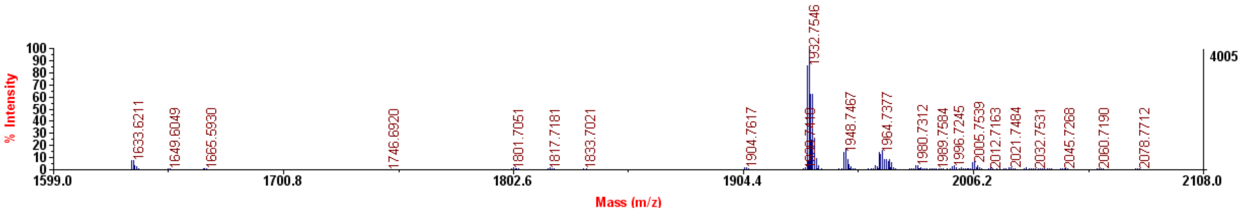

plate 34/line B/column 8

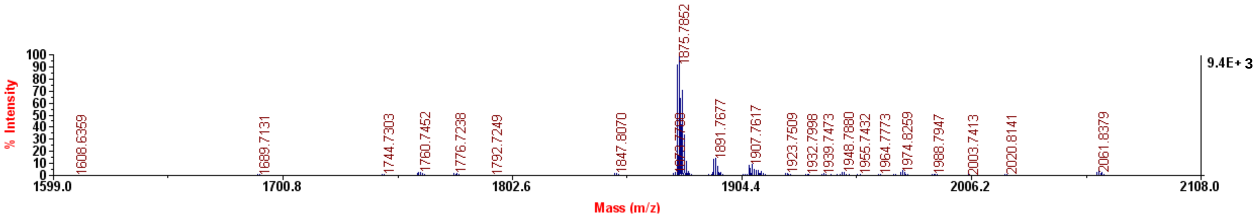

Supplementary Figure 97. MS spectra of plates 33 and 34. The data of D11 in plate 33 and G2, A3, G4, B6, and B8 in plate 34 are shown.

plate 34/line C/column 11

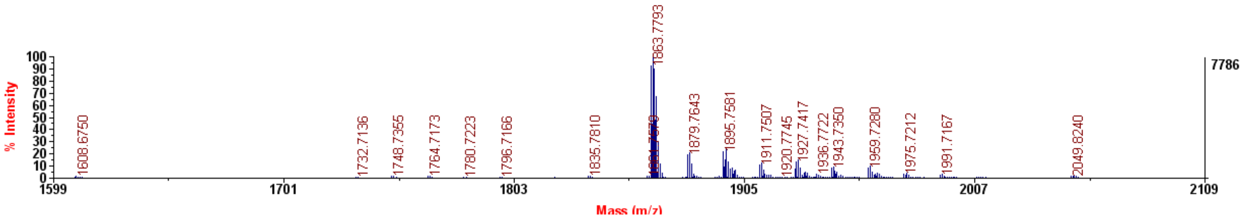

plate 35/line G/column 11

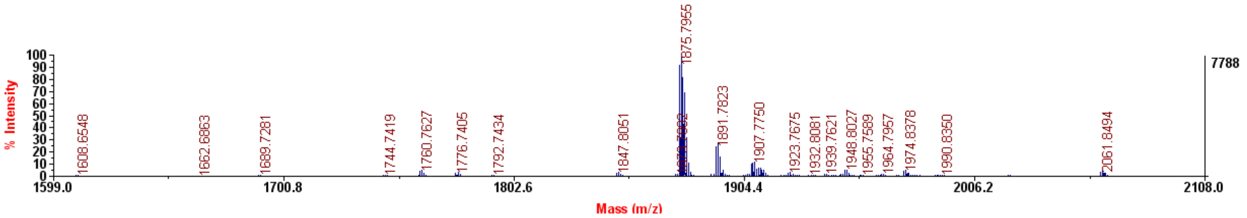

plate 36/line D/column 2

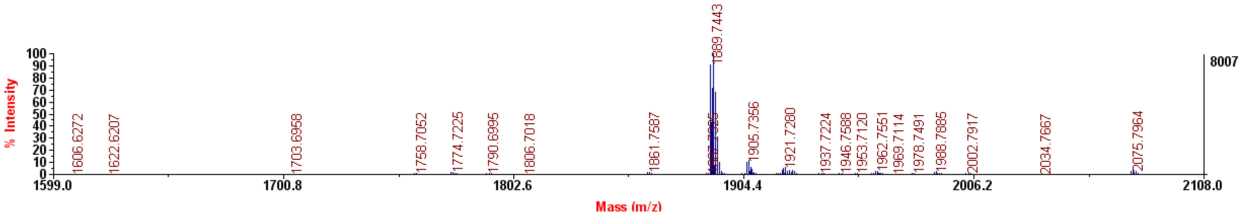

plate 36/line B/column 3

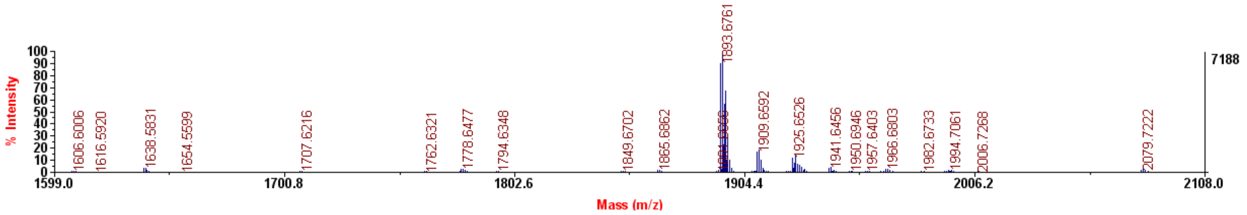

plate 36/line G/column 4

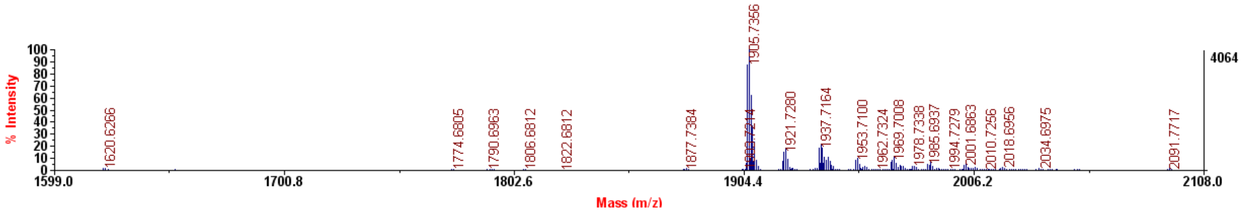

plate 36/line H/column 4

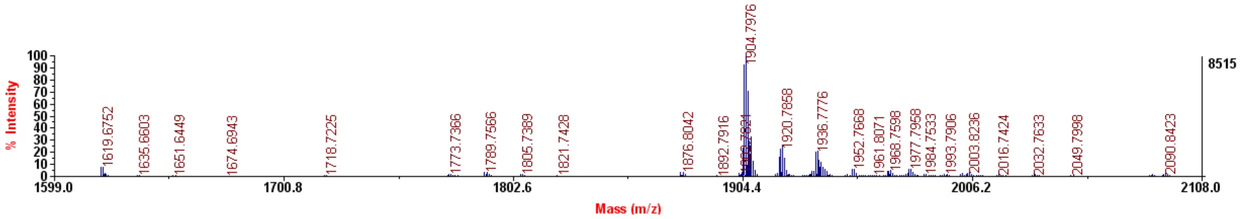

Supplementary Figure 98. MS spectra of plates 34, 35, and 36. The data of C11 in plate 34, G11 in plate 35, and D2, B3, G4, and H4 in plate 36 are shown.

plate 36/line A/column 7

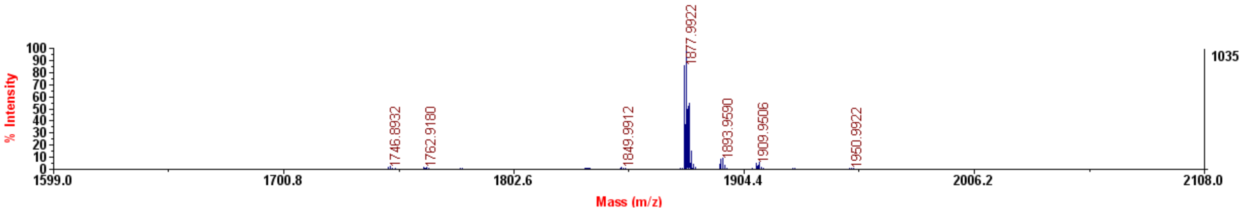

plate 37/line E/column 1

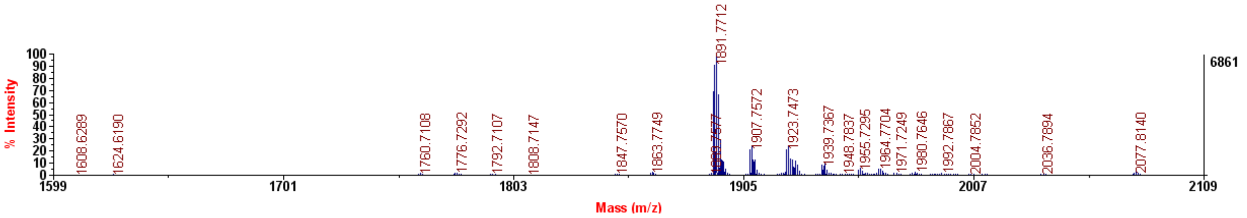

plate 37/line F/column 1

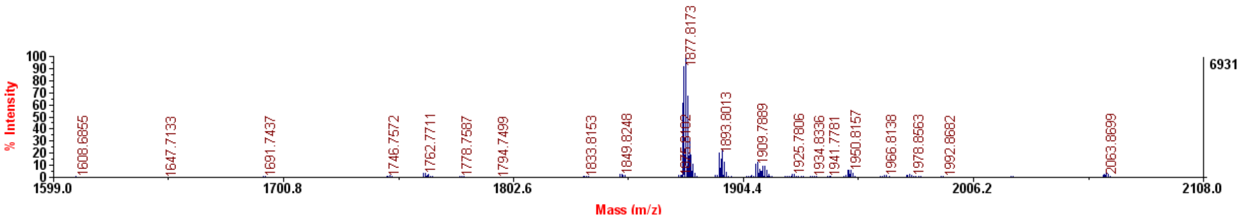

plate 37/line B/column 4

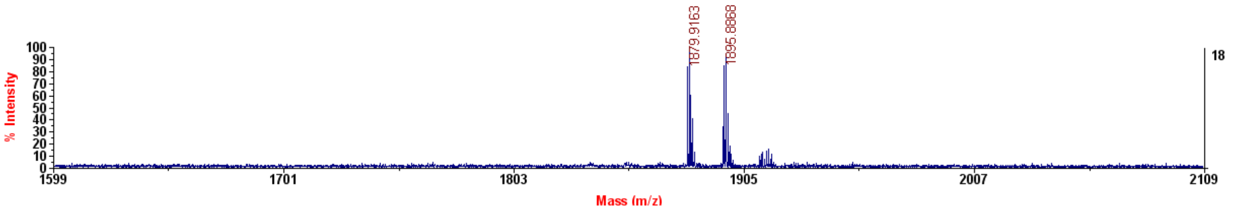

plate 37/line D/column 6

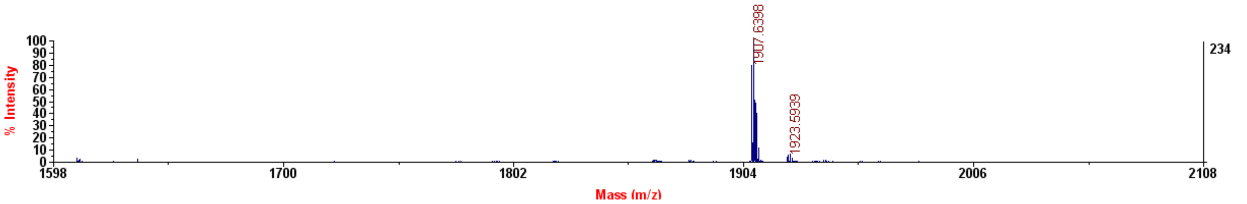

plate 37/line G/column 6

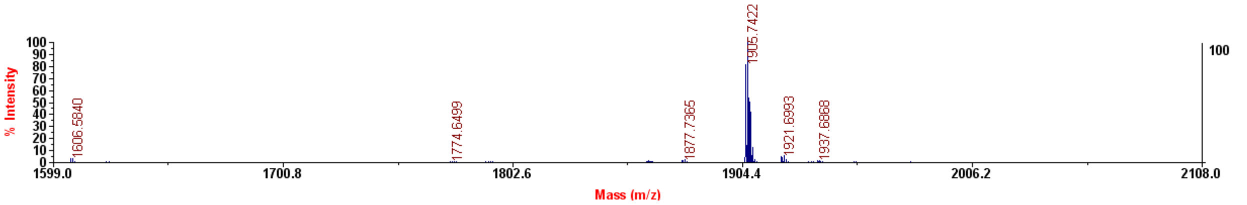

Supplementary Figure 99. MS spectra of plates 36 and 37. The data of A7 in plate 36 and E1, F1, B4, D6, and G6 in plate 37 are shown.

plate 37/line G/column 7

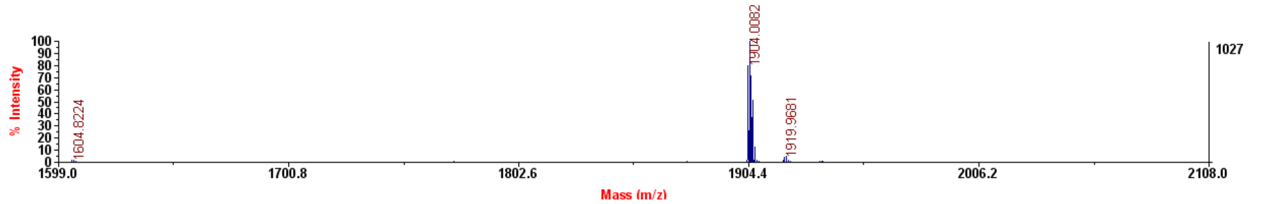

plate 37/line D/column 8

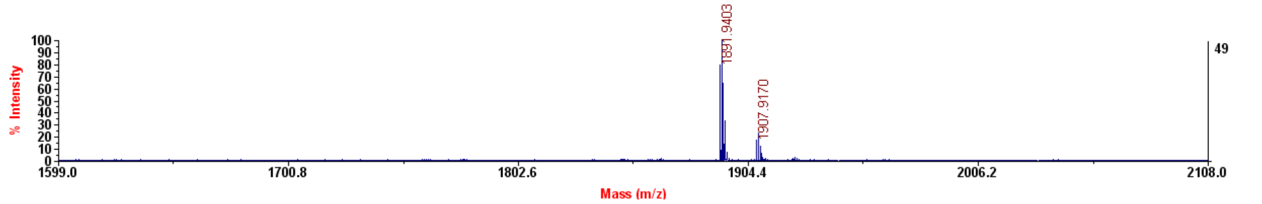

plate 37/line H/column 10

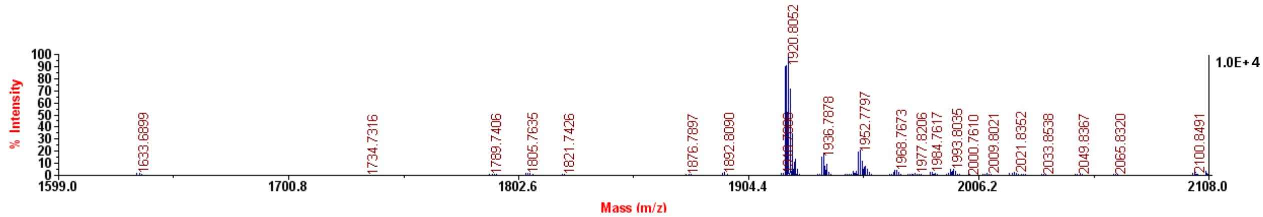

plate 38/line D/column 3

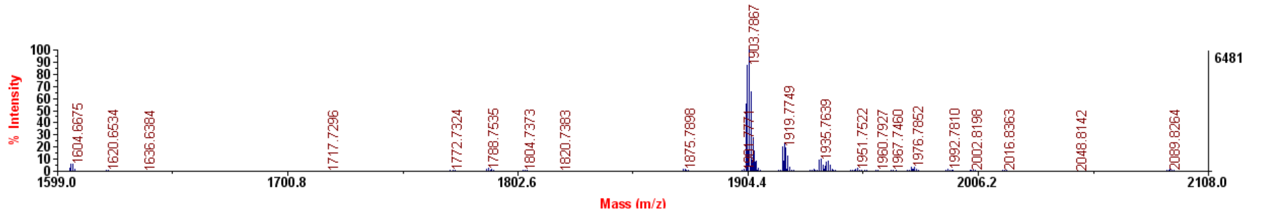

plate 38/line G/column 3

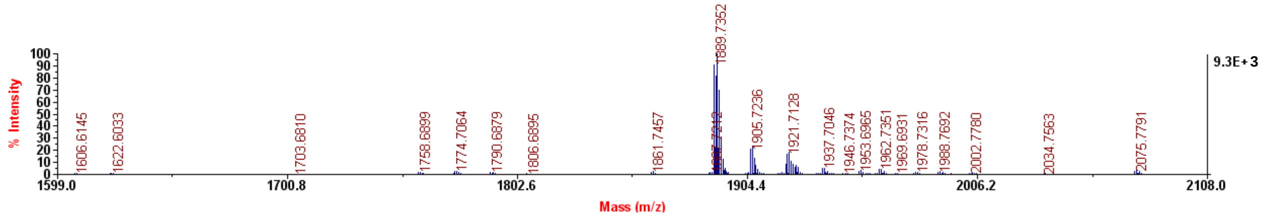

plate 38/line H/column 5

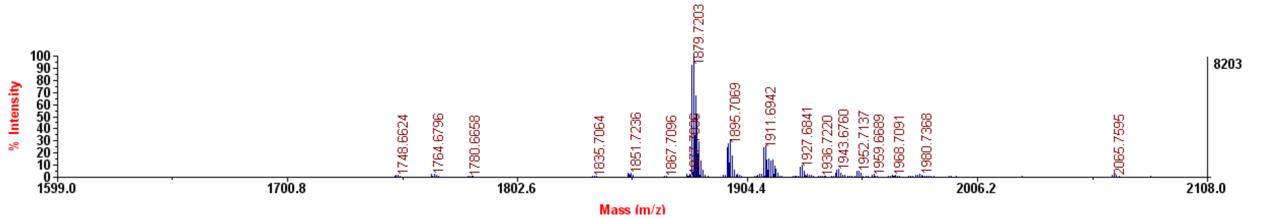

Supplementary Figure 100. MS spectra of plates 37 and 38. The data of G7, D8, and H10 in plate 37 and D3, G3, and H5 in plate 38 are shown.

plate 38/line A/column 6

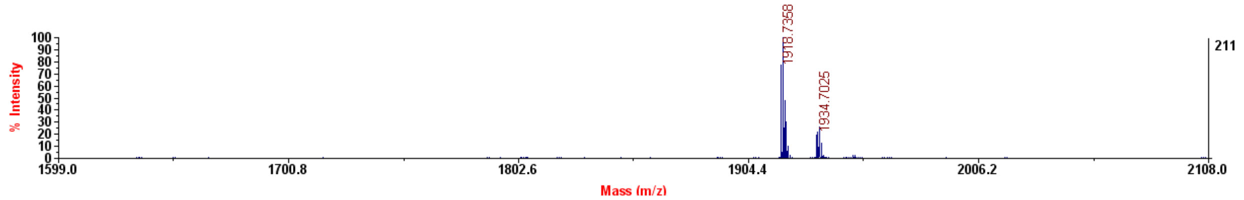

plate 38/line D/column 10

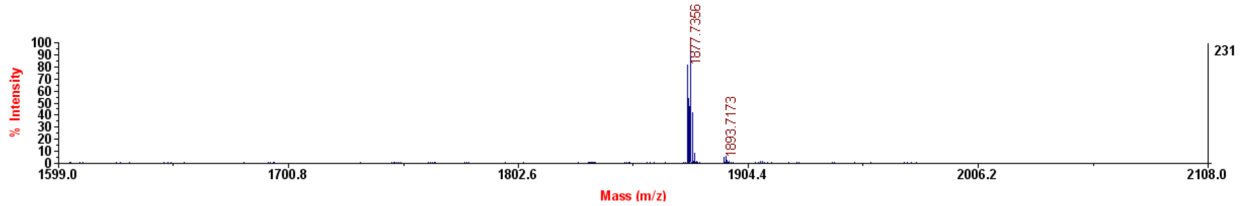

plate 38/line E/column 10

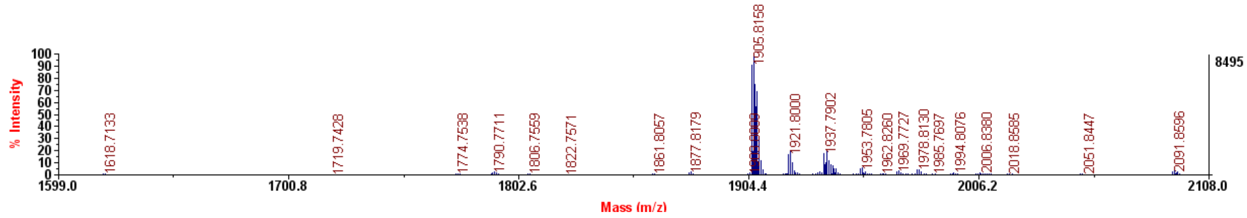

plate 38/line A/column 11

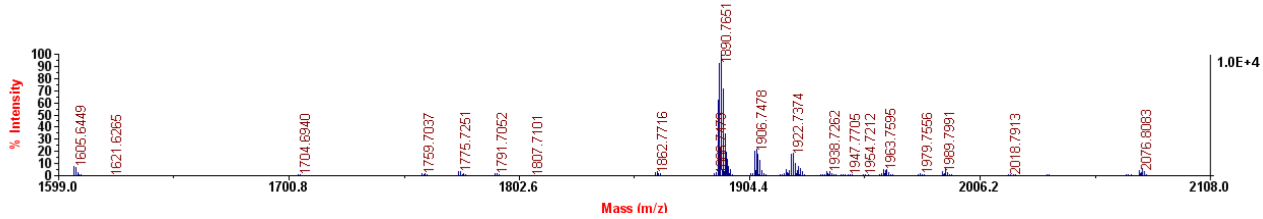

plate 38/line G/column 11

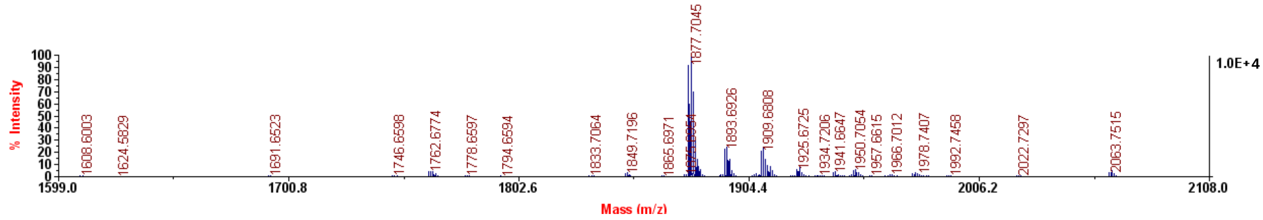

plate 39/line E/column 8

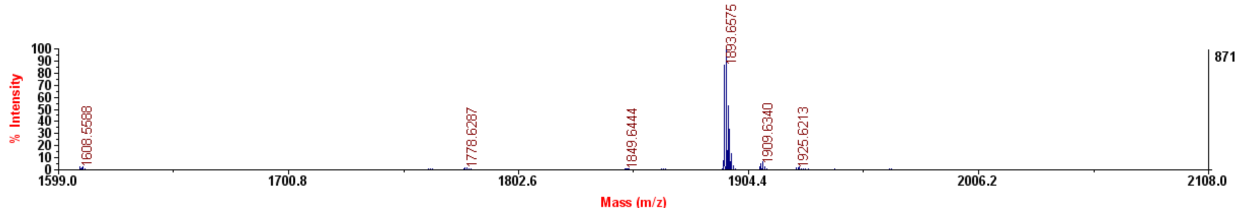

Supplementary Figure 101. MS spectra of plates 38 and 39. The data of A6, D10, E10, A11, and G11 in plate 38 and E8 in plate 39 are shown.

plate 40/line D/column 4

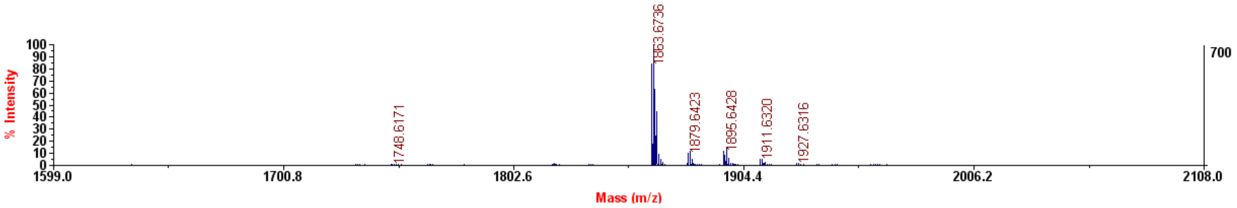

plate 40/line C/column 5

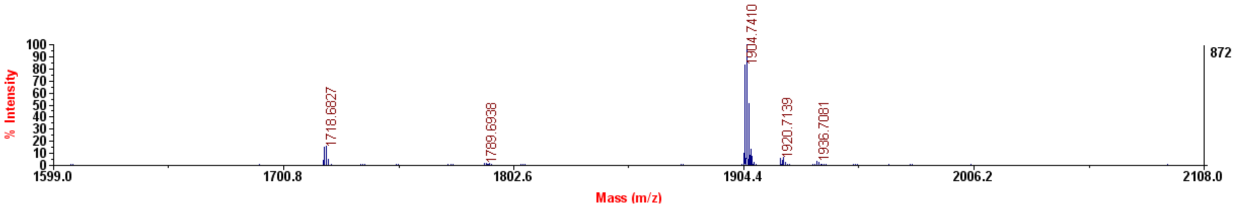

plate 40/line D/column 11

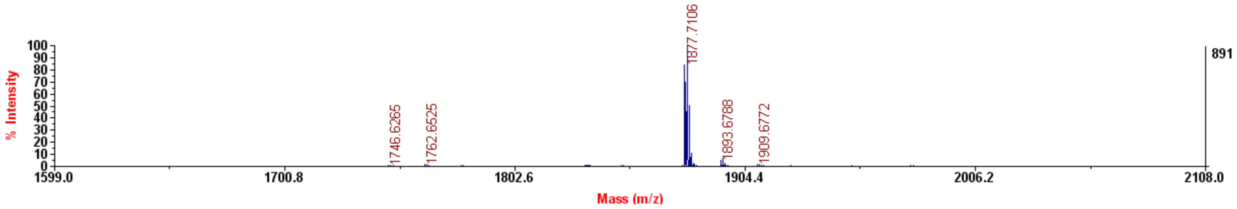

plate 41/line H/column 4

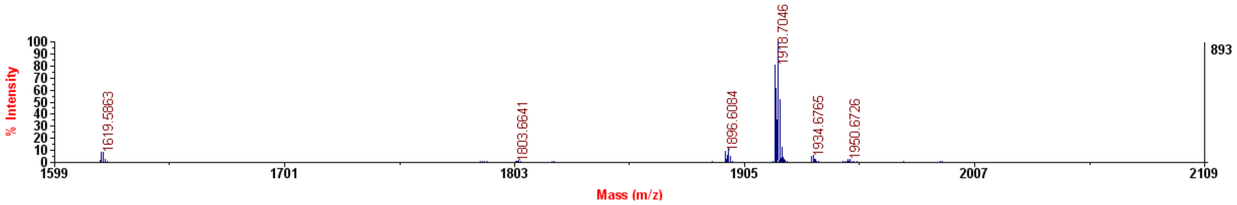

plate 41/line D/column 6

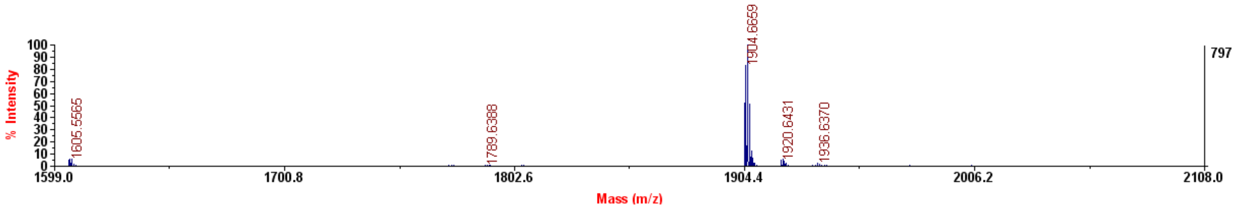

plate 41/line G/column 10

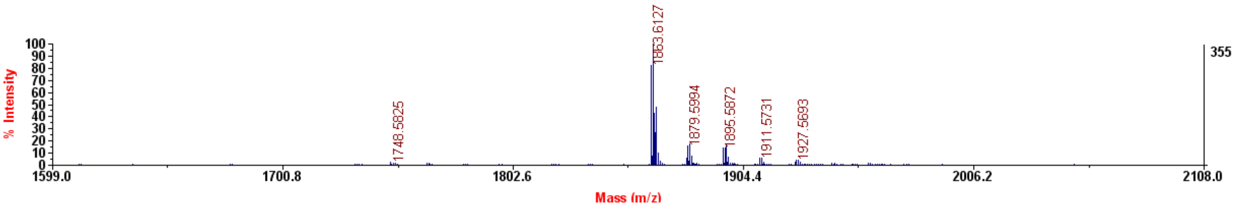

Supplementary Figure 102. MS spectra of plates 40 and 41. The data of D4, C5, and D11 in plate 40 and H4, D6, and G10 in plate 41 are shown.

plate 41/line A/column 11

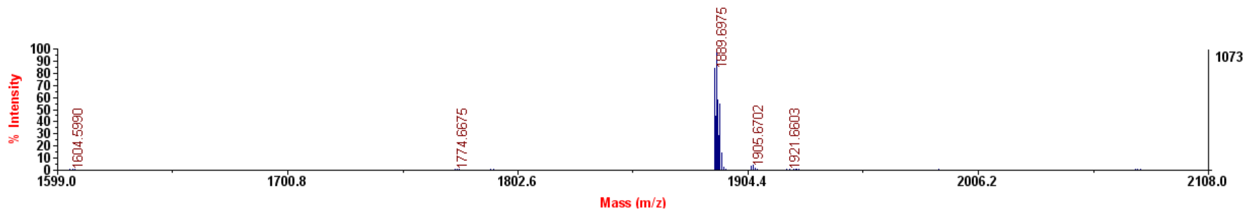

plate 43/line E/column 1

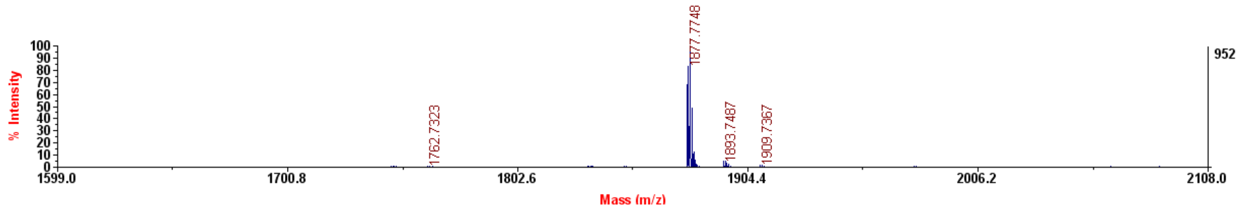

plate 43/line E/column 6

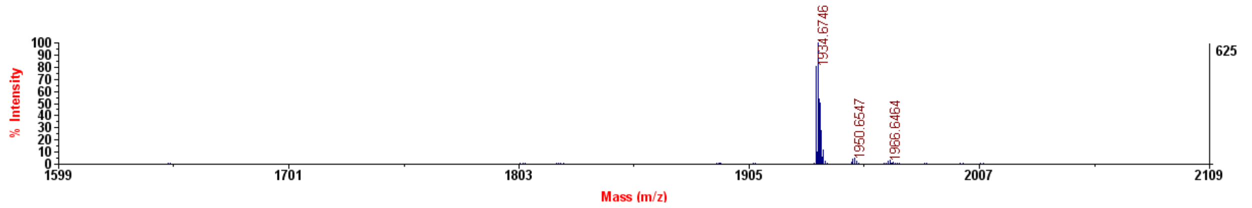

plate 43/line B/column 7

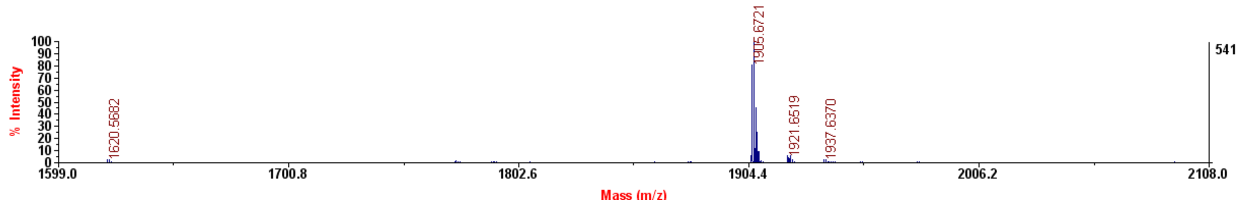

plate 43/line D/column 7

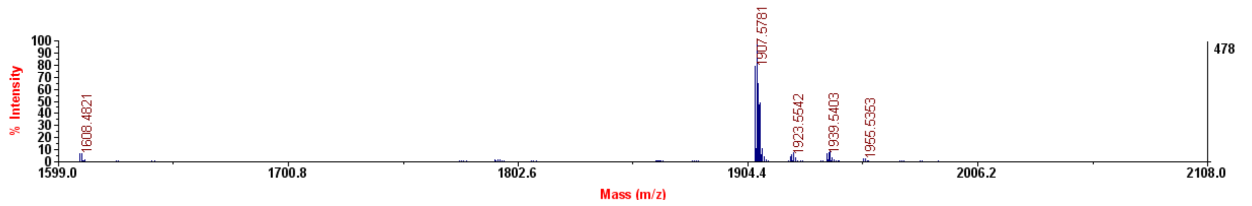

plate 43/line F/column 7

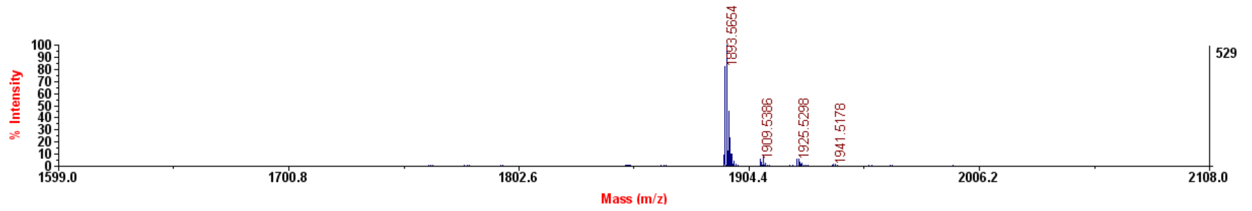

Supplementary Figure 103. MS spectra of plates 41 and 43. The data of A11 in plate 41 and E1, E6, B7, D7, and F7 in plate 43 are shown.

plate 43/line B/column 8

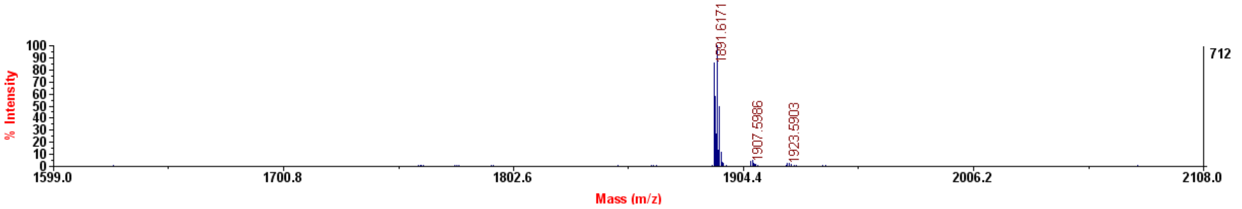

plate 43/line H/column 9

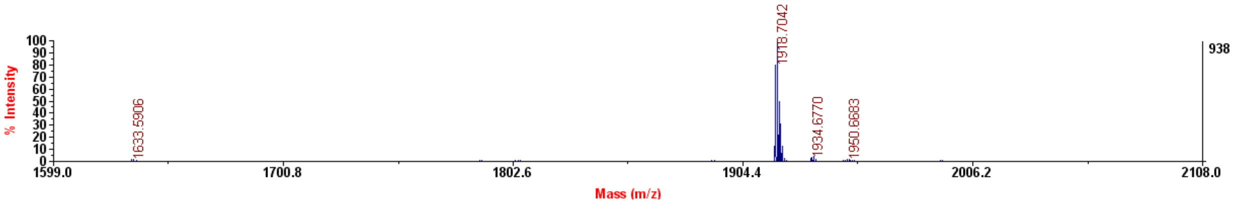

plate 44/line D/column 2

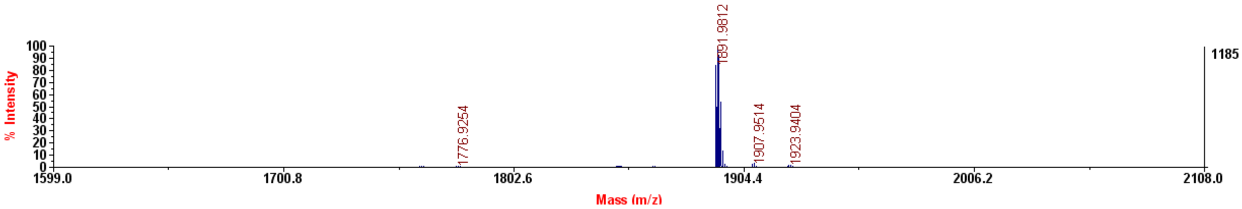

plate 44/line G/column 4

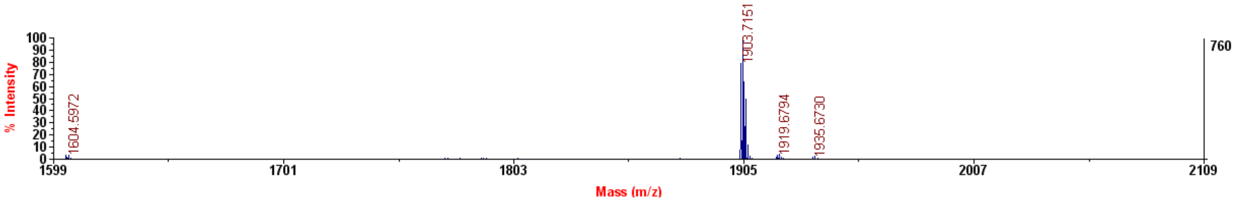

plate 44/line F/column 5

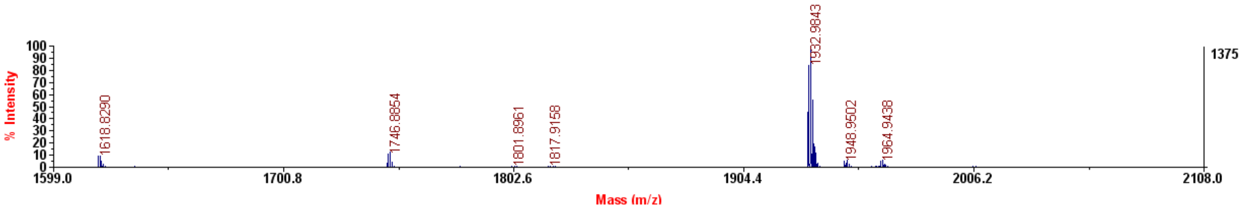

plate 44/line B/column 6

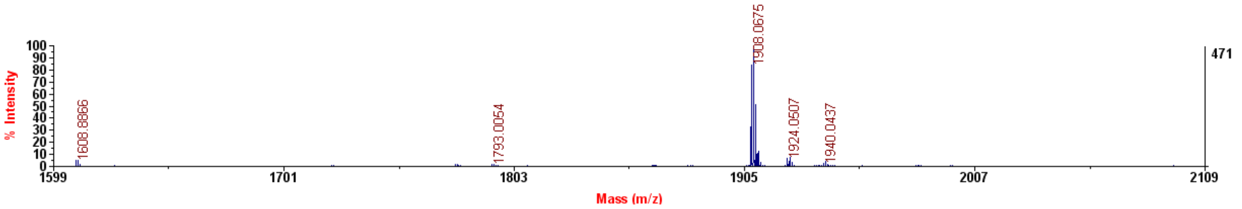

Supplementary Figure 104. MS spectra of plates 43 and 44. The data of B8 and H9 in plate 43 and D2, G4, F5, and B6 in plate 44 are shown.

plate 44/line E/column 6

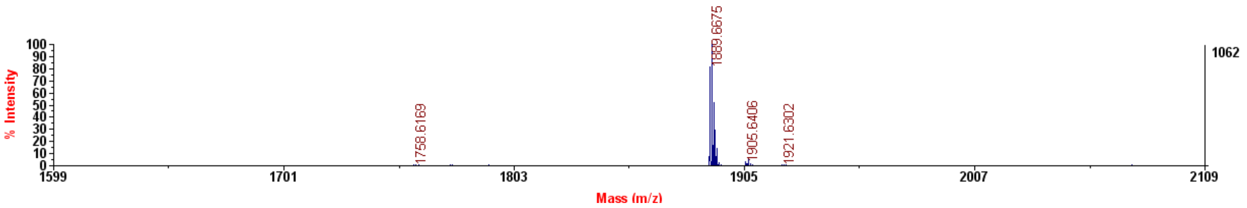

plate 44/line B/column 7

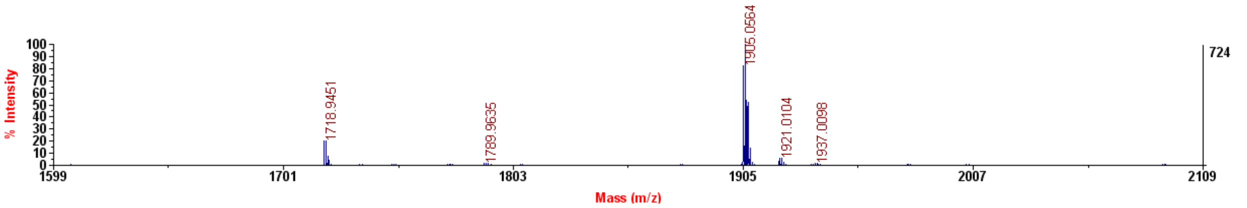

plate 44/line E/column 9

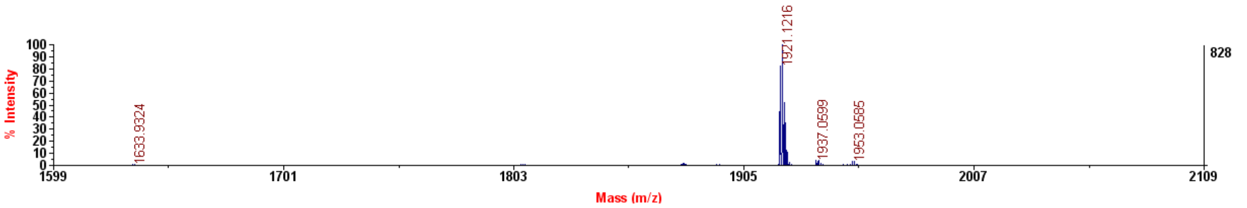

plate 44/line G/column 11

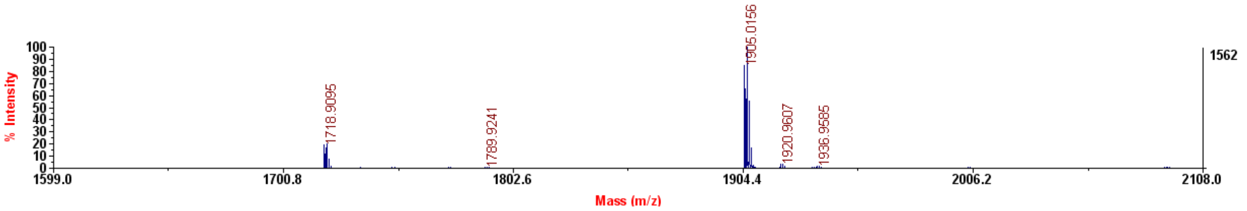

plate 45/line G/column 1

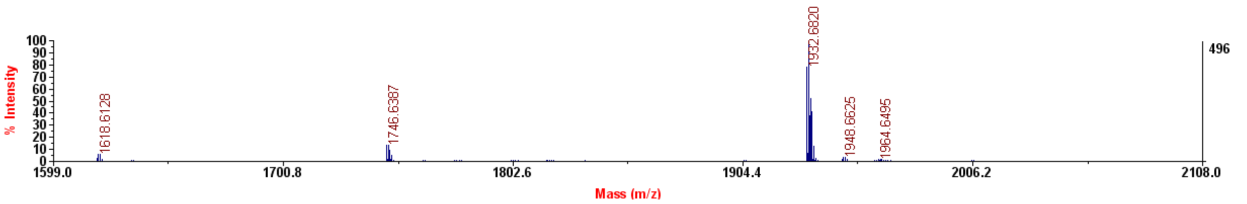

plate 45/line A/column 4

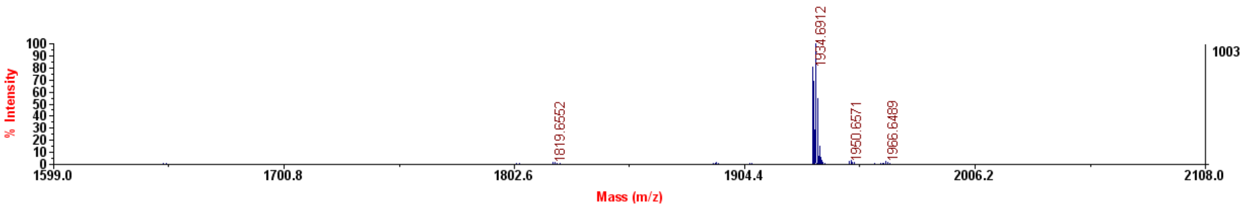

Supplementary Figure 105. MS spectra of plates 44 and 45. The data of E6, B7, E9, and G11 in plate 44 and G1 and A4 in plate 45 are shown.

plate 45/line C/column 4

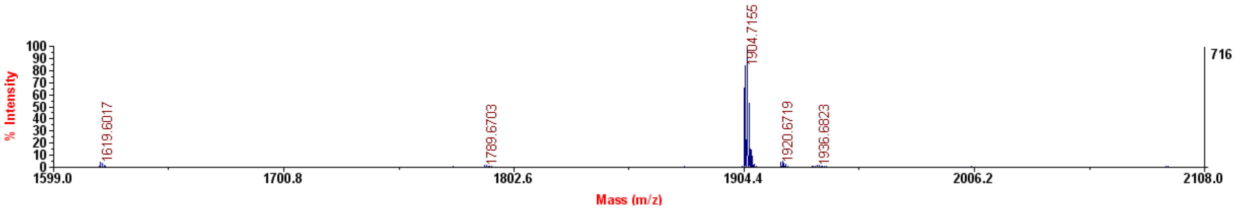

plate 45/line D/column 7

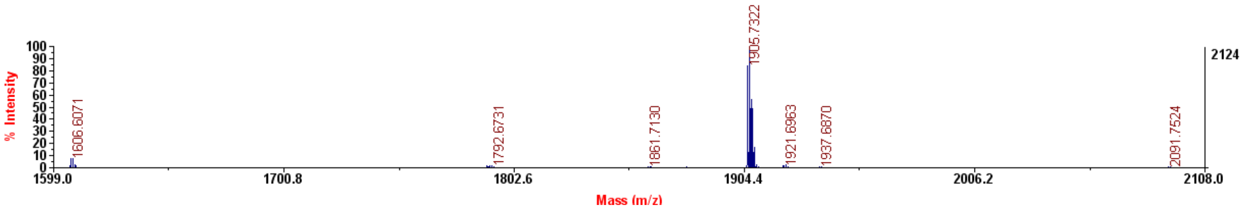

plate 46/line G/column 5

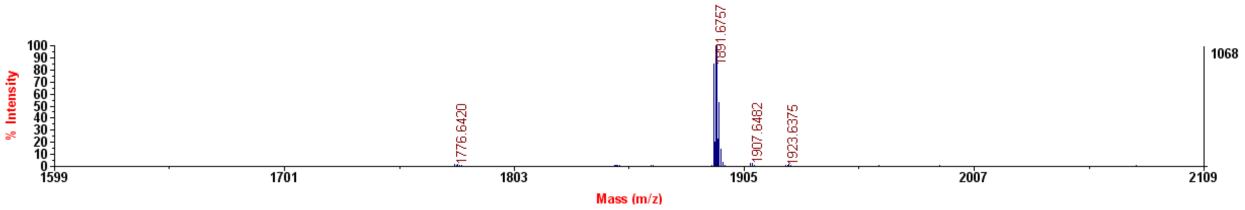

plate 46/line A/column 8

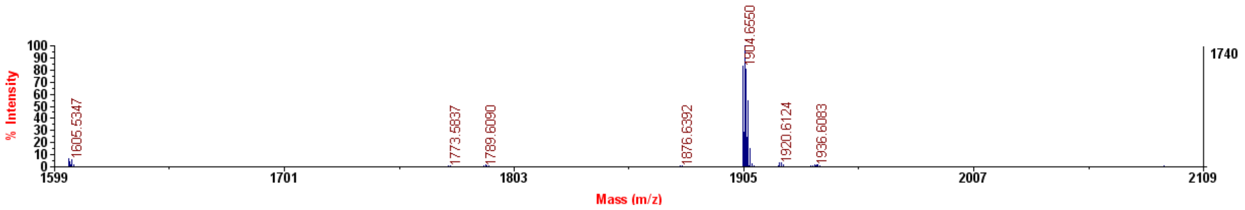

plate 47/line F/column 6

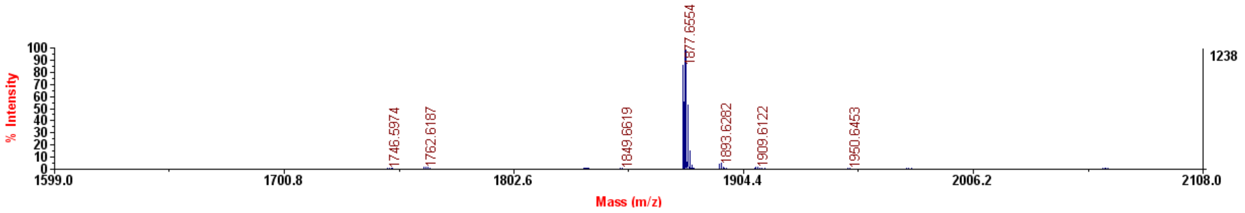

plate 47/line H/column 6

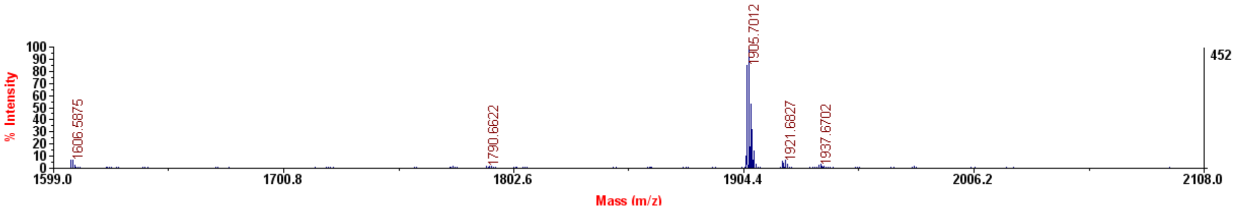

Supplementary Figure 106. MS spectra of plates 45, 46, and 47. The data of C4 and D7 in plate 45, G5 and A8 in plate 46, and F6 and H6 in plate 47 are shown.

plate 47/line G/column 8

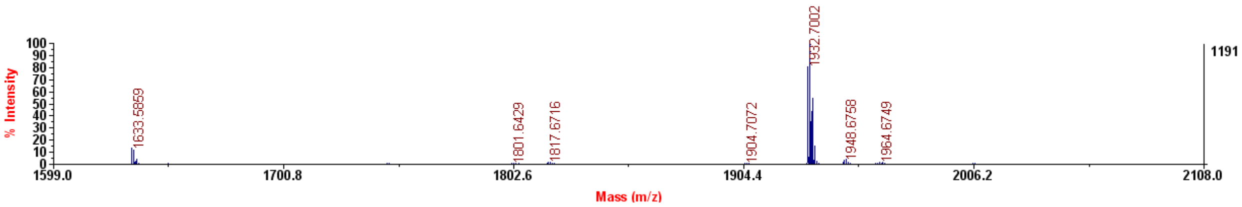

plate 48/line B/column 3

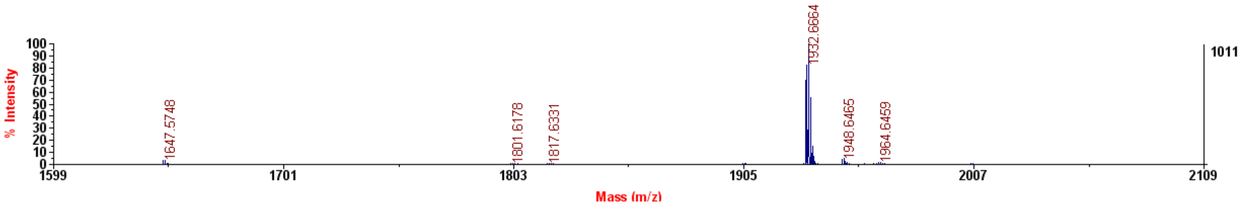

plate 48/line C/column 3

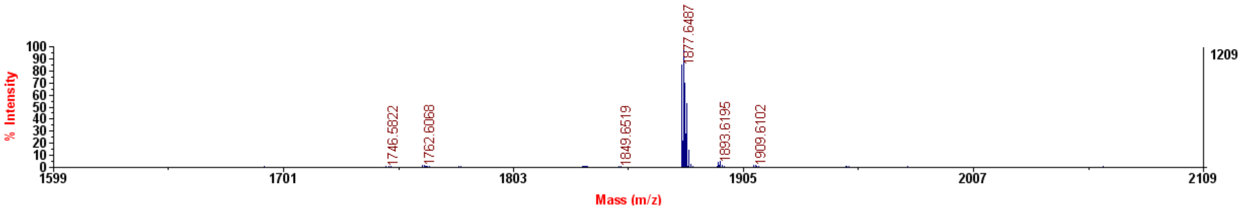

plate 48/line C/column 4

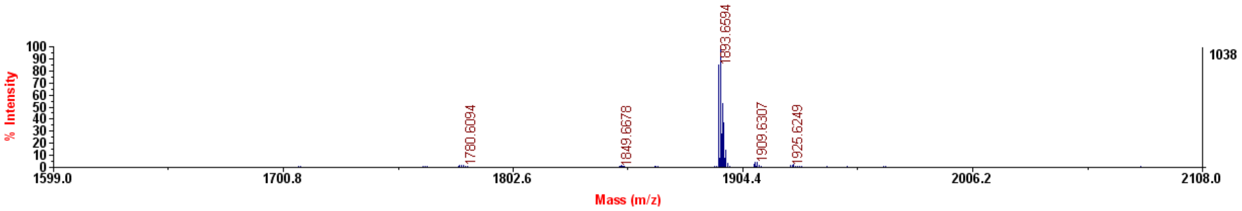

plate 48/line C/column 5

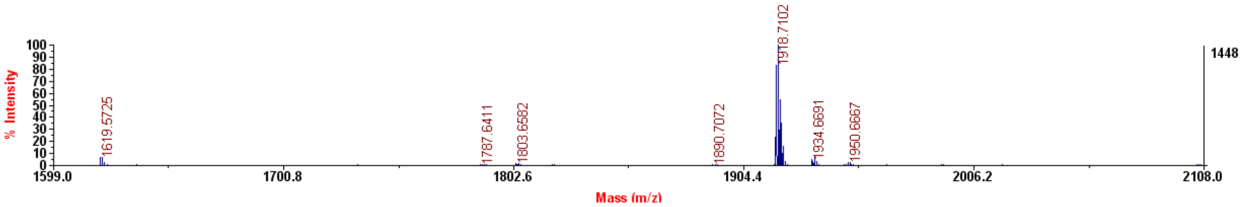

plate 48/line H/column 5

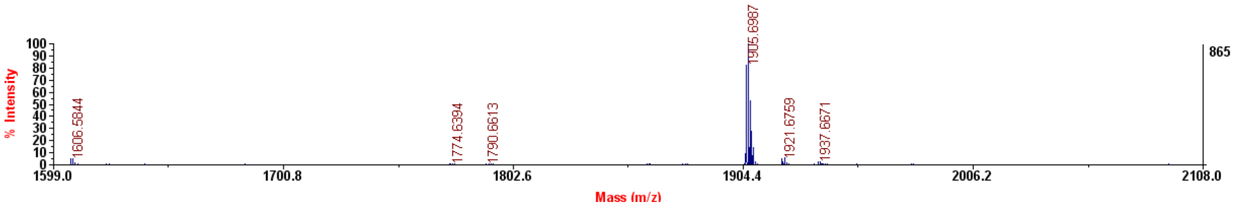

Supplementary Figure 107. MS spectra of plates 47 and 48. The data of G8 in plate 47 and B3, C3, C4, C5, and H5 in plate 48 are shown.

plate 48/line A/column 6

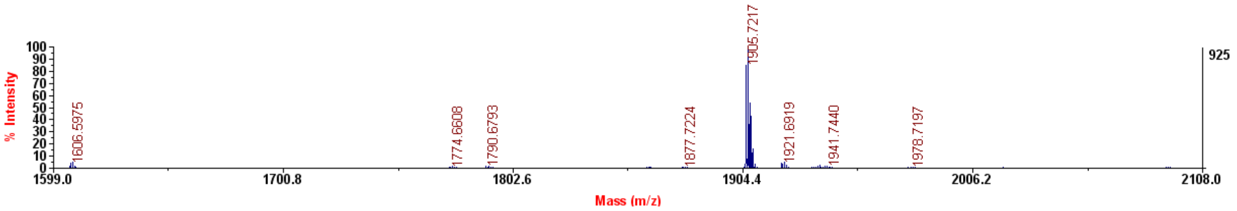

plate 48/line G/column 7

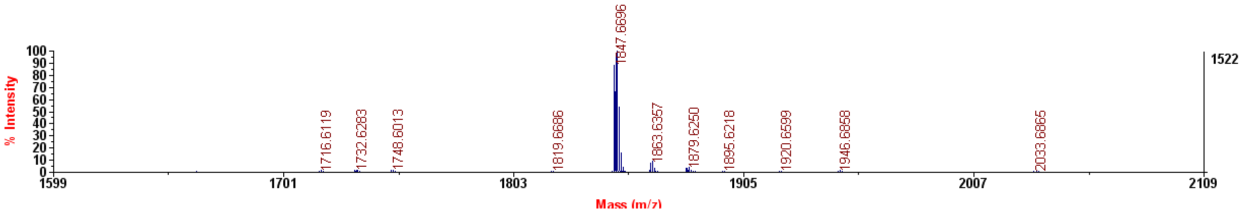

plate 48/line H/column 8

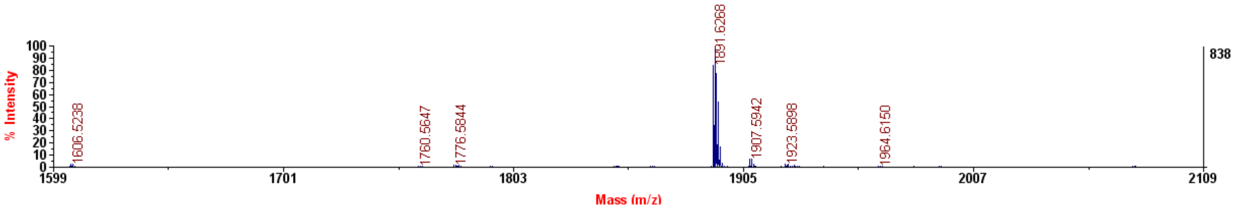

plate 48/line D/column 9

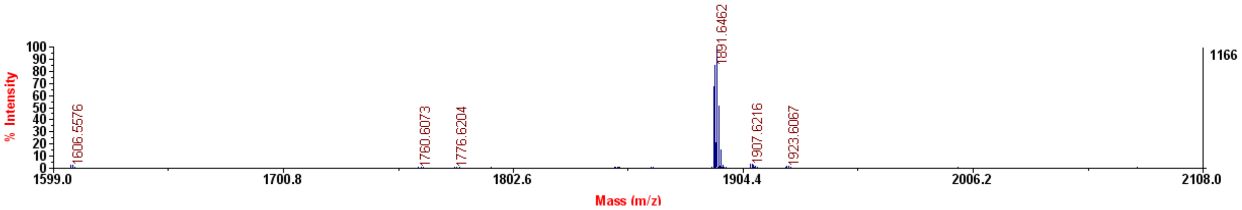

plate 49/line B/column 1

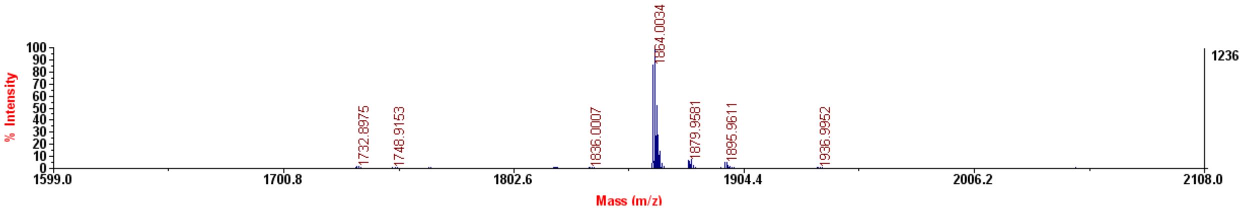

plate 49/line G/column 3

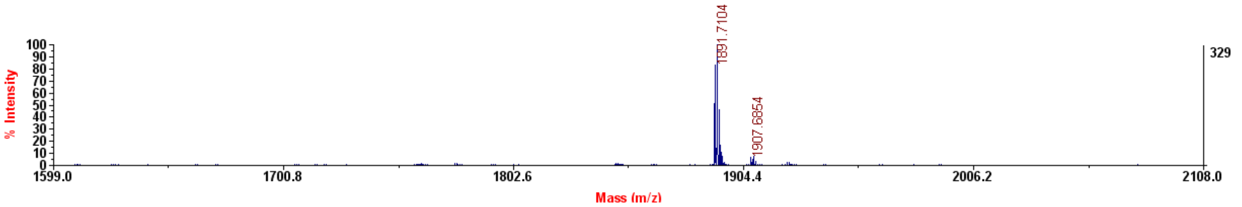

Supplementary Figure 108. MS spectra of plates 48 and 49. The data of A6, G7, H8, and D9 in plate 48 and B1 and G3 in plate 49 are shown.

plate 49/line A/column 6

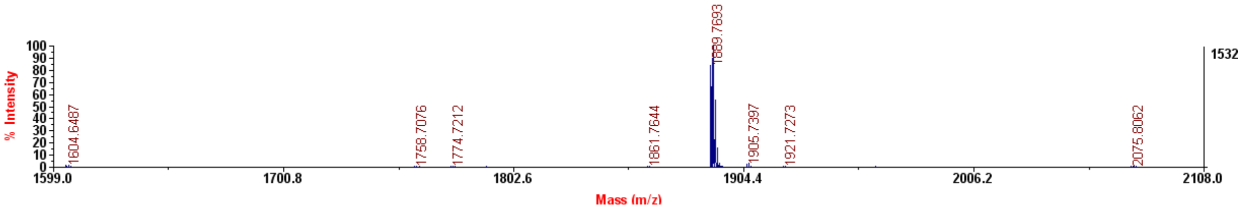

plate 49/line A/column 8

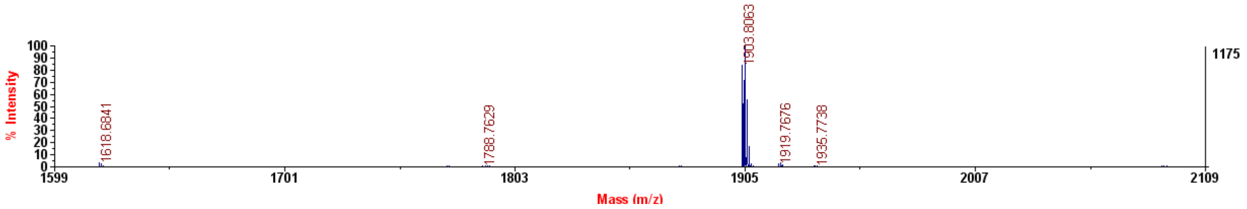

plate 49/line B/column 9

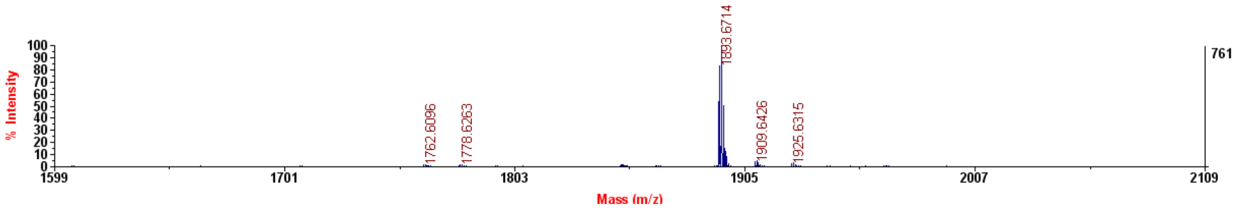

plate 49/line H/column 9

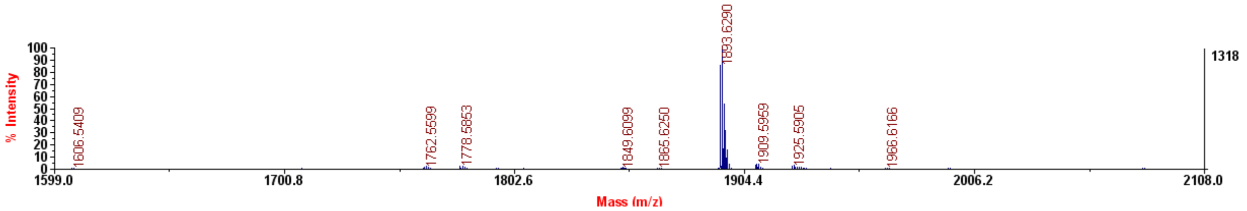

plate 49/line A/column 10

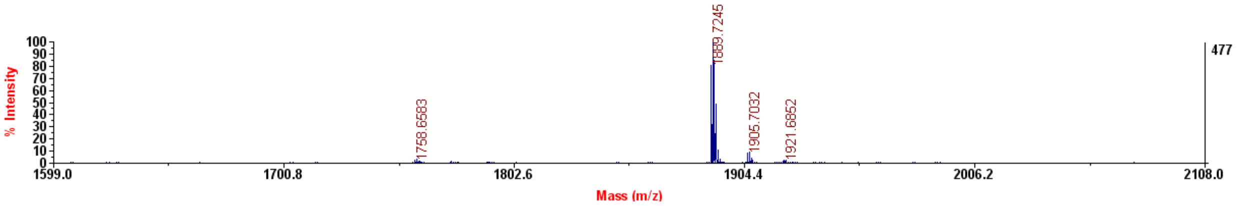

plate 50/line E/column 2

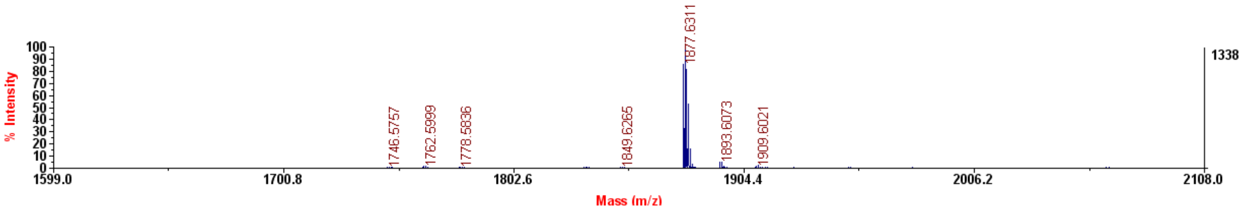

Supplementary Figure 109. MS spectra of plates 49 and 50. The data of A6, A8, B9, H9, and A10 in plate 49 and E2 in plate 50 are shown.

plate 50/line C/column 7

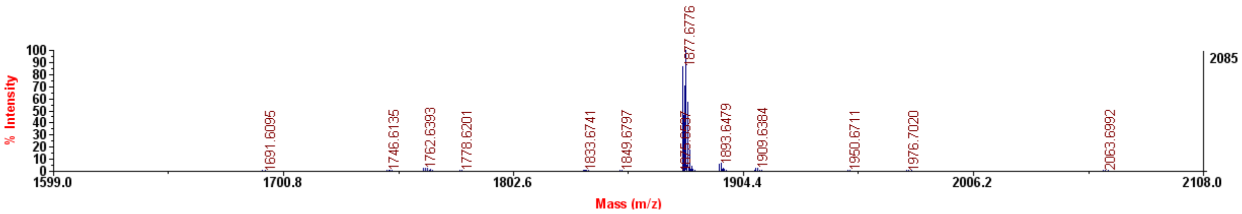

plate 50/line E/column 10

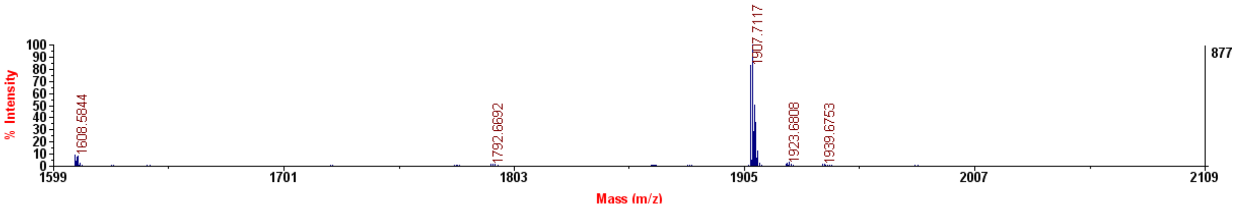

plate 50/line C/column 11

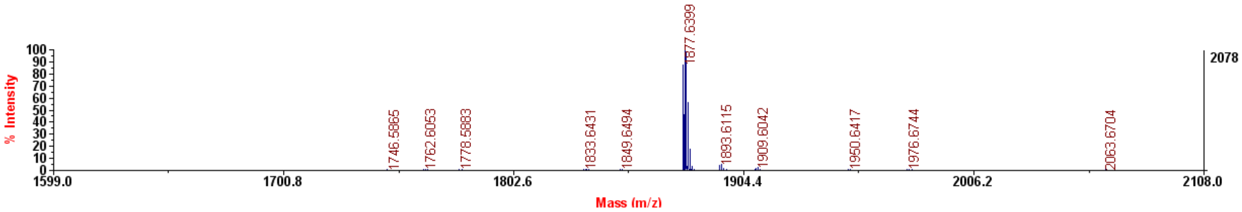

plate 51/line H/column 1

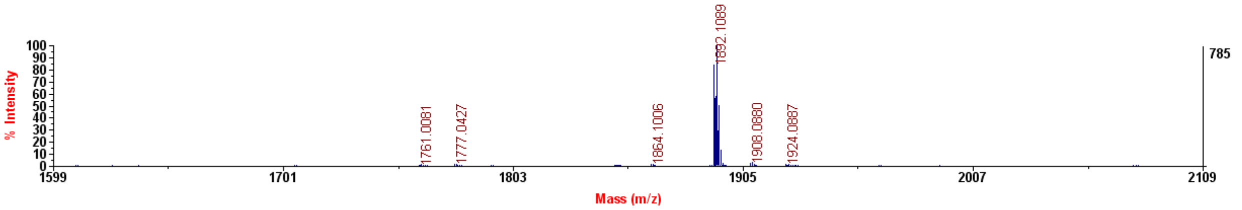

plate 51/line D/column 3

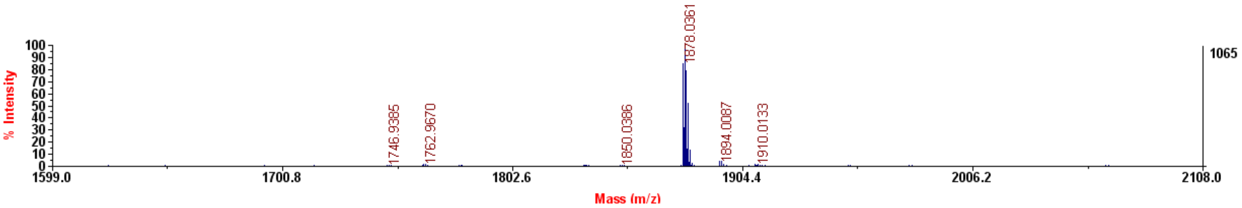

plate 51/line E/column 6

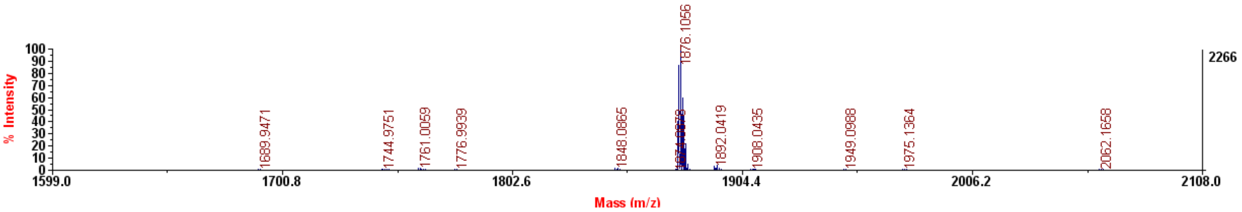

Supplementary Figure 110. MS spectra of plates 50 and 51. The data of C7, E10, and C11 in plate 50 and H1, D3, and E6 in plate 51 are shown.

plate 51/line E/column 8

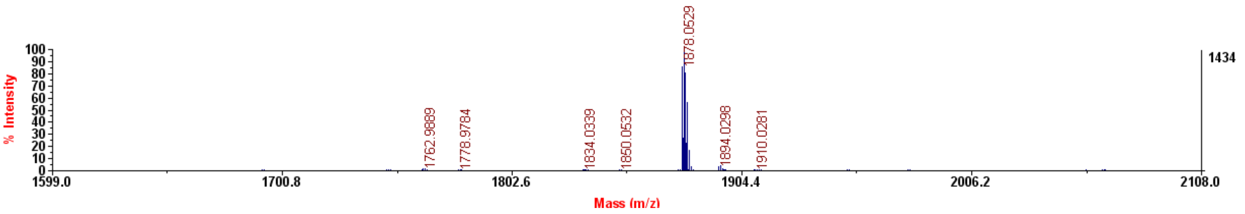

plate 51/line G/column 8

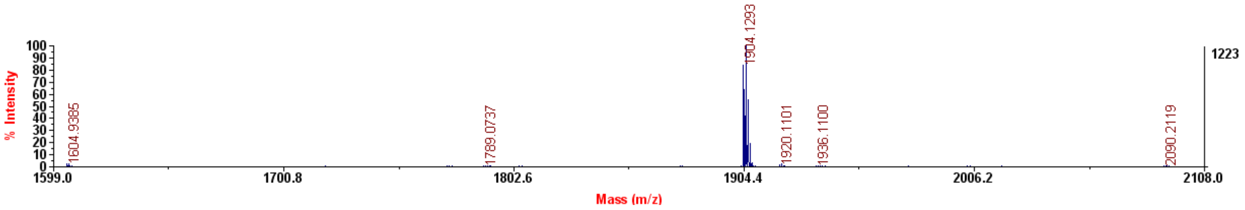

plate 52/line D/column 2

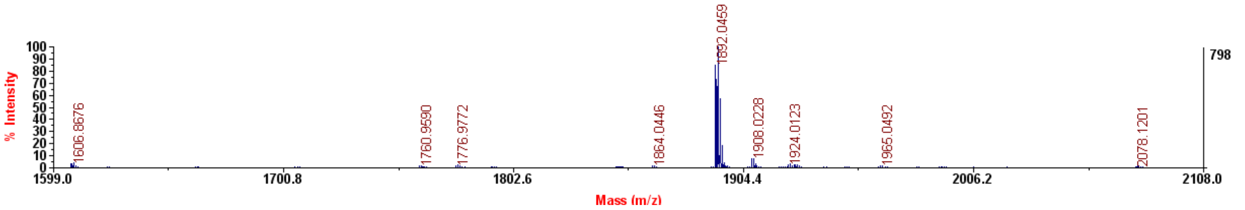

plate 52/line A/column 4

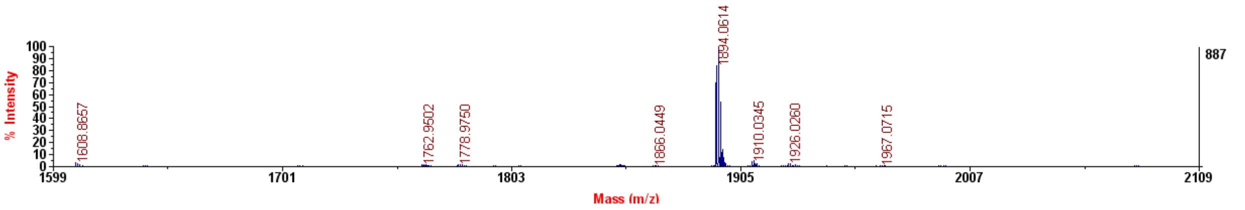

plate 52/line C/column 11

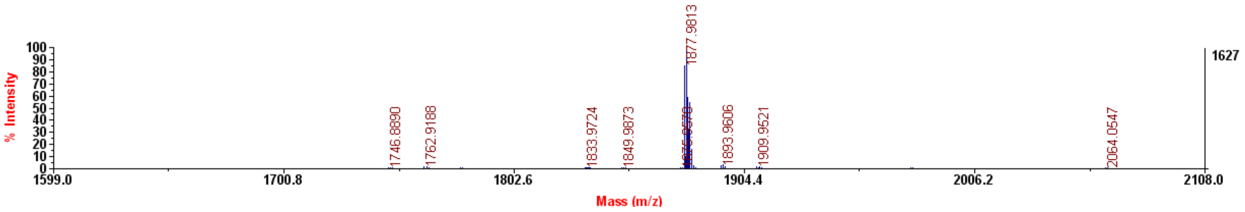

plate 52/line E/column 11

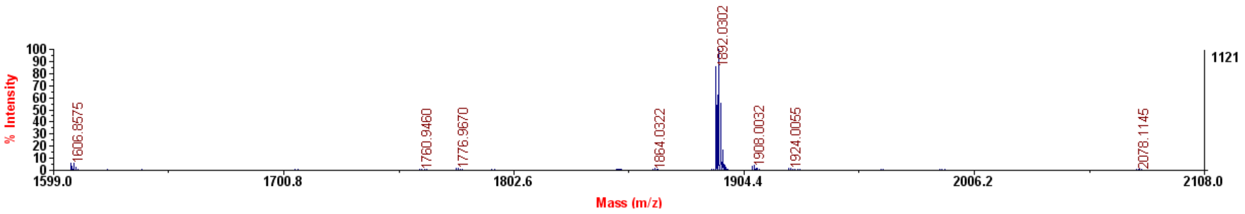

Supplementary Figure 111. MS spectra of plates 51 and 52. The data of E8 and G8 in plate 51 and D2, A4, C11, and E11 in plate 52 are shown.

plate 53/line D/column 6

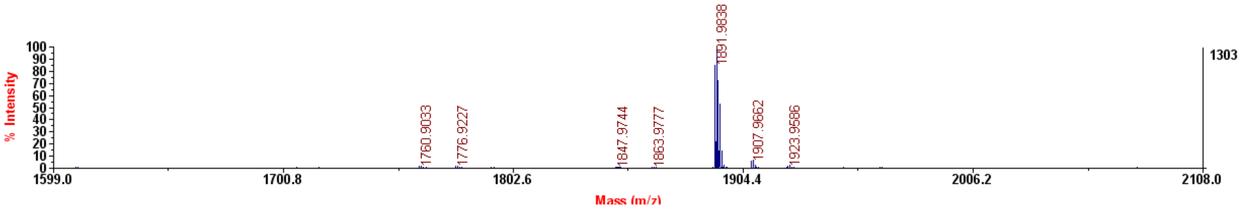

plate 53/line G/column 10

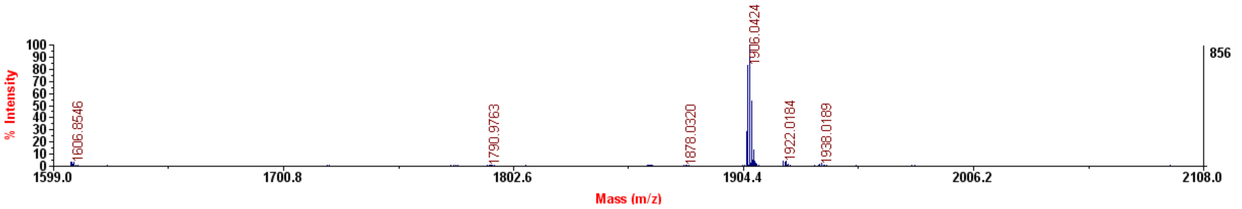

plate 53/line B/column 11

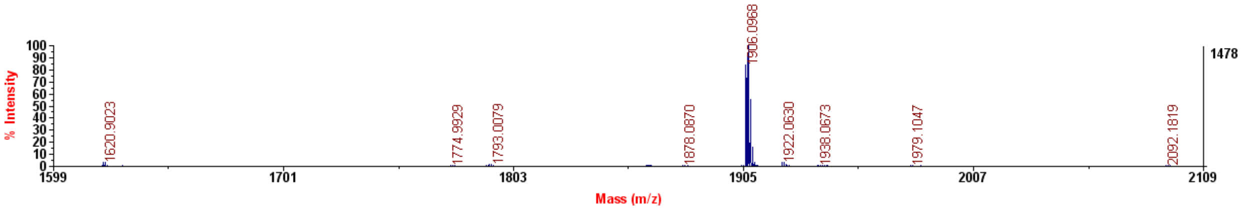

plate 54/line F/column 2

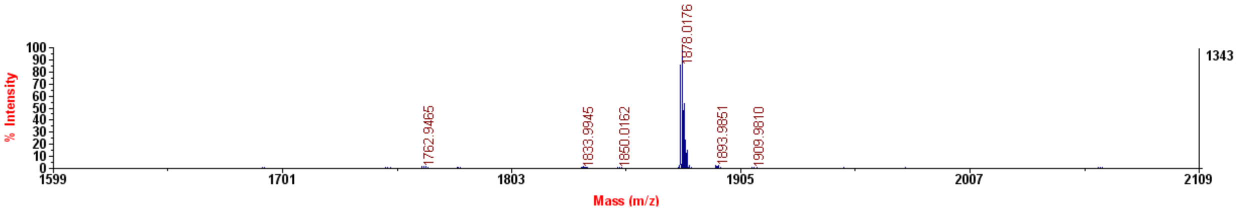

plate 54/line B/column 11

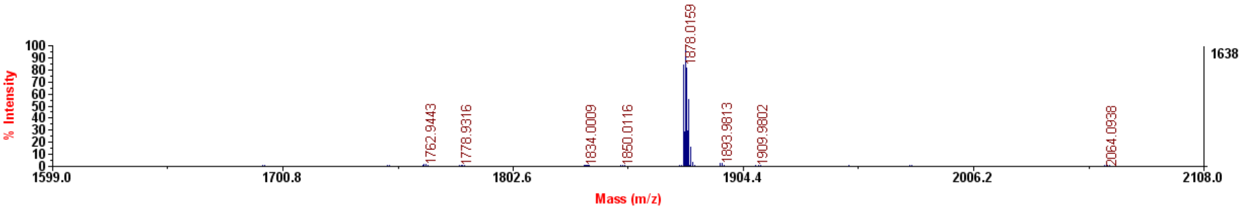

plate 54/line H/column 11

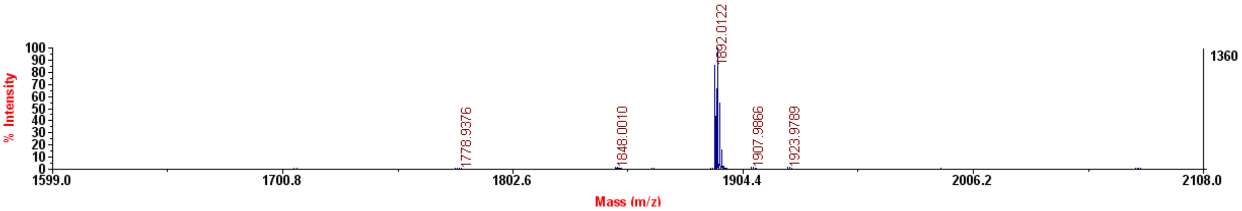

Supplementary Figure 112. MS spectra of plates 53 and 54. The data of D6, G10, and B11 in plate 53 and F2, B11, and H11 in plate 54 are shown.

plate 55/line H/column 3

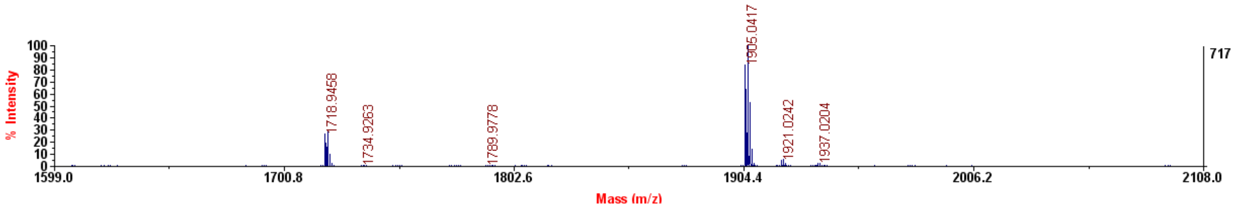

plate 55/line F/column 6

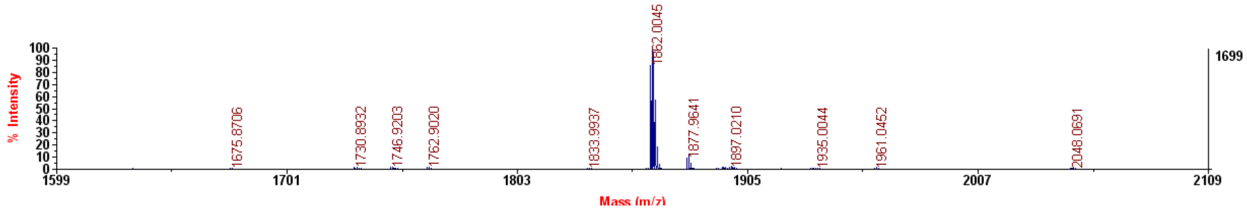

plate 55/line D/column 11

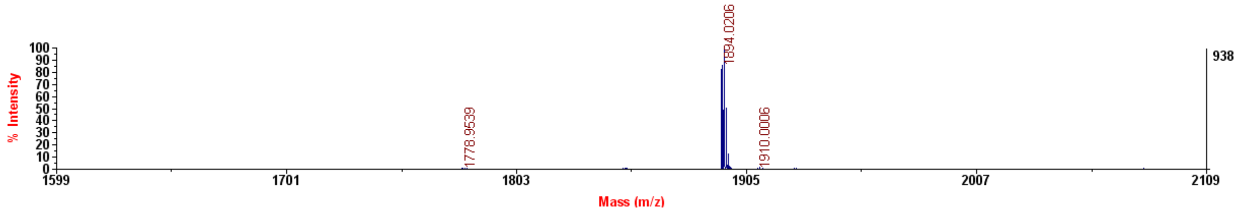

plate 56/line D/column 2

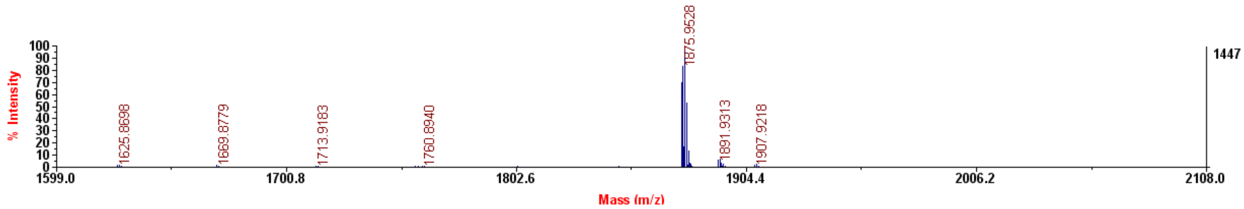

plate 56/line G/column 3

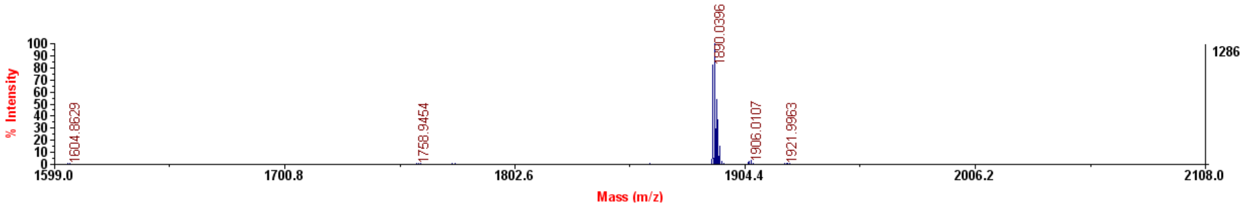

plate 56/line B/column 4

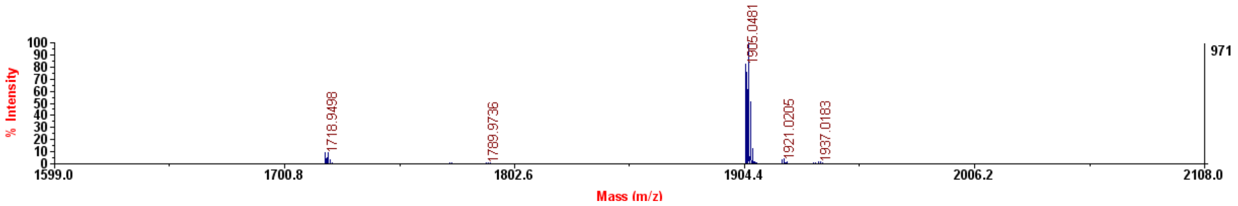

Supplementary Figure 113. MS spectra of plates 55 and 56. The data of H3, F6, and D11 in plate 55 and D2, G3, and B4 in plate 56 are shown.

plate 57/line F/column 6

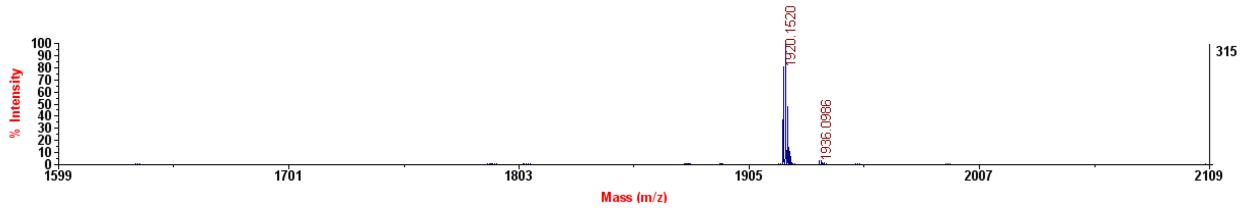

plate 58/line H/column 1

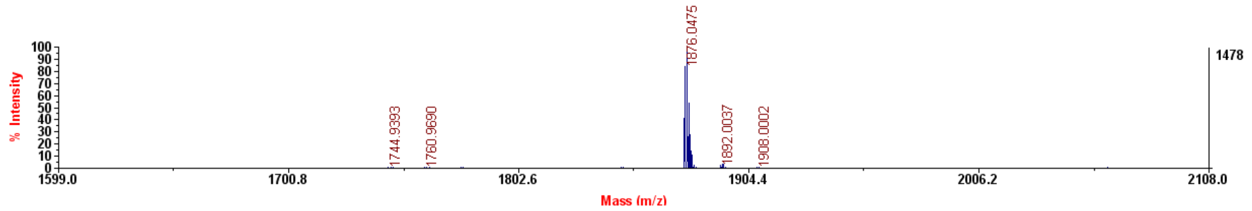

plate 58/line B/column 2

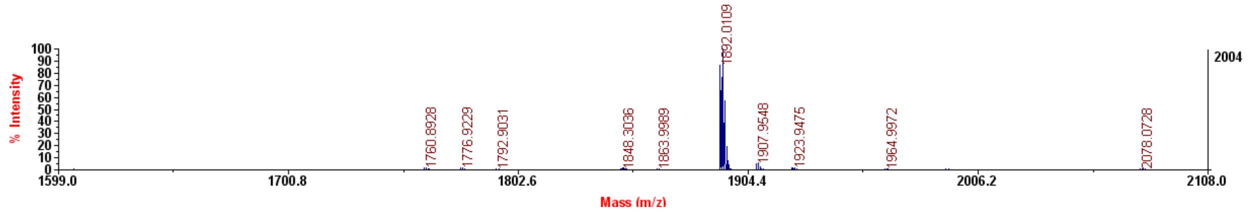

plate 58/line C/column 6

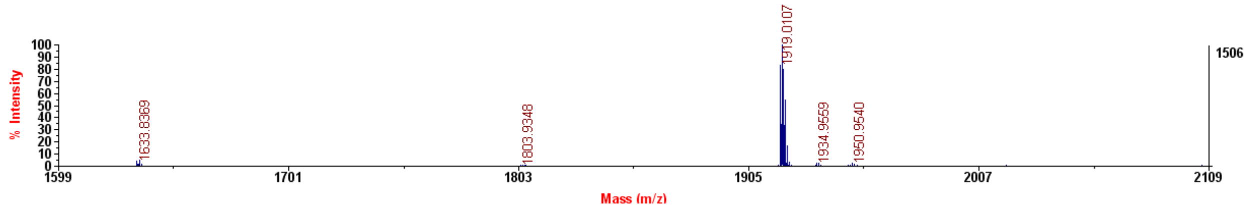

plate 59/line D/column 4

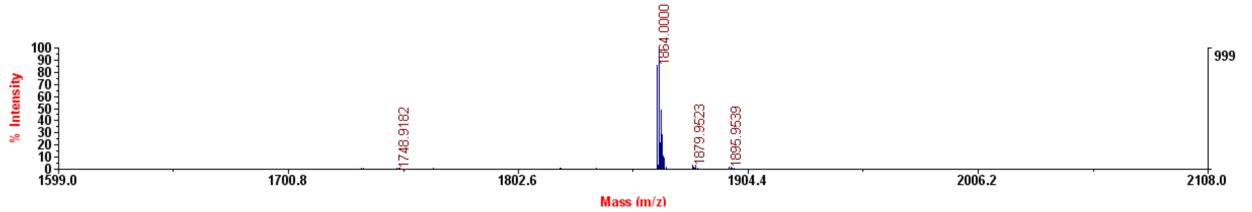

plate 59/line G/column 4

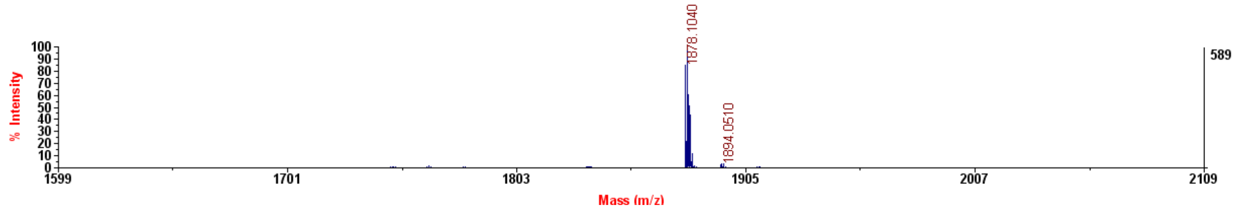

Supplementary Figure 114. MS spectra of plates 57, 58, and 59. The data of F6 in plate 57, H1, B2, and C6 in plate 58, and D4 and G4 in plate 59 are shown.

plate 59/line D/column 5

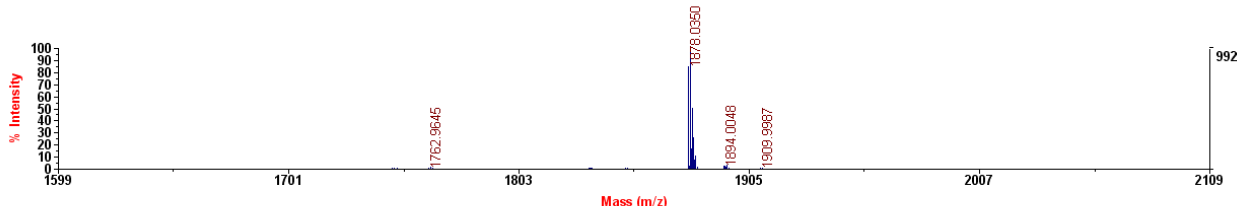

plate 59/line B/column 6

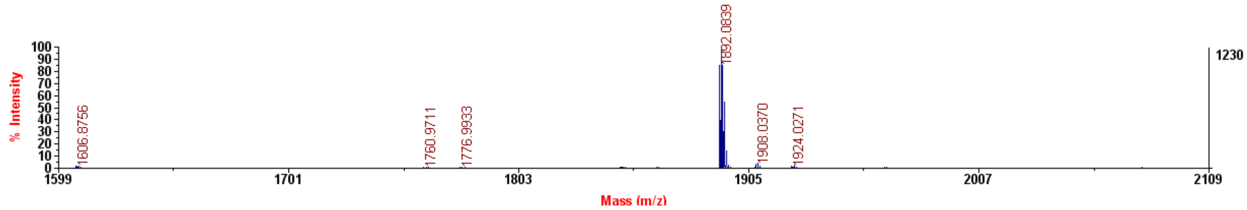

plate 59/line A/column 7

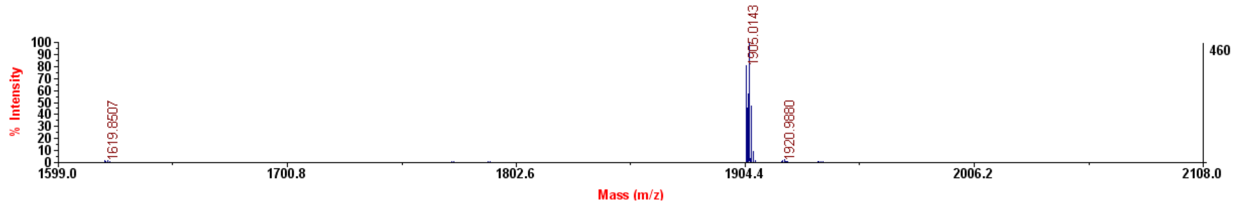

plate 59/line E/column 9

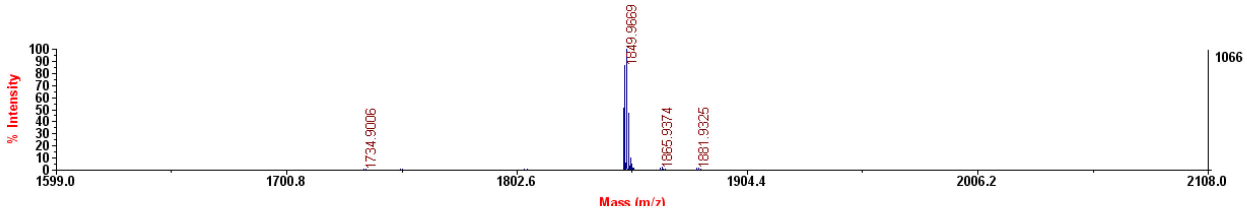

plate 59/line C/column 10

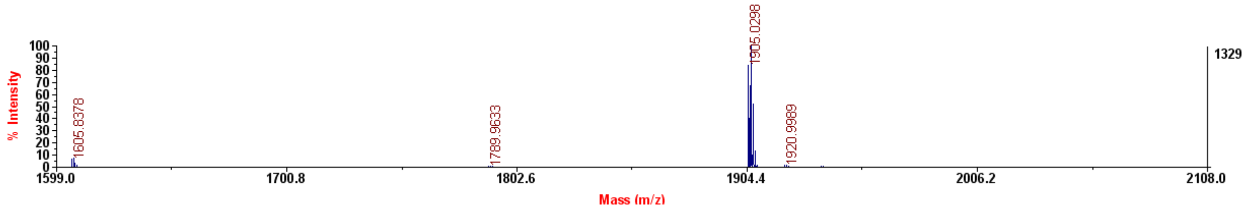

plate 59/line G/column 10

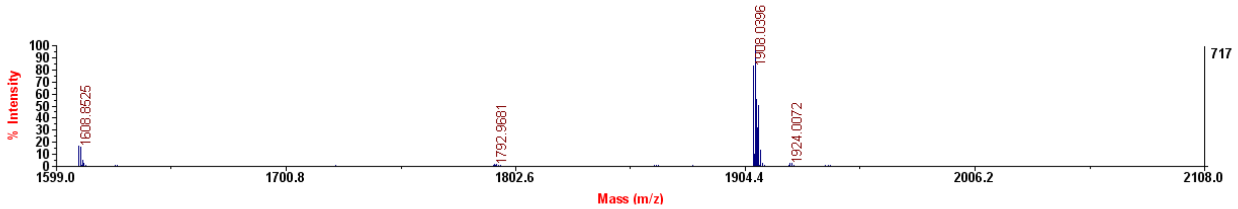

Supplementary Figure 115. MS spectra of plate 59. The data of D5, B6, A7, E9, C10, and G10 in plate 59 are shown.

plate 60/line A/column 3

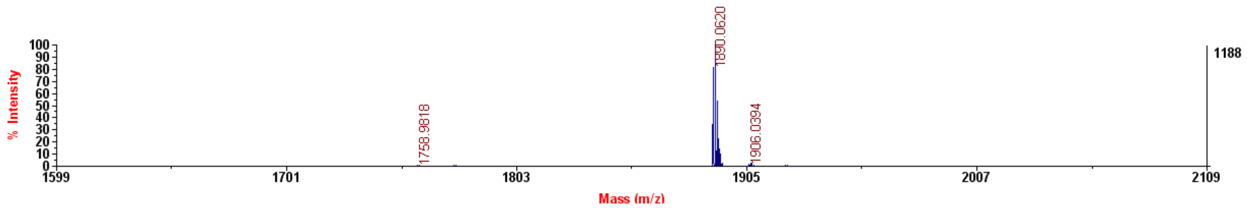

plate 60/line G/column 4

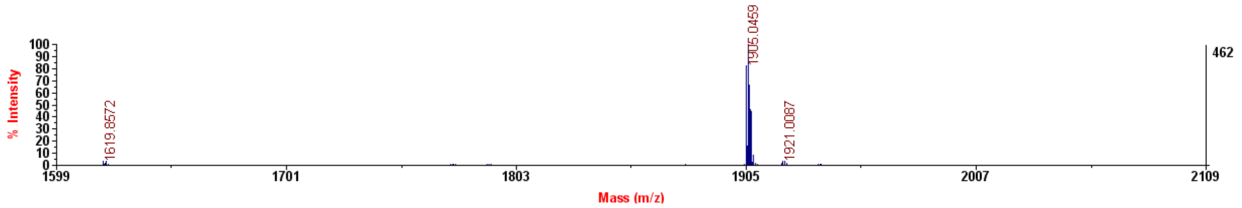

plate 60/line D/column 8

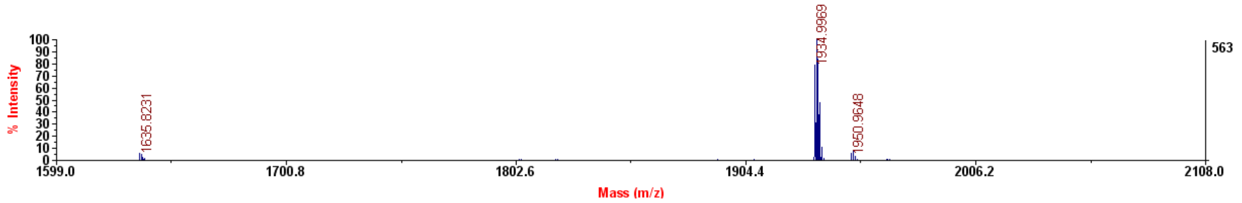

plate 60/line F/column 10

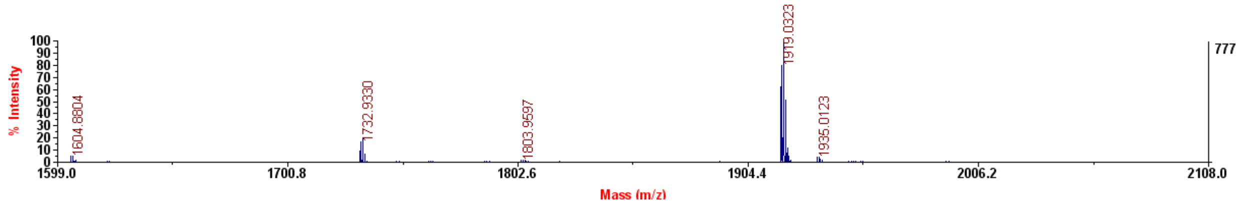

plate 60/line G/column 11

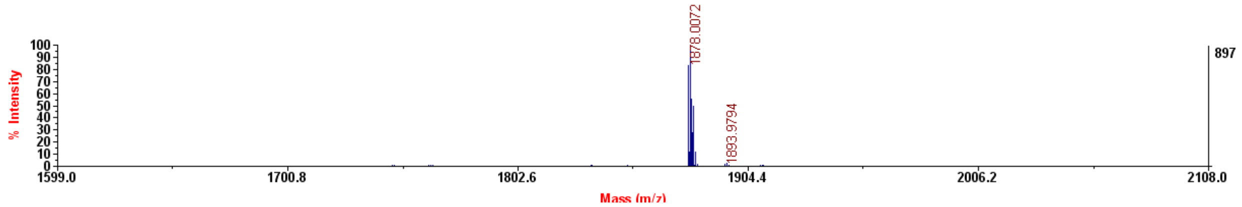

plate 61/line A/column 3

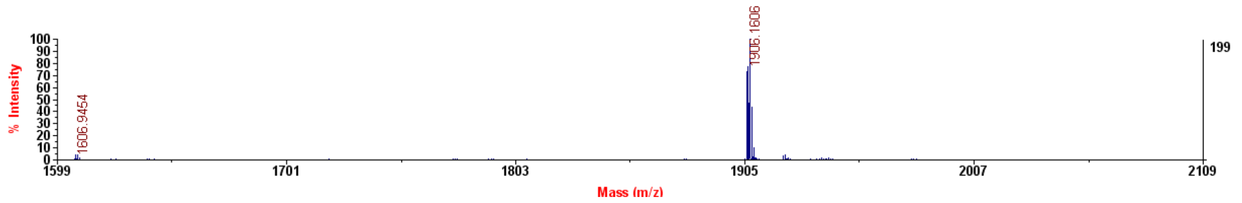

Supplementary Figure 116. MS spectra of plates 60 and 61. The data of A3, G4, D8, F10, and G11 in plate 60 and A3 in plate 61 are shown.

plate 61/line G/column 4

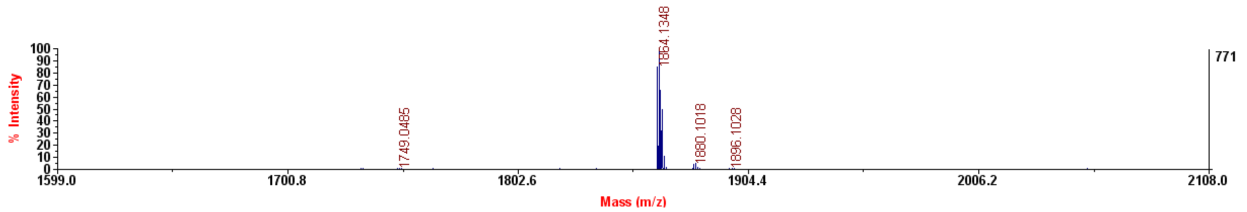

plate 61/line B/column 8

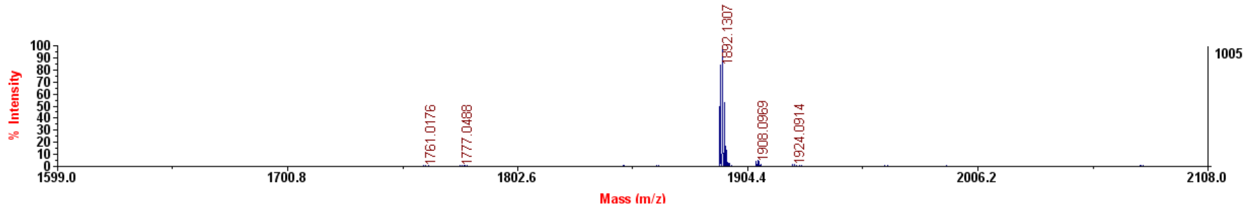

plate 61/line D/column 9

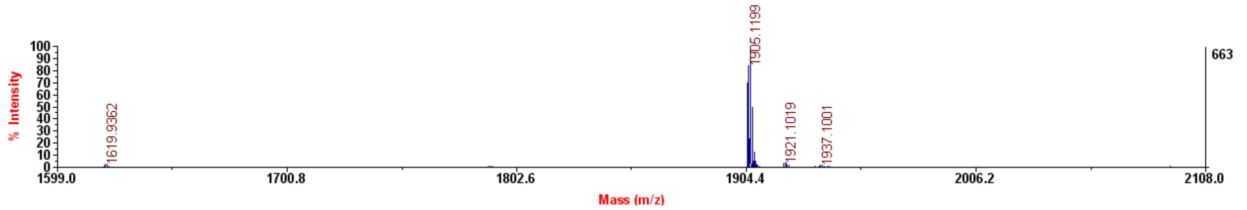

plate 61/line A/column 11

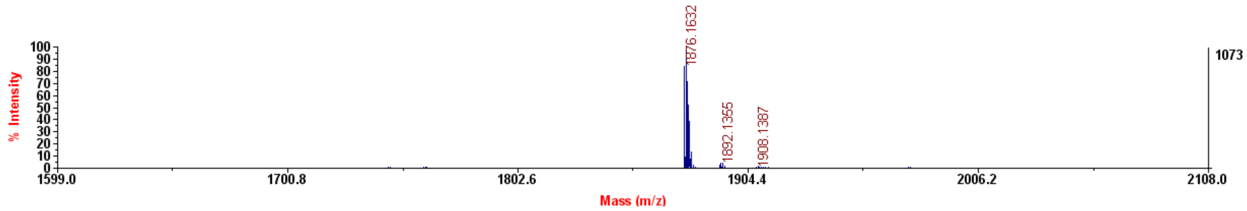

plate 62/line G/column 8

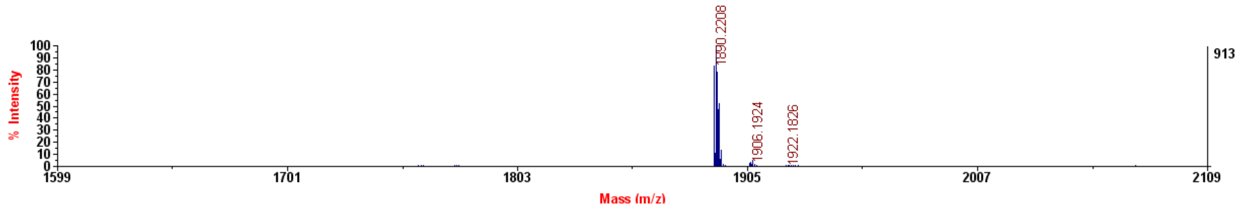

plate 62/line B/column 11

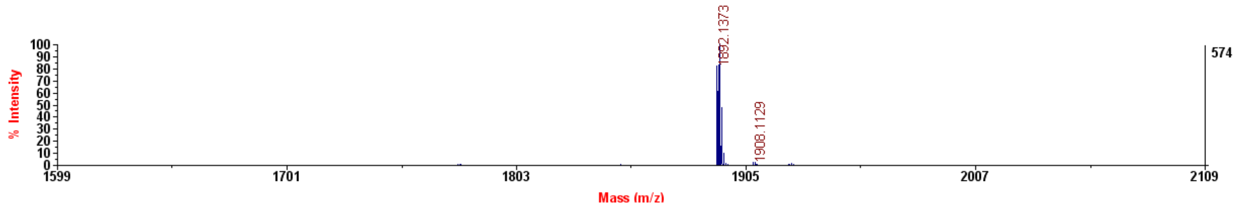

Supplementary Figure 117. MS spectra of plates 61 and 62. The data of G4, B8, D9, and A11 in plate 61 and G8 and B11 in plate 62 are shown.

plate 62/line H/column 11

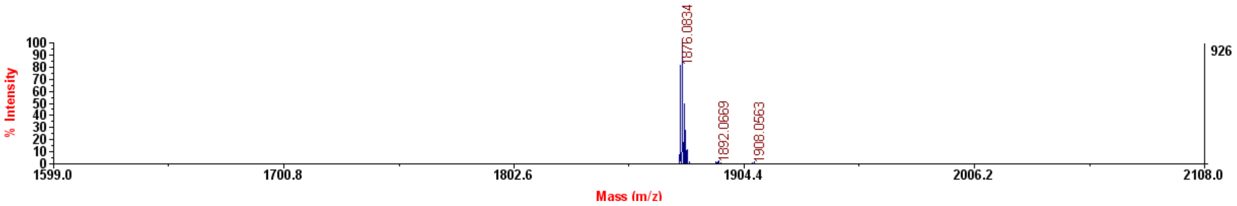

plate 63/line C/column 1

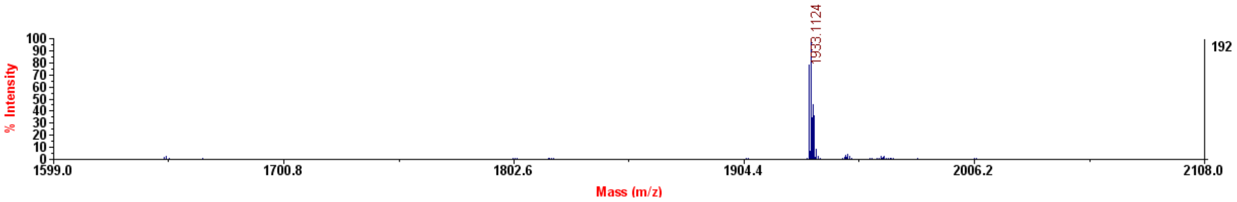

plate 63/line D/column 3

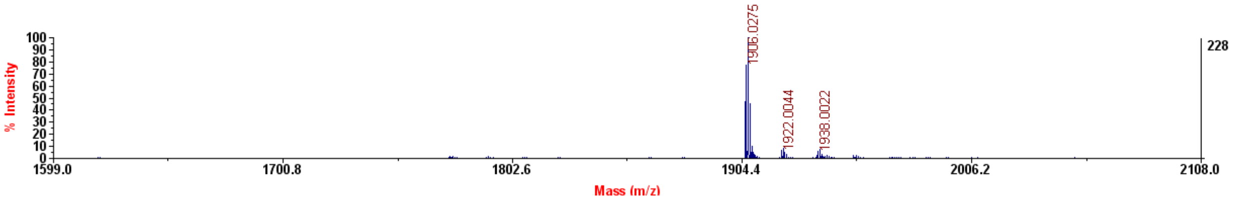

plate 63/line B/column 5

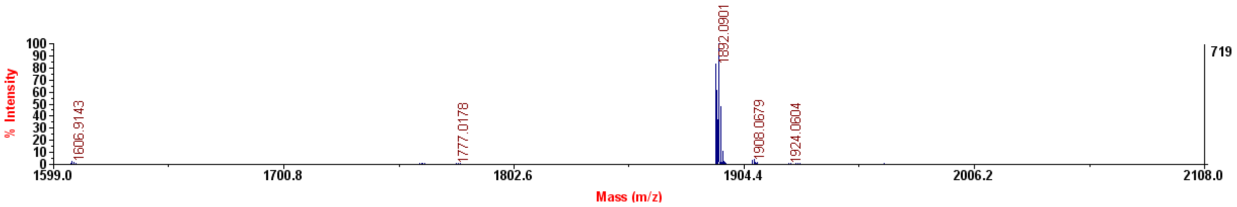

plate 63/line F/column 5

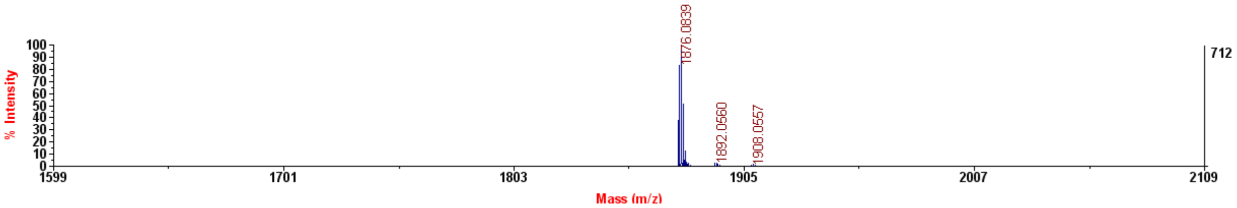

plate 63/line D/column 6

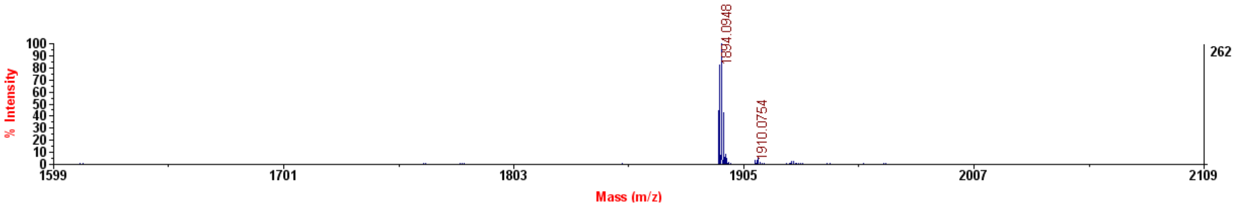

Supplementary Figure 118. MS spectra of plates 62 and 63. The data of H11 in plate 62 and C1, D3, B5, F5, and D6 in plate 63 are shown.

plate 63/line H/column 6

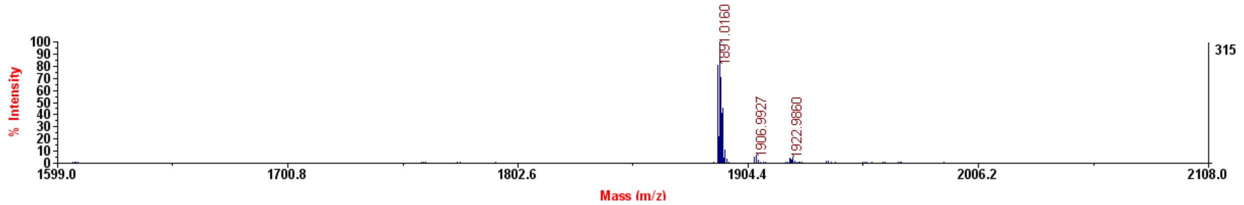

plate 63/line B/column 7

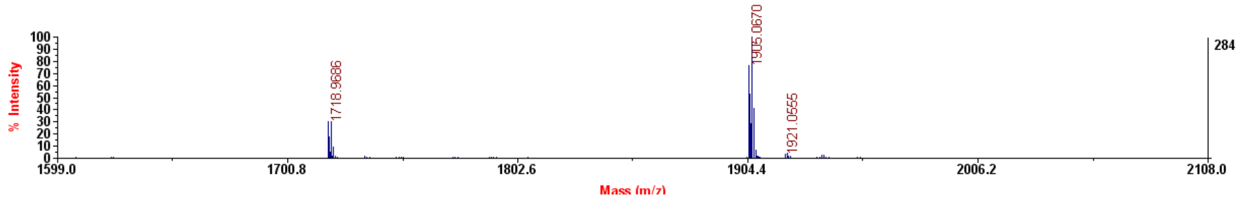

plate 63/line F/column 7

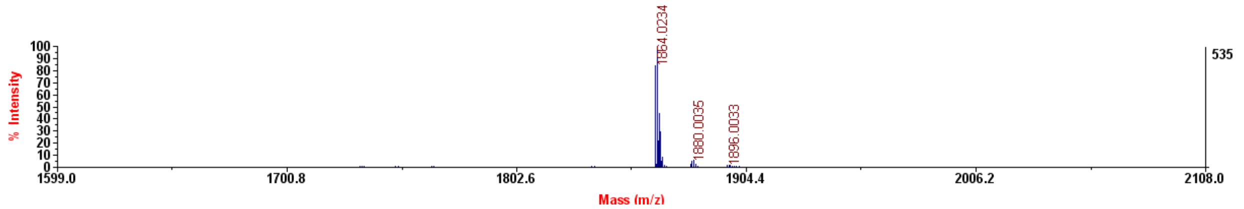

plate 63/line C/column 9

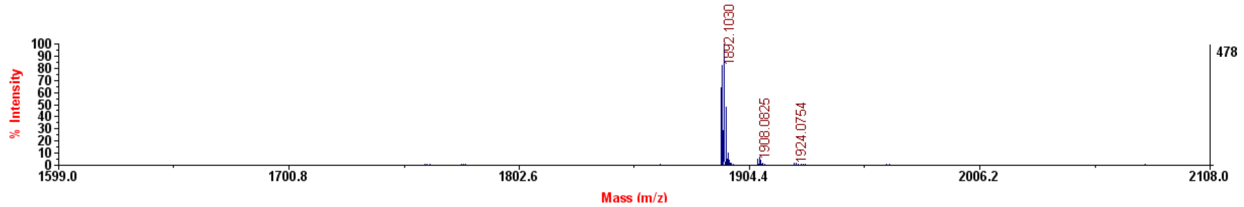

plate 63/line A/column 10

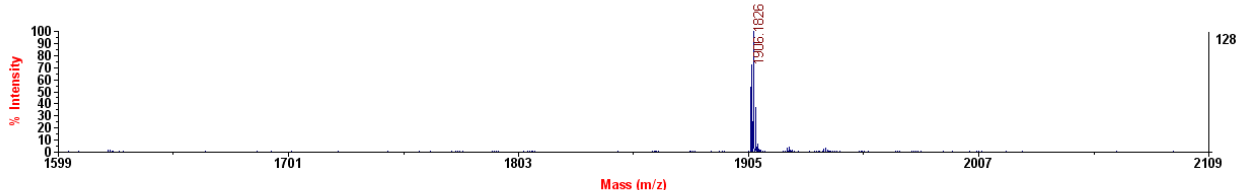

plate 63/line C/column 10

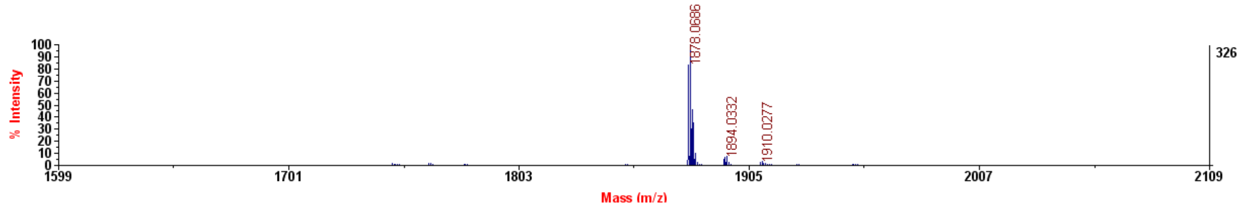

Supplementary Figure 119. MS spectra of plate 63. The data of H6, B7, F7, C9, A10, and C10 in plate 63 are shown.

plate 63/line B/column 11

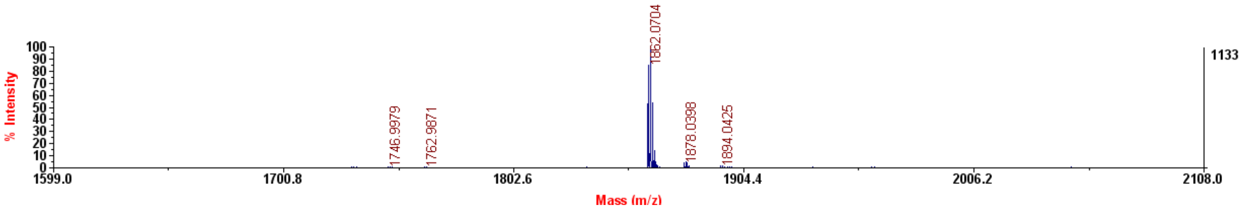

plate 63/line D/column 11

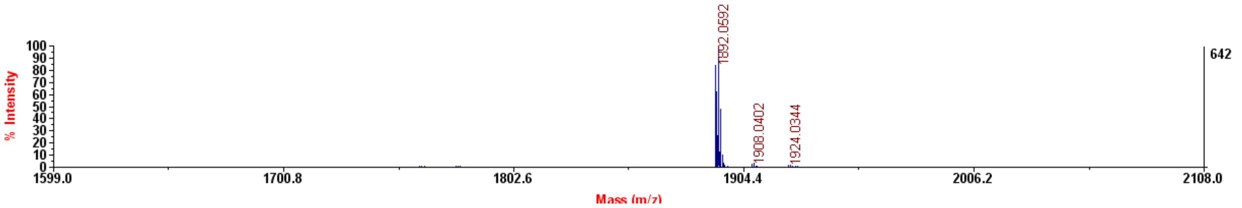

plate 64/line C/column 2

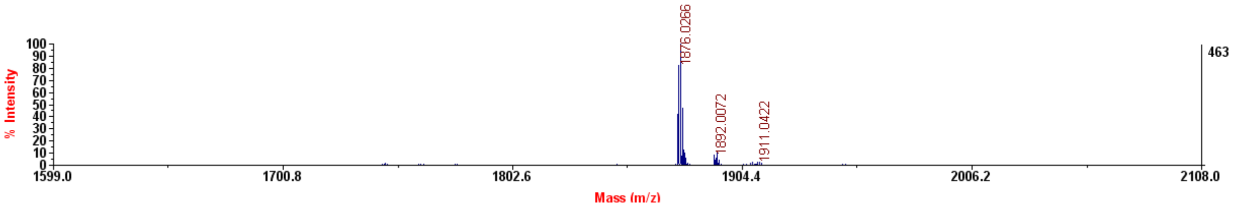

plate 64/line D/column 4

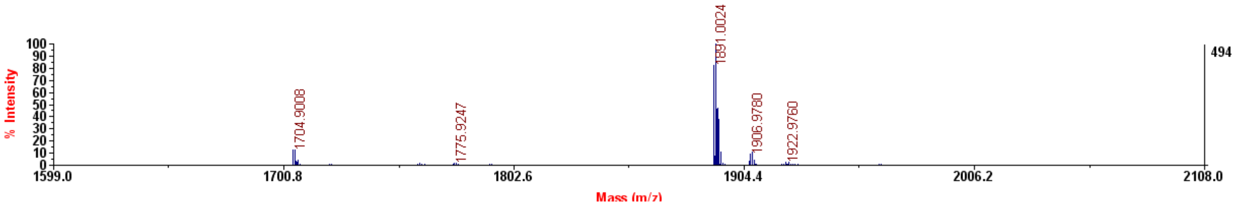

plate 64/line C/column 5

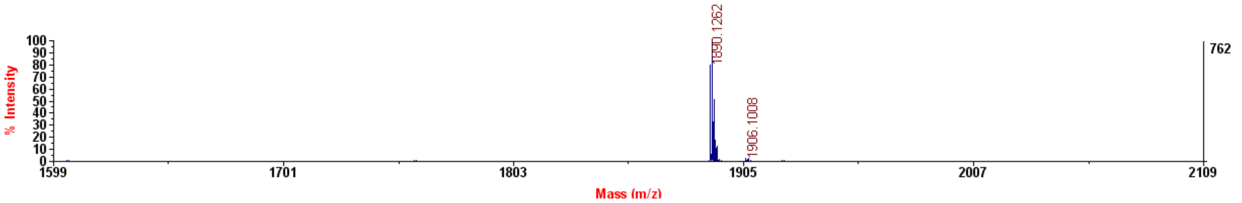

plate 64/line B/column 6

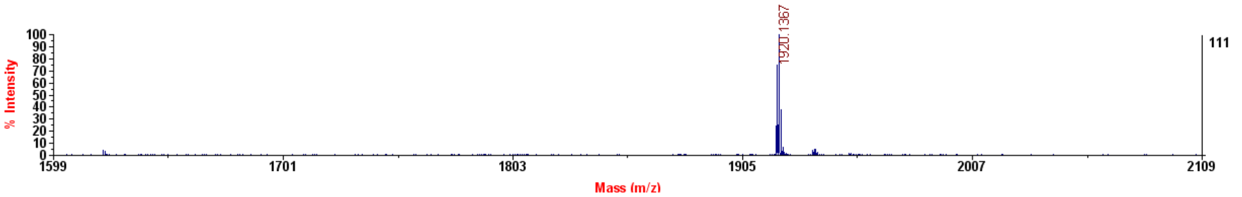

Supplementary Figure 120. MS spectra of plates 63 and 64. The data of B11 and D11 in plate 63 and C2, D4, C5, and B6 in plate 64 are shown.

plate 64/line G/column 6

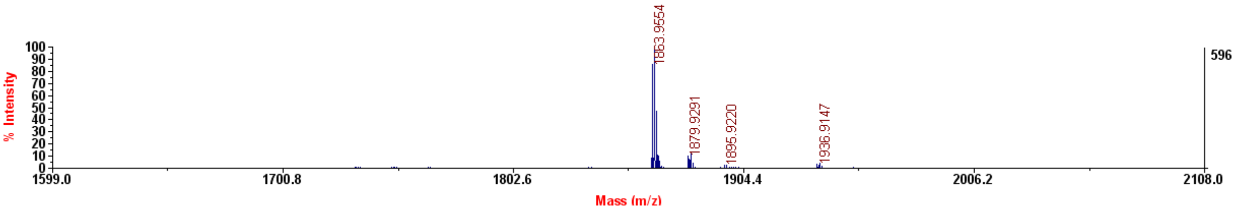

plate 64/line A/column 11

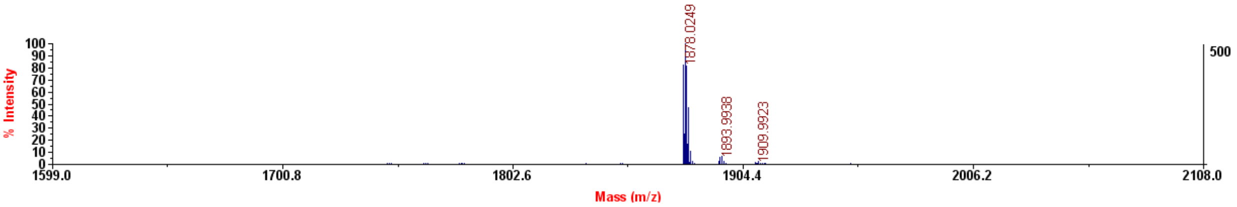

plate 65/line F/column 2

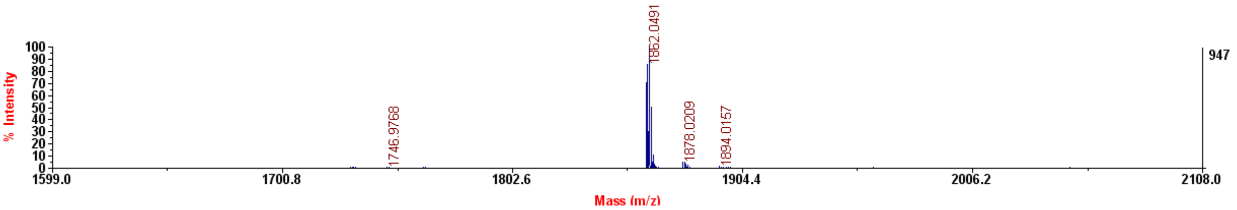

plate 65/line G/column 2

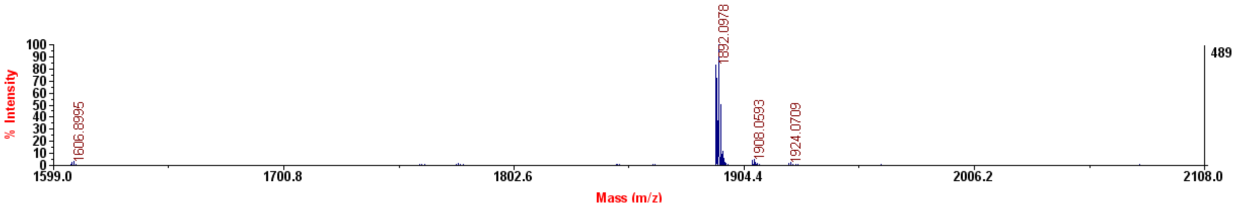

plate 65/line C/column 5

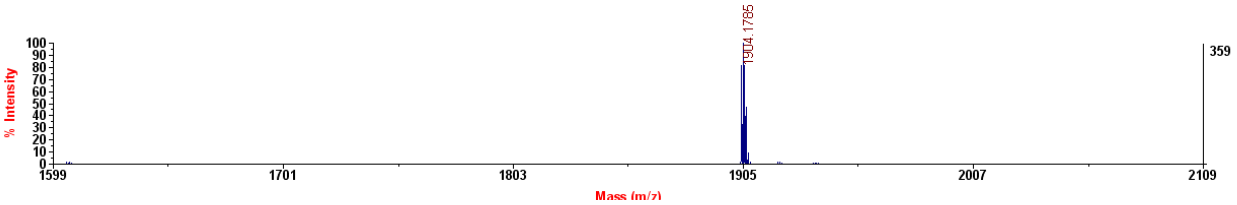

plate 65/line H/column 9

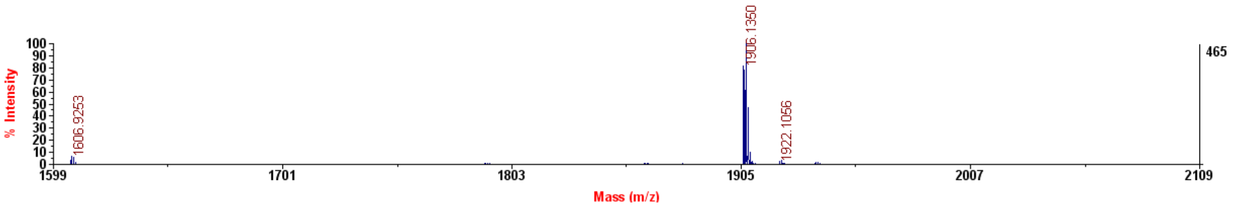

Supplementary Figure 121. MS spectra of plates 64 and 65. The data of G6 and A11 in plate 64 and F2, G2, C5, and H9 in plate 65 are shown.

plate 65/line F/column 10

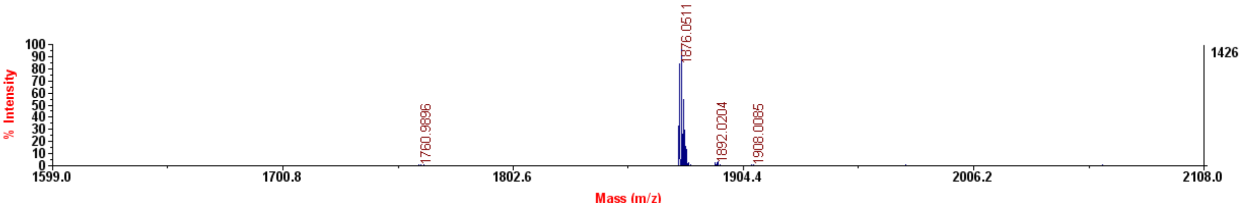

plate 65/line B/column 11

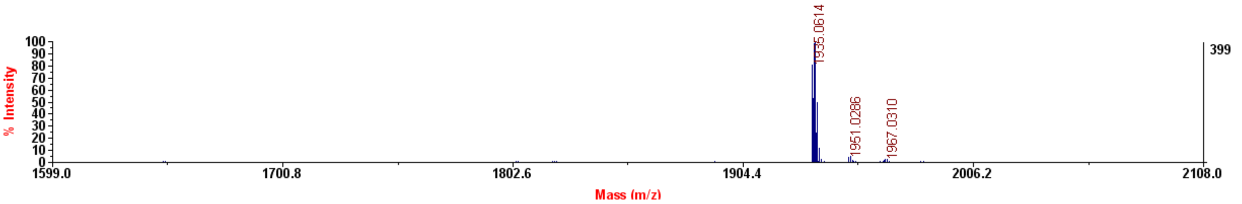

plate 65/line C/column 11

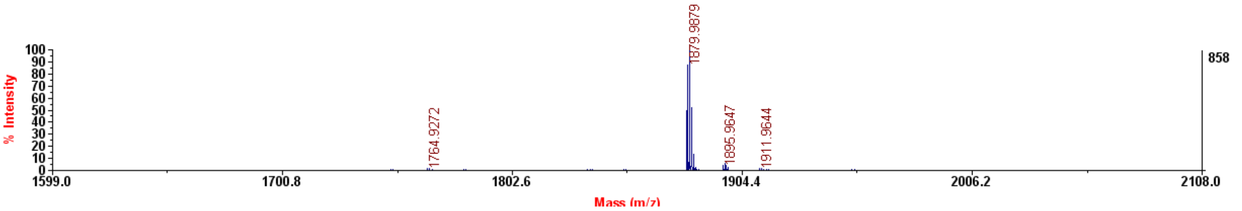

plate 66/line C/column 3

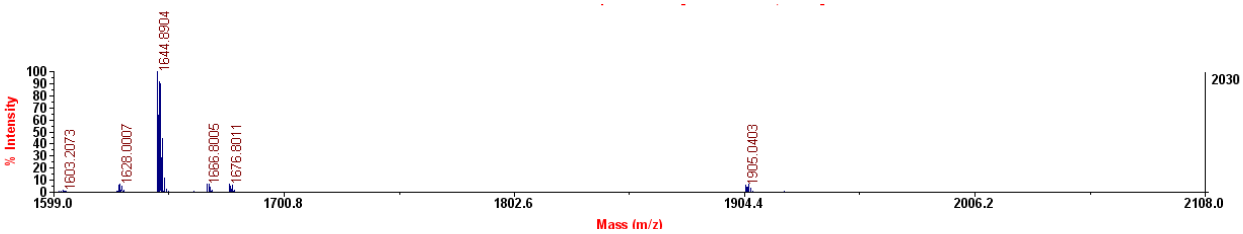

plate 66/line B/column 4

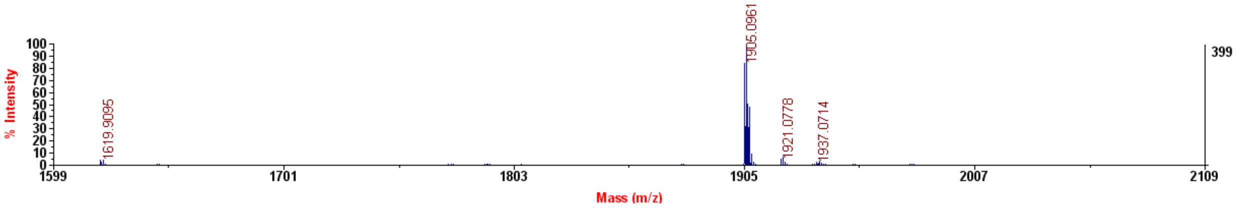

plate 66/line F/column 11

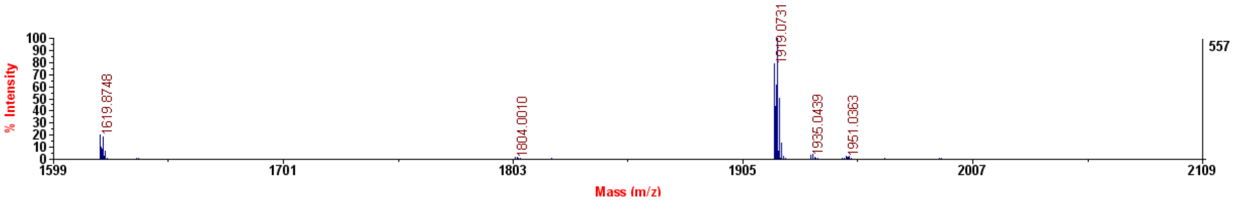

Supplementary Figure 122. MS spectra of plates 65 and 66. The data of F10, B11, and C11 in plate 65 and C3, B4, and F11 in plate 66 are shown.

plate 67/line H/column 4

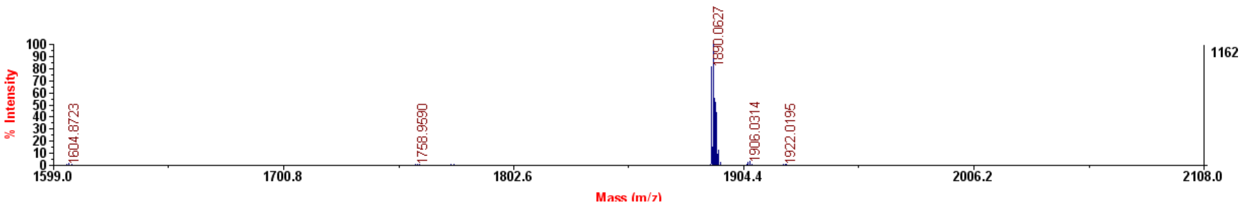

plate 68/line E/column 1

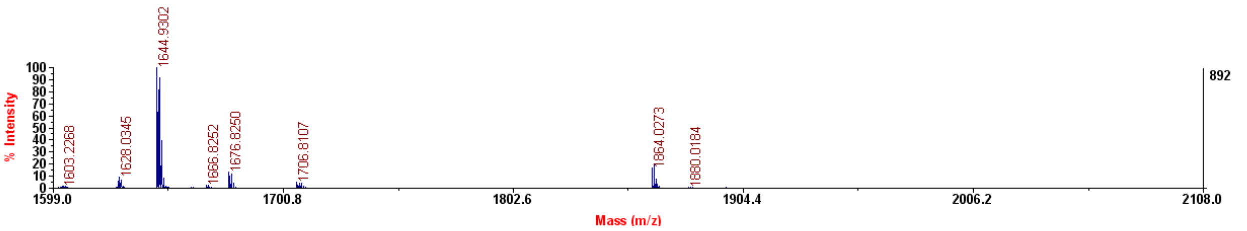

plate 68/line G/column 8

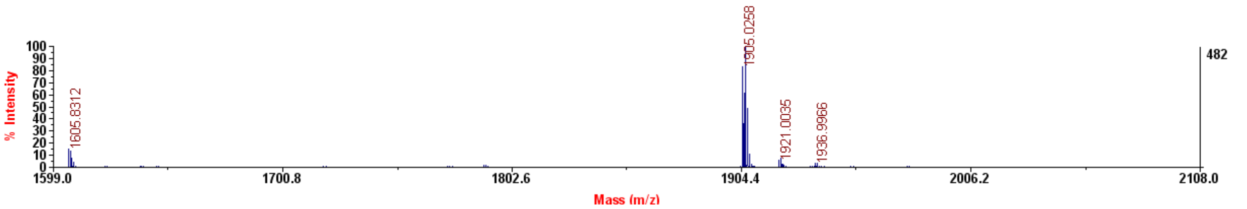

plate 68/line D/column 9

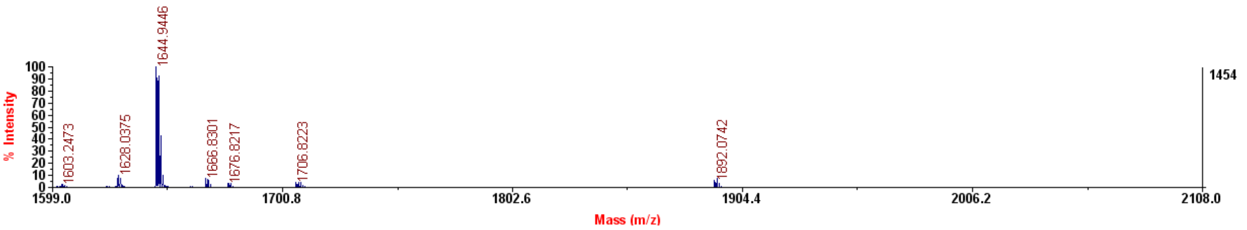

plate 69/line H/column 10

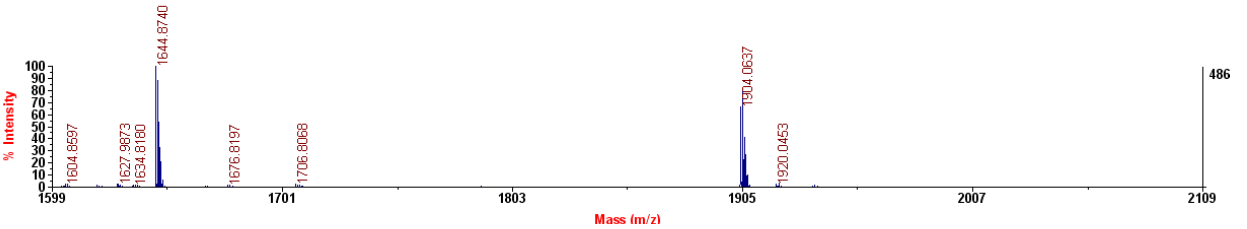

plate 70/line D/column 2

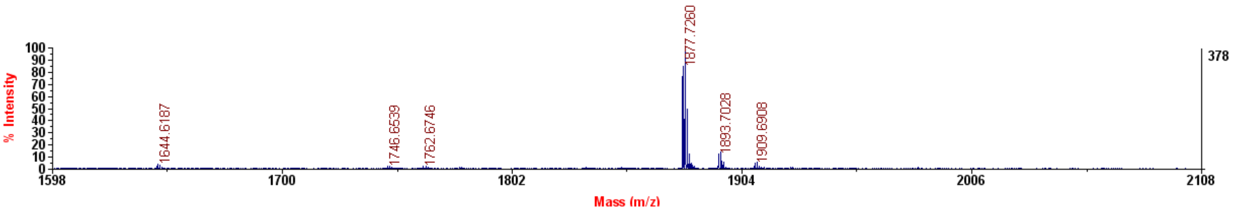

Supplementary Figure 123. MS spectra of plates 67, 68, 69, and 70. The data of H4 in plate 67, E1, G8, and D9 in plate 68, H10 in plate 69, and D2 in plate 70 are shown.

plate 70/line H/column 7

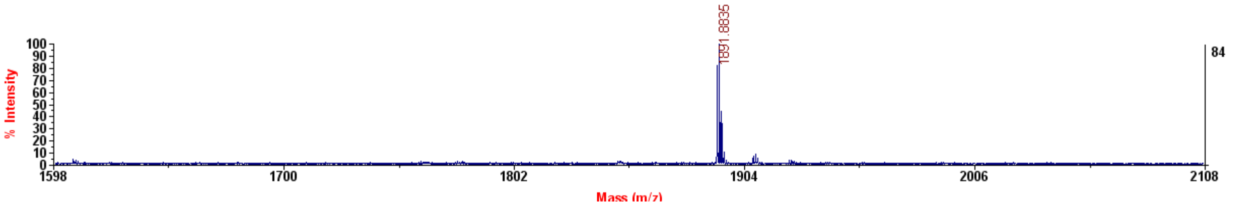

plate 70/line E/column 8

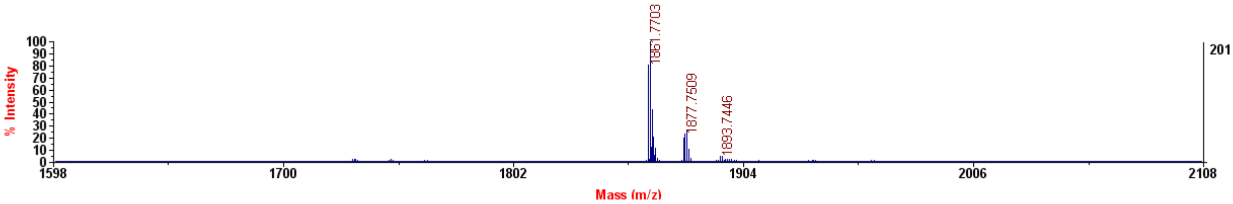

plate 71/line A/column 5

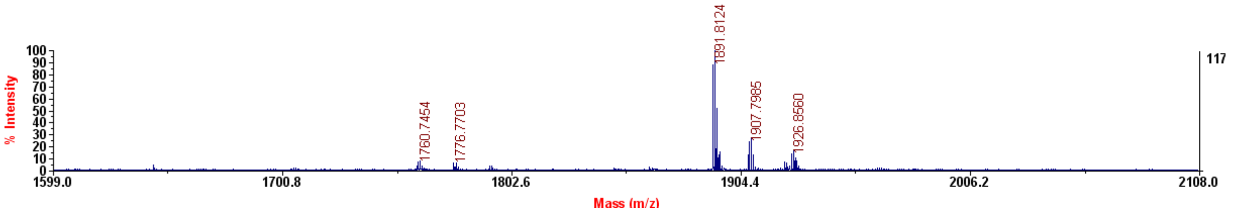

plate 71/line H/column 7

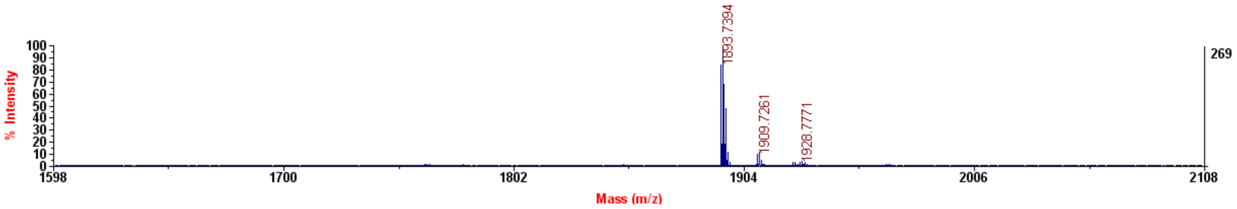

plate 71/line A/column 10

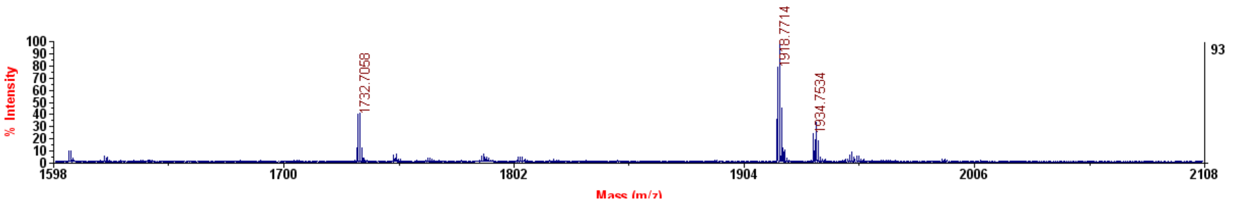

plate 72/line D/column 2

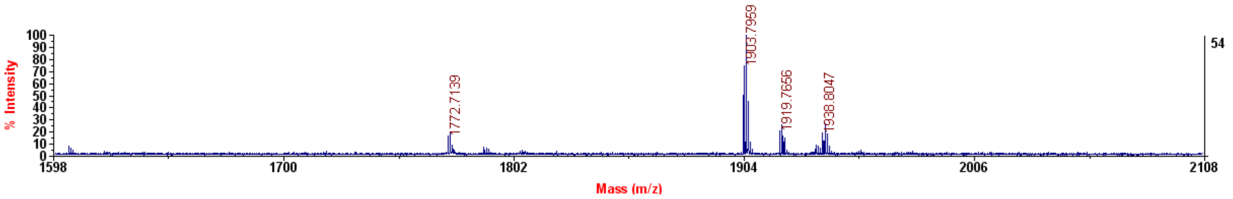

Supplementary Figure 124. MS spectra of plates 70, 71, and 72. The data of H7 and E8 in plate 70, A5, H7, and A10 in plate 71, and D2 in plate 72 are shown.

plate 72/line A/column 7

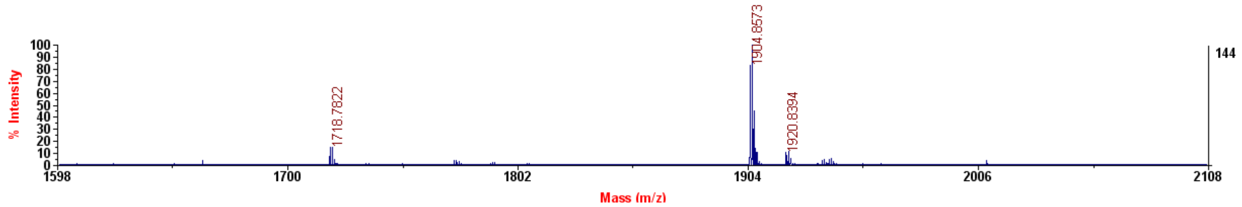

plate 72/line A/column 9

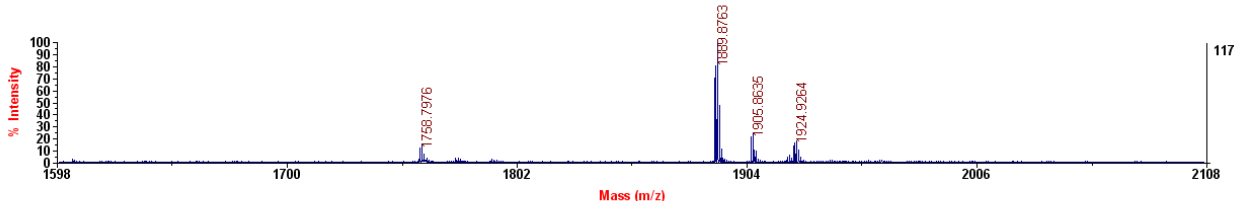

plate 72/line H/column 10

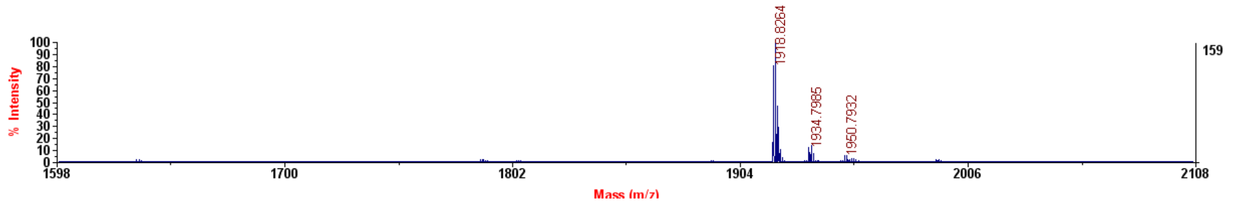

plate 74/line F/column 7

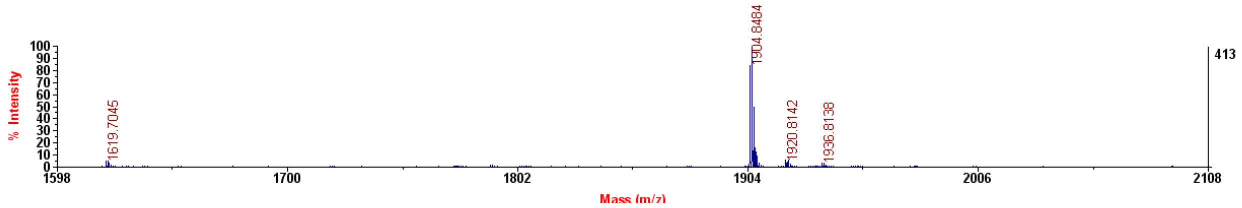

plate 74/line H/column 8

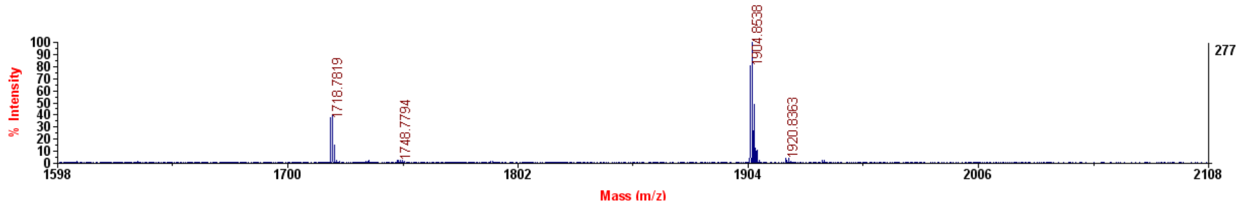

plate 74/line E/column 11

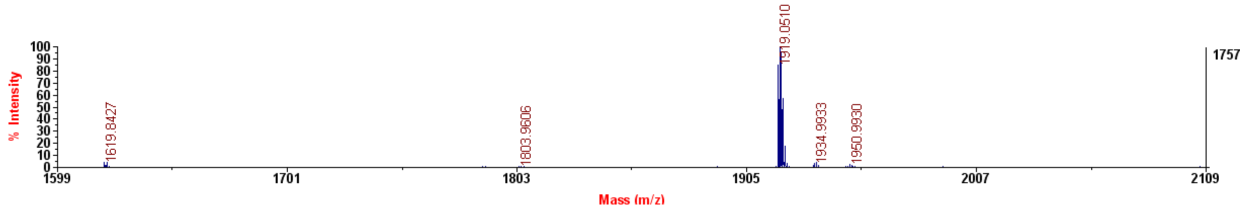

Supplementary Figure 125. MS spectra of plates 72 and 74. The data of A7, A9, and H10 in plate 72 and F7, H8, and E11 in plate 74 are shown.

plate 75/line E/column 1

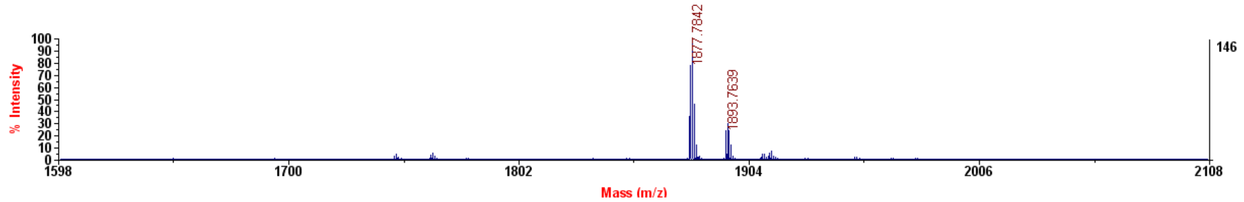

plate 75/line D/column 4

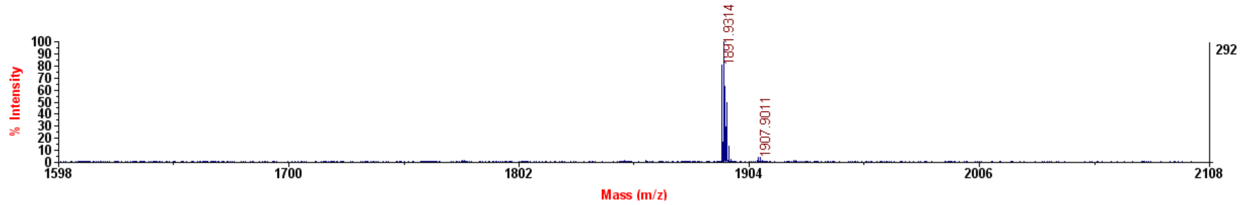

plate 75/line F/column 8

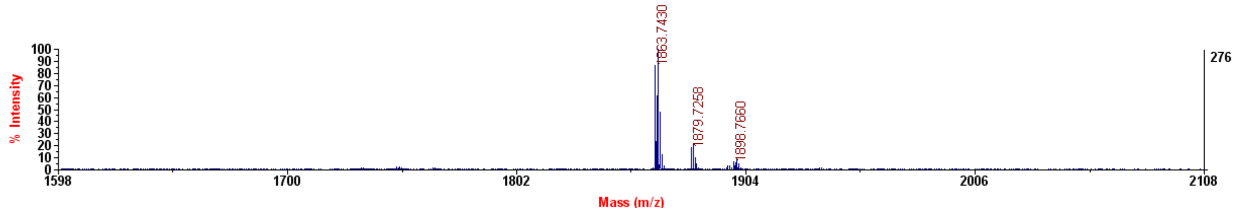

plate 76/line G/column 1

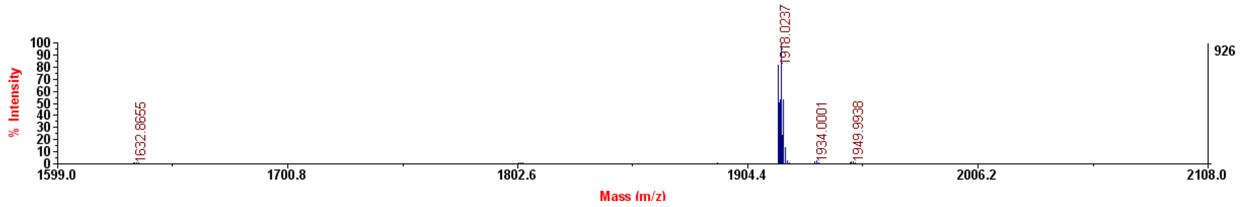

plate 76/line F/column 9

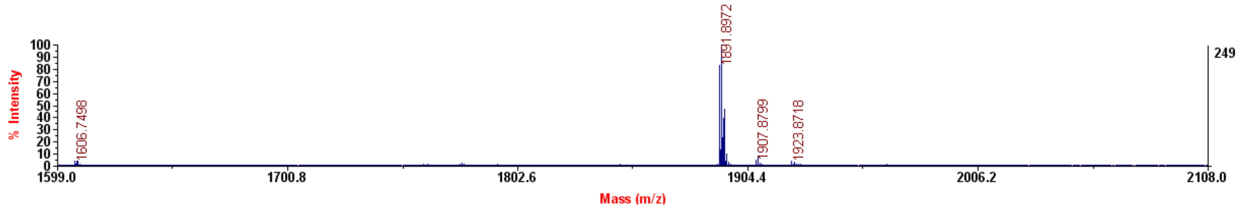

plate 76/line G/column 9

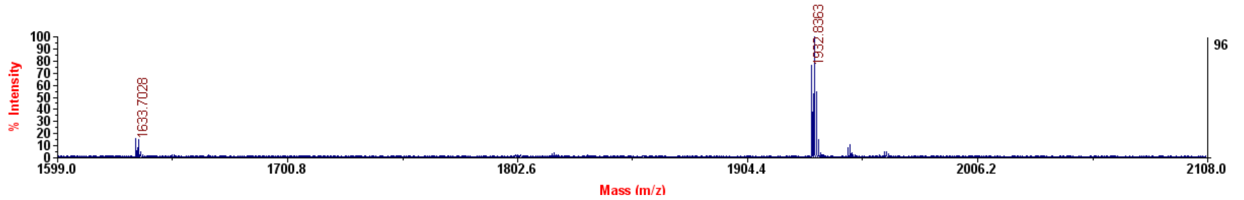

Supplementary Figure 126. MS spectra of plates 75 and 76. The data of E1, D4, and F8 in plate 75 and G1, F9, and G9 in plate 76 are shown.

plate 77/line F/column 6

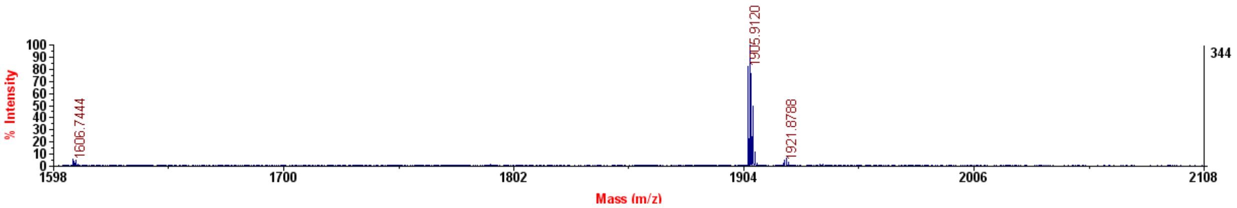

plate 77/line E/column 8

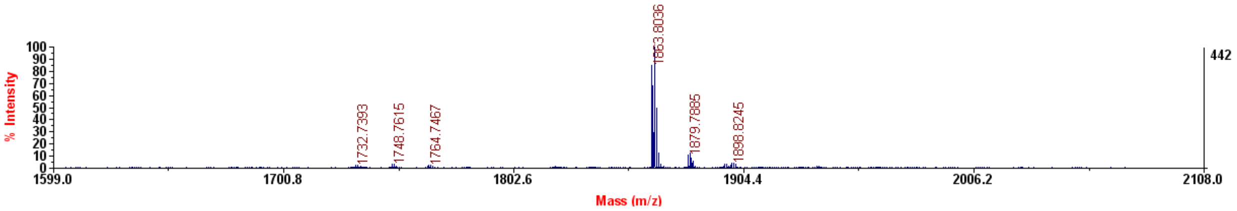

plate 77/line E/column 10

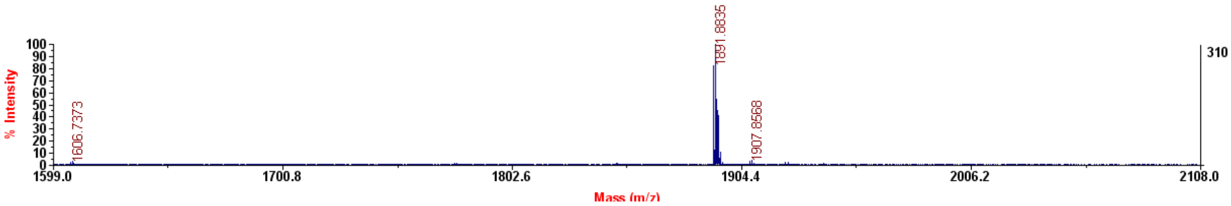

plate 77/line H/column 11

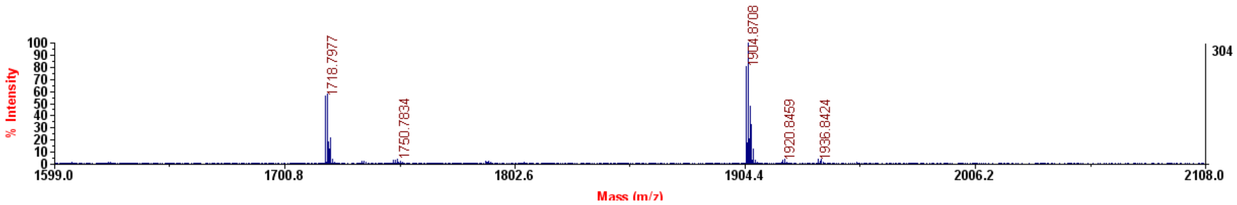

plate 78/line D/column 2

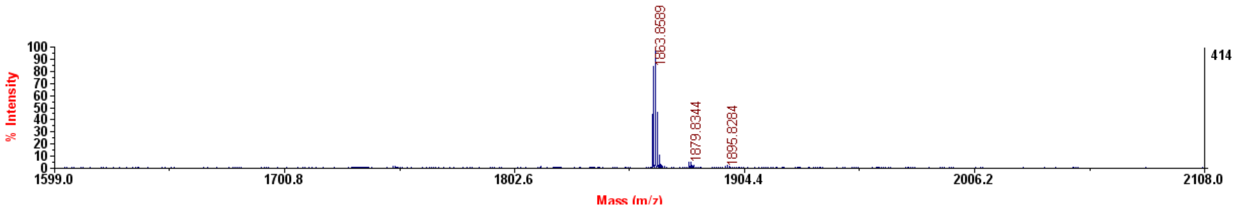

plate 78/line F/column 2

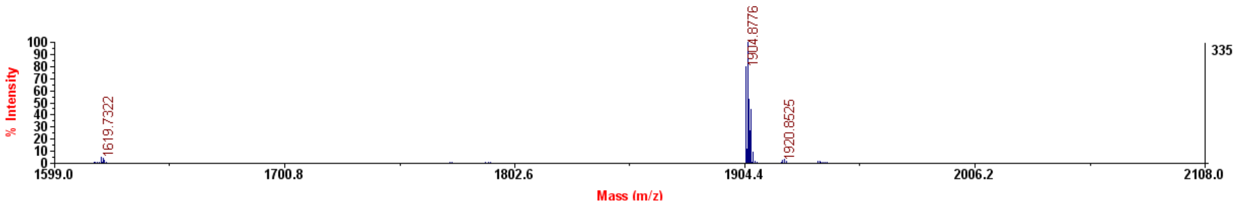

Supplementary Figure 127. MS spectra of plates 77 and 78. The data of F6, E8, E10, and H11 in plate 77 and D2 and F2 in plate 78 are shown.

plate 78/line E/column 3

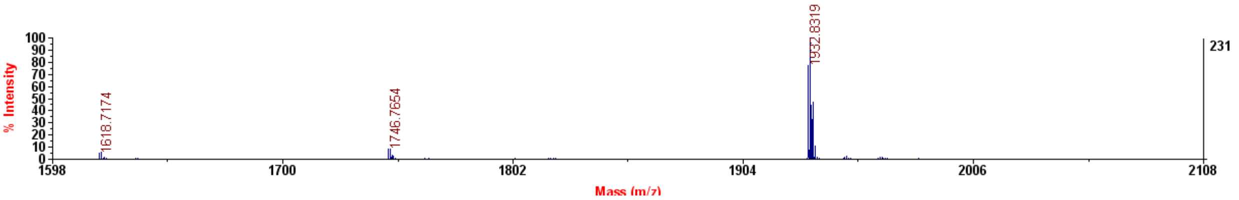

plate 78/line B/column 5

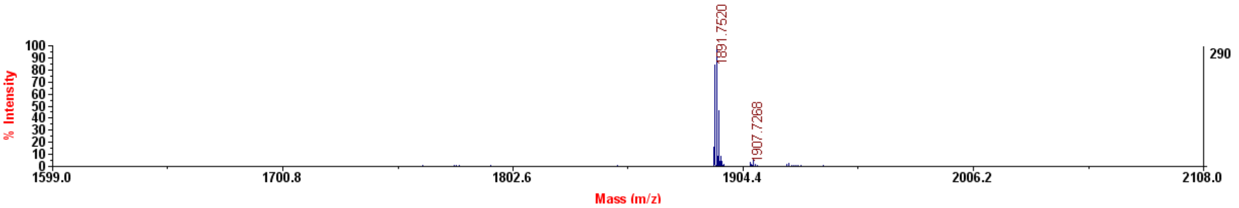

plate 78/line C/column 5

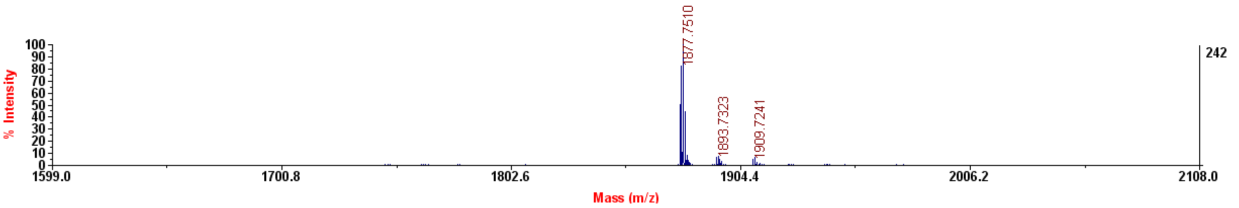

plate 78/line C/column 6

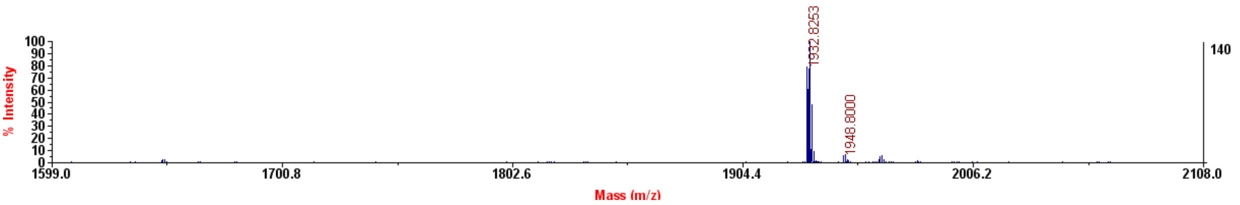

plate 78/line A/column 7

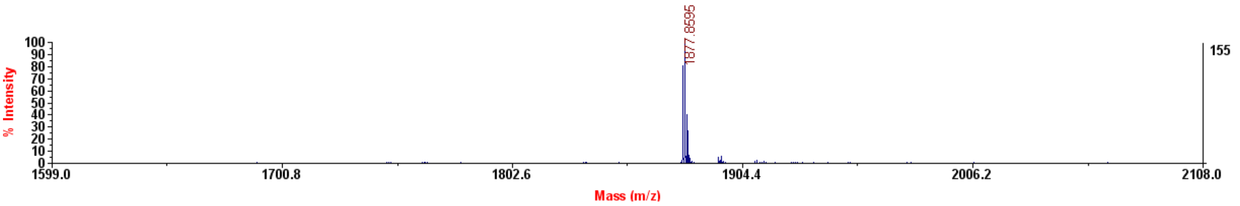

plate 78/line E/column 7

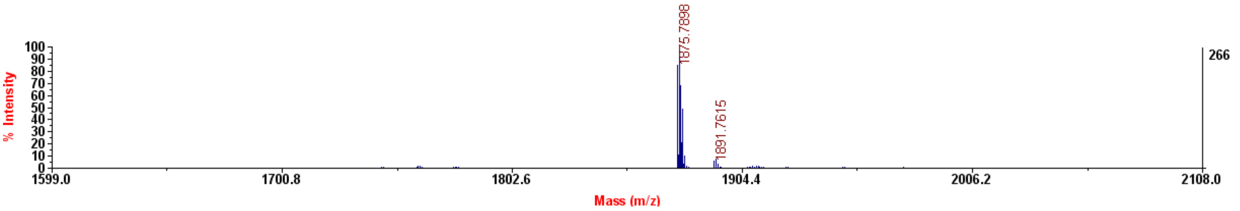

Supplementary Figure 128. MS spectra of plate 78. The data of E3, B5, C5, C6, A7, and E7 in plate 78 are shown.

plate 78/line F/column 7

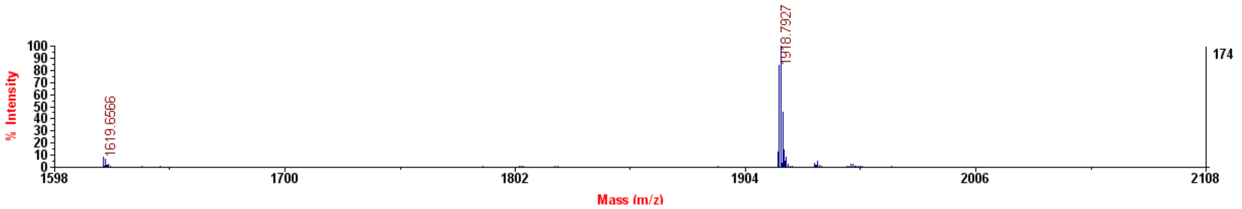

plate 78/line A/column 9

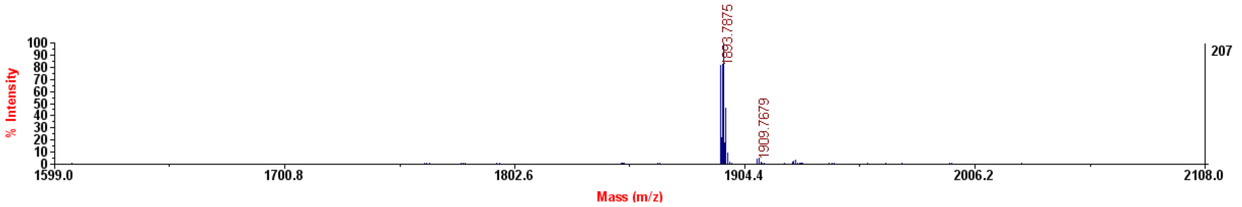

plate 78/line D/column 9

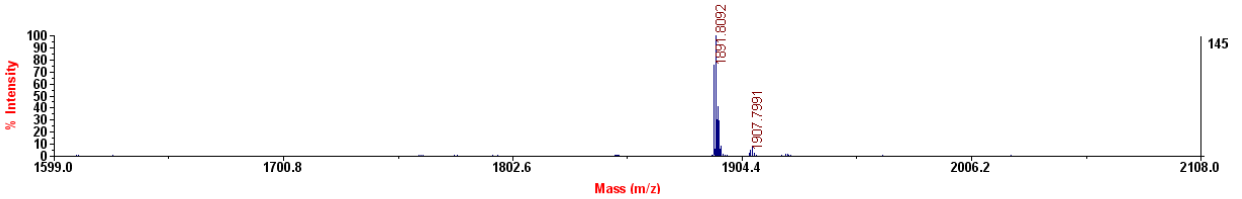

plate 78/line H/column 10

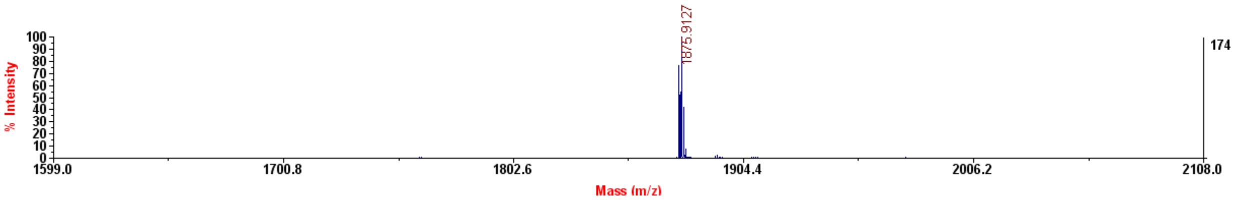

plate 78/line A/column 11

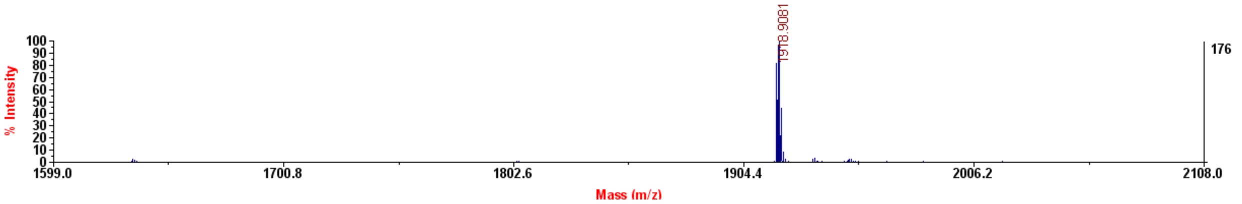

plate 78/line H/column 11

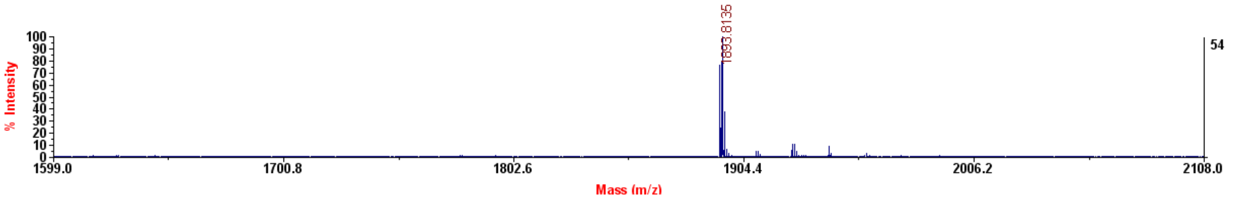

Supplementary Figure 129. MS spectra of plate 78. The data of F7, A9, D9, H10, A11, and H11 in plate 78 are shown.

plate 79/line C/column 1

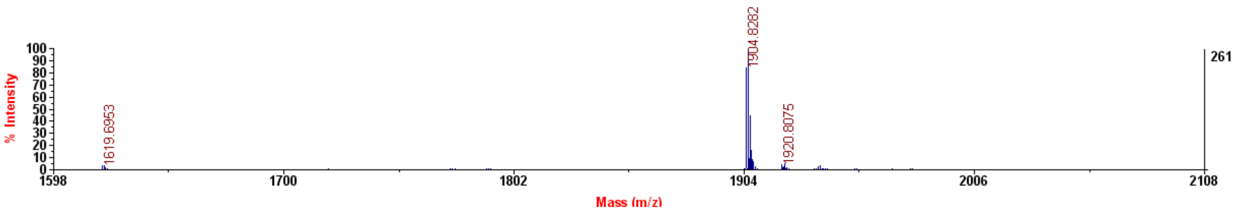

plate 79/line G/column 6

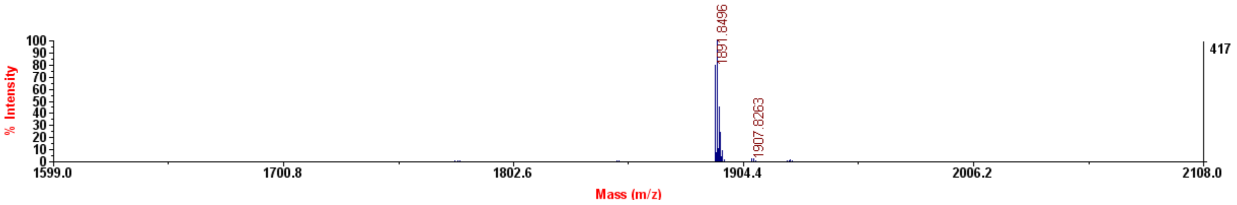

plate 79/line F/column 7

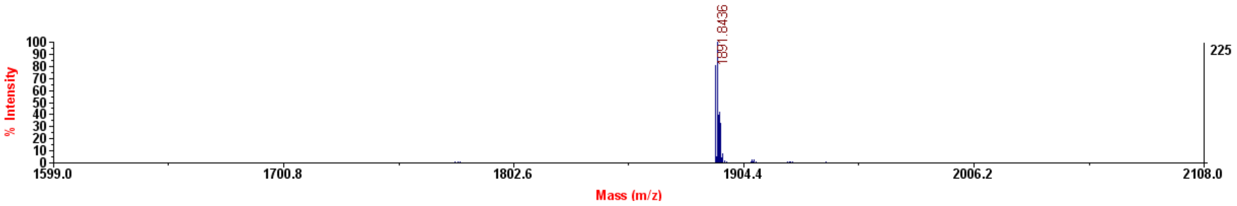

plate 79/line G/column 8

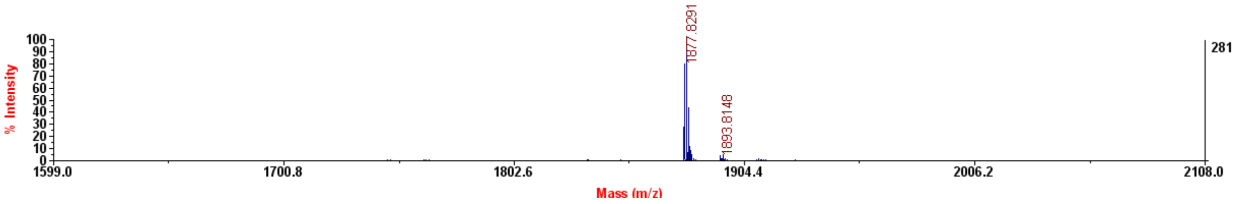

plate 80/line B/column 4

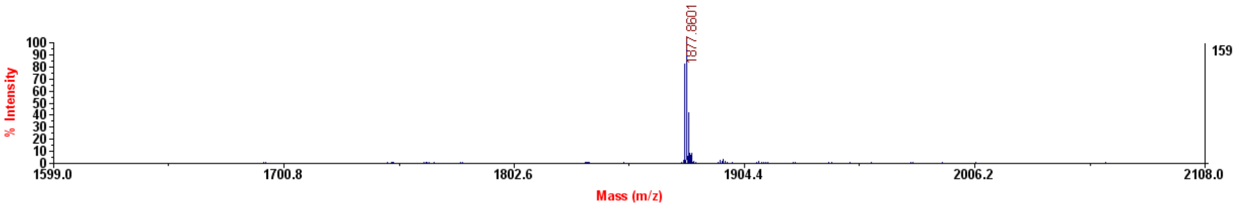

plate 80/line G/column 4

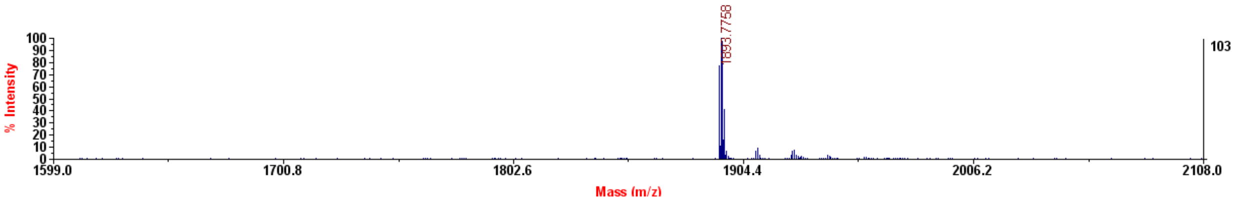

Supplementary Figure 130. MS spectra of plates 79 and 80. The data of C1, G6, F7, and G8 in plate 79 and B4 and G4 in plate 80 are shown.

plate 80/line A/column 9

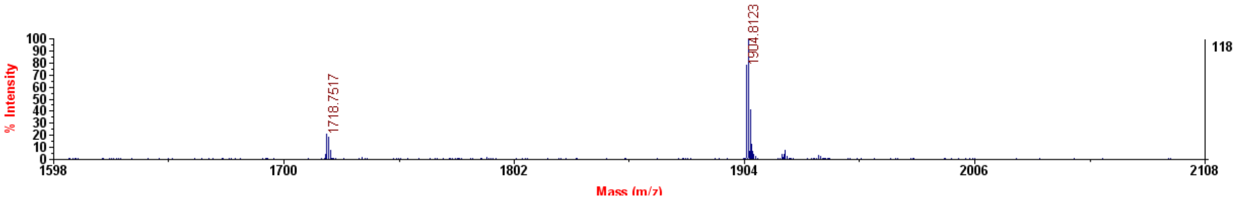

plate 80/line B/column 9

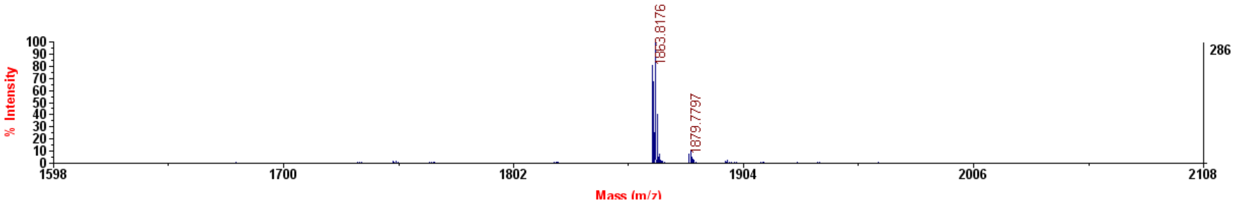

plate 80/line E/column 10

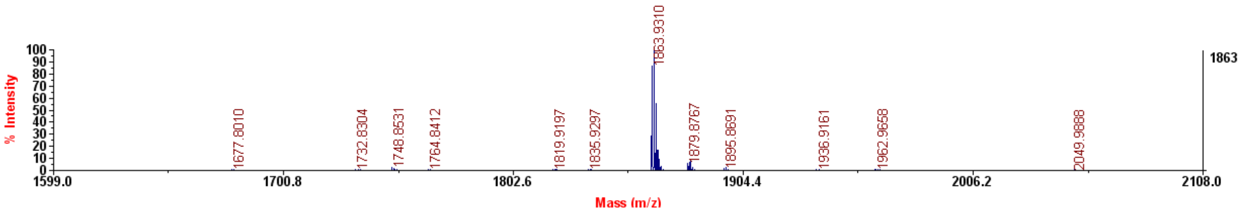

plate 80/line C/column 11

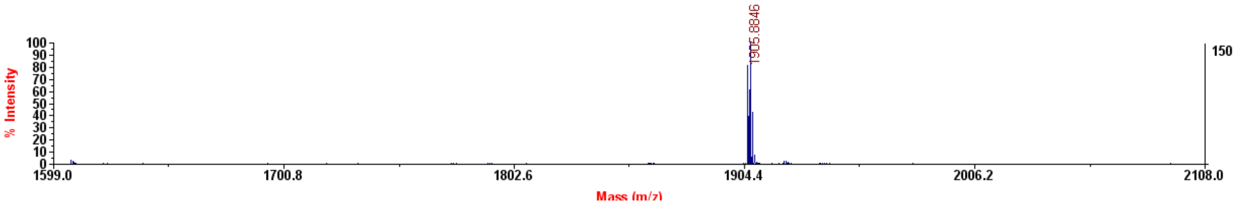

plate 81/line D/column 4

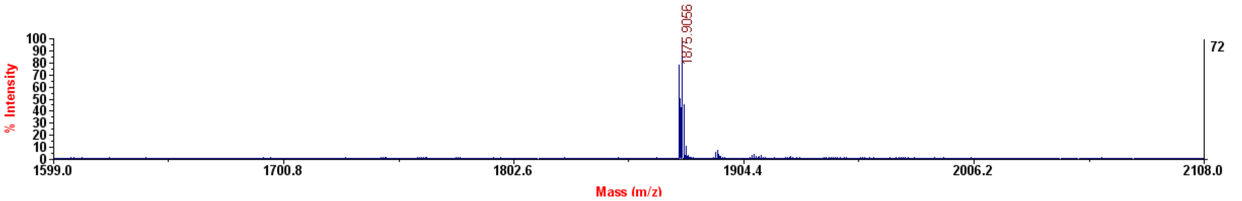

plate 81/line G/column 6

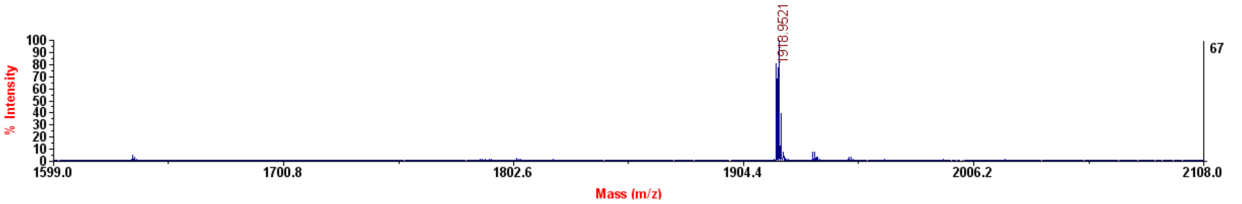

Supplementary Figure 131. MS spectra of plates 80 and 81. The data of A9, B9, E10, and C11 in plate 80 and D4 and G6 in plate 81 are shown.

plate 81/line C/column 8

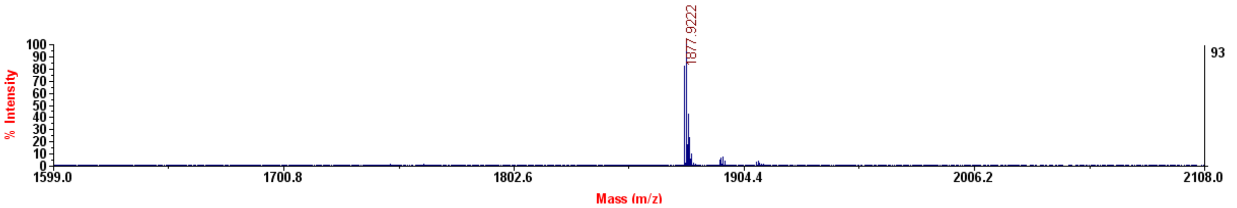

plate 81/line G/column 8

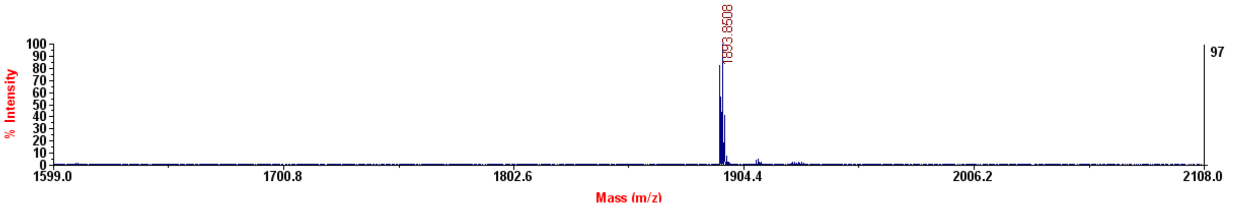

plate 81/line B/column 10

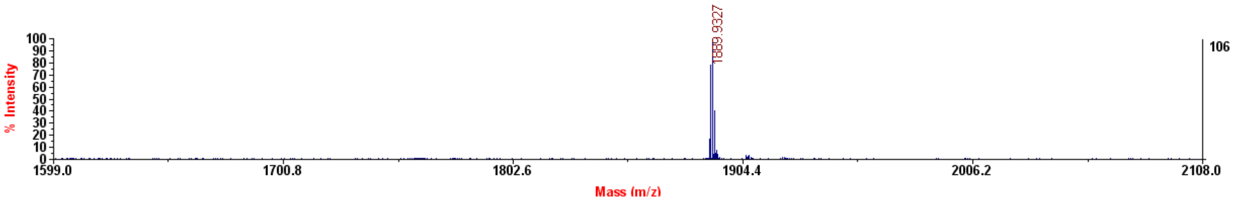

plate 81/line D/column 11

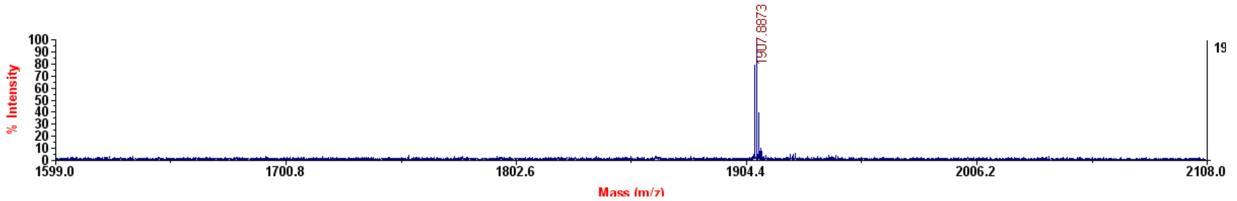

plate 82/line A/column 2

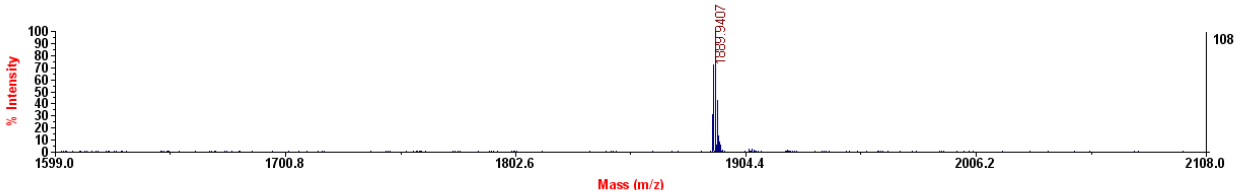

plate 82/line G/column 4

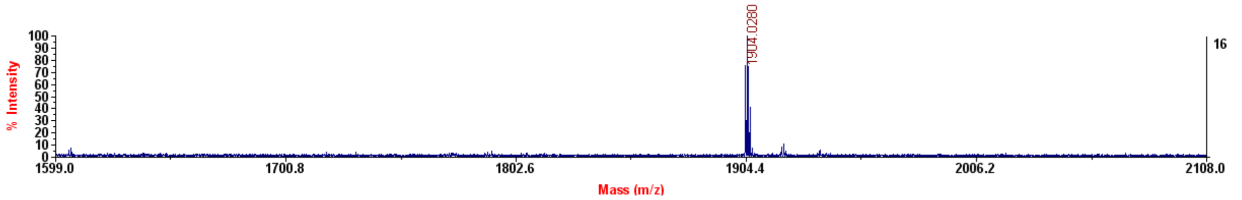

Supplementary Figure 132. MS spectra of plates 81 and 82. The data of C8, G8, B10, and D11 in plate 81 and A2 and G4 in plate 82 are shown.

plate 82/line F/column 5

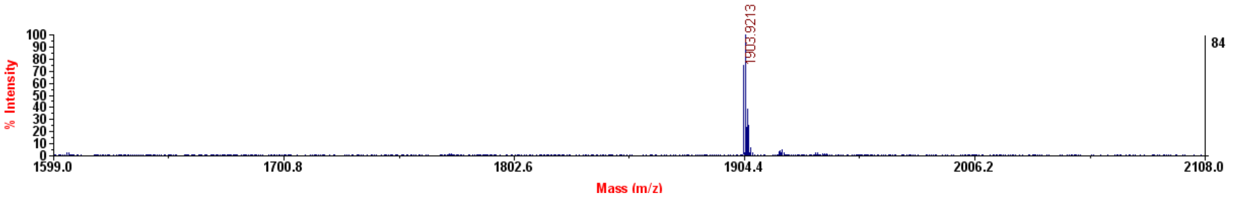

plate 82/line D/column 6

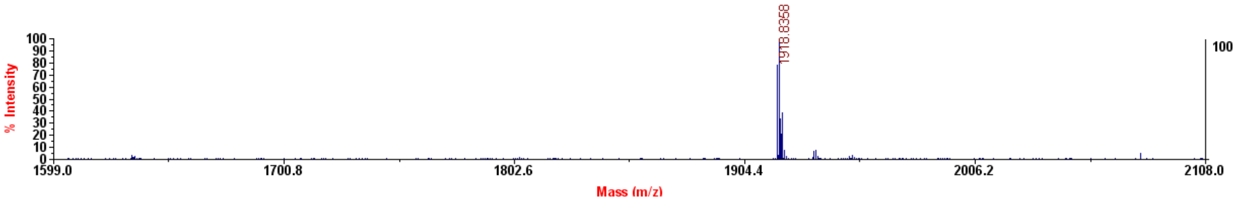

plate 82/line C/column 7

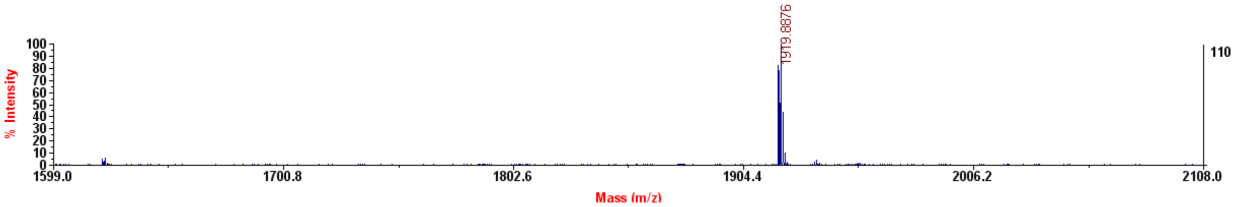

plate 82/line A/column 9

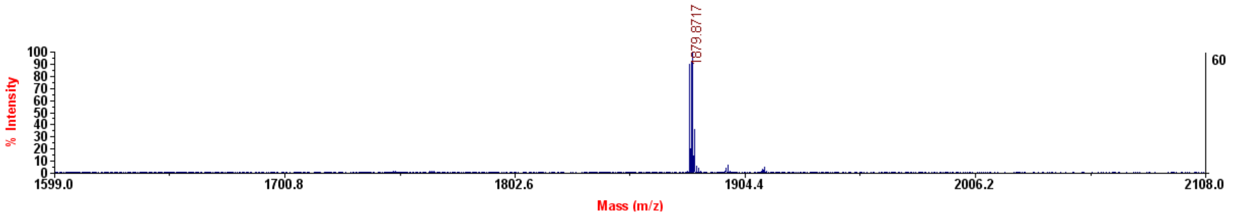

plate 82/line D/column 9

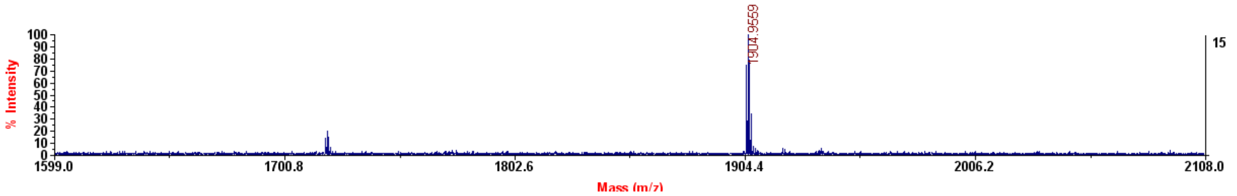

plate 83/line E/column 1

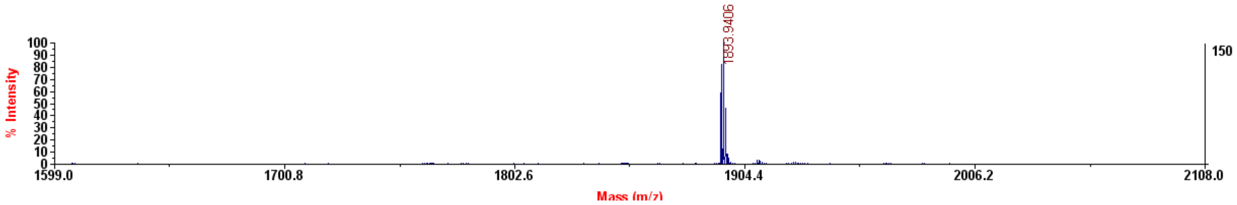

Supplementary Figure 133. MS spectra of plates 82 and 83. The data of F5, D6, C7, A9, and D9 in plate 82 and E1 in plate 83 are shown.

plate 83/line G/column 3

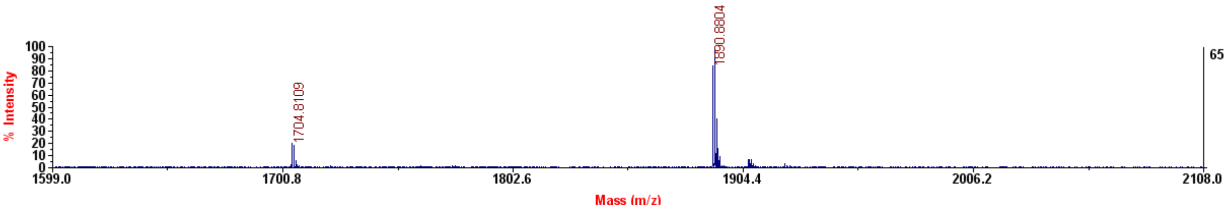

plate 83/line H/column 9

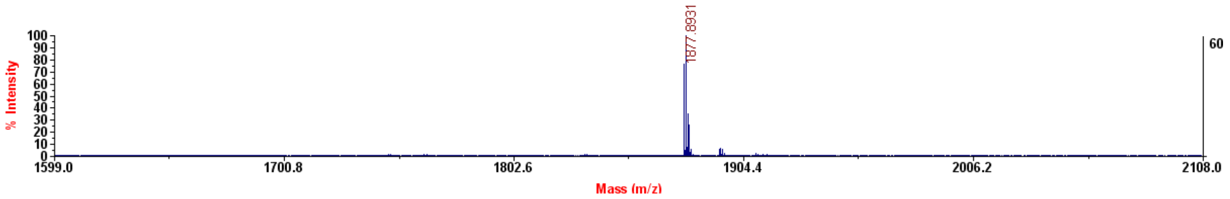

plate 83/line E/column 10

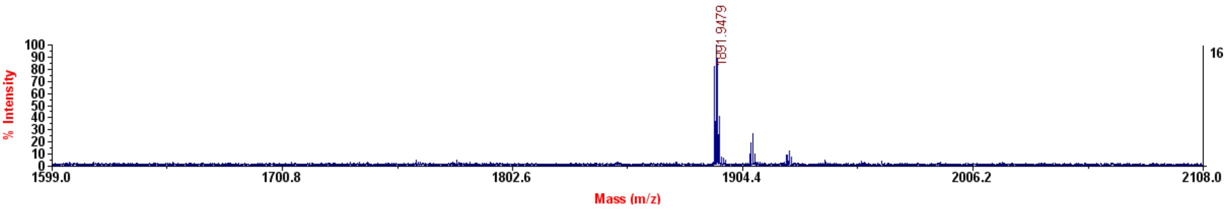

plate 83/line F/column 11

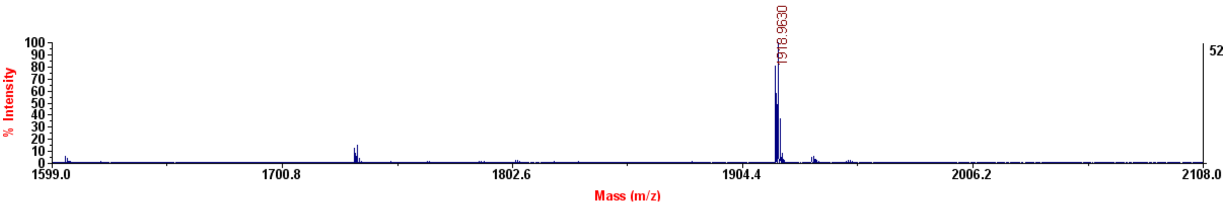

plate 84/line A/column 8

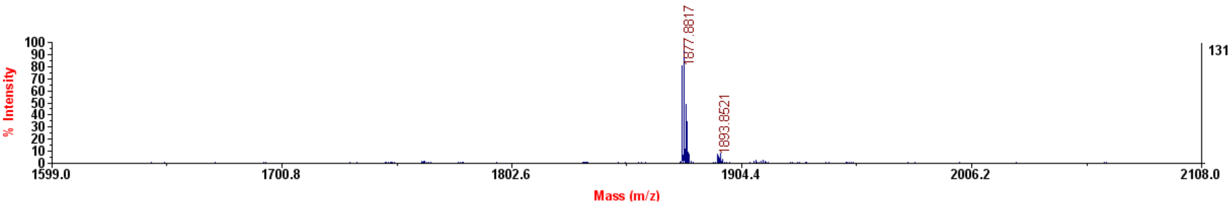

plate 84/line H/column 8

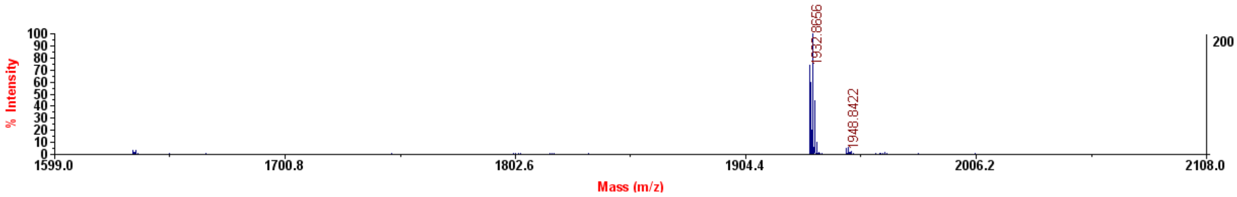

Supplementary Figure 134. MS spectra of plates 83 and 84. The data of G3, H9, E10, and F11 in plate 83 and A8 and H8 in plate 84 are shown.

plate 84/line H/column 9

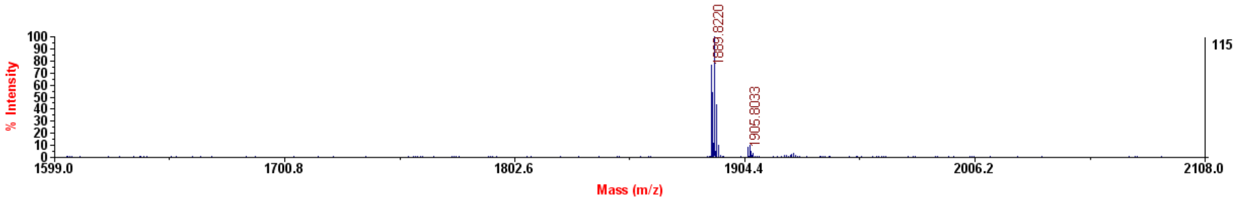

plate 85/line E/column 2

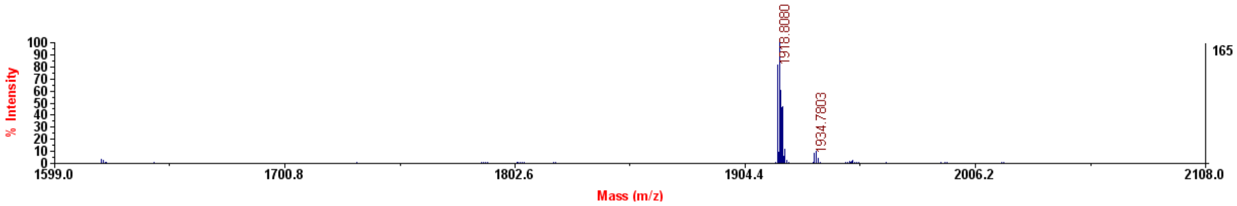

plate 85/line G/column 7

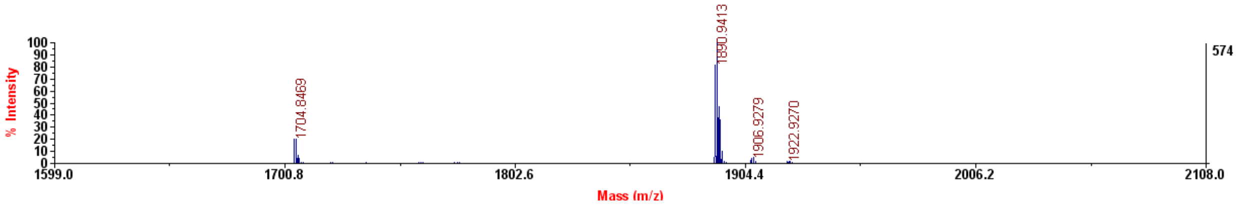

plate 85/line H/column 7

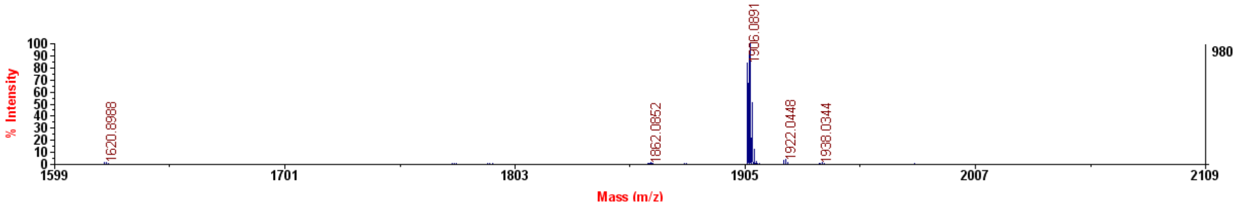

plate 85/line H/column 11

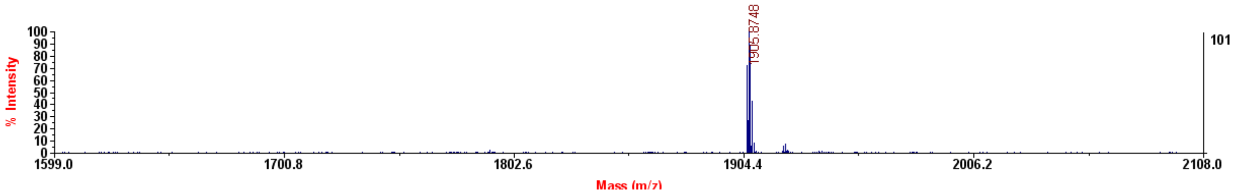

plate 86/line D/column 1

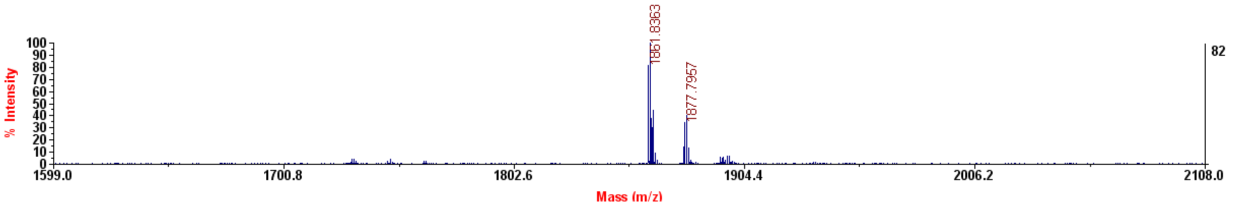

Supplementary Figure 135. MS spectra of plates 84, 85, and 86. The data of H9 in plate 84, E2, G7, H7, and H11 in plate 85, and D1 in plate 86 are shown.

plate 86/line D/column 3

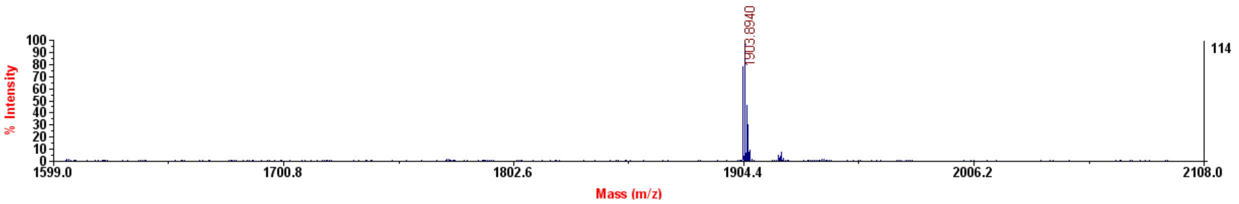

plate 86/line D/column 5

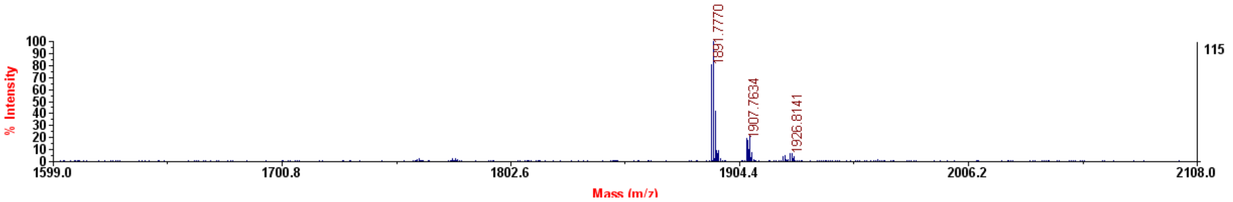

plate 88/line B/column 1

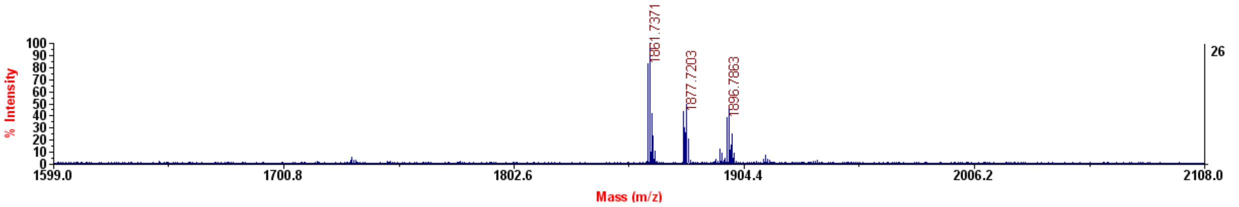

plate 88/line F/column 2

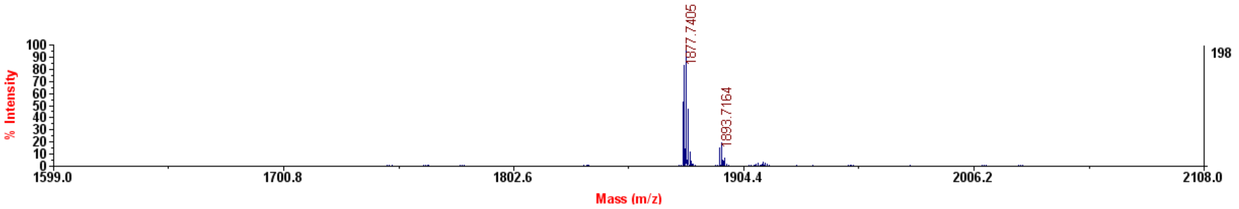

plate 89/line H/column 4

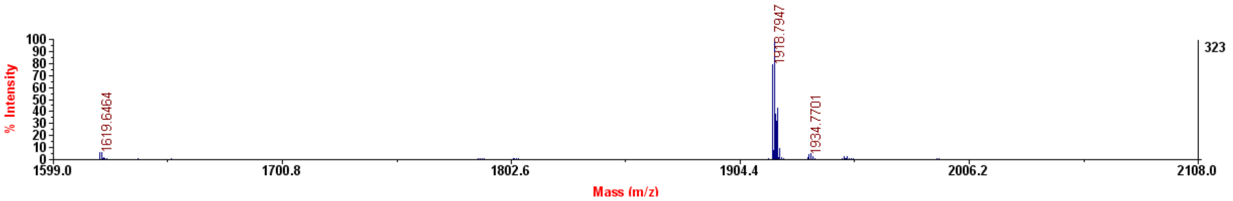

plate 89/line H/column 8

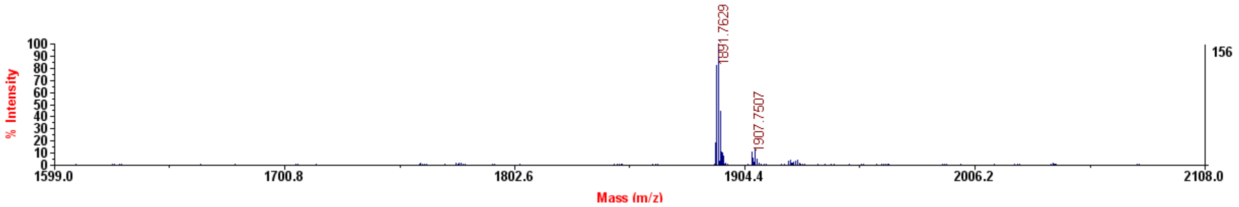

Supplementary Figure 136. MS spectra of plates 86, 88, and 89. The data of D3 and D5 in plate 86, B1 and F2 in plate 88, and H4 and H8 in plate 89 are shown.

plate 89/line E/column 9

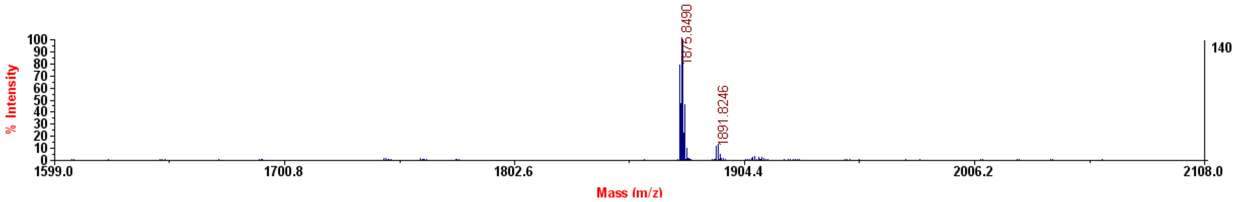

plate 89/line H/column 11

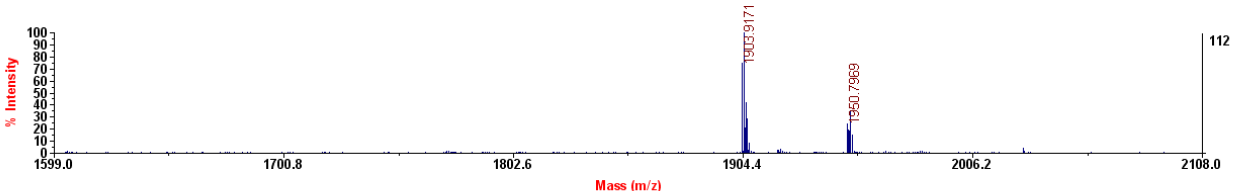

plate 90/line B/column 4

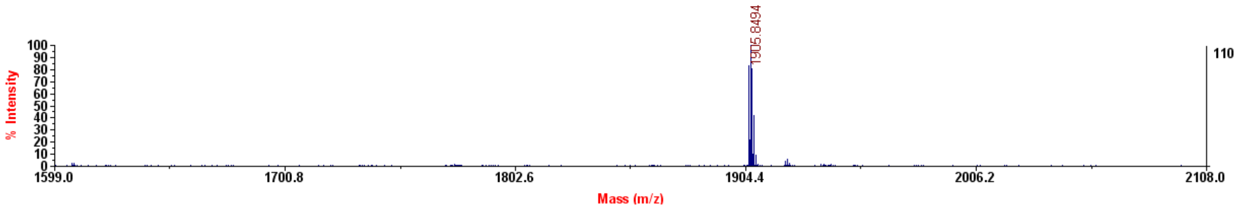

plate 90/line A/column 6

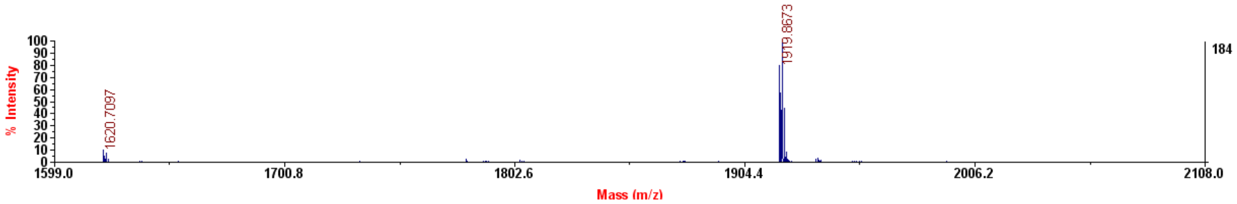

plate 90/line H/column 6

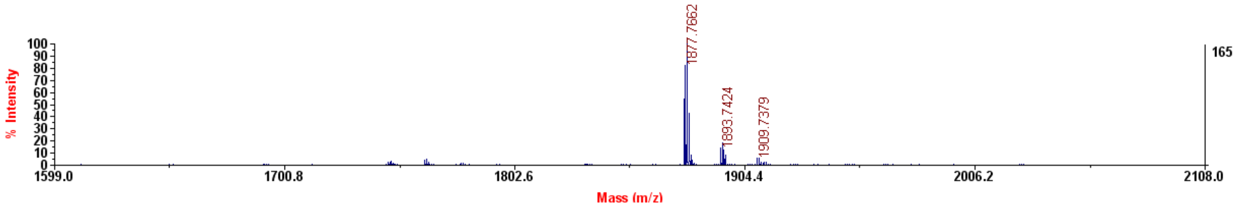

plate 90/line B/column 10

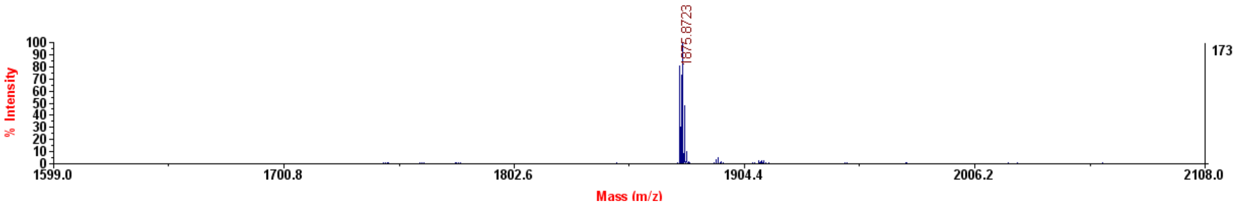

Supplementary Figure 137. MS spectra of plates 89 and 90. The data of E9 and H11 in plate 89 and B4, A6, H6, and B10 in plate 90 are shown.

plate 90/line D/column 11

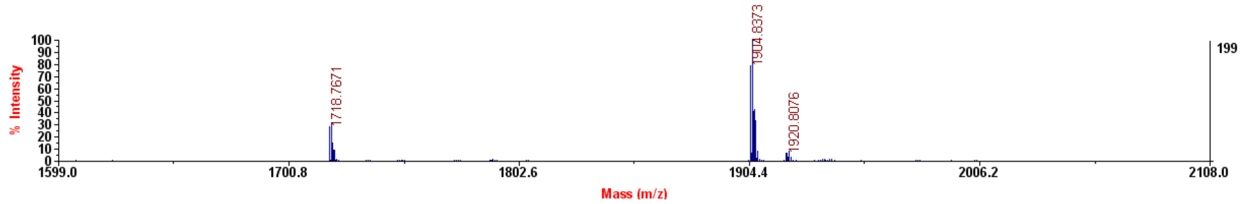

plate 91/line F/column 11

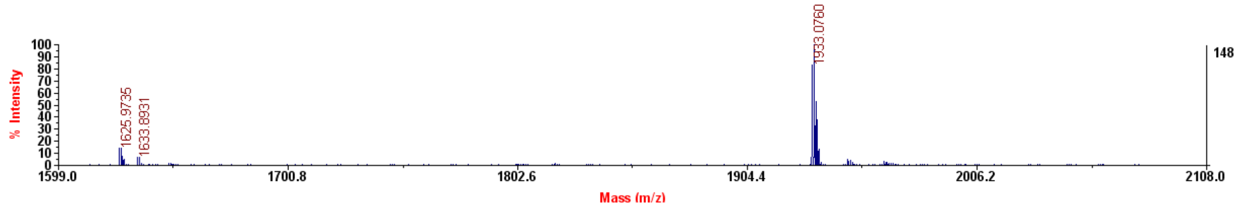

plate 92/line C/column 1

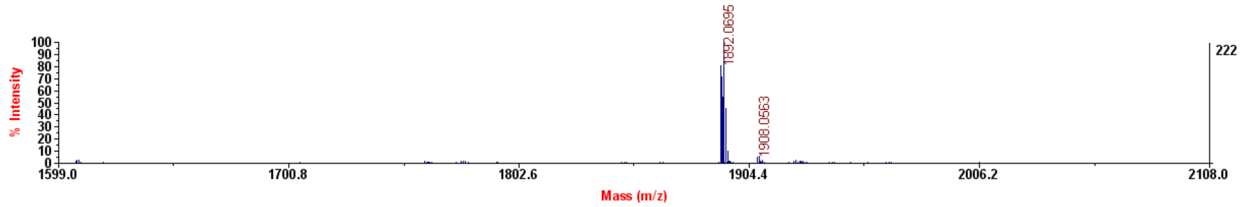

plate 92/line C/column 2

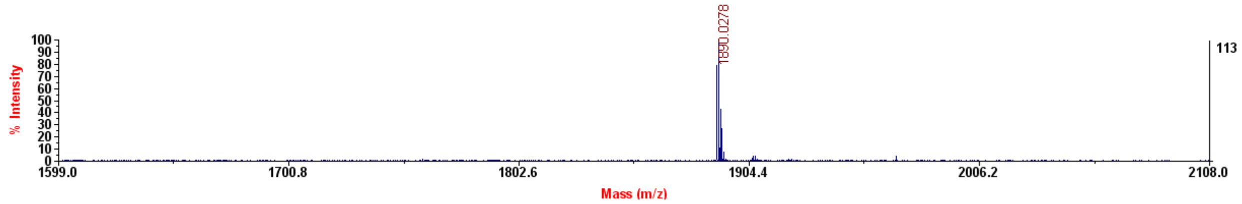

plate 92/line A/column 3

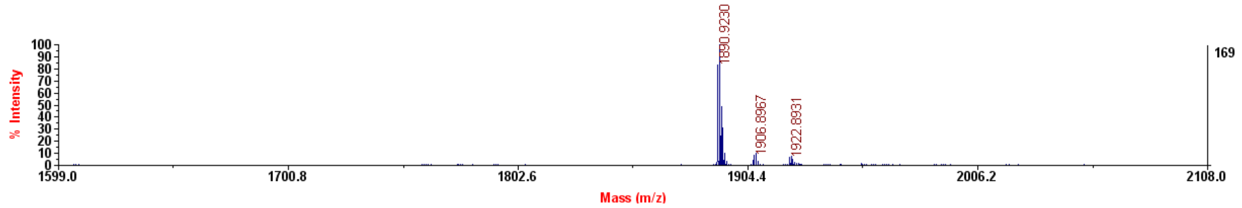

plate 92/line E/column 3

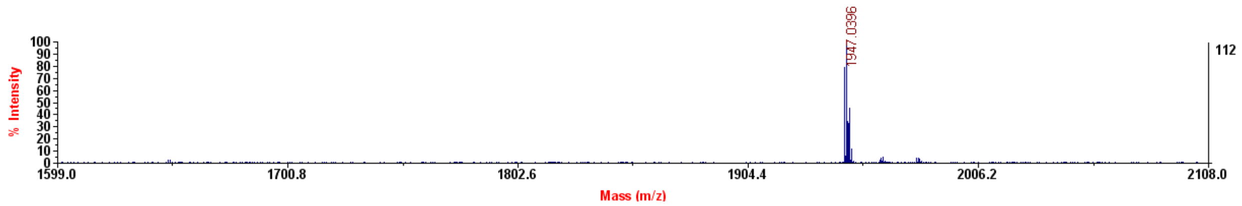

Supplementary Figure 138. MS spectra of plates 90, 91, and 92. The data of D11 in plate 90, F11 in plate 91, and C1, C2, A3, and E3 in plate 92 are shown.

plate 93/line B/column 1

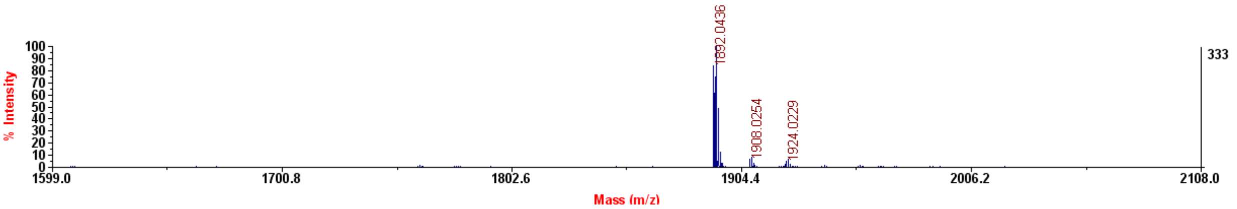

plate 93/line E/column 2

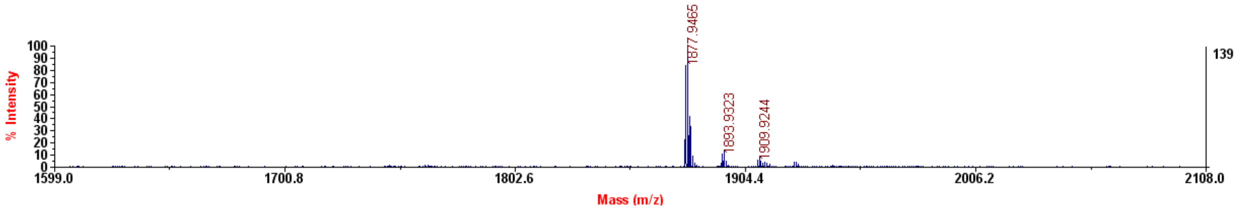

plate 94/line A/column 7

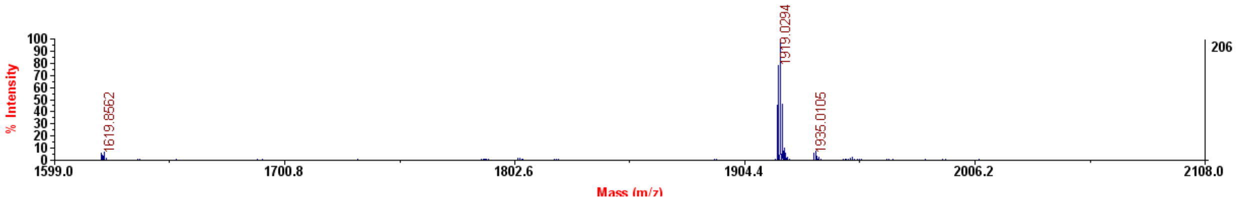

plate 94/line H/column 9

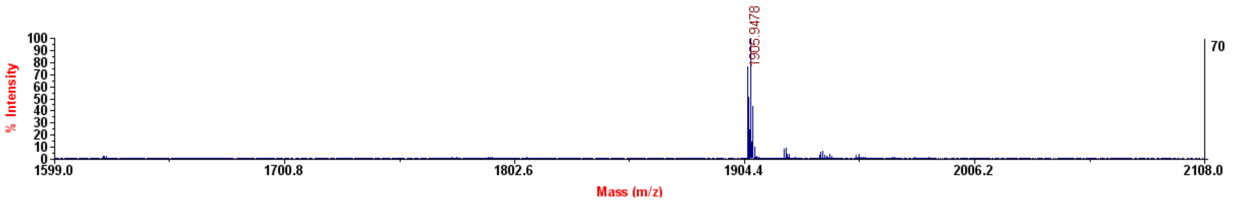

plate 95/line F/column 5

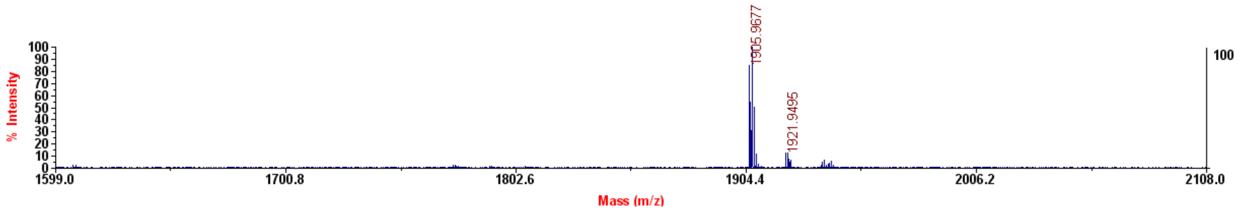

plate 95/line F/column 9

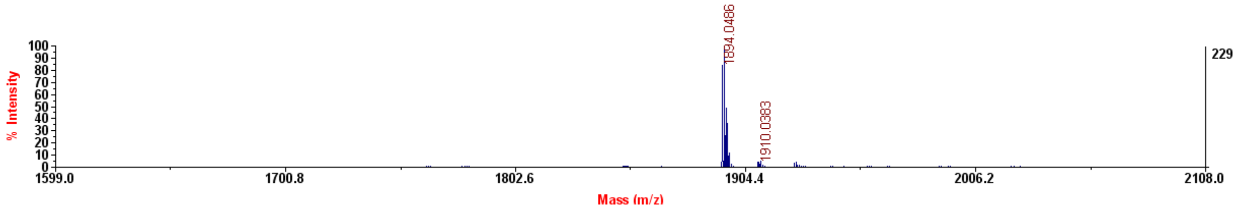

Supplementary Figure 139. MS spectra of plates 93, 94, and 95. The data of B1 and E2 in plate 93, A7 and H9 in plate 94, and F5 and F9 in plate 95 are shown.

plate 96/line E/column 1

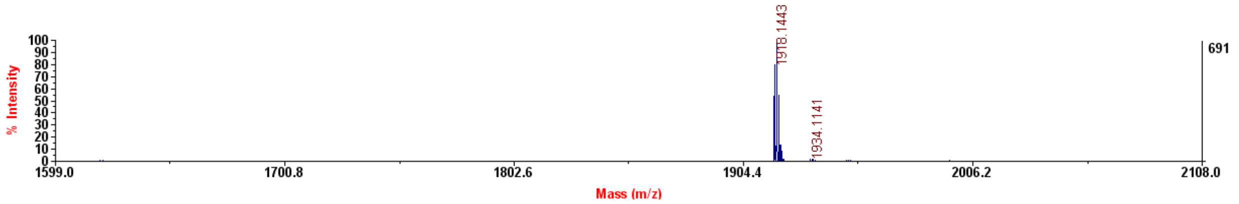

plate 96/line H/column 1

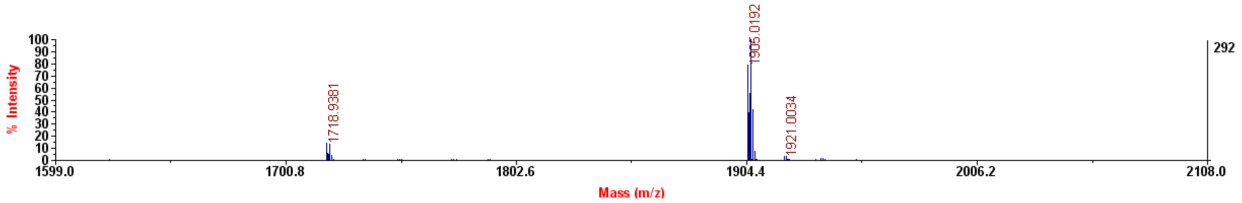

plate 96/line C/column 9

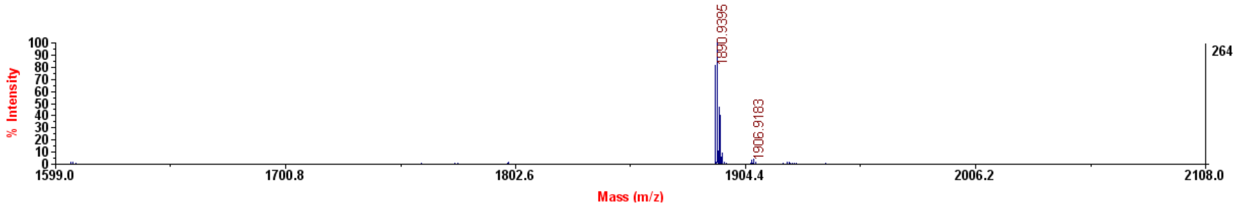

plate 96/line H/column 9

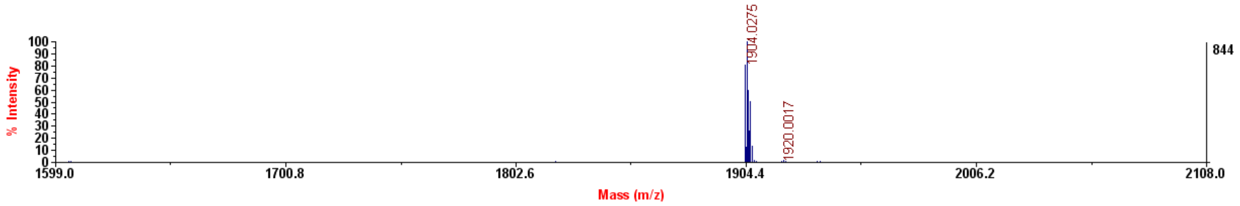

plate 96/line A/column 11

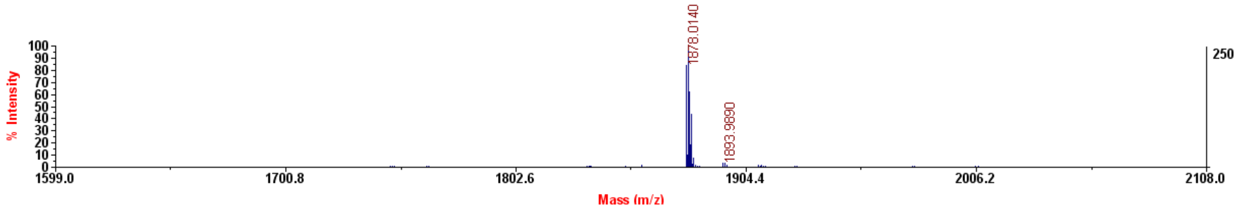

plate 97/line B/column 3

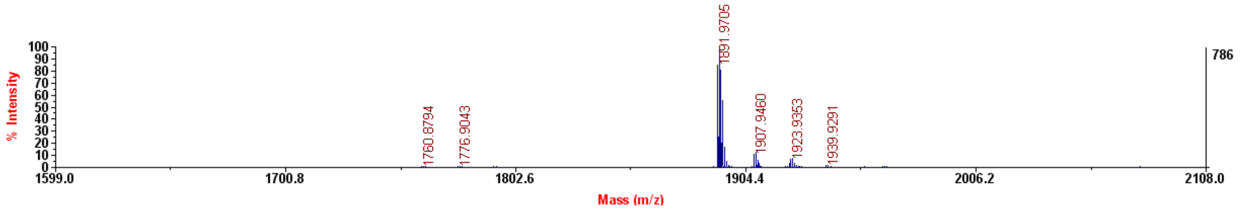

Supplementary Figure 140. MS spectra of plates 96 and 97. The data of E1, H1, C9, H9, and A11 in plate 96 and B3 in plate 97 are shown.

plate 97/line D/column 5

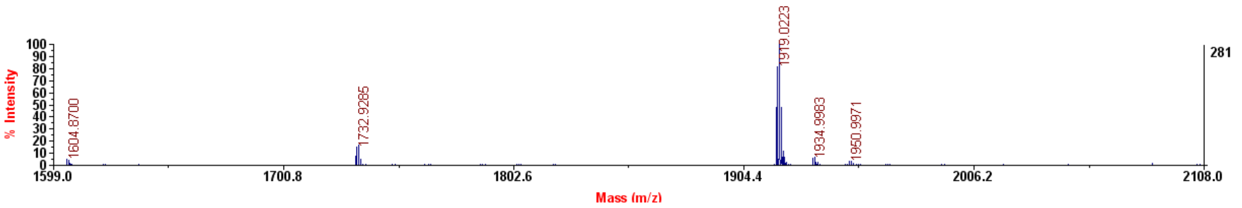

plate 97/line G/column 7

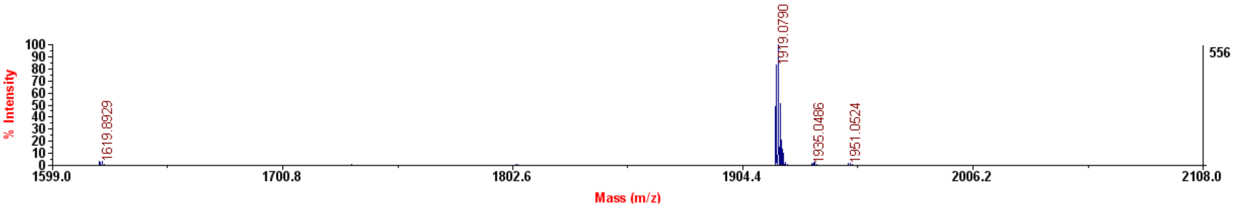

plate 97/line A/column 9

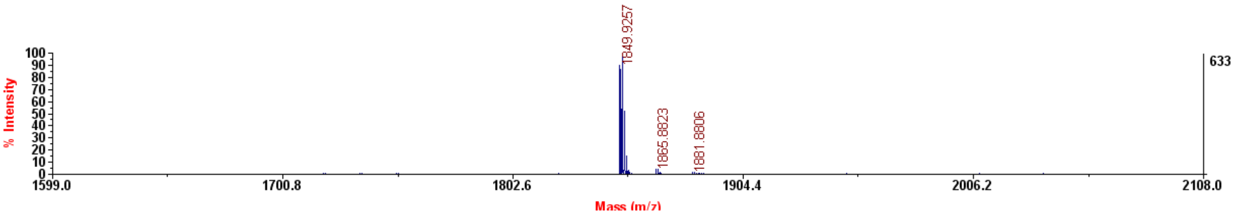

plate 97/line B/column 10

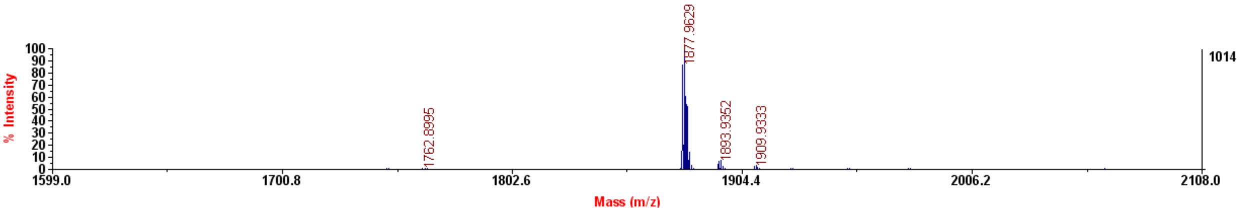

plate 97/line G/column 11

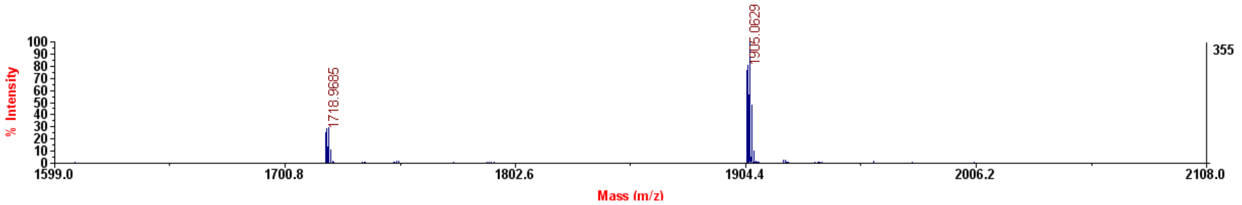

plate 98/line D/column 2

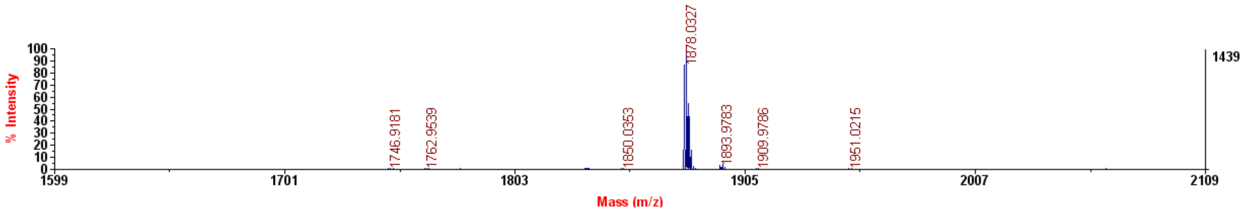

Supplementary Figure 141. MS spectra of plates 97 and 98. The data of D5, G7, A9, B10, and G11 in plate 97 and D2 in plate 98 are shown.

plate 98/line G/column 2

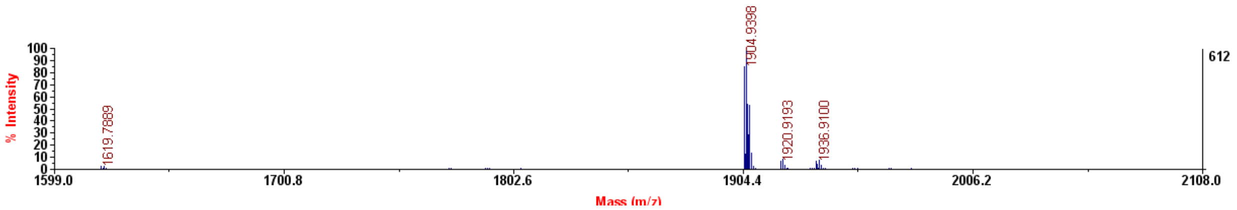

plate 98/line D/column 4

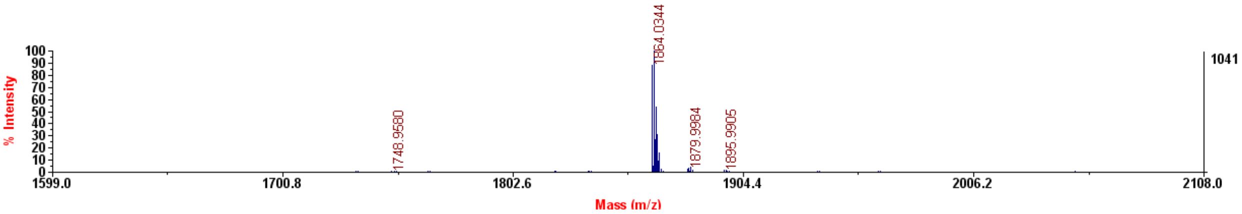

plate 98/line H/column 4

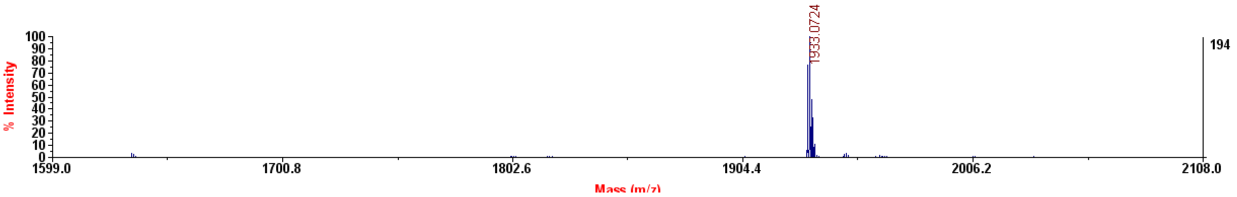

plate 98/line D/column 5

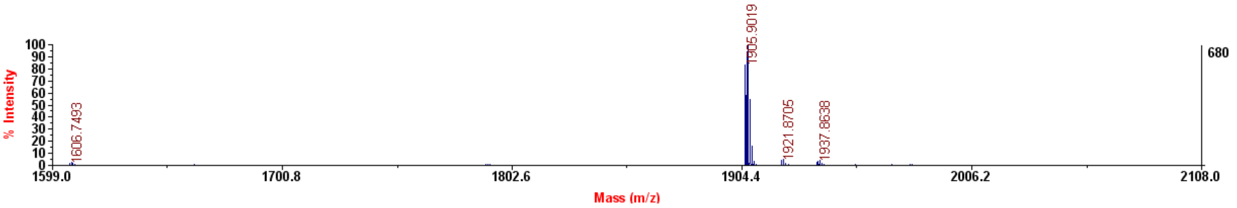

plate 98/line A/column 9

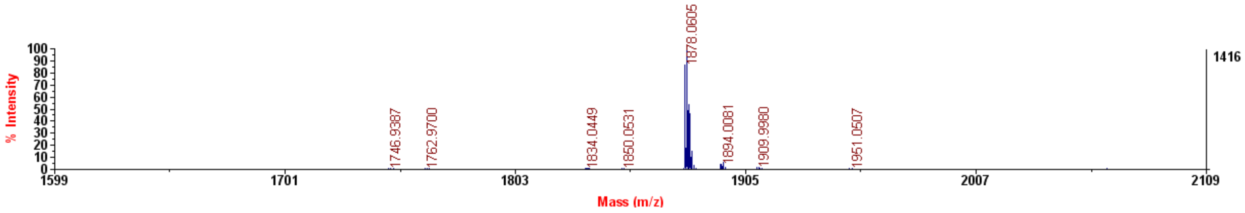

plate 98/line C/column 9

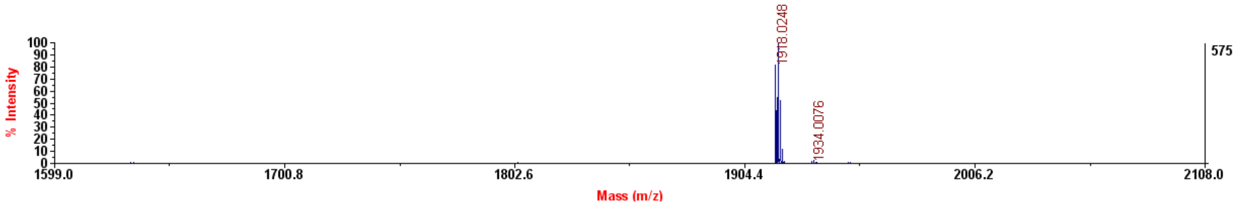

Supplementary Figure 142. MS spectra of plate 98. The data of G2, D4, H4, D5, A9, and C9 in plate 98 are shown.

plate 99/line F/column 1

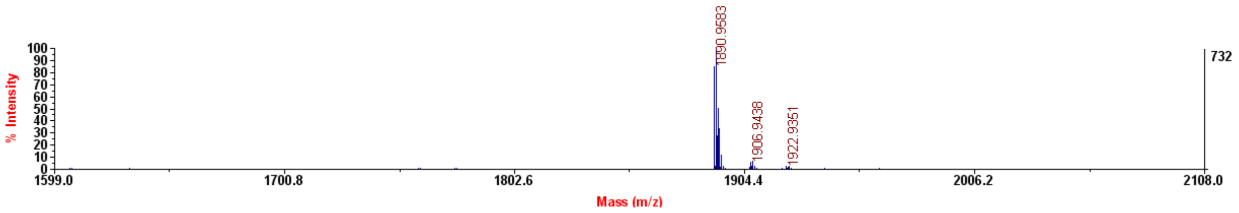

plate 99/line G/column 4

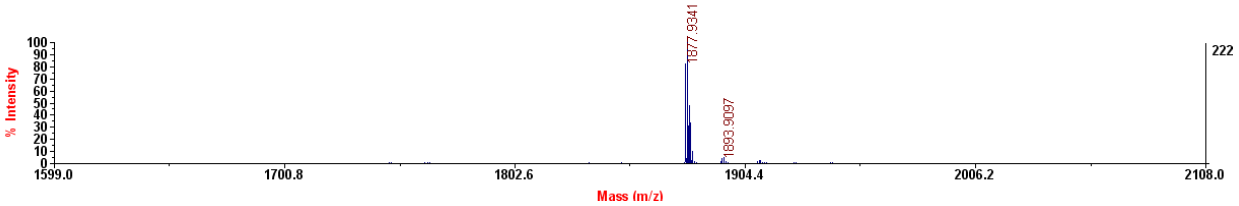

plate 99/line A/column 6

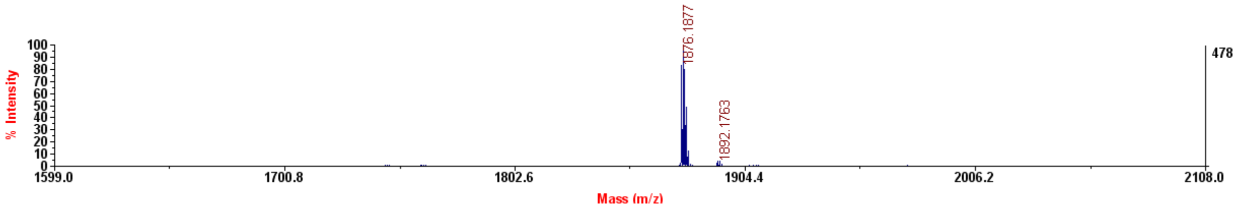

plate 99/line B/column 9

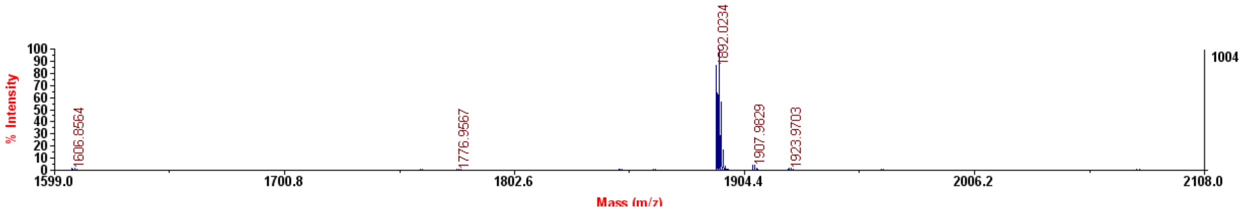

plate 99/line C/column 10

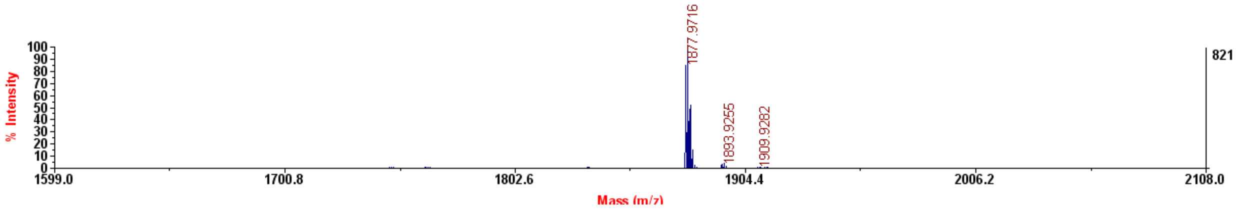

plate 99/line D/column 10

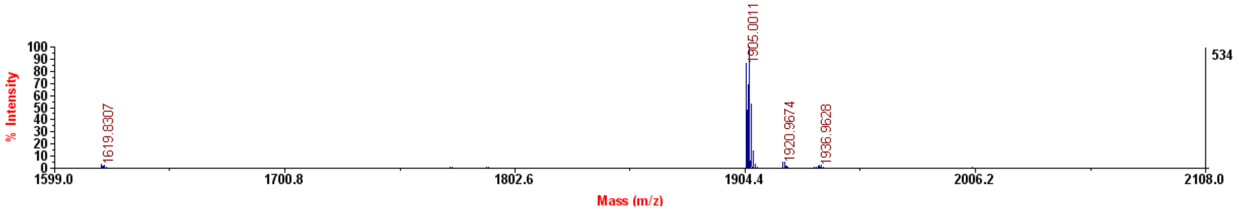

Supplementary Figure 143. MS spectra of plate 99. The data of F1, G4, A6, B9, C10, and D10 in plate 99 are shown.

plate 100/line E/column 1

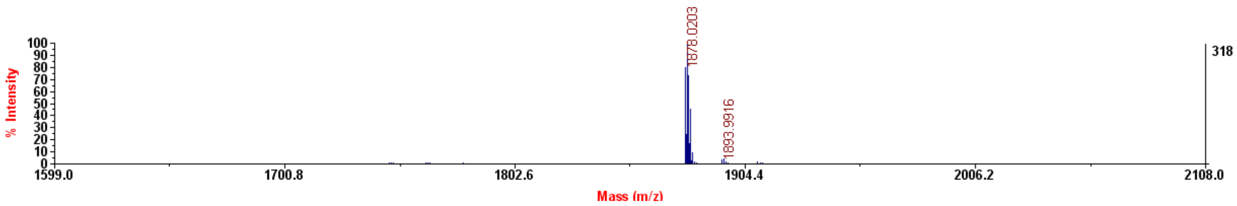

plate 100/line C/column 2

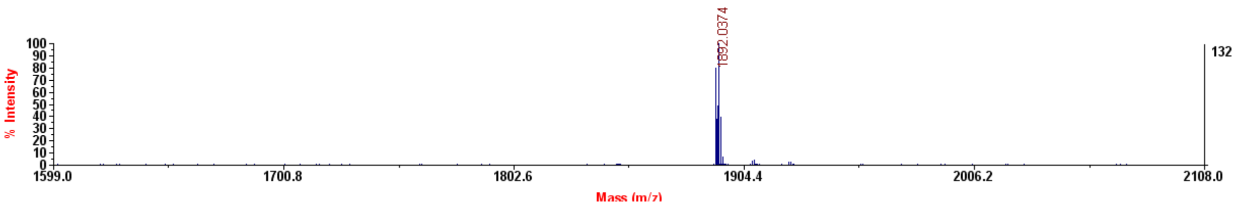

plate 100/line C/column 5

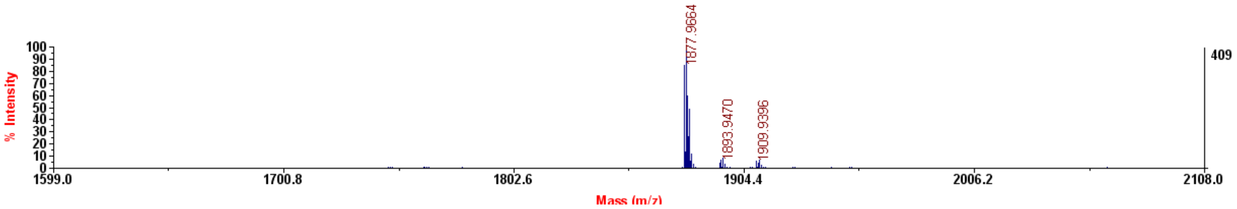

plate 100/line D/column 7

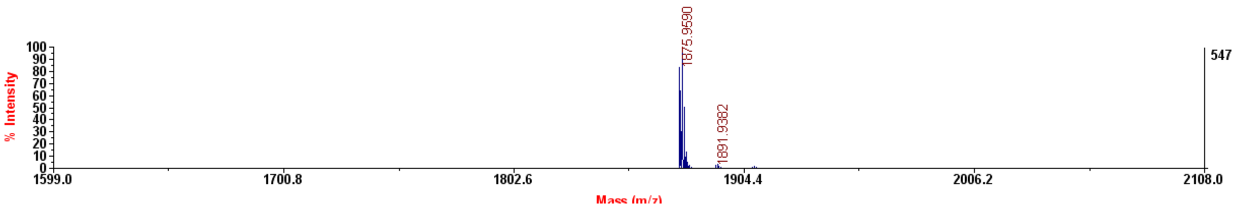

plate 100/line C/column 11

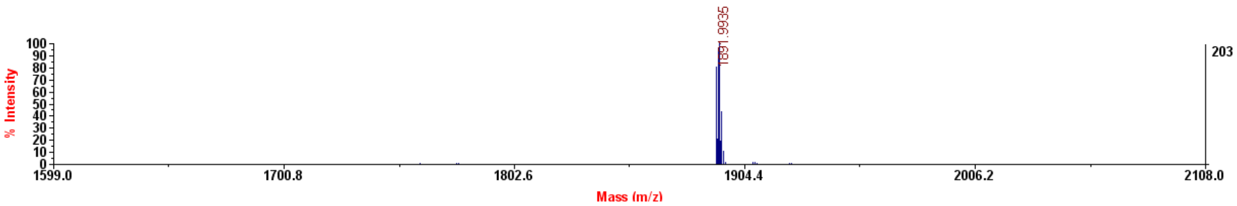

plate 100/line G/column 11

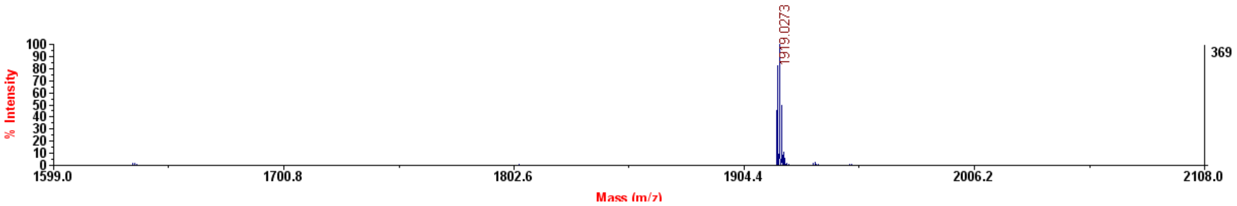

Supplementary Figure 144. MS spectra of plate 100. The data of E1, C2, C5, D7, C11, and G11 in plate 100 are shown.

plate 101/line B/column 4

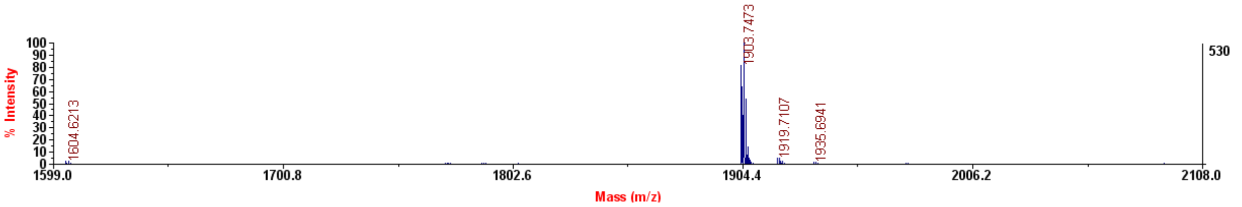

plate 101/line B/column 10

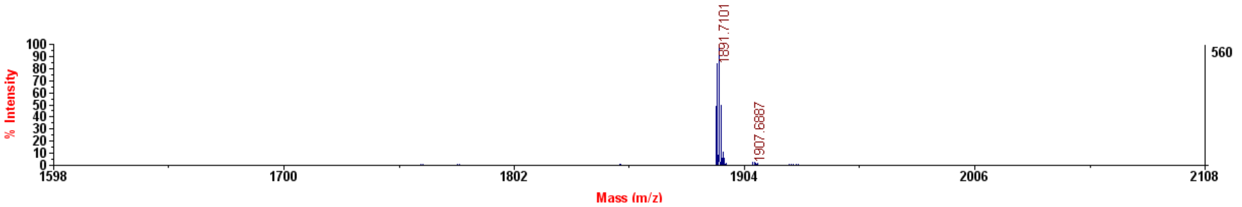

plate 101/line E/column 10

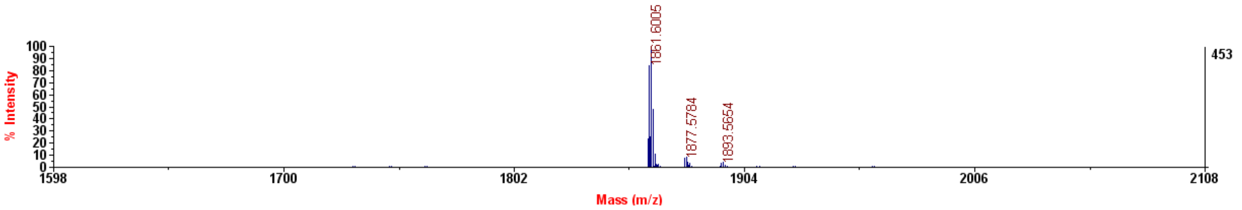

plate 102/line A/column 7

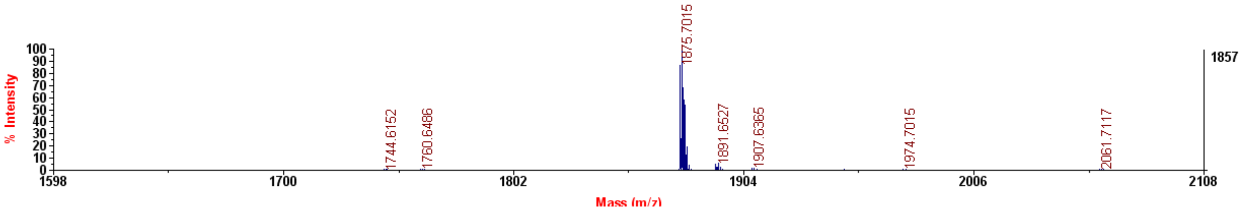

plate 102/line F/column 8

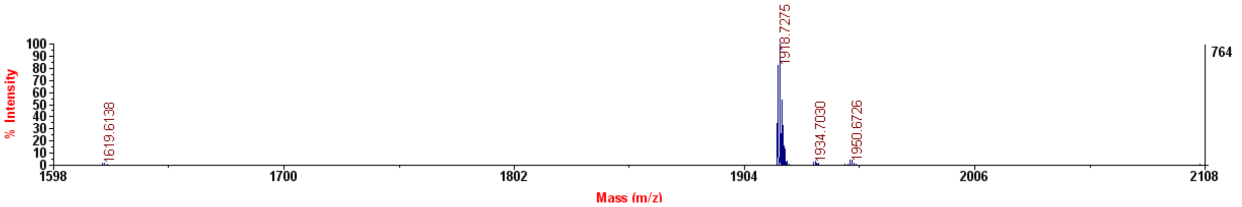

plate 102/line H/column 8

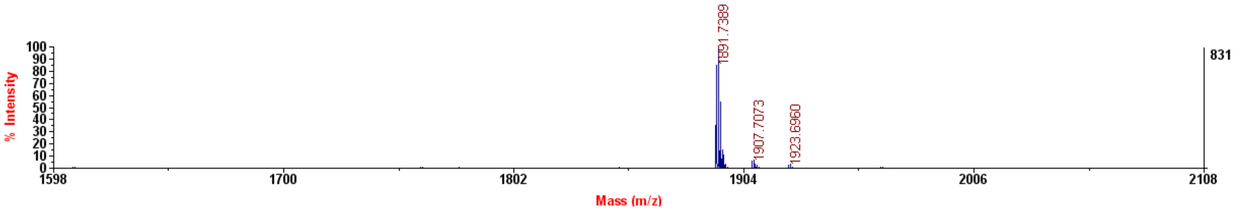

Supplementary Figure 145. MS spectra of plates 101 and 102. The data of B4, B10, and E10 in plate 101 and A7, F8, and H8 in plate 102 are shown.

plate 102/line H/column 9

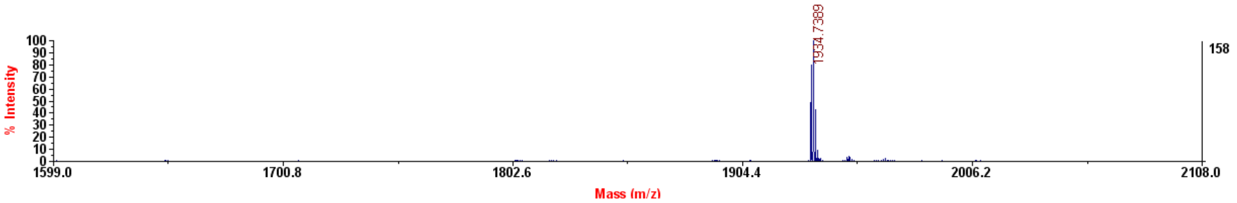

plate 102/line G/column 10

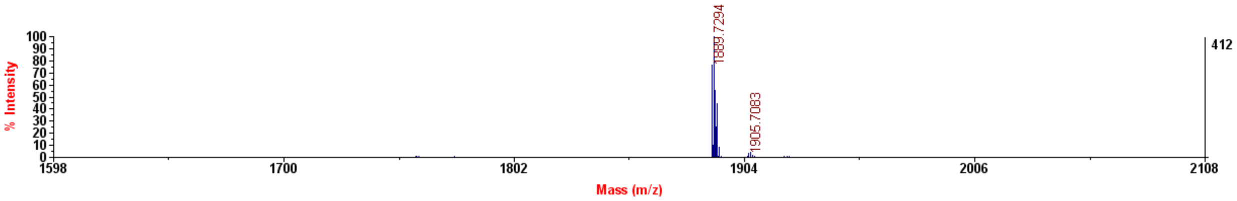

plate 102/line H/column 10

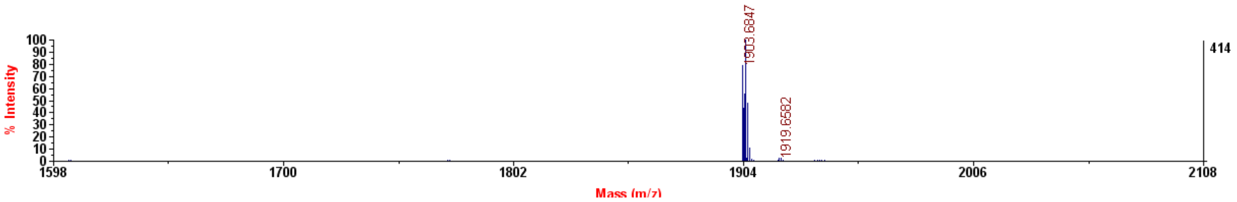

plate 103/line F/column 3

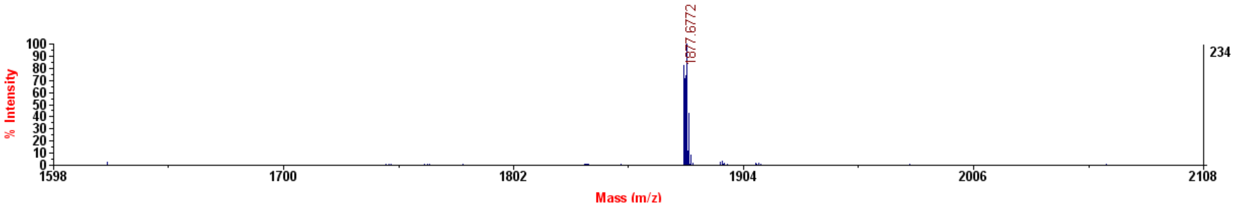

plate 103/line D/column 4

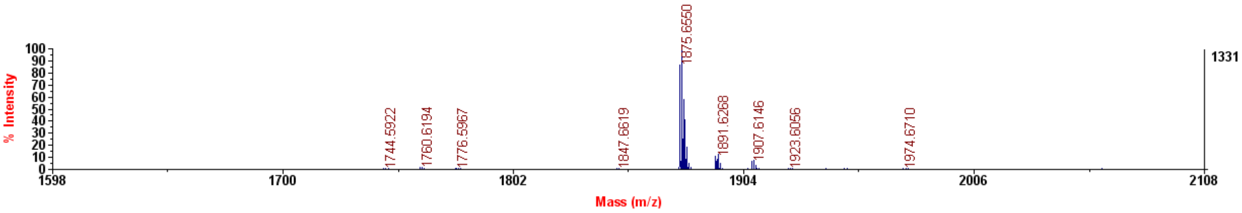

plate 103/line G/column 6

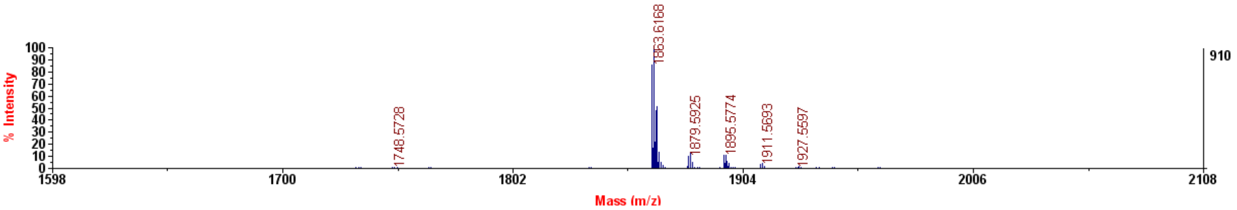

Supplementary Figure 146. MS spectra of plates 102 and 103. The data of H9, G10, and H10 in plate 102 and F3, D4, and G6 in plate 103 are shown.

plate 103/line B/column 7

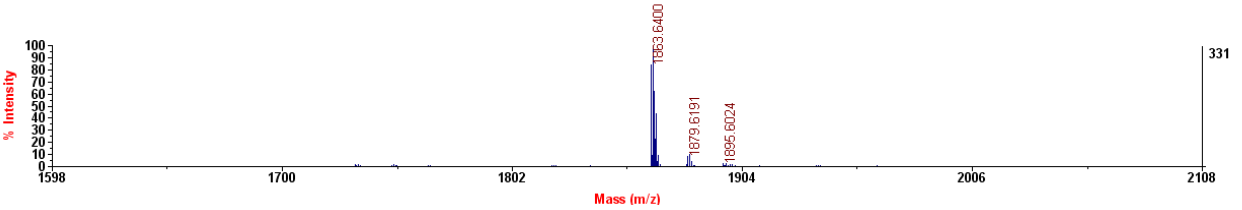

plate 103/line C/column 9

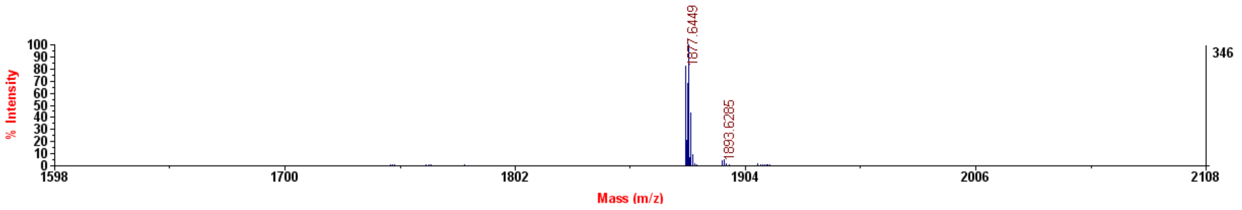

plate 104/line B/column 1

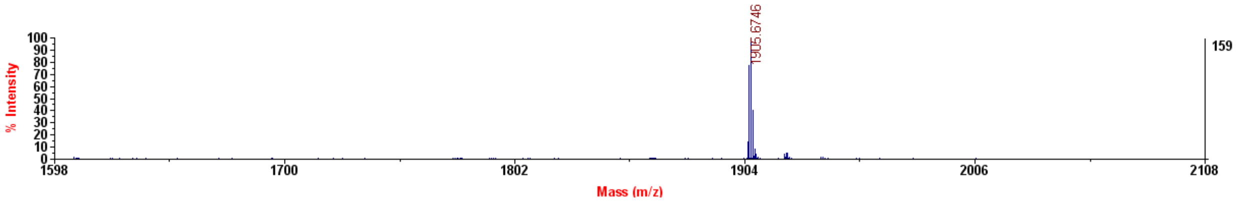

plate 104/line H/column 2

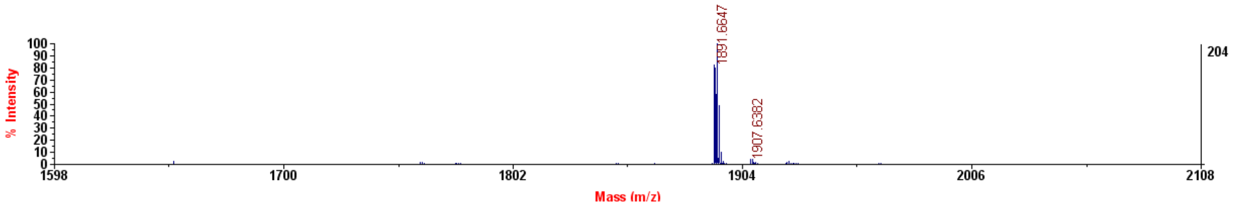

plate 104/line E/column 4

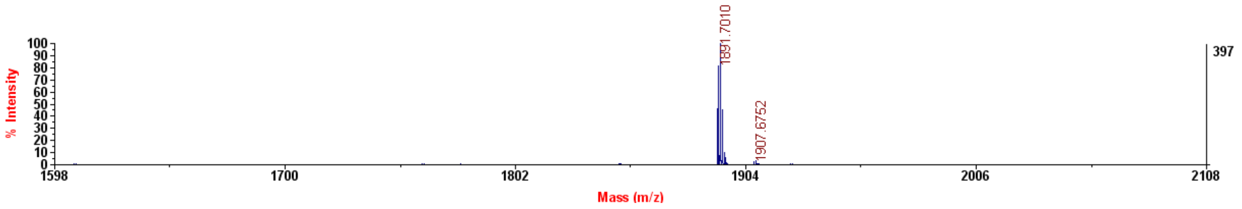

plate 104/line A/column 7

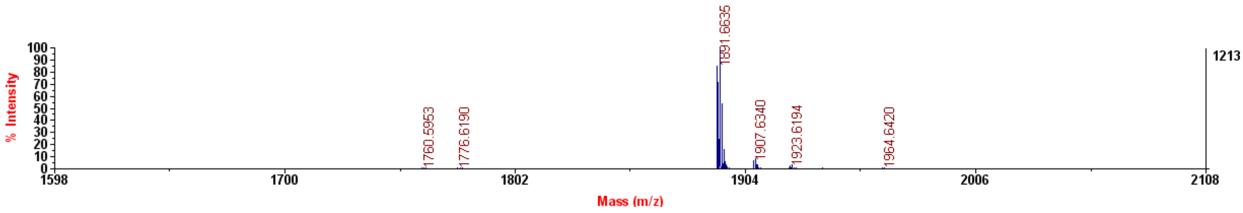

Supplementary Figure 147. MS spectra of plates 103 and 104. The data of B7 and C9 in plate 103 and B1, H2, E4, and A7 in plate 104 are shown.

plate 104/line A/column 11

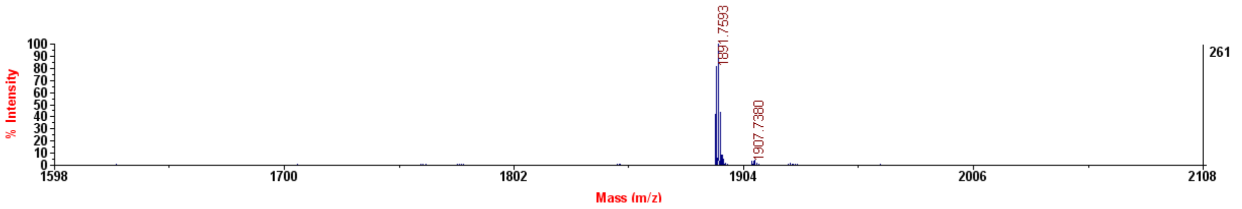

plate 105/line H/column 1

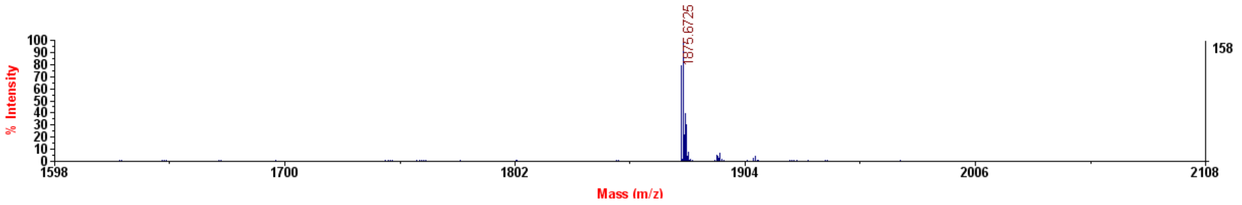

plate 105/line G/column 5

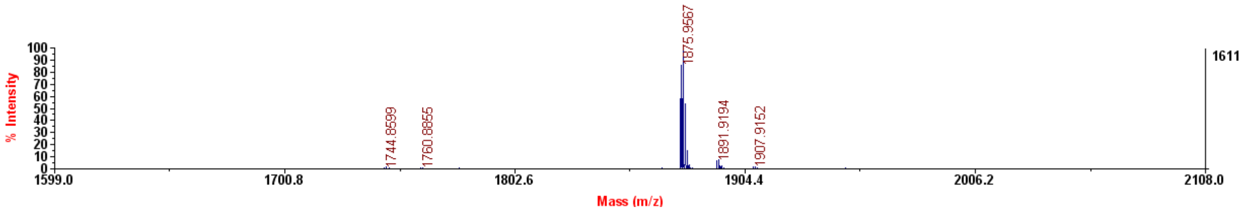

plate 105/line E/column 6

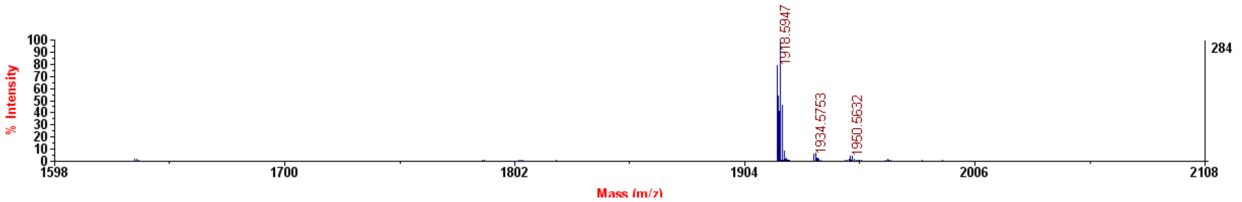

plate 105/line G/column 8

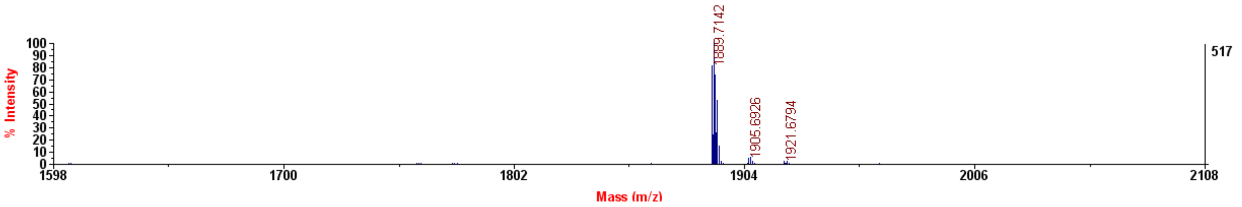

plate 106/line E/column 3

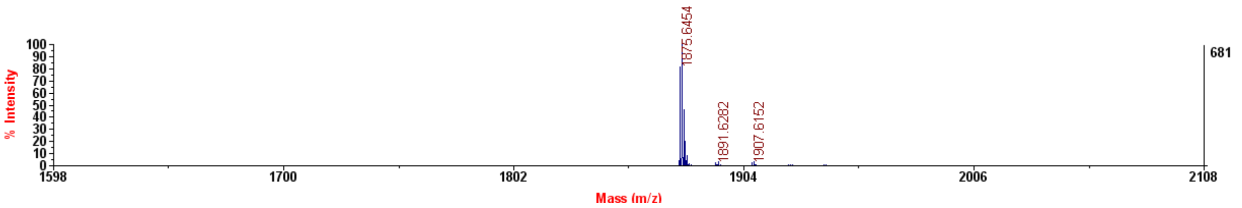

Supplementary Figure 148. MS spectra of plates 104, 105, and 106. The data of A11 in plate 104, H1, G5, E6, and G8 in plate 105, and E3 in plate 106 are shown.

plate 106/line H/column 8

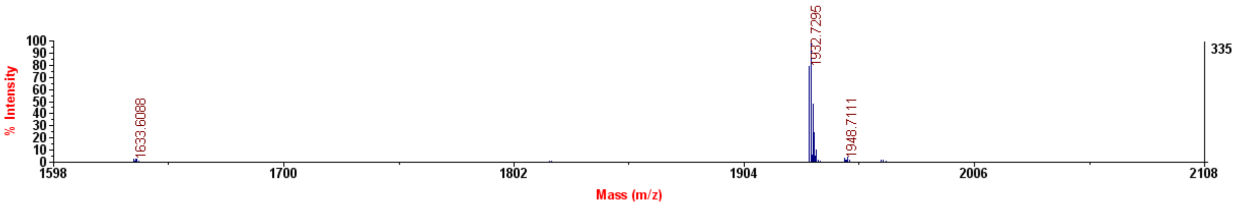

plate 106/line H/column 10

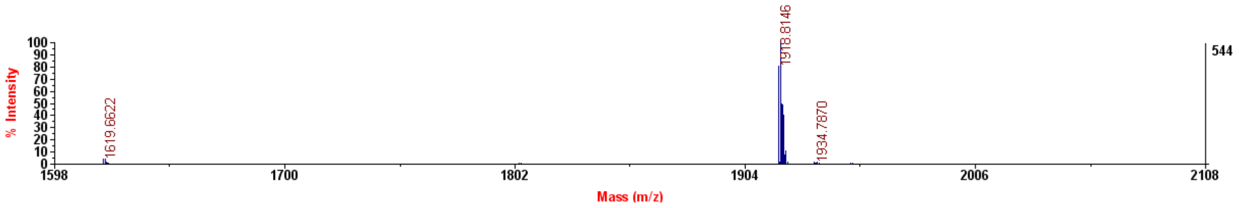

plate 106/line B/column 11

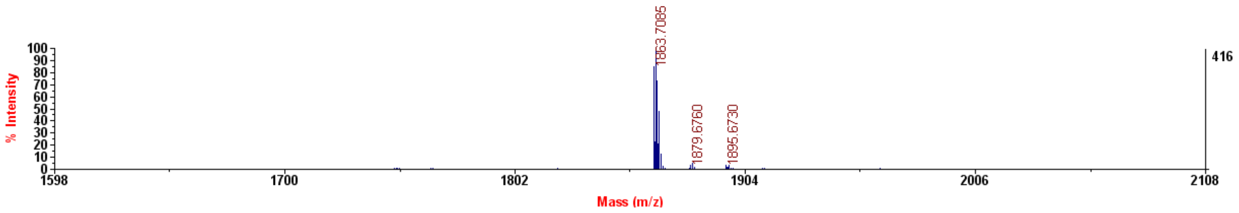

plate 108/line A/column 4

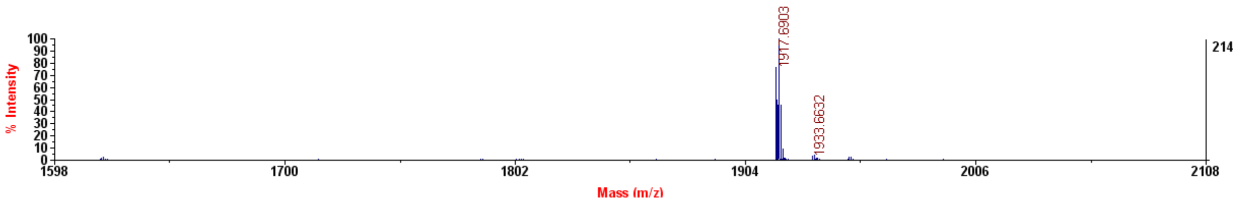

plate 108/line G/column 5

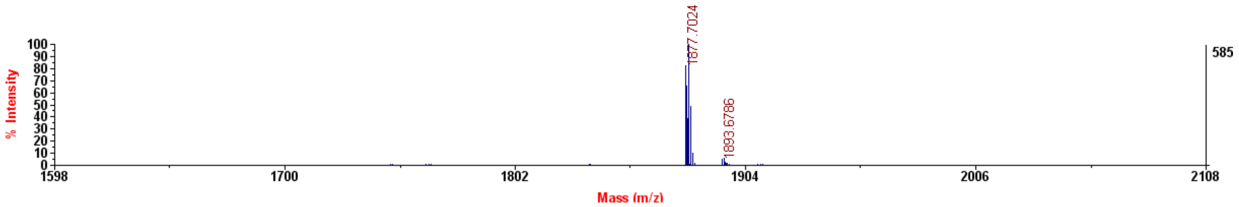

plate 108/line A/column 10

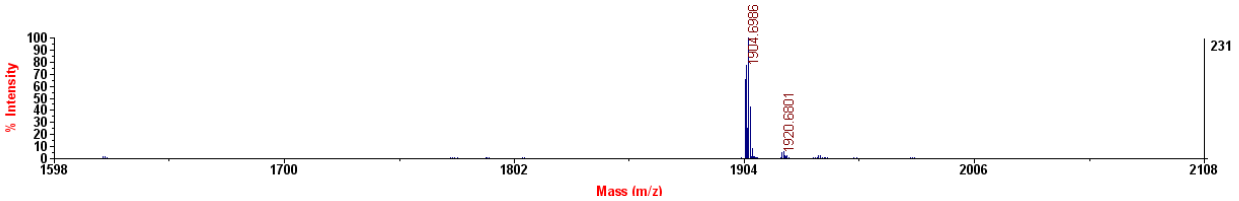

Supplementary Figure 149. MS spectra of plates 106 and 108. The data of H8, H10, and B11 in plate 106 and A4, G5, and A10 in plate 108 are shown.

plate 108/line G/column 11

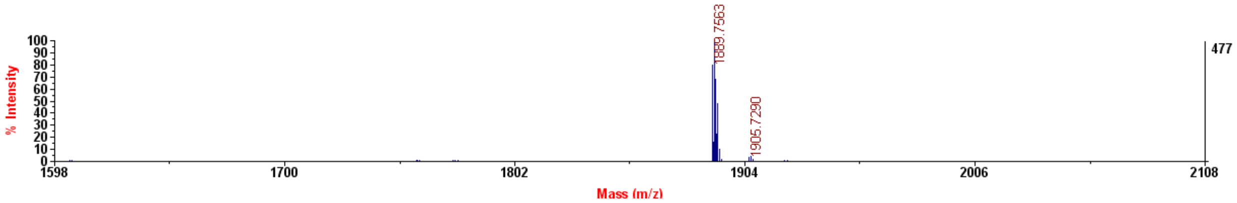

plate 109/line C/column 5

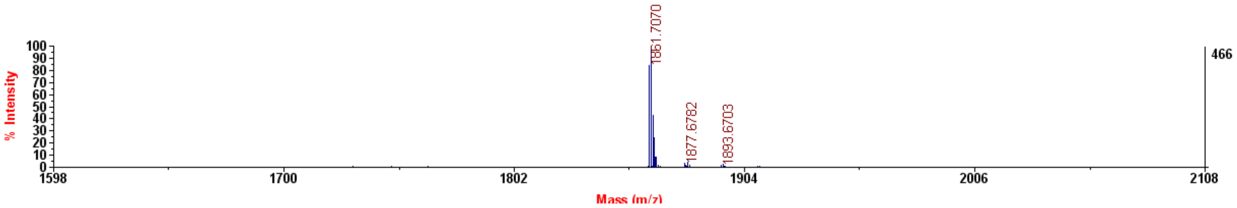

plate 109/line G/column 9

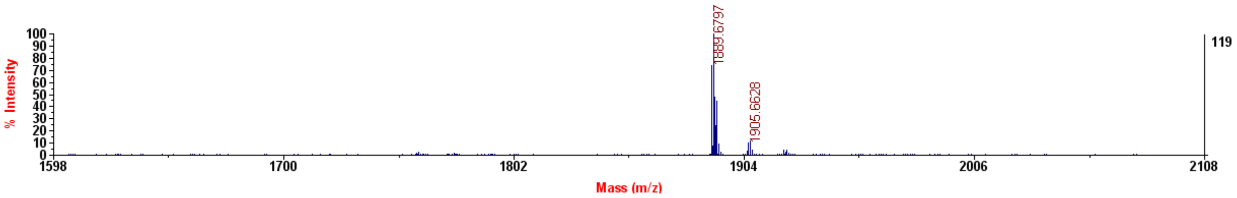

plate 109/line A/column 10

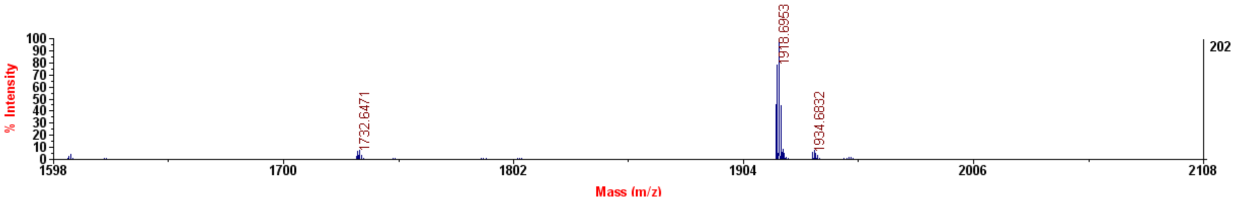

plate 109/line B/column 10

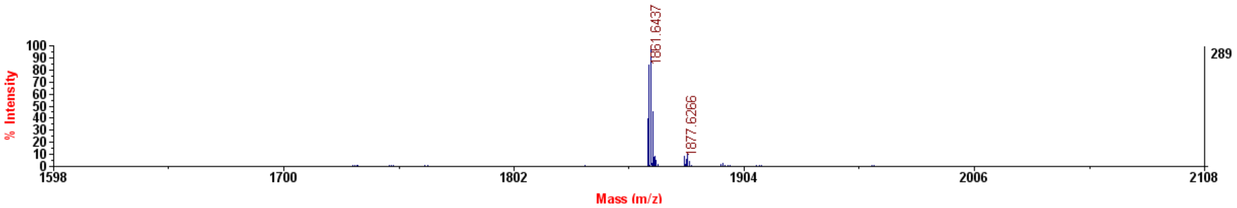

plate 110/line D/column 1

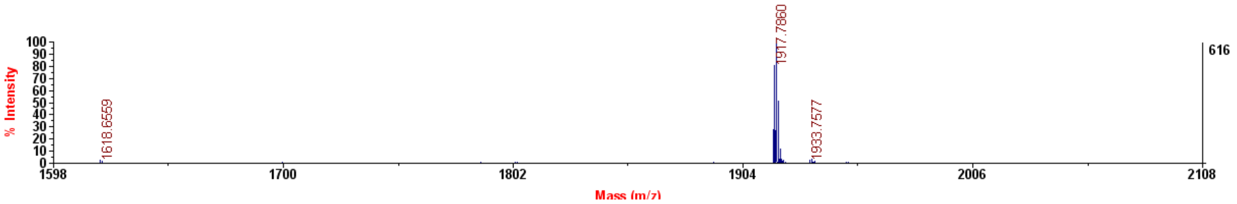

Supplementary Figure 150. MS spectra of plates 108, 109, and 110. The data of G11 in plate 108, C5, G9, A10, and B10 in plate 109, and D1 in plate 110 are shown.

plate 110/line A/column 2

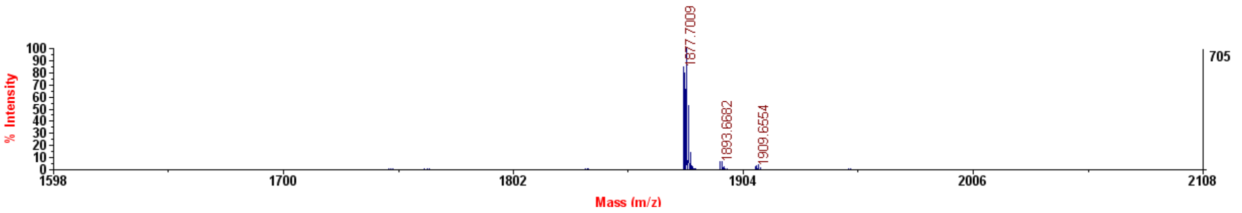

plate 110/line D/column 7

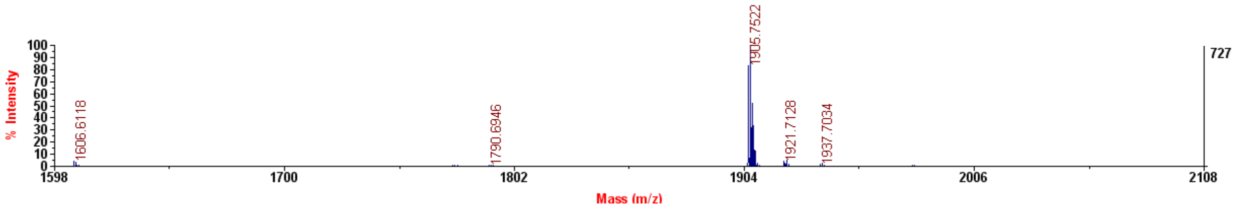

plate 111/line G/column 2

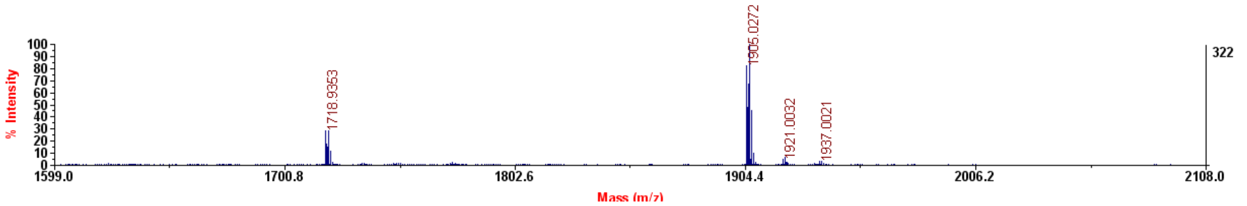

plate 111/line G/column 3

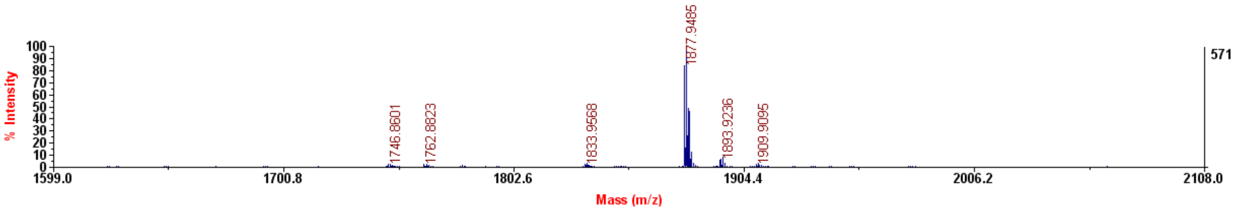

plate 111/line B/column 4

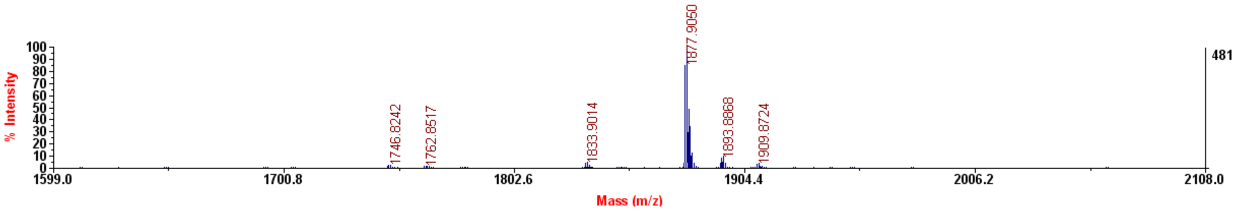

plate 111/line F/column 4

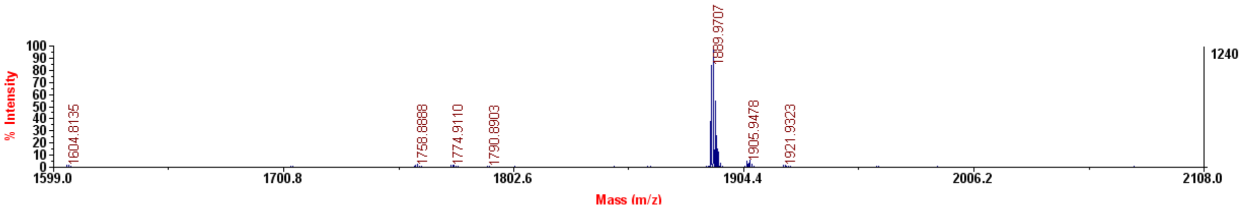

Supplementary Figure 151. MS spectra of plates 110 and 111. The data of A2 and D7 in plate 110 and G2, G3, B4, and F4 in plate 111 are shown.

plate 111/line G/column 11

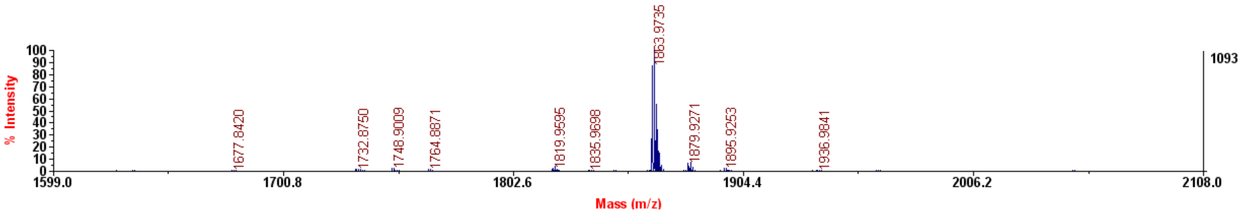

plate 112/line G/column 3

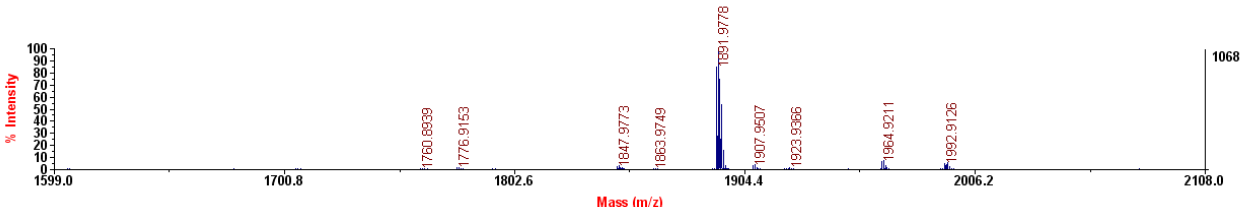

plate 112/line H/column 4

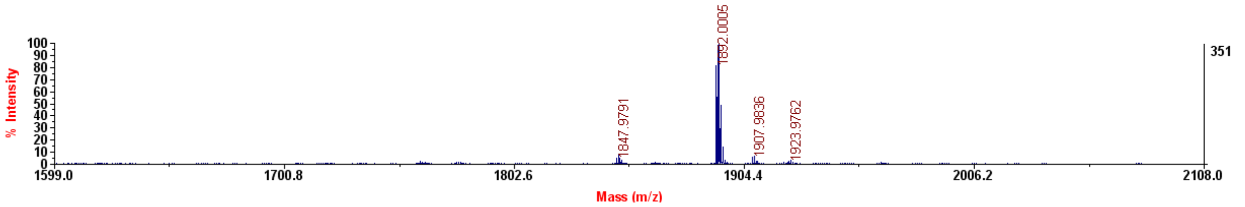

plate 113/line A/column 2

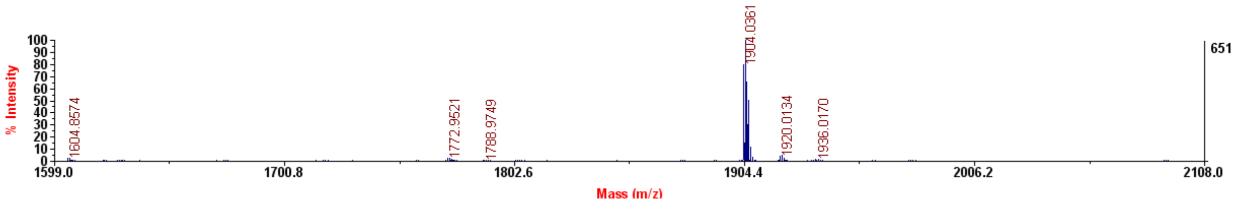

plate 113/line F/column 3

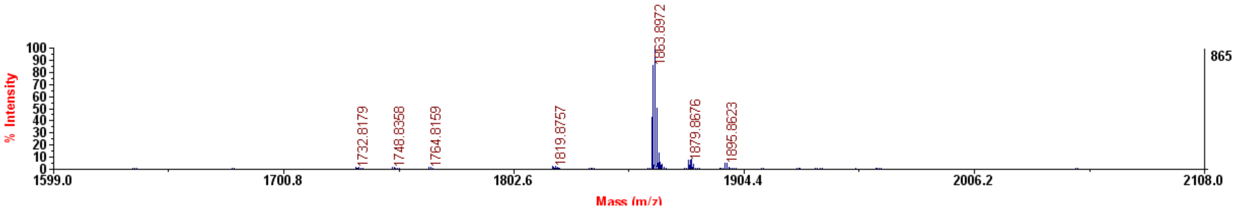

plate 114/line H/column 2

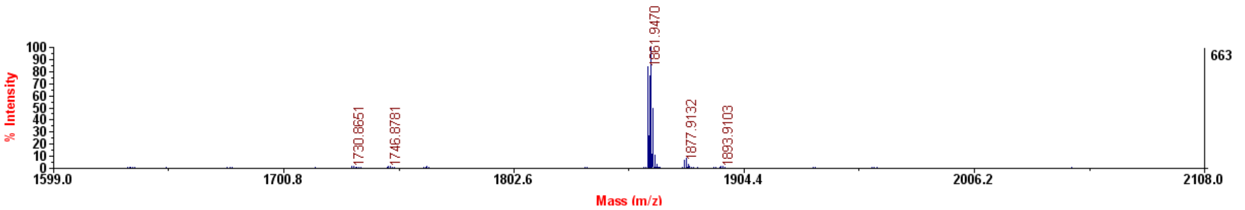

Supplementary Figure 152. MS spectra of plates 111, 112, 113, and 114. The data of G11 in plate 111, G3 and H4 in plate 112, A2 and F3 in plate 113, and H2 in plate 114 are shown.

plate 114/line B/column 4

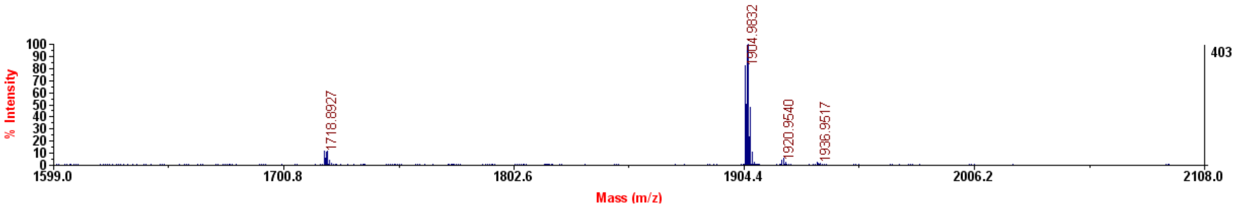

plate 114/line H/column 4

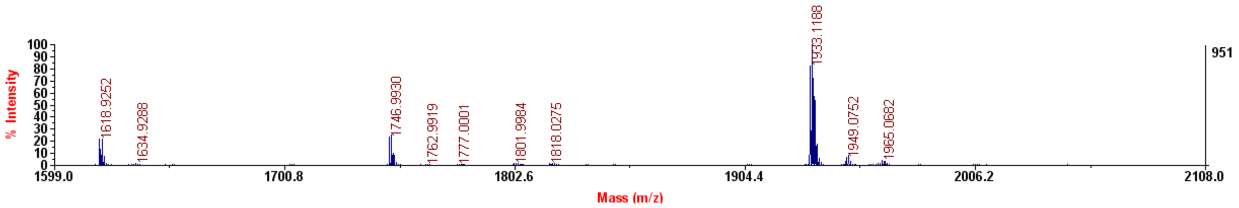

plate 114/line B/column 9

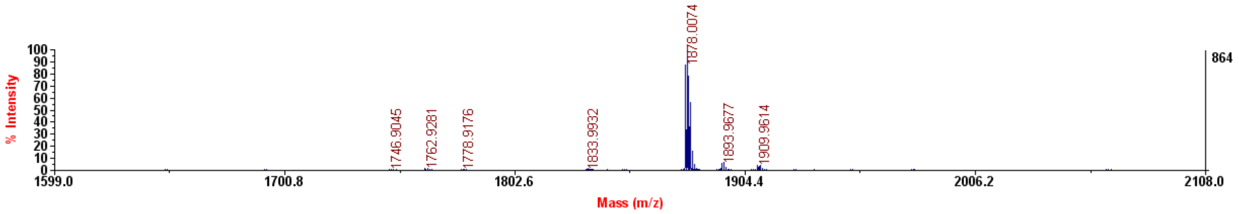

plate 114/line C/column 9

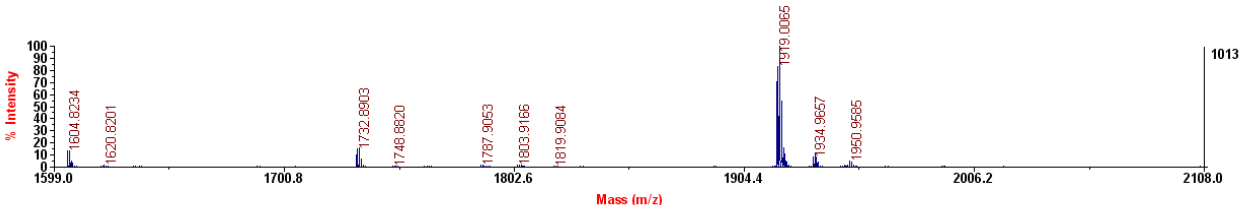

plate 117/line E/column 3

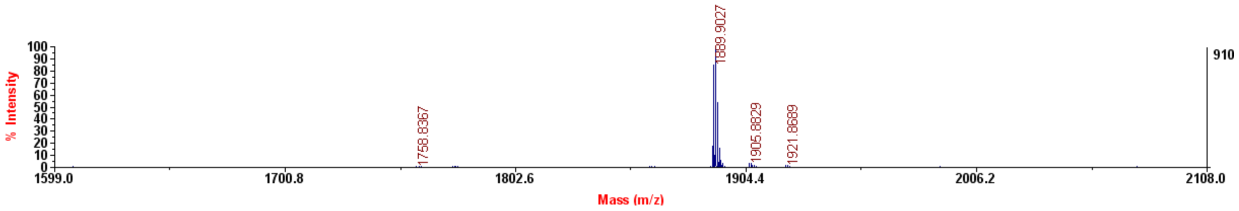

plate 117/line D/column 5

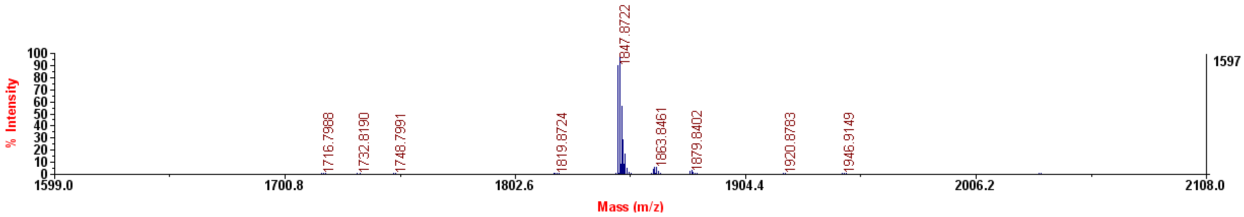

Supplementary Figure 153. MS spectra of plates 114 and 117. The data of B4, H4, B9, and C9 in plate 114 and E3 and D5 in plate 117 are shown.

plate 117/line A/column 10

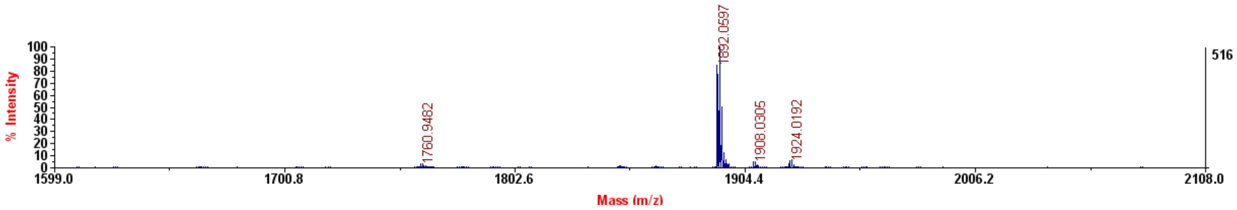

plate 118/line B/column 2

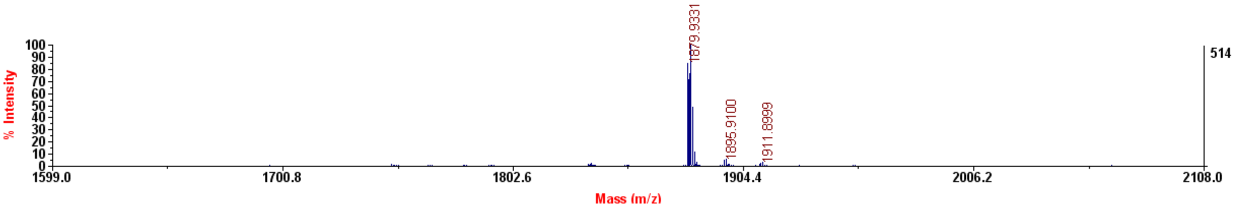

plate 118/line D/column 2

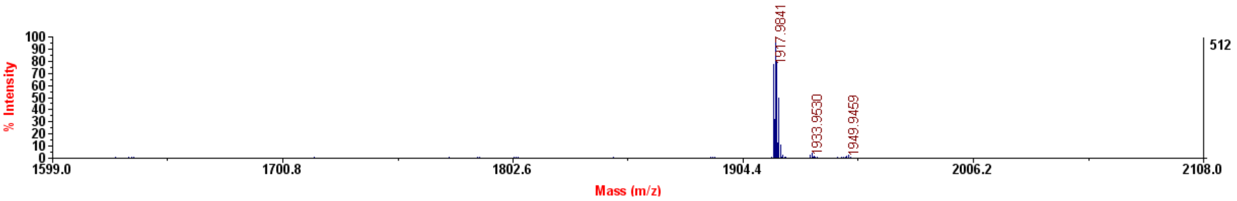

plate 118/line A/column 4

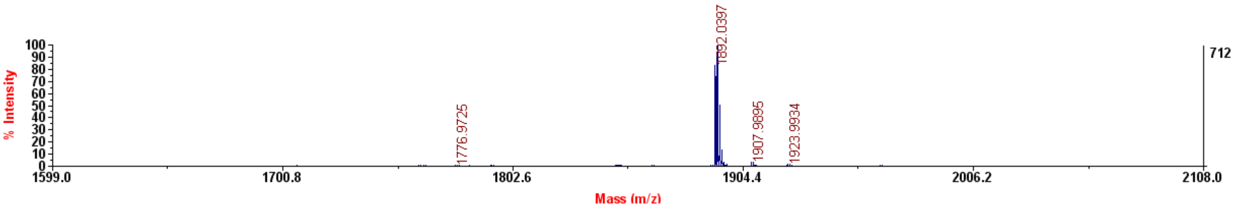

plate 118/line G/column 5

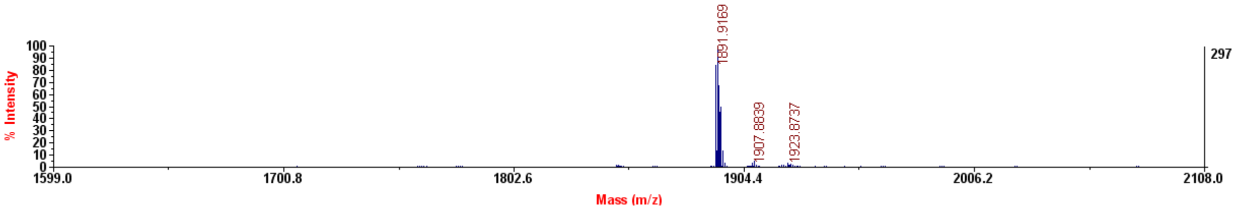

plate 118/line C/column 7

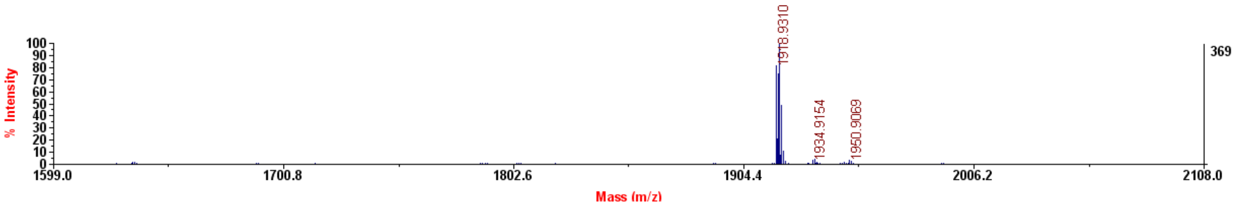

Supplementary Figure 154. MS spectra of plates 117 and 118. The data of A10 in plate 117 and B2, D2, A4, G5, and C7 in plate 118 are shown.

plate 118/line H/column 9

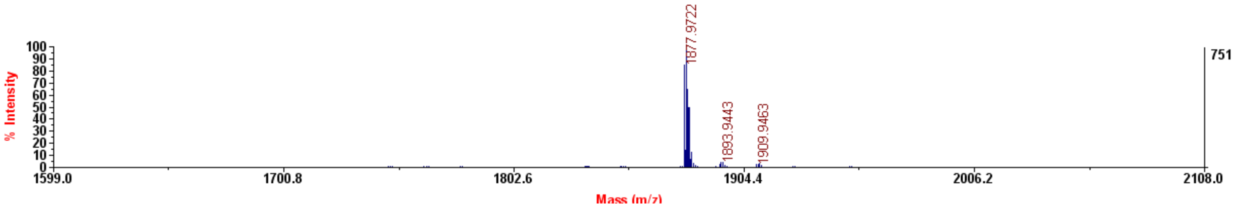

plate 118/line G/column 11

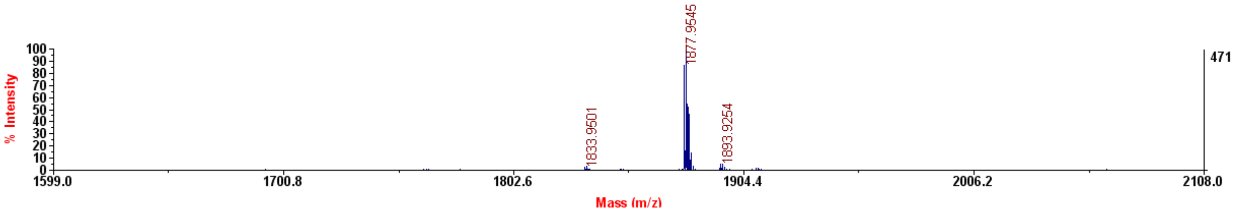

plate 119/line B/column 4

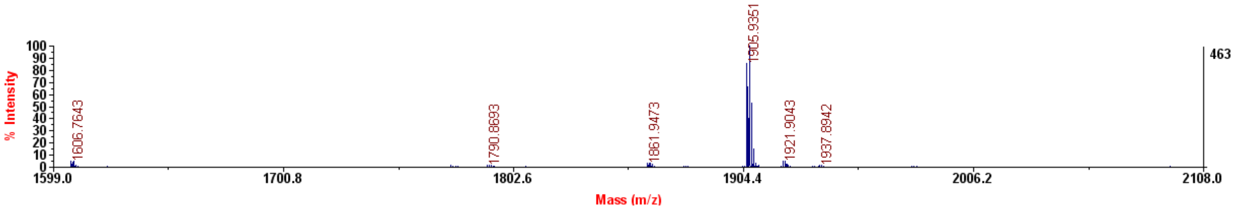

plate 119/line H/column 5

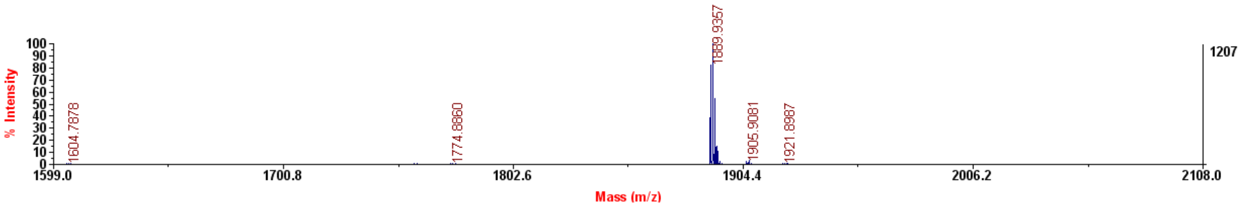

plate 119/line A/column 11

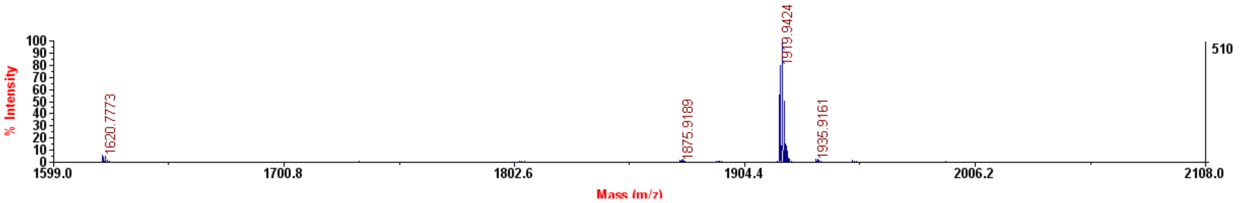

plate 119/line C/column 11

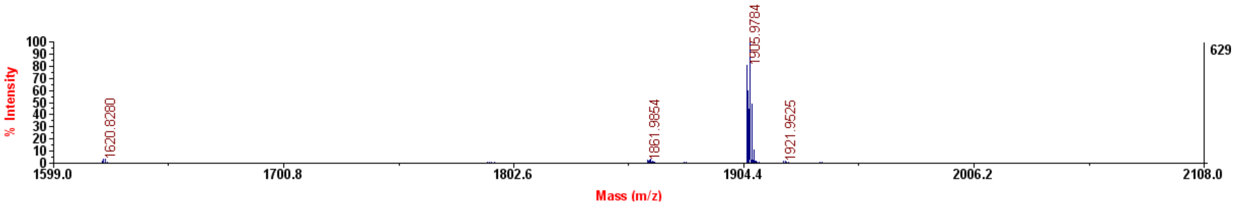

Supplementary Figure 155. MS spectra of plates 118 and 119. The data of H9 and G11 in plate 118 and B4, H5, A11, and C11 in plate 119 are shown.

plate 119/line D/column 11

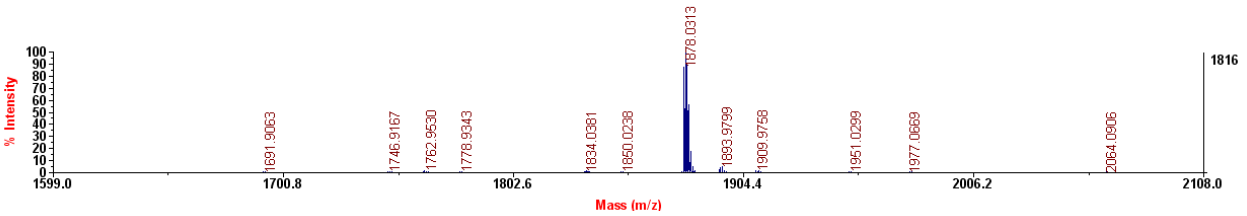

plate 120/line B/column 1

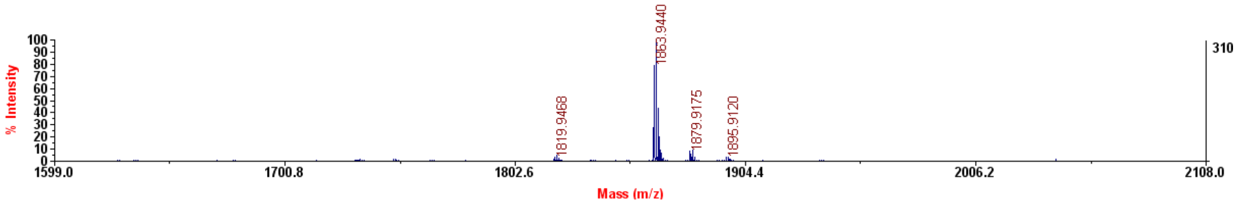

plate 120/line D/column 2

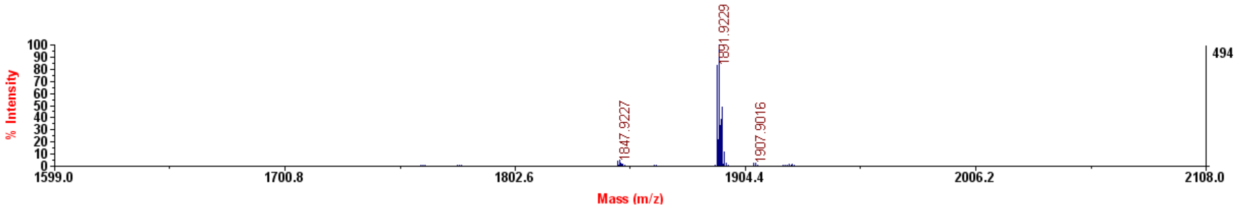

plate 120/line E/column 2

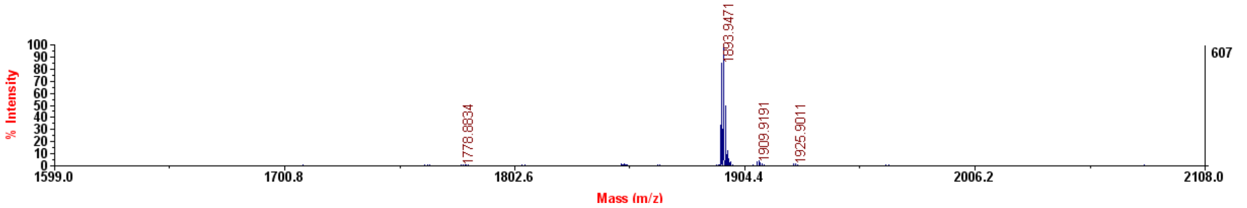

plate 120/line H/column 4

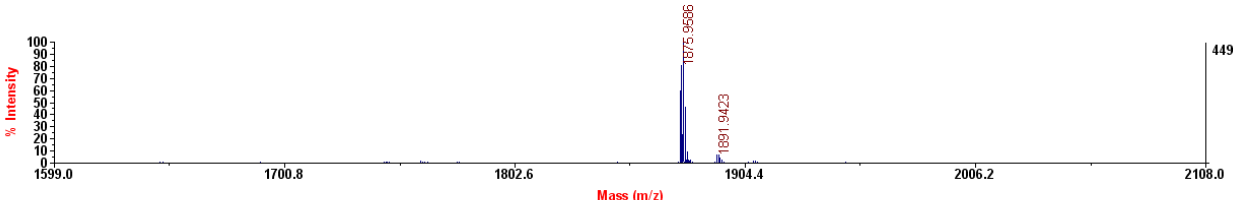

plate 120/line F/column 5

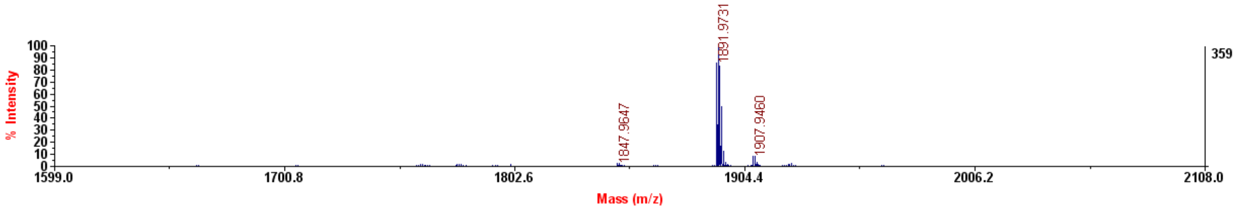

Supplementary Figure 156. MS spectra of plates 119 and 120. The data of D11 in plate 119 and B1, D2, E2, H4, and F5 in plate 120 are shown.

plate 121/line A/column 1

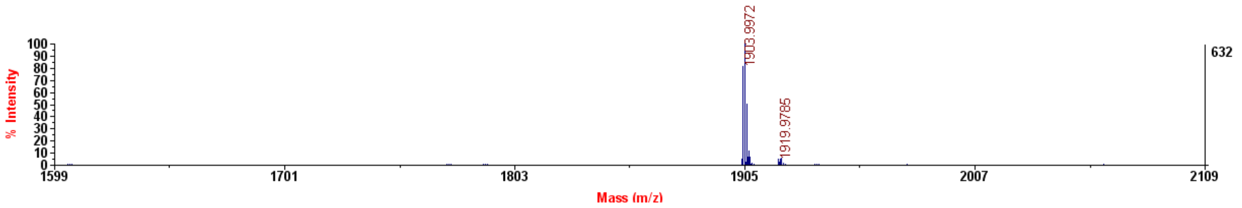

plate 121/line A/column 3

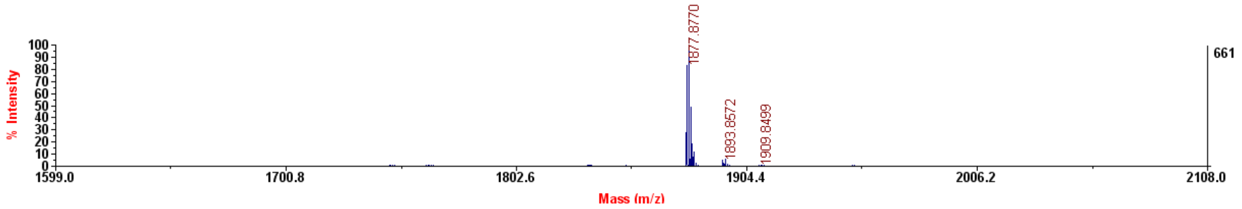

plate 121/line A/column 8

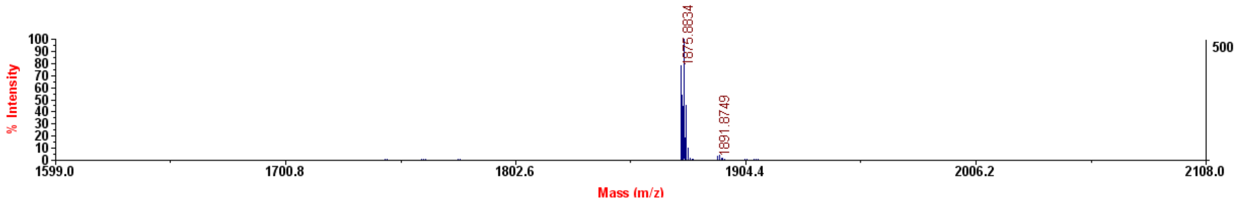

plate 121/line E/column 8

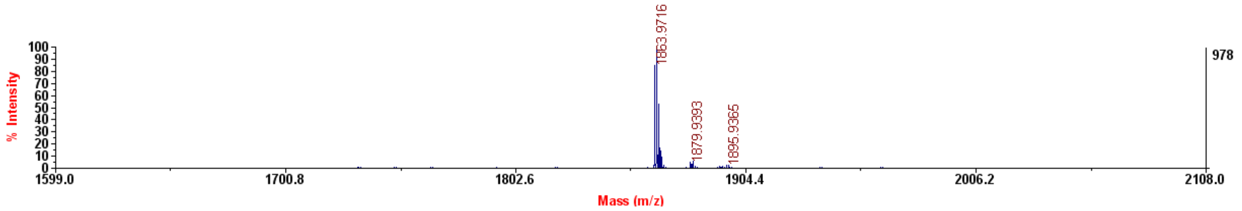

plate 121/line E/column 9

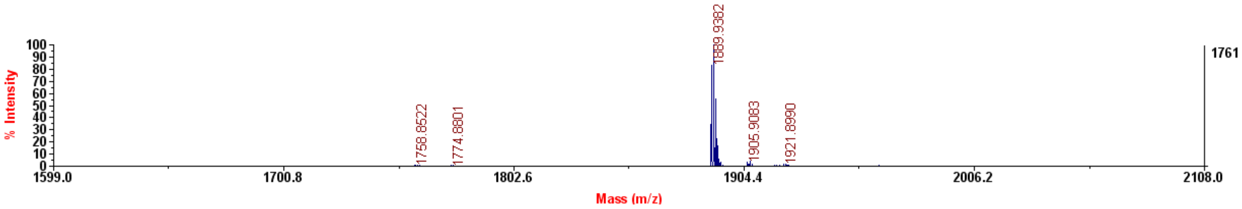

plate 121/line D/column 10

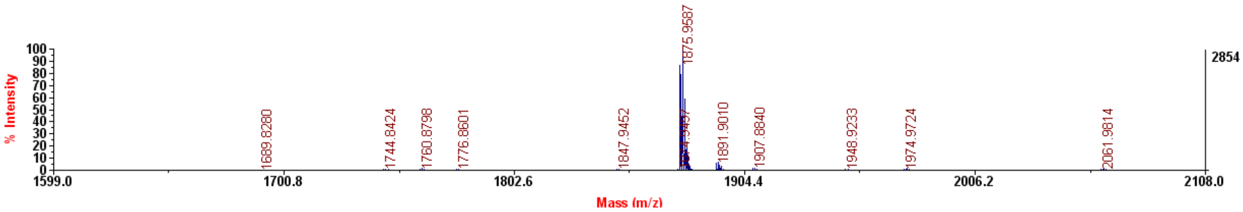

Supplementary Figure 157. MS spectra of plate 121. The data of A1, A3, A8, E8, E9, and D10 in plate 121 are shown.

plate 121/line E/column 10

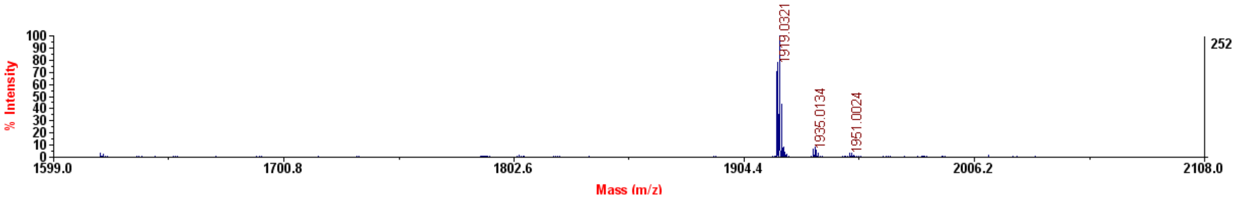

plate 121/line A/column 11

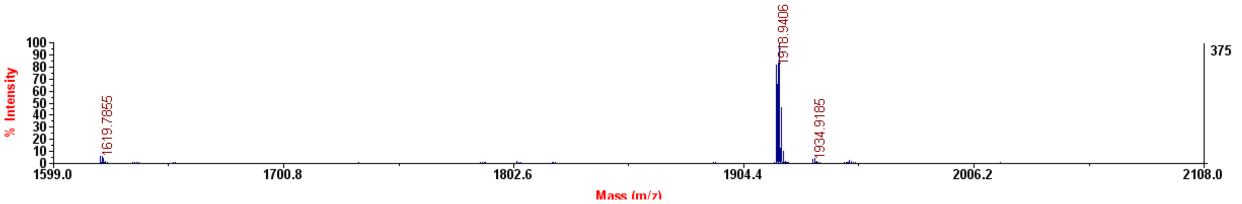

plate 121/line B/column 11

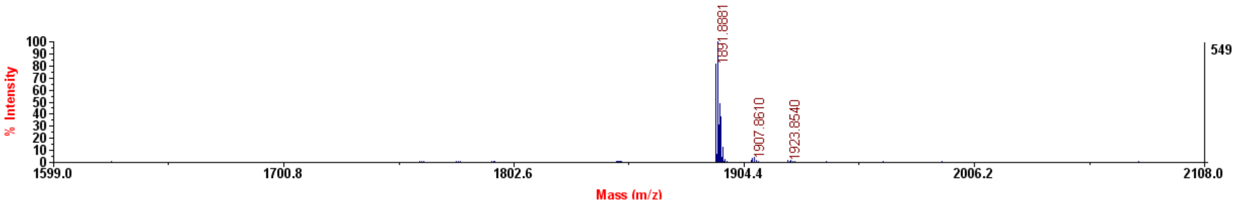

plate 121/line F/column 11

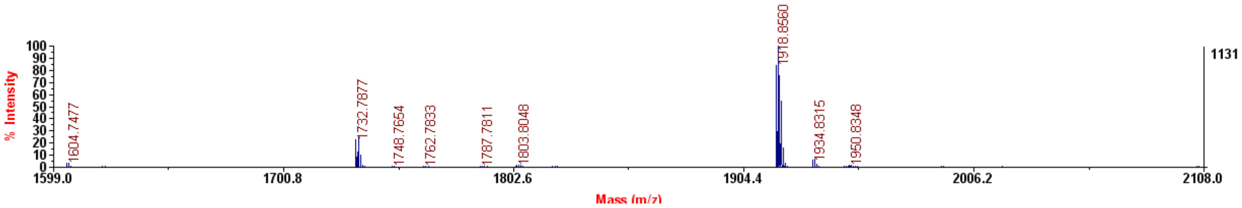

plate 122/line F/column 1

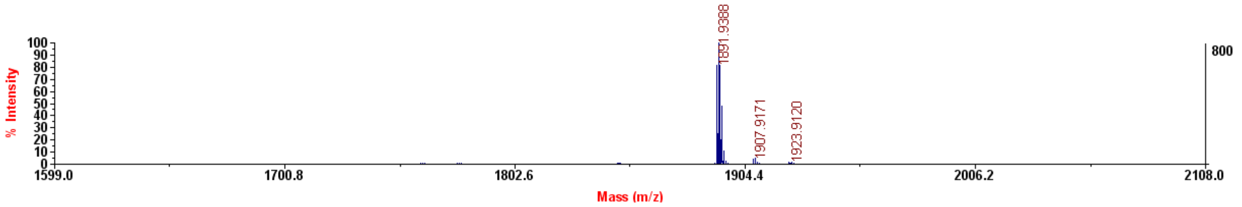

plate 122/line C/column 3

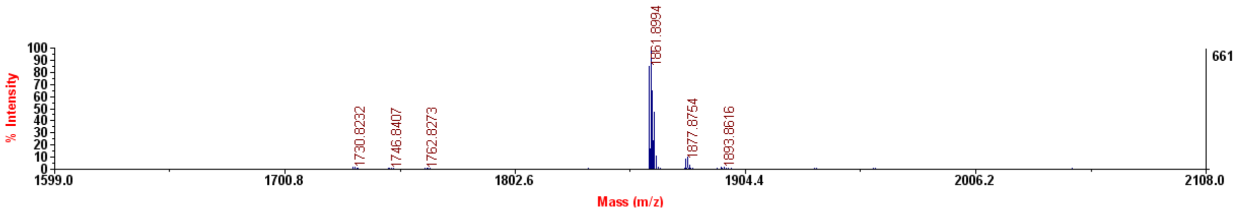

Supplementary Figure 158. MS spectra of plates 121 and 122. The data of E10, A11, B11, and F11 in plate 121 and F1 and C3 in plate 122 are shown.

plate 122/line F/column 4

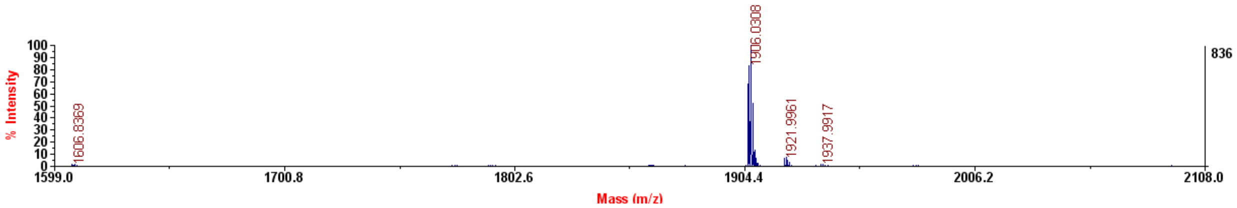

plate 127/line F/column 1

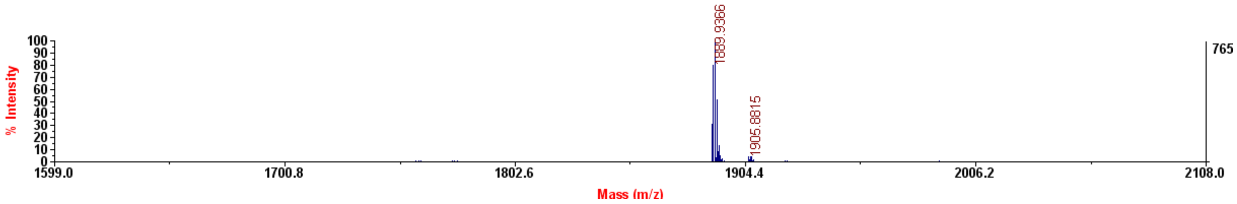

plate 127/line G/column 3

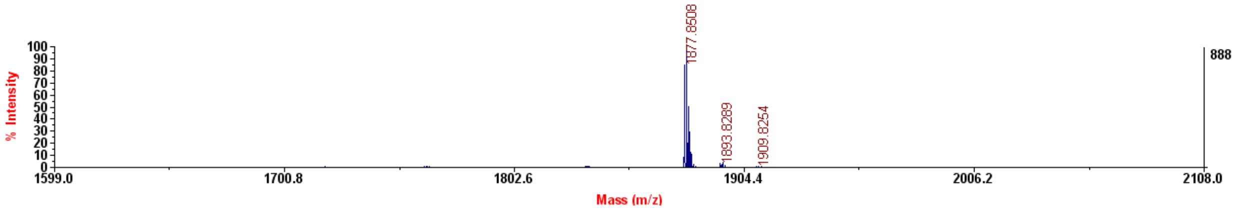

plate 127/line B/column 4

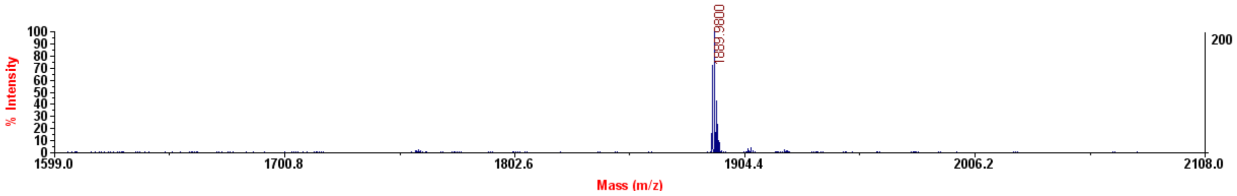

plate 127/line F/column 10

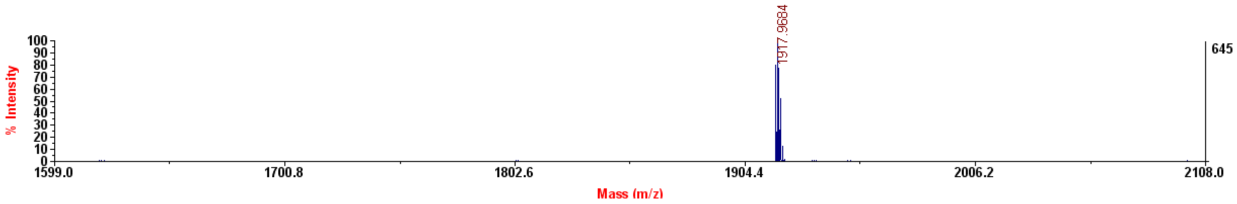

plate 127/line G/column 10

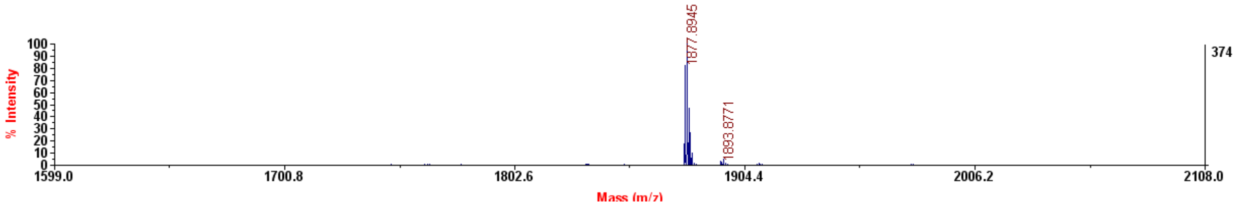

Supplementary Figure 159. MS spectra of plates 122 and 127. The data of F4 in plate 122 and F1, G3, B4, F10, and G10 in plate 127 are shown.

plate 127/line H/column 10

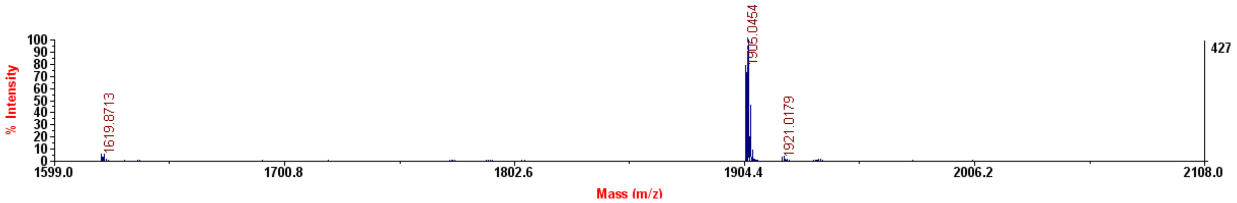

plate 128/line F/column 1

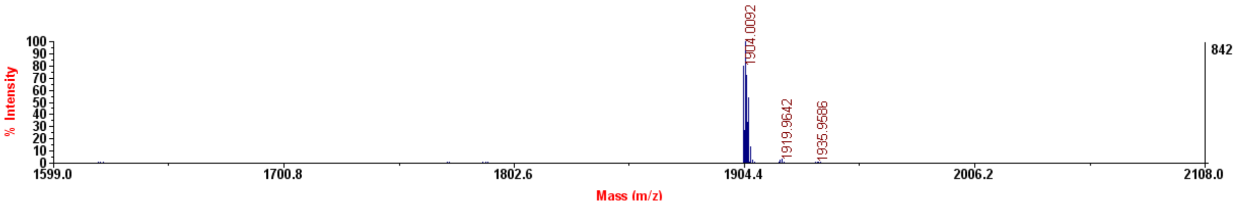

plate 128/line B/column 3

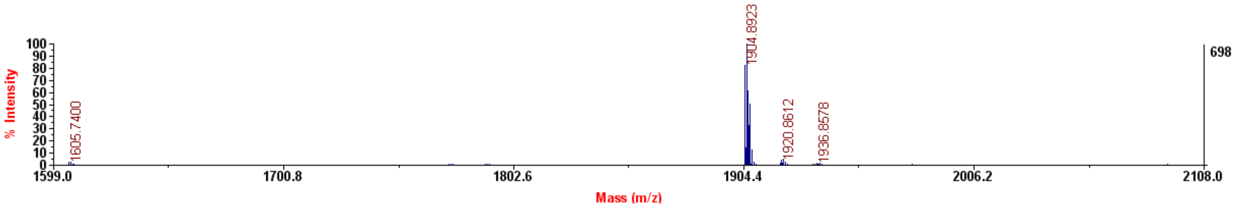

plate 128/line D/column 3

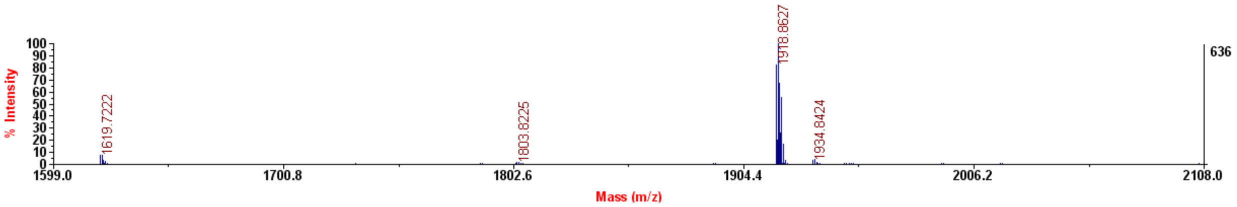

plate 128/line F/column 4

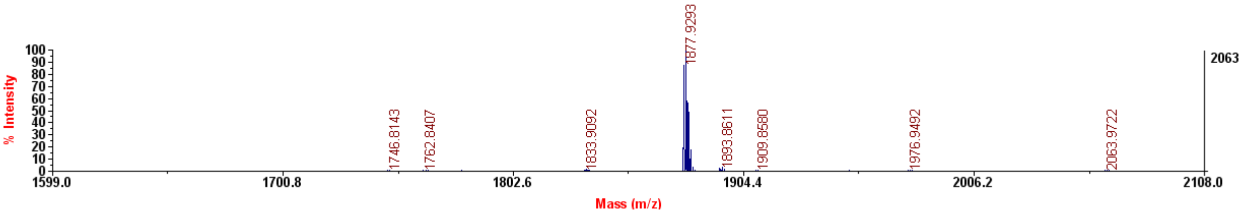

plate 128/line D/column 8

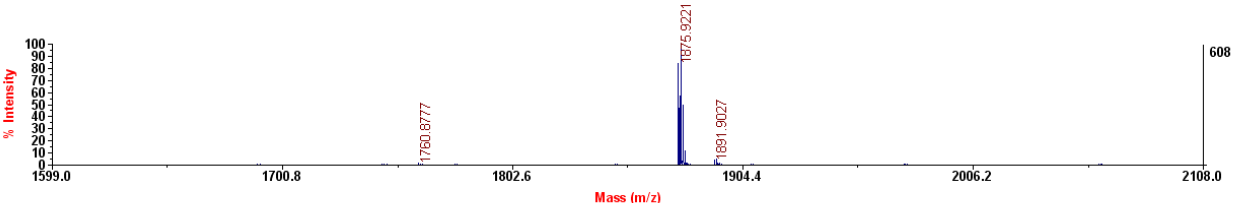

Supplementary Figure 160. MS spectra of plates 127 and 128. The data of H10 in plate 127 and F1, B3, D3, F4, and D8 in plate 128 are shown.

plate 128/line H/column 9

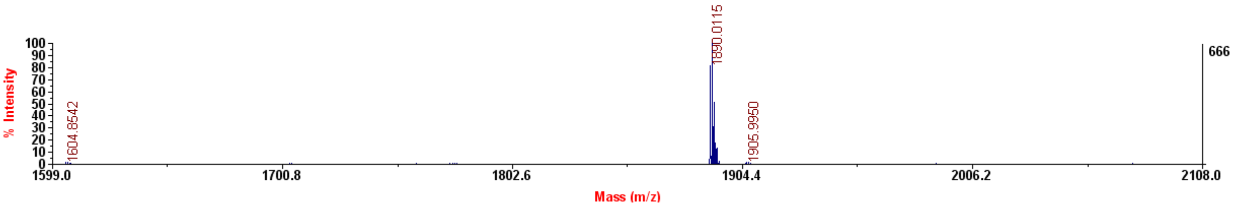

plate 128/line B/column 11

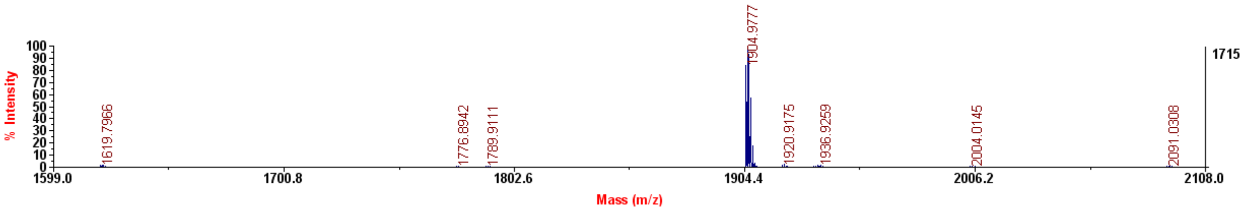

plate 128/line G/column 11

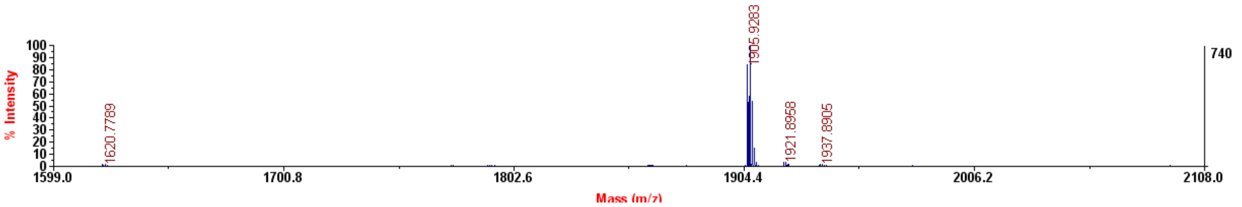

plate 129/line F/column 1

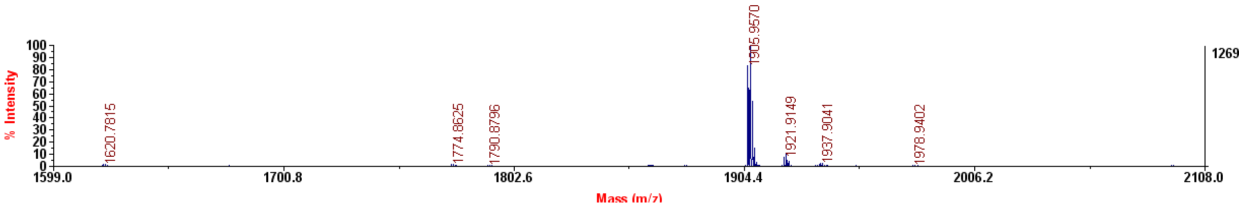

plate 129/line A/column 2

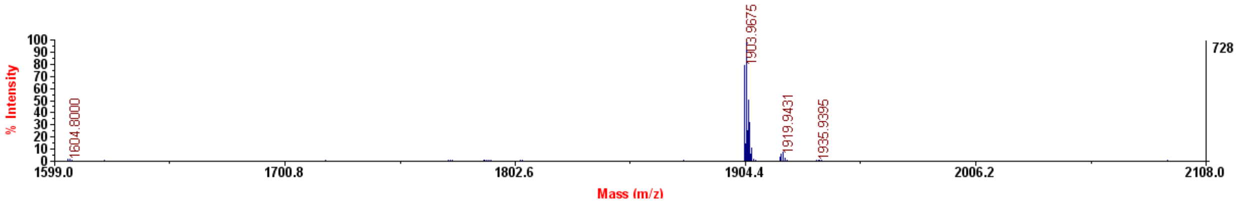

plate 129/line E/column 3

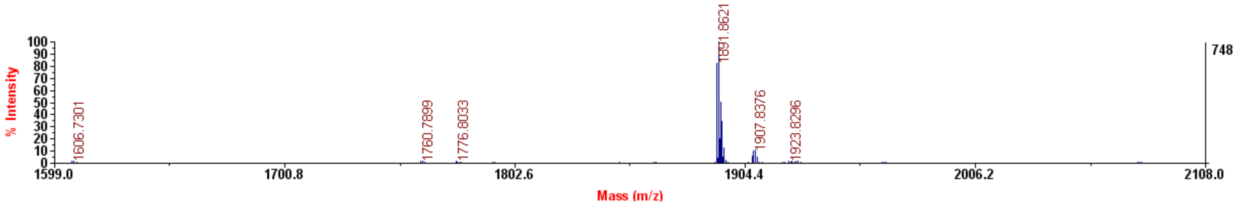

Supplementary Figure 161. MS spectra of plates 128 and 129. The data of H9, B11, and G11 in plate 128 and F1, A2, and E3 in plate 129 are shown.

plate 129/line C/column 4

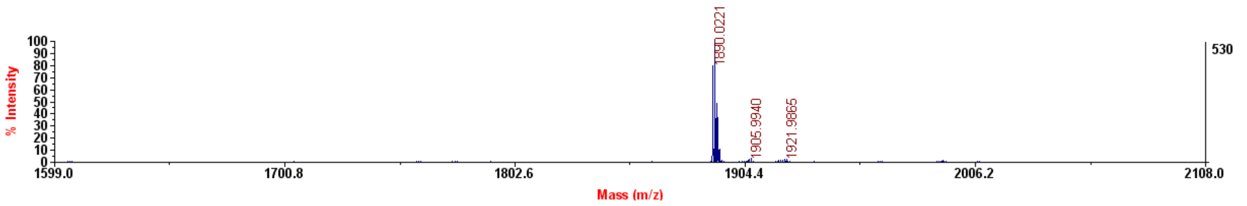

plate 129/line E/column 7

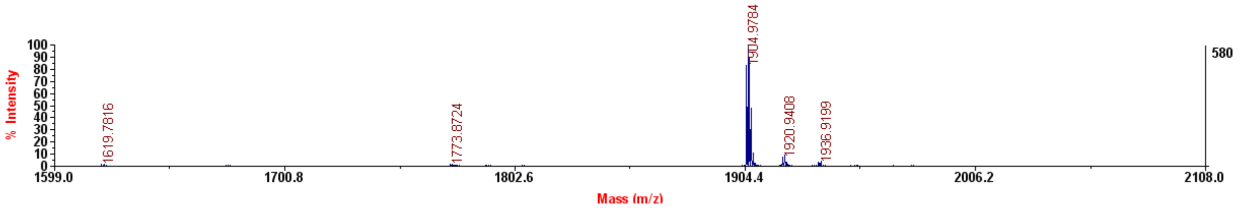

plate 129/line H/column 9

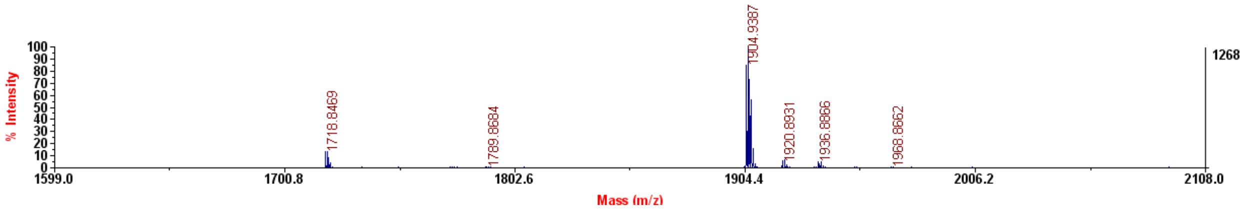

plate 129/line F/column 10

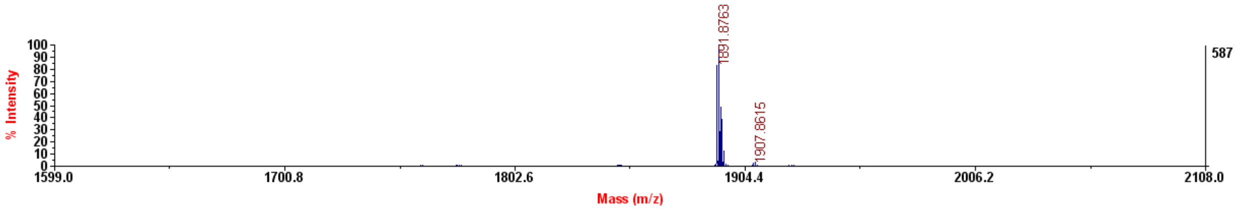

plate 129/line G/column 11

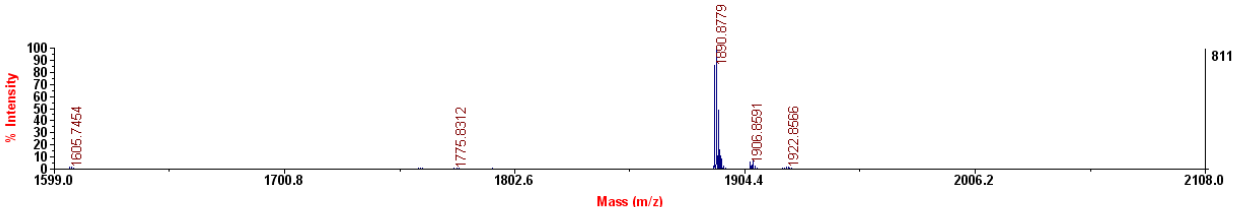

plate 130/line H/column 2

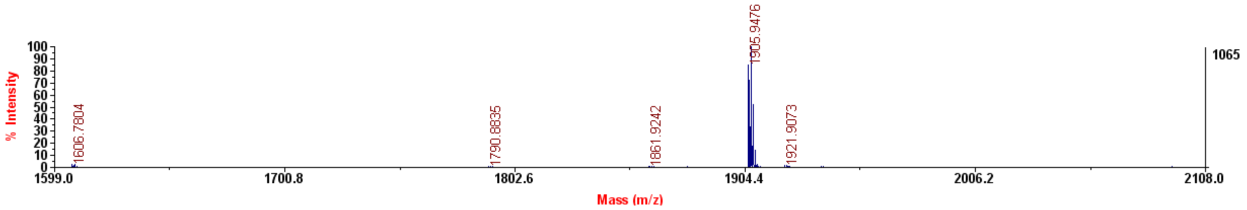

Supplementary Figure 162. MS spectra of plates 129 and 130. The data of C4, E7, H9, F10, and G11 in plate 129 and H2 in plate 130 are shown.

plate 130/line A/column 3

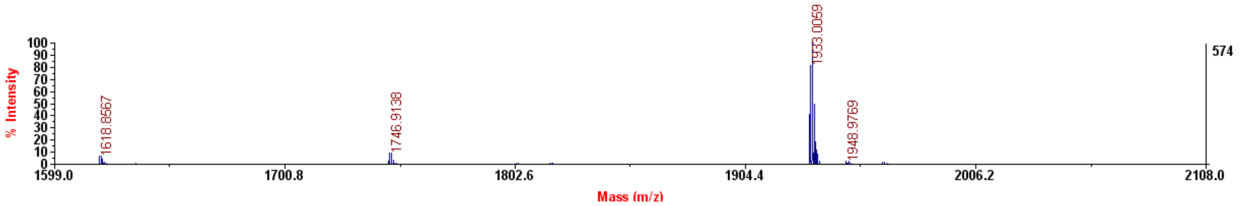

plate 130/line H/column 3

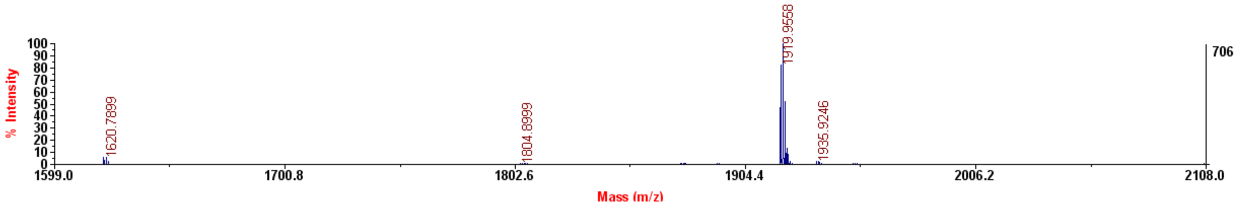

plate 130/line E/column 4

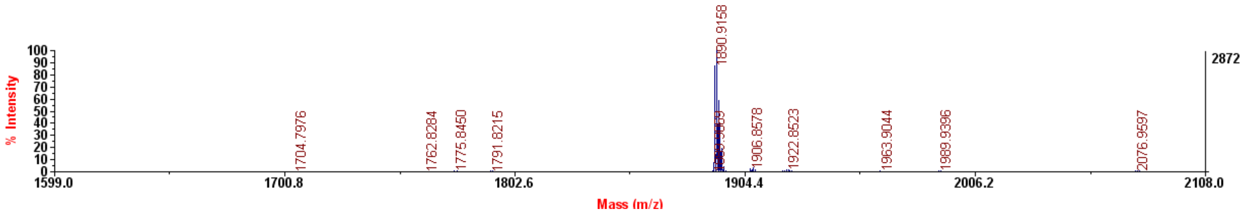

plate 130/line F/column 5

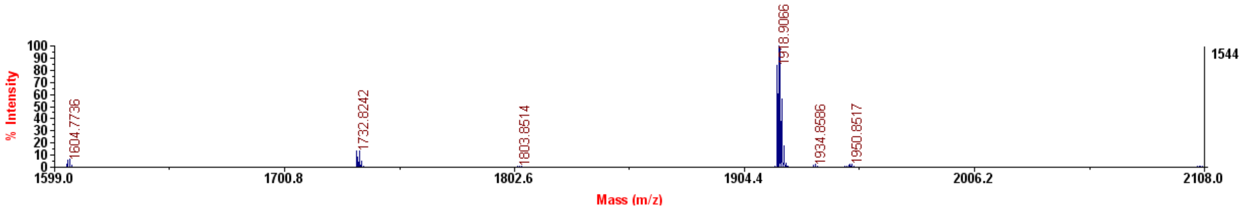

plate 130/line A/column 6

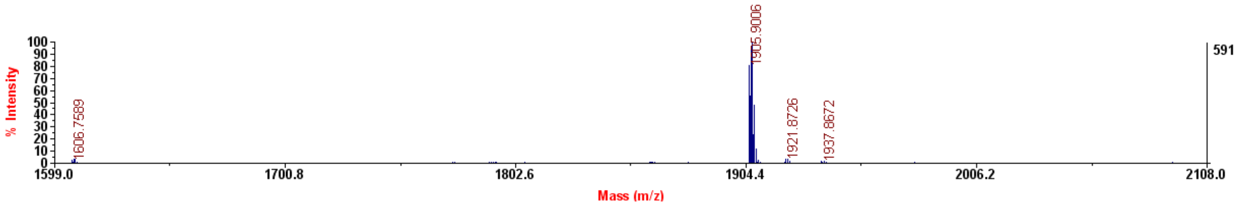

plate 130/line A/column 7

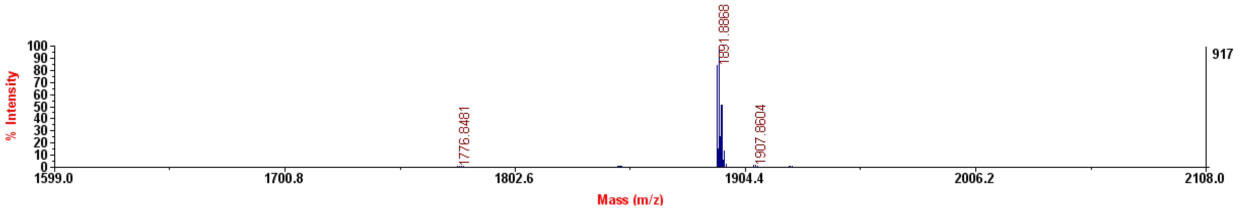

Supplementary Figure 163. MS spectra of plate 130. The data of A3, H3, E4, F5, A6, and A7 in plate 130 are shown.

plate 130/line F/column 7

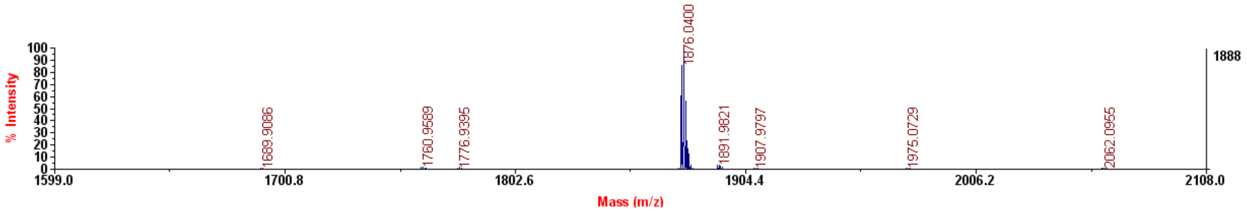

plate 130/line G/column 7

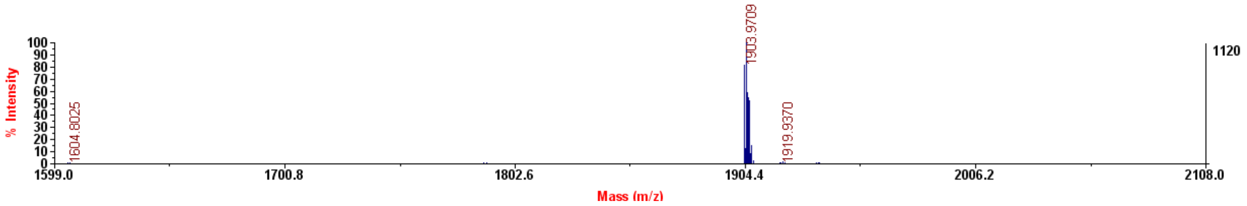

plate 130/line E/column 9

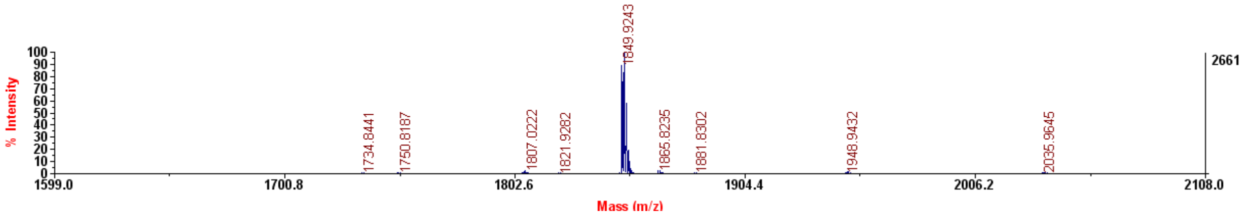

plate 132/line A/column 1

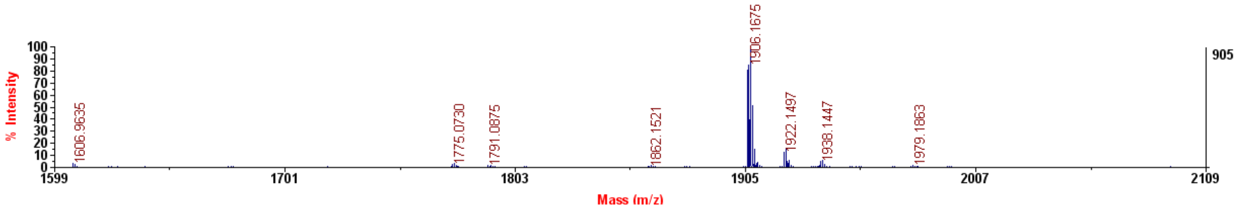

plate 132/line A/column 6

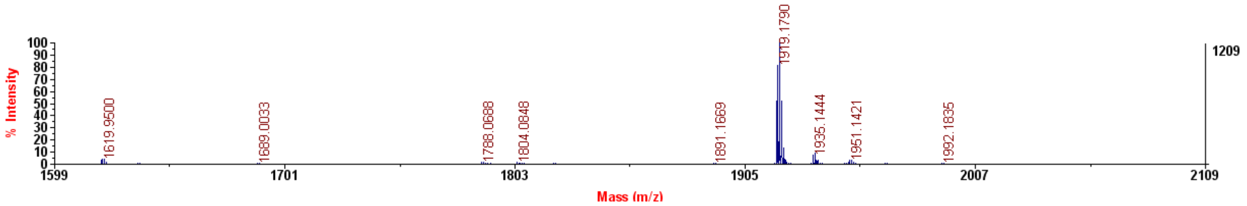

plate 132/line A/column 9

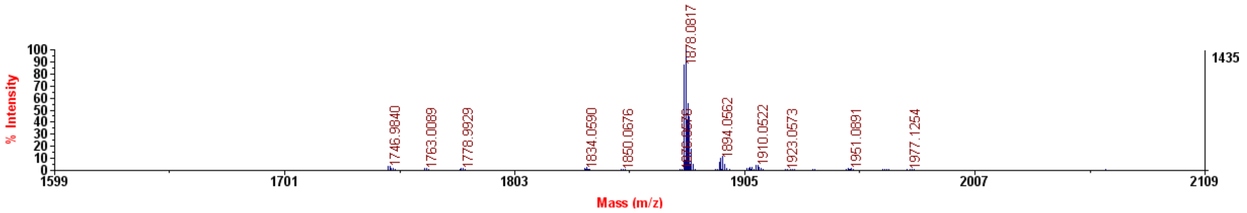

Supplementary Figure 164. MS spectra of plates 130 and 132. The data of F7, G7, and E9 in plate 130 and A1, A6, and A9 in plate 132 are shown.

plate 133/line D/column 2

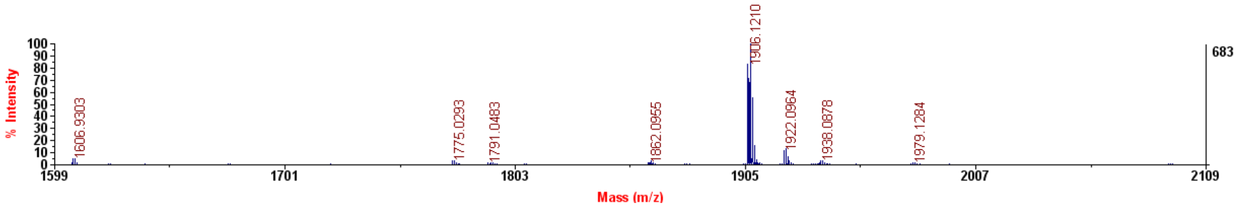

plate 134/line D/column 5

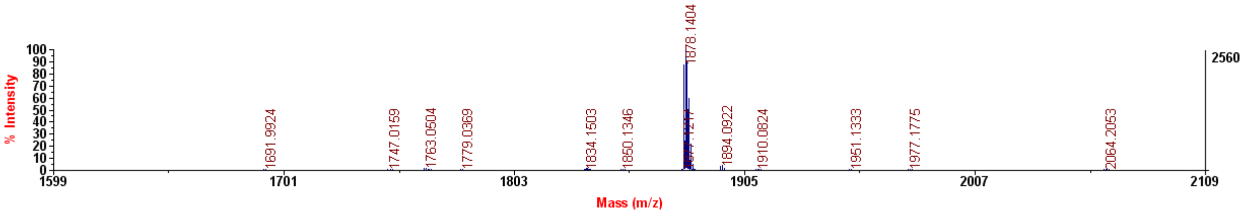

plate 134/line D/column 8

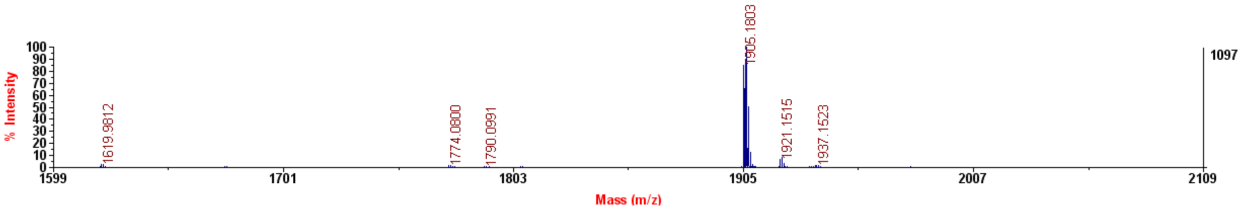

plate 137/line H/column 5

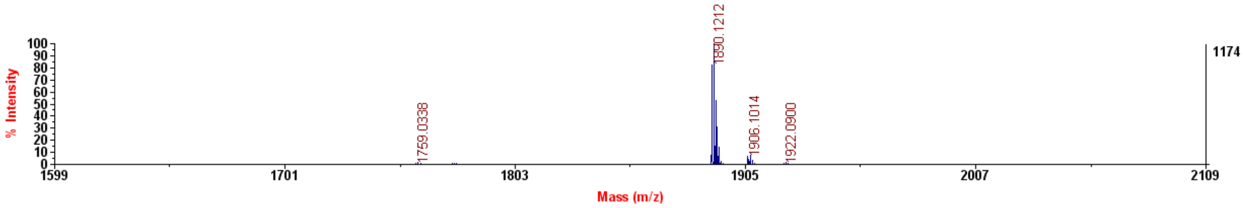

plate 137/line A/column 11

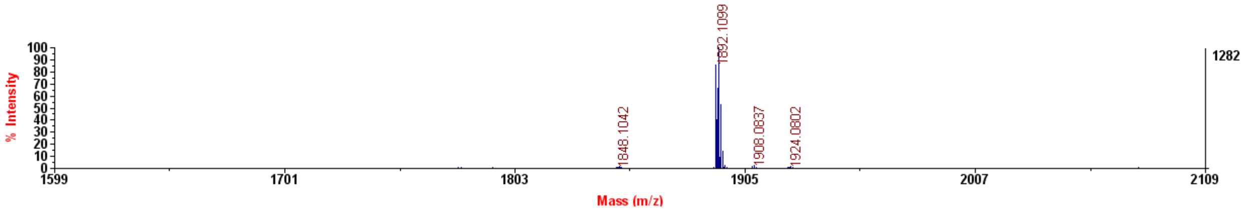

plate 138/line D/column 1

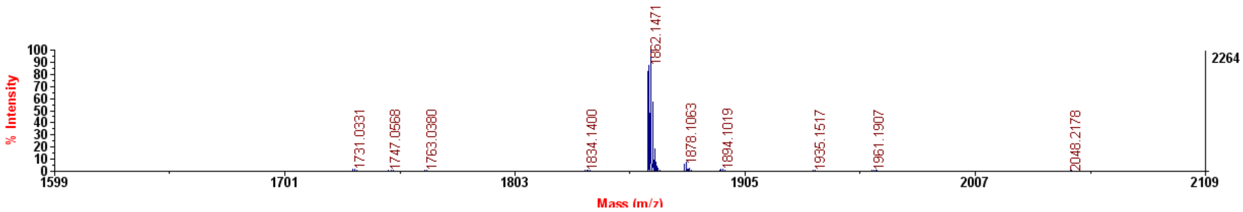

Supplementary Figure 165. MS spectra of plates 133, 134, 137, and 138. The data of D2 in plate 133, D5 and D8 in plate 134, H5 and A11 in plate 137, and D1 in plate 138 are shown.

plate 138/line C/column 2

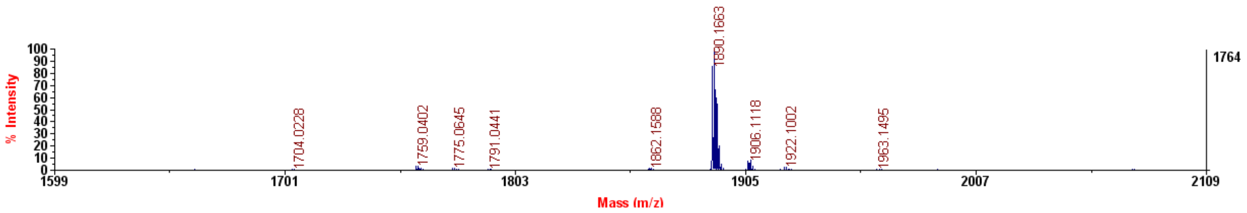

plate 139/line A/column 2

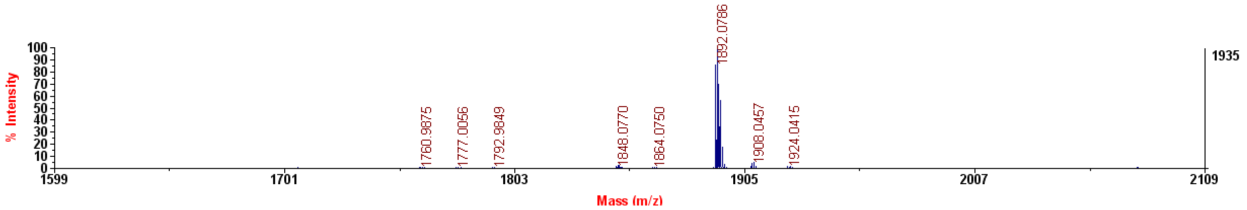

plate 140/line C/column 3

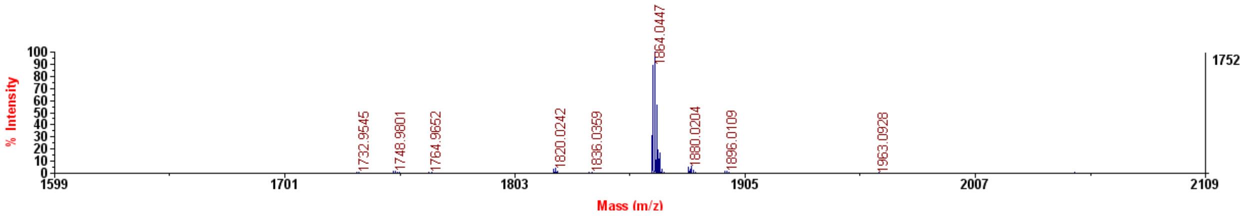

plate 140/line E/column 3

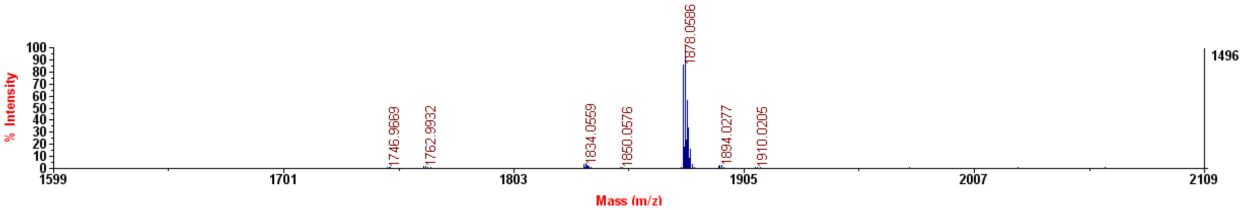

plate 140/line G/column 3

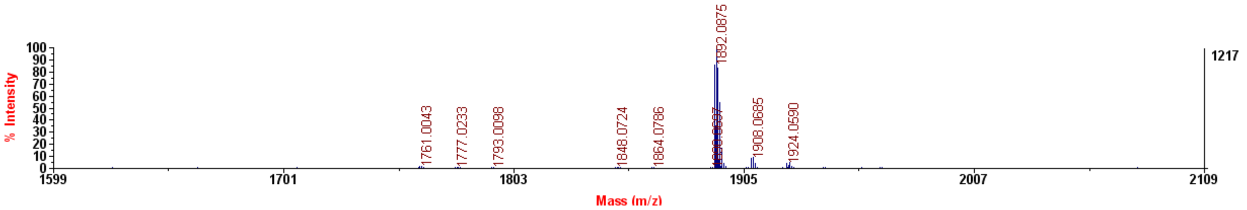

plate 140/line E/column 4

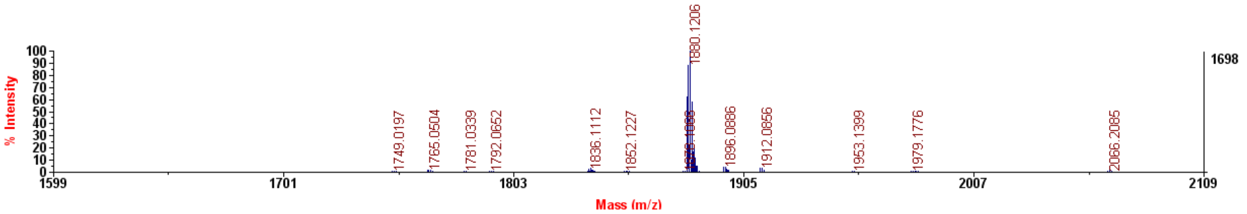

Supplementary Figure 166. MS spectra of plates 138, 139, and 140. The data of C2 in plate 138, A2 in plate 139, and C3, E3, G3, and E4 in plate 140 are shown.

plate 140/line B/column 5

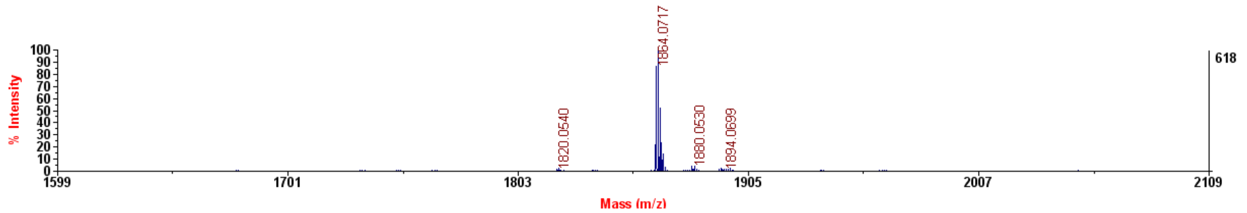

plate 140/line C/column 6

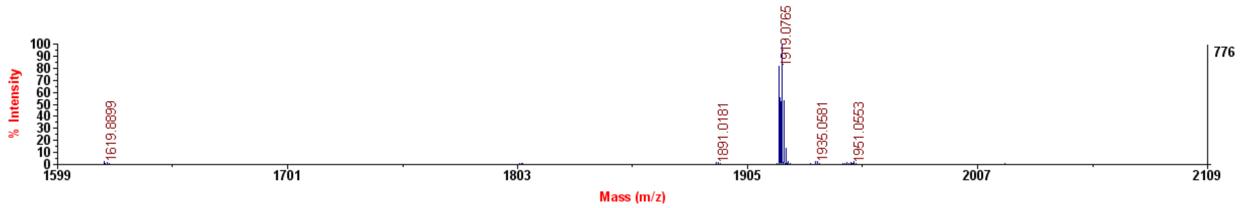

plate 141/line E/column 3

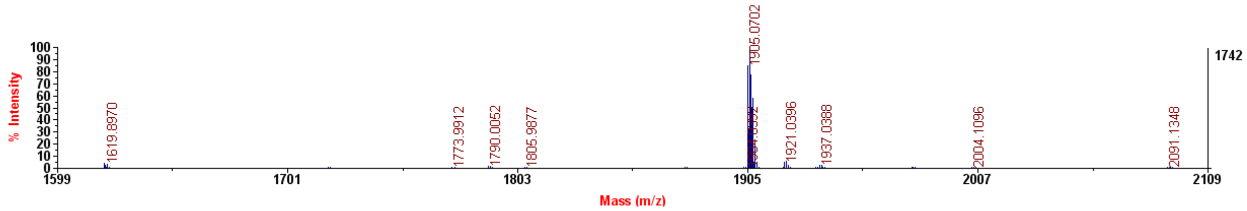

plate 141/line E/column 10

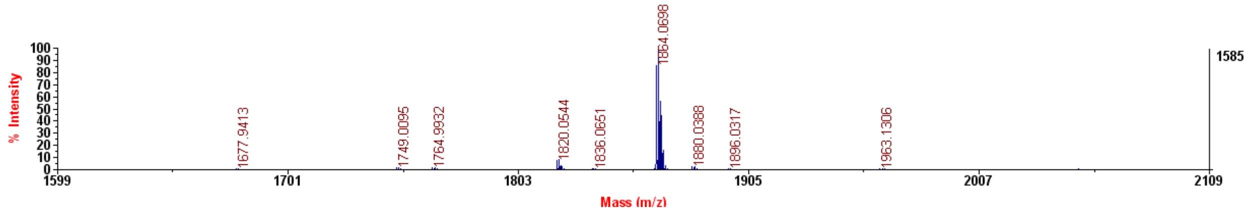

plate 141/line H/column 10

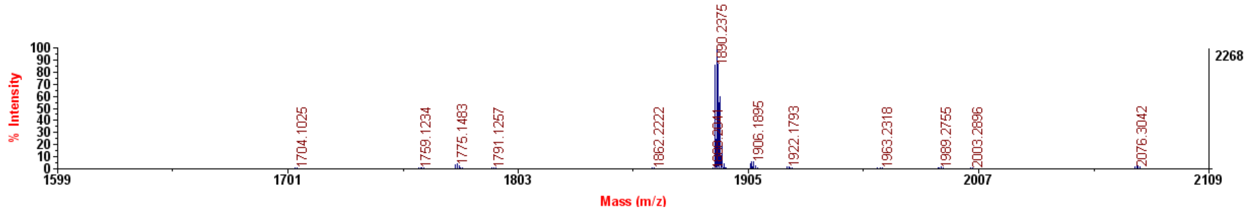

plate 143/line B/column 2

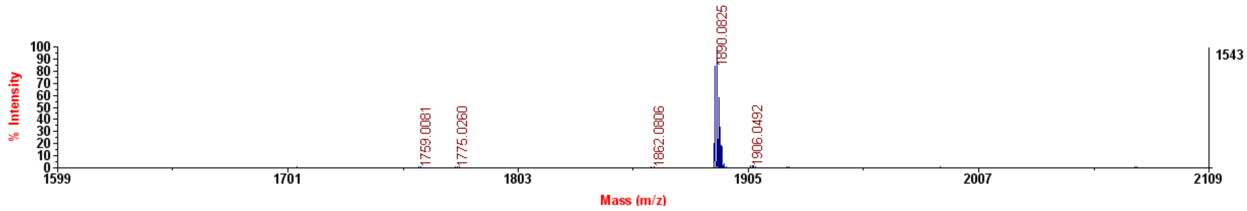

Supplementary Figure 167. MS spectra of plates 140, 141, and 143. The data of B5 and C6 in plate 140, E3, E10, and H10 in plate 141, and B2 in plate 143 are shown.

plate 143/line H/column 6

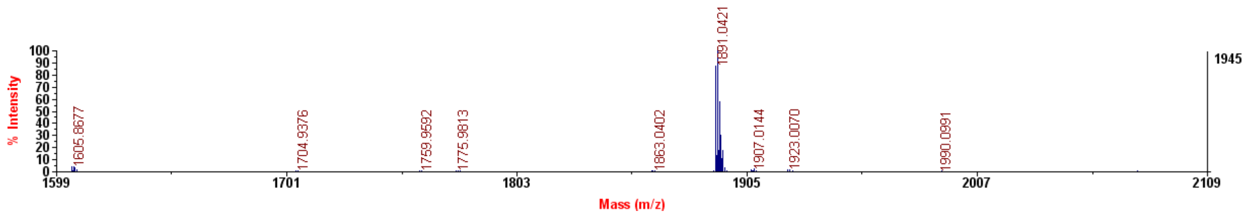

plate 143/line F/column 10

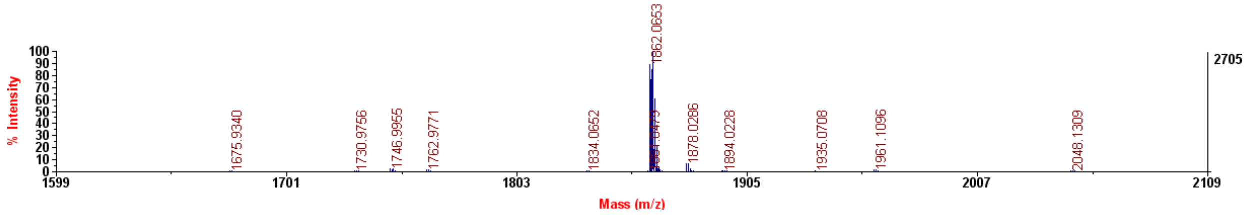

plate 144/line E/column 2

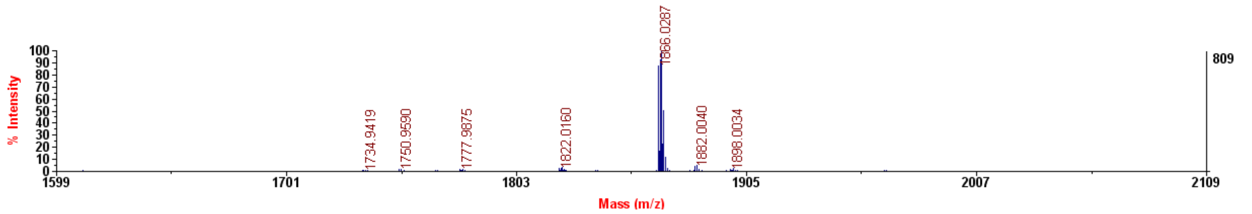

plate 144/line D/column 3

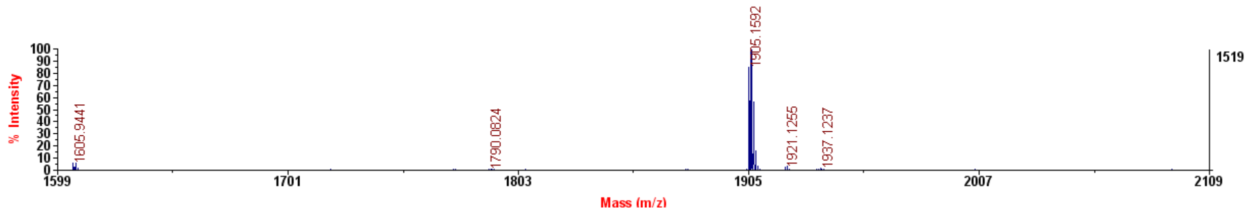

plate 144/line G/column 9

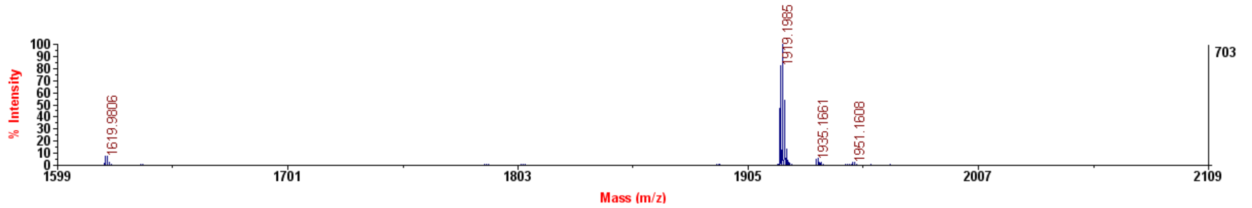

plate 144/line C/column 10

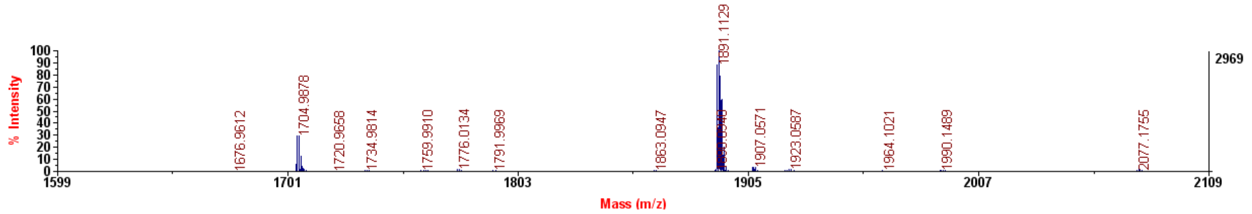

Supplementary Figure 168. MS spectra of plates 143 and 144. The data of H6 and F10 in plate 143 and E2, D3, G9, and C10 in plate 144 are shown.

plate 145/line C/column 1

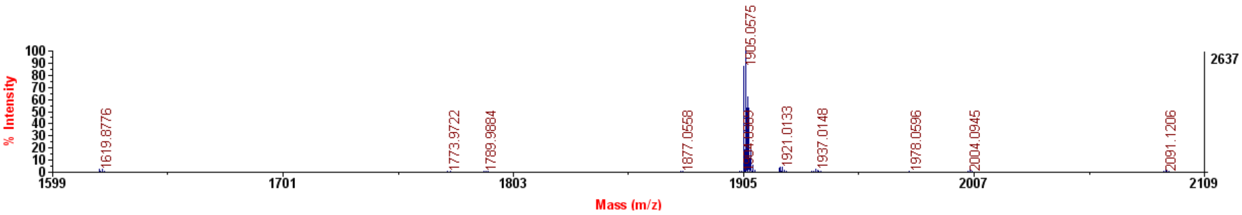

plate 145/line A/column 3

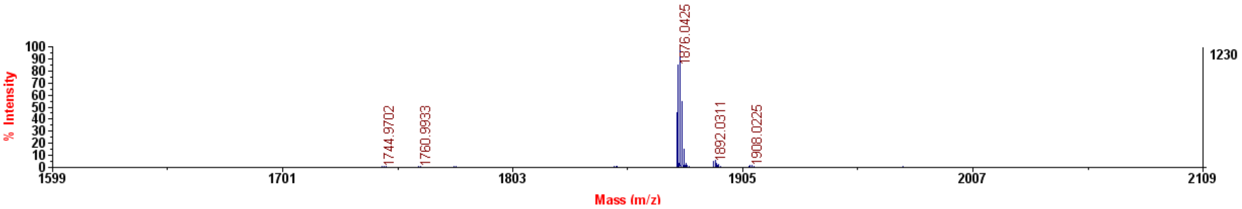

plate 145/line E/column 11

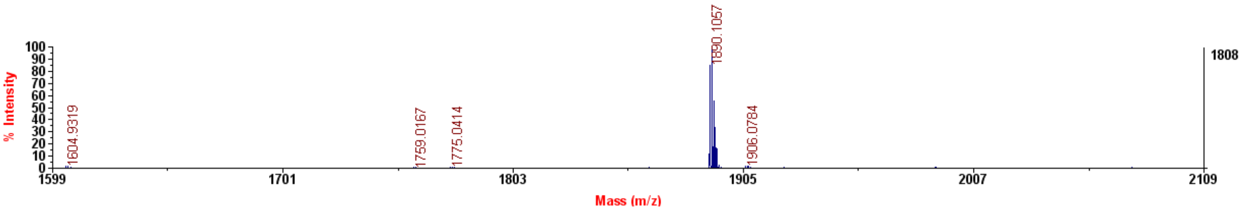

plate 146/line A/column 10

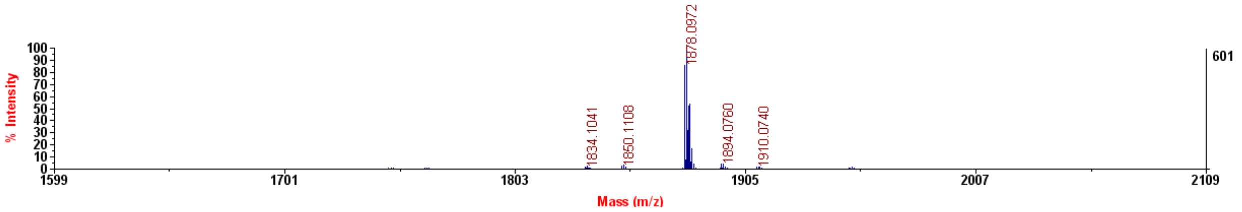

plate 146/line E/column 11

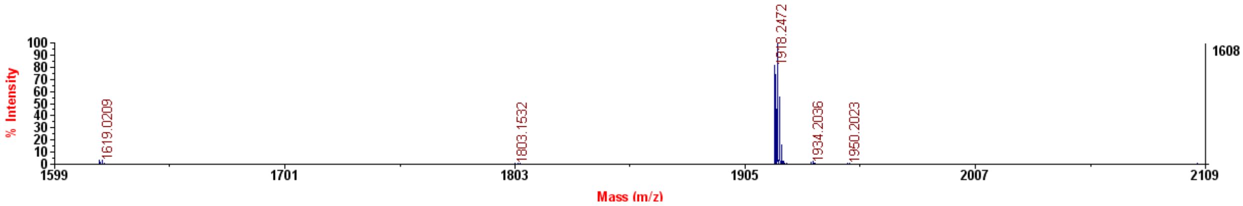

plate 147/line B/column 8

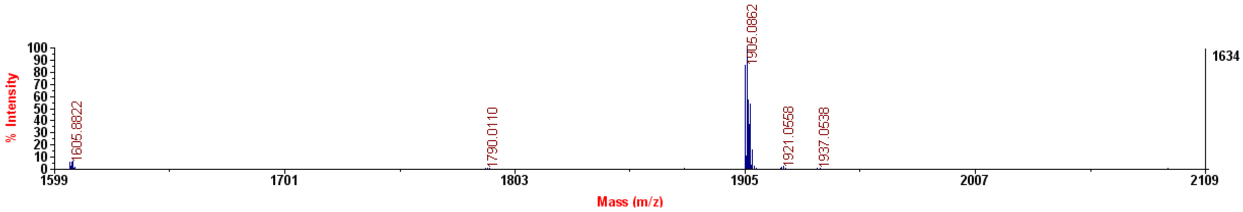

Supplementary Figure 169. MS spectra of plates 145, 146, and 147. The data of C1, A3, and E11 in plate 145, A10 and E11 in plate 146, and B8 in plate 147 are shown.

plate 147/line C/column 9

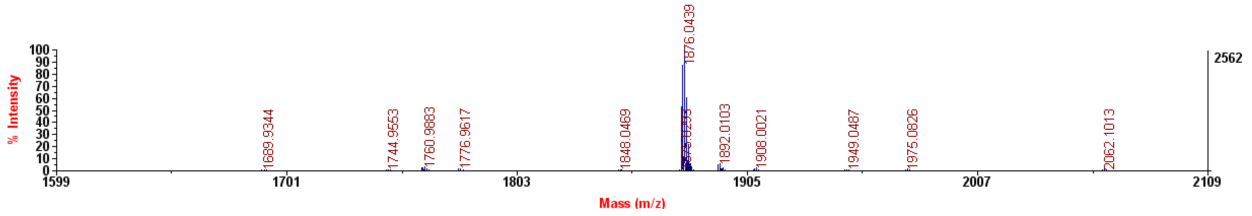

plate 147/line F/column 9

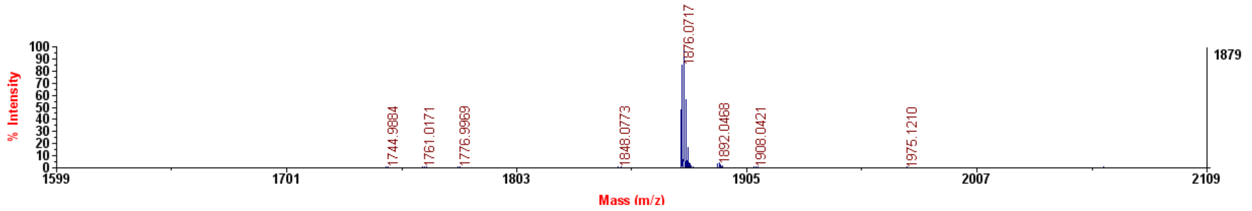

plate 148/line D/column 1

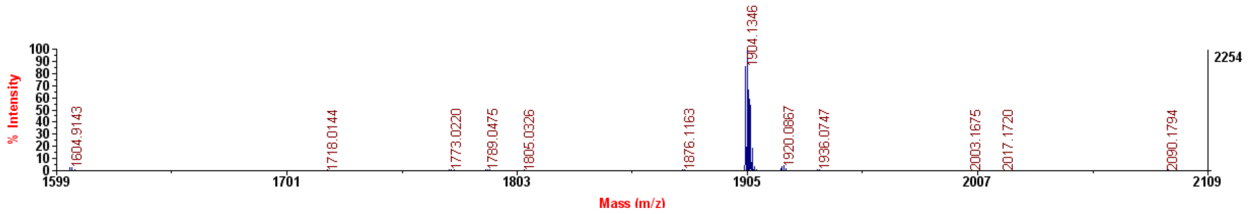

plate 148/line G/column 1

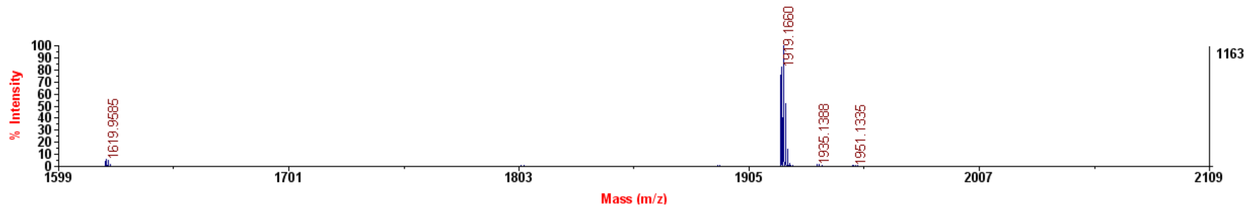

plate 148/line H/column 2

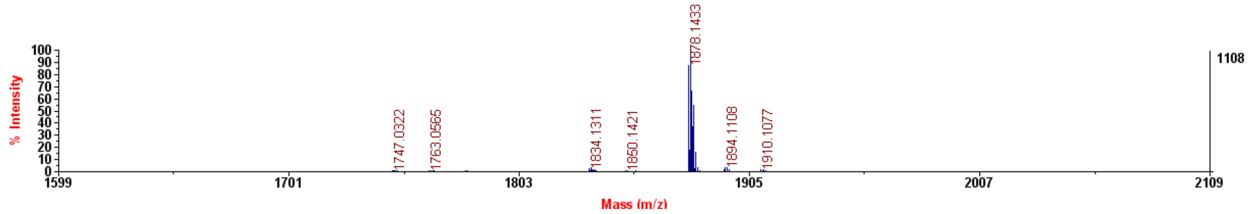

plate 148/line E/column 3

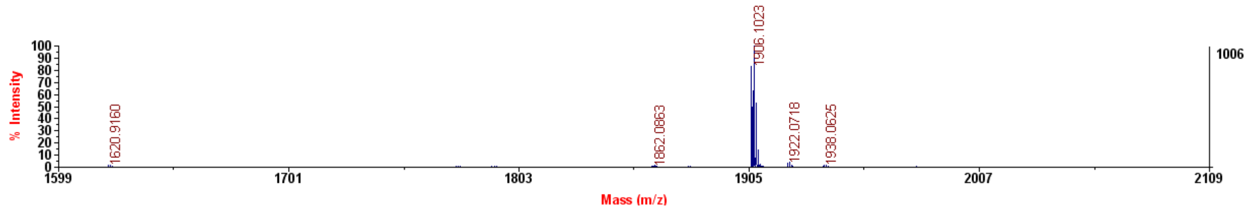

Supplementary Figure 170. MS spectra of plates 147 and 148. The data of C9 and F9 in plate 147 and D1, G1, H2, and E3 in plate 148 are shown.

plate 148/line B/column 5

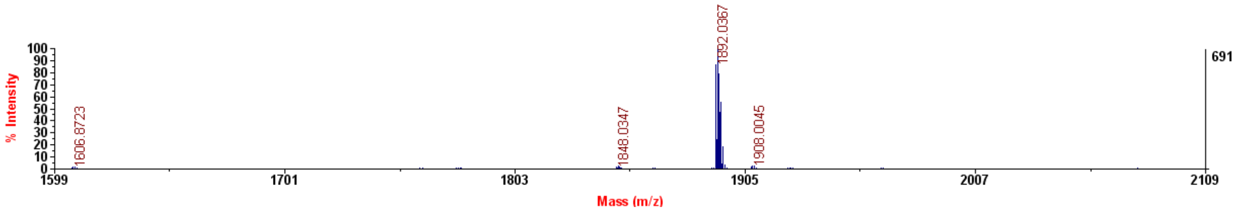

plate 148/line F/column 8

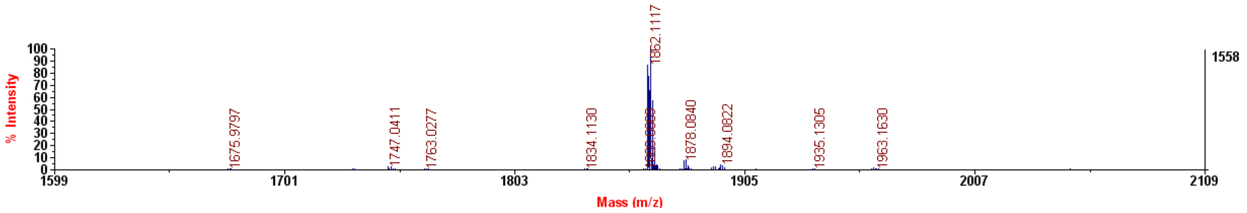

plate 148/line A/column 9

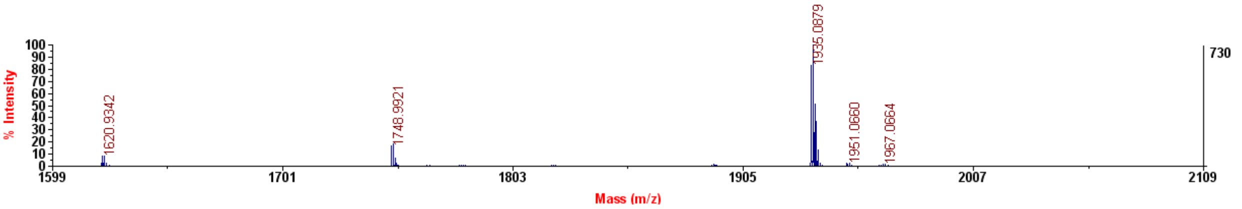

plate 148/line H/column 9

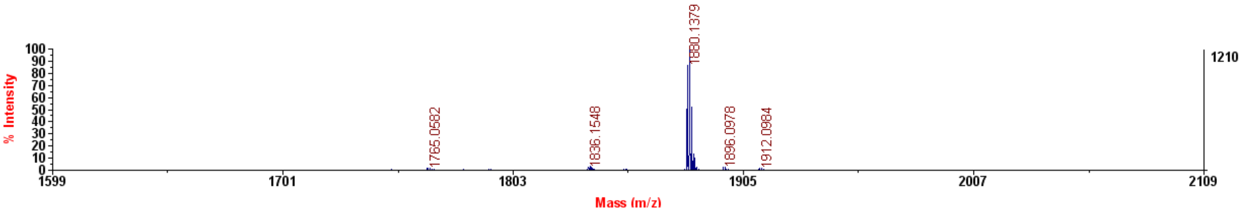

plate 149/line F/column 1

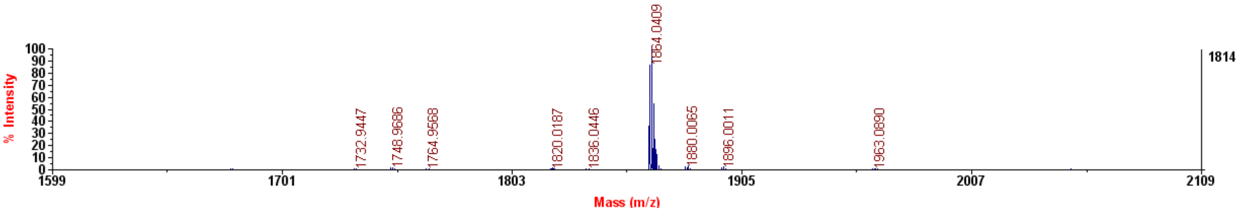

plate 149/line C/column 9

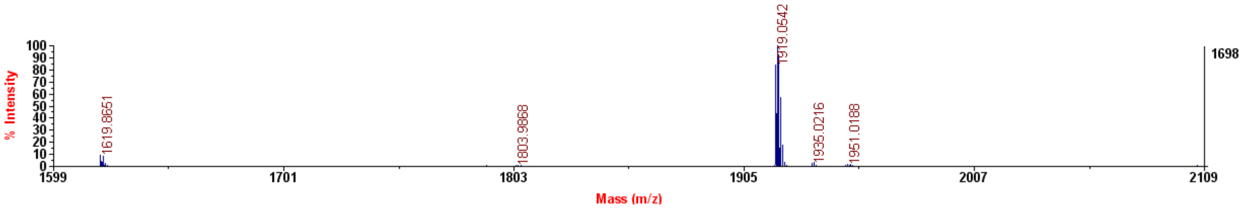

Supplementary Figure 171. MS spectra of plates 148 and 149. The data of B5, F8, A9, and H9 in plate 148 and F1 and C9 in plate 149 are shown.

plate 149/line H/column 9

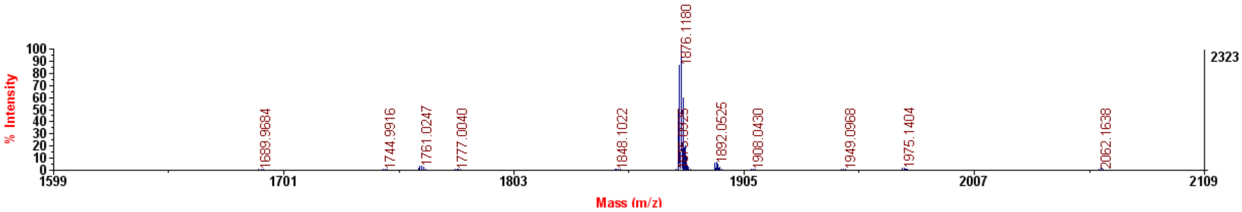

plate 150/line C/column 1

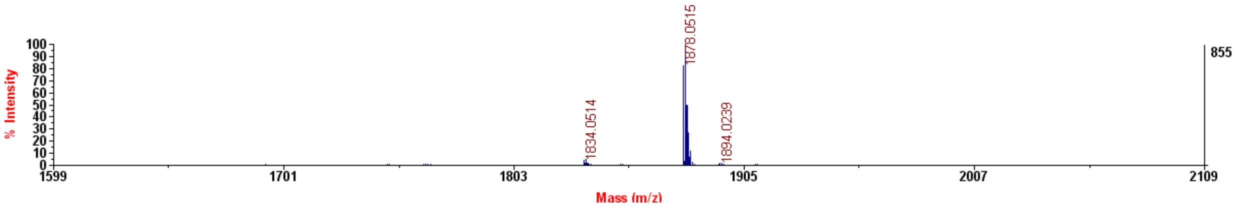

plate 150/line D/column 1

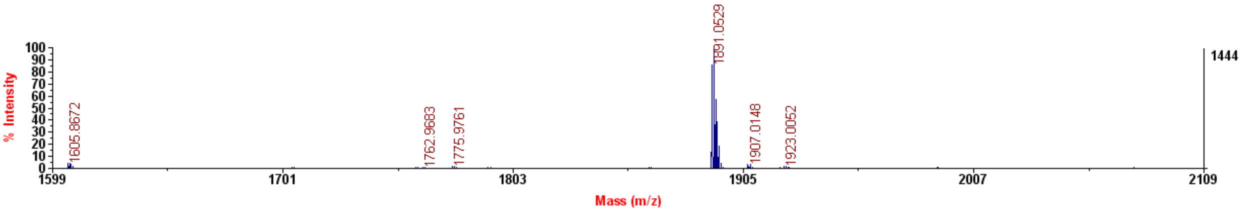

plate 150/line G/column 1

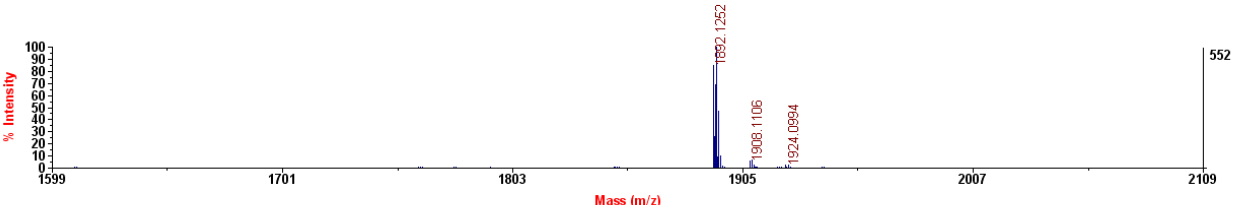

plate 150/line F/column 4

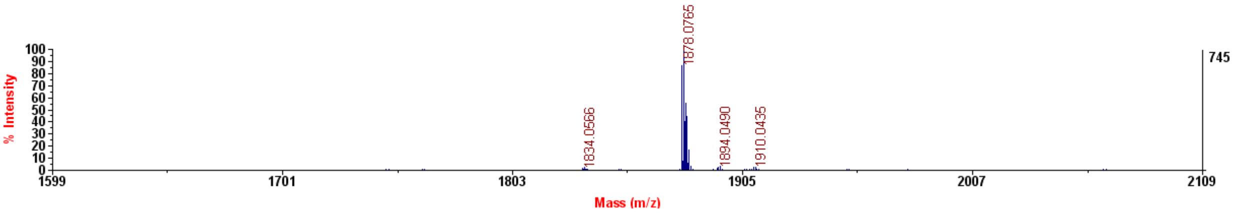

plate 150/line G/column 6

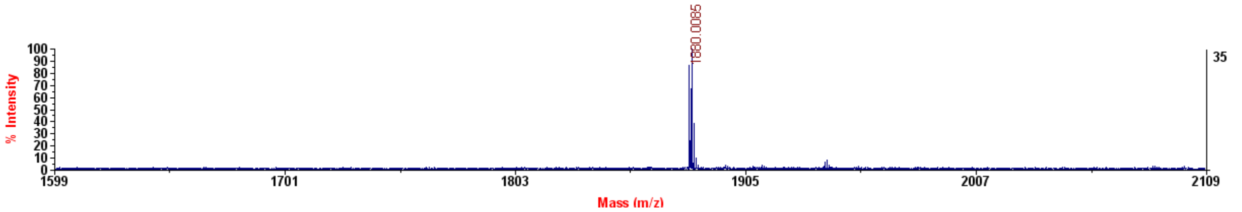

Supplementary Figure 172. MS spectra of plates 149 and 150. The data of H9 in plate 149 and C1, D1, G1, F4, and G6 in plate 150 are shown.

plate 151/line E/column 2

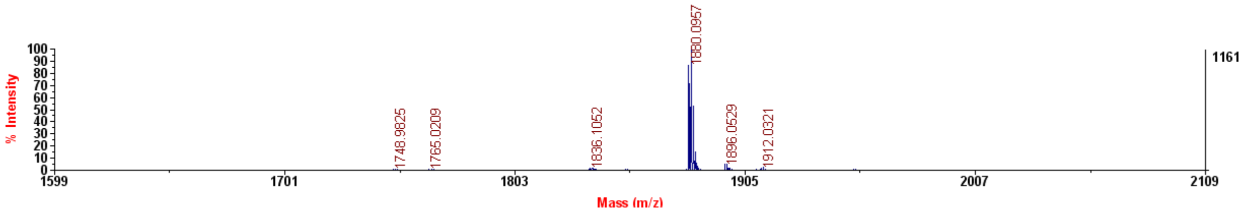

plate 151/line D/column 3

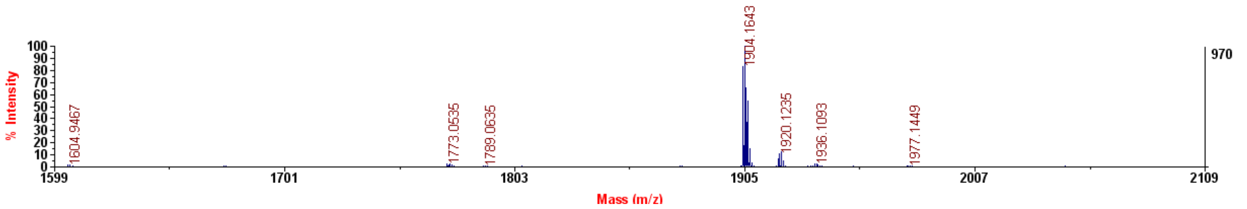

plate 151/line D/column 5

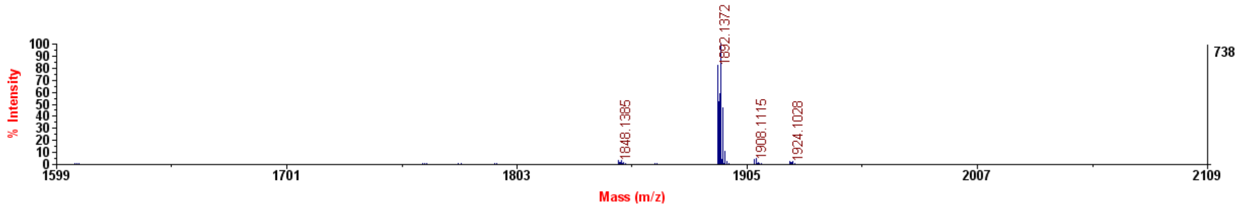

plate 151/line A/column 6

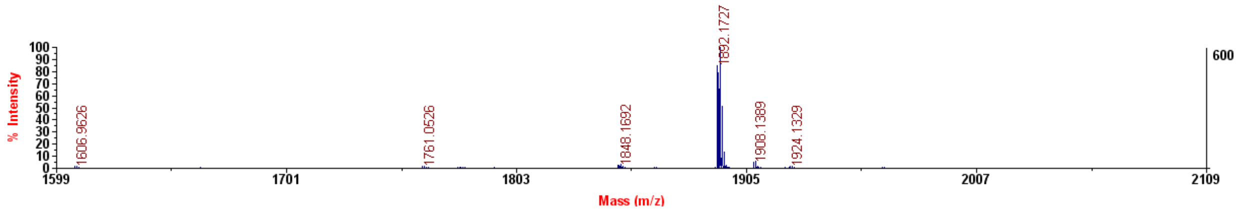

plate 151/line E/column 11

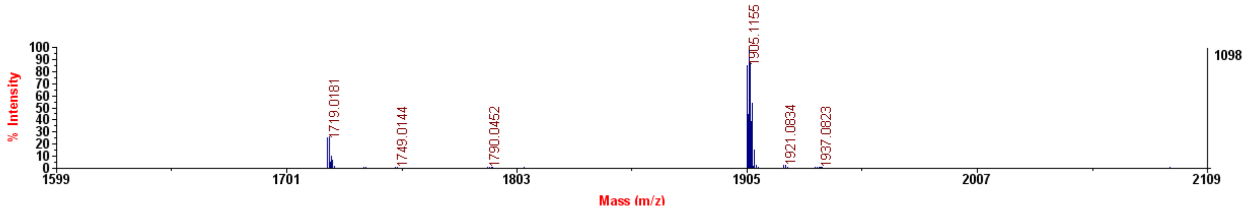

Supplementary Figure 173. MS spectra of plate 151. The data of E2, D3, D5, A6, and E11 in plate 151 are shown.

Supplementary References

1. Clement, N. R. & Gould, J. M. Pyranine (8-hydroxy-1,3,6-pyrenetrisulfonate) as a probe of internal aqueous hydrogen ion concentration in phospholipid vesicles. *Biochemistry* **20,** 1534–1538 (1981).

2. Aguedo, M., Waché, Y. & Belin, J.-M. Intracellular pH-dependent efflux of the fluorescent probe pyranine in the yeast *Yarrowia lipolytica*. *FEMS Microbiol. Lett.* **200,** 185–189 (2001).

3. Otis, F., Racine-Berthiaume, C. & Voyer, N. How far can a sodium ion travel within a lipid bilayer? *J. Am. Chem. Soc.* **133,** 6481–6483 (2011).

4. R Core Team. R: A language and environment for statistical computing. R Foundation for Statistical Computing, Vienna, Austria. URL http://www.R-project.org/ (2018).

5. Ritz, C., Baty, F., Streibig, J. C. & Gerhard, D. Dose-response analysis using R. *PLOS ONE* **10,** e0146021 (2015).

6. Clinical and Laboratory Standards Institute. Methods for dilution antimicrobial susceptibility tests for bacteria that grow aerobically; approved standard—eighth edition (CLSI document M07–A8). Clinical and Laboratory Standards Institute, Wayne, PA (2009).
