# Supplementary material for: Discovery of gramicidin A analogues with altered activities by multidimensional screening of a one-bead-one-compound library
Source: Nat Commun. 2020 Oct 1;11:4935. doi: 10.1038/s41467-020-18711-2 (PMC7531004; doi:10.1038/s41467-020-18711-2)
Supplement: Supplementary file 2 — Reporting Summary [file 41467_2020_18711_MOESM2_ESM.pdf]

nature research

Corresponding author(s): Masayuki Inoue

Last updated by author(s): Sep 1, 2020

Reporting Summary

Nature Research wishes to improve the reproducibility of the work that we publish. This form provides structure for consistency and transparency in reporting. For further information on Nature Research policies, see our Editorial Policies and the Editorial Policy Checklist.

Statistics

For all statistical analyses, confirm that the following items are present in the figure legend, table legend, main text, or Methods section.

n/a	Confirmed	
☐	☒	The exact sample size (n) for each experimental group/condition, given as a discrete number and unit of measurement
☐	☒	A statement on whether measurements were taken from distinct samples or whether the same sample was measured repeatedly
☒	☐	The statistical test(s) used AND whether they are one- or two-sided<br>*Only common tests should be described solely by name; describe more complex techniques in the Methods section.*
☒	☐	A description of all covariates tested
☒	☐	A description of any assumptions or corrections, such as tests of normality and adjustment for multiple comparisons
☐	☒	A full description of the statistical parameters including central tendency (e.g. means) or other basic estimates (e.g. regression coefficient) AND variation (e.g. standard deviation) or associated estimates of uncertainty (e.g. confidence intervals)
☒	☐	For null hypothesis testing, the test statistic (e.g. F, t, r) with confidence intervals, effect sizes, degrees of freedom and P value noted<br>*Give P values as exact values whenever suitable.*
☒	☐	For Bayesian analysis, information on the choice of priors and Markov chain Monte Carlo settings
☒	☐	For hierarchical and complex designs, identification of the appropriate level for tests and full reporting of outcomes
☒	☐	Estimates of effect sizes (e.g. Cohen's d, Pearson's r), indicating how they were calculated

Our web collection on statistics for biologists contains articles on many of the points above.

Software and code

Policy information about availability of computer code

Data collection	Optical rotations were recorded on a P-2200 polarimeter (JASCO). Infrared (IR) spectra were recorded on an FT/IR-4100 spectrometer (JASCO) as a thin film on CaF2. 1H and 13C NMR spectra were recorded on an ECX 500 (500 MHz for 1H NMR, 125 MHz for 13C NMR) spectrometer (JEOL). HRMS spectra were recorded on a MicrOTOFII (Bruker Daltonics) electrospray ionization time of flight (ESI-TOF) mass spectrometer. MALDI-TOF MS analysis and MS/MS sequencing analysis were performed on a TOF/TOF 5800 system (AB Sciex). UV absorbance was measured on a UV-1800 UV-VIS spectrophotometer (Shimadzu).
Data analysis	Sigmoidal curve fittings were performed on R (version 3.6.0 or 4.0.2) with drc package or GraphPad Prism (version 4.03).

For manuscripts utilizing custom algorithms or software that are central to the research but not yet described in published literature, software must be made available to editors and reviewers. We strongly encourage code deposition in a community repository (e.g. GitHub). See the Nature Research guidelines for submitting code & software for further information.

Data

Policy information about availability of data

All manuscripts must include a data availability statement. This statement should provide the following information, where applicable:

- Accession codes, unique identifiers, or web links for publicly available datasets
- A list of figures that have associated raw data
- A description of any restrictions on data availability

The data that support the findings of this study are available from the corresponding author upon reasonable request. The source data underlying Figure 3a, Supplementary Figure 1, 5, 6, and 7, and Supplementary Table 14 are provided with this paper.

Field-specific reporting

Please select the one below that is the best fit for your research. If you are not sure, read the appropriate sections before making your selection.

- [x] Life sciences
- [ ] Behavioural & social sciences
- [ ] Ecological, evolutionary & environmental sciences

For a reference copy of the document with all sections, see nature.com/documents/nr-reporting-summary-flat.pdf

Life sciences study design

All studies must disclose on these points even when the disclosure is negative.

Sample size	The sample size (n) of each experiment was provided in the figure/table legends in the main manuscript and in supplementary information.
Data exclusions	No data were excluded from the analyses.
Replication	In the figure legends, the numbers of replications were stated. All attempts at replication were successful.
Randomization	Randomization was not relevant because of the in vitro nature of our study.
Blinding	In antibacterial assays, the performers were blinded to the compounds. In other assays, blinding was not relevant because of the unknown activities of the synthetic compounds and the in vitro nature of our study.

Reporting for specific materials, systems and methods

We require information from authors about some types of materials, experimental systems and methods used in many studies. Here, indicate whether each material, system or method listed is relevant to your study. If you are not sure if a list item applies to your research, read the appropriate section before selecting a response.

Materials & experimental systems

n/a	Involved in the study	
x		Antibodies
	x	Eukaryotic cell lines
x		Palaeontology and archaeology
x		Animals and other organisms
x		Human research participants
x		Clinical data
x		Dual use research of concern

Methods

n/a	Involved in the study	
x		ChIP-seq
x		Flow cytometry
x		MRI-based neuroimaging

Eukaryotic cell lines

Policy information about cell lines

Cell line source(s)	Mouse leukemia cell line P388 was obtained from Institute of Development Aging and Cancer (Tohoku University).
Authentication	None of the cell line was authenticated beyond what was performed by Institute of Development Aging and Cancer.
Mycoplasma contamination	The cells were originally confirmed negative for mycoplasma. The early passage cells were used for the experiments.
Commonly misidentified lines (See ICLAC register)	No commonly misidentified cell line was used in this study.
